# Platelet-Rich Fibrin Increases BMP2 Expression in Oral Fibroblasts via Activation of TGF-β Signaling

**DOI:** 10.3390/ijms22157935

**Published:** 2021-07-25

**Authors:** Zahra Kargarpour, Jila Nasirzade, Layla Panahipour, Goran Mitulović, Richard J. Miron, Reinhard Gruber

**Affiliations:** 1 Department of Oral Biology, Medical University of Vienna, 1090 Vienna, Austria; zahra.kargarpooresfahani@meduniwien.ac.at (Z.K.); jila.nasirzaderajiri@meduniwien.ac.at (J.N.); layla.panahipour@meduniwien.ac.at (L.P.); 2 Clinical Department of Laboratory Medicine Proteomics Core Facility, Medical University Vienna, 1090 Vienna, Austria; goran.mitulovic@meduniwien.ac.at; 3 Department of Periodontology, School of Dental Medicine, University of Bern, 3010 Bern, Switzerland; richard.miron@zmk.unibe.ch

**Keywords:** platelet-rich fibrin, proteomics, BMP2, bone regeneration, platelet-poor plasma, buffy coat

## Abstract

Solid platelet-rich fibrin (PRF), consisting of coagulated plasma from fractionated blood, has been proposed to be a suitable carrier for recombinant bone morphogenetic protein 2 (BMP2) to target mesenchymal cells during bone regeneration. However, whether solid PRF can increase the expression of BMPs in mesenchymal cells remains unknown. Proteomics analysis confirmed the presence of TGF-β1 but not BMP2 in PRF lysates. According to the existing knowledge of recombinant TGF-β1, we hypothesized that PRF can increase BMP2 expression in mesenchymal cells. To test this hypothesis, we blocked TGF-β receptor 1 kinase with SB431542 in gingival fibroblasts exposed to PRF lysates. RT-PCR and immunoassays confirmed that solid PRF lysates caused a robust SB431542-dependent increase in BMP2 expression in gingival fibroblasts. Additionally, fractions of liquid PRF, namely platelet-poor plasma (PPP) and the buffy coat (BC) layer, but not heat-denatured PPP (Alb-gel), greatly induced the expression of BMP2 in gingival fibroblasts. Even though PRF has no detectable BMPs, PRF lysates similar to recombinant TGF-β1 had the capacity to provoke canonical BMP signaling, as indicated by the nuclear translocation of Smad1/5 and the increase in its phosphorylation. Taken together, our data suggest that PRF can activate TGF-β receptor 1 kinase and consequently induce the production of BMP2 in cells of the mesenchymal lineage.

## 1. Introduction

Platelet-rich fibrin (PRF) is generated by centrifuging blood, aiming not only to remove erythrocytes but also to obtain a fraction consisting of plasma enriched with platelets and leucocytes. Since the introduction of PRF use in 2006 [1], numerous protocols have been established with the goal of optimizing the ratio of platelet counts and the overall yield of the PRF fraction generated [2]. The protocols for preparing solid PRF consider the selection of blood collection tubes containing clot activators such as silica and silicone [3]. PRF membranes are produced by compressing the solid PRF clot so that the serum is removed. PRF membranes are then applied clinically to defects with the overall goal of supporting the natural processes of wound healing and bone regeneration [4,5], which require the coordinated action of growth factors, including those released from solid PRF membranes [6,7].

Apart from its intrinsic growth factors, solid PRF can serve as a carrier for the delivery of recombinant growth factors [8]. Among these proposed growth factors is bone morphogenetic protein (BMP2), a member of the transforming growth factor beta (TGF-β) superfamily that is characterized by its osteoinductive potential [8,9]. Based on FDA approval, recombinant BMP2 has been applied together with an absorbable collagen sponge into defect sites during spinal surgery [10] and bone augmentation during regenerative dentistry [11]. To combine the favorable healing properties of PRF with the osteoinduction potential of BMP2, various combinations have been introduced. Applying the combination of BMP2 and L-PRF leads to the early resolution of medication-related osteonecrosis of the jaws (MRONJ) [12]. L-PRF combined with open flap debridement augments the periodontal healing of chronic periodontitis patients by increasing BMP2 release [13]. Alternatively, PRF might serve as a carrier for transplanted cells producing BMP2 [14]. PRF targets pristine mesenchymal cells, becoming a potential source of BMP2 [15]. PRF from rabbits [16] and humans [17] may even release BMP2, but this finding has not been confirmed by proteomic analysis [18]. The content of TGF-β in a PRF preparation, however, could be responsible for its clinical activity. Therefore, the question of whether PRF can stimulate the expression of BMP2 in potential target cells remains.

This question is reasonable, as TGF-β1 is highly abundant in solid PRF [18] and is a known agonist of BMP2 expression in mesenchymal cells; for instance, recombinant TGF-β1 has been reported to increase BMP2 expression in bone marrow mesenchymal cells [19]. These findings indicate that TGF-β1 may modulate the BMP2-dependent autocrine/paracrine activity of the target cells. However, knowing that PRF contains TGF-β1 does not mean that PRF is synonymous with TGF-β activity. PRF is more complex than a single growth factor, and whether TGF-β activity accounts for the overall effects of PRF, and to what extent, should be based on experimental evidence. One strategy that allows the determination of TGF-β activity of PRF is based on blocking TGF-β receptor 1 kinase with SB431542 in a bioassay using mesenchymal cells [18,20,21].

In this study, we first performed a proteomics analysis to confirm that PRF membranes are devoid of BMP2; therefore, all BMP2 measured during the assay would be produced by the gingival fibroblasts. Second, we exposed gingival fibroblasts to PRF membrane lysates in the presence and absence of SB431542 to identify the expression of BMP2 that is controlled by activation of TGF-β receptor 1 kinase. Finally, we show that PRF can activate the Smad1/5 signaling pathway, which is usually activated by BMPs. Taken together, our data show that PRF releases TGF-β, which can activate the expression of BMP2 and probably also stimulate the Smad1/5 signaling pathway.

## 2. Results 

### 2.1. Proteomics Analysis of Solid PRF Lysates Shows TGF-β1 but Not BMP2

**Figure 1:**
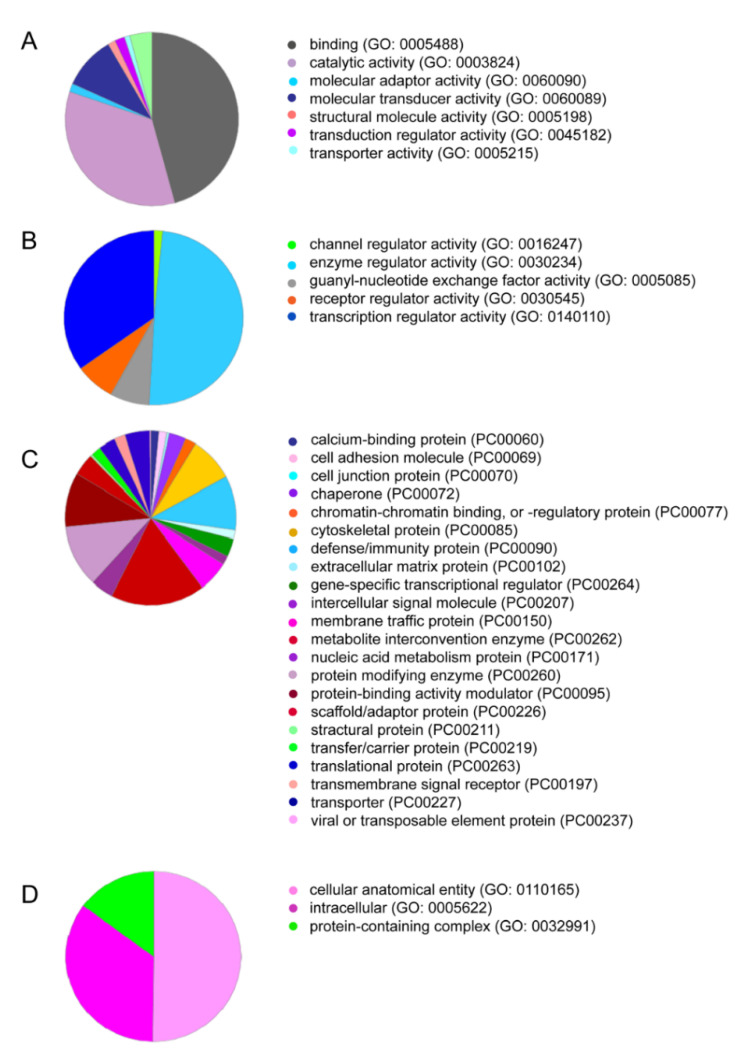
PRF lysates contain a large spectrum of proteins, including TGF-β1. PANTHER analysis for the protein IDs was conducted based on (**A**) molecular function, and among the sections, (**B**) molecular functional regulators including TGF-β1 are indicated. Classifications based on (**C**) protein class and (**D**) cellular components are also presented.

We recently performed a proteomic analysis of PRF lysates showing the presence of the classical growth factor TGF-β1, but neither of the BMP family members was identified [18]. We next examined our data from another independent proteomic analysis of the total PRF clots based on mass spectrometric analysis. This analysis confirmed the presence of TGF-β1 and the lack of BMP2 and other members of the BMP superfamily in the PRF lysates (Supplementary Table S1). The PANTHER classification system revealed 1791 GO terms (Supplementary Table S2) and 1269 functional hits (Figure 1A). The molecular functional regulator (GO:0098772) contained 126 genes with 126 functional hits (Figure 1B). For example, receptor regulator activity (GO: 0030545) contains nine members: transforming growth factor-β1 proprotein, platelet basic protein, follitropin subunit β, platelet factor 4 variant, ectodysplasin A, choriogonadotropin subunit β3, platelet factor 4, C-C motif chemokine S, and choriogonadotropin subunit beta variant 1. Subsequently, REVIGO analysis reduced the visualized gene ontology to 350 terms (Supplementary Table S3). Among the 1791 proteins identified, only a were few growth factors, e.g., TGF-β1, insulin-like growth factor II, myeloid-derived growth factor, epidermal growth factor, and hepatocyte growth factor-like protein, which were enriched in the intercellular signal molecule class (PC00102, Figure 1C). Taken together, the proteomic signature of PRF membranes confirmed the absence of BMPs. However, there is reason to assume, based on in vitro studies with recombinant TGF-β1 [19] and the strong TGF-β activity found in PRF lysates [18,20,21], that these growth factors are capable of inducing the expression of BMP2.

### 2.2. Solid PRF Lysates Stimulate the Expression of BMP2 in Gingival Fibroblasts

**Figure 2:**
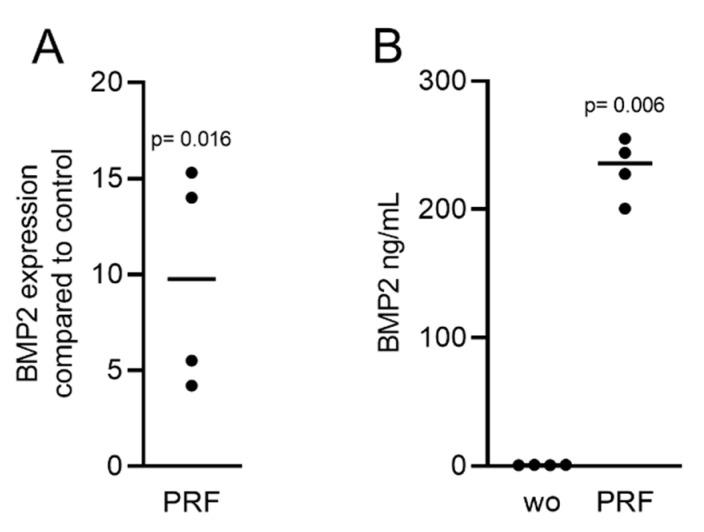
RT-PCR and immunoassays triggered by solid PRF lysates confirmed the presence of BMP2. Gingival fibroblasts were stimulated with 30% soluble PRF lysates. (**A**) Reverse transcription PCR analysis for BMP2 is indicated; (**B**) The levels of BMP2 in the supernatant of the fibroblasts are presented as ng/mL. *n* = 4. Statistical analysis was performed with a two-tailed *t*-test. *p* values are reported comparing the PRF group with the untreated control. Here, and throughout this study, significance was set at *p* < 0.05. ”wo” indicates without and represents unstimulated cells.

**Figure 3:**
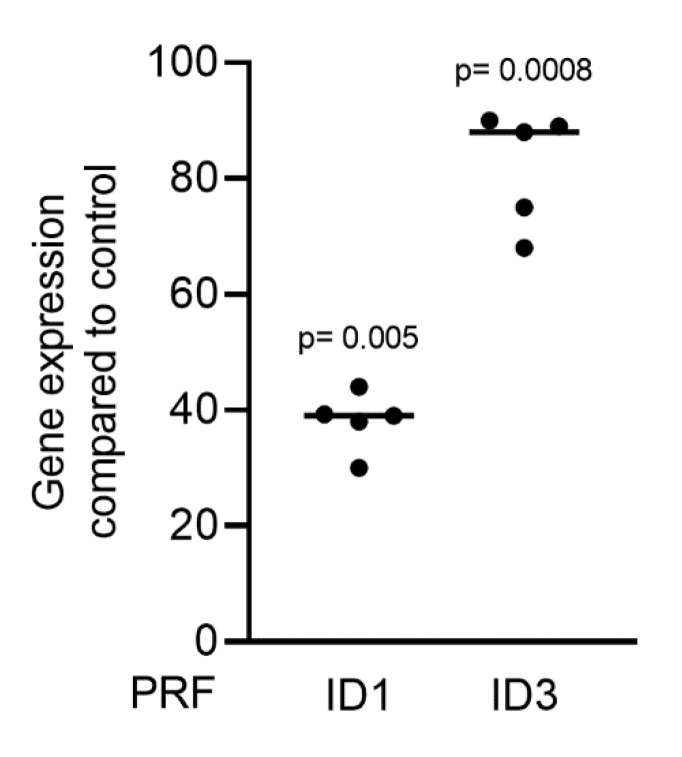
PRF lysates activate the TGF-β and BMP2 target genes ID1 and ID3. Gingival fibroblasts were treated with 30% soluble PRF lysates. PCR analysis of ID1 and ID3 is indicated. *n* = 5. Statistical analysis was performed with a Kruskal–Wallis test for multiple comparisons. *p* values are reported comparing the PRF group with the untreated control.

**Figure 4:**
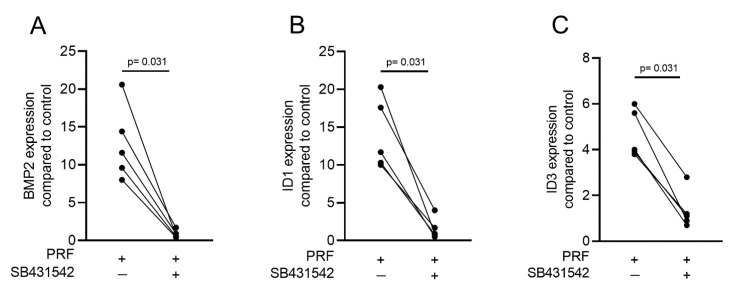
A TGF-β receptor I kinase antagonist blocks BMP2 and its corresponding genes. Gingival fibroblasts were stimulated with PRF lysates in the absence or presence of the TGF-β receptor I kinase antagonist SB431542 (10 µM). PCR analysis for (**A**) BMP2, (**B**) ID1, and (**C**) ID3 gene expression is presented. *n* = 5. Statistical analysis was performed with a two-tailed *t*-test.

Gingival fibroblasts were exposed to PRF lysates, focusing on the expression changes of BMP2. RT-PCR analysis showed that 30% of the PRF lysates increased the expression of BMP2 by up to 17-fold (Figure 2A). TGF-β1 at 30 ng/mL was used as the positive control. Immunoassays confirmed that BMP2 expression was translated into BMP2 at the nanogram per milliliter level (Figure 2B), but BMP2 signals were not observed when the PRF lysates were analyzed (data not shown). RT-PCR analysis further confirmed the increased expression of the typical TGF-β/BMP-regulated ID1 and ID3 genes by PRF lysates (Figure 3). SB431542 significantly suppressed the expression of the BMP2, ID1, and ID3 genes in gingival fibroblasts. These data suggest that PRF lysates can activate TGF-β receptor 1 kinase signaling and thereby increase the expression of BMP2 and the related genes ID1 and ID3 (Figure 4A–C).

### 2.3. PRF Can Activate Smad1/5 Signaling

**Figure 5:**
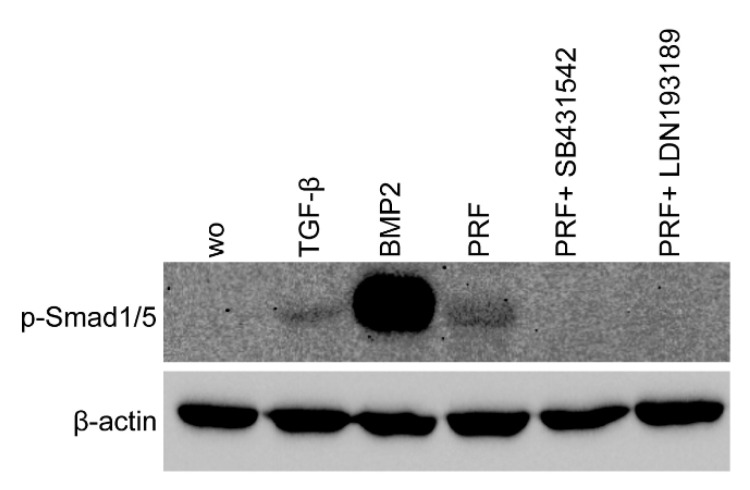
PRF lysates activated the phosphorylation of Smad1/5, which was reduced by treatment with a BMP2 inhibitor. Gingival fibroblasts were exposed to 30% PRF lysate or BMP2 overnight in the presence or absence of SB431542 and LDN193189. Western blot analysis showed a clear increase in the basal Smad1/5 phosphorylation signal in PRF lysates. However, in combination with SB431542 or LDN193189, the signal diminished. ”wo” indicates without and represents unstimulated cells.

**Figure 6:**
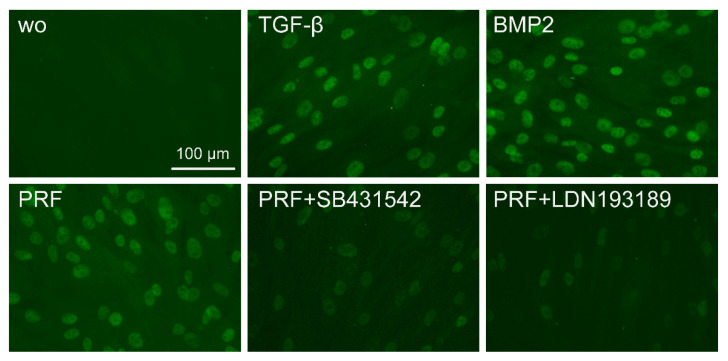
The PRF lysate attenuated the translocation of Smad1/5 from the cytoplasm into the nucleus. Gingival fibroblasts were exposed to TGF-β and BMP2 as positive controls and PRF in the presence or absence of SB431542 and LDN193189. Immunofluorescence analysis of the intracellular translocation of Smad1/5 into the nucleus is presented. ”wo” indicates without and represents unstimulated cells.

**Figure 7:**
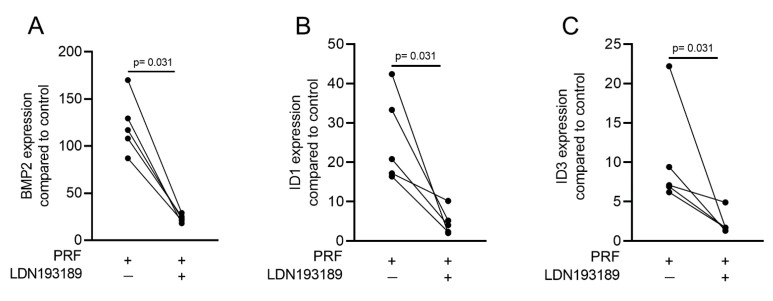
**A** potent ALK2/3 inhibitor can block BMP2 and ID genes. Gingival fibroblasts were exposed to PRF lysates in the presence or absence of 100 nM LDN193189. PCR analyses for (**A**) BMP2, (**B**) ID1, and (**C**) ID3 gene expression are presented. *n* = 5. Statistical analysis was performed with the two-tailed *t*-test.

Considering that TGF-β stimulates Smad1 phosphorylation in a variety of cell lines [20,21] and that TGF-β receptor 1 kinase mediates TGFβ-induced Smad1/5 and Smad2/3 phosphorylation in chondrocytes [22], we wondered whether PRF lysates can activate the BMP-related Smad1/5 signaling pathway apart from the known potent activation of Smad3 canonical TGF-β signaling [18,23]. Support for this claim came from our observations that PRF lysates caused weak phosphorylation of Smad1/5 in serum-starved cells while SB431542 and LDN193189 blocked the phosphorylation (Figure 5). PRF also initiated robust translocation of Smad1/5 into the nucleus in gingival fibroblasts (Figure 6). These data suggest that PRF lysates have at least a modest effect on the activation of canonical BMP-related Smad1/5 signaling. In support of this assumption, LDN193189, an inhibitor of BMP type I receptor signaling (ALK2/3), significantly suppressed the expression of the BMP2, ID1, and ID3 genes after treatment with PRF in gingival fibroblasts (Figure 7A–C), Thus, PRF lysates and TGF-β1, independent of the presence of BMPs, have BMP-like activity.

### 2.4. Liquid PRF Lysates Stimulate the Expression of BMP2 in Gingival Fibroblasts

**Figure 8:**
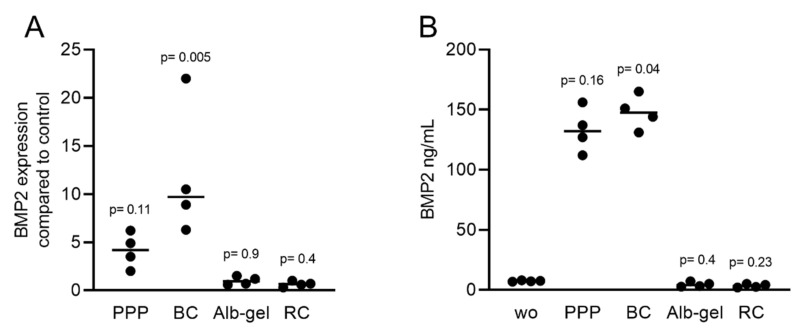
RT-PCR and immunoassays triggered by liquid PRF confirmed the expression of BMP2. Gingival fibroblasts were exposed to PPP, BC, Alb-gel, and RC fractions. (**A**) PCR analysis for BMP2 is indicated; (**B**) The levels of BMP2 in the supernatant of fibroblasts in the presence of PPP and BC are presented as ng/mL. *n* = 4. Statistical analysis was based on the Kruskal–Wallis test for multiple comparisons. ”wo” indicates without and represents unstimulated cells.

**Figure 9:**
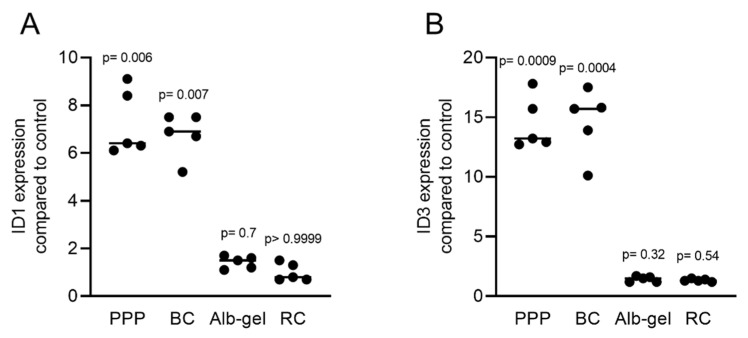
PRF lysates activate the expression of BMP2 and related genes ID1 and ID3. Gingival fibroblasts were treated with PPP, BC, Alb-gel, or RC, and TGF-β was used as the positive control. PCR analyses for (**A**) ID1 and (**B**) ID3 are presented. *n* = 5. Statistical analysis was based on the Kruskal–Wallis test for multiple comparisons.

Considering that the clinical applications of PRF include liquid PRF [24,25], and similar to our previous approach to identify the role of TGF-β activity in these various fractions [23], we determined the effects of lysates from platelet-poor plasma (PPP), buffy coat (BC), heated PPP (Alb-gel), and the remaining red clot on the expression of BMP2 in gingival fibroblasts. Consistent with the recent observation that PPP and BC, but not Alb-gel and red clot lysates, have TGF-β activity [23], we now show that this pattern is also valid for BMP2. Lysates of PPP and BC caused an increase in BMP2 expression in gingival fibroblasts based on the RT-PCR and immunoassay results (Figure 8A,B). As expected, further confirmation of the typical regulation of the ID1 and ID3 genes by TGF-β/BMP after treatment with liquid PRF lysates indicated upregulation of these genes (Figure 9A,B). Thus, not only lysates from solid PRF but also the respective liquid fractions drive the expression of BMP2 in gingival fibroblast cells of the mesenchymal lineage.

## 3. Discussion

This study was based on our recent observations that PRF is composed of a large spectrum of proteins containing TGF-β but not BMPs among the growth factors stored in PRF membranes [18]. Recombinant BMP2 has gained increasing attention because of its osteoinductive activity that drives bone formation in clinical scenarios of bone augmentation in dentistry [11] and enhanced bone remodeling capacity as an autogenous bone graft [26]. PRF has been proposed to serve as a carrier for recombinant BMP2 to achieve controlled release during fibrinolysis [8]. Considering that PRF contains TGF-β [18,20,21] and recombinant TGF-β1 can stimulate BMP2 expression in vitro [19], it is reasonable to suggest that PRF can support the expression of BMP2 in cells of the mesenchymal lineage. In support of this hypothesis, we show here that lysates obtained from solid and liquid PRF are both capable of causing a robust SB431542-dependent increase in BMP2 expression in gingival fibroblasts. Unexpectedly, the PRF lysates and recombinant TGF-β1 caused SB431542-dependent activation of Smad1/5 signaling. Thus, PRF-derived TGF-β not only causes the expression of BMP2 and ID genes but also activates Smad1/5 signaling that is apparently not caused by PRF-derived BMP2.

If we relate these findings to those of others, we learn that the question of whether PRF is a source of BMPs cannot ultimately be answered and this situation remains controversial. Our recent and current proteomic analysis of PRF [18] and data from the immunoassays are consistent with those of others who could not identify BMPs in PRF [27]. In contrast, the immunoassays gave signals for BMP2 [16,17], and proteomic analysis identified the release of BMP4, BMP5, BMP7, and GDF15 from solid PRF [28]. BMPs have also been identified by antibodies in lysates of human platelets [29] but not confirmed by proteomics, as reported in Platelet Web [30]. Nevertheless, we have accumulated evidence that PRF lysates activate Smad1/5 signaling, which is typically caused by BMPs [31], similar to the expression of the genes ID1 and ID3, both potential targets of BMP signaling [32]. In the present study, activation of Smad1/5 signaling is probably a consequence of PRF-derived TGF-β as it was blocked by SB431542 and recombinant TGF-β1 was activating Smad1/5 signaling. Further studies can be designed to recognize the effects of PRF on the activity of BMP2 in BMP-responsive reporter cell lines [33]. 

The clinical relevance of this study is hard to interpret even though PRF membranes are widely used in tissue regeneration, including in the dental field [34,35,36], but can be extended to studies showing the amazing capacity of PRF membranes to treat diabetic ulcers [37] and alveolar ridge preservation [38]. What remains unclear is which key components of PRF membranes appear in the proteomic signature, causing at least some of the beneficial effects of the treatment in clinical scenarios. Considering what we know about the role of TGF-β during the course of natural wound healing [39] and bone regeneration [40] and the local release of therapeutic doses of TGF-β at a defect site, the content of TGF-β in PRF membranes is a possible candidate to predict the clinical activity of a PRF preparation. Moreover, GO analysis tells us that the proteins associated with molecular transducer activity contain TGF-β1, but whether the changes in expression levels of the fibroblasts, including the growth factor BMP2, have an impact on the clinical outcome of PRF treatment remains unclear.

Our study simulates only the scenario where PRF membranes come in contact with local mesenchymal cells and experience a strong TGF-β receptor 1 kinase-dependent change in the gene expression signature. There are many more potential targets for PRF that need to be evaluated in this respect, particularly in cells related to innate immunity and the formation of blood vessels. Nevertheless, fibroblasts are known targets of TGF-β [41], and as they are part of the early stage of wound healing and bone regeneration [42,43], this research at least partially simulates a natural defect situation. To come closer to an answer, preclinical studies where the supposed beneficial effects of PRF are suppressed by SB431542 could be recommended. Unexpected were our findings with LDN193189. LDN193189 inhibits the BMP type I receptors ALK2 and ALK3, with a 200-fold selectivity for BMP versus TGF-β and was originally used by us to interrupt BMP2 autocrine activity. Our data, however, are more in favor of LDN193189 blocking TGF-β signaling. However, LDN212854 did not prevent the phosphorylation of Smad1/5 by TGF-β [21,44]. Moreover, TGFβ-induced Smad1 phosphorylation is independent of BMP type I receptors in certain cell lines [44]. Thus, more research is required to reveal how PRF provoke Smad1/5 phosphorylation and nuclear translation in gingival fibroblasts and thereby activate BMP signaling, a process that can even be enhanced by the expression of BMP2 in an autocrine mode of action.

## 4. Materials and Methods

### 4.1. Cell Culture

Approval for the collection of human gingiva was obtained from the Ethics Committee of the Medical University of Vienna (EK NR 631/2007), and patients signed informed consent forms. Three different strains of fibroblasts were prepared from explant cultures. Gingival fibroblasts were expanded in growth medium containing penicillin, streptomycin (Sigma Aldrich, St. Louis, MO, USA), and 10% fetal bovine serum (Bio&Sell GmbH, Nuremberg, Germany). Fibroblasts (30,000 cells/cm^2^) were exposed to the lysates of unheated and heated PPP (Alb-gel), buffy coat C-PRF (BC), and the red blood clot (RC) in serum-free medium for 24 h at 37 °C with 5% CO_2_ and 95% humidity. The BMP2 receptor was blocked with 10 µM SB431542 (Billerica, MA, USA) and 100 nM LDN193189, a potent and selective ALK2 and ALK3 inhibitor (Cayman, Hamburg, Germany). BMP2 at 300 ng/mL and TGF-β1 at 30 ng/mL (both from ProSpec-Tany TechnoGene Ltd., Rehovot, Israel) were used as the positive controls in the respective experiments.

### 4.2. Preparation of PPP, Alb-gel, Buffy Coat, and Red Clot

Volunteers signed informed consent forms, and the ethics committee of the Medical University of Vienna (1644/2018) approved the preparation of PRF. To prepare PRF gels [45], venous blood was collected from healthy volunteers, three females and three males aged 23 to 35 years old, in plastic tubes (“No Additive”, Greiner Bio-One GmbH, Kremsmünster, Austria) and centrifuged at 700× *g* for 8 min (swing-out rotor; Z 306 Hermle, Universal Centrifuge, Wehingen, Germany). The uppermost 2 mL of PPP and 1 mL of buffy coat, as well as a 1 mL erythrocyte fraction, were collected. To generate PPP gels [45], PPP was immediately heated at 75 °C for 10 min (Eppendorf, Thermomixer F1.5, Hamburg, Germany) before being placed on ice [46]. Each blood fraction was subjected to two freeze–thaw cycles followed by sonication (Sonopuls 2000.2, Bandelin electronic, Berlin, Germany). After centrifugation (Eppendorf, Hamburg, Germany) at 15,000× *g* for 10 min, 1 mL of the lysate was mixed with 0.5 mL of serum-free medium and stored at −20 °C for no longer than one month. Once the cells were ready for stimulation, the fractions were thawed, and cells were exposed as indicated above.

### 4.3. Proteomic Analysis

The detailed protocol is presented in the Supplementary Materials and was recently reported [47]. PRF lysates from a pool of three independent donors were subjected to proteomic analysis [48]. In brief, PRF lysates were first dissolved in 1% Rapigest in 50 mM TEAB, and the solution was filtered through a molecular-weight cutoff filter of 100 kDa. The resulting filtrate was then passed through a 50 kDa filter, and four fractions were generated—two filtrates and two concentrates. Extracted proteins were reconstituted from the membrane and from the filtrate, precipitated using methanol/dichloromethane, and digested with trypsin as described earlier [49]. In total, four fractions were generated and analyzed. Protein concentrations were determined using a DeNovix DS-11 FX Spectrophotometer (Wilmington, Waltham, MA USA), and proteins were reduced using 5 mM dithiothreitol (DTT; Sigma-Aldrich, Vienna, Austria) for 30 min at 60 °C and alkylated for 30 min with 15 mM iodoacetamide (IAA; Sigma-Aldrich, Vienna, Austria) in the dark. Finally, porcine trypsin (Promega, Vienna, Austria) was added at a ratio of 1:50 (*w*/*w*). After 16 h of incubation at 37 °C, aliquots of 20 µL were prepared and stored in 0.5 mL protein low-bind vials (Eppendorf, Vienna, Austria) at −20 °C until injection the next day. Nano-HPLC separation of each fraction was performed using a nanoRSLC UltiMate 3000 HPLC system from Thermo Fisher. Raw MS/MS files were analyzed using Proteome Discoverer 2.2 (Thermo Fisher Scientific, Bremen, Germany) and searching the SwissProt database (Homo sapiens, http://www.UniProt.org/proteomes/UP000009606, version from 10 May 2019). The search parameters are presented in the Supplementary Materials. All search results were refined and researched using Scaffold 4.6.5 (Proteome Software, Portland, OR, USA) [50]. The mass spectrometry proteomics data have been deposited to the ProteomeXchange Consortium (www.proteomexchange.org, accessed on 12 May 2019) via the PRIDE partner repository (www.ebi.ac.uk/pride, accessed on 12 May 2021) with the dataset identifiers PXD014382 and 10.6019/PXD014382. REVIGO analysis was used to reduce the redundancy of the GO terms (http://revigo.irb.hr/, accessed on 12 May 2021).

### 4.4. Reverse Transcription Quantitative Real-Time PCR (RT-qPCR) and Immunoassay

For RT-qPCR [51], total RNA was prepared with the ExtractMe total RNA kit (Blirt S.A., Gdańsk, Poland) followed by reverse transcription (LabQ, Labconsulting, Vienna, Austria) and polymerase chain reaction (LabQ, Labconsulting, Vienna, Austria) on a CFX Connect Real-Time PCR Detection System (Bio-Rad Laboratories, Hercules, CA, USA). The primer sequences were hGAPDH-F: AAGCCACATCGCTCAGACAC and hGAPDH-R: GCCCAATACGACCAAATCC; hBMP2-F: CAGACCACCGGTTGGAGA and hBMP2-R: CCACTCGTTTCTGGTAGTTCTTC; hID1-F: CCAGAACCGCAAGGTGAG and hID1-R: GGTCCCTGATGTAGTCGATGA; hID3-F: CATCTCCAACGACAAAAGGAG and hID3-R: CTTCCGGCAGGAGAGGTT. Amplification was performed with a CFX Connect Real-Time PCR Detection System. The expression levels were calculated after normalizing to the housekeeping gene GAPDH using the ΔΔ*C*_t_ method. The immunoassay for human BMP2 (DY218, R&D Systems, Minneapolis, MN, USA) was performed with the supernatant of gingival fibroblasts exposed to lysates of either PRF or PPP, heated PPP (Alb-gel), buffy coat (BC), or red clot (RC) after 24 h.

### 4.5. Immunostaining

Immunofluorescence analysis of p-Smad1/5 was performed on gingival fibroblasts seeded onto glass slides (Merck, Darmstadt, Germany) that were serum-starved overnight. Thereafter, the cells were treated with TGF-β, BMP2, or PRF for one hour followed by stimulation with the inhibitors SB431542 and LDN193189 for another 30 min in the respective wells. Cells were fixed in paraformaldehyde and blocked with 1% BSA and 0.3% Triton X-100 in PBS at room temperature for one hour. Cells were subsequently incubated with a phospho-Smad1/5 (Ser463/465) antibody (CS-9516, anti-rabbit IgG, 1:1000, Cell Signaling Technology) for another hour. An Alexa Fluor 488 secondary antibody (Cell Signaling Technology, Danvers, MA, USA) was applied for one hour. Cells were washed, and fluorescent images were captured (Euromex, Oxion fluorescence, Arnhem, The Netherlands).

### 4.6. Western Blot

Gingival fibroblasts were seeded at 30,000 cells/cm^2^ into 6-well plates. The following day, the medium was changed to serum-free medium overnight. The wells containing PRF were stimulated with 30% PRF overnight, and the following day, the supernatant was applied to the serum-starved cells for one hour with or without inhibitors SB431542 and LDN193189. Subsequently, all cells were stimulated for one hour. Extracts containing SDS buffer with protease and phosphatase inhibitors (cOmplete ULTRA Tablets and PhosSTOP; Roche, Mannheim, Germany) were separated by SDS-PAGE and transferred onto polyvinylidene fluoride (PVDF) membranes (Roche Diagnostics, Mannheim, Germany). Membranes were blocked, and the binding of the first phospho-Smad1/5 (Ser463/465) antibody (CS-9516, anti-rabbit IgG, 1:1000, Cell Signaling Technology) and actin (SC-47778, anti-mouse IgG, 1:1000, Santa Cruz Biotechnology, Dallas, TX, USA) was detected with the appropriate secondary antibody labeled with HRP (IgG, 1:10,000, Cell Signaling Technology). After exposure to the Clarity Western ECL Substrate (Bio-Rad Laboratories, Inc., Hercules, CA, USA), chemiluminescence signals were visualized with a ChemiDoc imaging system (Bio-Rad Laboratories).

### 4.7. Statistical Analysis

All experiments were performed four to five times. Each data point is representative of one independent experiment. Statistical analysis of BMP2, ID1, and ID3 expression and the BMP2 immunoassay were performed with the Kruskal–Wallis test for multiple comparisons and a two-tailed *t*-test for single comparisons. For multiple comparisons, all groups were compared with the untreated control group. In the PRF groups, each data point is representative of an individual donor. Analyses were performed using Prism v8 (GraphPad Software, La Jolla, CA, USA). Significance was set at *p* < 0.05.

## 5. Conclusions

In summary, we show here that TGF-β is part of the proteomic signature of PRF. Activation of the TGF-β R1 signaling pathway drives the expression of strongly regulated genes in gingival fibroblasts, including the growth factor BMP2 and consequently ID1 and ID3, which can generate a change in the autocrine/paracrine environment of a defect site and significantly affect gingival fibroblasts.

## Supplementary Materials

The following are available online at https://www.mdpi.com/article/10.3390/ijms22157935/s1. Table S1: List of the gene IDs obtained by PANTHER analysis, Table S2: List of the 1791 GO terms derived by PANTHER analysis system based on proteomics analysis, and Table S3: Revigo analysis reduced the visualize gene ontology to 350 GO term that are listed.
