# Supplementary material for: Platelet-Rich Fibrin Increases BMP2 Expression in Oral Fibroblasts via Activation of TGF-β Signaling
Source: Int J Mol Sci. 2021 Jul 25;22(15):7935. doi: 10.3390/ijms22157935 (PMC8347014; doi:10.3390/ijms22157935)
Supplement: Supplementary file 1 [file ijms-22-07935-s001.zip › Supplementary Table 1.pdf]

Table S1: List of the gene IDs obtained by PANTHER analysis.

Gene ID	Gene name	Protein class
HUMAN|HGNC=17955|UniProtKB=O43504	Ragulator complex protein LAMTOR5;LAMTOR5;ortholog	
HUMAN|HGNC=708|UniProtKB=O15511	Actin-related protein 2/3 complex subunit 5;ARPC5;ortholog	actin or actin-binding cytoskeletal protein(PC00041)
HUMAN|HGNC=316|UniProtKB=P43652	Afamin;AFM;ortholog	transfer/carrier protein(PC00219)
HUMAN|HGNC=10046|UniProtKB=P12724	Eosinophil cationic protein;RNASE3;ortholog	
HUMAN|HGNC=15578|UniProtKB=Q96D71	RalBP1-associated Eps domain-containing protein 1;REPS1;ortholog	membrane traffic protein(PC00150)
HUMAN|HGNC=6702|UniProtKB=Q32MZ4	Leucine-rich repeat flightless-interacting protein 1;LRRFIP1;ortholog	DNA-binding transcription factor(PC00218)
HUMAN|HGNC=5527|UniProtKB=P01860	Immunoglobulin heavy constant gamma 3;IGHG3;ortholog	immunoglobulin(PC00123)
HUMAN|HGNC=2315|UniProtKB=Q96FN4	Copine-2;CPNE2;ortholog	
HUMAN|HGNC=2095|UniProtKB=P10909	Clusterin;CLU;ortholog	
HUMAN|HGNC=12822|UniProtKB=Q9NQW7	Xaa-Pro aminopeptidase 1;XPNPEP1;ortholog	protease(PC00190)
HUMAN|HGNC=30391|UniProtKB=Q9UG01	Intraflagellar transport protein 172 homolog;IFT172;ortholog	structural protein(PC00211)
HUMAN|HGNC=5879|UniProtKB=P01699	Immunoglobulin lambda variable 1-44;IGLV1-44;ortholog	immunoglobulin(PC00123)
HUMAN|HGNC=20406|UniProtKB=P48668	Keratin, type II cytoskeletal 6C;KRT6C;ortholog	
HUMAN|HGNC=616|UniProtKB=P02749	Beta-2-glycoprotein 1;APOH;ortholog	
HUMAN|HGNC=30032|UniProtKB=Q6VY07	Phosphofurin acidic cluster sorting protein 1;PACS1;ortholog	
HUMAN|HGNC=9852|UniProtKB=O95373	Importin-7;IPO7;ortholog	transporter(PC00227)
HUMAN|HGNC=7446|UniProtKB=Q9Y4I5	Tesmin;TESMIN;ortholog	
HUMAN|HGNC=15523|UniProtKB=Q9Y2W7	Calsenilin;KCNIP3;ortholog	
HUMAN|HGNC=2873|UniProtKB=P00387	NADH-cytochrome b5 reductase 3;CYB5R3;ortholog	reductase(PC00198)
HUMAN|HGNC=13109|UniProtKB=P17038	Zinc finger protein 43;ZNF43;ortholog	C2H2 zinc finger transcription factor(PC00248)
HUMAN|HGNC=28648|UniProtKB=Q8IYB7	DIS3-like exonuclease 2;DIS3L2;ortholog	exoribonuclease(PC00099)
HUMAN|HGNC=5241|UniProtKB=P11142	Heat shock cognate 71 kDa protein;HSPA8;ortholog	
HUMAN|HGNC=17860|UniProtKB=Q8NC51	Plasminogen activator inhibitor 1 RNA-binding protein;SERBP1;ortholog	RNA metabolism protein(PC00031)
HUMAN|HGNC=5647|UniProtKB=A0A087WSY4	Immunoglobulin heavy variable 4-30-2;IGHV4-30-2;ortholog	immunoglobulin(PC00123)

<table>
<tr><td>HUMAN|HGNC=8587|UniProtKB=P22234</td><td>Multifunctional protein ADE2;PAICS;ortholog</td><td></td></tr>
<tr><td>HUMAN|HGNC=9456|UniProtKB=P07225</td><td>Vitamin K-dependent protein S;PROS1;ortholog</td><td></td></tr>
<tr><td>HUMAN|HGNC=5624|UniProtKB=A0A0B4J1X5</td><td>Immunoglobulin heavy variable 3-74;IGHV3-74;ortholog</td><td>immunoglobulin(PC00123)</td></tr>
<tr><td>HUMAN|HGNC=9536|UniProtKB=O14818</td><td>Proteasome subunit alpha type-7;PSMA7;ortholog</td><td>protease(PC00190)</td></tr>
<tr><td>HUMAN|HGNC=1481|UniProtKB=P04632</td><td>Calpain small subunit 1;CAPNS1;ortholog</td><td></td></tr>
<tr><td>HUMAN|HGNC=9878|UniProtKB=O95267</td><td>RAS guanyl-releasing protein 1;RASGRP1;ortholog</td><td>guanyl-nucleotide exchange factor(PC00113)</td></tr>
<tr><td>HUMAN|HGNC=20331|UniProtKB=Q14644</td><td>Ras GTPase-activating protein 3;RASA3;ortholog</td><td>GTPase-activating protein(PC00257)</td></tr>
<tr><td>HUMAN|HGNC=11124|UniProtKB=P55854</td><td>Small ubiquitin-related modifier 3;SUMO3;ortholog</td><td></td></tr>
<tr><td>HUMAN|HGNC=6413|UniProtKB=P13645</td><td>Keratin, type I cytoskeletal 10;KRT10;ortholog</td><td></td></tr>
<tr><td>HUMAN|HGNC=11027|UniProtKB=P02730</td><td>Band 3 anion transport protein;SLC4A1;ortholog</td><td>secondary carrier transporter(PC00258)</td></tr>
<tr><td>HUMAN|HGNC=4634|UniProtKB=P28161</td><td>Glutathione S-transferase Mu 2;GSTM2;ortholog</td><td>transferase(PC00220)</td></tr>
<tr><td>HUMAN|HGNC=7178|UniProtKB=Q13201</td><td>Multimerin-1;MMRN1;ortholog</td><td></td></tr>
<tr><td>HUMAN|HGNC=1101|UniProtKB=P51587</td><td>Breast cancer type 2 susceptibility protein;BRCA2;ortholog</td><td>damaged DNA-binding protein(PC00086)</td></tr>
<tr><td>HUMAN|HGNC=1540|UniProtKB=P08185</td><td>Corticosteroid-binding globulin;SERPINA6;ortholog</td><td>protease inhibitor(PC00191)</td></tr>
<tr><td>HUMAN|HGNC=28450|UniProtKB=Q8NC44</td><td>Reticulophagy regulator 2;RETREG2;ortholog</td><td></td></tr>
<tr><td>HUMAN|HGNC=4381|UniProtKB=Q14344</td><td>Guanine nucleotide-binding protein subunit alpha-13;GNA13;ortholog</td><td>heterotrimeric G-protein(PC00117)</td></tr>
<tr><td>HUMAN|HGNC=5877|UniProtKB=P01703</td><td>Immunoglobulin lambda variable 1-40;IGLV1-40;ortholog</td><td>immunoglobulin(PC00123)</td></tr>
<tr><td>HUMAN|HGNC=10252|UniProtKB=O75116</td><td>Rho-associated protein kinase 2;ROCK2;ortholog</td><td>non-receptor serine/threonine protein kinase(PC00167)</td></tr>
<tr><td>HUMAN|HGNC=3528|UniProtKB=P00742</td><td>Coagulation factor X;F10;ortholog</td><td>serine protease(PC00203)</td></tr>
<tr><td>HUMAN|HGNC=3338|UniProtKB=Q14247</td><td>Src substrate cortactin;CTTN;ortholog</td><td>non-motor actin binding protein(PC00165)</td></tr>
<tr><td>HUMAN|HGNC=10496|UniProtKB=P06703</td><td>Protein S100-A6;S100A6;ortholog</td><td>calmodulin-related(PC00061)</td></tr>
<tr><td>HUMAN|HGNC=11138|UniProtKB=P37840</td><td>Alpha-synuclein;SNCA;ortholog</td><td>membrane trafficking regulatory protein(PC00151)</td></tr>
<tr><td>HUMAN|HGNC=5213|UniProtKB=P51659</td><td>Peroxisomal multifunctional enzyme type 2;HSD17B4;ortholog</td><td></td></tr>
<tr><td>HUMAN|HGNC=17598|UniProtKB=Q9Y385</td><td>Ubiquitin-conjugating enzyme E2 J1;UBE2J1;ortholog</td><td>ubiquitin-protein ligase(PC00234)</td></tr>
<tr><td>HUMAN|HGNC=2753|UniProtKB=Q16698</td><td>2,4-dienoyl-CoA reductase, mitochondrial;DECR1;ortholog</td><td>oxidoreductase(PC00176)</td></tr>
</table>

HUMAN|HGNC=30765|UniProtKB=Q9UKE5	TRAF2 and NCK-interacting protein kinase;TNIK;ortholog	non-receptor serine/threonine protein kinase(PC00167)
HUMAN|HGNC=8508|UniProtKB=O95747	Serine/threonine-protein kinase OSR1;OXSR1;ortholog	
HUMAN|HGNC=12009|UniProtKB=P60174	Triosephosphate isomerase;TPI1;ortholog	isomerase(PC00135)
HUMAN|HGNC=13937|UniProtKB=O95866	Megakaryocyte and platelet inhibitory receptor G6b;MPIG6B;ortholog	transmembrane signal receptor(PC00197)
HUMAN|HGNC=14637|UniProtKB=Q86UK0	ATP-binding cassette sub-family A member 12;ABCA12;ortholog	ATP-binding cassette (ABC) transporter(PC00003)
HUMAN|HGNC=845|UniProtKB=O75947	ATP synthase subunit d, mitochondrial;ATP5PD;ortholog	ATP synthase(PC00002)
HUMAN|HGNC=3231|UniProtKB=Q9HCU4	Cadherin EGF LAG seven-pass G-type receptor 2;CELSR2;ortholog	cadherin(PC00057)
HUMAN|HGNC=10666|UniProtKB=Q9Y365	START domain-containing protein 10;STARD10;ortholog	
HUMAN|HGNC=10534|UniProtKB=Q9NR31	GTP-binding protein SAR1a;SAR1A;ortholog	
HUMAN|HGNC=8127|UniProtKB=O15294	UDP-N-acetylglucosamine--peptide N-acetylglucosaminyltransferase 110 kDa subunit;OGT;ortholog	protein modifying enzyme(PC00260)
HUMAN|HGNC=12612|UniProtKB=P54578	Ubiquitin carboxyl-terminal hydrolase 14;USP14;ortholog	cysteine protease(PC00081)
HUMAN|HGNC=4243|UniProtKB=P56159	GDNF family receptor alpha-1;GFRA1;ortholog	transmembrane signal receptor(PC00197)
HUMAN|HGNC=31797|UniProtKB=P0C7V6	Putative transcription factor SPT20 homolog-like 2;SUPT20HL2;ortholog	chromatin/chromatin-binding, or -regulatory protein(PC00077)
HUMAN|HGNC=29351|UniProtKB=Q9C0H5	Rho GTPase-activating protein 39;ARHGAP39;ortholog	
HUMAN|HGNC=5645|UniProtKB=A0A0C4DH34	Immunoglobulin heavy variable 4-28;IGHV4-28;ortholog	immunoglobulin(PC00123)
HUMAN|HGNC=15861|UniProtKB=Q9H5Z1	Probable ATP-dependent RNA helicase DHX35;DHX35;ortholog	RNA helicase(PC00032)
HUMAN|HGNC=4620|UniProtKB=P06396	Gelsolin;GSN;ortholog	non-motor actin binding protein(PC00165)
HUMAN|HGNC=2552|UniProtKB=Q13617	Cullin-2;CUL2;ortholog	ubiquitin-protein ligase(PC00234)
HUMAN|HGNC=9086|UniProtKB=P60201	Myelin proteolipid protein;PLP1;ortholog	myelin protein(PC00161)
HUMAN|HGNC=12088|UniProtKB=A0A075B6Z2	T cell receptor alpha joining 56;TRAJ56;ortholog	
HUMAN|HGNC=9065|UniProtKB=P19174	1-phosphatidylinositol 4,5-bisphosphate phosphodiesterase gamma-1;PLCG1;ortholog	phospholipase(PC00186)
HUMAN|HGNC=5157|UniProtKB=P00492	Hypoxanthine-guanine phosphoribosyltransferase;HPRT1;ortholog	glycosyltransferase(PC00111)
HUMAN|HGNC=9164|UniProtKB=Q99719	Septin-5;SEPTIN5;ortholog	cytoskeletal protein(PC00085)
HUMAN|HGNC=29233|UniProtKB=Q7Z434	Mitochondrial antiviral-signaling protein;MAVS;ortholog	
HUMAN|HGNC=6891|UniProtKB=Q15555	Microtubule-associated protein RP/EB family member 2;MAPRE2;ortholog	non-motor microtubule binding protein(PC00166)

HUMAN|HGNC=199|UniProtKB=O43506	Disintegrin and metalloproteinase domain-containing protein 20;ADAM20;ortholog	metalloprotease(PC00153)
HUMAN|HGNC=5636|UniProtKB=A0A075B7B8	IGv domain-containing protein;IGHV3OR16-12;ortholog	immunoglobulin(PC00123)
HUMAN|HGNC=12585|UniProtKB=P31930	Cytochrome b-c1 complex subunit 1, mitochondrial;UQCRC1;ortholog	metalloprotease(PC00153)
HUMAN|HGNC=6835|UniProtKB=P78559	Microtubule-associated protein 1A;MAP1A;ortholog	non-motor microtubule binding protein(PC00166)
HUMAN|HGNC=29162|UniProtKB=Q96BN6	Protein FAM149B1;FAM149B1;ortholog	
HUMAN|HGNC=1617|UniProtKB=P50991	T-complex protein 1 subunit delta;CCT4;ortholog	chaperonin(PC00073)
HUMAN|HGNC=12468|UniProtKB=P0CG48	Polyubiquitin-C;UBC;ortholog	
HUMAN|HGNC=24224|UniProtKB=Q9NYV4	Cyclin-dependent kinase 12;CDK12;ortholog	non-receptor serine/threonine protein kinase(PC00167)
HUMAN|HGNC=6767|UniProtKB=Q15797	Mothers against decapentaplegic homolog 1;SMAD1;ortholog	DNA-binding transcription factor(PC00218)
HUMAN|HGNC=12666|UniProtKB=P55072	Transitional endoplasmic reticulum ATPase;VCP;ortholog	
HUMAN|HGNC=17808|UniProtKB=Q9UPT8	Zinc finger CCCH domain-containing protein 4;ZC3H4;ortholog	zinc finger transcription factor(PC00244)
HUMAN|HGNC=8823|UniProtKB=P16284	Platelet endothelial cell adhesion molecule;PECAM1;ortholog	immunoglobulin receptor superfamily(PC00124)
HUMAN|HGNC=543|UniProtKB=P08758	Annexin A5;ANXA5;ortholog	calcium-binding protein(PC00060)
HUMAN|HGNC=9539|UniProtKB=P49721	Proteasome subunit beta type-2;PSMB2;ortholog	protease(PC00190)
HUMAN|HGNC=25758|UniProtKB=Q8TED0	U3 small nucleolar RNA-associated protein 15 homolog;UTP15;ortholog	RNA processing factor(PC00147)
HUMAN|HGNC=6137|UniProtKB=P17301	Integrin alpha-2;ITGA2;ortholog	integrin(PC00126)
HUMAN|HGNC=30138|UniProtKB=Q8N131	Porimin;TMEM123;ortholog	
HUMAN|HGNC=8927|UniProtKB=Q93100	Phosphorylase b kinase regulatory subunit beta;PHKB;ortholog	kinase activator(PC00138)
HUMAN|HGNC=8783|UniProtKB=Q08499	cAMP-specific 3',5'-cyclic phosphodiesterase 4D;PDE4D;ortholog	phosphodiesterase(PC00185)
HUMAN|HGNC=9571|UniProtKB=Q92530	Proteasome inhibitor PI31 subunit;PSMF1;ortholog	protease inhibitor(PC00191)
HUMAN|HGNC=3218|UniProtKB=Q12805	EGF-containing fibulin-like extracellular matrix protein 1;EFEMP1;ortholog	extracellular matrix structural protein(PC00103)
HUMAN|HGNC=23479|UniProtKB=Q96BY6	Dedicator of cytokinesis protein 10;DOCK10;ortholog	guanyl-nucleotide exchange factor(PC00113)
HUMAN|HGNC=9969|UniProtKB=P35251	Replication factor C subunit 1;RFC1;ortholog	DNA metabolism protein(PC00009)
HUMAN|HGNC=23074|UniProtKB=Q9NX70	Mediator of RNA polymerase II transcription subunit 29;MED29;ortholog	general transcription factor(PC00259)
HUMAN|HGNC=5048|UniProtKB=Q00839	Heterogeneous nuclear ribonucleoprotein U;HNRNPU;ortholog	
HUMAN|HGNC=4922|UniProtKB=P19367	Hexokinase-1;HK1;ortholog	kinase(PC00137)

HUMAN|HGNC=9459|UniProtKB=Q92786	Prospero homeobox protein 1;PROX1;ortholog	homeodomain transcription factor(PC00119)
HUMAN|HGNC=8548|UniProtKB=P07237	Protein disulfide-isomerase;P4HB;ortholog	chaperone(PC00072)
HUMAN|HGNC=545|UniProtKB=P20073	Annexin A7;ANXA7;ortholog	calcium-binding protein(PC00060)
HUMAN|HGNC=6441|UniProtKB=P19013	Keratin, type II cytoskeletal 4;KRT4;ortholog	
HUMAN|HGNC=10467|UniProtKB=Q16799	Reticulon-1;RTN1;ortholog	
HUMAN|HGNC=14674|UniProtKB=Q96QU1	Protocadherin-15;PCDH15;ortholog	cadherin(PC00057)
HUMAN|HGNC=12379|UniProtKB=Q15631	Translin;TSN;ortholog	
HUMAN|HGNC=9756|UniProtKB=O00391	Sulfhydryl oxidase 1;QSOX1;ortholog	oxidase(PC00175)
HUMAN|HGNC=18018|UniProtKB=Q8IX18	Probable ATP-dependent RNA helicase DHX40;DHX40;ortholog	RNA helicase(PC00032)
HUMAN|HGNC=7717|UniProtKB=P19404	NADH dehydrogenase [ubiquinone] flavoprotein 2, mitochondrial;NDUFV2;ortholog	dehydrogenase(PC00092)
HUMAN|HGNC=7618|UniProtKB=O14974	Protein phosphatase 1 regulatory subunit 12A;PPP1R12A;ortholog	phosphatase modulator(PC00184)
HUMAN|HGNC=26614|UniProtKB=Q8NB46	Serine/threonine-protein phosphatase 6 regulatory ankyrin repeat subunit C;ANKRD52;ortholog	scaffold/adaptor protein(PC00226)
HUMAN|HGNC=5834|UniProtKB=P06312	Immunoglobulin kappa variable 4-1;IGKV4-1;ortholog	immunoglobulin(PC00123)
HUMAN|HGNC=15663|UniProtKB=Q9NYU2	UDP-glucose:glycoprotein glucosyltransferase 1;UGGT1;ortholog	glycosyltransferase(PC00111)
HUMAN|HGNC=11476|UniProtKB=O15260	Surfeit locus protein 4;SURF4;ortholog	chaperone(PC00072)
HUMAN|HGNC=32153|UniProtKB=A6NM43	Putative T-complex protein 1 subunit theta-like 1;CCT8L1P;ortholog	chaperonin(PC00073)
HUMAN|HGNC=30456|UniProtKB=Q96DA0	Zymogen granule protein 16 homolog B;ZG16B;ortholog	
HUMAN|HGNC=11572|UniProtKB=P26639	Threonine--tRNA ligase 1, cytoplasmic;TARS1;ortholog	aminoacyl-tRNA synthetase(PC00047)
HUMAN|HGNC=243|UniProtKB=P35611	Alpha-adducin;ADD1;ortholog	non-motor actin binding protein(PC00165)
HUMAN|HGNC=15607|UniProtKB=Q14155	Rho guanine nucleotide exchange factor 7;ARHGEF7;ortholog	guanyl-nucleotide exchange factor(PC00113)
HUMAN|HGNC=823|UniProtKB=P25705	ATP synthase subunit alpha, mitochondrial;ATP5F1A;ortholog	ATP synthase(PC00002)
HUMAN|HGNC=29011|UniProtKB=O15050	TPR and ankyrin repeat-containing protein 1;TRANK1;ortholog	
HUMAN|HGNC=4127|UniProtKB=Q7Z7M9	Polypeptide N-acetylgalactosaminyltransferase 5;GALNT5;ortholog	glycosyltransferase(PC00111)
HUMAN|HGNC=317|UniProtKB=P02771	Alpha-fetoprotein;AFP;ortholog	transfer/carrier protein(PC00219)
HUMAN|HGNC=19296|UniProtKB=Q9UF33	Ephrin type-A receptor 6;EPHA6;ortholog	transmembrane signal receptor(PC00197)
HUMAN|HGNC=2312|UniProtKB=P15169	Carboxypeptidase N catalytic chain;CPN1;ortholog	protease(PC00190)

HUMAN|HGNC=23787|UniProtKB=Q9P1W3	Calcium permeable stress-gated cation channel 1;TMEM63C;ortholog	
HUMAN|HGNC=25694|UniProtKB=Q8TC07	TBC1 domain family member 15;TBC1D15;ortholog	GTPase-activating protein(PC00257)
HUMAN|HGNC=20076|UniProtKB=Q96K76	Ubiquitin carboxyl-terminal hydrolase 47;USP47;ortholog	cysteine protease(PC00081)
HUMAN|HGNC=25631|UniProtKB=Q96MT7	Cilia- and flagella-associated protein 44;CFAP44;ortholog	
HUMAN|HGNC=7228|UniProtKB=P22897	Macrophage mannose receptor 1;MRC1;ortholog	
HUMAN|HGNC=4747|UniProtKB=P58876	Histone H2B type 1-D;H2BC5;ortholog	histone(PC00118)
HUMAN|HGNC=28972|UniProtKB=Q8IXZ2	Zinc finger CCCH domain-containing protein 3;ZC3H3;ortholog	
HUMAN|HGNC=12407|UniProtKB=P68366	Tubulin alpha-4A chain;TUBA4A;ortholog	tubulin(PC00228)
HUMAN|HGNC=11283|UniProtKB=P12931	Proto-oncogene tyrosine-protein kinase Src;SRC;ortholog	
HUMAN|HGNC=2974|UniProtKB=P50570	Dynamin-2;DNM2;ortholog	membrane traffic protein(PC00150)
HUMAN|HGNC=9240|UniProtKB=P02775	Platelet basic protein;PPBP;ortholog	chemokine(PC00074)
HUMAN|HGNC=13420|UniProtKB=Q86TP1	Exopolyphosphatase PRUNE1;PRUNE1;ortholog	
HUMAN|HGNC=1630|UniProtKB=P48509	CD151 antigen;CD151;ortholog	
HUMAN|HGNC=7670|UniProtKB=Q9Y6Q9	Nuclear receptor coactivator 3;NCOA3;ortholog	histone modifying enzyme(PC00261)
HUMAN|HGNC=13406|UniProtKB=Q9Y6V0	Protein piccolo;PCLO;ortholog	
HUMAN|HGNC=535|UniProtKB=P50995	Annexin A11;ANXA11;ortholog	calcium-binding protein(PC00060)
HUMAN|HGNC=21013|UniProtKB=Q6UX71	Plexin domain-containing protein 2;PLXDC2;ortholog	
HUMAN|HGNC=14261|UniProtKB=Q9NS28	Regulator of G-protein signaling 18;RGS18;ortholog	GTPase-activating protein(PC00257)
HUMAN|HGNC=2905|UniProtKB=O14490	Disks large-associated protein 1;DLGAP1;ortholog	scaffold/adaptor protein(PC00226)
HUMAN|HGNC=9141|UniProtKB=Q15126	Phosphomevalonate kinase;PMVK;ortholog	kinase(PC00137)
HUMAN|HGNC=4443|UniProtKB=P40197	Platelet glycoprotein V;GP5;ortholog	
HUMAN|HGNC=115|UniProtKB=P53396	ATP-citrate synthase;ACLY;ortholog	transferase(PC00220)
HUMAN|HGNC=18311|UniProtKB=Q9BS26	Endoplasmic reticulum resident protein 44;ERP44;ortholog	
HUMAN|HGNC=1353|UniProtKB=P07358	Complement component C8 beta chain;C8B;ortholog	
HUMAN|HGNC=5|UniProtKB=P04217	Alpha-1B-glycoprotein;A1BG;ortholog	immunoglobulin receptor superfamily(PC00124)
HUMAN|HGNC=11075|UniProtKB=O14745	Na(+)/H(+) exchange regulatory cofactor NHE-RF1;SLC9A3R1;ortholog	
HUMAN|HGNC=167|UniProtKB=P61163	Alpha-centractin;ACTR1A;ortholog	actin and actin related protein(PC00039)

HUMAN|HGNC=13933|UniProtKB=Q5SQ64	Lymphocyte antigen 6 complex locus protein G6f;LY6G6F;ortholog	
HUMAN|HGNC=4459|UniProtKB=P80108	Phosphatidylinositol-glycan-specific phospholipase D;GPLD1;ortholog	phospholipase(PC00186)
HUMAN|HGNC=17213|UniProtKB=Q9BWP8	Collectin-11;COLEC11;ortholog	surfactant(PC00212)
HUMAN|HGNC=14685|UniProtKB=Q9Y624	Junctional adhesion molecule A;F11R;ortholog	
HUMAN|HGNC=1491|UniProtKB=P47756	F-actin-capping protein subunit beta;CAPZB;ortholog	non-motor actin binding protein(PC00165)
HUMAN|HGNC=3350|UniProtKB=P06733	Alpha-enolase;ENO1;ortholog	lyase(PC00144)
HUMAN|HGNC=9784|UniProtKB=P61020	Ras-related protein Rab-5B;RAB5B;ortholog	
HUMAN|HGNC=5246|UniProtKB=P04792	Heat shock protein beta-1;HSPB1;ortholog	
HUMAN|HGNC=19286|UniProtKB=Q6P3W7	SCY1-like protein 2;SCYL2;ortholog	non-receptor serine/threonine protein kinase(PC00167)
HUMAN|HGNC=3602|UniProtKB=Q9UBX5	Fibulin-5;FBLN5;ortholog	extracellular matrix structural protein(PC00103)
HUMAN|HGNC=22197|UniProtKB=O43299	AP-5 complex subunit zeta-1;AP5Z1;ortholog	
HUMAN|HGNC=16754|UniProtKB=Q96P50	Arf-GAP with coiled-coil, ANK repeat and PH domain-containing protein 3;ACAP3;ortholog	
HUMAN|HGNC=12754|UniProtKB=O75083	WD repeat-containing protein 1;WDR1;ortholog	non-motor actin binding protein(PC00165)
HUMAN|HGNC=5800|UniProtKB=A0A075B6S2	Immunoglobulin kappa variable 2D-29;IGKV2D-29;ortholog	immunoglobulin(PC00123)
HUMAN|HGNC=15878|UniProtKB=Q9NQ75	Cas scaffolding protein family member 4;CASS4;ortholog	
HUMAN|HGNC=31104|UniProtKB=Q92614	Unconventional myosin-XVIIIa;MYO18A;ortholog	
HUMAN|HGNC=11071|UniProtKB=P19634	Sodium/hydrogen exchanger 1;SLC9A1;ortholog	secondary carrier transporter(PC00258)
HUMAN|HGNC=4162|UniProtKB=P41250	Glycine--tRNA ligase;GARS1;ortholog	aminoacyl-tRNA synthetase(PC00047)
HUMAN|HGNC=13206|UniProtKB=O60296	Trafficking kinesin-binding protein 2;TRAK2;ortholog	membrane traffic protein(PC00150)
HUMAN|HGNC=5461|UniProtKB=P78318	Immunoglobulin-binding protein 1;IGBP1;ortholog	chaperone(PC00072)
HUMAN|HGNC=4831|UniProtKB=P69891	Hemoglobin subunit gamma-1;HBG1;ortholog	
HUMAN|HGNC=20063|UniProtKB=Q86T82	Ubiquitin carboxyl-terminal hydrolase 37;USP37;ortholog	cysteine protease(PC00081)
HUMAN|HGNC=19740|UniProtKB=Q96RV3	Pecanex-like protein 1;PCNX1;ortholog	
HUMAN|HGNC=2067|UniProtKB=O00151	PDZ and LIM domain protein 1;PDLIM1;ortholog	actin or actin-binding cytoskeletal protein(PC00041)
HUMAN|HGNC=28843|UniProtKB=Q9HC44	Vasculin-like protein 1;GPBP1L1;ortholog	

HUMAN|HGNC=7380|UniProtKB=P26927	Hepatocyte growth factor-like protein;MST1;ortholog	serine protease(PC00203)
HUMAN|HGNC=3309|UniProtKB=P08246	Neutrophil elastase;ELANE;ortholog	serine protease(PC00203)
HUMAN|HGNC=24251|UniProtKB=Q9Y3A6	Transmembrane emp24 domain-containing protein 5;TMED5;ortholog	vesicle coat protein(PC00235)
HUMAN|HGNC=669|UniProtKB=P08134	Rho-related GTP-binding protein RhoC;RHOC;ortholog	small GTPase(PC00208)
HUMAN|HGNC=24175|UniProtKB=Q9P035	Very-long-chain (3R)-3-hydroxyacyl-CoA dehydratase 3;HACD3;ortholog	
HUMAN|HGNC=12852|UniProtKB=P61981	14-3-3 protein gamma;YWHAG;ortholog	scaffold/adaptor protein(PC00226)
HUMAN|HGNC=5581|UniProtKB=P01766	Immunoglobulin heavy variable 3-13;IGHV3-13;ortholog	immunoglobulin(PC00123)
HUMAN|HGNC=9449|UniProtKB=P04156	Major prion protein;PRNP;ortholog	calcium-binding protein(PC00060)
HUMAN|HGNC=164|UniProtKB=P35609	Alpha-actinin-2;ACTN2;ortholog	
HUMAN|HGNC=37094|UniProtKB=P0CG39	POTE ankyrin domain family member J;POTEJ;ortholog	membrane traffic protein(PC00150)
HUMAN|HGNC=18325|UniProtKB=Q2WGN9	GRB2-associated-binding protein 4;GAB4;ortholog	scaffold/adaptor protein(PC00226)
HUMAN|HGNC=5857|UniProtKB=P0DOY3	Immunoglobulin lambda constant 3;IGLC3;ortholog	immunoglobulin(PC00123)
HUMAN|HGNC=2696|UniProtKB=Q9UJU6	Drebrin-like protein;DBNL;ortholog	non-motor actin binding protein(PC00165)
HUMAN|HGNC=3662|UniProtKB=P02675	Fibrinogen beta chain;FGB;ortholog	intercellular signal molecule(PC00207)
HUMAN|HGNC=17865|UniProtKB=Q96A26	Protein FAM162A;FAM162A;ortholog	
HUMAN|HGNC=7176|UniProtKB=P14780	Matrix metalloproteinase-9;MMP9;ortholog	metalloprotease(PC00153)
HUMAN|HGNC=11125|UniProtKB=P61956	Small ubiquitin-related modifier 2;SUMO2;ortholog	
HUMAN|HGNC=92|UniProtKB=P49748	Very long-chain specific acyl-CoA dehydrogenase, mitochondrial;ACADVL;ortholog	dehydrogenase(PC00092)
HUMAN|HGNC=9553|UniProtKB=P62333	26S proteasome regulatory subunit 10B;PSMC6;ortholog	protease(PC00190)
HUMAN|HGNC=1908|UniProtKB=Q96RL7	Vacuolar protein sorting-associated protein 13A;VPS13A;ortholog	membrane traffic protein(PC00150)
HUMAN|HGNC=5033|UniProtKB=P22626	Heterogeneous nuclear ribonucleoproteins A2/B1;HNRNPA2B1;ortholog	
HUMAN|HGNC=30178|UniProtKB=Q9Y570	Protein phosphatase methylesterase 1;PPME1;ortholog	protein modifying enzyme(PC00260)
HUMAN|HGNC=5781|UniProtKB=A0A0C4DH68	Immunoglobulin kappa variable 2-24;IGKV2-24;ortholog	immunoglobulin(PC00123)
HUMAN|HGNC=9866|UniProtKB=P13631	Retinoic acid receptor gamma;RARG;ortholog	C4 zinc finger nuclear receptor(PC00169)
HUMAN|HGNC=2689|UniProtKB=P09172	Dopamine beta-hydroxylase;DBH;ortholog	hydroxylase(PC00122)
HUMAN|HGNC=9281|UniProtKB=P62136	Serine/threonine-protein phosphatase PP1-alpha catalytic subunit;PPP1CA;ortholog	protein phosphatase(PC00195)
HUMAN|HGNC=291|UniProtKB=P30566	Adenylosuccinate lyase;ADSL;ortholog	lyase(PC00144)

HUMAN|HGNC=10499|UniProtKB=P06702	Protein S100-A9;S100A9;ortholog	calmodulin-related(PC00061)
HUMAN|HGNC=3149|UniProtKB=Q13011	Delta(3,5)-Delta(2,4)-dienoyl-CoA isomerase, mitochondrial;ECH1;ortholog	hydratase(PC00120)
HUMAN|HGNC=5549|UniProtKB=A0A0C4DH31	Immunoglobulin heavy variable 1-18;IGHV1-18;ortholog	immunoglobulin(PC00123)
HUMAN|HGNC=11343|UniProtKB=P50502	Hsc70-interacting protein;ST13;ortholog	
HUMAN|HGNC=658|UniProtKB=P84085	ADP-ribosylation factor 5;ARF5;ortholog	G-protein(PC00020)
HUMAN|HGNC=7579|UniProtKB=P35579	Myosin-9;MYH9;ortholog	
HUMAN|HGNC=29427|UniProtKB=Q8TF17	SH3 domain and tetratricopeptide repeat-containing protein 2;SH3TC2;ortholog	
HUMAN|HGNC=3465|UniProtKB=P10768	S-formylglutathione hydrolase;ESD;ortholog	serine protease(PC00203)
HUMAN|HGNC=13397|UniProtKB=Q9UHQ9	NADH-cytochrome b5 reductase 1;CYB5R1;ortholog	reductase(PC00198)
HUMAN|HGNC=5716|UniProtKB=P01834	Immunoglobulin kappa constant;IGKC;ortholog	immunoglobulin(PC00123)
HUMAN|HGNC=871|UniProtKB=Q9UII2	ATPase inhibitor, mitochondrial;ATP5IF1;ortholog	ligase(PC00142)
HUMAN|HGNC=19246|UniProtKB=Q9NYB0	Telomeric repeat-binding factor 2-interacting protein 1;TERF2IP;ortholog	DNA metabolism protein(PC00009)
HUMAN|HGNC=9545|UniProtKB=P28062	Proteasome subunit beta type-8;PSMB8;ortholog	protease(PC00190)
HUMAN|HGNC=11030|UniProtKB=Q9Y6R1	Electrogenic sodium bicarbonate cotransporter 1;SLC4A4;ortholog	secondary carrier transporter(PC00258)
HUMAN|HGNC=4932|UniProtKB=P01889	HLA class I histocompatibility antigen, B alpha chain;HLA-B;ortholog	major histocompatibility complex protein(PC00149)
HUMAN|HGNC=21449|UniProtKB=Q86UX2	Inter-alpha-trypsin inhibitor heavy chain H5;ITIH5;ortholog	protease inhibitor(PC00191)
HUMAN|HGNC=9723|UniProtKB=P11216	Glycogen phosphorylase, brain form;PYGB;ortholog	glycosyltransferase(PC00111)
HUMAN|HGNC=29827|UniProtKB=O14950	Myosin regulatory light chain 12B;MYL12B;ortholog	actin or actin-binding cytoskeletal protein(PC00041)
HUMAN|HGNC=584|UniProtKB=P02743	Serum amyloid P-component;APCS;ortholog	
HUMAN|HGNC=29953|UniProtKB=Q8WWZ8	Oncoprotein-induced transcript 3 protein;OIT3;ortholog	transmembrane signal receptor(PC00197)
HUMAN|HGNC=4440|UniProtKB=P13224	Platelet glycoprotein Ib beta chain;GP1BB;ortholog	
HUMAN|HGNC=16854|UniProtKB=Q9Y4G8	Rap guanine nucleotide exchange factor 2;RAPGEF2;ortholog	guanyl-nucleotide exchange factor(PC00113)
HUMAN|HGNC=12494|UniProtKB=Q13404	Ubiquitin-conjugating enzyme E2 variant 1;UBE2V1;ortholog	ubiquitin-protein ligase(PC00234)
HUMAN|HGNC=27424|UniProtKB=Q5T481	RNA-binding protein 20;RBM20;ortholog	
HUMAN|HGNC=30747|UniProtKB=P61201	COP9 signalosome complex subunit 2;COPS2;ortholog	
HUMAN|HGNC=7127|UniProtKB=P40692	DNA mismatch repair protein Mlh1;MLH1;ortholog	DNA metabolism protein(PC00009)

HUMAN|HGNC=10825|UniProtKB=P78314	SH3 domain-binding protein 2;SH3BP2;ortholog	
HUMAN|HGNC=15667|UniProtKB=P58546	Myotrophin;MTPN;ortholog	
HUMAN|HGNC=17073|UniProtKB=Q9UNA1	Rho GTPase-activating protein 26;ARHGAP26;ortholog	GTPase-activating protein(PC00257)
HUMAN|HGNC=12692|UniProtKB=P08670	Vimentin;VIM;ortholog	
HUMAN|HGNC=4861|UniProtKB=Q9NRZ9	Lymphoid-specific helicase;HELLS;ortholog	
HUMAN|HGNC=6396|UniProtKB=O00505	Importin subunit alpha-4;KPNA3;ortholog	transporter(PC00227)
HUMAN|HGNC=26027|UniProtKB=Q9H0A8	COMM domain-containing protein 4;COMMD4;ortholog	
HUMAN|HGNC=13518|UniProtKB=Q9Y696	Chloride intracellular channel protein 4;CLIC4;ortholog	ion channel(PC00133)
HUMAN|HGNC=6922|UniProtKB=P11226	Mannose-binding protein C;MBL2;ortholog	extracellular matrix structural protein(PC00103)
HUMAN|HGNC=13919|UniProtKB=P46379	Large proline-rich protein BAG6;BAG6;ortholog	chaperone(PC00072)
HUMAN|HGNC=6711|UniProtKB=Q06643	Lymphotoxin-beta;LTB;ortholog	
HUMAN|HGNC=399|UniProtKB=P02768	Albumin;ALB;ortholog	transfer/carrier protein(PC00219)
HUMAN|HGNC=15453|UniProtKB=Q9H3P7	Golgi resident protein GCP60;ACBD3;ortholog	
HUMAN|HGNC=17136|UniProtKB=Q8IY81	pre-rRNA 2'-O-ribose RNA methyltransferase FTSJ3;FTSJ3;ortholog	RNA methyltransferase(PC00033)
HUMAN|HGNC=28959|UniProtKB=Q14008	Cytoskeleton-associated protein 5;CKAP5;ortholog	non-motor microtubule binding protein(PC00166)
HUMAN|HGNC=10690|UniProtKB=O95810	Caveolae-associated protein 2;CAVIN2;ortholog	membrane traffic protein(PC00150)
HUMAN|HGNC=13759|UniProtKB=Q7L576	Cytoplasmic FMR1-interacting protein 1;CYFIP1;ortholog	scaffold/adaptor protein(PC00226)
HUMAN|HGNC=12840|UniProtKB=P54577	Tyrosine--tRNA ligase, cytoplasmic;YARS1;ortholog	translational protein(PC00263)
HUMAN|HGNC=20597|UniProtKB=P61960	Ubiquitin-fold modifier 1;UFM1;ortholog	
HUMAN|HGNC=29229|UniProtKB=Q9ULI1	NACHT and WD repeat domain-containing protein 2;NWD2;ortholog	
HUMAN|HGNC=5897|UniProtKB=A0A075B6K4	Immunoglobulin lambda variable 3-10;IGLV3-10;ortholog	immunoglobulin(PC00123)
HUMAN|HGNC=30244|UniProtKB=Q6P1A2	Lysophospholipid acyltransferase 5;LPCAT3;ortholog	acetyltransferase(PC00038)
HUMAN|HGNC=7892|UniProtKB=P00491	Purine nucleoside phosphorylase;PNP;ortholog	nucleotide kinase(PC00172)
HUMAN|HGNC=2478|UniProtKB=Q15828	Cystatin-M;CST6;ortholog	
HUMAN|HGNC=30220|UniProtKB=Q96AA3	Protein RFT1 homolog;RFT1;ortholog	
HUMAN|HGNC=25452|UniProtKB=Q8N4L2	Type 2 phosphatidylinositol 4,5-bisphosphate 4-phosphatase;PIP4P2;ortholog	

HUMAN|HGNC=603|UniProtKB=P04114	Apolipoprotein B-100;APOB;ortholog	apolipoprotein(PC00052)
HUMAN|HGNC=24331|UniProtKB=Q5VYJ5	MAM and LDL-receptor class A domain-containing protein 1;MALRD1;ortholog	
HUMAN|HGNC=24319|UniProtKB=Q8IY22	C-Maf-inducing protein;CMIP;ortholog	
HUMAN|HGNC=846|UniProtKB=P56385	ATP synthase subunit e, mitochondrial;ATP5ME;ortholog	ATP synthase(PC00002)
HUMAN|HGNC=21883|UniProtKB=Q9H3E2	Sorting nexin-25;SNX25;ortholog	scaffold/adaptor protein(PC00226)
HUMAN|HGNC=12363|UniProtKB=P49815	Tuberin;TSC2;ortholog	GTPase-activating protein(PC00257)
HUMAN|HGNC=6111|UniProtKB=Q13576	Ras GTPase-activating-like protein IQGAP2;IQGAP2;ortholog	GTPase-activating protein(PC00257)
HUMAN|HGNC=983|UniProtKB=P06276	Cholinesterase;BCHE;ortholog	esterase(PC00097)
HUMAN|HGNC=166|UniProtKB=O43707	Alpha-actinin-4;ACTN4;ortholog	
HUMAN|HGNC=20175|UniProtKB=Q9Y3E0	Vesicle transport protein GOT1B;GOLT1B;ortholog	lipase(PC00143)
HUMAN|HGNC=11364|UniProtKB=P40763	Signal transducer and activator of transcription 3;STAT3;ortholog	DNA-binding transcription factor(PC00218)
HUMAN|HGNC=9847|UniProtKB=P43487	Ran-specific GTPase-activating protein;RANBP1;ortholog	scaffold/adaptor protein(PC00226)
HUMAN|HGNC=2363|UniProtKB=P46109	Crk-like protein;CRKL;ortholog	scaffold/adaptor protein(PC00226)
HUMAN|HGNC=11771|UniProtKB=Q15582	Transforming growth factor-beta-induced protein ig-h3;TGFBI;ortholog	cell adhesion molecule(PC00069)
HUMAN|HGNC=2952|UniProtKB=Q96JB1	Dynein heavy chain 8, axonemal;DNAH8;ortholog	microtubule binding motor protein(PC00156)
HUMAN|HGNC=25541|UniProtKB=P57088	Transmembrane protein 33;TMEM33;ortholog	
HUMAN|HGNC=30512|UniProtKB=Q5JSH3	WD repeat-containing protein 44;WDR44;ortholog	
HUMAN|HGNC=11092|UniProtKB=P03973	Antileukoproteinase;SLPI;ortholog	protease inhibitor(PC00191)
HUMAN|HGNC=12469|UniProtKB=P22314	Ubiquitin-like modifier-activating enzyme 1;UBA1;ortholog	ubiquitin-protein ligase(PC00234)
HUMAN|HGNC=20292|UniProtKB=Q9Y376	Calcium-binding protein 39;CAB39;ortholog	
HUMAN|HGNC=16015|UniProtKB=Q9H8S9	MOB kinase activator 1A;MOB1A;ortholog	kinase activator(PC00138)
HUMAN|HGNC=5164|UniProtKB=Q9Y251	Heparanase;HPSE;ortholog	glycosidase(PC00110)
HUMAN|HGNC=19072|UniProtKB=Q96J01	THO complex subunit 3;THOC3;ortholog	RNA metabolism protein(PC00031)
HUMAN|HGNC=6522|UniProtKB=P04180	Phosphatidylcholine-sterol acyltransferase;LCAT;ortholog	acyltransferase(PC00042)
HUMAN|HGNC=9308|UniProtKB=Q15257	Serine/threonine-protein phosphatase 2A activator;PTPA;ortholog	phosphatase activator(PC00182)
HUMAN|HGNC=11226|UniProtKB=Q96JI7	Spatacsin;SPG11;ortholog	
HUMAN|HGNC=24588|UniProtKB=Q86XP6	Gastrokine-2;GKN2;ortholog	

HUMAN|HGNC=7421|UniProtKB=P00403	Cytochrome c oxidase subunit 2;MT-CO2;ortholog	oxidoreductase(PC00176)
HUMAN|HGNC=9021|UniProtKB=P14618	Pyruvate kinase PKM;PKM;ortholog	kinase(PC00137)
HUMAN|HGNC=3244|UniProtKB=Q9NZN3	EH domain-containing protein 3;EHD3;ortholog	membrane traffic protein(PC00150)
HUMAN|HGNC=3377|UniProtKB=P11171	Protein 4.1;EPB41;ortholog	
HUMAN|HGNC=11317|UniProtKB=Q04837	Single-stranded DNA-binding protein, mitochondrial;SSBP1;ortholog	DNA metabolism protein(PC00009)
HUMAN|HGNC=12527|UniProtKB=Q16851	UTP--glucose-1-phosphate uridylyltransferase;UGP2;ortholog	
HUMAN|HGNC=23172|UniProtKB=Q8N699	Myc target protein 1;MYCT1;ortholog	
HUMAN|HGNC=586|UniProtKB=P13798	Acylamino-acid-releasing enzyme;APEH;ortholog	serine protease(PC00203)
HUMAN|HGNC=2211|UniProtKB=P12109	Collagen alpha-1(VI) chain;COL6A1;ortholog	extracellular matrix structural protein(PC00103)
HUMAN|HGNC=1352|UniProtKB=P07357	Complement component C8 alpha chain;C8A;ortholog	
HUMAN|HGNC=9304|UniProtKB=P63151	Serine/threonine-protein phosphatase 2A 55 kDa regulatory subunit B alpha isoform;PPP2R2A;ortholog	protein phosphatase(PC00195)
HUMAN|HGNC=6637|UniProtKB=P20700	Lamin-B1;LMNB1;ortholog	
HUMAN|HGNC=7539|UniProtKB=Q9NR99	Matrix-remodeling-associated protein 5;MXRA5;ortholog	
HUMAN|HGNC=5901|UniProtKB=A0A075B6K0	Immunoglobulin lambda variable 3-16;IGLV3-16;ortholog	immunoglobulin(PC00123)
HUMAN|HGNC=10011|UniProtKB=Q15382	GTP-binding protein Rheb;RHEB;ortholog	small GTPase(PC00208)
HUMAN|HGNC=6383|UniProtKB=P01042	Kininogen-1;KNG1;ortholog	protease inhibitor(PC00191)
HUMAN|HGNC=1504|UniProtKB=P42574	Caspase-3;CASP3;ortholog	protease(PC00190)
HUMAN|HGNC=9959|UniProtKB=P51606	N-acylglucosamine 2-epimerase;RENBP;ortholog	epimerase/racemase(PC00096)
HUMAN|HGNC=15567|UniProtKB=Q9UJC5	SH3 domain-binding glutamic acid-rich-like protein 2;SH3BGRL2;ortholog	
HUMAN|HGNC=7413|UniProtKB=Q13126	S-methyl-5'-thioadenosine phosphorylase;MTAP;ortholog	nucleotide kinase(PC00172)
HUMAN|HGNC=8630|UniProtKB=P30086	Phosphatidylethanolamine-binding protein 1;PEBP1;ortholog	protease inhibitor(PC00191)
HUMAN|HGNC=23791|UniProtKB=Q27J81	Inverted formin-2;INF2;ortholog	
HUMAN|HGNC=4439|UniProtKB=P07359	Platelet glycoprotein Ib alpha chain;GP1BA;ortholog	transmembrane signal receptor(PC00197)
HUMAN|HGNC=4638|UniProtKB=P09211	Glutathione S-transferase P;GSTP1;ortholog	transferase(PC00220)
HUMAN|HGNC=3625|UniProtKB=O75636	Ficolin-3;FCN3;ortholog	intercellular signal molecule(PC00207)
HUMAN|HGNC=13667|UniProtKB=Q9BXS5	AP-1 complex subunit mu-1;AP1M1;ortholog	membrane traffic protein(PC00150)

HUMAN|HGNC=21035|UniProtKB=Q8N392	Rho GTPase-activating protein 18;ARHGAP18;ortholog	GTPase-activating protein(PC00257)
HUMAN|HGNC=19829|UniProtKB=Q8N128	Protein FAM177A1;FAM177A1;ortholog	
HUMAN|HGNC=7106|UniProtKB=P54252	Ataxin-3;ATXN3;ortholog	chromatin/chromatin-binding, or -regulatory protein(PC00077)
HUMAN|HGNC=29531|UniProtKB=Q6PKG0	La-related protein 1;LARP1;ortholog	RNA metabolism protein(PC00031)
HUMAN|HGNC=2665|UniProtKB=P08174	Complement decay-accelerating factor;CD55;ortholog	
HUMAN|HGNC=14060|UniProtKB=Q9UBZ9	DNA repair protein REV1;REV1;ortholog	DNA metabolism protein(PC00009)
HUMAN|HGNC=12648|UniProtKB=Q9P0L0	Vesicle-associated membrane protein-associated protein A;VAPA;ortholog	membrane trafficking regulatory protein(PC00151)
HUMAN|HGNC=4312|UniProtKB=P48507	Glutamate--cysteine ligase regulatory subunit;GCLM;ortholog	ligase(PC00142)
HUMAN|HGNC=18621|UniProtKB=Q9Y2V7	Conserved oligomeric Golgi complex subunit 6;COG6;ortholog	
HUMAN|HGNC=4632|UniProtKB=P09488	Glutathione S-transferase Mu 1;GSTM1;ortholog	transferase(PC00220)
HUMAN|HGNC=28817|UniProtKB=Q96AG4	Leucine-rich repeat-containing protein 59;LRRC59;ortholog	scaffold/adaptor protein(PC00226)
HUMAN|HGNC=12736|UniProtKB=O43516	WAS/WASL-interacting protein family member 1;WIPF1;ortholog	actin or actin-binding cytoskeletal protein(PC00041)
HUMAN|HGNC=3531|UniProtKB=P00488	Coagulation factor XIII A chain;F13A1;ortholog	transferase(PC00220)
HUMAN|HGNC=5622|UniProtKB=A0A0B4J1Y9	Immunoglobulin heavy variable 3-72;IGHV3-72;ortholog	immunoglobulin(PC00123)
HUMAN|HGNC=5594|UniProtKB=P0DP03	Immunoglobulin heavy variable 3-30-5;IGHV3-30-5;ortholog	immunoglobulin(PC00123)
HUMAN|HGNC=8876|UniProtKB=P17858	ATP-dependent 6-phosphofructokinase, liver type;PFKL;ortholog	carbohydrate kinase(PC00065)
HUMAN|HGNC=11424|UniProtKB=O43815	Striatin;STRN;ortholog	
HUMAN|HGNC=9295|UniProtKB=Q15435	Protein phosphatase 1 regulatory subunit 7;PPP1R7;ortholog	phosphatase modulator(PC00184)
HUMAN|HGNC=4036|UniProtKB=O15117	FYN-binding protein 1;FYB1;ortholog	scaffold/adaptor protein(PC00226)
HUMAN|HGNC=418|UniProtKB=P09972	Fructose-bisphosphate aldolase C;ALDOC;ortholog	aldolase(PC00044)
HUMAN|HGNC=21605|UniProtKB=Q14151	Scaffold attachment factor B2;SAFB2;ortholog	
HUMAN|HGNC=29890|UniProtKB=Q86UT6	NLR family member X1;NLRX1;ortholog	scaffold/adaptor protein(PC00226)
HUMAN|HGNC=29213|UniProtKB=Q8WUJ3	Cell migration-inducing and hyaluronan-binding protein;CEMIP;ortholog	
HUMAN|HGNC=9386|UniProtKB=Q9UGJ0	5'-AMP-activated protein kinase subunit gamma-2;PRKAG2;ortholog	kinase modulator(PC00140)
HUMAN|HGNC=13238|UniProtKB=Q9HDC9	Adipocyte plasma membrane-associated protein;APMAP;ortholog	

HUMAN|HGNC=706|UniProtKB=O15145	Actin-related protein 2/3 complex subunit 3;ARPC3;ortholog	actin or actin-binding cytoskeletal protein(PC00041)
HUMAN|HGNC=598|UniProtKB=Q06481	Amyloid-like protein 2;APLP2;ortholog	protease inhibitor(PC00191)
HUMAN|HGNC=30547|UniProtKB=Q96K17	Transcription factor BTF3 homolog 4;BTF3L4;ortholog	DNA-binding transcription factor(PC00218)
HUMAN|HGNC=8968|UniProtKB=P01833	Polymeric immunoglobulin receptor;PIGR;ortholog	immunoglobulin receptor superfamily(PC00124)
HUMAN|HGNC=23186|UniProtKB=Q14677	Clathrin interactor 1;CLINT1;ortholog	
HUMAN|HGNC=2092|UniProtKB=Q00610	Clathrin heavy chain 1;CLTC;ortholog	vesicle coat protein(PC00235)
HUMAN|HGNC=10996|UniProtKB=O14975	Very long-chain acyl-CoA synthetase;SLC27A2;ortholog	secondary carrier transporter(PC00258)
HUMAN|HGNC=25012|UniProtKB=Q3ZCW2	Galectin-related protein;LGALSL;ortholog	extracellular matrix protein(PC00102)
HUMAN|HGNC=11820|UniProtKB=P01033	Metalloproteinase inhibitor 1;TIMP1;ortholog	protease inhibitor(PC00191)
HUMAN|HGNC=28973|UniProtKB=Q14165	Malectin;MLEC;ortholog	
HUMAN|HGNC=5468|UniProtKB=P35858	Insulin-like growth factor-binding protein complex acid labile subunit;IGFALS;ortholog	transmembrane signal receptor(PC00197)
HUMAN|HGNC=16369|UniProtKB=Q99497	Parkinson disease protein 7;PARK7;ortholog	
HUMAN|HGNC=12674|UniProtKB=Q9Y277	Voltage-dependent anion-selective channel protein 3;VDAC3;ortholog	voltage-gated ion channel(PC00241)
HUMAN|HGNC=9469|UniProtKB=O14668	Transmembrane gamma-carboxyglutamic acid protein 1;PRRG1;ortholog	serine protease(PC00203)
HUMAN|HGNC=6740|UniProtKB=P61626	Lysozyme C;LYZ;ortholog	
HUMAN|HGNC=24700|UniProtKB=Q5QNW6	Histone H2B type 2-F;H2BC18;ortholog	histone(PC00118)
HUMAN|HGNC=28928|UniProtKB=Q86Y46	Keratin, type II cytoskeletal 73;KRT73;ortholog	
HUMAN|HGNC=6901|UniProtKB=P48740	Mannan-binding lectin serine protease 1;MASP1;ortholog	serine protease(PC00203)
HUMAN|HGNC=6168|UniProtKB=Q06033	Inter-alpha-trypsin inhibitor heavy chain H3;ITIH3;ortholog	protease inhibitor(PC00191)
HUMAN|HGNC=5889|UniProtKB=A0A075B6J9	Immunoglobulin lambda variable 2-18;IGLV2-18;ortholog	immunoglobulin(PC00123)
HUMAN|HGNC=2876|UniProtKB=O60610	Protein diaphanous homolog 1;DIAPH1;ortholog	
HUMAN|HGNC=9568|UniProtKB=Q06323	Proteasome activator complex subunit 1;PSME1;ortholog	
HUMAN|HGNC=5759|UniProtKB=A0A087WSZ0	Immunoglobulin kappa variable 1D-8;IGKV1D-8;ortholog	immunoglobulin(PC00123)
HUMAN|HGNC=7097|UniProtKB=P14174	Macrophage migration inhibitory factor;MIF;ortholog	decarboxylase(PC00089)
HUMAN|HGNC=9259|UniProtKB=P30405	Peptidyl-prolyl cis-trans isomerase F, mitochondrial;PPIF;ortholog	chaperone(PC00072)
HUMAN|HGNC=20451|UniProtKB=Q8IWB7	WD repeat and FYVE domain-containing protein 1;WDFY1;ortholog	

HUMAN|HGNC=14313|UniProtKB=Q8WYA0	Intraflagellar transport protein 81 homolog;IFT81;ortholog	
HUMAN|HGNC=9275|UniProtKB=P35813	Protein phosphatase 1A;PPM1A;ortholog	
HUMAN|Gene=SELO|UniProtKB=Q9BVL4	Protein adenylyltransferase SelO, mitochondrial;SELENOO;ortholog	
HUMAN|HGNC=12855|UniProtKB=P63104	14-3-3 protein zeta/delta;YWHAZ;ortholog	scaffold/adaptor protein(PC00226)
HUMAN|HGNC=6040|UniProtKB=Q13418	Integrin-linked protein kinase;ILK;ortholog	non-receptor serine/threonine protein kinase(PC00167)
HUMAN|HGNC=11085|UniProtKB=O75093	Slit homolog 1 protein;SLIT1;ortholog	
HUMAN|HGNC=9885|UniProtKB=P29374	AT-rich interactive domain-containing protein 4A;ARID4A;ortholog	transcription cofactor(PC00217)
HUMAN|HGNC=3964|UniProtKB=P01225	Follitropin subunit beta;FSHB;ortholog	peptide hormone(PC00179)
HUMAN|HGNC=10542|UniProtKB=O95248	Myotubularin-related protein 5;SBF1;ortholog	phosphatase(PC00181)
HUMAN|HGNC=33704|UniProtKB=Q66K66	Transmembrane protein 198;TMEM198;ortholog	
HUMAN|HGNC=21689|UniProtKB=Q9Y3D6	Mitochondrial fission 1 protein;FIS1;ortholog	
HUMAN|HGNC=9551|UniProtKB=P43686	26S proteasome regulatory subunit 6B;PSMC4;ortholog	protease(PC00190)
HUMAN|HGNC=29513|UniProtKB=O60306	RNA helicase aquarius;AQR;ortholog	
HUMAN|HGNC=25900|UniProtKB=Q9H425	Uncharacterized protein C1orf198;C1orf198;ortholog	
HUMAN|HGNC=369|UniProtKB=Q9UKA4	A-kinase anchor protein 11;AKAP11;ortholog	scaffold/adaptor protein(PC00226)
HUMAN|HGNC=11442|UniProtKB=O15400	Syntaxin-7;STX7;ortholog	SNARE protein(PC00034)
HUMAN|HGNC=5258|UniProtKB=P08238	Heat shock protein HSP 90-beta;HSP90AB1;ortholog	Hsp90 family chaperone(PC00028)
HUMAN|HGNC=4226|UniProtKB=P31150	Rab GDP dissociation inhibitor alpha;GDI1;ortholog	G-protein modulator(PC00022)
HUMAN|HGNC=11005|UniProtKB=P11166	Solute carrier family 2, facilitated glucose transporter member 1;SLC2A1;ortholog	
HUMAN|HGNC=613|UniProtKB=P02649	Apolipoprotein E;APOE;ortholog	apolipoprotein(PC00052)
HUMAN|HGNC=3250|UniProtKB=P47813	Eukaryotic translation initiation factor 1A, X-chromosomal;EIF1AX;ortholog	translation initiation factor(PC00224)
HUMAN|Gene=HV108_HUMAN|UniProtKB=P0DP01	Immunoglobulin heavy variable 1-8;IGHV1-8;ortholog	immunoglobulin(PC00123)
HUMAN|HGNC=17234|UniProtKB=Q9BXX3	Ankyrin repeat domain-containing protein 30A;ANKRD30A;ortholog	
HUMAN|HGNC=10074|UniProtKB=P13489	Ribonuclease inhibitor;RNH1;ortholog	
HUMAN|HGNC=5046|UniProtKB=P52272	Heterogeneous nuclear ribonucleoprotein M;HNRNPM;ortholog	RNA splicing factor(PC00148)
HUMAN|HGNC=12729|UniProtKB=P23381	Tryptophan--tRNA ligase, cytoplasmic;WARS1;ortholog	aminoacyl-tRNA synthetase(PC00047)

HUMAN|HGNC=1243|UniProtKB=Q07021	Complement component 1 Q subcomponent-binding protein, mitochondrial;C1QBP;ortholog	
HUMAN|HGNC=7544|UniProtKB=Q96S97	Myeloid-associated differentiation marker;MYADM;ortholog	
HUMAN|HGNC=7060|UniProtKB=P08493	Matrix Gla protein;MGP;ortholog	
HUMAN|HGNC=13633|UniProtKB=Q15848	Adiponectin;ADIPOQ;ortholog	
HUMAN|HGNC=6703|UniProtKB=Q9Y608	Leucine-rich repeat flightless-interacting protein 2;LRRFIP2;ortholog	DNA-binding transcription factor(PC00218)
HUMAN|HGNC=5598|UniProtKB=A0A0C4DH35	Probable non-functional immunoglobulin heavy variable 3-35;IGHV3-35;ortholog	immunoglobulin(PC00123)
HUMAN|HGNC=11828|UniProtKB=Q9UDY2	Tight junction protein ZO-2;TJP2;ortholog	tight junction(PC00214)
HUMAN|HGNC=19176|UniProtKB=Q96DZ9	CKLF-like MARVEL transmembrane domain-containing protein 5;CMTM5;ortholog	membrane traffic protein(PC00150)
HUMAN|HGNC=108|UniProtKB=P22303	Acetylcholinesterase;ACHE;ortholog	esterase(PC00097)
HUMAN|HGNC=12635|UniProtKB=P46939	Utrophin;UTRN;ortholog	
HUMAN|HGNC=9581|UniProtKB=Q9H939	Proline-serine-threonine phosphatase-interacting protein 2;PSTPIP2;ortholog	actin or actin-binding cytoskeletal protein(PC00041)
HUMAN|HGNC=10381|UniProtKB=P04843	Dolichyl-diphosphooligosaccharide--protein glycosyltransferase subunit 1;RPN1;ortholog	glycosyltransferase(PC00111)
HUMAN|HGNC=5550|UniProtKB=P23083	Immunoglobulin heavy variable 1-2;IGHV1-2;ortholog	immunoglobulin(PC00123)
HUMAN|HGNC=25443|UniProtKB=Q9NSK7	Protein C19orf12;C19orf12;ortholog	
HUMAN|HGNC=25842|UniProtKB=Q6UWX4	HHIP-like protein 2;HHIPL2;ortholog	protein-binding activity modulator(PC00095)
HUMAN|HGNC=5634|UniProtKB=A0A075B7F0	Ig-like domain-containing protein;IGHV3OR16-10;ortholog	immunoglobulin(PC00123)
HUMAN|HGNC=5171|UniProtKB=P02790	Hemopexin;HPX;ortholog	metalloprotease(PC00153)
HUMAN|HGNC=649|UniProtKB=P48444	Coatomer subunit delta;ARCN1;ortholog	vesicle coat protein(PC00235)
HUMAN|HGNC=6541|UniProtKB=P07195	L-lactate dehydrogenase B chain;LDHB;ortholog	dehydrogenase(PC00092)
HUMAN|HGNC=2232|UniProtKB=P35606	Coatomer subunit beta';COPB2;ortholog	vesicle coat protein(PC00235)
HUMAN|HGNC=2188|UniProtKB=Q99715	Collagen alpha-1(XII) chain;COL12A1;ortholog	extracellular matrix structural protein(PC00103)
HUMAN|HGNC=12491|UniProtKB=P61081	NEDD8-conjugating enzyme Ubc12;UBE2M;ortholog	ubiquitin-protein ligase(PC00234)
HUMAN|HGNC=9302|UniProtKB=P30153	Serine/threonine-protein phosphatase 2A 65 kDa regulatory subunit A alpha isoform;PPP2R1A;ortholog	phosphatase modulator(PC00184)
HUMAN|HGNC=2367|UniProtKB=P02741	C-reactive protein;CRP;ortholog	
HUMAN|HGNC=9071|UniProtKB=P00747	Plasminogen;PLG;ortholog	serine protease(PC00203)
HUMAN|HGNC=3482|UniProtKB=P38117	Electron transfer flavoprotein subunit beta;ETFB;ortholog	oxidoreductase(PC00176)

HUMAN|HGNC=30167|UniProtKB=P13667	Protein disulfide-isomerase A4;PDIA4;ortholog	chaperone(PC00072)
HUMAN|HGNC=20778|UniProtKB=P07437	Tubulin beta chain;TUBB;ortholog	tubulin(PC00228)
HUMAN|HGNC=26853|UniProtKB=Q8N7Z5	Ankyrin repeat domain-containing protein 31;ANKRD31;ortholog	
HUMAN|HGNC=2209|UniProtKB=P20908	Collagen alpha-1(V) chain;COL5A1;ortholog	extracellular matrix structural protein(PC00103)
HUMAN|HGNC=21539|UniProtKB=Q9BX46	RNA-binding protein 24;RBM24;ortholog	
HUMAN|HGNC=5575|UniProtKB=A0A0B4J1V2	Immunoglobulin heavy variable 2-26;IGHV2-26;ortholog	immunoglobulin(PC00123)
HUMAN|HGNC=12347|UniProtKB=Q9Y4A5	Transformation/transcription domain-associated protein;TRRAP;ortholog	non-receptor serine/threonine protein kinase(PC00167)
HUMAN|HGNC=5655|UniProtKB=A0A0C4DH41	Immunoglobulin heavy variable 4-61;IGHV4-61;ortholog	immunoglobulin(PC00123)
HUMAN|HGNC=5856|UniProtKB=P0DOY2	Immunoglobulin lambda constant 2;IGLC2;ortholog	immunoglobulin(PC00123)
HUMAN|HGNC=24822|UniProtKB=Q9H479	Fructosamine-3-kinase;FN3K;ortholog	kinase(PC00137)
HUMAN|HGNC=15474|UniProtKB=Q9Y566	SH3 and multiple ankyrin repeat domains protein 1;SHANK1;ortholog	
HUMAN|HGNC=17468|UniProtKB=Q96HC4	PDZ and LIM domain protein 5;PDLIM5;ortholog	actin or actin-binding cytoskeletal protein(PC00041)
HUMAN|HGNC=5898|UniProtKB=A0A075B6K2	Immunoglobulin lambda variable 3-12;IGLV3-12;ortholog	immunoglobulin(PC00123)
HUMAN|HGNC=12601|UniProtKB=O75445	Usherin;USH2A;ortholog	extracellular matrix protein(PC00102)
HUMAN|HGNC=8087|UniProtKB=P29728	2'-5'-oligoadenylate synthase 2;OAS2;ortholog	nucleotidyltransferase(PC00174)
HUMAN|HGNC=169|UniProtKB=P61160	Actin-related protein 2;ACTR2;ortholog	actin and actin related protein(PC00039)
HUMAN|HGNC=20250|UniProtKB=Q96IF1	LIM domain-containing protein ajuba;AJUBA;ortholog	transcription cofactor(PC00217)
HUMAN|HGNC=27937|UniProtKB=Q6ZVH7	Espin-like protein;ESPNL;ortholog	non-motor actin binding protein(PC00165)
HUMAN|HGNC=1093|UniProtKB=P07738	Bisphosphoglycerate mutase;BPGM;ortholog	mutase(PC00160)
HUMAN|HGNC=29884|UniProtKB=Q8TF42	Ubiquitin-associated and SH3 domain-containing protein B;UBASH3B;ortholog	
HUMAN|HGNC=9392|UniProtKB=P31323	cAMP-dependent protein kinase type II-beta regulatory subunit;PRKAR2B;ortholog	kinase modulator(PC00140)
HUMAN|HGNC=4890|UniProtKB=P36980	Complement factor H-related protein 2;CFHR2;ortholog	
HUMAN|HGNC=9782|UniProtKB=P61018	Ras-related protein Rab-4B;RAB4B;ortholog	
HUMAN|HGNC=15487|UniProtKB=Q9H3N1	Thioredoxin-related transmembrane protein 1;TMX1;ortholog	
HUMAN|HGNC=1406|UniProtKB=Q9Y698	Voltage-dependent calcium channel gamma-2 subunit;CACNG2;ortholog	voltage-gated ion channel(PC00241)

HUMAN|HGNC=5253|UniProtKB=P07900	Heat shock protein HSP 90-alpha;HSP90AA1;ortholog	Hsp90 family chaperone(PC00028)
HUMAN|HGNC=30688|UniProtKB=Q86VP6	Cullin-associated NEDD8-dissociated protein 1;CAND1;ortholog	ubiquitin-protein ligase(PC00234)
HUMAN|HGNC=17292|UniProtKB=Q8IWA5	Choline transporter-like protein 2;SLC44A2;ortholog	secondary carrier transporter(PC00258)
HUMAN|HGNC=8738|UniProtKB=Q15113	Procollagen C-endopeptidase enhancer 1;PCOLCE;ortholog	serine protease(PC00203)
HUMAN|HGNC=122|UniProtKB=P24666	Low molecular weight phosphotyrosine protein phosphatase;ACP1;ortholog	protein phosphatase(PC00195)
HUMAN|HGNC=30168|UniProtKB=Q15084	Protein disulfide-isomerase A6;PDIA6;ortholog	chaperone(PC00072)
HUMAN|HGNC=8824|UniProtKB=P36955	Pigment epithelium-derived factor;SERPINF1;ortholog	protease inhibitor(PC00191)
HUMAN|HGNC=3750|UniProtKB=Q13045	Protein flightless-1 homolog;FLII;ortholog	non-motor actin binding protein(PC00165)
HUMAN|HGNC=16257|UniProtKB=Q9H4B7	Tubulin beta-1 chain;TUBB1;ortholog	tubulin(PC00228)
HUMAN|HGNC=7803|UniProtKB=Q12986	Transcriptional repressor NF-X1;NFX1;ortholog	zinc finger transcription factor(PC00244)
HUMAN|HGNC=17103|UniProtKB=Q9Y5L0	Transportin-3;TNPO3;ortholog	transporter(PC00227)
HUMAN|HGNC=19974|UniProtKB=Q8IUC4	Rhophilin-2;RHPN2;ortholog	scaffold/adaptor protein(PC00226)
HUMAN|HGNC=1690|UniProtKB=O43866	CD5 antigen-like;CD5L;ortholog	
HUMAN|HGNC=17970|UniProtKB=Q9BPZ3	Polyadenylate-binding protein-interacting protein 2;PAIP2;ortholog	
HUMAN|HGNC=333|UniProtKB=P01019	Angiotensinogen;AGT;ortholog	protease inhibitor(PC00191)
HUMAN|HGNC=121|UniProtKB=O15254	Peroxisomal acyl-coenzyme A oxidase 3;ACOX3;ortholog	oxidoreductase(PC00176)
HUMAN|HGNC=30550|UniProtKB=O60337	E3 ubiquitin-protein ligase MARCHF6;MARCHF6;ortholog	ubiquitin-protein ligase(PC00234)
HUMAN|HGNC=29480|UniProtKB=P02750	Leucine-rich alpha-2-glycoprotein;LRG1;ortholog	
HUMAN|HGNC=28164|UniProtKB=Q96M27	Protein PRRC1;PRRC1;ortholog	
HUMAN|HGNC=4398|UniProtKB=P62879	Guanine nucleotide-binding protein G(I)/G(S)/G(T) subunit beta-2;GNB2;ortholog	heterotrimeric G-protein(PC00117)
HUMAN|HGNC=13531|UniProtKB=Q9Y2Q0	Phospholipid-transporting ATPase IA;ATP8A1;ortholog	primary active transporter(PC00068)
HUMAN|HGNC=1455|UniProtKB=P27797	Calreticulin;CALR;ortholog	chaperone(PC00072)
HUMAN|HGNC=2898|UniProtKB=P09622	Dihydrolipoyl dehydrogenase, mitochondrial;DLD;ortholog	oxidoreductase(PC00176)
HUMAN|HGNC=18233|UniProtKB=Q8NDB2	B-cell scaffold protein with ankyrin repeats;BANK1;ortholog	
HUMAN|HGNC=20589|UniProtKB=Q9P0J7	E3 ubiquitin-protein ligase KCMF1;KCMF1;ortholog	ubiquitin-protein ligase(PC00234)
HUMAN|HGNC=2239|UniProtKB=Q9UNS2	COP9 signalosome complex subunit 3;COPS3;ortholog	protease(PC00190)
HUMAN|HGNC=23112|UniProtKB=Q8TDW7	Protocadherin Fat 3;FAT3;ortholog	cadherin(PC00057)

HUMAN|HGNC=1704|UniProtKB=Q9UIB8	SLAM family member 5;CD84;ortholog	immunoglobulin receptor superfamily(PC00124)
HUMAN|HGNC=15720|UniProtKB=Q13033	Striatin-3;STRN3;ortholog	
HUMAN|HGNC=9317|UniProtKB=P63098	Calcineurin subunit B type 1;PPP3R1;ortholog	
HUMAN|HGNC=5478|UniProtKB=P01876	Immunoglobulin heavy constant alpha 1;IGHA1;ortholog	immunoglobulin(PC00123)
HUMAN|HGNC=28920|UniProtKB=Q5SW79	Centrosomal protein of 170 kDa;CEP170;ortholog	scaffold/adaptor protein(PC00226)
HUMAN|HGNC=2288|UniProtKB=P14406	Cytochrome c oxidase subunit 7A2, mitochondrial;COX7A2;ortholog	oxidase(PC00175)
HUMAN|HGNC=19162|UniProtKB=Q9P2P6	StAR-related lipid transfer protein 9;STARD9;ortholog	
HUMAN|HGNC=16998|UniProtKB=P49755	Transmembrane emp24 domain-containing protein 10;TMED10;ortholog	vesicle coat protein(PC00235)
HUMAN|HGNC=9857|UniProtKB=P61224	Ras-related protein Rap-1b;RAP1B;ortholog	small GTPase(PC00208)
HUMAN|HGNC=11937|UniProtKB=P32970	CD70 antigen;CD70;ortholog	
HUMAN|HGNC=7863|UniProtKB=Q13423	NAD(P) transhydrogenase, mitochondrial;NNT;ortholog	dehydrogenase(PC00092)
HUMAN|HGNC=29219|UniProtKB=Q6ZU35	Capping protein inhibiting regulator of actin dynamics;CRACD;ortholog	
HUMAN|HGNC=363|UniProtKB=P27144	Adenylate kinase 4, mitochondrial;AK4;ortholog	nucleotide kinase(PC00172)
HUMAN|HGNC=12203|UniProtKB=A0A075B6N3	T cell receptor beta variable 24-1;TRBV24-1;ortholog	
HUMAN|HGNC=4141|UniProtKB=P04406	Glyceraldehyde-3-phosphate dehydrogenase;GAPDH;ortholog	dehydrogenase(PC00092)
HUMAN|HGNC=1373|UniProtKB=P00918	Carbonic anhydrase 2;CA2;ortholog	
HUMAN|HGNC=1553|UniProtKB=Q13185	Chromobox protein homolog 3;CBX3;ortholog	
HUMAN|HGNC=18874|UniProtKB=O43561	Linker for activation of T-cells family member 1;LAT;ortholog	
HUMAN|HGNC=29374|UniProtKB=Q76NI1	Kinase non-catalytic C-lobe domain-containing protein 1;KNDC1;ortholog	guanyl-nucleotide exchange factor(PC00113)
HUMAN|Gene=SEP15|UniProtKB=O60613	Selenoprotein F;SELENOF;ortholog	
HUMAN|HGNC=8862|UniProtKB=P10720	Platelet factor 4 variant;PF4V1;ortholog	chemokine(PC00074)
HUMAN|HGNC=17643|UniProtKB=Q9HD26	Golgi-associated PDZ and coiled-coil motif-containing protein;GOPC;ortholog	membrane traffic protein(PC00150)
HUMAN|HGNC=8647|UniProtKB=Q15365	Poly(rC)-binding protein 1;PCBP1;ortholog	RNA metabolism protein(PC00031)
HUMAN|HGNC=20100|UniProtKB=A0A1B0GTZ2	Putative coiled-coil domain-containing protein 196;CCDC196;ortholog	
HUMAN|HGNC=17089|UniProtKB=Q8NF91	Nesprin-1;SYNE1;ortholog	
HUMAN|HGNC=7326|UniProtKB=P20585	DNA mismatch repair protein Msh3;MSH3;ortholog	DNA metabolism protein(PC00009)
HUMAN|HGNC=26951|UniProtKB=Q86YL5	Testis development-related protein;TDRP;ortholog	

HUMAN|HGNC=2593|UniProtKB=P05093	Steroid 17-alpha-hydroxylase/17,20 lyase;CYP17A1;ortholog	
HUMAN|HGNC=8941|UniProtKB=P01009	Alpha-1-antitrypsin;SERPINA1;ortholog	protease inhibitor(PC00191)
HUMAN|HGNC=1736|UniProtKB=P60953	Cell division control protein 42 homolog;CDC42;ortholog	small GTPase(PC00208)
HUMAN|HGNC=12013|UniProtKB=P67936	Tropomyosin alpha-4 chain;TPM4;ortholog	actin binding motor protein(PC00040)
HUMAN|HGNC=8878|UniProtKB=Q01813	ATP-dependent 6-phosphofructokinase, platelet type;PFKP;ortholog	carbohydrate kinase(PC00065)
HUMAN|HGNC=5237|UniProtKB=P34932	Heat shock 70 kDa protein 4;HSPA4;ortholog	
HUMAN|HGNC=533|UniProtKB=P04083	Annexin A1;ANXA1;ortholog	calcium-binding protein(PC00060)
HUMAN|HGNC=21073|UniProtKB=Q8NBS9	Thioredoxin domain-containing protein 5;TXNDC5;ortholog	chaperone(PC00072)
HUMAN|HGNC=5927|UniProtKB=P01721	Immunoglobulin lambda variable 6-57;IGLV6-57;ortholog	immunoglobulin(PC00123)
HUMAN|HGNC=14247|UniProtKB=O75964	ATP synthase subunit g, mitochondrial;ATP5MG;ortholog	ATP synthase(PC00002)
HUMAN|HGNC=8583|UniProtKB=P05121	Plasminogen activator inhibitor 1;SERPINE1;ortholog	protease inhibitor(PC00191)
HUMAN|HGNC=1117|UniProtKB=Q9UPA5	Protein bassoon;BSN;ortholog	
HUMAN|HGNC=10447|UniProtKB=P10301	Ras-related protein R-Ras;RRAS;ortholog	small GTPase(PC00208)
HUMAN|HGNC=5582|UniProtKB=A0A0B4J1V0	Immunoglobulin heavy variable 3-15;IGHV3-15;ortholog	immunoglobulin(PC00123)
HUMAN|HGNC=9364|UniProtKB=Q92954	Proteoglycan 4;PRG4;ortholog	
HUMAN|HGNC=11319|UniProtKB=P28290	Protein ITPRID2;ITPRID2;ortholog	
HUMAN|HGNC=2252|UniProtKB=P31146	Coronin-1A;CORO1A;ortholog	non-motor actin binding protein(PC00165)
HUMAN|HGNC=12738|UniProtKB=Q969T9	WW domain-binding protein 2;WBP2;ortholog	
HUMAN|HGNC=5730|UniProtKB=A0A0C4DH73	Immunoglobulin kappa variable 1-12;IGKV1-12;ortholog	immunoglobulin(PC00123)
HUMAN|HGNC=5466|UniProtKB=P01344	Insulin-like growth factor II;IGF2;ortholog	growth factor(PC00112)
HUMAN|HGNC=16948|UniProtKB=Q969H8	Myeloid-derived growth factor;MYDGF;ortholog	growth factor(PC00112)
HUMAN|HGNC=5918|UniProtKB=A0A075B6K5	Immunoglobulin lambda variable 3-9;IGLV3-9;ortholog	immunoglobulin(PC00123)
HUMAN|HGNC=26340|UniProtKB=Q32M84	BTB/POZ domain-containing protein 16;BTBD16;ortholog	
HUMAN|HGNC=6461|UniProtKB=Q9NSB2	Keratin, type II cuticular Hb4;KRT84;ortholog	
HUMAN|HGNC=24552|UniProtKB=Q3LXA3	Triokinase/FMN cyclase;TKFC;ortholog	cyclase(PC00079)
HUMAN|HGNC=29380|UniProtKB=Q96ME7	Zinc finger protein 512;ZNF512;ortholog	
HUMAN|HGNC=16643|UniProtKB=Q14BN4	Sarcolemmal membrane-associated protein;SLMAP;ortholog	scaffold/adaptor protein(PC00226)

HUMAN|HGNC=913|UniProtKB=P20160	Azurocidin;AZU1;ortholog	serine protease(PC00203)
HUMAN|HGNC=2695|UniProtKB=Q16643	Drebrin;DBN1;ortholog	non-motor actin binding protein(PC00165)
HUMAN|HGNC=4393|UniProtKB=P11488	Guanine nucleotide-binding protein G(t) subunit alpha-1;GNAT1;ortholog	heterotrimeric G-protein(PC00117)
HUMAN|HGNC=4888|UniProtKB=Q03591	Complement factor H-related protein 1;CFHR1;ortholog	
HUMAN|HGNC=2712|UniProtKB=Q13561	Dynactin subunit 2;DCTN2;ortholog	microtubule binding motor protein(PC00156)
HUMAN|HGNC=7218|UniProtKB=P05164	Myeloperoxidase;MPO;ortholog	peroxidase(PC00180)
HUMAN|Gene=KV311_HUMAN|UniProtKB=P04433	Immunoglobulin kappa variable 3-11;IGKV3-11;ortholog	immunoglobulin(PC00123)
HUMAN|HGNC=1663|UniProtKB=P16671	Platelet glycoprotein 4;CD36;ortholog	membrane trafficking regulatory protein(PC00151)
HUMAN|HGNC=21295|UniProtKB=Q8TE68	Epidermal growth factor receptor kinase substrate 8-like protein 1;EPS8L1;ortholog	scaffold/adaptor protein(PC00226)
HUMAN|HGNC=8710|UniProtKB=Q9Y5G1	Protocadherin gamma-B3;PCDHGB3;ortholog	cadherin(PC00057)
HUMAN|HGNC=3551|UniProtKB=P00740	Coagulation factor IX;F9;ortholog	serine protease(PC00203)
HUMAN|HGNC=16272|UniProtKB=Q96JK9	Mastermind-like protein 3;MAML3;ortholog	transcription cofactor(PC00217)
HUMAN|HGNC=10898|UniProtKB=Q15477	Helicase SKI2W;SKIV2L;ortholog	DNA metabolism protein(PC00009)
HUMAN|HGNC=19860|UniProtKB=Q8NCU1	Uncharacterized protein CCDC197;CCDC197;ortholog	
HUMAN|HGNC=26068|UniProtKB=Q6IAA8	Ragulator complex protein LAMTOR1;LAMTOR1;ortholog	
HUMAN|HGNC=10432|UniProtKB=P51812	Ribosomal protein S6 kinase alpha-3;RPS6KA3;ortholog	protein modifying enzyme(PC00260)
HUMAN|HGNC=5929|UniProtKB=P04211	Immunoglobulin lambda variable 7-43;IGLV7-43;ortholog	immunoglobulin(PC00123)
HUMAN|HGNC=8993|UniProtKB=P12273	Prolactin-inducible protein;PIP;ortholog	
HUMAN|HGNC=30140|UniProtKB=A7MCY6	TANK-binding kinase 1-binding protein 1;TBKBP1;ortholog	
HUMAN|HGNC=17859|UniProtKB=Q5SRE5	Nucleoporin NUP188 homolog;NUP188;ortholog	
HUMAN|HGNC=1623|UniProtKB=P50990	T-complex protein 1 subunit theta;CCT8;ortholog	chaperonin(PC00073)
HUMAN|HGNC=6416|UniProtKB=P02533	Keratin, type I cytoskeletal 14;KRT14;ortholog	
HUMAN|HGNC=21726|UniProtKB=Q96PE2	Rho guanine nucleotide exchange factor 17;ARHGEF17;ortholog	guanyl-nucleotide exchange factor(PC00113)
HUMAN|HGNC=692|UniProtKB=P40616	ADP-ribosylation factor-like protein 1;ARL1;ortholog	G-protein(PC00020)
HUMAN|HGNC=5743|UniProtKB=A0A0C4DH67	Immunoglobulin kappa variable 1-8;IGKV1-8;ortholog	immunoglobulin(PC00123)
HUMAN|HGNC=3285|UniProtKB=P23588	Eukaryotic translation initiation factor 4B;EIF4B;ortholog	translation initiation factor(PC00224)

HUMAN|HGNC=23117|UniProtKB=Q6UWF7	NXPE family member 4;NXPE4;ortholog	
HUMAN|HGNC=15687|UniProtKB=O75563	Src kinase-associated phosphoprotein 2;SKAP2;ortholog	scaffold/adaptor protein(PC00226)
HUMAN|HGNC=9565|UniProtKB=P51665	26S proteasome non-ATPase regulatory subunit 7;PSMD7;ortholog	translation initiation factor(PC00224)
HUMAN|HGNC=6925|UniProtKB=P02686	Myelin basic protein;MBP;ortholog	myelin protein(PC00161)
HUMAN|HGNC=11446|UniProtKB=O00186	Syntaxin-binding protein 3;STXBP3;ortholog	membrane trafficking regulatory protein(PC00151)
HUMAN|HGNC=20165|UniProtKB=Q9P2P1	Protein NYNRIN;NYNRIN;ortholog	endoribonuclease(PC00094)
HUMAN|HGNC=20514|UniProtKB=Q8N257	Histone H2B type 3-B;H2BU1;ortholog	histone(PC00118)
HUMAN|HGNC=14111|UniProtKB=Q9HC38	Glyoxalase domain-containing protein 4;GLOD4;ortholog	
HUMAN|HGNC=2300|UniProtKB=Q96IY4	Carboxypeptidase B2;CPB2;ortholog	metalloprotease(PC00153)
HUMAN|HGNC=3481|UniProtKB=P13804	Electron transfer flavoprotein subunit alpha, mitochondrial;ETFA;ortholog	oxidoreductase(PC00176)
HUMAN|HGNC=4390|UniProtKB=P50148	Guanine nucleotide-binding protein G(q) subunit alpha;GNAQ;ortholog	heterotrimeric G-protein(PC00117)
HUMAN|HGNC=3230|UniProtKB=Q9NYQ7	Cadherin EGF LAG seven-pass G-type receptor 3;CELSR3;ortholog	cadherin(PC00057)
HUMAN|HGNC=5617|UniProtKB=A0A075B6Q5	Immunoglobulin heavy variable 3-64;IGHV3-64;ortholog	immunoglobulin(PC00123)
HUMAN|HGNC=12022|UniProtKB=P13693	Translationally-controlled tumor protein;TPT1;ortholog	non-motor microtubule binding protein(PC00166)
HUMAN|HGNC=16075|UniProtKB=Q9H082	Ras-related protein Rab-33B;RAB33B;ortholog	
HUMAN|HGNC=3537|UniProtKB=P25116	Proteinase-activated receptor 1;F2R;ortholog	G-protein coupled receptor(PC00021)
HUMAN|HGNC=15818|UniProtKB=Q9H0Z9	RNA-binding protein 38;RBM38;ortholog	
HUMAN|HGNC=8575|UniProtKB=P68402	Platelet-activating factor acetylhydrolase IB subunit beta;PAFAH1B2;ortholog	protein modifying enzyme(PC00260)
HUMAN|HGNC=5604|UniProtKB=A0A0B4J1X8	Immunoglobulin heavy variable 3-43;IGHV3-43;ortholog	immunoglobulin(PC00123)
HUMAN|HGNC=14683|UniProtKB=Q9Y2G5	GDP-fucose protein O-fucosyltransferase 2;POFUT2;ortholog	glycosyltransferase(PC00111)
HUMAN|HGNC=30535|UniProtKB=Q8WVJ2	NudC domain-containing protein 2;NUDCD2;ortholog	microtubule or microtubule-binding cytoskeletal protein(PC00157)
HUMAN|HGNC=29158|UniProtKB=Q9Y2G9	Protein strawberry notch homolog 2;SBNO2;ortholog	
HUMAN|HGNC=1618|UniProtKB=P48643	T-complex protein 1 subunit epsilon;CCT5;ortholog	chaperonin(PC00073)
HUMAN|HGNC=9750|UniProtKB=P20742	Pregnancy zone protein;PZP;ortholog	protease inhibitor(PC00191)
HUMAN|HGNC=620|UniProtKB=P05067	Amyloid-beta precursor protein;APP;ortholog	protease inhibitor(PC00191)

HUMAN|HGNC=1173|UniProtKB=Q5H9R7	Serine/threonine-protein phosphatase 6 regulatory subunit 3;PPP6R3;ortholog	phosphatase modulator(PC00184)
HUMAN|HGNC=9353|UniProtKB=P32119	Peroxiredoxin-2;PRDX2;ortholog	peroxidase(PC00180)
HUMAN|HGNC=4234|UniProtKB=P55040	GTP-binding protein GEM;GEM;ortholog	
HUMAN|HGNC=11594|UniProtKB=Q96SF7	T-box transcription factor TBX15;TBX15;ortholog	Rel homology transcription factor(PC00252)
HUMAN|HGNC=7665|UniProtKB=O43639	Cytoplasmic protein NCK2;NCK2;ortholog	scaffold/adaptor protein(PC00226)
HUMAN|HGNC=15902|UniProtKB=Q8IXH8	Cadherin-like protein 26;CDH26;ortholog	cadherin(PC00057)
HUMAN|HGNC=19983|UniProtKB=Q86YS6	Ras-related protein Rab-43;RAB43;ortholog	
HUMAN|HGNC=5931|UniProtKB=A0A075B6I0	Immunoglobulin lambda variable 8-61;IGLV8-61;ortholog	immunoglobulin(PC00123)
HUMAN|HGNC=1062|UniProtKB=P53004	Biliverdin reductase A;BLVRA;ortholog	dehydrogenase(PC00092)
HUMAN|HGNC=7642|UniProtKB=Q99747	Gamma-soluble NSF attachment protein;NAPG;ortholog	membrane traffic protein(PC00150)
HUMAN|HGNC=21354|UniProtKB=Q86XR7	TIR domain-containing adapter molecule 2;TICAM2;ortholog	vesicle coat protein(PC00235)
HUMAN|HGNC=25573|UniProtKB=Q8IYD2	Kelch domain-containing protein 8A;KLHDC8A;ortholog	
HUMAN|HGNC=23410|UniProtKB=Q969X1	Protein lifeguard 3;TMBIM1;ortholog	
HUMAN|HGNC=11429|UniProtKB=O75558	Syntaxin-11;STX11;ortholog	SNARE protein(PC00034)
HUMAN|HGNC=6400|UniProtKB=Q14974	Importin subunit beta-1;KPNB1;ortholog	transporter(PC00227)
HUMAN|HGNC=4622|UniProtKB=Q8IYD1	Eukaryotic peptide chain release factor GTP-binding subunit ERF3B;GSPT2;ortholog	translation factor(PC00223)
HUMAN|HGNC=25135|UniProtKB=Q6UWE0	E3 ubiquitin-protein ligase LRSAM1;LRSAM1;ortholog	
HUMAN|HGNC=6564|UniProtKB=Q08380	Galectin-3-binding protein;LGALS3BP;ortholog	
HUMAN|HGNC=851|UniProtKB=P38606	V-type proton ATPase catalytic subunit A;ATP6V1A;ortholog	ATP synthase(PC00002)
HUMAN|HGNC=1318|UniProtKB=P01024	Complement C3;C3;ortholog	protease inhibitor(PC00191)
HUMAN|HGNC=16391|UniProtKB=Q9H257	Caspase recruitment domain-containing protein 9;CARD9;ortholog	
HUMAN|HGNC=15842|UniProtKB=Q96RT1	Erbin;ERBIN;ortholog	scaffold/adaptor protein(PC00226)
HUMAN|HGNC=25272|UniProtKB=Q8TBB5	Kelch domain-containing protein 4;KLHDC4;ortholog	
HUMAN|Gene=EIF2S3L|UniProtKB=Q2VIR3	Eukaryotic translation initiation factor 2 subunit 3B;EIF2S3B;ortholog	translation initiation factor(PC00224)
HUMAN|HGNC=14451|UniProtKB=Q9BQK8	Phosphatidate phosphatase LPIN3;LPIN3;ortholog	
HUMAN|HGNC=5145|UniProtKB=P37235	Hippocalcin-like protein 1;HPCAL1;ortholog	
HUMAN|HGNC=24254|UniProtKB=Q96CN7	Isochorismatase domain-containing protein 1;ISOC1;ortholog	hydrolase(PC00121)

HUMAN|HGNC=2253|UniProtKB=Q9BR76	Coronin-1B;CORO1B;ortholog	non-motor actin binding protein(PC00165)
HUMAN|HGNC=16925|UniProtKB=Q96P48	Arf-GAP with Rho-GAP domain, ANK repeat and PH domain-containing protein 1;ARAP1;ortholog	GTPase-activating protein(PC00257)
HUMAN|HGNC=3423|UniProtKB=P11678	Eosinophil peroxidase;EPX;ortholog	peroxidase(PC00180)
HUMAN|HGNC=1133|UniProtKB=Q06187	Tyrosine-protein kinase BTK;BTK;ortholog	
HUMAN|HGNC=9075|UniProtKB=P08697	Alpha-2-antiplasmin;SERPINF2;ortholog	protease inhibitor(PC00191)
HUMAN|HGNC=17177|UniProtKB=Q8IUI8	Cytokine receptor-like factor 3;CRLF3;ortholog	DNA metabolism protein(PC00009)
HUMAN|HGNC=11756|UniProtKB=Q03403	Trefoil factor 2;TFF2;ortholog	intercellular signal molecule(PC00207)
HUMAN|HGNC=5880|UniProtKB=P01700	Immunoglobulin lambda variable 1-47;IGLV1-47;ortholog	immunoglobulin(PC00123)
HUMAN|HGNC=6444|UniProtKB=P04259	Keratin, type II cytoskeletal 6B;KRT6B;ortholog	
HUMAN|HGNC=17904|UniProtKB=Q9NRR4	Ribonuclease 3;DROSHA;ortholog	endoribonuclease(PC00094)
HUMAN|HGNC=39025|UniProtKB=Q9QC07	Endogenous retrovirus group K member 18 Pol protein;ERVK-18;ortholog	viral or transposable element protein(PC00237)
HUMAN|HGNC=23594|UniProtKB=Q709C8	Vacuolar protein sorting-associated protein 13C;VPS13C;ortholog	membrane traffic protein(PC00150)
HUMAN|HGNC=453|UniProtKB=P02760	Protein AMBP;AMBP;ortholog	
HUMAN|HGNC=10305|UniProtKB=P50914	60S ribosomal protein L14;RPL14;ortholog	ribosomal protein(PC00202)
HUMAN|HGNC=4433|UniProtKB=P00505	Aspartate aminotransferase, mitochondrial;GOT2;ortholog	transaminase(PC00216)
HUMAN|HGNC=19100|UniProtKB=Q5VWK5	Interleukin-23 receptor;IL23R;ortholog	transmembrane signal receptor(PC00197)
HUMAN|HGNC=25268|UniProtKB=Q96LR9	Apolipoprotein L domain-containing protein 1;APOLD1;ortholog	apolipoprotein(PC00052)
HUMAN|HGNC=163|UniProtKB=P12814	Alpha-actinin-1;ACTN1;ortholog	
HUMAN|HGNC=3542|UniProtKB=P12259	Coagulation factor V;F5;ortholog	oxidoreductase(PC00176)
HUMAN|HGNC=18370|UniProtKB=Q9H0U4	Ras-related protein Rab-1B;RAB1B;ortholog	
HUMAN|HGNC=21213|UniProtKB=Q5TH69	Brefeldin A-inhibited guanine nucleotide-exchange protein 3;ARFGEF3;ortholog	guanyl-nucleotide exchange factor(PC00113)
HUMAN|HGNC=10385|UniProtKB=P25398	40S ribosomal protein S12;RPS12;ortholog	ribosomal protein(PC00202)
HUMAN|HGNC=3311|UniProtKB=P30740	Leukocyte elastase inhibitor;SERPINB1;ortholog	protease inhibitor(PC00191)
HUMAN|HGNC=6840|UniProtKB=Q02750	Dual specificity mitogen-activated protein kinase kinase 1;MAP2K1;ortholog	non-receptor serine/threonine protein kinase(PC00167)
HUMAN|HGNC=4045|UniProtKB=O75084	Frizzled-7;FZD7;ortholog	transmembrane signal receptor(PC00197)
HUMAN|HGNC=17712|UniProtKB=O15304	Apoptosis regulatory protein Siva;SIVA1;ortholog	

HUMAN|HGNC=13726|UniProtKB=Q8NEZ4	Histone-lysine N-methyltransferase 2C;KMT2C;ortholog	
HUMAN|HGNC=7910|UniProtKB=P06748	Nucleophosmin;NPM1;ortholog	chaperone(PC00072)
HUMAN|HGNC=14871|UniProtKB=Q96A00	Protein phosphatase 1 regulatory subunit 14A;PPP1R14A;ortholog	phosphatase modulator(PC00184)
HUMAN|HGNC=9840|UniProtKB=P11234	Ras-related protein Ral-B;RALB;ortholog	small GTPase(PC00208)
HUMAN|HGNC=24550|UniProtKB=Q9H8H3	Methyltransferase-like protein 7A;METTL7A;ortholog	methyltransferase(PC00155)
HUMAN|HGNC=1622|UniProtKB=Q99832	T-complex protein 1 subunit eta;CCT7;ortholog	chaperonin(PC00073)
HUMAN|HGNC=11881|UniProtKB=P62328	Thymosin beta-4;TMSB4X;ortholog	
HUMAN|HGNC=3331|UniProtKB=P50402	Emerin;EMD;ortholog	
HUMAN|HGNC=11845|UniProtKB=Q9Y490	Talin-1;TLN1;ortholog	
HUMAN|HGNC=11119|UniProtKB=Q99835	Smoothened homolog;SMO;ortholog	transmembrane signal receptor(PC00197)
HUMAN|HGNC=16961|UniProtKB=Q15762	CD226 antigen;CD226;ortholog	
HUMAN|HGNC=6401|UniProtKB=Q92973	Transportin-1;TNPO1;ortholog	transporter(PC00227)
HUMAN|HGNC=11275|UniProtKB=Q01082	Spectrin beta chain, non-erythrocytic 1;SPTBN1;ortholog	
HUMAN|HGNC=2295|UniProtKB=P00450	Ceruloplasmin;CP;ortholog	oxidase(PC00175)
HUMAN|HGNC=10382|UniProtKB=P04844	Dolichyl-diphosphooligosaccharide--protein glycosyltransferase subunit 2;RPN2;ortholog	glycosyltransferase(PC00111)
HUMAN|HGNC=1441|UniProtKB=Q05682	Caldesmon;CALD1;ortholog	non-motor actin binding protein(PC00165)
HUMAN|HGNC=10594|UniProtKB=Q01118	Sodium channel protein type 7 subunit alpha;SCN7A;ortholog	voltage-gated ion channel(PC00241)
HUMAN|HGNC=12851|UniProtKB=P62258	14-3-3 protein epsilon;YWHAE;ortholog	scaffold/adaptor protein(PC00226)
HUMAN|HGNC=6138|UniProtKB=P08514	Integrin alpha-IIb;ITGA2B;ortholog	integrin(PC00126)
HUMAN|HGNC=9879|UniProtKB=Q7LDG7	RAS guanyl-releasing protein 2;RASGRP2;ortholog	guanyl-nucleotide exchange factor(PC00113)
HUMAN|HGNC=4187|UniProtKB=P02774	Vitamin D-binding protein;GC;ortholog	transfer/carrier protein(PC00219)
HUMAN|HGNC=469|UniProtKB=Q01433	AMP deaminase 2;AMPD2;ortholog	deaminase(PC00088)
HUMAN|HGNC=4755|UniProtKB=Q93079	Histone H2B type 1-H;H2BC9;ortholog	histone(PC00118)
HUMAN|HGNC=15461|UniProtKB=P55145	Mesencephalic astrocyte-derived neurotrophic factor;MANF;ortholog	
HUMAN|HGNC=12726|UniProtKB=P04275	von Willebrand factor;VWF;ortholog	extracellular matrix protein(PC00102)
HUMAN|HGNC=8784|UniProtKB=O76074	cGMP-specific 3',5'-cyclic phosphodiesterase;PDE5A;ortholog	phosphodiesterase(PC00185)

HUMAN|HGNC=9381|UniProtKB=P22694	cAMP-dependent protein kinase catalytic subunit beta;PRKACB;ortholog	non-receptor serine/threonine protein kinase(PC00167)
HUMAN|HGNC=4606|UniProtKB=P30101	Protein disulfide-isomerase A3;PDIA3;ortholog	chaperone(PC00072)
HUMAN|HGNC=15476|UniProtKB=P63167	Dynein light chain 1, cytoplasmic;DYNLL1;ortholog	microtubule or microtubule-binding cytoskeletal protein(PC00157)
HUMAN|HGNC=9540|UniProtKB=P49720	Proteasome subunit beta type-3;PSMB3;ortholog	protease(PC00190)
HUMAN|HGNC=13506|UniProtKB=Q8TBJ5	Fez family zinc finger protein 2;FEZF2;ortholog	C2H2 zinc finger transcription factor(PC00248)
HUMAN|HGNC=2951|UniProtKB=Q9C0G6	Dynein heavy chain 6, axonemal;DNAH6;ortholog	microtubule binding motor protein(PC00156)
HUMAN|HGNC=4248|UniProtKB=Q92820	Gamma-glutamyl hydrolase;GGH;ortholog	cysteine protease(PC00081)
HUMAN|HGNC=14952|UniProtKB=Q8TAE6	Protein phosphatase 1 regulatory subunit 14C;PPP1R14C;ortholog	phosphatase modulator(PC00184)
HUMAN|HGNC=3611|UniProtKB=P30273	High affinity immunoglobulin epsilon receptor subunit gamma;FCER1G;ortholog	immunoglobulin receptor superfamily(PC00124)
HUMAN|HGNC=17772|UniProtKB=Q99757	Thioredoxin, mitochondrial;TXN2;ortholog	oxidoreductase(PC00176)
HUMAN|HGNC=7237|UniProtKB=Q9Y6F6	Inositol 1,4,5-triphosphate receptor associated 1;IRAG1;ortholog	scaffold/adaptor protein(PC00226)
HUMAN|HGNC=3151|UniProtKB=P30084	Enoyl-CoA hydratase, mitochondrial;ECHS1;ortholog	hydratase(PC00120)
HUMAN|HGNC=4750|UniProtKB=Q99879	Histone H2B type 1-M;H2BC14;ortholog	histone(PC00118)
HUMAN|HGNC=15855|UniProtKB=Q9NPY3	Complement component C1q receptor;CD93;ortholog	extracellular matrix structural protein(PC00103)
HUMAN|HGNC=1122|UniProtKB=P43251	Biotinidase;BTD;ortholog	hydrolase(PC00121)
HUMAN|HGNC=414|UniProtKB=P04075	Fructose-bisphosphate aldolase A;ALDOA;ortholog	aldolase(PC00044)
HUMAN|HGNC=5746|UniProtKB=P01611	Immunoglobulin kappa variable 1D-12;IGKV1D-12;ortholog	immunoglobulin(PC00123)
HUMAN|HGNC=19312|UniProtKB=Q56P03	E2F-associated phosphoprotein;EAPP;ortholog	
HUMAN|HGNC=27146|UniProtKB=Q5TA31	E3 ubiquitin-protein ligase RNF187;RNF187;ortholog	ubiquitin-protein ligase(PC00234)
HUMAN|HGNC=5006|UniProtKB=P04035	3-hydroxy-3-methylglutaryl-coenzyme A reductase;HMGCR;ortholog	reductase(PC00198)
HUMAN|HGNC=18985|UniProtKB=Q7Z4W1	L-xylulose reductase;DCXR;ortholog	reductase(PC00198)
HUMAN|HGNC=3208|UniProtKB=P24534	Elongation factor 1-beta;EEF1B2;ortholog	translation elongation factor(PC00222)
HUMAN|HGNC=4456|UniProtKB=P43304	Glycerol-3-phosphate dehydrogenase, mitochondrial;GPD2;ortholog	dehydrogenase(PC00092)
HUMAN|HGNC=9411|UniProtKB=P14314	Glucosidase 2 subunit beta;PRKCSH;ortholog	protein-binding activity modulator(PC00095)

HUMAN|HGNC=24718|UniProtKB=Q96JJ7	Protein disulfide-isomerase TMX3;TMX3;ortholog	chaperone(PC00072)
HUMAN|HGNC=12690|UniProtKB=P09327	Villin-1;VIL1;ortholog	non-motor actin binding protein(PC00165)
HUMAN|HGNC=2062|UniProtKB=O00299	Chloride intracellular channel protein 1;CLIC1;ortholog	ion channel(PC00133)
HUMAN|HGNC=11172|UniProtKB=Q13596	Sorting nexin-1;SNX1;ortholog	scaffold/adaptor protein(PC00226)
HUMAN|HGNC=3711|UniProtKB=P62942	Peptidyl-prolyl cis-trans isomerase FKBP1A;FKBP1A;ortholog	chaperone(PC00072)
HUMAN|HGNC=20954|UniProtKB=Q9NP79	Vacuolar protein sorting-associated protein VTA1 homolog;VTA1;ortholog	membrane traffic protein(PC00150)
HUMAN|HGNC=705|UniProtKB=O15144	Actin-related protein 2/3 complex subunit 2;ARPC2;ortholog	
HUMAN|HGNC=17821|UniProtKB=O00148	ATP-dependent RNA helicase DDX39A;DDX39A;ortholog	
HUMAN|HGNC=4829|UniProtKB=P02042	Hemoglobin subunit delta;HBD;ortholog	
HUMAN|HGNC=1449|UniProtKB=P0DP25	Calmodulin-3;CALM3;ortholog	calmodulin-related(PC00061)
HUMAN|HGNC=2720|UniProtKB=Q9ULH1	Arf-GAP with SH3 domain, ANK repeat and PH domain-containing protein 1;ASAP1;ortholog	
HUMAN|HGNC=23169|UniProtKB=Q9Y224	RNA transcription, translation and transport factor protein;RTRAF;ortholog	
HUMAN|HGNC=5633|UniProtKB=A0A075B7D8	Ig-like domain-containing protein;IGHV3OR15-7;ortholog	immunoglobulin(PC00123)
HUMAN|HGNC=5821|UniProtKB=A0A075B6H7	Probable non-functional immunoglobulin kappa variable 3-7;IGKV3-7;ortholog	immunoglobulin(PC00123)
HUMAN|HGNC=4242|UniProtKB=O94808	Glutamine--fructose-6-phosphate aminotransferase [isomerizing] 2;GFPT2;ortholog	transaminase(PC00216)
HUMAN|HGNC=18344|UniProtKB=Q9BX68	Histidine triad nucleotide-binding protein 2, mitochondrial;HINT2;ortholog	nucleotide phosphatase(PC00173)
HUMAN|HGNC=9465|UniProtKB=P11908	Ribose-phosphate pyrophosphokinase 2;PRPS2;ortholog	nucleotide kinase(PC00172)
HUMAN|HGNC=1516|UniProtKB=P04040	Catalase;CAT;ortholog	peroxidase(PC00180)
HUMAN|HGNC=5156|UniProtKB=P00739	Haptoglobin-related protein;HPR;ortholog	serine protease(PC00203)
HUMAN|HGNC=4057|UniProtKB=P11413	Glucose-6-phosphate 1-dehydrogenase;G6PD;ortholog	dehydrogenase(PC00092)
HUMAN|HGNC=10823|UniProtKB=O75368	SH3 domain-binding glutamic acid-rich-like protein;SH3BGRL;ortholog	
HUMAN|HGNC=8527|UniProtKB=P55809	Succinyl-CoA:3-ketoacid coenzyme A transferase 1, mitochondrial;OXCT1;ortholog	transferase(PC00220)
HUMAN|HGNC=29557|UniProtKB=Q0ZGT2	Nexilin;NEXN;ortholog	scaffold/adaptor protein(PC00226)
HUMAN|HGNC=9635|UniProtKB=Q12974	Protein tyrosine phosphatase type IVA 2;PTP4A2;ortholog	protein phosphatase(PC00195)
HUMAN|HGNC=5756|UniProtKB=P04432	Immunoglobulin kappa variable 1D-39;IGKV1D-39;ortholog	immunoglobulin(PC00123)
HUMAN|HGNC=9992|UniProtKB=O43665	Regulator of G-protein signaling 10;RGS10;ortholog	
HUMAN|HGNC=3702|UniProtKB=Q13642	Four and a half LIM domains protein 1;FHL1;ortholog	

HUMAN|HGNC=29534|UniProtKB=Q9BSJ8	Extended synaptotagmin-1;ESYT1;ortholog	
HUMAN|HGNC=24641|UniProtKB=Q7Z3J2	VPS35 endosomal protein sorting factor-like;VPS35L;ortholog	
HUMAN|HGNC=28771|UniProtKB=Q9BVC6	Transmembrane protein 109;TMEM109;ortholog	
HUMAN|HGNC=5036|UniProtKB=Q14103	Heterogeneous nuclear ribonucleoprotein D0;HNRNPD;ortholog	
HUMAN|HGNC=2734|UniProtKB=Q92499	ATP-dependent RNA helicase DDX1;DDX1;ortholog	
HUMAN|HGNC=1801|UniProtKB=O95674	Phosphatidate cytidylyltransferase 2;CDS2;ortholog	transferase(PC00220)
HUMAN|HGNC=4684|UniProtKB=P33402	Guanylate cyclase soluble subunit alpha-2;GUCY1A2;ortholog	guanylate cyclase(PC00114)
HUMAN|HGNC=20635|UniProtKB=Q14997	Proteasome activator complex subunit 4;PSME4;ortholog	
HUMAN|HGNC=12841|UniProtKB=P07947	Tyrosine-protein kinase Yes;YES1;ortholog	
HUMAN|HGNC=11034|UniProtKB=Q2Y0W8	Electroneutral sodium bicarbonate exchanger 1;SLC4A8;ortholog	secondary carrier transporter(PC00258)
HUMAN|HGNC=9355|UniProtKB=P30044	Peroxiredoxin-5, mitochondrial;PRDX5;ortholog	oxidoreductase(PC00176)
HUMAN|HGNC=10869|UniProtKB=Q92185	Alpha-N-acetylneuraminide alpha-2,8-sialyltransferase;ST8SIA1;ortholog	transferase(PC00220)
HUMAN|HGNC=885|UniProtKB=O75882	Attractin;ATRN;ortholog	extracellular matrix protein(PC00102)
HUMAN|HGNC=4623|UniProtKB=P00390	Glutathione reductase, mitochondrial;GSR;ortholog	reductase(PC00198)
HUMAN|HGNC=11274|UniProtKB=P11277	Spectrin beta chain, erythrocytic;SPTB;ortholog	
HUMAN|HGNC=7967|UniProtKB=Q96RI1	Bile acid receptor;NR1H4;ortholog	C4 zinc finger nuclear receptor(PC00169)
HUMAN|HGNC=26525|UniProtKB=Q6P387	Uncharacterized protein C16orf46;C16orf46;ortholog	
HUMAN|HGNC=17059|UniProtKB=Q9NTJ5	Phosphatidylinositol-3-phosphatase SAC1;SACM1L;ortholog	phosphatase(PC00181)
HUMAN|HGNC=24013|UniProtKB=Q5QJE6	Deoxynucleotidyltransferase terminal-interacting protein 2;DNTTIP2;ortholog	
HUMAN|HGNC=18587|UniProtKB=O94901	SUN domain-containing protein 1;SUN1;ortholog	non-motor microtubule binding protein(PC00166)
HUMAN|HGNC=12401|UniProtKB=P33981	Dual specificity protein kinase TTK;TTK;ortholog	non-receptor serine/threonine protein kinase(PC00167)
HUMAN|HGNC=3754|UniProtKB=P21333	Filamin-A;FLNA;ortholog	
HUMAN|HGNC=17038|UniProtKB=Q99685	Monoglyceride lipase;MGLL;ortholog	phospholipase(PC00186)
HUMAN|HGNC=3214|UniProtKB=P13639	Elongation factor 2;EEF2;ortholog	translation elongation factor(PC00222)
HUMAN|HGNC=32060|UniProtKB=Q5TEC6	Histone H3;H3-2;ortholog	histone(PC00118)
HUMAN|HGNC=9537|UniProtKB=P20618	Proteasome subunit beta type-1;PSMB1;ortholog	protease(PC00190)

HUMAN|HGNC=26533|UniProtKB=Q96M83	Coiled-coil domain-containing protein 7;CCDC7;ortholog	
HUMAN|HGNC=1458|UniProtKB=O43852	Calumenin;CALU;ortholog	calmodulin-related(PC00061)
HUMAN|HGNC=6902|UniProtKB=O00187	Mannan-binding lectin serine protease 2;MASP2;ortholog	serine protease(PC00203)
HUMAN|HGNC=4566|UniProtKB=P62993	Growth factor receptor-bound protein 2;GRB2;ortholog	
HUMAN|HGNC=11491|UniProtKB=P43405	Tyrosine-protein kinase SYK;SYK;ortholog	
HUMAN|HGNC=3200|UniProtKB=Q5VTE0	Putative elongation factor 1-alpha-like 3;EEF1A1P5;ortholog	translation factor(PC00223)
HUMAN|HGNC=18411|UniProtKB=Q3KNW1	Zinc finger protein SNAI3;SNAI3;ortholog	C2H2 zinc finger transcription factor(PC00248)
HUMAN|HGNC=26212|UniProtKB=P61009	Signal peptidase complex subunit 3;SPCS3;ortholog	protease(PC00190)
HUMAN|HGNC=8498|UniProtKB=P02763	Alpha-1-acid glycoprotein 1;ORM1;ortholog	
HUMAN|HGNC=14902|UniProtKB=Q9NRW1	Ras-related protein Rab-6B;RAB6B;ortholog	
HUMAN|HGNC=143|UniProtKB=P68032	Actin, alpha cardiac muscle 1;ACTC1;ortholog	actin and actin related protein(PC00039)
HUMAN|HGNC=3048|UniProtKB=Q02413	Desmoglein-1;DSG1;ortholog	cadherin(PC00057)
HUMAN|HGNC=5732|UniProtKB=P04430	Immunoglobulin kappa variable 1-16;IGKV1-16;ortholog	immunoglobulin(PC00123)
HUMAN|HGNC=15447|UniProtKB=Q9Y4G6	Talin-2;TLN2;ortholog	
HUMAN|HGNC=1990|UniProtKB=Q8WWK9	Cytoskeleton-associated protein 2;CKAP2;ortholog	non-motor microtubule binding protein(PC00166)
HUMAN|HGNC=24437|UniProtKB=Q96KP4	Cytosolic non-specific dipeptidase;CNDP2;ortholog	metalloprotease(PC00153)
HUMAN|HGNC=17196|UniProtKB=Q8NG08	DNA helicase B;HELB;ortholog	RNA helicase(PC00032)
HUMAN|HGNC=5585|UniProtKB=A0A0C4DH32	Immunoglobulin heavy variable 3-20;IGHV3-20;ortholog	immunoglobulin(PC00123)
HUMAN|HGNC=4385|UniProtKB=P04899	Guanine nucleotide-binding protein G(i) subunit alpha-2;GNAI2;ortholog	heterotrimeric G-protein(PC00117)
HUMAN|HGNC=9559|UniProtKB=Q13200	26S proteasome non-ATPase regulatory subunit 2;PSMD2;ortholog	protease(PC00190)
HUMAN|HGNC=343|UniProtKB=P23526	Adenosylhomocysteinase;AHCY;ortholog	hydrolase(PC00121)
HUMAN|HGNC=10045|UniProtKB=P10153	Non-secretory ribonuclease;RNASE2;ortholog	
HUMAN|HGNC=16996|UniProtKB=Q15363	Transmembrane emp24 domain-containing protein 2;TMED2;ortholog	vesicle coat protein(PC00235)
HUMAN|HGNC=5896|UniProtKB=P01715	Immunoglobulin lambda variable 3-1;IGLV3-1;ortholog	immunoglobulin(PC00123)
HUMAN|HGNC=18144|UniProtKB=Q8IZU8	Dermatan-sulfate epimerase-like protein;DSEL;ortholog	
HUMAN|HGNC=9314|UniProtKB=Q08209	Serine/threonine-protein phosphatase 2B catalytic subunit alpha isoform;PPP3CA;ortholog	protein phosphatase(PC00195)

HUMAN|HGNC=412|UniProtKB=P49189	4-trimethylaminobutyraldehyde dehydrogenase;ALDH9A1;ortholog	dehydrogenase(PC00092)
HUMAN|HGNC=4208|UniProtKB=P23434	Glycine cleavage system H protein, mitochondrial;GCSH;ortholog	
HUMAN|HGNC=4798|UniProtKB=Q14520	Hyaluronan-binding protein 2;HABP2;ortholog	serine protease(PC00203)
HUMAN|HGNC=1738|UniProtKB=Q9Y5S2	Serine/threonine-protein kinase MRCK beta;CDC42BPB;ortholog	non-receptor serine/threonine protein kinase(PC00167)
HUMAN|HGNC=11219|UniProtKB=P09486	SPARC;SPARC;ortholog	extracellular matrix glycoprotein(PC00100)
HUMAN|HGNC=654|UniProtKB=P61204	ADP-ribosylation factor 3;ARF3;ortholog	G-protein(PC00020)
HUMAN|HGNC=1717|UniProtKB=Q16181	Septin-7;SEPTIN7;ortholog	cytoskeletal protein(PC00085)
HUMAN|HGNC=16268|UniProtKB=Q8IY17	Patatin-like phospholipase domain-containing protein 6;PNPLA6;ortholog	esterase(PC00097)
HUMAN|HGNC=20726|UniProtKB=Q8WVM8	Sec1 family domain-containing protein 1;SCFD1;ortholog	membrane trafficking regulatory protein(PC00151)
HUMAN|HGNC=26406|UniProtKB=Q96NL6	Sodium channel and clathrin linker 1;SCLT1;ortholog	
HUMAN|HGNC=9801|UniProtKB=P63000	Ras-related C3 botulinum toxin substrate 1;RAC1;ortholog	small GTPase(PC00208)
HUMAN|HGNC=15750|UniProtKB=P60981	Destrin;DSTN;ortholog	non-motor actin binding protein(PC00165)
HUMAN|HGNC=11512|UniProtKB=Q9H2B2	Synaptotagmin-4;SYT4;ortholog	membrane trafficking regulatory protein(PC00151)
HUMAN|HGNC=7007|UniProtKB=P61006	Ras-related protein Rab-8A;RAB8A;ortholog	
HUMAN|HGNC=29353|UniProtKB=Q6PI77	Protein BHLHb9;BHLHB9;ortholog	basic helix-loop-helix transcription factor(PC00055)
HUMAN|HGNC=6167|UniProtKB=P19823	Inter-alpha-trypsin inhibitor heavy chain H2;ITIH2;ortholog	protease inhibitor(PC00191)
HUMAN|HGNC=626|UniProtKB=P07741	Adenine phosphoribosyltransferase;APRT;ortholog	transferase(PC00220)
HUMAN|HGNC=1393|UniProtKB=O60840	Voltage-dependent L-type calcium channel subunit alpha-1F;CACNA1F;ortholog	voltage-gated ion channel(PC00241)
HUMAN|HGNC=9759|UniProtKB=P61026	Ras-related protein Rab-10;RAB10;ortholog	
HUMAN|HGNC=25951|UniProtKB=Q6ULP2	Aftiphilin;AFTPH;ortholog	
HUMAN|HGNC=1548|UniProtKB=P16152	Carbonyl reductase [NADPH] 1;CBR1;ortholog	reductase(PC00198)
HUMAN|HGNC=837|UniProtKB=P30049	ATP synthase subunit delta, mitochondrial;ATP5F1D;ortholog	ATP synthase(PC00002)
HUMAN|HGNC=7183|UniProtKB=P41218	Myeloid cell nuclear differentiation antigen;MNDA;ortholog	DNA-binding transcription factor(PC00218)
HUMAN|HGNC=26427|UniProtKB=Q96ND8	Zinc finger protein 583;ZNF583;ortholog	
HUMAN|HGNC=6091|UniProtKB=P06213	Insulin receptor;INSR;ortholog	transmembrane signal receptor(PC00197)

HUMAN|HGNC=22934|UniProtKB=Q8NAB2	Kelch repeat and BTB domain-containing protein 3;KBTBD3;ortholog	scaffold/adaptor protein(PC00226)
HUMAN|HGNC=8997|UniProtKB=P48426	Phosphatidylinositol 5-phosphate 4-kinase type-2 alpha;PIP4K2A;ortholog	kinase(PC00137)
HUMAN|HGNC=4838|UniProtKB=P05546	Heparin cofactor 2;SERPIND1;ortholog	protease inhibitor(PC00191)
HUMAN|HGNC=5044|UniProtKB=P61978	Heterogeneous nuclear ribonucleoprotein K;HNRNPK;ortholog	RNA metabolism protein(PC00031)
HUMAN|HGNC=7900|UniProtKB=P55786	Puromycin-sensitive aminopeptidase;NPEPPS;ortholog	metalloprotease(PC00153)
HUMAN|HGNC=9772|UniProtKB=Q13637	Ras-related protein Rab-32;RAB32;ortholog	
HUMAN|HGNC=9531|UniProtKB=P25787	Proteasome subunit alpha type-2;PSMA2;ortholog	protease(PC00190)
HUMAN|HGNC=11358|UniProtKB=O75886	Signal transducing adapter molecule 2;STAM2;ortholog	scaffold/adaptor protein(PC00226)
HUMAN|HGNC=6529|UniProtKB=Q13094	Lymphocyte cytosolic protein 2;LCP2;ortholog	scaffold/adaptor protein(PC00226)
HUMAN|HGNC=6499|UniProtKB=P11279	Lysosome-associated membrane glycoprotein 1;LAMP1;ortholog	membrane trafficking regulatory protein(PC00151)
HUMAN|HGNC=14882|UniProtKB=Q9NQ79	Cartilage acidic protein 1;CRTAC1;ortholog	
HUMAN|HGNC=1328|UniProtKB=P20851	C4b-binding protein beta chain;C4BPB;ortholog	
HUMAN|HGNC=24054|UniProtKB=Q8NG31	Kinetochore scaffold 1;KNL1;ortholog	
HUMAN|HGNC=2551|UniProtKB=Q13616	Cullin-1;CUL1;ortholog	ubiquitin-protein ligase(PC00234)
HUMAN|HGNC=9556|UniProtKB=O00231	26S proteasome non-ATPase regulatory subunit 11;PSMD11;ortholog	
HUMAN|HGNC=3300|UniProtKB=P63241	Eukaryotic translation initiation factor 5A-1;EIF5A;ortholog	translation initiation factor(PC00224)
HUMAN|HGNC=11590|UniProtKB=Q16650	T-box brain protein 1;TBR1;ortholog	Rel homology transcription factor(PC00252)
HUMAN|HGNC=30646|UniProtKB=Q7KZF4	Staphylococcal nuclease domain-containing protein 1;SND1;ortholog	
HUMAN|HGNC=16273|UniProtKB=Q96A49	Synapse-associated protein 1;SYAP1;ortholog	
HUMAN|HGNC=6325|UniProtKB=O60282	Kinesin heavy chain isoform 5C;KIF5C;ortholog	microtubule binding motor protein(PC00156)
HUMAN|HGNC=17780|UniProtKB=Q562R1	Beta-actin-like protein 2;ACTBL2;ortholog	actin and actin related protein(PC00039)
HUMAN|HGNC=4749|UniProtKB=Q99877	Histone H2B type 1-N;H2BC15;ortholog	histone(PC00118)
HUMAN|HGNC=8601|UniProtKB=Q06141	Regenerating islet-derived protein 3-alpha;REG3A;ortholog	
HUMAN|HGNC=9462|UniProtKB=P60891	Ribose-phosphate pyrophosphokinase 1;PRPS1;ortholog	nucleotide kinase(PC00172)
HUMAN|HGNC=17305|UniProtKB=Q9Y6J9	TAF6-like RNA polymerase II p300/CBP-associated factor-associated factor 65 kDa subunit 6L;TAF6L;ortholog	general transcription factor(PC00259)
HUMAN|HGNC=1241|UniProtKB=P02745	Complement C1q subcomponent subunit A;C1QA;ortholog	

HUMAN|HGNC=5735|UniProtKB=A0A075B6S5	Immunoglobulin kappa variable 1-27;IGKV1-27;ortholog	immunoglobulin(PC00123)
HUMAN|HGNC=4387|UniProtKB=P08754	Guanine nucleotide-binding protein G(i) subunit alpha;GNAI3;ortholog	heterotrimeric G-protein(PC00117)
HUMAN|HGNC=29801|UniProtKB=Q7L9L4	MOB kinase activator 1B;MOB1B;ortholog	kinase activator(PC00138)
HUMAN|HGNC=37215|UniProtKB=A6NIK2	Leucine-rich repeat-containing protein 10B;LRRC10B;ortholog	
HUMAN|HGNC=12628|UniProtKB=P45974	Ubiquitin carboxyl-terminal hydrolase 5;USP5;ortholog	cysteine protease(PC00081)
HUMAN|HGNC=30052|UniProtKB=Q8TEM1	Nuclear pore membrane glycoprotein 210;NUP210;ortholog	transporter(PC00227)
HUMAN|HGNC=1869|UniProtKB=P11597	Cholesteryl ester transfer protein;CETP;ortholog	
HUMAN|HGNC=26801|UniProtKB=Q8N8Q8	Cytochrome c oxidase assembly protein COX18, mitochondrial;COX18;ortholog	transporter(PC00227)
HUMAN|HGNC=12976|UniProtKB=O15231	Zinc finger protein 185;ZNF185;ortholog	
HUMAN|HGNC=1368|UniProtKB=P00915	Carbonic anhydrase 1;CA1;ortholog	
HUMAN|HGNC=6510|UniProtKB=P16949	Stathmin;STMN1;ortholog	
HUMAN|HGNC=4699|UniProtKB=P46976	Glycogenin-1;GYG1;ortholog	glycosyltransferase(PC00111)
HUMAN|HGNC=21101|UniProtKB=O60888	Protein CutA;CUTA;ortholog	primary active transporter(PC00068)
HUMAN|HGNC=28883|UniProtKB=Q9H2U2	Inorganic pyrophosphatase 2, mitochondrial;PPA2;ortholog	pyrophosphatase(PC00196)
HUMAN|HGNC=5601|UniProtKB=A0A0C4DH36	Probable non-functional immunoglobulin heavy variable 3-38;IGHV3-38;ortholog	immunoglobulin(PC00123)
HUMAN|HGNC=9380|UniProtKB=P17612	cAMP-dependent protein kinase catalytic subunit alpha;PRKACA;ortholog	non-receptor serine/threonine protein kinase(PC00167)
HUMAN|HGNC=15912|UniProtKB=Q9UNZ2	NSFL1 cofactor p47;NSFL1C;ortholog	protein-binding activity modulator(PC00095)
HUMAN|HGNC=13200|UniProtKB=Q15942	Zyxin;ZYX;ortholog	actin or actin-binding cytoskeletal protein(PC00041)
HUMAN|HGNC=25153|UniProtKB=Q96EY5	Multivesicular body subunit 12A;MVB12A;ortholog	
HUMAN|HGNC=9935|UniProtKB=Q14257	Reticulocalbin-2;RCN2;ortholog	calmodulin-related(PC00061)
HUMAN|HGNC=1228|UniProtKB=P05155	Plasma protease C1 inhibitor;SERPING1;ortholog	protease inhibitor(PC00191)
HUMAN|HGNC=17102|UniProtKB=Q92619	Rho GTPase-activating protein 45;ARHGAP45;ortholog	
HUMAN|HGNC=12853|UniProtKB=Q04917	14-3-3 protein eta;YWHAH;ortholog	scaffold/adaptor protein(PC00226)
HUMAN|HGNC=18673|UniProtKB=Q96JB5	CDK5 regulatory subunit-associated protein 3;CDK5RAP3;ortholog	
HUMAN|HGNC=6439|UniProtKB=P35908	Keratin, type II cytoskeletal 2 epidermal;KRT2;ortholog	
HUMAN|HGNC=12854|UniProtKB=P27348	14-3-3 protein theta;YWHAQ;ortholog	scaffold/adaptor protein(PC00226)

HUMAN|HGNC=4444|UniProtKB=P14770	Platelet glycoprotein IX;GP9;ortholog	
HUMAN|HGNC=1358|UniProtKB=P02748	Complement component C9;C9;ortholog	
HUMAN|HGNC=4926|UniProtKB=O15212	Prefoldin subunit 6;PFDN6;ortholog	
HUMAN|HGNC=16|UniProtKB=P01011	Alpha-1-antichymotrypsin;SERPINA3;ortholog	protease inhibitor(PC00191)
HUMAN|HGNC=5526|UniProtKB=P01859	Immunoglobulin heavy constant gamma 2;IGHG2;ortholog	immunoglobulin(PC00123)
HUMAN|HGNC=9532|UniProtKB=P25788	Proteasome subunit alpha type-3;PSMA3;ortholog	protease(PC00190)
HUMAN|HGNC=12672|UniProtKB=P45880	Voltage-dependent anion-selective channel protein 2;VDAC2;ortholog	voltage-gated ion channel(PC00241)
HUMAN|HGNC=20|UniProtKB=P49588	Alanine--tRNA ligase, cytoplasmic;AARS1;ortholog	aminoacyl-tRNA synthetase(PC00047)
HUMAN|HGNC=19347|UniProtKB=O14529	Homeobox protein cut-like 2;CUX2;ortholog	homeodomain transcription factor(PC00119)
HUMAN|HGNC=11256|UniProtKB=Q13103	Secreted phosphoprotein 24;SPP2;ortholog	
HUMAN|HGNC=2444|UniProtKB=P41240	Tyrosine-protein kinase CSK;CSK;ortholog	
HUMAN|HGNC=18170|UniProtKB=P30085	UMP-CMP kinase;CMPK1;ortholog	nucleotide kinase(PC00172)
HUMAN|HGNC=28101|UniProtKB=Q2M2H8	Probable maltase-glucoamylase 2;MGAM2;ortholog	glucosidase(PC00108)
HUMAN|HGNC=735|UniProtKB=Q13510	Acid ceramidase;ASAH1;ortholog	cysteine protease(PC00081)
HUMAN|HGNC=7547|UniProtKB=P10243	Myb-related protein A;MYBL1;ortholog	
HUMAN|HGNC=10719|UniProtKB=Q13228	Methanethiol oxidase;SELENBP1;ortholog	defense/immunity protein(PC00090)
HUMAN|HGNC=12436|UniProtKB=O43396	Thioredoxin-like protein 1;TXNL1;ortholog	oxidoreductase(PC00176)
HUMAN|HGNC=7629|UniProtKB=E9PAV3	Nascent polypeptide-associated complex subunit alpha, muscle-specific form;NACA;ortholog	basic helix-loop-helix transcription factor(PC00055)
HUMAN|HGNC=10464|UniProtKB=Q15404	Ras suppressor protein 1;RSU1;ortholog	scaffold/adaptor protein(PC00226)
HUMAN|Gene=ENH1_HUMAN|UniProtKB=Q9N2K0	HERV-H_2q24.3 provirus ancestral Env polyprotein;-;ortholog	viral or transposable element protein(PC00237)
HUMAN|HGNC=24087|UniProtKB=Q0VD83	Apolipoprotein B receptor;APOBR;ortholog	
HUMAN|HGNC=11617|UniProtKB=Q15369	Elongin-C;ELOC;ortholog	general transcription factor(PC00259)
HUMAN|HGNC=16867|UniProtKB=O75150	E3 ubiquitin-protein ligase BRE1B;RNF40;ortholog	
HUMAN|HGNC=24808|UniProtKB=O14545	TRAF-type zinc finger domain-containing protein 1;TRAFD1;ortholog	
HUMAN|HGNC=14653|UniProtKB=Q9HBI1	Beta-parvin;PARVB;ortholog	actin and actin related protein(PC00039)
HUMAN|HGNC=3383|UniProtKB=P27105	Erythrocyte band 7 integral membrane protein;STOM;ortholog	cytoskeletal protein(PC00085)

HUMAN|HGNC=30025|UniProtKB=Q5VV67	Peroxisome proliferator-activated receptor gamma coactivator-related protein 1;PPRC1;ortholog	transcription cofactor(PC00217)
HUMAN|HGNC=5908|UniProtKB=P01717	Immunoglobulin lambda variable 3-25;IGLV3-25;ortholog	immunoglobulin(PC00123)
HUMAN|HGNC=17169|UniProtKB=Q13162	Peroxiredoxin-4;PRDX4;ortholog	peroxidase(PC00180)
HUMAN|HGNC=12420|UniProtKB=P49411	Elongation factor Tu, mitochondrial;TUFM;ortholog	translation elongation factor(PC00222)
HUMAN|HGNC=21696|UniProtKB=Q9Y3Q8	TSC22 domain family protein 4;TSC22D4;ortholog	
HUMAN|HGNC=11891|UniProtKB=P05452	Tetranectin;CLEC3B;ortholog	intercellular signal molecule(PC00207)
HUMAN|HGNC=11453|UniProtKB=P50225	Sulfotransferase 1A1;SULT1A1;ortholog	transferase(PC00220)
HUMAN|HGNC=10519|UniProtKB=Q9NZJ4	Sacsin;SACS;ortholog	
HUMAN|HGNC=20710|UniProtKB=Q9BTP6	Zinc finger BED domain-containing protein 2;ZBED2;ortholog	
HUMAN|HGNC=26486|UniProtKB=Q6PJW8	Consortin;CNST;ortholog	
HUMAN|HGNC=29141|UniProtKB=O94967	WD repeat-containing protein 47;WDR47;ortholog	
HUMAN|HGNC=3282|UniProtKB=P60842	Eukaryotic initiation factor 4A-I;EIF4A1;ortholog	
HUMAN|HGNC=5576|UniProtKB=P01817	Immunoglobulin heavy variable 2-5;IGHV2-5;ortholog	immunoglobulin(PC00123)
HUMAN|HGNC=12733|UniProtKB=Q9Y6W5	Wiskott-Aldrich syndrome protein family member 2;WASF2;ortholog	non-motor actin binding protein(PC00165)
HUMAN|HGNC=10774|UniProtKB=P23246	Splicing factor, proline- and glutamine-rich;SFPQ;ortholog	RNA metabolism protein(PC00031)
HUMAN|HGNC=3756|UniProtKB=Q14315	Filamin-C;FLNC;ortholog	
HUMAN|HGNC=18271|UniProtKB=Q8N8S7	Protein enabled homolog;ENAH;ortholog	scaffold/adaptor protein(PC00226)
HUMAN|HGNC=11655|UniProtKB=P17987	T-complex protein 1 subunit alpha;TCP1;ortholog	chaperonin(PC00073)
HUMAN|HGNC=27023|UniProtKB=A6NMZ7	Collagen alpha-6(VI) chain;COL6A6;ortholog	
HUMAN|HGNC=5610|UniProtKB=P01767	Immunoglobulin heavy variable 3-53;IGHV3-53;ortholog	immunoglobulin(PC00123)
HUMAN|HGNC=5882|UniProtKB=P01701	Immunoglobulin lambda variable 1-51;IGLV1-51;ortholog	immunoglobulin(PC00123)
HUMAN|HGNC=667|UniProtKB=P61586	Transforming protein RhoA;RHOA;ortholog	small GTPase(PC00208)
HUMAN|HGNC=27257|UniProtKB=Q3MIP1	Inositol 1,4,5-trisphosphate receptor-interacting protein-like 2;ITPRIPL2;ortholog	
HUMAN|HGNC=3718|UniProtKB=P26885	Peptidyl-prolyl cis-trans isomerase FKBP2;FKBP2;ortholog	
HUMAN|HGNC=1692|UniProtKB=P08962	CD63 antigen;CD63;ortholog	
HUMAN|HGNC=746|UniProtKB=P04424	Argininosuccinate lyase;ASL;ortholog	lyase(PC00144)
HUMAN|HGNC=2254|UniProtKB=Q9ULV4	Coronin-1C;CORO1C;ortholog	non-motor actin binding protein(PC00165)

HUMAN|HGNC=13312|UniProtKB=P78417	Glutathione S-transferase omega-1;GSTO1;ortholog	
HUMAN|HGNC=9700|UniProtKB=Q8IVU1	Immunoglobulin superfamily DCC subclass member 3;IGDCC3;ortholog	
HUMAN|HGNC=13529|UniProtKB=Q9Y2B0	Protein canopy homolog 2;CNPY2;ortholog	
HUMAN|HGNC=5261|UniProtKB=P10809	60 kDa heat shock protein, mitochondrial;HSPD1;ortholog	
HUMAN|HGNC=2761|UniProtKB=P59665	Neutrophil defensin 1;DEFA1;ortholog	antimicrobial response protein(PC00051)
HUMAN|HGNC=24607|UniProtKB=Q86VF2	Immunoglobulin-like and fibronectin type III domain-containing protein 1;IGFN1;ortholog	
HUMAN|HGNC=5739|UniProtKB=A0A075B6S9	Probable non-functional immunoglobulinn kappa variable 1-37;IGKV1-37;ortholog	immunoglobulin(PC00123)
HUMAN|HGNC=30306|UniProtKB=Q99623	Prohibitin-2;PHB2;ortholog	
HUMAN|Ensembl=ENSG00000282150|UniProtKB=A0A0J9YY99	Ig-like domain-containing protein;ENSG00000282150;ortholog	immunoglobulin(PC00123)
HUMAN|HGNC=4621|UniProtKB=P15170	Eukaryotic peptide chain release factor GTP-binding subunit ERF3A;GSPT1;ortholog	translation factor(PC00223)
HUMAN|HGNC=9504|UniProtKB=O43739	Cytohesin-3;CYTH3;ortholog	guanyl-nucleotide exchange factor(PC00113)
HUMAN|HGNC=11007|UniProtKB=P11169	Solute carrier family 2, facilitated glucose transporter member 3;SLC2A3;ortholog	
HUMAN|HGNC=5541|UniProtKB=P01871	Immunoglobulin heavy constant mu;IGHM;ortholog	immunoglobulin(PC00123)
HUMAN|HGNC=2520|UniProtKB=Q9NRF8	CTP synthase 2;CTPS2;ortholog	ligase(PC00142)
HUMAN|HGNC=17497|UniProtKB=Q8WZA0	Protein LZIC;LZIC;ortholog	
HUMAN|HGNC=568|UniProtKB=O14617	AP-3 complex subunit delta-1;AP3D1;ortholog	transporter(PC00227)
HUMAN|HGNC=806|UniProtKB=P54709	Sodium/potassium-transporting ATPase subunit beta-3;ATP1B3;ortholog	primary active transporter(PC00068)
HUMAN|HGNC=11984|UniProtKB=Q6ZVM7	TOM1-like protein 2;TOM1L2;ortholog	
HUMAN|HGNC=8010|UniProtKB=Q9Y4C0	Neurexin-3;NRXN3;ortholog	
HUMAN|HGNC=6387|UniProtKB=Q07866	Kinesin light chain 1;KLC1;ortholog	microtubule binding motor protein(PC00156)
HUMAN|HGNC=188|UniProtKB=O14672	Disintegrin and metalloproteinase domain-containing protein 10;ADAM10;ortholog	protease(PC00190)
HUMAN|HGNC=12755|UniProtKB=Q9UNX4	WD repeat-containing protein 3;WDR3;ortholog	
HUMAN|HGNC=31418|UniProtKB=Q5VYM1	Uncharacterized protein C9orf131;C9orf131;ortholog	
HUMAN|HGNC=16906|UniProtKB=Q9Y2Q3	Glutathione S-transferase kappa 1;GSTK1;ortholog	transferase(PC00220)
HUMAN|HGNC=26350|UniProtKB=Q7Z5J8	Ankyrin and armadillo repeat-containing protein;ANKAR;ortholog	
HUMAN|HGNC=19985|UniProtKB=P53999	Activated RNA polymerase II transcriptional coactivator p15;SUB1;ortholog	general transcription factor(PC00259)

HUMAN|HGNC=24335|UniProtKB=Q99627	COP9 signalosome complex subunit 8;COPS8;ortholog	scaffold/adaptor protein(PC00226)
HUMAN|HGNC=15999|UniProtKB=Q9NZV5	Selenoprotein N;SELENON;ortholog	
HUMAN|HGNC=5816|UniProtKB=P01624	Immunoglobulin kappa variable 3-15;IGKV3-15;ortholog	immunoglobulin(PC00123)
HUMAN|HGNC=11985|UniProtKB=O94826	Mitochondrial import receptor subunit TOM70;TOMM70;ortholog	primary active transporter(PC00068)
HUMAN|HGNC=5733|UniProtKB=P01599	Immunoglobulin kappa variable 1-17;IGKV1-17;ortholog	immunoglobulin(PC00123)
HUMAN|HGNC=6207|UniProtKB=P14923	Junction plakoglobin;JUP;ortholog	
HUMAN|HGNC=11763|UniProtKB=P02786	Transferrin receptor protein 1;TFRC;ortholog	metalloprotease(PC00153)
HUMAN|HGNC=1165|UniProtKB=Q9Y4D2	Diacylglycerol lipase-alpha;DAGLA;ortholog	lipase(PC00143)
HUMAN|HGNC=18263|UniProtKB=Q9UL25	Ras-related protein Rab-21;RAB21;ortholog	
HUMAN|HGNC=11873|UniProtKB=Q9NYL9	Tropomodulin-3;TMOD3;ortholog	actin or actin-binding cytoskeletal protein(PC00041)
HUMAN|HGNC=7849|UniProtKB=P15531	Nucleoside diphosphate kinase A;NME1;ortholog	kinase(PC00137)
HUMAN|HGNC=11445|UniProtKB=Q15833	Syntaxin-binding protein 2;STXBP2;ortholog	membrane trafficking regulatory protein(PC00151)
HUMAN|HGNC=21245|UniProtKB=Q6UXB8	Peptidase inhibitor 16;PI16;ortholog	defense/immunity protein(PC00090)
HUMAN|HGNC=1735|UniProtKB=Q16543	Hsp90 co-chaperone Cdc37;CDC37;ortholog	chaperone(PC00072)
HUMAN|HGNC=26281|UniProtKB=Q6ZN84	Coiled-coil domain-containing protein 81;CCDC81;ortholog	
HUMAN|HGNC=19880|UniProtKB=Q9Y6C2	EMILIN-1;EMILIN1;ortholog	
HUMAN|HGNC=4719|UniProtKB=P16401	Histone H1.5;H1-5;ortholog	histone(PC00118)
HUMAN|HGNC=5783|UniProtKB=A0A075B6P5	Immunoglobulin kappa variable 2-28;IGKV2-28;ortholog	immunoglobulin(PC00123)
HUMAN|HGNC=4138|UniProtKB=Q14697	Neutral alpha-glucosidase AB;GANAB;ortholog	glucosidase(PC00108)
HUMAN|HGNC=34383|UniProtKB=A6NGG8	Photoreceptor cilium actin regulator;PCARE;ortholog	
HUMAN|HGNC=1095|UniProtKB=P17213	Bactericidal permeability-increasing protein;BPI;ortholog	
HUMAN|HGNC=9352|UniProtKB=Q06830	Peroxiredoxin-1;PRDX1;ortholog	peroxidase(PC00180)
HUMAN|HGNC=10474|UniProtKB=Q9Y265	RuvB-like 1;RUVBL1;ortholog	
HUMAN|HGNC=2231|UniProtKB=P53618	Coatomer subunit beta;COPB1;ortholog	vesicle coat protein(PC00235)
HUMAN|HGNC=9583|UniProtKB=P26599	Polypyrimidine tract-binding protein 1;PTBP1;ortholog	
HUMAN|HGNC=6421|UniProtKB=P19012	Keratin, type I cytoskeletal 15;KRT15;ortholog	

HUMAN|HGNC=347|UniProtKB=Q09666	Neuroblast differentiation-associated protein AHNAK;AHNAK;ortholog	
HUMAN|HGNC=15754|UniProtKB=P24844	Myosin regulatory light polypeptide 9;MYL9;ortholog	actin or actin-binding cytoskeletal protein(PC00041)
HUMAN|HGNC=6984|UniProtKB=P23368	NAD-dependent malic enzyme, mitochondrial;ME2;ortholog	oxidoreductase(PC00176)
HUMAN|HGNC=2328|UniProtKB=P50416	Carnitine O-palmitoyltransferase 1, liver isoform;CPT1A;ortholog	acyltransferase(PC00042)
HUMAN|HGNC=10661|UniProtKB=P31431	Syndecan-4;SDC4;ortholog	transmembrane signal receptor(PC00197)
HUMAN|HGNC=23708|UniProtKB=Q6ZR52	Zinc finger protein 493;ZNF493;ortholog	C2H2 zinc finger transcription factor(PC00248)
HUMAN|HGNC=5480|UniProtKB=P01880	Immunoglobulin heavy constant delta;IGHD;ortholog	immunoglobulin(PC00123)
HUMAN|HGNC=1488|UniProtKB=P52907	F-actin-capping protein subunit alpha-1;CAPZA1;ortholog	non-motor actin binding protein(PC00165)
HUMAN|HGNC=1709|UniProtKB=P21926	CD9 antigen;CD9;ortholog	
HUMAN|HGNC=24748|UniProtKB=Q68D51	DENN domain-containing protein 2C;DENND2C;ortholog	
HUMAN|HGNC=4716|UniProtKB=P16403	Histone H1.2;H1-2;ortholog	histone(PC00118)
HUMAN|HGNC=10377|UniProtKB=P05387	60S acidic ribosomal protein P2;RPLP2;ortholog	ribosomal protein(PC00202)
HUMAN|HGNC=9255|UniProtKB=P23284	Peptidyl-prolyl cis-trans isomerase B;PPIB;ortholog	chaperone(PC00072)
HUMAN|HGNC=6735|UniProtKB=P07948	Tyrosine-protein kinase Lyn;LYN;ortholog	
HUMAN|HGNC=5836|UniProtKB=A0A0C4DH24	Immunoglobulin kappa variable 6-21;IGKV6-21;ortholog	immunoglobulin(PC00123)
HUMAN|HGNC=23432|UniProtKB=Q9NXG0	Centlein;CNTLN;ortholog	
HUMAN|HGNC=3211|UniProtKB=P29692	Elongation factor 1-delta;EEF1D;ortholog	translation elongation factor(PC00222)
HUMAN|HGNC=4376|UniProtKB=P36959	GMP reductase 1;GMPR;ortholog	reductase(PC00198)
HUMAN|HGNC=8948|UniProtKB=P29622	Kallistatin;SERPINA4;ortholog	protease inhibitor(PC00191)
HUMAN|HGNC=1682|UniProtKB=Q08722	Leukocyte surface antigen CD47;CD47;ortholog	cell adhesion molecule(PC00069)
HUMAN|HGNC=2528|UniProtKB=P53634	Dipeptidyl peptidase 1;CTSC;ortholog	cysteine protease(PC00081)
HUMAN|HGNC=8062|UniProtKB=P49790	Nuclear pore complex protein Nup153;NUP153;ortholog	transporter(PC00227)
HUMAN|HGNC=3380|UniProtKB=Q9Y2J2	Band 4.1-like protein 3;EPB41L3;ortholog	
HUMAN|HGNC=23151|UniProtKB=Q86UX7	Fermitin family homolog 3;FERMT3;ortholog	
HUMAN|HGNC=14977|UniProtKB=Q9Y5W7	Sorting nexin-14;SNX14;ortholog	scaffold/adaptor protein(PC00226)
HUMAN|HGNC=9673|UniProtKB=Q12913	Receptor-type tyrosine-protein phosphatase eta;PTPRJ;ortholog	protein phosphatase(PC00195)

HUMAN|HGNC=11510|UniProtKB=Q8N9I0	Synaptotagmin-2;SYT2;ortholog	membrane trafficking regulatory protein(PC00151)
HUMAN|HGNC=25544|UniProtKB=Q9HCI7	E3 ubiquitin-protein ligase MSL2;MSL2;ortholog	
HUMAN|HGNC=19825|UniProtKB=Q6SA08	Testis-specific serine/threonine-protein kinase 4;TSSK4;ortholog	non-receptor serine/threonine protein kinase(PC00167)
HUMAN|HGNC=20369|UniProtKB=Q9H4X1	Regulator of cell cycle RGCC;RGCC;ortholog	
HUMAN|HGNC=18585|UniProtKB=Q96DH6	RNA-binding protein Musashi homolog 2;MSI2;ortholog	
HUMAN|HGNC=9778|UniProtKB=P20337	Ras-related protein Rab-3B;RAB3B;ortholog	
HUMAN|HGNC=2267|UniProtKB=P20674	Cytochrome c oxidase subunit 5A, mitochondrial;COX5A;ortholog	oxidase(PC00175)
HUMAN|HGNC=1472|UniProtKB=P49913	Cathelicidin antimicrobial peptide;CAMP;ortholog	antimicrobial response protein(PC00051)
HUMAN|HGNC=3148|UniProtKB=P19971	Thymidine phosphorylase;TYMP;ortholog	glycosyltransferase(PC00111)
HUMAN|HGNC=29058|UniProtKB=O60268	Uncharacterized protein KIAA0513;KIAA0513;ortholog	
HUMAN|HGNC=7219|UniProtKB=Q00013	55 kDa erythrocyte membrane protein;MPP1;ortholog	scaffold/adaptor protein(PC00226)
HUMAN|HGNC=17259|UniProtKB=Q9Y4Z0	U6 snRNA-associated Sm-like protein LSm4;LSM4;ortholog	RNA splicing factor(PC00148)
HUMAN|HGNC=19986|UniProtKB=P99999	Cytochrome c;CYCS;ortholog	
HUMAN|HGNC=29416|UniProtKB=Q5T7W0	Zinc finger protein 618;ZNF618;ortholog	C2H2 zinc finger transcription factor(PC00248)
HUMAN|HGNC=24356|UniProtKB=Q9P126	C-type lectin domain family 1 member B;CLEC1B;ortholog	
HUMAN|HGNC=20347|UniProtKB=Q9H9C1	Spermatogenesis-defective protein 39 homolog;VIPAS39;ortholog	
HUMAN|HGNC=13487|UniProtKB=Q96QK1	Vacuolar protein sorting-associated protein 35;VPS35;ortholog	membrane traffic protein(PC00150)
HUMAN|HGNC=25571|UniProtKB=Q9NVG8	TBC1 domain family member 13;TBC1D13;ortholog	GTPase-activating protein(PC00257)
HUMAN|HGNC=17801|UniProtKB=Q8NHY6	Zinc finger protein 28 homolog;ZFP28;ortholog	
HUMAN|HGNC=30377|UniProtKB=Q14160	Protein scribble homolog;SCRIB;ortholog	scaffold/adaptor protein(PC00226)
HUMAN|HGNC=4396|UniProtKB=P62873	Guanine nucleotide-binding protein G(I)/G(S)/G(T) subunit beta-1;GNB1;ortholog	heterotrimeric G-protein(PC00117)
HUMAN|HGNC=29190|UniProtKB=Q9UPQ9	Trinucleotide repeat-containing gene 6B protein;TNRC6B;ortholog	RNA metabolism protein(PC00031)
HUMAN|HGNC=11320|UniProtKB=Q8IZP0	Abl interactor 1;ABI1;ortholog	scaffold/adaptor protein(PC00226)
HUMAN|HGNC=16500|UniProtKB=Q9UN19	Dual adapter for phosphotyrosine and 3-phosphotyrosine and 3-phosphoinositide;DAPP1;ortholog	
HUMAN|HGNC=1452|UniProtKB=P27482	Calmodulin-like protein 3;CALML3;ortholog	calmodulin-related(PC00061)

HUMAN|HGNC=8881|UniProtKB=P07737	Profilin-1;PFN1;ortholog	non-motor actin binding protein(PC00165)
HUMAN|HGNC=9391|UniProtKB=P13861	cAMP-dependent protein kinase type II-alpha regulatory subunit;PRKAR2A;ortholog	kinase modulator(PC00140)
HUMAN|HGNC=9361|UniProtKB=P10124	Serglycin;SRGN;ortholog	
HUMAN|HGNC=12458|UniProtKB=P62987	Ubiquitin-60S ribosomal protein L40;UBA52;ortholog	
HUMAN|HGNC=15749|UniProtKB=Q9NP55	BPI fold-containing family A member 1;BPIFA1;ortholog	
HUMAN|HGNC=17905|UniProtKB=Q9Y613	FH1/FH2 domain-containing protein 1;FHOD1;ortholog	
HUMAN|HGNC=14085|UniProtKB=Q9NQC3	Reticulon-4;RTN4;ortholog	
HUMAN|HGNC=12647|UniProtKB=Q9BV40	Vesicle-associated membrane protein 8;VAMP8;ortholog	
HUMAN|HGNC=5386|UniProtKB=P51553	Isocitrate dehydrogenase [NAD] subunit gamma, mitochondrial;IDH3G;ortholog	dehydrogenase(PC00092)
HUMAN|HGNC=31332|UniProtKB=Q6IQ22	Ras-related protein Rab-12;RAB12;ortholog	
HUMAN|HGNC=33895|UniProtKB=Q6S8J3	POTE ankyrin domain family member E;POTEE;ortholog	membrane traffic protein(PC00150)
HUMAN|HGNC=9093|UniProtKB=P55058	Phospholipid transfer protein;PLTP;ortholog	
HUMAN|HGNC=10475|UniProtKB=Q9Y230	RuvB-like 2;RUVBL2;ortholog	
HUMAN|HGNC=23378|UniProtKB=Q5HYC2	Uncharacterized protein KIAA2026;KIAA2026;ortholog	
HUMAN|HGNC=9868|UniProtKB=Q99969	Retinoic acid receptor responder protein 2;RARRES2;ortholog	
HUMAN|HGNC=5586|UniProtKB=A0A0B4J1V1	Immunoglobulin heavy variable 3-21;IGHV3-21;ortholog	immunoglobulin(PC00123)
HUMAN|Gene=HV309_HUMAN|UniProtKB=P01782	Immunoglobulin heavy variable 3-9;IGHV3-9;ortholog	immunoglobulin(PC00123)
HUMAN|HGNC=3579|UniProtKB=P16930	Fumarylacetoacetase;FAH;ortholog	
HUMAN|HGNC=6970|UniProtKB=P40925	Malate dehydrogenase, cytoplasmic;MDH1;ortholog	dehydrogenase(PC00092)
HUMAN|HGNC=23664|UniProtKB=Q5TCZ1	SH3 and PX domain-containing protein 2A;SH3PXD2A;ortholog	
HUMAN|HGNC=2153|UniProtKB=Q9NQW8	Cyclic nucleotide-gated cation channel beta-3;CNGB3;ortholog	ligand-gated ion channel(PC00141)
HUMAN|HGNC=11619|UniProtKB=Q15370	Elongin-B;ELOB;ortholog	general transcription factor(PC00259)
HUMAN|HGNC=1859|UniProtKB=Q9BV73	Centrosome-associated protein CEP250;CEP250;ortholog	chromatin/chromatin-binding, or -regulatory protein(PC00077)
HUMAN|HGNC=9472|UniProtKB=Q99538	Legumain;LGMN;ortholog	cysteine protease(PC00081)
HUMAN|HGNC=618|UniProtKB=O14791	Apolipoprotein L1;APOL1;ortholog	apolipoprotein(PC00052)
HUMAN|HGNC=395|UniProtKB=P13716	Delta-aminolevulinic acid dehydratase;ALAD;ortholog	dehydratase(PC00091)

HUMAN|HGNC=30026|UniProtKB=Q8TD55	Pleckstrin homology domain-containing family O member 2;PLEKHO2;ortholog	
HUMAN|HGNC=11559|UniProtKB=P37837	Transaldolase;TALDO1;ortholog	aldolase(PC00044)
HUMAN|HGNC=24729|UniProtKB=Q96LP6	Uncharacterized protein C12orf42;C12orf42;ortholog	
HUMAN|HGNC=21393|UniProtKB=Q9Y3V2	RWD domain-containing protein 3;RWDD3;ortholog	
HUMAN|HGNC=22931|UniProtKB=Q9P2N7	Kelch-like protein 13;KLHL13;ortholog	
HUMAN|HGNC=830|UniProtKB=P06576	ATP synthase subunit beta, mitochondrial;ATP5F1B;ortholog	ATP synthase(PC00002)
HUMAN|HGNC=29021|UniProtKB=O15078	Centrosomal protein of 290 kDa;CEP290;ortholog	
HUMAN|HGNC=15713|UniProtKB=Q9UJX4	Anaphase-promoting complex subunit 5;ANAPC5;ortholog	
HUMAN|HGNC=3157|UniProtKB=Q92838	Ectodysplasin-A;EDA;ortholog	
HUMAN|HGNC=2389|UniProtKB=P02511	Alpha-crystallin B chain;CRYAB;ortholog	
HUMAN|HGNC=10833|UniProtKB=Q9Y371	Endophilin-B1;SH3GLB1;ortholog	
HUMAN|HGNC=11583|UniProtKB=P05543	Thyroxine-binding globulin;SERPINA7;ortholog	protease inhibitor(PC00191)
HUMAN|HGNC=30796|UniProtKB=Q9Y3F4	Serine-threonine kinase receptor-associated protein;STRAP;ortholog	translation initiation factor(PC00224)
HUMAN|HGNC=23077|UniProtKB=Q96FW1	Ubiquitin thioesterase OTUB1;OTUB1;ortholog	cysteine protease(PC00081)
HUMAN|HGNC=29035|UniProtKB=O43314	Inositol hexakisphosphate and diphosphoinositol-pentakisphosphate kinase 2;PPIP5K2;ortholog	nucleotide kinase(PC00172)
HUMAN|HGNC=1331|UniProtKB=P01031	Complement C5;C5;ortholog	protease inhibitor(PC00191)
HUMAN|HGNC=25361|UniProtKB=Q8N264	Rho GTPase-activating protein 24;ARHGAP24;ortholog	
HUMAN|HGNC=12652|UniProtKB=P50552	Vasodilator-stimulated phosphoprotein;VASP;ortholog	scaffold/adaptor protein(PC00226)
HUMAN|HGNC=1324|UniProtKB=P0C0L5	Complement C4-B;C4B_2;ortholog	protease inhibitor(PC00191)
HUMAN|HGNC=6153|UniProtKB=P05556	Integrin beta-1;ITGB1;ortholog	integrin(PC00126)
HUMAN|HGNC=8868|UniProtKB=Q9NQP4	Prefoldin subunit 4;PFDN4;ortholog	
HUMAN|HGNC=8988|UniProtKB=Q13526	Peptidyl-prolyl cis-trans isomerase NIMA-interacting 1;PIN1;ortholog	chaperone(PC00072)
HUMAN|HGNC=18808|UniProtKB=Q9Y6E2	Basic leucine zipper and W2 domain-containing protein 2;BZW2;ortholog	basic leucine zipper transcription factor(PC00056)
HUMAN|HGNC=1681|UniProtKB=P16070	CD44 antigen;CD44;ortholog	transmembrane signal receptor(PC00197)
HUMAN|HGNC=19368|UniProtKB=Q4G0P3	Hydrocephalus-inducing protein homolog;HYDIN;ortholog	
HUMAN|HGNC=20373|UniProtKB=Q9NZD8	Maspardin;SPG21;ortholog	

HUMAN|HGNC=7373|UniProtKB=P26038	Moesin;MSN;ortholog	actin or actin-binding cytoskeletal protein(PC00041)
HUMAN|HGNC=8728|UniProtKB=P22061	Protein-L-isoaspartate(D-aspartate) O-methyltransferase;PCMT1;ortholog	methyltransferase(PC00155)
HUMAN|HGNC=3530|UniProtKB=P00748	Coagulation factor XII;F12;ortholog	serine protease(PC00203)
HUMAN|HGNC=5662|UniProtKB=A0A0B4J1U7	Immunoglobulin heavy variable 6-1;IGHV6-1;ortholog	immunoglobulin(PC00123)
HUMAN|HGNC=1226|UniProtKB=O14908	PDZ domain-containing protein GIPC1;GIPC1;ortholog	scaffold/adaptor protein(PC00226)
HUMAN|HGNC=14008|UniProtKB=Q9BW11	Max dimerization protein 3;MXD3;ortholog	basic helix-loop-helix transcription factor(PC00055)
HUMAN|HGNC=7640|UniProtKB=Q99733	Nucleosome assembly protein 1-like 4;NAP1L4;ortholog	chromatin/chromatin-binding, or -regulatory protein(PC00077)
HUMAN|HGNC=20040|UniProtKB=Q01518	Adenylyl cyclase-associated protein 1;CAP1;ortholog	actin or actin-binding cytoskeletal protein(PC00041)
HUMAN|HGNC=20296|UniProtKB=Q9Y2H6	Fibronectin type-III domain-containing protein 3A;FNDC3A;ortholog	ubiquitin-protein ligase(PC00234)
HUMAN|HGNC=8888|UniProtKB=P18669	Phosphoglycerate mutase 1;PGAM1;ortholog	mutase(PC00160)
HUMAN|HGNC=6714|UniProtKB=Q14766	Latent-transforming growth factor beta-binding protein 1;LTBP1;ortholog	extracellular matrix structural protein(PC00103)
HUMAN|HGNC=3594|UniProtKB=P49327	Fatty acid synthase;FASN;ortholog	
HUMAN|HGNC=1620|UniProtKB=P40227	T-complex protein 1 subunit zeta;CCT6A;ortholog	chaperonin(PC00073)
HUMAN|HGNC=23643|UniProtKB=Q8IVF6	Ankyrin repeat domain-containing protein 18A;ANKRD18A;ortholog	
HUMAN|HGNC=11554|UniProtKB=P37802	Transgelin-2;TAGLN2;ortholog	non-motor actin binding protein(PC00165)
HUMAN|HGNC=3153|UniProtKB=Q16610	Extracellular matrix protein 1;ECM1;ortholog	
HUMAN|HGNC=10396|UniProtKB=P62249	40S ribosomal protein S16;RPS16;ortholog	ribosomal protein(PC00202)
HUMAN|HGNC=11766|UniProtKB=P01137	Transforming growth factor beta-1 proprotein;TGFB1;ortholog	growth factor(PC00112)
HUMAN|HGNC=5652|UniProtKB=A0A075B6R2	Immunoglobulin heavy variable 4-4;IGHV4-4;ortholog	immunoglobulin(PC00123)
HUMAN|HGNC=5269|UniProtKB=P61604	10 kDa heat shock protein, mitochondrial;HSPE1;ortholog	chaperonin(PC00073)
HUMAN|HGNC=10353|UniProtKB=P36578	60S ribosomal protein L4;RPL4;ortholog	ribosomal protein(PC00202)
HUMAN|HGNC=33108|UniProtKB=Q6NX45	Zinc finger protein 774;ZNF774;ortholog	
HUMAN|HGNC=29327|UniProtKB=Q5VZ46	Uncharacterized protein KIAA1614;KIAA1614;ortholog	tight junction(PC00214)
HUMAN|HGNC=9406|UniProtKB=Q16513	Serine/threonine-protein kinase N2;PKN2;ortholog	non-receptor serine/threonine protein kinase(PC00167)

HUMAN|HGNC=2292|UniProtKB=P15954	Cytochrome c oxidase subunit 7C, mitochondrial;COX7C;ortholog	oxidase(PC00175)
HUMAN|HGNC=31073|UniProtKB=Q9Y2K3	Myosin-15;MYH15;ortholog	
HUMAN|HGNC=20508|UniProtKB=Q8IUE6	Histone H2A type 2-B;H2AC21;ortholog	histone(PC00118)
HUMAN|HGNC=15568|UniProtKB=Q9H299	SH3 domain-binding glutamic acid-rich-like protein 3;SH3BGRL3;ortholog	
HUMAN|HGNC=4563|UniProtKB=O75791	GRB2-related adapter protein 2;GRAP2;ortholog	
HUMAN|HGNC=2047|UniProtKB=O00501	Claudin-5;CLDN5;ortholog	tight junction(PC00214)
HUMAN|HGNC=17060|UniProtKB=Q92845	Kinesin-associated protein 3;KIFAP3;ortholog	microtubule binding motor protein(PC00156)
HUMAN|HGNC=16452|UniProtKB=P0DN86	Choriogonadotropin subunit beta 3;CGB8;ortholog	peptide hormone(PC00179)
HUMAN|HGNC=28338|UniProtKB=Q6ZUT9	DENN domain-containing protein 5B;DENND5B;ortholog	
HUMAN|HGNC=910|UniProtKB=P25311	Zinc-alpha-2-glycoprotein;AZGP1;ortholog	major histocompatibility complex protein(PC00149)
HUMAN|HGNC=16108|UniProtKB=Q8TDL5	BPI fold-containing family B member 1;BPIFB1;ortholog	
HUMAN|HGNC=2422|UniProtKB=O75390	Citrate synthase, mitochondrial;CS;ortholog	transferase(PC00220)
HUMAN|HGNC=1245|UniProtKB=P02747	Complement C1q subcomponent subunit C;C1QC;ortholog	
HUMAN|HGNC=23526|UniProtKB=Q8NE86	Calcium uniporter protein, mitochondrial;MCU;ortholog	
HUMAN|HGNC=24056|UniProtKB=Q8NHP1	Aflatoxin B1 aldehyde reductase member 4;AKR7L;ortholog	reductase(PC00198)
HUMAN|HGNC=782|UniProtKB=O95260	Arginyl-tRNA--protein transferase 1;ATE1;ortholog	translational protein(PC00263)
HUMAN|HGNC=7850|UniProtKB=P22392	Nucleoside diphosphate kinase B;NME2;ortholog	kinase(PC00137)
HUMAN|HGNC=4037|UniProtKB=P06241	Tyrosine-protein kinase Fyn;FYN;ortholog	
HUMAN|HGNC=9530|UniProtKB=P25786	Proteasome subunit alpha type-1;PSMA1;ortholog	protease(PC00190)
HUMAN|HGNC=5381|UniProtKB=P14735	Insulin-degrading enzyme;IDE;ortholog	metalloprotease(PC00153)
HUMAN|HGNC=24811|UniProtKB=Q14554	Protein disulfide-isomerase A5;PDIA5;ortholog	chaperone(PC00072)
HUMAN|HGNC=5887|UniProtKB=P01706	Immunoglobulin lambda variable 2-11;IGLV2-11;ortholog	immunoglobulin(PC00123)
HUMAN|HGNC=1745|UniProtKB=O00311	Cell division cycle 7-related protein kinase;CDC7;ortholog	non-receptor serine/threonine protein kinase(PC00167)
HUMAN|HGNC=15588|UniProtKB=Q96C24	Synaptotagmin-like protein 4;SYTL4;ortholog	
HUMAN|HGNC=5043|UniProtKB=P31942	Heterogeneous nuclear ribonucleoprotein H3;HNRNPH3;ortholog	RNA splicing factor(PC00148)
HUMAN|HGNC=1459|UniProtKB=Q14012	Calcium/calmodulin-dependent protein kinase type 1;CAMK1;ortholog	non-receptor serine/threonine protein kinase(PC00167)

HUMAN|HGNC=2362|UniProtKB=P46108	Adapter molecule crk;CRK;ortholog	scaffold/adaptor protein(PC00226)
HUMAN|HGNC=7734|UniProtKB=P07197	Neurofilament medium polypeptide;NEFM;ortholog	
HUMAN|HGNC=15935|UniProtKB=Q9H4I2	Zinc fingers and homeoboxes protein 3;ZHX3;ortholog	homeodomain transcription factor(PC00119)
HUMAN|HGNC=1365|UniProtKB=Q9UGQ2	Calcium channel flower homolog;CACFD1;ortholog	
HUMAN|HGNC=16831|UniProtKB=O95153	Peripheral-type benzodiazepine receptor-associated protein 1;TSPOAP1;ortholog	
HUMAN|HGNC=5619|UniProtKB=A0A0C4DH42	Immunoglobulin heavy variable 3-66;IGHV3-66;ortholog	immunoglobulin(PC00123)
HUMAN|HGNC=3757|UniProtKB=O75955	Flotillin-1;FLOT1;ortholog	
HUMAN|HGNC=775|UniProtKB=P01008	Antithrombin-III;SERPINC1;ortholog	protease inhibitor(PC00191)
HUMAN|HGNC=800|UniProtKB=P50993	Sodium/potassium-transporting ATPase subunit alpha-2;ATP1A2;ortholog	primary active transporter(PC00068)
HUMAN|HGNC=612|UniProtKB=P05090	Apolipoprotein D;APOD;ortholog	apolipoprotein(PC00052)
HUMAN|HGNC=655|UniProtKB=P18085	ADP-ribosylation factor 4;ARF4;ortholog	G-protein(PC00020)
HUMAN|HGNC=25364|UniProtKB=Q9H0R4	Haloacid dehalogenase-like hydrolase domain-containing protein 2;HDHD2;ortholog	phosphatase(PC00181)
HUMAN|HGNC=6513|UniProtKB=Q14847	LIM and SH3 domain protein 1;LASP1;ortholog	
HUMAN|HGNC=4384|UniProtKB=P63096	Guanine nucleotide-binding protein G(i) subunit alpha-1;GNAI1;ortholog	heterotrimeric G-protein(PC00117)
HUMAN|HGNC=11834|UniProtKB=P29401	Transketolase;TKT;ortholog	transketolase(PC00221)
HUMAN|HGNC=4116|UniProtKB=Q14376	UDP-glucose 4-epimerase;GALE;ortholog	epimerase/racemase(PC00096)
HUMAN|HGNC=14906|UniProtKB=Q8N4C6	Ninein;NIN;ortholog	
HUMAN|HGNC=6047|UniProtKB=Q16891	MICOS complex subunit MIC60;IMMT;ortholog	
HUMAN|HGNC=25564|UniProtKB=Q9NVJ2	ADP-ribosylation factor-like protein 8B;ARL8B;ortholog	
HUMAN|HGNC=18396|UniProtKB=O95969	Secretoglobin family 1D member 2;SCGB1D2;ortholog	transfer/carrier protein(PC00219)
HUMAN|HGNC=1546|UniProtKB=P50454	Serpin H1;SERPINH1;ortholog	protease inhibitor(PC00191)
HUMAN|HGNC=11387|UniProtKB=P31948	Stress-induced-phosphoprotein 1;STIP1;ortholog	
HUMAN|HGNC=8905|UniProtKB=P36871	Phosphoglucomutase-1;PGM1;ortholog	mutase(PC00160)
HUMAN|HGNC=5270|UniProtKB=P25685	DnaJ homolog subfamily B member 1;DNAJB1;ortholog	chaperone(PC00072)
HUMAN|HGNC=5383|UniProtKB=P48735	Isocitrate dehydrogenase [NADP], mitochondrial;IDH2;ortholog	dehydrogenase(PC00092)
HUMAN|HGNC=20675|UniProtKB=Q96KN2	Beta-Ala-His dipeptidase;CNDP1;ortholog	metalloprotease(PC00153)

HUMAN|HGNC=9552|UniProtKB=P62195	26S proteasome regulatory subunit 8;PSMC5;ortholog	protease(PC00190)
HUMAN|HGNC=11180|UniProtKB=P04179	Superoxide dismutase [Mn], mitochondrial;SOD2;ortholog	oxidoreductase(PC00176)
HUMAN|HGNC=17717|UniProtKB=Q9UEW8	STE20/SPS1-related proline-alanine-rich protein kinase;STK39;ortholog	
HUMAN|HGNC=5888|UniProtKB=P01704	Immunoglobulin lambda variable 2-14;IGLV2-14;ortholog	immunoglobulin(PC00123)
HUMAN|HGNC=11609|UniProtKB=P24557	Thromboxane-A synthase;TBXAS1;ortholog	isomerase(PC00135)
HUMAN|HGNC=18002|UniProtKB=Q9NS69	Mitochondrial import receptor subunit TOM22 homolog;TOMM22;ortholog	primary active transporter(PC00068)
HUMAN|HGNC=12405|UniProtKB=P02766	Transthyretin;TTR;ortholog	hydrolase(PC00121)
HUMAN|HGNC=9846|UniProtKB=P62826	GTP-binding nuclear protein Ran;RAN;ortholog	small GTPase(PC00208)
HUMAN|HGNC=3658|UniProtKB=Q9UGM5	Fetuin-B;FETUB;ortholog	protease inhibitor(PC00191)
HUMAN|HGNC=11582|UniProtKB=Q15813	Tubulin-specific chaperone E;TBCE;ortholog	chaperone(PC00072)
HUMAN|HGNC=9855|UniProtKB=P62834	Ras-related protein Rap-1A;RAP1A;ortholog	small GTPase(PC00208)
HUMAN|HGNC=812|UniProtKB=P16615	Sarcoplasmic/endoplasmic reticulum calcium ATPase 2;ATP2A2;ortholog	primary active transporter(PC00068)
HUMAN|HGNC=11131|UniProtKB=O00161	Synaptosomal-associated protein 23;SNAP23;ortholog	SNARE protein(PC00034)
HUMAN|HGNC=9621|UniProtKB=Q6IBS0	Twinfilin-2;TWF2;ortholog	non-motor actin binding protein(PC00165)
HUMAN|HGNC=659|UniProtKB=P62330	ADP-ribosylation factor 6;ARF6;ortholog	G-protein(PC00020)
HUMAN|HGNC=2717|UniProtKB=Q16531	DNA damage-binding protein 1;DDB1;ortholog	RNA processing factor(PC00147)
HUMAN|HGNC=10839|UniProtKB=P04278	Sex hormone-binding globulin;SHBG;ortholog	
HUMAN|HGNC=361|UniProtKB=P00568	Adenylate kinase isoenzyme 1;AK1;ortholog	nucleotide kinase(PC00172)
HUMAN|HGNC=26224|UniProtKB=Q9H5Z6	Protein FAM124B;FAM124B;ortholog	
HUMAN|HGNC=2464|UniProtKB=P13611	Versican core protein;VCAN;ortholog	extracellular matrix glycoprotein(PC00100)
HUMAN|HGNC=10055|UniProtKB=Q8N5U6	RING finger protein 10;RNF10;ortholog	
HUMAN|HGNC=5930|UniProtKB=A0A075B6I9	Immunoglobulin lambda variable 7-46;IGLV7-46;ortholog	immunoglobulin(PC00123)
HUMAN|HGNC=8016|UniProtKB=P46459	Vesicle-fusing ATPase;NSF;ortholog	membrane traffic protein(PC00150)
HUMAN|HGNC=26022|UniProtKB=Q7L0Y3	tRNA methyltransferase 10 homolog C;TRMT10C;ortholog	RNA methyltransferase(PC00033)
HUMAN|HGNC=1325|UniProtKB=P04003	C4b-binding protein alpha chain;C4BPA;ortholog	
HUMAN|HGNC=26200|UniProtKB=Q9H668	CST complex subunit STN1;STN1;ortholog	

HUMAN|HGNC=9944|UniProtKB=P35241	Radixin;RDX;ortholog	actin or actin-binding cytoskeletal protein(PC00041)
HUMAN|HGNC=2431|UniProtKB=P55060	Exportin-2;CSE1L;ortholog	transporter(PC00227)
HUMAN|HGNC=2090|UniProtKB=P09496	Clathrin light chain A;CLTA;ortholog	vesicle coat protein(PC00235)
HUMAN|HGNC=13572|UniProtKB=Q9Y6R7	IgGFc-binding protein;FCGBP;ortholog	extracellular matrix protein(PC00102)
HUMAN|HGNC=10469|UniProtKB=O95197	Reticulon-3;RTN3;ortholog	
HUMAN|HGNC=3600|UniProtKB=P23142	Fibulin-1;FBLN1;ortholog	
HUMAN|HGNC=3353|UniProtKB=P09104	Gamma-enolase;ENO2;ortholog	lyase(PC00144)
HUMAN|HGNC=12028|UniProtKB=P14625	Endoplasmin;HSP90B1;ortholog	Hsp90 family chaperone(PC00028)
HUMAN|HGNC=29585|UniProtKB=Q8N6V9	Testis-expressed protein 9;TEX9;ortholog	actin binding motor protein(PC00040)
HUMAN|HGNC=2265|UniProtKB=P13073	Cytochrome c oxidase subunit 4 isoform 1, mitochondrial;COX4I1;ortholog	oxidase(PC00175)
HUMAN|HGNC=26161|UniProtKB=P57737	Coronin-7;CORO7;ortholog	non-motor actin binding protein(PC00165)
HUMAN|HGNC=21314|UniProtKB=Q8WXC6	COP9 signalosome complex subunit 9;COPS9;ortholog	
HUMAN|HGNC=9055|UniProtKB=Q00722	1-phosphatidylinositol 4,5-bisphosphate phosphodiesterase beta-2;PLCB2;ortholog	phospholipase(PC00186)
HUMAN|HGNC=19071|UniProtKB=Q86V81	THO complex subunit 4;ALYREF;ortholog	RNA metabolism protein(PC00031)
HUMAN|HGNC=16476|UniProtKB=Q9H0E2	Toll-interacting protein;TOLLIP;ortholog	
HUMAN|HGNC=8912|UniProtKB=P35232	Prohibitin;PHB;ortholog	
HUMAN|HGNC=1473|UniProtKB=P27824	Calnexin;CANX;ortholog	chaperone(PC00072)
HUMAN|HGNC=1063|UniProtKB=P30043	Flavin reductase (NADPH);BLVRB;ortholog	reductase(PC00198)
HUMAN|HGNC=554|UniProtKB=Q10567	AP-1 complex subunit beta-1;AP1B1;ortholog	membrane traffic protein(PC00150)
HUMAN|HGNC=6526|UniProtKB=P80188	Neutrophil gelatinase-associated lipocalin;LCN2;ortholog	transfer/carrier protein(PC00219)
HUMAN|HGNC=833|UniProtKB=P36542	ATP synthase subunit gamma, mitochondrial;ATP5F1C;ortholog	ATP synthase(PC00002)
HUMAN|HGNC=3624|UniProtKB=Q15485	Ficolin-2;FCN2;ortholog	intercellular signal molecule(PC00207)
HUMAN|HGNC=5606|UniProtKB=P01763	Immunoglobulin heavy variable 3-48;IGHV3-48;ortholog	immunoglobulin(PC00123)
HUMAN|HGNC=14569|UniProtKB=Q9UK76	Jupiter microtubule associated homolog 1;JPT1;ortholog	
HUMAN|HGNC=10701|UniProtKB=Q15436	Protein transport protein Sec23A;SEC23A;ortholog	vesicle coat protein(PC00235)
HUMAN|HGNC=12644|UniProtKB=Q15836	Vesicle-associated membrane protein 3;VAMP3;ortholog	

HUMAN|HGNC=11168|UniProtKB=Q13884	Beta-1-syntrophin;SNTB1;ortholog	
HUMAN|HGNC=7716|UniProtKB=P49821	NADH dehydrogenase [ubiquinone] flavoprotein 1, mitochondrial;NDUFV1;ortholog	oxidoreductase(PC00176)
HUMAN|HGNC=5905|UniProtKB=P80748	Immunoglobulin lambda variable 3-21;IGLV3-21;ortholog	immunoglobulin(PC00123)
HUMAN|HGNC=4298|UniProtKB=P16278	Beta-galactosidase;GLB1;ortholog	galactosidase(PC00104)
HUMAN|HGNC=16969|UniProtKB=Q92598	Heat shock protein 105 kDa;HSPH1;ortholog	
HUMAN|HGNC=12724|UniProtKB=P04004	Vitronectin;VTN;ortholog	
HUMAN|HGNC=253|UniProtKB=P11766	Alcohol dehydrogenase class-3;ADH5;ortholog	dehydrogenase(PC00092)
HUMAN|HGNC=2973|UniProtKB=O00429	Dynamin-1-like protein;DNM1L;ortholog	membrane traffic protein(PC00150)
HUMAN|HGNC=170|UniProtKB=P61158	Actin-related protein 3;ACTR3;ortholog	actin and actin related protein(PC00039)
HUMAN|HGNC=12463|UniProtKB=P0CG47	Polyubiquitin-B;UBB;ortholog	
HUMAN|HGNC=30866|UniProtKB=Q8IX04	Ubiquitin-conjugating enzyme E2 variant 3;UEVLD;ortholog	ubiquitin-protein ligase(PC00234)
HUMAN|HGNC=4844|UniProtKB=P14317	Hematopoietic lineage cell-specific protein;HCLS1;ortholog	non-motor actin binding protein(PC00165)
HUMAN|HGNC=9922|UniProtKB=P02753	Retinol-binding protein 4;RBP4;ortholog	transfer/carrier protein(PC00219)
HUMAN|HGNC=3616|UniProtKB=P12318	Low affinity immunoglobulin gamma Fc region receptor II-a;FCGR2A;ortholog	immunoglobulin receptor superfamily(PC00124)
HUMAN|HGNC=11579|UniProtKB=O75347	Tubulin-specific chaperone A;TBCA;ortholog	chaperonin(PC00073)
HUMAN|HGNC=30389|UniProtKB=Q96A65	Exocyst complex component 4;EXOC4;ortholog	membrane traffic protein(PC00150)
HUMAN|Ensembl=ENSG00000282639|UniProtKB=A0A0J9YX35	Immunoglobulin heavy variable 3-64D;IGHV3-64D;ortholog	immunoglobulin(PC00123)
HUMAN|HGNC=26195|UniProtKB=Q86XH1	Dynein regulatory complex protein 11;IQCA1;ortholog	
HUMAN|HGNC=20120|UniProtKB=Q17RC7	Exocyst complex component 3-like protein 4;EXOC3L4;ortholog	membrane traffic protein(PC00150)
HUMAN|HGNC=10372|UniProtKB=P05386	60S acidic ribosomal protein P1;RPLP1;ortholog	ribosomal protein(PC00202)
HUMAN|HGNC=28235|UniProtKB=Q96IU4	Protein ABHD14B;ABHD14B;ortholog	
HUMAN|HGNC=19267|UniProtKB=Q8NFP4	MAM domain-containing glycosylphosphatidylinositol anchor protein 1;MDGA1;ortholog	
HUMAN|HGNC=9535|UniProtKB=P60900	Proteasome subunit alpha type-6;PSMA6;ortholog	protease(PC00190)
HUMAN|HGNC=1742|UniProtKB=P50851	Lipopolysaccharide-responsive and beige-like anchor protein;LRBA;ortholog	
HUMAN|HGNC=19194|UniProtKB=Q96RW7	Hemicentin-1;HMCN1;ortholog	
HUMAN|HGNC=380|UniProtKB=P14550	Aldo-keto reductase family 1 member A1;AKR1A1;ortholog	reductase(PC00198)

HUMAN|HGNC=9069|UniProtKB=Q15149	Plectin;PLEC;ortholog	intermediate filament binding protein(PC00130)
HUMAN|HGNC=6876|UniProtKB=Q16539	Mitogen-activated protein kinase 14;MAPK14;ortholog	non-receptor serine/threonine protein kinase(PC00167)
HUMAN|HGNC=30384|UniProtKB=Q9UMX5	Neudesin;NENF;ortholog	transmembrane signal receptor(PC00197)
HUMAN|HGNC=15679|UniProtKB=Q8WZ74	Cortactin-binding protein 2;CTTNBP2;ortholog	
HUMAN|HGNC=20774|UniProtKB=P04350	Tubulin beta-4A chain;TUBB4A;ortholog	tubulin(PC00228)
HUMAN|HGNC=14237|UniProtKB=Q96NT3	Protein GUCD1;GUCD1;ortholog	guanylate cyclase(PC00114)
HUMAN|HGNC=14511|UniProtKB=P82663	28S ribosomal protein S25, mitochondrial;MRPS25;ortholog	ribosomal protein(PC00202)
HUMAN|HGNC=14545|UniProtKB=Q8IV61	Ras guanyl-releasing protein 3;RASGRP3;ortholog	guanyl-nucleotide exchange factor(PC00113)
HUMAN|HGNC=14362|UniProtKB=Q7Z5P9	Mucin-19;MUC19;ortholog	
HUMAN|HGNC=18807|UniProtKB=O94983	Calmodulin-binding transcription activator 2;CAMTA2;ortholog	DNA-binding transcription factor(PC00218)
HUMAN|HGNC=11231|UniProtKB=Q8WXF7	Atlastin-1;ATL1;ortholog	heterotrimeric G-protein(PC00117)
HUMAN|HGNC=12476|UniProtKB=P61077	Ubiquitin-conjugating enzyme E2 D3;UBE2D3;ortholog	ubiquitin-protein ligase(PC00234)
HUMAN|HGNC=6142|UniProtKB=P23229	Integrin alpha-6;ITGA6;ortholog	integrin(PC00126)
HUMAN|HGNC=20356|UniProtKB=Q9BXV9	EKC/KEOPS complex subunit GON7;GON7;ortholog	
HUMAN|HGNC=16937|UniProtKB=O75915	PRA1 family protein 3;ARL6IP5;ortholog	amino acid transporter(PC00046)
HUMAN|HGNC=26378|UniProtKB=Q8N7C3	Probable E3 ubiquitin-protein ligase TRIML2;TRIML2;ortholog	ubiquitin-protein ligase(PC00234)
HUMAN|HGNC=6149|UniProtKB=P11215	Integrin alpha-M;ITGAM;ortholog	integrin(PC00126)
HUMAN|HGNC=13520|UniProtKB=Q14696	LRP chaperone MESD;MESD;ortholog	
HUMAN|HGNC=3265|UniProtKB=P05198	Eukaryotic translation initiation factor 2 subunit 1;EIF2S1;ortholog	translation initiation factor(PC00224)
HUMAN|HGNC=19388|UniProtKB=P49593	Protein phosphatase 1F;PPM1F;ortholog	protein phosphatase(PC00195)
HUMAN|HGNC=3297|UniProtKB=P78344	Eukaryotic translation initiation factor 4 gamma 2;EIF4G2;ortholog	translation initiation factor(PC00224)
HUMAN|HGNC=5657|UniProtKB=P0DP08	Immunoglobulin heavy variable 4-38-2;IGHV4-38-2;ortholog	immunoglobulin(PC00123)
HUMAN|HGNC=1615|UniProtKB=P78371	T-complex protein 1 subunit beta;CCT2;ortholog	chaperonin(PC00073)
HUMAN|HGNC=5553|UniProtKB=A0A0A0MS14	Immunoglobulin heavy variable 1-45;IGHV1-45;ortholog	immunoglobulin(PC00123)
HUMAN|HGNC=20766|UniProtKB=Q71U36	Tubulin alpha-1A chain;TUBA1A;ortholog	tubulin(PC00228)
HUMAN|HGNC=226|UniProtKB=P78563	Double-stranded RNA-specific editase 1;ADARB1;ortholog	RNA metabolism protein(PC00031)

HUMAN|HGNC=12590|UniProtKB=P07919	Cytochrome b-c1 complex subunit 6, mitochondrial;UQCRH;ortholog	reductase(PC00198)
HUMAN|HGNC=8903|UniProtKB=O95336	6-phosphogluconolactonase;PGLS;ortholog	hydrolase(PC00121)
HUMAN|HGNC=9788|UniProtKB=P51149	Ras-related protein Rab-7a;RAB7A;ortholog	
HUMAN|HGNC=20434|UniProtKB=Q86YW5	Trem-like transcript 1 protein;TREML1;ortholog	immunoglobulin receptor superfamily(PC00124)
HUMAN|HGNC=9554|UniProtKB=Q99460	26S proteasome non-ATPase regulatory subunit 1;PSMD1;ortholog	protease(PC00190)
HUMAN|HGNC=3755|UniProtKB=O75369	Filamin-B;FLNB;ortholog	
HUMAN|HGNC=55|UniProtKB=O15439	Multidrug resistance-associated protein 4;ABCC4;ortholog	ATP-binding cassette (ABC) transporter(PC00003)
HUMAN|HGNC=5861|UniProtKB=A0M8Q6	Immunoglobulin lambda constant 7;IGLC7;ortholog	immunoglobulin(PC00123)
HUMAN|HGNC=24878|UniProtKB=Q9BVK6	Transmembrane emp24 domain-containing protein 9;TMED9;ortholog	vesicle coat protein(PC00235)
HUMAN|HGNC=5528|UniProtKB=P01861	Immunoglobulin heavy constant gamma 4;IGHG4;ortholog	immunoglobulin(PC00123)
HUMAN|HGNC=5588|UniProtKB=P01764	Immunoglobulin heavy variable 3-23;IGHV3-23;ortholog	immunoglobulin(PC00123)
HUMAN|HGNC=26639|UniProtKB=Q8NBI6	Xyloside xylosyltransferase 1;XXYLT1;ortholog	transferase(PC00220)
HUMAN|HGNC=25346|UniProtKB=Q5RHP9	Glutamate-rich protein 3;ERICH3;ortholog	
HUMAN|HGNC=2690|UniProtKB=P07108	Acyl-CoA-binding protein;DBI;ortholog	transfer/carrier protein(PC00219)
HUMAN|HGNC=23845|UniProtKB=Q8IY92	Structure-specific endonuclease subunit SLX4;SLX4;ortholog	
HUMAN|HGNC=82|UniProtKB=P09110	3-ketoacyl-CoA thiolase, peroxisomal;ACAA1;ortholog	acetyltransferase(PC00038)
HUMAN|HGNC=23212|UniProtKB=Q7Z406	Myosin-14;MYH14;ortholog	
HUMAN|HGNC=5659|UniProtKB=A0A0C4DH38	Immunoglobulin heavy variable 5-51;IGHV5-51;ortholog	immunoglobulin(PC00123)
HUMAN|HGNC=2313|UniProtKB=P22792	Carboxypeptidase N subunit 2;CPN2;ortholog	transmembrane signal receptor(PC00197)
HUMAN|HGNC=118|UniProtKB=Q99798	Aconitate hydratase, mitochondrial;ACO2;ortholog	hydratase(PC00120)
HUMAN|HGNC=5825|UniProtKB=A0A0C4DH25	Immunoglobulin kappa variable 3D-20;IGKV3D-20;ortholog	immunoglobulin(PC00123)
HUMAN|HGNC=5525|UniProtKB=P01857	Immunoglobulin heavy constant gamma 1;IGHG1;ortholog	immunoglobulin(PC00123)
HUMAN|HGNC=25087|UniProtKB=Q8IWE2	Protein NOXP20;FAM114A1;ortholog	
HUMAN|HGNC=29284|UniProtKB=Q9P265	Disco-interacting protein 2 homolog B;DIP2B;ortholog	
HUMAN|HGNC=2850|UniProtKB=Q9Y6T7	Diacylglycerol kinase beta;DGKB;ortholog	kinase(PC00137)
HUMAN|HGNC=6737|UniProtKB=O75608	Acyl-protein thioesterase 1;LYPLA1;ortholog	phospholipase(PC00186)
HUMAN|HGNC=9666|UniProtKB=P08575	Receptor-type tyrosine-protein phosphatase C;PTPRC;ortholog	protein phosphatase(PC00195)

HUMAN|HGNC=6890|UniProtKB=Q15691	Microtubule-associated protein RP/EB family member 1;MAPRE1;ortholog	non-motor microtubule binding protein(PC00166)
HUMAN|HGNC=1346|UniProtKB=P10643	Complement component C7;C7;ortholog	
HUMAN|HGNC=25490|UniProtKB=Q96HA8	Protein N-terminal glutamine amidohydrolase;NTAQ1;ortholog	
HUMAN|HGNC=4244|UniProtKB=O00451	GDNF family receptor alpha-2;GFRA2;ortholog	transmembrane signal receptor(PC00197)
HUMAN|HGNC=15722|UniProtKB=Q8WXQ8	Carboxypeptidase A5;CPA5;ortholog	metalloprotease(PC00153)
HUMAN|HGNC=5563|UniProtKB=A0A075B7D0	Ig-like domain-containing protein;IGHV1OR15-1;ortholog	immunoglobulin(PC00123)
HUMAN|HGNC=10840|UniProtKB=P29353	SHC-transforming protein 1;SHC1;ortholog	scaffold/adaptor protein(PC00226)
HUMAN|HGNC=27302|UniProtKB=Q5T440	Putative transferase CAF17, mitochondrial;IBA57;ortholog	
HUMAN|HGNC=5141|UniProtKB=P00738	Haptoglobin;HP;ortholog	serine protease(PC00203)
HUMAN|HGNC=9349|UniProtKB=Q9NQX1	PR domain zinc finger protein 5;PRDM5;ortholog	C2H2 zinc finger transcription factor(PC00248)
HUMAN|HGNC=2711|UniProtKB=Q14203	Dynactin subunit 1;DCTN1;ortholog	chaperone(PC00072)
HUMAN|HGNC=10404|UniProtKB=P15880	40S ribosomal protein S2;RPS2;ortholog	ribosomal protein(PC00202)
HUMAN|HGNC=3011|UniProtKB=Q07507	Dermatopontin;DPT;ortholog	cell adhesion molecule(PC00069)
HUMAN|HGNC=16053|UniProtKB=Q8N1V2	Cilia- and flagella-associated protein 52;CFAP52;ortholog	
HUMAN|HGNC=23818|UniProtKB=Q14684	Ribosomal RNA processing protein 1 homolog B;RRP1B;ortholog	
HUMAN|HGNC=2482|UniProtKB=P04080	Cystatin-B;CSTB;ortholog	protease inhibitor(PC00191)
HUMAN|HGNC=813|UniProtKB=Q93084	Sarcoplasmic/endoplasmic reticulum calcium ATPase 3;ATP2A3;ortholog	primary active transporter(PC00068)
HUMAN|HGNC=9813|UniProtKB=P54727	UV excision repair protein RAD23 homolog B;RAD23B;ortholog	damaged DNA-binding protein(PC00086)
HUMAN|HGNC=117|UniProtKB=P21399	Cytoplasmic aconitate hydratase;ACO1;ortholog	RNA metabolism protein(PC00031)
HUMAN|HGNC=8906|UniProtKB=Q96G03	Phosphoglucomutase-2;PGM2;ortholog	mutase(PC00160)
HUMAN|HGNC=463|UniProtKB=Q9UKV5	E3 ubiquitin-protein ligase AMFR;AMFR;ortholog	ubiquitin-protein ligase(PC00234)
HUMAN|HGNC=13061|UniProtKB=Q14587	Zinc finger protein 268;ZNF268;ortholog	C2H2 zinc finger transcription factor(PC00248)
HUMAN|HGNC=7687|UniProtKB=O00483	Cytochrome c oxidase subunit NDUFA4;NDUFA4;ortholog	oxidoreductase(PC00176)
HUMAN|HGNC=15532|UniProtKB=Q9BX67	Junctional adhesion molecule C;JAM3;ortholog	
HUMAN|HGNC=30594|UniProtKB=Q9H3U1	Protein unc-45 homolog A;UNC45A;ortholog	
HUMAN|HGNC=9761|UniProtKB=Q15907	Ras-related protein Rab-11B;RAB11B;ortholog	

HUMAN|HGNC=5244|UniProtKB=P38646	Stress-70 protein, mitochondrial;HSPA9;ortholog	
HUMAN|HGNC=4851|UniProtKB=P42858	Huntingtin;HTT;ortholog	
HUMAN|HGNC=11869|UniProtKB=Q9UHN6	Cell surface hyaluronidase;CEMIP2;ortholog	
HUMAN|HGNC=7515|UniProtKB=P98088	Mucin-5AC;MUC5AC;ortholog	extracellular matrix protein(PC00102)
HUMAN|HGNC=7198|UniProtKB=Q86VD1	MORC family CW-type zinc finger protein 1;MORC1;ortholog	
HUMAN|HGNC=1354|UniProtKB=P07360	Complement component C8 gamma chain;C8G;ortholog	
HUMAN|HGNC=11740|UniProtKB=P02787	Serotransferrin;TF;ortholog	transfer/carrier protein(PC00219)
HUMAN|HGNC=6971|UniProtKB=P40926	Malate dehydrogenase, mitochondrial;MDH2;ortholog	dehydrogenase(PC00092)
HUMAN|HGNC=34489|UniProtKB=A8K5M9	Uncharacterized protein C15orf62, mitochondrial;C15orf62;ortholog	
HUMAN|HGNC=30004|UniProtKB=P0DMN0	Sulfotransferase 1A4;SULT1A4;ortholog	transferase(PC00220)
HUMAN|HGNC=10992|UniProtKB=P12236	ADP/ATP translocase 3;SLC25A6;ortholog	transfer/carrier protein(PC00219)
HUMAN|HGNC=17474|UniProtKB=Q96AP7	Endothelial cell-selective adhesion molecule;ESAM;ortholog	
HUMAN|HGNC=4762|UniProtKB=P57053	Histone H2B type F-S;H2BS1;ortholog	histone(PC00118)
HUMAN|HGNC=17894|UniProtKB=Q14966	Zinc finger protein 638;ZNF638;ortholog	
HUMAN|HGNC=12691|UniProtKB=P15311	Ezrin;EZR;ortholog	actin or actin-binding cytoskeletal protein(PC00041)
HUMAN|HGNC=3354|UniProtKB=P13929	Beta-enolase;ENO3;ortholog	lyase(PC00144)
HUMAN|HGNC=7568|UniProtKB=P35580	Myosin-10;MYH10;ortholog	
HUMAN|HGNC=16804|UniProtKB=O00308	NEDD4-like E3 ubiquitin-protein ligase WWP2;WWP2;ortholog	ubiquitin-protein ligase(PC00234)
HUMAN|HGNC=10494|UniProtKB=P26447	Protein S100-A4;S100A4;ortholog	calmodulin-related(PC00061)
HUMAN|HGNC=19747|UniProtKB=Q86UK5	Limbin;EVC2;ortholog	
HUMAN|HGNC=8766|UniProtKB=Q8WUM4	Programmed cell death 6-interacting protein;PDCD6IP;ortholog	membrane traffic protein(PC00150)
HUMAN|HGNC=3252|UniProtKB=O14602	Eukaryotic translation initiation factor 1A, Y-chromosomal;EIF1AY;ortholog	translation initiation factor(PC00224)
HUMAN|HGNC=25234|UniProtKB=Q9BUV0	Arginine/serine-rich protein 1;RSRP1;ortholog	
HUMAN|HGNC=19155|UniProtKB=P57773	Gap junction alpha-9 protein;GJA9;ortholog	gap junction(PC00105)
HUMAN|HGNC=8569|UniProtKB=P29122	Proprotein convertase subtilisin/kexin type 6;PCSK6;ortholog	serine protease(PC00203)
HUMAN|HGNC=28477|UniProtKB=Q8NBM8	Prenylcysteine oxidase-like;PCYOX1L;ortholog	lyase(PC00144)

HUMAN|HGNC=23243|UniProtKB=Q9NQR7	Coiled-coil domain-containing protein 177;CCDC177;ortholog	
HUMAN|HGNC=24103|UniProtKB=Q9H1I8	Activating signal cointegrator 1 complex subunit 2;ASCC2;ortholog	
HUMAN|HGNC=5817|UniProtKB=P01619	Immunoglobulin kappa variable 3-20;IGKV3-20;ortholog	immunoglobulin(PC00123)
HUMAN|HGNC=23168|UniProtKB=Q8IYD8	Fanconi anemia group M protein;FANCM;ortholog	DNA metabolism protein(PC00009)
HUMAN|HGNC=6427|UniProtKB=Q04695	Keratin, type I cytoskeletal 17;KRT17;ortholog	
HUMAN|HGNC=26363|UniProtKB=Q86YJ7	Ankyrin repeat domain-containing protein 13B;ANKRD13B;ortholog	
HUMAN|HGNC=9758|UniProtKB=P62820	Ras-related protein Rab-1A;RAB1A;ortholog	
HUMAN|HGNC=18142|UniProtKB=Q9Y4D1	Disheveled-associated activator of morphogenesis 1;DAAM1;ortholog	
HUMAN|HGNC=21685|UniProtKB=Q6YHK3	CD109 antigen;CD109;ortholog	protease inhibitor(PC00191)
HUMAN|HGNC=15527|UniProtKB=Q86Z14	Beta-klotho;KLB;ortholog	glycosidase(PC00110)
HUMAN|HGNC=15985|UniProtKB=Q4LDE5	Sushi, von Willebrand factor type A, EGF and pentraxin domain-containing protein 1;SVEP1;ortholog	
HUMAN|HGNC=12586|UniProtKB=P22695	Cytochrome b-c1 complex subunit 2, mitochondrial;UQCRC2;ortholog	metalloprotease(PC00153)
HUMAN|HGNC=8891|UniProtKB=P52209	6-phosphogluconate dehydrogenase, decarboxylating;PGD;ortholog	dehydrogenase(PC00092)
HUMAN|HGNC=14669|UniProtKB=P81605	Dermcidin;DCD;ortholog	
HUMAN|HGNC=9533|UniProtKB=P25789	Proteasome subunit alpha type-4;PSMA4;ortholog	protease(PC00190)
HUMAN|HGNC=1050|UniProtKB=P55957	BH3-interacting domain death agonist;BID;ortholog	
HUMAN|HGNC=9785|UniProtKB=P51148	Ras-related protein Rab-5C;RAB5C;ortholog	
HUMAN|HGNC=6501|UniProtKB=P13473	Lysosome-associated membrane glycoprotein 2;LAMP2;ortholog	membrane trafficking regulatory protein(PC00151)
HUMAN|HGNC=15480|UniProtKB=Q9NSV4	Protein diaphanous homolog 3;DIAPH3;ortholog	
HUMAN|HGNC=3540|UniProtKB=Q96RI0	Proteinase-activated receptor 4;F2RL3;ortholog	G-protein coupled receptor(PC00021)
HUMAN|HGNC=2911|UniProtKB=P36957	Dihydrolipoyllysine-residue succinyltransferase component of 2-oxoglutarate dehydrogenase complex, mitochondrial;DLST;ortholog	transferase(PC00220)
HUMAN|HGNC=13954|UniProtKB=O60814	Histone H2B type 1-K;H2BC12;ortholog	histone(PC00118)
HUMAN|HGNC=25501|UniProtKB=Q9NW68	BSD domain-containing protein 1;BSDC1;ortholog	
HUMAN|HGNC=5479|UniProtKB=P01877	Immunoglobulin heavy constant alpha 2;IGHA2;ortholog	immunoglobulin(PC00123)
HUMAN|HGNC=2678|UniProtKB=P14868	Aspartate--tRNA ligase, cytoplasmic;DARS1;ortholog	aminoacyl-tRNA synthetase(PC00047)

HUMAN|HGNC=1743|UniProtKB=Q99459	Cell division cycle 5-like protein;CDC5L;ortholog	
HUMAN|HGNC=18647|UniProtKB=Q8WWV3	Reticulon-4-interacting protein 1, mitochondrial;RTN4IP1;ortholog	dehydrogenase(PC00092)
HUMAN|HGNC=10608|UniProtKB=P47872	Secretin receptor;SCTR;ortholog	transmembrane signal receptor(PC00197)
HUMAN|HGNC=51815|UniProtKB=A0A1W2PPM1	Cytoplasmic polyadenylated homeobox-like;CPHXL;ortholog	homeodomain transcription factor(PC00119)
HUMAN|HGNC=561|UniProtKB=O95782	AP-2 complex subunit alpha-1;AP2A1;ortholog	membrane traffic protein(PC00150)
HUMAN|HGNC=1874|UniProtKB=P23528	Cofilin-1;CFL1;ortholog	non-motor actin binding protein(PC00165)
HUMAN|HGNC=11408|UniProtKB=Q13043	Serine/threonine-protein kinase 4;STK4;ortholog	non-receptor serine/threonine protein kinase(PC00167)
HUMAN|HGNC=3534|UniProtKB=P05160	Coagulation factor XIII B chain;F13B;ortholog	
HUMAN|HGNC=15848|UniProtKB=Q14141	Septin-6;SEPTIN6;ortholog	cytoskeletal protein(PC00085)
HUMAN|HGNC=30282|UniProtKB=Q3MIN7	Ral guanine nucleotide dissociation stimulator-like 3;RGL3;ortholog	guanyl-nucleotide exchange factor(PC00113)
HUMAN|HGNC=26664|UniProtKB=Q8N841	Tubulin polyglutamylase TTLL6;TTLL6;ortholog	microtubule or microtubule-binding cytoskeletal protein(PC00157)
HUMAN|HGNC=25648|UniProtKB=Q8N5J2	Ubiquitin carboxyl-terminal hydrolase MINDY-1;MINDY1;ortholog	
HUMAN|HGNC=29084|UniProtKB=O75123	Zinc finger protein 623;ZNF623;ortholog	
HUMAN|HGNC=29608|UniProtKB=Q86UE4	Protein LYRIC;MTDH;ortholog	
HUMAN|HGNC=16298|UniProtKB=Q504T8	Midnolin;MIDN;ortholog	
HUMAN|HGNC=14388|UniProtKB=Q9HCN6	Platelet glycoprotein VI;GP6;ortholog	immunoglobulin receptor superfamily(PC00124)
HUMAN|HGNC=12879|UniProtKB=P21506	Zinc finger protein 10;ZNF10;ortholog	C2H2 zinc finger transcription factor(PC00248)
HUMAN|HGNC=23147|UniProtKB=Q70J99	Protein unc-13 homolog D;UNC13D;ortholog	
HUMAN|HGNC=12591|UniProtKB=P06132	Uroporphyrinogen decarboxylase;UROD;ortholog	methyltransferase(PC00155)
HUMAN|HGNC=12665|UniProtKB=P18206	Vinculin;VCL;ortholog	
HUMAN|HGNC=9859|UniProtKB=P52306	Rap1 GTPase-GDP dissociation stimulator 1;RAP1GDS1;ortholog	
HUMAN|HGNC=555|UniProtKB=O43747	AP-1 complex subunit gamma-1;AP1G1;ortholog	membrane traffic protein(PC00150)
HUMAN|HGNC=28422|UniProtKB=Q8NCE0	tRNA-splicing endonuclease subunit Sen2;TSEN2;ortholog	endodeoxyribonuclease(PC00093)
HUMAN|HGNC=25654|UniProtKB=Q86YA3	Protein ZGRF1;ZGRF1;ortholog	

HUMAN|HGNC=992|UniProtKB=Q07817	Bcl-2-like protein 1;BCL2L1;ortholog	
HUMAN|HGNC=492|UniProtKB=P16157	Ankyrin-1;ANK1;ortholog	
HUMAN|HGNC=23416|UniProtKB=Q641Q2	WASH complex subunit 2A;WASHC2A;ortholog	scaffold/adaptor protein(PC00226)
HUMAN|HGNC=2230|UniProtKB=P53621	Coatomer subunit alpha;COPA;ortholog	vesicle coat protein(PC00235)
HUMAN|HGNC=25620|UniProtKB=Q8WWA1	Transmembrane protein 40;TMEM40;ortholog	
HUMAN|HGNC=6156|UniProtKB=P05106	Integrin beta-3;ITGB3;ortholog	integrin(PC00126)
HUMAN|HGNC=6412|UniProtKB=P04264	Keratin, type II cytoskeletal 1;KRT1;ortholog	
HUMAN|HGNC=9253|UniProtKB=P62937	Peptidyl-prolyl cis-trans isomerase A;PPIA;ortholog	chaperone(PC00072)
HUMAN|HGNC=5741|UniProtKB=P01602	Immunoglobulin kappa variable 1-5;IGKV1-5;ortholog	immunoglobulin(PC00123)
HUMAN|HGNC=13485|UniProtKB=Q9BXU7	Ubiquitin carboxyl-terminal hydrolase 26;USP26;ortholog	cysteine protease(PC00081)
HUMAN|HGNC=9354|UniProtKB=P30048	Thioredoxin-dependent peroxide reductase, mitochondrial;PRDX3;ortholog	peroxidase(PC00180)
HUMAN|HGNC=12492|UniProtKB=P61088	Ubiquitin-conjugating enzyme E2 N;UBE2N;ortholog	ubiquitin-protein ligase(PC00234)
HUMAN|HGNC=251|UniProtKB=P00326	Alcohol dehydrogenase 1C;ADH1C;ortholog	dehydrogenase(PC00092)
HUMAN|HGNC=24358|UniProtKB=Q9H2G4	Testis-specific Y-encoded-like protein 2;TSPYL2;ortholog	chromatin/chromatin-binding, or -regulatory protein(PC00077)
HUMAN|HGNC=9725|UniProtKB=P06737	Glycogen phosphorylase, liver form;PYGL;ortholog	glycosyltransferase(PC00111)
HUMAN|HGNC=42937|UniProtKB=P0DME0	Protein SETSIP;SETSIP;ortholog	chromatin/chromatin-binding, or -regulatory protein(PC00077)
HUMAN|HGNC=22125|UniProtKB=O95522	PRAME family member 12;PRAMEF12;ortholog	
HUMAN|HGNC=2988|UniProtKB=Q92608	Dedicator of cytokinesis protein 2;DOCK2;ortholog	guanyl-nucleotide exchange factor(PC00113)
HUMAN|HGNC=28472|UniProtKB=Q9BTV4	Transmembrane protein 43;TMEM43;ortholog	
HUMAN|HGNC=14258|UniProtKB=Q9Y5K6	CD2-associated protein;CD2AP;ortholog	
HUMAN|HGNC=4746|UniProtKB=P62807	Histone H2B type 1-C/E/F/G/I;H2BC4;ortholog	histone(PC00118)
HUMAN|HGNC=9862|UniProtKB=P61225	Ras-related protein Rap-2b;RAP2B;ortholog	small GTPase(PC00208)
HUMAN|HGNC=19760|UniProtKB=Q96T51	RUN and FYVE domain-containing protein 1;RUFY1;ortholog	
HUMAN|HGNC=17973|UniProtKB=Q9P109	Beta-1,3-galactosyl-O-glycosyl-glycoprotein beta-1,6-N-acetylglucosaminyltransferase 4;GCNT4;ortholog	glycosyltransferase(PC00111)
HUMAN|HGNC=22958|UniProtKB=Q9NR12	PDZ and LIM domain protein 7;PDLIM7;ortholog	actin or actin-binding cytoskeletal protein(PC00041)

HUMAN|HGNC=6463|UniProtKB=O43790	Keratin, type II cuticular Hb6;KRT86;ortholog	
HUMAN|HGNC=17376|UniProtKB=Q9UIJ7	GTP:AMP phosphotransferase AK3, mitochondrial;AK3;ortholog	nucleotide kinase(PC00172)
HUMAN|HGNC=8955|UniProtKB=P50453	Serpin B9;SERPINB9;ortholog	protease inhibitor(PC00191)
HUMAN|HGNC=8861|UniProtKB=P02776	Platelet factor 4;PF4;ortholog	chemokine(PC00074)
HUMAN|HGNC=21054|UniProtKB=Q86UC2	Radial spoke head protein 3 homolog;RSPH3;ortholog	structural protein(PC00211)
HUMAN|HGNC=10760|UniProtKB=Q01105	Protein SET;SET;ortholog	chromatin/chromatin-binding, or -regulatory protein(PC00077)
HUMAN|HGNC=18173|UniProtKB=Q9NZ08	Endoplasmic reticulum aminopeptidase 1;ERAP1;ortholog	metalloprotease(PC00153)
HUMAN|HGNC=5785|UniProtKB=P06310	Immunoglobulin kappa variable 2-30;IGKV2-30;ortholog	immunoglobulin(PC00123)
HUMAN|HGNC=24485|UniProtKB=Q9UFP1	Golgi-associated kinase 1A;GASK1A;ortholog	
HUMAN|HGNC=9604|UniProtKB=P23219	Prostaglandin G/H synthase 1;PTGS1;ortholog	oxygenase(PC00177)
HUMAN|HGNC=6407|UniProtKB=P01116	GTPase KRas;KRAS;ortholog	small GTPase(PC00208)
HUMAN|HGNC=29502|UniProtKB=Q0ZLH3	Pejvakin;PJVK;ortholog	
HUMAN|HGNC=37237|UniProtKB=A6NDY0	Embryonic polyadenylate-binding protein 2;PABPN1L;ortholog	translation initiation factor(PC00224)
HUMAN|HGNC=11148|UniProtKB=Q16658	Fascin;FSCN1;ortholog	non-motor actin binding protein(PC00165)
HUMAN|HGNC=8504|UniProtKB=Q969R2	Oxysterol-binding protein 2;OSBP2;ortholog	
HUMAN|HGNC=16090|UniProtKB=O00264	Membrane-associated progesterone receptor component 1;PGRMC1;ortholog	transmembrane signal receptor(PC00197)
HUMAN|HGNC=6038|UniProtKB=Q12906	Interleukin enhancer-binding factor 3;ILF3;ortholog	RNA metabolism protein(PC00031)
HUMAN|HGNC=10721|UniProtKB=P16109	P-selectin;SELP;ortholog	
HUMAN|HGNC=7377|UniProtKB=Q9UJ68	Mitochondrial peptide methionine sulfoxide reductase;MSRA;ortholog	reductase(PC00198)
HUMAN|HGNC=16768|UniProtKB=O60391	Glutamate receptor ionotropic, NMDA 3B;GRIN3B;ortholog	transmembrane signal receptor(PC00197)
HUMAN|HGNC=6169|UniProtKB=Q14624	Inter-alpha-trypsin inhibitor heavy chain H4;ITIH4;ortholog	protease inhibitor(PC00191)
HUMAN|HGNC=8043|UniProtKB=Q02818	Nucleobindin-1;NUCB1;ortholog	calmodulin-related(PC00061)
HUMAN|HGNC=30268|UniProtKB=Q96AX2	Ras-related protein Rab-37;RAB37;ortholog	
HUMAN|HGNC=6517|UniProtKB=P18428	Lipopolysaccharide-binding protein;LBP;ortholog	
HUMAN|HGNC=16695|UniProtKB=P51572	B-cell receptor-associated protein 31;BCAP31;ortholog	membrane traffic protein(PC00150)
HUMAN|HGNC=19409|UniProtKB=O43300	Leucine-rich repeat transmembrane neuronal protein 2;LRRTM2;ortholog	

HUMAN|HGNC=10363|UniProtKB=P18124	60S ribosomal protein L7;RPL7;ortholog	ribosomal protein(PC00202)
HUMAN|HGNC=2532|UniProtKB=P08311	Cathepsin G;CTSG;ortholog	serine protease(PC00203)
HUMAN|HGNC=30205|UniProtKB=Q9H7C9	Mth938 domain-containing protein;AAMDC;ortholog	
HUMAN|HGNC=25473|UniProtKB=Q9P2N6	KAT8 regulatory NSL complex subunit 3;KANSL3;ortholog	
HUMAN|HGNC=26555|UniProtKB=Q3SY00	Testis-specific protein 10-interacting protein;TSGA10IP;ortholog	
HUMAN|HGNC=26928|UniProtKB=Q9UKY7	Protein CDV3 homolog;CDV3;ortholog	
HUMAN|HGNC=17168|UniProtKB=Q9H2M9	Rab3 GTPase-activating protein non-catalytic subunit;RAB3GAP2;ortholog	G-protein modulator(PC00022)
HUMAN|HGNC=4472|UniProtKB=Q14330	N-arachidonyl glycine receptor;GPR18;ortholog	G-protein coupled receptor(PC00021)
HUMAN|HGNC=7729|UniProtKB=Q15019	Septin-2;SEPTIN2;ortholog	cytoskeletal protein(PC00085)
HUMAN|HGNC=14294|UniProtKB=Q9BYB0	SH3 and multiple ankyrin repeat domains protein 3;SHANK3;ortholog	
HUMAN|HGNC=30611|UniProtKB=Q8TCJ2	Dolichyl-diphosphooligosaccharide--protein glycosyltransferase subunit STT3B;STT3B;ortholog	glycosyltransferase(PC00111)
HUMAN|HGNC=16276|UniProtKB=Q9C035	Tripartite motif-containing protein 5;TRIM5;ortholog	ubiquitin-protein ligase(PC00234)
HUMAN|HGNC=16051|UniProtKB=Q8TEW0	Partitioning defective 3 homolog;PARD3;ortholog	
HUMAN|HGNC=6446|UniProtKB=P05787	Keratin, type II cytoskeletal 8;KRT8;ortholog	
HUMAN|HGNC=2335|UniProtKB=Q2VPA4	Complement component receptor 1-like protein;CR1L;ortholog	
HUMAN|HGNC=607|UniProtKB=P02654	Apolipoprotein C-I;APOC1;ortholog	apolipoprotein(PC00052)
HUMAN|HGNC=679|UniProtKB=P52566	Rho GDP-dissociation inhibitor 2;ARHGDIB;ortholog	G-protein modulator(PC00022)
HUMAN|HGNC=4883|UniProtKB=P08603	Complement factor H;CFH;ortholog	
HUMAN|HGNC=5238|UniProtKB=P11021	Endoplasmic reticulum chaperone BiP;HSPA5;ortholog	
HUMAN|HGNC=3069|UniProtKB=P51452	Dual specificity protein phosphatase 3;DUSP3;ortholog	
HUMAN|HGNC=8761|UniProtKB=Q9BUL8	Programmed cell death protein 10;PDCD10;ortholog	
HUMAN|HGNC=5240|UniProtKB=P48741	Putative heat shock 70 kDa protein 7;HSPA7;ortholog	
HUMAN|HGNC=23688|UniProtKB=Q86UU0	B-cell CLL/lymphoma 9-like protein;BCL9L;ortholog	
HUMAN|HGNC=25296|UniProtKB=Q8IYM0	Protein FAM186B;FAM186B;ortholog	
HUMAN|HGNC=18423|UniProtKB=O75140	GATOR complex protein DEPDC5;DEPDC5;ortholog	GTPase-activating protein(PC00257)
HUMAN|HGNC=8765|UniProtKB=O75340	Programmed cell death protein 6;PDCD6;ortholog	actin or actin-binding cytoskeletal protein(PC00041)

HUMAN|HGNC=17964|UniProtKB=Q8TC12	Retinol dehydrogenase 11;RDH11;ortholog	dehydrogenase(PC00092)
HUMAN|HGNC=4814|UniProtKB=O60229	Kalirin;KALRN;ortholog	guanyl-nucleotide exchange factor(PC00113)
HUMAN|HGNC=4374|UniProtKB=O60234	Glia maturation factor gamma;GMFG;ortholog	intercellular signal molecule(PC00207)
HUMAN|HGNC=20625|UniProtKB=M0R2J8	Doublecortin domain-containing protein 1;DCDC1;ortholog	
HUMAN|HGNC=18304|UniProtKB=Q14019	Coactosin-like protein;COTL1;ortholog	non-motor actin binding protein(PC00165)
HUMAN|HGNC=33749|UniProtKB=Q52MB2	Coiled-coil domain-containing protein 184;CCDC184;ortholog	
HUMAN|HGNC=7694|UniProtKB=O14561	Acyl carrier protein, mitochondrial;NDUFAB1;ortholog	transfer/carrier protein(PC00219)
HUMAN|HGNC=5742|UniProtKB=A0A0C4DH72	Immunoglobulin kappa variable 1-6;IGKV1-6;ortholog	immunoglobulin(PC00123)
HUMAN|HGNC=12410|UniProtKB=Q9NY65	Tubulin alpha-8 chain;TUBA8;ortholog	tubulin(PC00228)
HUMAN|HGNC=25035|UniProtKB=Q5VVQ6	Ubiquitin thioesterase OTU1;YOD1;ortholog	cysteine protease(PC00081)
HUMAN|HGNC=6528|UniProtKB=P13796	Plastin-2;LCP1;ortholog	non-motor actin binding protein(PC00165)
HUMAN|HGNC=15582|UniProtKB=Q8WXI7	Mucin-16;MUC16;ortholog	
HUMAN|HGNC=5890|UniProtKB=P01705	Immunoglobulin lambda variable 2-23;IGLV2-23;ortholog	immunoglobulin(PC00123)
HUMAN|HGNC=1424|UniProtKB=P27708	CAD protein;CAD;ortholog	ligase(PC00142)
HUMAN|HGNC=21740|UniProtKB=Q6ZV73	FYVE, RhoGEF and PH domain-containing protein 6;FGD6;ortholog	guanyl-nucleotide exchange factor(PC00113)
HUMAN|HGNC=30697|UniProtKB=Q8TB96	T-cell immunomodulatory protein;ITFG1;ortholog	
HUMAN|HGNC=24636|UniProtKB=Q9UHB6	LIM domain and actin-binding protein 1;LIMA1;ortholog	actin or actin-binding cytoskeletal protein(PC00041)
HUMAN|HGNC=24903|UniProtKB=Q9Y520	Protein PRRC2C;PRRC2C;ortholog	RNA metabolism protein(PC00031)
HUMAN|HGNC=9070|UniProtKB=P08567	Pleckstrin;PLEK;ortholog	cytoskeletal protein(PC00085)
HUMAN|HGNC=1090|UniProtKB=Q03001	Dystonin;DST;ortholog	intermediate filament binding protein(PC00130)
HUMAN|HGNC=29007|UniProtKB=Q14CM0	FERM and PDZ domain-containing protein 4;FRMPD4;ortholog	
HUMAN|HGNC=14452|UniProtKB=Q9NYG5	Anaphase-promoting complex subunit 11;ANAPC11;ortholog	ubiquitin-protein ligase(PC00234)
HUMAN|HGNC=1247|UniProtKB=P09871	Complement C1s subcomponent;C1S;ortholog	serine protease(PC00203)
HUMAN|HGNC=5654|UniProtKB=P01825	Immunoglobulin heavy variable 4-59;IGHV4-59;ortholog	immunoglobulin(PC00123)
HUMAN|HGNC=94|UniProtKB=Q9BWD1	Acetyl-CoA acetyltransferase, cytosolic;ACAT2;ortholog	acyltransferase(PC00042)
HUMAN|HGNC=362|UniProtKB=P54819	Adenylate kinase 2, mitochondrial;AK2;ortholog	nucleotide kinase(PC00172)

HUMAN|HGNC=4458|UniProtKB=P06744	Glucose-6-phosphate isomerase;GPI;ortholog	isomerase(PC00135)
HUMAN|HGNC=25316|UniProtKB=Q5VZ19	Tudor domain-containing protein 10;TDRD10;ortholog	scaffold/adaptor protein(PC00226)
HUMAN|HGNC=8898|UniProtKB=P07205	Phosphoglycerate kinase 2;PGK2;ortholog	carbohydrate kinase(PC00065)
HUMAN|HGNC=8533|UniProtKB=P51575	P2X purinoceptor 1;P2RX1;ortholog	ligand-gated ion channel(PC00141)
HUMAN|HGNC=9112|UniProtKB=Q6P1K2	Polyamine-modulated factor 1;PMF1;ortholog	
HUMAN|HGNC=6317|UniProtKB=O43896	Kinesin-like protein KIF1C;KIF1C;ortholog	microtubule binding motor protein(PC00156)
HUMAN|HGNC=12669|UniProtKB=P21796	Voltage-dependent anion-selective channel protein 1;VDAC1;ortholog	voltage-gated ion channel(PC00241)
HUMAN|HGNC=5228|UniProtKB=P25686	DnaJ homolog subfamily B member 2;DNAJB2;ortholog	chaperone(PC00072)
HUMAN|HGNC=14248|UniProtKB=Q96RN5	Mediator of RNA polymerase II transcription subunit 15;MED15;ortholog	
HUMAN|HGNC=6050|UniProtKB=P29218	Inositol monophosphatase 1;IMPA1;ortholog	phosphatase(PC00181)
HUMAN|HGNC=17820|UniProtKB=Q9H0P0	Cytosolic 5'-nucleotidase 3A;NT5C3A;ortholog	esterase(PC00097)
HUMAN|HGNC=2213|UniProtKB=P12111	Collagen alpha-3(VI) chain;COL6A3;ortholog	extracellular matrix structural protein(PC00103)
HUMAN|HGNC=570|UniProtKB=P53677	AP-3 complex subunit mu-2;AP3M2;ortholog	membrane traffic protein(PC00150)
HUMAN|HGNC=237|UniProtKB=O43306	Adenylate cyclase type 6;ADCY6;ortholog	
HUMAN|HGNC=12475|UniProtKB=P62837	Ubiquitin-conjugating enzyme E2 D2;UBE2D2;ortholog	ubiquitin-protein ligase(PC00234)
HUMAN|HGNC=8052|UniProtKB=Q9UKK9	ADP-sugar pyrophosphatase;NUDT5;ortholog	pyrophosphatase(PC00196)
HUMAN|HGNC=9207|UniProtKB=Q9UFF9	CCR4-NOT transcription complex subunit 8;CNOT8;ortholog	mRNA polyadenylation factor(PC00146)
HUMAN|HGNC=11957|UniProtKB=Q93074	Mediator of RNA polymerase II transcription subunit 12;MED12;ortholog	general transcription factor(PC00259)
HUMAN|HGNC=7571|UniProtKB=Q9UKX3	Myosin-13;MYH13;ortholog	
HUMAN|HGNC=4718|UniProtKB=P10412	Histone H1.4;H1-4;ortholog	histone(PC00118)
HUMAN|HGNC=3213|UniProtKB=P26641	Elongation factor 1-gamma;EEF1G;ortholog	
HUMAN|HGNC=7732|UniProtKB=Q15843	NEDD8;NEDD8;ortholog	
HUMAN|HGNC=12388|UniProtKB=Q16762	Thiosulfate sulfurtransferase;TST;ortholog	transferase(PC00220)
HUMAN|HGNC=707|UniProtKB=P59998	Actin-related protein 2/3 complex subunit 4;ARPC4;ortholog	actin or actin-binding cytoskeletal protein(PC00041)
HUMAN|HGNC=24293|UniProtKB=Q70JA7	Chondroitin sulfate synthase 3;CHSY3;ortholog	glycosyltransferase(PC00111)
HUMAN|HGNC=5910|UniProtKB=P01718	Immunoglobulin lambda variable 3-27;IGLV3-27;ortholog	immunoglobulin(PC00123)

HUMAN|HGNC=10899|UniProtKB=P63208	S-phase kinase-associated protein 1;SKP1;ortholog	ubiquitin-protein ligase(PC00234)
HUMAN|HGNC=7641|UniProtKB=P54920	Alpha-soluble NSF attachment protein;NAPA;ortholog	membrane traffic protein(PC00150)
HUMAN|HGNC=9388|UniProtKB=P10644	cAMP-dependent protein kinase type I-alpha regulatory subunit;PRKAR1A;ortholog	kinase modulator(PC00140)
HUMAN|HGNC=24634|UniProtKB=Q9UBC2	Epidermal growth factor receptor substrate 15-like 1;EPS15L1;ortholog	membrane traffic protein(PC00150)
HUMAN|HGNC=6561|UniProtKB=P09382	Galectin-1;LGALS1;ortholog	extracellular matrix protein(PC00102)
HUMAN|HGNC=7590|UniProtKB=Q15746	Myosin light chain kinase, smooth muscle;MYLK;ortholog	
HUMAN|HGNC=1628|UniProtKB=P08571	Monocyte differentiation antigen CD14;CD14;ortholog	transmembrane signal receptor(PC00197)
HUMAN|HGNC=38476|UniProtKB=B9A064	Immunoglobulin lambda-like polypeptide 5;IGLL5;ortholog	immunoglobulin(PC00123)
HUMAN|HGNC=12010|UniProtKB=P09493	Tropomyosin alpha-1 chain;TPM1;ortholog	actin binding motor protein(PC00040)
HUMAN|HGNC=8510|UniProtKB=Q92882	Osteoclast-stimulating factor 1;OSTF1;ortholog	
HUMAN|HGNC=255|UniProtKB=P28332	Alcohol dehydrogenase 6;ADH6;ortholog	dehydrogenase(PC00092)
HUMAN|HGNC=10582|UniProtKB=Q9Y5Y9	Sodium channel protein type 10 subunit alpha;SCN10A;ortholog	voltage-gated ion channel(PC00241)
HUMAN|HGNC=5551|UniProtKB=A0A0C4DH33	Immunoglobulin heavy variable 1-24;IGHV1-24;ortholog	immunoglobulin(PC00123)
HUMAN|HGNC=18039|UniProtKB=Q9UGL1	Lysine-specific demethylase 5B;KDM5B;ortholog	histone modifying enzyme(PC00261)
HUMAN|HGNC=6535|UniProtKB=P00338	L-lactate dehydrogenase A chain;LDHA;ortholog	dehydrogenase(PC00092)
HUMAN|HGNC=4335|UniProtKB=P00367	Glutamate dehydrogenase 1, mitochondrial;GLUD1;ortholog	dehydrogenase(PC00092)
HUMAN|HGNC=16753|UniProtKB=P30041	Peroxiredoxin-6;PRDX6;ortholog	peroxidase(PC00180)
HUMAN|HGNC=26586|UniProtKB=Q8N2A0	Putative uncharacterized protein encoded by LINC00269;LINC00269;ortholog	
HUMAN|HGNC=20771|UniProtKB=P68371	Tubulin beta-4B chain;TUBB4B;ortholog	tubulin(PC00228)
HUMAN|HGNC=4800|UniProtKB=Q99714	3-hydroxyacyl-CoA dehydrogenase type-2;HSD17B10;ortholog	oxidoreductase(PC00176)
HUMAN|HGNC=19375|UniProtKB=Q9BV35	Calcium-binding mitochondrial carrier protein SCaMC-3;SLC25A23;ortholog	mitochondrial carrier protein(PC00158)
HUMAN|HGNC=16089|UniProtKB=O15173	Membrane-associated progesterone receptor component 2;PGRMC2;ortholog	transmembrane signal receptor(PC00197)
HUMAN|HGNC=600|UniProtKB=P02647	Apolipoprotein A-I;APOA1;ortholog	apolipoprotein(PC00052)
HUMAN|HGNC=12012|UniProtKB=P06753	Tropomyosin alpha-3 chain;TPM3;ortholog	actin binding motor protein(PC00040)
HUMAN|HGNC=11126|UniProtKB=P53814	Smoothelin;SMTN;ortholog	
HUMAN|HGNC=23435|UniProtKB=Q8N4J0	Carnosine N-methyltransferase;CARNMT1;ortholog	
HUMAN|HGNC=4227|UniProtKB=P50395	Rab GDP dissociation inhibitor beta;GDI2;ortholog	G-protein modulator(PC00022)

HUMAN|HGNC=7223|UniProtKB=P25325	3-mercaptopyruvate sulfurtransferase;MPST;ortholog	transferase(PC00220)
HUMAN|HGNC=10497|UniProtKB=P31151	Protein S100-A7;S100A7;ortholog	calmodulin-related(PC00061)
HUMAN|HGNC=14265|UniProtKB=Q86UP0	Cadherin-24;CDH24;ortholog	cadherin(PC00057)
HUMAN|HGNC=29125|UniProtKB=Q9UQ16	Dynamin-3;DNM3;ortholog	membrane traffic protein(PC00150)
HUMAN|HGNC=9767|UniProtKB=O00194	Ras-related protein Rab-27B;RAB27B;ortholog	
HUMAN|HGNC=24723|UniProtKB=Q9H6R3	Acyl-CoA synthetase short-chain family member 3, mitochondrial;ACSS3;ortholog	ligase(PC00142)
HUMAN|HGNC=5554|UniProtKB=P01743	Immunoglobulin heavy variable 1-46;IGHV1-46;ortholog	immunoglobulin(PC00123)
HUMAN|HGNC=23397|UniProtKB=Q5T1M5	FK506-binding protein 15;FKBP15;ortholog	
HUMAN|HGNC=29989|UniProtKB=Q86YP4	Transcriptional repressor p66-alpha;GATAD2A;ortholog	DNA-binding transcription factor(PC00218)
HUMAN|HGNC=10378|UniProtKB=P52815	39S ribosomal protein L12, mitochondrial;MRPL12;ortholog	ribosomal protein(PC00202)
HUMAN|HGNC=575|UniProtKB=Q9Y587	AP-4 complex subunit sigma-1;AP4S1;ortholog	vesicle coat protein(PC00235)
HUMAN|HGNC=17150|UniProtKB=Q9Y2V2	Calcium-regulated heat-stable protein 1;CARHSP1;ortholog	RNA metabolism protein(PC00031)
HUMAN|HGNC=544|UniProtKB=P08133	Annexin A6;ANXA6;ortholog	calcium-binding protein(PC00060)
HUMAN|HGNC=12016|UniProtKB=P29144	Tripeptidyl-peptidase 2;TPP2;ortholog	serine protease(PC00203)
HUMAN|HGNC=17943|UniProtKB=Q9GZP9	Derlin-2;DERL2;ortholog	primary active transporter(PC00068)
HUMAN|HGNC=8078|UniProtKB=O95158	Neurexophilin-4;NXPH4;ortholog	intercellular signal molecule(PC00207)
HUMAN|HGNC=9645|UniProtKB=Q05209	Tyrosine-protein phosphatase non-receptor type 12;PTPN12;ortholog	
HUMAN|HGNC=10569|UniProtKB=P29508	Serpin B3;SERPINB3;ortholog	protease inhibitor(PC00191)
HUMAN|HGNC=1339|UniProtKB=P13671	Complement component C6;C6;ortholog	
HUMAN|HGNC=12011|UniProtKB=P07951	Tropomyosin beta chain;TPM2;ortholog	actin binding motor protein(PC00040)
HUMAN|HGNC=11767|UniProtKB=O43294	Transforming growth factor beta-1-induced transcript 1 protein;TGFB1I1;ortholog	actin or actin-binding cytoskeletal protein(PC00041)
HUMAN|HGNC=9087|UniProtKB=Q04941	Proteolipid protein 2;PLP2;ortholog	membrane traffic protein(PC00150)
HUMAN|HGNC=3419|UniProtKB=P42566	Epidermal growth factor receptor substrate 15;EPS15;ortholog	membrane traffic protein(PC00150)
HUMAN|HGNC=4432|UniProtKB=P17174	Aspartate aminotransferase, cytoplasmic;GOT1;ortholog	transaminase(PC00216)
HUMAN|HGNC=2475|UniProtKB=P01034	Cystatin-C;CST3;ortholog	
HUMAN|HGNC=6707|UniProtKB=P33241	Lymphocyte-specific protein 1;LSP1;ortholog	non-motor actin binding protein(PC00165)

HUMAN|HGNC=6484|UniProtKB=Q16363	Laminin subunit alpha-4;LAMA4;ortholog	
HUMAN|HGNC=9451|UniProtKB=P04070	Vitamin K-dependent protein C;PROC;ortholog	serine protease(PC00203)
HUMAN|HGNC=18399|UniProtKB=Q8N5I4	Dehydrogenase/reductase SDR family member on chromosome X;DHRSX;ortholog	dehydrogenase(PC00092)
HUMAN|HGNC=26922|UniProtKB=Q5T890	DNA excision repair protein ERCC-6-like 2;ERCC6L2;ortholog	
HUMAN|HGNC=24932|UniProtKB=Q9NZ63	Telomere length and silencing protein 1 homolog;C9orf78;ortholog	
HUMAN|HGNC=850|UniProtKB=P48047	ATP synthase subunit O, mitochondrial;ATP5PO;ortholog	ATP synthase(PC00002)
HUMAN|HGNC=15606|UniProtKB=Q9UHA4	Ragulator complex protein LAMTOR3;LAMTOR3;ortholog	scaffold/adaptor protein(PC00226)
HUMAN|HGNC=21874|UniProtKB=Q96ME1	F-box/LRR-repeat protein 18;FBXL18;ortholog	
HUMAN|HGNC=16701|UniProtKB=P19105	Myosin regulatory light chain 12A;MYL12A;ortholog	actin or actin-binding cytoskeletal protein(PC00041)
HUMAN|HGNC=14959|UniProtKB=Q96T37	RNA-binding protein 15;RBM15;ortholog	RNA metabolism protein(PC00031)
HUMAN|HGNC=9786|UniProtKB=P20340	Ras-related protein Rab-6A;RAB6A;ortholog	
HUMAN|HGNC=711|UniProtKB=P49407	Beta-arrestin-1;ARRB1;ortholog	scaffold/adaptor protein(PC00226)
HUMAN|HGNC=18368|UniProtKB=Q9UM07	Protein-arginine deiminase type-4;PADI4;ortholog	
HUMAN|HGNC=26531|UniProtKB=Q96M89	Coiled-coil domain-containing protein 138;CCDC138;ortholog	
HUMAN|HGNC=5239|UniProtKB=P17066	Heat shock 70 kDa protein 6;HSPA6;ortholog	
HUMAN|HGNC=6724|UniProtKB=P51884	Lumican;LUM;ortholog	
HUMAN|HGNC=673|UniProtKB=Q07960	Rho GTPase-activating protein 1;ARHGAP1;ortholog	GTPase-activating protein(PC00257)
HUMAN|HGNC=11326|UniProtKB=P51571	Translocon-associated protein subunit delta;SSR4;ortholog	
HUMAN|HGNC=6171|UniProtKB=Q08881	Tyrosine-protein kinase ITK/TSK;ITK;ortholog	
HUMAN|HGNC=25708|UniProtKB=Q96JM4	Leucine-rich repeat and IQ domain-containing protein 1;LRRIQ1;ortholog	
HUMAN|HGNC=12582|UniProtKB=P14927	Cytochrome b-c1 complex subunit 7;UQCRB;ortholog	reductase(PC00198)
HUMAN|HGNC=11133|UniProtKB=O95721	Synaptosomal-associated protein 29;SNAP29;ortholog	SNARE protein(PC00034)
HUMAN|HGNC=5933|UniProtKB=A0A0B4J1Y8	Immunoglobulin lambda variable 9-49;IGLV9-49;ortholog	immunoglobulin(PC00123)
HUMAN|HGNC=10632|UniProtKB=P13501	C-C motif chemokine 5;CCL5;ortholog	cytokine(PC00083)
HUMAN|HGNC=29262|UniProtKB=Q9P2H3	Intraflagellar transport protein 80 homolog;IFT80;ortholog	
HUMAN|HGNC=704|UniProtKB=O15143	Actin-related protein 2/3 complex subunit 1B;ARPC1B;ortholog	actin or actin-binding cytoskeletal protein(PC00041)

HUMAN|HGNC=7821|UniProtKB=P14543	Nidogen-1;NID1;ortholog	transmembrane signal receptor(PC00197)
HUMAN|HGNC=3189|UniProtKB=P68104	Elongation factor 1-alpha 1;EEF1A1;ortholog	translation factor(PC00223)
HUMAN|HGNC=9414|UniProtKB=Q13976	cGMP-dependent protein kinase 1;PRKG1;ortholog	non-receptor serine/threonine protein kinase(PC00167)
HUMAN|HGNC=4801|UniProtKB=P40939	Trifunctional enzyme subunit alpha, mitochondrial;HADHA;ortholog	
HUMAN|HGNC=9395|UniProtKB=P05771	Protein kinase C beta type;PRKCB;ortholog	non-receptor serine/threonine protein kinase(PC00167)
HUMAN|HGNC=11088|UniProtKB=Q9H2G2	STE20-like serine/threonine-protein kinase;SLK;ortholog	
HUMAN|HGNC=10989|UniProtKB=Q00325	Phosphate carrier protein, mitochondrial;SLC25A3;ortholog	mitochondrial carrier protein(PC00158)
HUMAN|HGNC=11403|UniProtKB=Q9Y6E0	Serine/threonine-protein kinase 24;STK24;ortholog	
HUMAN|HGNC=25516|UniProtKB=Q8TER5	Rho guanine nucleotide exchange factor 40;ARHGEF40;ortholog	guanyl-nucleotide exchange factor(PC00113)
HUMAN|HGNC=2728|UniProtKB=P39656	Dolichyl-diphosphooligosaccharide--protein glycosyltransferase 48 kDa subunit;DDOST;ortholog	glycosyltransferase(PC00111)
HUMAN|HGNC=9299|UniProtKB=P67775	Serine/threonine-protein phosphatase 2A catalytic subunit alpha isoform;PPP2CA;ortholog	protein phosphatase(PC00195)
HUMAN|HGNC=11357|UniProtKB=Q92783	Signal transducing adapter molecule 1;STAM;ortholog	scaffold/adaptor protein(PC00226)
HUMAN|HGNC=21230|UniProtKB=Q9P1F3	Costars family protein ABRACL;ABRACL;ortholog	
HUMAN|HGNC=11099|UniProtKB=Q14527	Helicase-like transcription factor;HLTF;ortholog	
HUMAN|HGNC=3382|UniProtKB=Q08495	Dematin;DMTN;ortholog	actin or actin-binding cytoskeletal protein(PC00041)
HUMAN|HGNC=13921|UniProtKB=O95870	Phosphatidylserine lipase ABHD16A;ABHD16A;ortholog	serine protease(PC00203)
HUMAN|HGNC=1507|UniProtKB=P55212	Caspase-6;CASP6;ortholog	protease(PC00190)
HUMAN|HGNC=8044|UniProtKB=P80303	Nucleobindin-2;NUCB2;ortholog	calmodulin-related(PC00061)
HUMAN|HGNC=43788|UniProtKB=Q8NHM4	Putative trypsin-6;PRSS3P2;ortholog	serine protease(PC00203)
HUMAN|HGNC=16721|UniProtKB=A6NKQ9	Choriogonadotropin subunit beta variant 1;CGB1;ortholog	peptide hormone(PC00179)
HUMAN|HGNC=6834|UniProtKB=P27338	Amine oxidase [flavin-containing] B;MAOB;ortholog	oxidase(PC00175)
HUMAN|HGNC=16499|UniProtKB=Q96DA2	Ras-related protein Rab-39B;RAB39B;ortholog	
HUMAN|HGNC=20471|UniProtKB=O95777	U6 snRNA-associated Sm-like protein LSm8;LSM8;ortholog	RNA splicing factor(PC00148)
HUMAN|HGNC=10498|UniProtKB=P05109	Protein S100-A8;S100A8;ortholog	calmodulin-related(PC00061)
HUMAN|HGNC=20908|UniProtKB=Q86YF9	Zinc finger protein DZIP1;DZIP1;ortholog	

HUMAN|HGNC=4827|UniProtKB=P68871	Hemoglobin subunit beta;HBB;ortholog	
HUMAN|HGNC=22880|UniProtKB=Q96JG8	Melanoma-associated antigen D4;MAGED4B;ortholog	scaffold/adaptor protein(PC00226)
HUMAN|HGNC=8515|UniProtKB=Q9HC10	Otoferlin;OTOF;ortholog	membrane trafficking regulatory protein(PC00151)
HUMAN|HGNC=10458|UniProtKB=Q92681	Regulatory solute carrier protein family 1 member 1;RSC1A1;ortholog	membrane trafficking regulatory protein(PC00151)
HUMAN|HGNC=1476|UniProtKB=P07384	Calpain-1 catalytic subunit;CAPN1;ortholog	cysteine protease(PC00081)
HUMAN|HGNC=30904|UniProtKB=O60763	General vesicular transport factor p115;USO1;ortholog	membrane traffic protein(PC00150)
HUMAN|HGNC=4823|UniProtKB=P69905	Hemoglobin subunit alpha;HBA2;ortholog	
HUMAN|HGNC=3815|UniProtKB=Q12951	Forkhead box protein I1;FOXI1;ortholog	winged helix/forkhead transcription factor(PC00246)
HUMAN|HGNC=7516|UniProtKB=Q9HC84	Mucin-5B;MUC5B;ortholog	extracellular matrix protein(PC00102)
HUMAN|HGNC=4555|UniProtKB=P22352	Glutathione peroxidase 3;GPX3;ortholog	peroxidase(PC00180)
HUMAN|HGNC=20473|UniProtKB=Q9BX63	Fanconi anemia group J protein;BRIP1;ortholog	DNA helicase(PC00011)
HUMAN|HGNC=24728|UniProtKB=A2RTX5	Threonine--tRNA ligase 2, cytoplasmic;TARS3;ortholog	aminoacyl-tRNA synthetase(PC00047)
HUMAN|HGNC=4635|UniProtKB=P21266	Glutathione S-transferase Mu 3;GSTM3;ortholog	transferase(PC00220)
HUMAN|HGNC=18451|UniProtKB=Q8NI22	Multiple coagulation factor deficiency protein 2;MCFD2;ortholog	membrane traffic protein(PC00150)
HUMAN|HGNC=4395|UniProtKB=P19086	Guanine nucleotide-binding protein G(z) subunit alpha;GNAZ;ortholog	heterotrimeric G-protein(PC00117)
HUMAN|HGNC=12403|UniProtKB=Q8WZ42	Titin;TTN;ortholog	
HUMAN|HGNC=5753|UniProtKB=P01593	Immunoglobulin kappa variable 1D-33;IGKV1D-33;ortholog	immunoglobulin(PC00123)
HUMAN|HGNC=119|UniProtKB=Q15067	Peroxisomal acyl-coenzyme A oxidase 1;ACOX1;ortholog	oxidoreductase(PC00176)
HUMAN|HGNC=12849|UniProtKB=P31946	14-3-3 protein beta/alpha;YWHAB;ortholog	scaffold/adaptor protein(PC00226)
HUMAN|HGNC=10991|UniProtKB=P05141	ADP/ATP translocase 2;SLC25A5;ortholog	transfer/carrier protein(PC00219)
HUMAN|HGNC=5593|UniProtKB=P0DP02	Immunoglobulin heavy variable 3-30-3;IGHV3-30-3;ortholog	immunoglobulin(PC00123)
HUMAN|HGNC=794|UniProtKB=P31939	Bifunctional purine biosynthesis protein ATIC;ATIC;ortholog	
HUMAN|HGNC=6871|UniProtKB=P28482	Mitogen-activated protein kinase 1;MAPK1;ortholog	non-receptor serine/threonine protein kinase(PC00167)
HUMAN|HGNC=6752|UniProtKB=P20645	Cation-dependent mannose-6-phosphate receptor;M6PR;ortholog	membrane traffic protein(PC00150)
HUMAN|HGNC=9261|UniProtKB=Q13356	RING-type E3 ubiquitin-protein ligase PPIL2;PPIL2;ortholog	chaperone(PC00072)

HUMAN|HGNC=25397|UniProtKB=Q6MZP7	Protein lin-54 homolog;LIN54;ortholog	
HUMAN|HGNC=6631|UniProtKB=P49257	Protein ERGIC-53;LMAN1;ortholog	membrane traffic protein(PC00150)
HUMAN|HGNC=16524|UniProtKB=P61106	Ras-related protein Rab-14;RAB14;ortholog	
HUMAN|HGNC=5620|UniProtKB=P01780	Immunoglobulin heavy variable 3-7;IGHV3-7;ortholog	immunoglobulin(PC00123)
HUMAN|HGNC=18449|UniProtKB=P28838	Cytosol aminopeptidase;LAP3;ortholog	metalloprotease(PC00153)
HUMAN|HGNC=25240|UniProtKB=Q4KMQ2	Anoctamin-6;ANO6;ortholog	
HUMAN|HGNC=7002|UniProtKB=A6NDR6	Putative homeobox protein Meis3-like 1;MEIS3P1;ortholog	homeodomain transcription factor(PC00119)
HUMAN|HGNC=19692|UniProtKB=Q96F24	Nuclear receptor-binding factor 2;NRBF2;ortholog	transcription cofactor(PC00217)
HUMAN|HGNC=2713|UniProtKB=O75935	Dynactin subunit 3;DCTN3;ortholog	microtubule binding motor protein(PC00156)
HUMAN|HGNC=14010|UniProtKB=Q8IWI9	MAX gene-associated protein;MGA;ortholog	Rel homology transcription factor(PC00252)
HUMAN|HGNC=678|UniProtKB=P52565	Rho GDP-dissociation inhibitor 1;ARHGDIA;ortholog	G-protein modulator(PC00022)
HUMAN|HGNC=21416|UniProtKB=Q92485	Acid sphingomyelinase-like phosphodiesterase 3b;SMPDL3B;ortholog	phosphodiesterase(PC00185)
HUMAN|HGNC=20653|UniProtKB=Q8IVB4	Sodium/hydrogen exchanger 9;SLC9A9;ortholog	secondary carrier transporter(PC00258)
HUMAN|HGNC=3951|UniProtKB=Q16595	Frataxin, mitochondrial;FXN;ortholog	transfer/carrier protein(PC00219)
HUMAN|HGNC=13582|UniProtKB=Q9UK99	F-box only protein 3;FBXO3;ortholog	
HUMAN|HGNC=7133|UniProtKB=O14686	Histone-lysine N-methyltransferase 2D;KMT2D;ortholog	
HUMAN|HGNC=2949|UniProtKB=Q8TD57	Dynein heavy chain 3, axonemal;DNAH3;ortholog	microtubule binding motor protein(PC00156)
HUMAN|HGNC=12488|UniProtKB=P68036	Ubiquitin-conjugating enzyme E2 L3;UBE2L3;ortholog	ubiquitin-protein ligase(PC00234)
HUMAN|HGNC=20851|UniProtKB=Q5T0N5	Formin-binding protein 1-like;FNBP1L;ortholog	
HUMAN|HGNC=24731|UniProtKB=Q8NBF2	NHL repeat-containing protein 2;NHLRC2;ortholog	
HUMAN|HGNC=29943|UniProtKB=Q9NT68	Teneurin-2;TENM2;ortholog	
HUMAN|HGNC=30410|UniProtKB=Q9BXA6	Testis-specific serine/threonine-protein kinase 6;TSSK6;ortholog	non-receptor serine/threonine protein kinase(PC00167)
HUMAN|HGNC=144|UniProtKB=P63261	Actin, cytoplasmic 2;ACTG1;ortholog	actin and actin related protein(PC00039)
HUMAN|HGNC=9495|UniProtKB=P24158	Myeloblastin;PRTN3;ortholog	serine protease(PC00203)
HUMAN|HGNC=3694|UniProtKB=P02679	Fibrinogen gamma chain;FGG;ortholog	intercellular signal molecule(PC00207)
HUMAN|HGNC=537|UniProtKB=P07355	Annexin A2;ANXA2;ortholog	calcium-binding protein(PC00060)

HUMAN|HGNC=16219|UniProtKB=Q8TEA8	D-aminoacyl-tRNA deacylase 1;DTD1;ortholog	esterase(PC00097)
HUMAN|HGNC=28512|UniProtKB=A6NEQ2	Protein FAM181B;FAM181B;ortholog	
HUMAN|HGNC=24526|UniProtKB=Q6DD88	Atlastin-3;ATL3;ortholog	heterotrimeric G-protein(PC00117)
HUMAN|HGNC=29051|UniProtKB=O75071	EF-hand calcium-binding domain-containing protein 14;EFCAB14;ortholog	
HUMAN|HGNC=8648|UniProtKB=Q15366	Poly(rC)-binding protein 2;PCBP2;ortholog	RNA metabolism protein(PC00031)
HUMAN|HGNC=30013|UniProtKB=Q96PD5	N-acetylmuramoyl-L-alanine amidase;PGLYRP2;ortholog	
HUMAN|HGNC=5596|UniProtKB=P01772	Immunoglobulin heavy variable 3-33;IGHV3-33;ortholog	immunoglobulin(PC00123)
HUMAN|HGNC=25457|UniProtKB=Q8N3F0	Maturin;MTURN;ortholog	
HUMAN|HGNC=18756|UniProtKB=Q9BYZ6	Rho-related BTB domain-containing protein 2;RHOBTB2;ortholog	small GTPase(PC00208)
HUMAN|HGNC=20731|UniProtKB=Q9HAV0	Guanine nucleotide-binding protein subunit beta-4;GNB4;ortholog	heterotrimeric G-protein(PC00117)
HUMAN|HGNC=6442|UniProtKB=P13647	Keratin, type II cytoskeletal 5;KRT5;ortholog	
HUMAN|HGNC=9658|UniProtKB=P29350	Tyrosine-protein phosphatase non-receptor type 6;PTPN6;ortholog	protein phosphatase(PC00195)
HUMAN|HGNC=4624|UniProtKB=P48637	Glutathione synthetase;GSS;ortholog	ligase(PC00142)
HUMAN|HGNC=9802|UniProtKB=P15153	Ras-related C3 botulinum toxin substrate 2;RAC2;ortholog	small GTPase(PC00208)
HUMAN|HGNC=8942|UniProtKB=P48595	Serpin B10;SERPINB10;ortholog	protease inhibitor(PC00191)
HUMAN|HGNC=22222|UniProtKB=Q9C0H2	Protein tweety homolog 3;TTYH3;ortholog	ion channel(PC00133)
HUMAN|HGNC=5895|UniProtKB=P01709	Immunoglobulin lambda variable 2-8;IGLV2-8;ortholog	immunoglobulin(PC00123)
HUMAN|HGNC=23192|UniProtKB=Q9H5L6	DNA transposase THAP9;THAP9;ortholog	
HUMAN|HGNC=5382|UniProtKB=O75874	Isocitrate dehydrogenase [NADP] cytoplasmic;IDH1;ortholog	dehydrogenase(PC00092)
HUMAN|HGNC=9300|UniProtKB=P62714	Serine/threonine-protein phosphatase 2A catalytic subunit beta isoform;PPP2CB;ortholog	protein phosphatase(PC00195)
HUMAN|HGNC=6766|UniProtKB=Q8WXG6	MAP kinase-activating death domain protein;MADD;ortholog	
HUMAN|HGNC=11179|UniProtKB=P00441	Superoxide dismutase [Cu-Zn];SOD1;ortholog	oxidoreductase(PC00176)
HUMAN|HGNC=18676|UniProtKB=Q86XP3	ATP-dependent RNA helicase DDX42;DDX42;ortholog	
HUMAN|HGNC=6616|UniProtKB=P48059	LIM and senescent cell antigen-like-containing domain protein 1;LIMS1;ortholog	cell junction protein(PC00070)
HUMAN|HGNC=21265|UniProtKB=Q9NZP8	Complement C1r subcomponent-like protein;C1RL;ortholog	serine protease(PC00203)
HUMAN|HGNC=11056|UniProtKB=P48067	Sodium- and chloride-dependent glycine transporter 1;SLC6A9;ortholog	primary active transporter(PC00068)
HUMAN|HGNC=5713|UniProtKB=P01591	Immunoglobulin J chain;JCHAIN;ortholog	immunoglobulin(PC00123)

HUMAN|HGNC=12649|UniProtKB=O95292	Vesicle-associated membrane protein-associated protein B/C;VAPB;ortholog	membrane trafficking regulatory protein(PC00151)
HUMAN|HGNC=16820|UniProtKB=Q14162	Scavenger receptor class F member 1;SCARF1;ortholog	membrane traffic protein(PC00150)
HUMAN|HGNC=652|UniProtKB=P84077	ADP-ribosylation factor 1;ARF1;ortholog	G-protein(PC00020)
HUMAN|HGNC=4553|UniProtKB=P07203	Glutathione peroxidase 1;GPX1;ortholog	peroxidase(PC00180)
HUMAN|HGNC=6447|UniProtKB=P35527	Keratin, type I cytoskeletal 9;KRT9;ortholog	
HUMAN|HGNC=18994|UniProtKB=Q8TDC3	Serine/threonine-protein kinase BRSK1;BRSK1;ortholog	non-receptor serine/threonine protein kinase(PC00167)
HUMAN|HGNC=615|UniProtKB=Q13790	Apolipoprotein F;APOF;ortholog	apolipoprotein(PC00052)
HUMAN|HGNC=28984|UniProtKB=Q12768	WASH complex subunit 5;WASHC5;ortholog	membrane traffic protein(PC00150)
HUMAN|HGNC=2479|UniProtKB=O76096	Cystatin-F;CST7;ortholog	
HUMAN|HGNC=15904|UniProtKB=Q9H8E8	Cysteine-rich protein 2-binding protein;KAT14;ortholog	
HUMAN|HGNC=5580|UniProtKB=P01762	Immunoglobulin heavy variable 3-11;IGHV3-11;ortholog	immunoglobulin(PC00123)
HUMAN|HGNC=3778|UniProtKB=P02751	Fibronectin;FN1;ortholog	intercellular signal molecule(PC00207)
HUMAN|HGNC=29129|UniProtKB=O94919	Endonuclease domain-containing 1 protein;ENDOD1;ortholog	
