# Supplementary material for: Platelet-Rich Fibrin Increases BMP2 Expression in Oral Fibroblasts via Activation of TGF-β Signaling
Source: Int J Mol Sci. 2021 Jul 25;22(15):7935. doi: 10.3390/ijms22157935 (PMC8347014; doi:10.3390/ijms22157935)
Supplement: Supplementary file 1 [file ijms-22-07935-s001.zip › Supplementary Table 2.pdf]

Table S2. List of the 1791 GO terms derived by PANTHER analysis system based on proteomics analysis.

<table>
<tr><th>GO term</th><th>Gene ID</th><th>Name</th></tr>
<tr><td>PC00123</td><td>HUMAN|HGNC=5575|UniProtKB=A0A0B4J1V2,HUMAN|HGNC=5825|UniProtKB=A0A0C4DH25,HUMAN|HGNC=5549|UniProtKB=A0A0C4DH31,HUMAN|HGNC=5480|UniProtKB=P01880,HUMAN|HGNC=5659|UniProtKB=A0A0C4DH38,HUMAN|Gene=KV311_HUMAN|UniProtKB=P04433,HUMAN|HGNC=38476|UniProtKB=B9A064,HUMAN|HGNC=5877|UniProtKB=P01703,HUMAN|HGNC=5834|UniProtKB=P06312,HUMAN|HGNC=5861|UniProtKB=A0M8Q6,HUMAN|HGNC=5647|UniProtKB=A0A087WSY4,HUMAN|HGNC=5606|UniProtKB=P01763,HUMAN|HGNC=5759|UniProtKB=A0A087WSZ0,HUMAN|HGNC=5576|UniProtKB=P01817,HUMAN|HGNC=5541|UniProtKB=P01871,HUMAN|HGNC=5585|UniProtKB=A0A0C4DH32,HUMAN|HGNC=5897|UniProtKB=A0A075B6K4,HUMAN|HGNC=5526|UniProtKB=P01859,HUMAN|Ensembl=ENSG00000282639|UniProtKB=A0A0J9YX35,HUMAN|HGNC=5739|UniProtKB=A0A075B6S9,HUMAN|HGNC=5880|UniProtKB=P01700,HUMAN|HGNC=5624|UniProtKB=A0A0B4J1X5,HUMAN|HGNC=5905|UniProtKB=P80748,HUMAN|HGNC=5896|UniProtKB=P01715,HUMAN|HGNC=5580|UniProtKB=P01762,HUMAN|HGNC=5633|UniProtKB=A0A075B7D8,HUMAN|HGNC=5582|UniProtKB=A0A0B4J1V0,HUMAN|HGNC=5478|UniProtKB=P01876,HUMAN|HGNC=5652|UniProtKB=A0A075B6R2,HUMAN|HGNC=5836|UniProtKB=A0A0C4DH24,HUMAN|HGNC=5895|UniProtKB=P01709,HUMAN|HGNC=5619|UniProtKB=A0A0C4DH42,HUMAN|HGNC=5887|UniProtKB=P01706,HUMAN|HGNC=5604|UniProtKB=A0A0B4J1X8,HUMAN|HGNC=5662|UniProtKB=A0A0B4J1U7,HUMAN|HGNC=5746|UniProtKB=P01611,HUMAN|HGNC=5821|UniProtKB=A0A075B6H7,HUMAN|HGNC=5713|UniProtKB=P01591,HUMAN|HGNC=5857|UniProtKB=P0DOY3,HUMAN|HGNC=5889|UniProtKB=A0A075B6J9,HUMAN|HGNC=5610|UniProtKB=P01767,HUMAN|Ensembl=ENSG00000282150|UniProtKB=A0A0J9YY99,HUMAN|HGNC=5730|UniProtKB=A0A0C4DH73,HUMAN|HGNC=5527|UniProtKB=P01860,HUMAN|HGNC=5783|UniProtKB=A0A075B6P5,HUMAN|HGNC=5753|UniProtKB=P01593,HUMAN|HGNC=5879|UniProtKB=P01699,HUMAN|HGNC=5800|UniProtKB=A0A075B6S2,HUMAN|HGNC=5645|UniProtKB=A0A0C4DH34,HUMAN|HGNC=5554|UniProtKB=P01743,HUMAN|HGNC=5742|UniProtKB=A0A0C4DH72,HUMAN|HGNC=5634|UniProtKB=A0A075B7F0,HUMAN|HGNC=5816|UniProtKB=P01624,HUMAN|HGNC=5910|UniProtKB=P01718,HUMAN|HGNC=5931|UniProtKB=A0A075B6I0,HUMAN|HGNC=5933|UniProtKB=A0A0B4J1Y8,HUMAN|HGNC=5563|UniProtKB=A0A075B7D0,HUMAN|HGNC=5856|UniProtKB=P0DOY2,HUMAN|HGNC=5890|UniProtKB=P01705,HUMAN|HGNC=5732|UniProtKB=P04430,HUMAN|HGNC=5733|UniProtKB=P01599,HUMAN|HGNC=5528|UniProtKB=P01861,HUMAN|HGNC=5888|UniProtKB=P01704,HUMAN|HGNC=5551|UniProtKB=A0A0C4DH33,HUMAN|HGNC=5927|UniProtKB=P01721,HUMAN|HGNC=5918|UniProtKB=A0A075B6K5,HUMAN|HGNC=5657|UniProtKB=P0DP08,HUMAN|HGNC=5781|UniProtKB=A0A0C4DH68,HUMAN|HGNC=5930|UniProtKB=A0A075B6I9,HUMAN|HGNC=5901|UniProtKB=A0A075B6K0,HUMAN|HGNC=5743|UniProtKB=A0A0C4DH67,HUMAN|Gene=HV309_HUMAN|UniProtKB=P01782,HUMAN|HGNC=5654|UniProtKB=P01825,HUMAN|HGNC=5553|UniProtKB=A0A0A0MS14,HUMAN|G</td><td>immunoglobulin</td></tr>
</table>

	ene=HV108_HUMAN|UniProtKB=P0DP01,HUMAN|HGNC=5620|UniProtKB=P01780,HUMAN|HGNC=5598|UniProtKB=A0A0C4DH35,HUMAN|HGNC=5756|UniProtKB=P04432,HUMAN|HGNC=5617|UniProtKB=A0A075B6Q5,HUMAN|HGNC=5594|UniProtKB=P0DP03,HUMAN|HGNC=5735|UniProtKB=A0A075B6S5,HUMAN|HGNC=5655|UniProtKB=A0A0C4DH41,HUMAN|HGNC=5636|UniProtKB=A0A075B7B8,HUMAN|HGNC=5586|UniProtKB=A0A0B4J1V1,HUMAN|HGNC=5581|UniProtKB=P01766,HUMAN|HGNC=5622|UniProtKB=A0A0B4J1Y9,HUMAN|HGNC=5785|UniProtKB=P06310,HUMAN|HGNC=5593|UniProtKB=P0DP02,HUMAN|HGNC=5479|UniProtKB=P01877,HUMAN|HGNC=5596|UniProtKB=P01772,HUMAN|HGNC=5898|UniProtKB=A0A075B6K2,HUMAN|HGNC=5550|UniProtKB=P23083,HUMAN|HGNC=5929|UniProtKB=P04211,HUMAN|HGNC=5882|UniProtKB=P01701,HUMAN|HGNC=5525|UniProtKB=P01857,HUMAN|HGNC=5908|UniProtKB=P01717,HUMAN|HGNC=5601|UniProtKB=A0A0C4DH36,HUMAN|HGNC=5716|UniProtKB=P01834,HUMAN|HGNC=5588|UniProtKB=P01764,HUMAN|HGNC=5741|UniProtKB=P01602,HUMAN|HGNC=5817|UniProtKB=P01619	
GO:0009396	HUMAN|HGNC=871|UniProtKB=Q9UII2	folic acid-containing compound biosynthetic process
GO:0035267	HUMAN|HGNC=17780|UniProtKB=Q562R1,HUMAN|HGNC=12347|UniProtKB=Q9Y4A5,HUMAN|HGNC=10474|UniProtKB=Q9Y265,HUMAN|HGNC=10475|UniProtKB=Q9Y230	NuA4 histone acetyltransferase complex
GO:0004842	HUMAN|HGNC=12488|UniProtKB=P68036,HUMAN|HGNC=12491|UniProtKB=P61081,HUMAN|HGNC=12492|UniProtKB=P61088,HUMAN|HGNC=11617|UniProtKB=Q15369,HUMAN|HGNC=12476|UniProtKB=P61077,HUMAN|HGNC=2552|UniProtKB=Q13617,HUMAN|HGNC=12475|UniProtKB=P62837,HUMAN|HGNC=2551|UniProtKB=Q13616,HUMAN|HGNC=12494|UniProtKB=Q13404,HUMAN|HGNC=17598|UniProtKB=Q9Y385	ubiquitin-protein transferase activity
GO:0043254	HUMAN|HGNC=7641|UniProtKB=P54920,HUMAN|HGNC=10534|UniProtKB=Q9NR31	regulation of protein-containing complex assembly
PC00147	HUMAN|HGNC=25758|UniProtKB=Q8TED0,HUMAN|HGNC=2717|UniProtKB=Q16531	RNA processing factor
GO:0004674	HUMAN|HGNC=9391|UniProtKB=P13861,HUMAN|HGNC=9414|UniProtKB=Q13976,HUMAN|HGNC=9406|UniProtKB=Q16513,HUMAN|HGNC=9380|UniProtKB=P17612,HUMAN|HGNC=12401|UniProtKB=P33981,HUMAN|HGNC=30765|UniProtKB=Q9UKE5,HUMAN|HGNC=9392|UniProtKB=P31323,HUMAN|HGNC=9395|UniProtKB=P05771,HUMAN|HGNC=18994|UniProtKB=Q8TDC3,HUMAN|HGNC=10432|UniProtKB=P51812,HUMAN|HGNC=1459|UniProtKB=Q14012,HUMAN|HGNC=11408|UniProtKB=Q13043,HUMAN|HGNC=30410|UniProtKB=Q9BXA6,HUMAN|HGNC=7590|UniProtKB=Q15746,HUMAN|HGNC=10252|UniProtKB=O75116,HUMAN|HGNC=20292|UniProtKB=Q9Y376,HUMAN|HGNC=19825|UniProtKB=Q6SA08,HUMAN|HGNC=9381|UniProtKB=P22694,HUMAN|HGNC=9386|UniProtKB=Q9UGJ0,HUMAN|HGNC=1745|UniProtKB=O00311,HUMAN|HGNC=1738|UniProtKB=Q9Y5S2	protein serine/threonine kinase activity
GO:0016740	HUMAN|HGNC=11834|UniProtKB=P29401,HUMAN|HGNC=28959|UniProtKB=Q14008,HUMAN|HGNC=8527|UniProtKB=P55809,HUMAN|HGNC=12388|UniProtKB=Q16762,HUMAN|HGNC=7223|UniProtKB=P25325	transferase activity

<table>
<tr><td>GO:0030990</td><td>HUMAN|HGNC=8898|UniProtKB=P07205,HUMAN|HGNC=2949|UniProtKB=Q8TD57,HUMAN|HGNC=26555|UniProtKB=Q3SY00,HUMAN|HGNC=20908|UniProtKB=Q86YF9,HUMAN|HGNC=2952|UniProtKB=Q96JB1,HUMAN|HGNC=19747|UniProtKB=Q86UK5,HUMAN|HGNC=14906|UniProtKB=Q8N4C6,HUMAN|HGNC=26664|UniProtKB=Q8N841,HUMAN|HGNC=11119|UniProtKB=Q99835,HUMAN|HGNC=17060|UniProtKB=Q92845</td><td>intraciliary transport particle</td></tr>
<tr><td>GO:0007283</td><td>HUMAN|HGNC=26951|UniProtKB=Q86YL5</td><td>spermatogenesis</td></tr>
<tr><td>GO:0034620</td><td>HUMAN|HGNC=5241|UniProtKB=P11142,HUMAN|HGNC=5261|UniProtKB=P10809,HUMAN|HGNC=5239|UniProtKB=P17066,HUMAN|HGNC=5240|UniProtKB=P48741</td><td>cellular response to unfolded protein</td></tr>
<tr><td>GO:0032880</td><td>HUMAN|HGNC=16276|UniProtKB=Q9C035</td><td>regulation of protein localization</td></tr>
<tr><td>GO:0097192</td><td>HUMAN|HGNC=992|UniProtKB=Q07817</td><td>extrinsic apoptotic signaling pathway in absence of ligand</td></tr>
<tr><td>GO:0018193</td><td>HUMAN|HGNC=9261|UniProtKB=Q13356,HUMAN|HGNC=26664|UniProtKB=Q8N841</td><td>peptidyl-amino acid modification</td></tr>
<tr><td>GO:0043542</td><td>HUMAN|HGNC=10499|UniProtKB=P06702,HUMAN|HGNC=533|UniProtKB=P04083,HUMAN|HGNC=7178|UniProtKB=Q13201</td><td>endothelial cell migration</td></tr>
<tr><td>GO:0097060</td><td>HUMAN|HGNC=188|UniProtKB=O14672</td><td>synaptic membrane</td></tr>
<tr><td>GO:0098609</td><td>HUMAN|HGNC=6616|UniProtKB=P48059,HUMAN|HGNC=3757|UniProtKB=O75955,HUMAN|HGNC=3230|UniProtKB=Q9NYQ7,HUMAN|HGNC=3048|UniProtKB=Q02413,HUMAN|HGNC=11828|UniProtKB=Q9UDY2,HUMAN|HGNC=15447|UniProtKB=Q9Y4G6,HUMAN|HGNC=3231|UniProtKB=Q9HCU4,HUMAN|HGNC=30377|UniProtKB=Q14160,HUMAN|HGNC=23112|UniProtKB=Q8TDW7</td><td>cell-cell adhesion</td></tr>
<tr><td>GO:0032956</td><td>HUMAN|HGNC=18756|UniProtKB=Q9BYZ6,HUMAN|HGNC=6111|UniProtKB=Q13576,HUMAN|HGNC=9802|UniProtKB=P15153,HUMAN|HGNC=9801|UniProtKB=P63000</td><td>regulation of actin cytoskeleton organization</td></tr>
<tr><td>GO:0051539</td><td>HUMAN|HGNC=118|UniProtKB=Q99798,HUMAN|HGNC=117|UniProtKB=P21399</td><td>4 iron, 4 sulfur cluster binding</td></tr>
<tr><td>GO:0090263</td><td>HUMAN|HGNC=33704|UniProtKB=Q66K66,HUMAN|HGNC=9275|UniProtKB=P35813</td><td>positive regulation of canonical Wnt signaling pathway</td></tr>
<tr><td>GO:0008287</td><td>HUMAN|HGNC=9302|UniProtKB=P30153,HUMAN|HGNC=9308|UniProtKB=Q15257,HUMAN|HGNC=9314|UniProtKB=Q08209,HUMAN|HGNC=9304|UniProtKB=P63151</td><td>protein serine/threonine phosphatase complex</td></tr>
<tr><td>GO:0008175</td><td>HUMAN|HGNC=26022|UniProtKB=Q7L0Y3</td><td>tRNA methyltransferase activity</td></tr>
<tr><td>GO:0000712</td><td>HUMAN|HGNC=23845|UniProtKB=Q8IY92</td><td>resolution of meiotic recombination intermediates</td></tr>
<tr><td>GO:0006414</td><td>HUMAN|HGNC=10372|UniProtKB=P05386,HUMAN|HGNC=3189|UniProtKB=P68104,HUMAN|HGNC=20|UniProtKB=P49588,HUMAN|HGNC=4162|UniProtKB=P41250,HUMAN|HGNC=3213|UniProtKB=P26641,HUMAN|HGNC=3200|UniProtKB=Q5VTE0,HUMAN|HGNC=3282|UniProtKB=P60842,HUMAN|HGNC=13759|UniProtKB=Q7L576,HUMAN|HGNC=10404|UniProtKB=P15880,HUMAN|HGNC=11572|UniProtKB=P26639,HUMAN|HGNC=17894|UniProtKB=Q14966,HUMAN|HGNC=9207|UniProtKB=Q9UFF9,HUMAN|HGNC=4622|UniProtKB=Q8IYD1,HUMAN|HGNC=1243|UniProtKB=Q07021,HU</td><td>translational elongation</td></tr>
</table>

GO:0001508	MAN|HGNC=4621|UniProtKB=P15170,HUMAN|HGNC=17970|UniProtKB=Q9BPZ3,HUMAN|Gene=EIF2S3L|UniProtKB=Q2VIR3,HUMAN|HGNC=12729|UniProtKB=P23381,HUMAN|HGNC=3285|UniProtKB=P23588,HUMAN|HGNC=3211|UniProtKB=P29692,HUMAN|HGNC=27424|UniProtKB=Q5T481,HUMAN|HGNC=24728|UniProtKB=A2RTX5,HUMAN|HGNC=3300|UniProtKB=P63241,HUMAN|HGNC=2678|UniProtKB=P14868,HUMAN|HGNC=3214|UniProtKB=P13639,HUMAN|HGNC=9583|UniProtKB=P26599,HUMAN|HGNC=3208|UniProtKB=P24534,HUMAN|HGNC=12420|UniProtKB=P49411 HUMAN|HGNC=1406|UniProtKB=Q9Y698,HUMAN|HGNC=10594|UniProtKB=Q01118,HUMAN|HGNC=4390|UniProtKB=P50148,HUMAN|HGNC=10582|UniProtKB=Q9Y5Y9	action potential
GO:1990023	HUMAN|HGNC=5048|UniProtKB=Q00839	mitotic spindle midzone
GO:0004714	HUMAN|HGNC=19296|UniProtKB=Q9UF33,HUMAN|HGNC=6091|UniProtKB=P06213	transmembrane receptor protein tyrosine kinase activity
PC00099	HUMAN|HGNC=28648|UniProtKB=Q8IYB7	exoribonuclease
GO:0008277	HUMAN|HGNC=711|UniProtKB=P49407	regulation of G protein-coupled receptor signaling pathway
GO:0035735	HUMAN|HGNC=15476|UniProtKB=P63167,HUMAN|HGNC=15848|UniProtKB=Q14141,HUMAN|HGNC=16053|UniProtKB=Q8N1V2,HUMAN|HGNC=20908|UniProtKB=Q86YF9,HUMAN|HGNC=2952|UniProtKB=Q96JB1,HUMAN|HGNC=25631|UniProtKB=Q96MT7,HUMAN|HGNC=1717|UniProtKB=Q16181,HUMAN|HGNC=3244|UniProtKB=Q9NZN3,HUMAN|HGNC=29262|UniProtKB=Q9P2H3,HUMAN|HGNC=7729|UniProtKB=Q15019	intraciliary transport involved in cilium assembly
GO:0046390	HUMAN|HGNC=9465|UniProtKB=P11908,HUMAN|HGNC=11834|UniProtKB=P29401,HUMAN|HGNC=9462|UniProtKB=P60891	ribose phosphate biosynthetic process
GO:0046488	HUMAN|HGNC=17059|UniProtKB=Q9NTJ5,HUMAN|HGNC=10542|UniProtKB=O95248	phosphatidylinositol metabolic process
GO:0015318	HUMAN|HGNC=10989|UniProtKB=Q00325,HUMAN|HGNC=11027|UniProtKB=P02730	inorganic molecular entity transmembrane transporter activity
GO:0019752	HUMAN|HGNC=117|UniProtKB=P21399,HUMAN|HGNC=5382|UniProtKB=O75874,HUMAN|HGNC=6970|UniProtKB=P40925,HUMAN|HGNC=5383|UniProtKB=P48735,HUMAN|HGNC=5386|UniProtKB=P51553	carboxylic acid metabolic process
GO:0010975	HUMAN|HGNC=11590|UniProtKB=Q16650	regulation of neuron projection development
GO:0005634	HUMAN|HGNC=19985|UniProtKB=P53999,HUMAN|HGNC=9545|UniProtKB=P28062,HUMAN|HGNC=9414|UniProtKB=Q13976,HUMAN|HGNC=16369|UniProtKB=Q99497,HUMAN|HGNC=12401|UniProtKB=P33981,HUMAN|HGNC=10496|UniProtKB=P06703,HUMAN|HGNC=9554|UniProtKB=Q99460,HUMAN|HGNC=8988|UniProtKB=Q13526,HUMAN|HGNC=4719|UniProtKB=P16401,HUMAN|HGNC=9536|UniProtKB=O14818,HUMAN|HGNC=12468|UniProtKB=P0CG48,HUMAN|HGNC=9846|UniProtKB=P62826,HUMAN|HGNC=17894|UniProtKB=Q14966,HUMAN|HGNC=9281|UniProtKB=P62136,HUMAN|HGNC=17598|UniProtKB=Q9Y385,HUMAN|HGNC=30646|UniProtKB=Q7KZF4,HUMAN|HGNC=25397|UniProtKB=Q6MZP7,HUMAN|HGNC=12476|UniProtKB=P61077,HUMAN|HGNC=14959|UniProtKB=Q96T37,HUMAN|HGNC=7732|UniProtKB=Q15843,H	nucleus

UMAN|HGNC=20250|UniProtKB=Q96IF1,HUMAN|HGNC=9275|Un
iProtKB=P35813,HUMAN|HGNC=13485|UniProtKB=Q9BXU7,HUM
AN|HGNC=9531|UniProtKB=P25787,HUMAN|HGNC=30025|UniPr
otKB=Q5VV67,HUMAN|HGNC=9459|UniProtKB=Q92786,HUMAN|
HGNC=6767|UniProtKB=Q15797,HUMAN|HGNC=11179|UniProtK
B=P00441,HUMAN|HGNC=5253|UniProtKB=P07900,HUMAN|HGN
C=9539|UniProtKB=P49721,HUMAN|HGNC=14060|UniProtKB=Q9
UBZ9,HUMAN|HGNC=9349|UniProtKB=Q9NQX1,HUMAN|HGNC=1
9825|UniProtKB=Q6SA08,HUMAN|HGNC=7326|UniProtKB=P2058
5,HUMAN|HGNC=25035|UniProtKB=Q5VVQ6,HUMAN|HGNC=938
6|UniProtKB=Q9UGJ0,HUMAN|HGNC=23818|UniProtKB=Q14684,
HUMAN|HGNC=7803|UniProtKB=Q12986,HUMAN|HGNC=20165|
UniProtKB=Q9P2P1,HUMAN|HGNC=9533|UniProtKB=P25789,HU
MAN|HGNC=29158|UniProtKB=Q9Y2G9,HUMAN|HGNC=23708|U
niProtKB=Q6ZR52,HUMAN|HGNC=9380|UniProtKB=P17612,HUMA
N|HGNC=29084|UniProtKB=O75123,HUMAN|HGNC=347|UniProt
KB=Q09666,HUMAN|HGNC=12463|UniProtKB=P0CG47,HUMAN|H
GNC=51815|UniProtKB=A0A1W2PPM1,HUMAN|HGNC=12458|Uni
ProtKB=P62987,HUMAN|HGNC=5238|UniProtKB=P11021,HUMAN
|HGNC=14451|UniProtKB=Q9BQK8,HUMAN|HGNC=16298|UniPro
tKB=Q504T8,HUMAN|HGNC=9381|UniProtKB=P22694,HUMAN|H
GNC=28972|UniProtKB=Q8IXZ2,HUMAN|HGNC=13506|UniProtKB
=Q8TBJ5,HUMAN|HGNC=33108|UniProtKB=Q6NX45,HUMAN|HG
NC=13061|UniProtKB=Q14587,HUMAN|HGNC=2062|UniProtKB=O
00299,HUMAN|HGNC=21605|UniProtKB=Q14151,HUMAN|HGNC=
18807|UniProtKB=O94983,HUMAN|HGNC=21539|UniProtKB=Q9B
X46,HUMAN|HGNC=7446|UniProtKB=Q9Y4I5,HUMAN|HGNC=429
37|UniProtKB=P0DME0,HUMAN|HGNC=17801|UniProtKB=Q8NHY
6,HUMAN|HGNC=7106|UniProtKB=P54252,HUMAN|HGNC=9885|
UniProtKB=P29374,HUMAN|HGNC=6871|UniProtKB=P28482,HUM
AN|HGNC=9308|UniProtKB=Q15257,HUMAN|HGNC=17177|UniPr
otKB=Q8IUI8,HUMAN|HGNC=5044|UniProtKB=P61978,HUMAN|H
GNC=11125|UniProtKB=P61956,HUMAN|HGNC=13518|UniProtKB
=Q9Y696,HUMAN|HGNC=1745|UniProtKB=O00311,HUMAN|HGN
C=28422|UniProtKB=Q8NCE0,HUMAN|HGNC=7670|UniProtKB=Q9
Y6Q9,HUMAN|HGNC=20597|UniProtKB=P61960,HUMAN|HGNC=3
0688|UniProtKB=Q86VP6,HUMAN|HGNC=3213|UniProtKB=P2664
1,HUMAN|HGNC=12666|UniProtKB=P55072,HUMAN|HGNC=8052
|UniProtKB=Q9UKK9,HUMAN|HGNC=15935|UniProtKB=Q9H4I2,H
UMAN|HGNC=12475|UniProtKB=P62837,HUMAN|HGNC=19312|
UniProtKB=Q56P03,HUMAN|HGNC=9261|UniProtKB=Q13356,HU
MAN|HGNC=10774|UniProtKB=P23246,HUMAN|HGNC=7640|Uni
ProtKB=Q99733,HUMAN|HGNC=18994|UniProtKB=Q8TDC3,HUMA
N|HGNC=7198|UniProtKB=Q86VD1,HUMAN|HGNC=6876|UniProt
KB=Q16539,HUMAN|HGNC=10432|UniProtKB=P51812,HUMAN|H
GNC=5237|UniProtKB=P34932,HUMAN|HGNC=20063|UniProtKB=
Q86T82,HUMAN|HGNC=9530|UniProtKB=P25786,HUMAN|HGNC=
28843|UniProtKB=Q9HC44,HUMAN|HGNC=12488|UniProtKB=P68
036,HUMAN|HGNC=12491|UniProtKB=P61081,HUMAN|HGNC=32
060|UniProtKB=Q5TEC6,HUMAN|HGNC=6400|UniProtKB=Q14974
,HUMAN|HGNC=16969|UniProtKB=Q92598,HUMAN|HGNC=5241|
UniProtKB=P11142,HUMAN|HGNC=1173|UniProtKB=Q5H9R7,HU
MAN|HGNC=9583|UniProtKB=P26599,HUMAN|HGNC=12628|Uni
ProtKB=P45974,HUMAN|HGNC=4718|UniProtKB=P10412,HUMAN

<table>
<tr><td></td><td>|HGNC=12738|UniProtKB=Q969T9,HUMAN|HGNC=12494|UniProtKB=Q13404,HUMAN|HGNC=24358|UniProtKB=Q9H2G4,HUMAN|HGNC=6401|UniProtKB=Q92973,HUMAN|HGNC=23077|UniProtKB=Q96FW1,HUMAN|HGNC=8783|UniProtKB=Q08499,HUMAN|HGNC=11124|UniProtKB=P55854,HUMAN|HGNC=20635|UniProtKB=Q14997,HUMAN|HGNC=12469|UniProtKB=P22314,HUMAN|HGNC=12612|UniProtKB=P54578,HUMAN|HGNC=10760|UniProtKB=Q01105,HUMAN|HGNC=20473|UniProtKB=Q9BX63,HUMAN|HGNC=26427|UniProtKB=Q96ND8,HUMAN|HGNC=9559|UniProtKB=Q13200,HUMAN|HGNC=9635|UniProtKB=Q12974,HUMAN|HGNC=25490|UniProtKB=Q96HA8,HUMAN|HGNC=9540|UniProtKB=P49720,HUMAN|HGNC=15912|UniProtKB=Q9UNZ2,HUMAN|HGNC=29327|UniProtKB=Q5VZ46,HUMAN|HGNC=15818|UniProtKB=Q9H0Z9,HUMAN|HGNC=3750|UniProtKB=Q13045,HUMAN|HGNC=4861|UniProtKB=Q9NRZ9,HUMAN|HGNC=17808|UniProtKB=Q9UPT8,HUMAN|HGNC=17860|UniProtKB=Q8NC51,HUMAN|HGNC=15667|UniProtKB=P58546,HUMAN|HGNC=26951|UniProtKB=Q86YL5,HUMAN|HGNC=24054|UniProtKB=Q8NG31,HUMAN|HGNC=2717|UniProtKB=Q16531,HUMAN|HGNC=18676|UniProtKB=Q86XP3,HUMAN|HGNC=27424|UniProtKB=Q5T481,HUMAN|HGNC=13109|UniProtKB=P17038,HUMAN|HGNC=9535|UniProtKB=P60900,HUMAN|HGNC=12492|UniProtKB=P61088,HUMAN|HGNC=12379|UniProtKB=Q15631,HUMAN|HGNC=30410|UniProtKB=Q9BXA6,HUMAN|HGNC=4716|UniProtKB=P16403,HUMAN|HGNC=9645|UniProtKB=Q05209,HUMAN|HGNC=10899|UniProtKB=P63208,HUMAN|HGNC=7547|UniProtKB=P10243,HUMAN|HGNC=9532|UniProtKB=P25788,HUMAN|HGNC=9537|UniProtKB=P20618,HUMAN|HGNC=20076|UniProtKB=Q96K76</td><td></td></tr>
<tr><td>GO:0140098</td><td>HUMAN|HGNC=10898|UniProtKB=Q15477,HUMAN|HGNC=17821|UniProtKB=O00148</td><td>catalytic activity, acting on RNA</td></tr>
<tr><td>GO:0043473</td><td>HUMAN|HGNC=9767|UniProtKB=O00194</td><td>pigmentation</td></tr>
<tr><td>GO:0030148</td><td>HUMAN|HGNC=24175|UniProtKB=Q9P035</td><td>sphingolipid biosynthetic process</td></tr>
<tr><td>GO:0033043</td><td>HUMAN|HGNC=8761|UniProtKB=Q9BUL8,HUMAN|HGNC=3350|UniProtKB=P06733,HUMAN|HGNC=3353|UniProtKB=P09104,HUMAN|HGNC=10534|UniProtKB=Q9NR31,HUMAN|HGNC=3354|UniProtKB=P13929</td><td>regulation of organelle organization</td></tr>
<tr><td>GO:0042632</td><td>HUMAN|HGNC=1869|UniProtKB=P11597,HUMAN|HGNC=603|UniProtKB=P04114</td><td>cholesterol homeostasis</td></tr>
<tr><td>GO:2000042</td><td>HUMAN|HGNC=17196|UniProtKB=Q8NG08</td><td>negative regulation of double-strand break repair via homologous recombination</td></tr>
<tr><td>GO:0007265</td><td>HUMAN|HGNC=9855|UniProtKB=P62834,HUMAN|HGNC=9862|UniProtKB=P61225,HUMAN|HGNC=6407|UniProtKB=P01116,HUMAN|HGNC=6766|UniProtKB=Q8WXG6,HUMAN|HGNC=9857|UniProtKB=P61224,HUMAN|HGNC=25361|UniProtKB=Q8N264,HUMAN|HGNC=9840|UniProtKB=P11234</td><td>Ras protein signal transduction</td></tr>
<tr><td>GO:0008180</td><td>HUMAN|HGNC=30747|UniProtKB=P61201,HUMAN|HGNC=21314|UniProtKB=Q8WXC6,HUMAN|HGNC=5239|UniProtKB=P17066,HUMAN|HGNC=5240|UniProtKB=P48741,HUMAN|HGNC=2239|UniProtKB=Q9UNS2</td><td>COP9 signalosome</td></tr>
</table>

GO:0004115	HUMAN|HGNC=8783|UniProtKB=Q08499	3',5'-cyclic-AMP phosphodiesterase activity
GO:0000228	HUMAN|HGNC=5048|UniProtKB=Q00839,HUMAN|HGNC=23845|UniProtKB=Q8IY92	nuclear chromosome
PC00223	HUMAN|HGNC=3189|UniProtKB=P68104,HUMAN|HGNC=4622|UniProtKB=Q8IYD1,HUMAN|HGNC=4621|UniProtKB=P15170,HUMAN|HGNC=3200|UniProtKB=Q5VTE0	translation factor
GO:0001707	HUMAN|HGNC=2188|UniProtKB=Q99715	mesoderm formation
GO:0036156	HUMAN|HGNC=8898|UniProtKB=P07205,HUMAN|HGNC=2949|UniProtKB=Q8TD57	inner dynein arm
GO:0016251	HUMAN|HGNC=17305|UniProtKB=Q9Y6J9	RNA polymerase II general transcription initiation factor activity
GO:0070628	HUMAN|HGNC=20635|UniProtKB=Q14997,HUMAN|HGNC=9813|UniProtKB=P54727	proteasome binding
GO:0048199	HUMAN|HGNC=10534|UniProtKB=Q9NR31,HUMAN|HGNC=24878|UniProtKB=Q9BVK6	vesicle targeting, to, from or within Golgi
GO:0097153	HUMAN|HGNC=1504|UniProtKB=P42574,HUMAN|HGNC=1507|UniProtKB=P55212	cysteine-type endopeptidase activity involved in apoptotic process
GO:1990904	HUMAN|HGNC=5043|UniProtKB=P31942,HUMAN|HGNC=3214|UniProtKB=P13639,HUMAN|HGNC=7910|UniProtKB=P06748	ribonucleoprotein complex
GO:1903008	HUMAN|HGNC=12666|UniProtKB=P55072	organelle disassembly
GO:0051205	HUMAN|HGNC=26801|UniProtKB=Q8N8Q8	protein insertion into membrane
GO:0034641	HUMAN|HGNC=2328|UniProtKB=P50416	cellular nitrogen compound metabolic process
GO:0006623	HUMAN|HGNC=568|UniProtKB=O14617,HUMAN|HGNC=1908|UniProtKB=Q96RL7,HUMAN|HGNC=23594|UniProtKB=Q709C8	protein targeting to vacuole
GO:0071944	HUMAN|HGNC=25648|UniProtKB=Q8N5J2	cell periphery
GO:1902911	HUMAN|HGNC=9386|UniProtKB=Q9UGJ0,HUMAN|HGNC=6091|UniProtKB=P06213	protein kinase complex
GO:0050839	HUMAN|HGNC=29943|UniProtKB=Q9NT68,HUMAN|HGNC=11828|UniProtKB=Q9UDY2,HUMAN|HGNC=29557|UniProtKB=Q0ZGT2,HUMAN|HGNC=11771|UniProtKB=Q15582	cell adhesion molecule binding
GO:0051092	HUMAN|HGNC=30140|UniProtKB=A7MCY6,HUMAN|HGNC=16391|UniProtKB=Q9H257,HUMAN|HGNC=16276|UniProtKB=Q9C035,HUMAN|HGNC=1628|UniProtKB=P08571,HUMAN|HGNC=3157|UniProtKB=Q92838,HUMAN|HGNC=29890|UniProtKB=Q86UT6,HUMAN|HGNC=9275|UniProtKB=P35813	positive regulation of NF-kappaB transcription factor activity
GO:0006470	HUMAN|HGNC=1173|UniProtKB=Q5H9R7,HUMAN|HGNC=19388|UniProtKB=P49593,HUMAN|HGNC=9673|UniProtKB=Q12913,HUMAN|HGNC=9304|UniProtKB=P63151	protein dephosphorylation
GO:1901361	HUMAN|HGNC=2689|UniProtKB=P09172,HUMAN|HGNC=82|UniProtKB=P09110	organic cyclic compound catabolic process
GO:0030154	HUMAN|HGNC=9866|UniProtKB=P13631,HUMAN|HGNC=6767|UniProtKB=Q15797,HUMAN|HGNC=4037|UniProtKB=P06241,HUMAN|HGNC=2188|UniProtKB=Q99715,HUMAN|HGNC=9583|UniProtKB=P26599,HUMAN|HGNC=3815|UniProtKB=Q12951,HUMAN|HGNC=7967|UniProtKB=Q96RI1,HUMAN|HGNC=12841|UniProtKB=P	cell differentiation

<table>
<tr><td></td><td>07947,HUMAN|HGNC=7640|UniProtKB=Q99733,HUMAN|HGNC=6735|UniProtKB=P07948,HUMAN|HGNC=24903|UniProtKB=Q9Y520</td><td></td></tr>
<tr><td>GO:2001237</td><td>HUMAN|HGNC=11283|UniProtKB=P12931</td><td>negative regulation of extrinsic apoptotic signaling pathway</td></tr>
<tr><td>GO:0006900</td><td>HUMAN|HGNC=5575|UniProtKB=A0A0B4J1V2,HUMAN|HGNC=607|UniProtKB=P02654,HUMAN|HGNC=9784|UniProtKB=P61020,HUMAN|HGNC=5645|UniProtKB=A0A0C4DH34,HUMAN|HGNC=5549|UniProtKB=A0A0C4DH31,HUMAN|HGNC=555|UniProtKB=O43747,HUMAN|HGNC=5554|UniProtKB=P01743,HUMAN|HGNC=5480|UniProtKB=P01880,HUMAN|HGNC=5659|UniProtKB=A0A0C4DH38,HUMAN|HGNC=5634|UniProtKB=A0A075B7F0,HUMAN|HGNC=38476|UniProtKB=B9A064,HUMAN|HGNC=2090|UniProtKB=P09496,HUMAN|HGNC=5861|UniProtKB=A0M8Q6,HUMAN|HGNC=5647|UniProtKB=A0A087WSY4,HUMAN|HGNC=5563|UniProtKB=A0A075B7D0,HUMAN|HGNC=5856|UniProtKB=P0DOY2,HUMAN|HGNC=1406|UniProtKB=Q9Y698,HUMAN|HGNC=3757|UniProtKB=O75955,HUMAN|HGNC=5606|UniProtKB=P01763,HUMAN|HGNC=9785|UniProtKB=P51148,HUMAN|HGNC=9801|UniProtKB=P63000,HUMAN|HGNC=5576|UniProtKB=P01817,HUMAN|HGNC=5528|UniProtKB=P01861,HUMAN|HGNC=5541|UniProtKB=P01871,HUMAN|HGNC=5585|UniProtKB=A0A0C4DH32,HUMAN|HGNC=9782|UniProtKB=P61018,HUMAN|HGNC=9802|UniProtKB=P15153,HUMAN|HGNC=5551|UniProtKB=A0A0C4DH33,HUMAN|HGNC=3244|UniProtKB=Q9NZN3,HUMAN|HGNC=11510|UniProtKB=Q8N9I0,HUMAN|HGNC=7228|UniProtKB=P22897,HUMAN|HGNC=5526|UniProtKB=P01859,HUMAN|HGNC=5657|UniProtKB=P0DP08,HUMAN|Ensembl=ENSG00000282639|UniProtKB=A0A0J9YX35,HUMAN|HGNC=19760|UniProtKB=Q96T51,HUMAN|Gene=HV309_HUMAN|UniProtKB=P01782,HUMAN|HGNC=18756|UniProtKB=Q9BYZ6,HUMAN|HGNC=5654|UniProtKB=P01825,HUMAN|HGNC=5553|UniProtKB=A0A0A0MS14,HUMAN|Gene=HV108_HUMAN|UniProtKB=P0DP01,HUMAN|HGNC=5624|UniProtKB=A0A0B4J1X5,HUMAN|HGNC=5620|UniProtKB=P01780,HUMAN|HGNC=23186|UniProtKB=Q14677,HUMAN|HGNC=5598|UniProtKB=A0A0C4DH35,HUMAN|HGNC=5580|UniProtKB=P01762,HUMAN|HGNC=5633|UniProtKB=A0A075B7D8,HUMAN|HGNC=5582|UniProtKB=A0A0B4J1V0,HUMAN|HGNC=5478|UniProtKB=P01876,HUMAN|HGNC=26068|UniProtKB=Q6IAA8,HUMAN|HGNC=5617|UniProtKB=A0A075B6Q5,HUMAN|HGNC=711|UniProtKB=P49407,HUMAN|HGNC=5594|UniProtKB=P0DP03,HUMAN|HGNC=5655|UniProtKB=A0A0C4DH41,HUMAN|HGNC=5636|UniProtKB=A0A075B7B8,HUMAN|HGNC=5586|UniProtKB=A0A0B4J1V1,HUMAN|HGNC=5581|UniProtKB=P01766,HUMAN|HGNC=5622|UniProtKB=A0A0B4J1Y9,HUMAN|HGNC=5652|UniProtKB=A0A075B6R2,HUMAN|HGNC=5593|UniProtKB=P0DP02,HUMAN|HGNC=11138|UniProtKB=P37840,HUMAN|HGNC=3419|UniProtKB=P42566,HUMAN|HGNC=5479|UniProtKB=P01877,HUMAN|HGNC=561|UniProtKB=O95782,HUMAN|HGNC=5619|UniProtKB=A0A0C4DH42,HUMAN|HGNC=5596|UniProtKB=P01772,HUMAN|HGNC=5550|UniProtKB=P23083,HUMAN|HGNC=5604|UniProtKB=A0A0B4J1X8,HUMAN|HGNC=570|UniProtKB=P53677,HUMAN|HGNC=2974|UniProtKB=P50570,HUMAN|HGNC=5525|UniProtKB=P01857,HUMAN|HGNC=5662|UniProtKB=A0A0B4J1U7,HUMAN|HGNC=24634|U</td><td>vesicle budding from membrane</td></tr>
</table>

<table>
<tr><td></td><td>niProtKB=Q9UBC2,HUMAN|HGNC=5601|UniProtKB=A0A0C4DH36,HUMAN|HGNC=5716|UniProtKB=P01834,HUMAN|HGNC=5588|UniProtKB=P01764,HUMAN|HGNC=15578|UniProtKB=Q96D71,HUMAN|HGNC=5857|UniProtKB=P0DOY3,HUMAN|HGNC=1736|UniProtKB=P60953,HUMAN|HGNC=5610|UniProtKB=P01767,HUMAN|Ensembl=ENSG00000282150|UniProtKB=A0A0J9YY99,HUMAN|HGNC=29125|UniProtKB=Q9UQ16,HUMAN|HGNC=5527|UniProtKB=P01860,HUMAN|HGNC=2092|UniProtKB=Q00610</td><td></td></tr>
<tr><td>GO:0050830</td><td>HUMAN|HGNC=30013|UniProtKB=Q96PD5,HUMAN|HGNC=2761|UniProtKB=P59665,HUMAN|HGNC=6517|UniProtKB=P18428,HUMAN|HGNC=1472|UniProtKB=P49913,HUMAN|HGNC=6740|UniProtKB=P61626</td><td>defense response to Gram-positive bacterium</td></tr>
<tr><td>GO:0031672</td><td>HUMAN|HGNC=7618|UniProtKB=O14974</td><td>A band</td></tr>
<tr><td>GO:0000045</td><td>HUMAN|HGNC=15912|UniProtKB=Q9UNZ2,HUMAN|HGNC=9758|UniProtKB=P62820,HUMAN|HGNC=18370|UniProtKB=Q9H0U4</td><td>autophagosome assembly</td></tr>
<tr><td>GO:0031305</td><td>HUMAN|HGNC=6047|UniProtKB=Q16891,HUMAN|HGNC=23526|UniProtKB=Q8NE86,HUMAN|HGNC=26801|UniProtKB=Q8N8Q8,HUMAN|HGNC=10989|UniProtKB=Q00325</td><td>integral component of mitochondrial inner membrane</td></tr>
<tr><td>GO:0060589</td><td>HUMAN|HGNC=871|UniProtKB=Q9UII2</td><td>nucleoside-triphosphatase regulator activity</td></tr>
<tr><td>GO:0006351</td><td>HUMAN|HGNC=7803|UniProtKB=Q12986,HUMAN|HGNC=29158|UniProtKB=Q9Y2G9,HUMAN|HGNC=10774|UniProtKB=P23246,HUMAN|HGNC=4719|UniProtKB=P16401,HUMAN|HGNC=27146|UniProtKB=Q5TA31,HUMAN|HGNC=1101|UniProtKB=P51587,HUMAN|HGNC=29890|UniProtKB=Q86UT6,HUMAN|HGNC=4861|UniProtKB=Q9NRZ9,HUMAN|HGNC=28843|UniProtKB=Q9HC44,HUMAN|HGNC=30747|UniProtKB=P61201,HUMAN|HGNC=19246|UniProtKB=Q9NYB0,HUMAN|HGNC=18411|UniProtKB=Q3KNW1,HUMAN|HGNC=12347|UniProtKB=Q9Y4A5,HUMAN|HGNC=25397|UniProtKB=Q6MZP7,HUMAN|HGNC=20508|UniProtKB=Q8IUE6,HUMAN|HGNC=3157|UniProtKB=Q92838,HUMAN|HGNC=7446|UniProtKB=Q9Y4I5,HUMAN|HGNC=20250|UniProtKB=Q96IF1,HUMAN|HGNC=9275|UniProtKB=P35813,HUMAN|HGNC=30140|UniProtKB=A7MCY6,HUMAN|HGNC=16391|UniProtKB=Q9H257,HUMAN|HGNC=16276|UniProtKB=Q9C035,HUMAN|HGNC=4716|UniProtKB=P16403,HUMAN|HGNC=9349|UniProtKB=Q9NQX1,HUMAN|HGNC=4718|UniProtKB=P10412,HUMAN|HGNC=1628|UniProtKB=P08571,HUMAN|HGNC=12738|UniProtKB=Q969T9</td><td>transcription, DNA-templated</td></tr>
<tr><td>GO:0044325</td><td>HUMAN|HGNC=30032|UniProtKB=Q6VY07,HUMAN|HGNC=3702|UniProtKB=Q13642,HUMAN|HGNC=17643|UniProtKB=Q9HD26,HUMAN|HGNC=492|UniProtKB=P16157</td><td>ion channel binding</td></tr>
<tr><td>GO:0004332</td><td>HUMAN|HGNC=8876|UniProtKB=P17858,HUMAN|HGNC=418|UniProtKB=P09972,HUMAN|HGNC=414|UniProtKB=P04075,HUMAN|HGNC=8878|UniProtKB=Q01813</td><td>fructose-bisphosphate aldolase activity</td></tr>
<tr><td>GO:0019439</td><td>HUMAN|HGNC=2689|UniProtKB=P09172,HUMAN|HGNC=82|UniProtKB=P09110</td><td>aromatic compound catabolic process</td></tr>
<tr><td>GO:0006163</td><td>HUMAN|HGNC=362|UniProtKB=P54819,HUMAN|HGNC=363|UniProtKB=P27144,HUMAN|HGNC=361|UniProtKB=P00568,HUMAN|HGNC=18170|UniProtKB=P30085,HUMAN|HGNC=17376|UniProtKB=Q9UIJ7</td><td>purine nucleotide metabolic process</td></tr>
<tr><td>PC00246</td><td>HUMAN|HGNC=3815|UniProtKB=Q12951</td><td>winged helix/forkhead transcription factor</td></tr>
</table>

GO:0030496	HUMAN|HGNC=22931|UniProtKB=Q9P2N7	midbody
GO:0099601	HUMAN|HGNC=2905|UniProtKB=O14490	regulation of neurotransmitter receptor activity
GO:0042073	HUMAN|HGNC=30391|UniProtKB=Q9UG01,HUMAN|HGNC=14313|UniProtKB=Q8WYA0	intraciliary transport
GO:0005783	HUMAN|HGNC=9935|UniProtKB=Q14257,HUMAN|HGNC=12666|UniProtKB=P55072,HUMAN|HGNC=9411|UniProtKB=P14314,HUMAN|HGNC=17943|UniProtKB=Q9GZP9,HUMAN|HGNC=463|UniProtKB=Q9UKV5,HUMAN|HGNC=17059|UniProtKB=Q9NTJ5,HUMAN|HGNC=1458|UniProtKB=O43852,HUMAN|HGNC=13529|UniProtKB=Q9Y2B0,HUMAN|HGNC=16998|UniProtKB=P49755,HUMAN|HGNC=25541|UniProtKB=P57088,HUMAN|HGNC=16695|UniProtKB=P51572,HUMAN|HGNC=10996|UniProtKB=O14975,HUMAN|HGNC=30167|UniProtKB=P13667,HUMAN|HGNC=2479|UniProtKB=O76096,HUMAN|HGNC=16268|UniProtKB=Q8IY17,HUMAN|HGNC=15461|UniProtKB=P55145,HUMAN|HGNC=21354|UniProtKB=Q86XR7,HUMAN|HGNC=24251|UniProtKB=Q9Y3A6,HUMAN|HGNC=8548|UniProtKB=P07237,HUMAN|HGNC=1546|UniProtKB=P50454,HUMAN|HGNC=20175|UniProtKB=Q9Y3E0,HUMAN|HGNC=4606|UniProtKB=P30101,HUMAN|HGNC=8504|UniProtKB=Q969R2,HUMAN|HGNC=15663|UniProtKB=Q9NYU2,HUMAN|HGNC=16996|UniProtKB=Q15363,HUMAN|HGNC=24878|UniProtKB=Q9BVK6	endoplasmic reticulum
GO:0090501	HUMAN|HGNC=8087|UniProtKB=P29728	RNA phosphodiester bond hydrolysis
GO:0071383	HUMAN|HGNC=533|UniProtKB=P04083	cellular response to steroid hormone stimulus
GO:0061512	HUMAN|HGNC=15476|UniProtKB=P63167,HUMAN|HGNC=15848|UniProtKB=Q14141,HUMAN|HGNC=16053|UniProtKB=Q8N1V2,HUMAN|HGNC=20908|UniProtKB=Q86YF9,HUMAN|HGNC=2952|UniProtKB=Q96JB1,HUMAN|HGNC=25631|UniProtKB=Q96MT7,HUMAN|HGNC=1717|UniProtKB=Q16181,HUMAN|HGNC=3244|UniProtKB=Q9NZN3,HUMAN|HGNC=29262|UniProtKB=Q9P2H3,HUMAN|HGNC=7729|UniProtKB=Q15019	protein localization to cilium
GO:0008652	HUMAN|HGNC=746|UniProtKB=P04424	cellular amino acid biosynthetic process
GO:0043679	HUMAN|HGNC=11138|UniProtKB=P37840	axon terminus
GO:0005912	HUMAN|HGNC=16051|UniProtKB=Q8TEW0,HUMAN|HGNC=2067|UniProtKB=O00151,HUMAN|HGNC=15902|UniProtKB=Q8IXH8,HUMAN|HGNC=17468|UniProtKB=Q96HC4,HUMAN|HGNC=243|UniProtKB=P35611,HUMAN|HGNC=6207|UniProtKB=P14923,HUMAN|HGNC=14265|UniProtKB=Q86UP0,HUMAN|HGNC=19194|UniProtKB=Q96RW7,HUMAN|HGNC=22958|UniProtKB=Q9NR12,HUMAN|HGNC=20250|UniProtKB=Q96IF1,HUMAN|HGNC=30377|UniProtKB=Q14160	adherens junction
GO:0007009	HUMAN|HGNC=544|UniProtKB=P08133,HUMAN|HGNC=8515|UniProtKB=Q9HC10	plasma membrane organization
GO:0006893	HUMAN|HGNC=30389|UniProtKB=Q96A65	Golgi to plasma membrane transport
GO:0009888	HUMAN|HGNC=12601|UniProtKB=O75445,HUMAN|HGNC=885|UniProtKB=O75882	tissue development

<table>
<tr><td>GO:0018279</td><td>HUMAN|HGNC=10381|UniProtKB=P04843,HUMAN|HGNC=2728|UniProtKB=P39656,HUMAN|HGNC=15663|UniProtKB=Q9NYU2</td><td>protein N-linked glycosylation via asparagine</td></tr>
<tr><td>GO:0006928</td><td>HUMAN|HGNC=12736|UniProtKB=O43516</td><td>movement of cell or subcellular component</td></tr>
<tr><td>GO:1901214</td><td>HUMAN|HGNC=11138|UniProtKB=P37840</td><td>regulation of neuron death</td></tr>
<tr><td>GO:0045104</td><td>HUMAN|HGNC=1090|UniProtKB=Q03001,HUMAN|HGNC=9069|UniProtKB=Q15149</td><td>intermediate filament cytoskeleton organization</td></tr>
<tr><td>GO:0005509</td><td>HUMAN|HGNC=537|UniProtKB=P07355,HUMAN|HGNC=8044|UniProtKB=P80303,HUMAN|HGNC=12022|UniProtKB=P13693,HUMAN|HGNC=9935|UniProtKB=Q14257,HUMAN|HGNC=545|UniProtKB=P20073,HUMAN|HGNC=3048|UniProtKB=Q02413,HUMAN|HGNC=1455|UniProtKB=P27797,HUMAN|HGNC=533|UniProtKB=P04083,HUMAN|HGNC=1449|UniProtKB=P0DP25,HUMAN|HGNC=1473|UniProtKB=P27824,HUMAN|HGNC=11219|UniProtKB=P09486,HUMAN|HGNC=535|UniProtKB=P50995,HUMAN|HGNC=15902|UniProtKB=Q8IXH8,HUMAN|HGNC=1458|UniProtKB=O43852,HUMAN|HGNC=11510|UniProtKB=Q8N9I0,HUMAN|HGNC=544|UniProtKB=P08133,HUMAN|HGNC=14265|UniProtKB=Q86UP0,HUMAN|HGNC=1452|UniProtKB=P27482,HUMAN|HGNC=11512|UniProtKB=Q9H2B2,HUMAN|HGNC=8515|UniProtKB=Q9HC10,HUMAN|HGNC=8043|UniProtKB=Q02818,HUMAN|HGNC=543|UniProtKB=P08758</td><td>calcium ion binding</td></tr>
<tr><td>GO:0007200</td><td>HUMAN|HGNC=3537|UniProtKB=P25116,HUMAN|HGNC=4472|UniProtKB=Q14330,HUMAN|HGNC=4390|UniProtKB=P50148,HUMAN|HGNC=3540|UniProtKB=Q96RI0</td><td>phospholipase C-activating G protein-coupled receptor signaling pathway</td></tr>
<tr><td>GO:0005779</td><td>HUMAN|HGNC=21689|UniProtKB=Q9Y3D6</td><td>integral component of peroxisomal membrane</td></tr>
<tr><td>GO:0015909</td><td>HUMAN|HGNC=533|UniProtKB=P04083</td><td>long-chain fatty acid transport</td></tr>
<tr><td>GO:0048259</td><td>HUMAN|HGNC=3757|UniProtKB=O75955</td><td>regulation of receptor-mediated endocytosis</td></tr>
<tr><td>GO:0034765</td><td>HUMAN|HGNC=16804|UniProtKB=O00308</td><td>regulation of ion transmembrane transport</td></tr>
<tr><td>GO:0044275</td><td>HUMAN|HGNC=24552|UniProtKB=Q3LXA3,HUMAN|HGNC=12009|UniProtKB=P60174,HUMAN|HGNC=9723|UniProtKB=P11216,HUMAN|HGNC=8891|UniProtKB=P52209,HUMAN|HGNC=9725|UniProtKB=P06737</td><td>cellular carbohydrate catabolic process</td></tr>
<tr><td>GO:0010629</td><td>HUMAN|HGNC=2479|UniProtKB=O76096,HUMAN|HGNC=9583|UniProtKB=P26599</td><td>negative regulation of gene expression</td></tr>
<tr><td>GO:0042470</td><td>HUMAN|HGNC=9772|UniProtKB=Q13637,HUMAN|HGNC=9767|UniProtKB=O00194</td><td>melanosome</td></tr>
<tr><td>GO:0002682</td><td>HUMAN|HGNC=8993|UniProtKB=P12273</td><td>regulation of immune system process</td></tr>
<tr><td>GO:0003676</td><td>HUMAN|HGNC=14959|UniProtKB=Q96T37</td><td>nucleic acid binding</td></tr>
<tr><td>GO:0019899</td><td>HUMAN|HGNC=20040|UniProtKB=Q01518,HUMAN|HGNC=871|UniProtKB=Q9UII2</td><td>enzyme binding</td></tr>
<tr><td>PC00181</td><td>HUMAN|HGNC=25364|UniProtKB=Q9H0R4,HUMAN|HGNC=17059|UniProtKB=Q9NTJ5,HUMAN|HGNC=6050|UniProtKB=P29218,HUMAN|HGNC=10542|UniProtKB=O95248</td><td>phosphatase</td></tr>
<tr><td>GO:0006405</td><td>HUMAN|HGNC=8062|UniProtKB=P49790,HUMAN|HGNC=17859|UniProtKB=Q5SRE5</td><td>RNA export from nucleus</td></tr>
<tr><td>GO:0000902</td><td>HUMAN|HGNC=18756|UniProtKB=Q9BYZ6,HUMAN|HGNC=20040|UniProtKB=Q01518,HUMAN|HGNC=13759|UniProtKB=Q7L576,H</td><td>cell morphogenesis</td></tr>
</table>

<table>
<tr><td></td><td>UMAN|HGNC=15902|UniProtKB=Q8IXH8,HUMAN|HGNC=533|UniProtKB=P04083,HUMAN|HGNC=14265|UniProtKB=Q86UP0,HUMAN|HGNC=1491|UniProtKB=P47756</td><td></td></tr>
<tr><td>GO:0005688</td><td>HUMAN|HGNC=20471|UniProtKB=O95777,HUMAN|HGNC=17259|UniProtKB=Q9Y4Z0</td><td>U6 snRNP</td></tr>
<tr><td>GO:0042327</td><td>HUMAN|HGNC=8898|UniProtKB=P07205</td><td>positive regulation of phosphorylation</td></tr>
<tr><td>GO:0019773</td><td>HUMAN|HGNC=9535|UniProtKB=P60900,HUMAN|HGNC=9533|UniProtKB=P25789,HUMAN|HGNC=9536|UniProtKB=O14818,HUMAN|HGNC=9530|UniProtKB=P25786,HUMAN|HGNC=9532|UniProtKB=P25788,HUMAN|HGNC=9531|UniProtKB=P25787</td><td>proteasome core complex, alpha-subunit complex</td></tr>
<tr><td>GO:0006298</td><td>HUMAN|HGNC=7127|UniProtKB=P40692,HUMAN|HGNC=7326|UniProtKB=P20585</td><td>mismatch repair</td></tr>
<tr><td>GO:0044389</td><td>HUMAN|HGNC=11124|UniProtKB=P55854,HUMAN|HGNC=11125|UniProtKB=P61956</td><td>ubiquitin-like protein ligase binding</td></tr>
<tr><td>GO:0033522</td><td>HUMAN|HGNC=23077|UniProtKB=Q96FW1</td><td>histone H2A ubiquitination</td></tr>
<tr><td>GO:0006955</td><td>HUMAN|HGNC=5753|UniProtKB=P01593,HUMAN|HGNC=5879|UniProtKB=P01699,HUMAN|HGNC=5825|UniProtKB=A0A0C4DH25,HUMAN|HGNC=5800|UniProtKB=A0A075B6S2,HUMAN|HGNC=5742|UniProtKB=A0A0C4DH72,HUMAN|HGNC=5816|UniProtKB=P01624,HUMAN|Gene=KV311_HUMAN|UniProtKB=P04433,HUMAN|HGNC=2444|UniProtKB=P41240,HUMAN|HGNC=5910|UniProtKB=P01718,HUMAN|HGNC=5931|UniProtKB=A0A075B6I0,HUMAN|HGNC=5933|UniProtKB=A0A0B4J1Y8,HUMAN|HGNC=5877|UniProtKB=P01703,HUMAN|HGNC=5834|UniProtKB=P06312,HUMAN|HGNC=5890|UniProtKB=P01705,HUMAN|HGNC=5732|UniProtKB=P04430,HUMAN|HGNC=5759|UniProtKB=A0A087WSZ0,HUMAN|HGNC=5733|UniProtKB=P01599,HUMAN|HGNC=5888|UniProtKB=P01704,HUMAN|HGNC=3309|UniProtKB=P08246,HUMAN|HGNC=5927|UniProtKB=P01721,HUMAN|HGNC=5897|UniProtKB=A0A075B6K4,HUMAN|HGNC=5918|UniProtKB=A0A075B6K5,HUMAN|HGNC=5781|UniProtKB=A0A0C4DH68,HUMAN|HGNC=5930|UniProtKB=A0A075B6I9,HUMAN|HGNC=5901|UniProtKB=A0A075B6K0,HUMAN|HGNC=5743|UniProtKB=A0A0C4DH67,HUMAN|HGNC=5739|UniProtKB=A0A075B6S9,HUMAN|HGNC=5880|UniProtKB=P01700,HUMAN|HGNC=5905|UniProtKB=P80748,HUMAN|HGNC=5756|UniProtKB=P04432,HUMAN|HGNC=5896|UniProtKB=P01715,HUMAN|HGNC=910|UniProtKB=P25311,HUMAN|HGNC=5735|UniProtKB=A0A075B6S5,HUMAN|HGNC=5785|UniProtKB=P06310,HUMAN|HGNC=5836|UniProtKB=A0A0C4DH24,HUMAN|HGNC=5895|UniProtKB=P01709,HUMAN|HGNC=5898|UniProtKB=A0A075B6K2,HUMAN|HGNC=5887|UniProtKB=P01706,HUMAN|HGNC=5929|UniProtKB=P04211,HUMAN|HGNC=5882|UniProtKB=P01701,HUMAN|HGNC=5908|UniProtKB=P01717,HUMAN|HGNC=5746|UniProtKB=P01611,HUMAN|HGNC=5821|UniProtKB=A0A075B6H7,HUMAN|HGNC=4036|UniProtKB=O15117,HUMAN|HGNC=5889|UniProtKB=A0A075B6J9,HUMAN|HGNC=5741|UniProtKB=P01602,HUMAN|HGNC=5730|UniProtKB=A0A0C4DH73,HUMAN|HGNC=5817|UniProtKB=P01619,HUMAN|HGNC=5783|UniProtKB=A0A075B6P5</td><td>immune response</td></tr>
<tr><td>GO:0010646</td><td>HUMAN|HGNC=11510|UniProtKB=Q8N9I0,HUMAN|HGNC=11512|UniProtKB=Q9H2B2</td><td>regulation of cell communication</td></tr>
<tr><td>GO:0043491</td><td>HUMAN|HGNC=18233|UniProtKB=Q8NDB2,HUMAN|HGNC=6091|UniProtKB=P06213</td><td>protein kinase B signaling</td></tr>
</table>

GO:0003682	HUMAN|HGNC=5048|UniProtKB=Q00839,HUMAN|HGNC=10760|UniProtKB=Q01105,HUMAN|HGNC=7640|UniProtKB=Q99733,HUMAN|HGNC=4861|UniProtKB=Q9NRZ9,HUMAN|HGNC=42937|UniProtKB=P0DME0,HUMAN|HGNC=7910|UniProtKB=P06748,HUMAN|HGNC=24358|UniProtKB=Q9H2G4	chromatin binding
GO:0021915	HUMAN|HGNC=12652|UniProtKB=P50552	neural tube development
GO:0004180	HUMAN|HGNC=25648|UniProtKB=Q8N5J2,HUMAN|HGNC=15722|UniProtKB=Q8WXQ8,HUMAN|HGNC=2300|UniProtKB=Q96IY4,HUMAN|HGNC=11763|UniProtKB=P02786,HUMAN|HGNC=2312|UniProtKB=P15169	carboxypeptidase activity
GO:0022625	HUMAN|HGNC=10372|UniProtKB=P05386,HUMAN|HGNC=10363|UniProtKB=P18124,HUMAN|HGNC=10305|UniProtKB=P50914,HUMAN|HGNC=10353|UniProtKB=P36578	cytosolic large ribosomal subunit
PC00031	HUMAN|HGNC=8648|UniProtKB=Q15366,HUMAN|HGNC=19071|UniProtKB=Q86V81,HUMAN|HGNC=29190|UniProtKB=Q9UPQ9,HUMAN|HGNC=14959|UniProtKB=Q96T37,HUMAN|HGNC=10774|UniProtKB=P23246,HUMAN|HGNC=17150|UniProtKB=Q9Y2V2,HUMAN|HGNC=117|UniProtKB=P21399,HUMAN|HGNC=29531|UniProtKB=Q6PKG0,HUMAN|HGNC=8647|UniProtKB=Q15365,HUMAN|HGNC=226|UniProtKB=P78563,HUMAN|HGNC=5044|UniProtKB=P61978,HUMAN|HGNC=6038|UniProtKB=Q12906,HUMAN|HGNC=17860|UniProtKB=Q8NC51,HUMAN|HGNC=24903|UniProtKB=Q9Y520,HUMAN|HGNC=19072|UniProtKB=Q96J01	RNA metabolism protein
GO:0016324	HUMAN|HGNC=16051|UniProtKB=Q8TEW0,HUMAN|HGNC=29327|UniProtKB=Q5VZ46,HUMAN|HGNC=29953|UniProtKB=Q8WWZ8,HUMAN|HGNC=11075|UniProtKB=O14745	apical plasma membrane
PC00241	HUMAN|HGNC=1406|UniProtKB=Q9Y698,HUMAN|HGNC=12669|UniProtKB=P21796,HUMAN|HGNC=12674|UniProtKB=Q9Y277,HUMAN|HGNC=12672|UniProtKB=P45880,HUMAN|HGNC=10594|UniProtKB=Q01118,HUMAN|HGNC=10582|UniProtKB=Q9Y5Y9,HUMAN|HGNC=1393|UniProtKB=O60840	voltage-gated ion channel
GO:0016887	HUMAN|HGNC=800|UniProtKB=P50993,HUMAN|HGNC=2949|UniProtKB=Q8TD57,HUMAN|HGNC=7127|UniProtKB=P40692,HUMAN|HGNC=5239|UniProtKB=P17066,HUMAN|HGNC=12666|UniProtKB=P55072,HUMAN|HGNC=813|UniProtKB=Q93084,HUMAN|HGNC=14637|UniProtKB=Q86UK0,HUMAN|HGNC=823|UniProtKB=P25705,HUMAN|HGNC=8016|UniProtKB=P46459,HUMAN|HGNC=5241|UniProtKB=P11142,HUMAN|HGNC=2951|UniProtKB=Q9C0G6,HUMAN|HGNC=2952|UniProtKB=Q96JB1,HUMAN|HGNC=871|UniProtKB=Q9UII2,HUMAN|HGNC=55|UniProtKB=O15439,HUMAN|HGNC=5238|UniProtKB=P11021,HUMAN|HGNC=5240|UniProtKB=P48741,HUMAN|HGNC=851|UniProtKB=P38606,HUMAN|HGNC=806|UniProtKB=P54709,HUMAN|HGNC=13531|UniProtKB=Q9Y2Q0,HUMAN|HGNC=830|UniProtKB=P06576,HUMAN|HGNC=812|UniProtKB=P16615	ATPase activity
GO:0007399	HUMAN|HGNC=3778|UniProtKB=P02751,HUMAN|HGNC=4243|UniProtKB=P56159,HUMAN|HGNC=4244|UniProtKB=O00451	nervous system development
GO:0070971	HUMAN|HGNC=10534|UniProtKB=Q9NR31,HUMAN|HGNC=10701|UniProtKB=Q15436	endoplasmic reticulum exit site
GO:0140030	HUMAN|HGNC=20635|UniProtKB=Q14997	modification-dependent protein binding

GO:0005793	HUMAN|HGNC=8044|UniProtKB=P80303,HUMAN|HGNC=21354|UniProtKB=Q86XR7,HUMAN|HGNC=24251|UniProtKB=Q9Y3A6,HUMAN|HGNC=6631|UniProtKB=P49257,HUMAN|HGNC=16998|UniProtKB=P49755,HUMAN|HGNC=16996|UniProtKB=Q15363,HUMAN|HGNC=8043|UniProtKB=Q02818,HUMAN|HGNC=24878|UniProtKB=Q9BVK6	endoplasmic reticulum-Golgi intermediate compartment
GO:0017134	HUMAN|HGNC=15527|UniProtKB=Q86Z14	fibroblast growth factor binding
GO:0051536	HUMAN|HGNC=3951|UniProtKB=Q16595	iron-sulfur cluster binding
GO:0044877	HUMAN|HGNC=4395|UniProtKB=P19086,HUMAN|HGNC=11424|UniProtKB=O43815,HUMAN|HGNC=4384|UniProtKB=P63096,HUMAN|HGNC=12733|UniProtKB=Q9Y6W5,HUMAN|HGNC=4387|UniProtKB=P08754,HUMAN|HGNC=4374|UniProtKB=O60234,HUMAN|HGNC=4385|UniProtKB=P04899,HUMAN|HGNC=4390|UniProtKB=P50148,HUMAN|HGNC=492|UniProtKB=P16157,HUMAN|HGNC=15720|UniProtKB=Q13033,HUMAN|HGNC=4381|UniProtKB=Q14344,HUMAN|HGNC=4393|UniProtKB=P11488,HUMAN|HGNC=8515|UniProtKB=Q9HC10	protein-containing complex binding
GO:0019955	HUMAN|HGNC=1681|UniProtKB=P16070,HUMAN|HGNC=19100|UniProtKB=Q5VWK5	cytokine binding
GO:0004623	HUMAN|HGNC=533|UniProtKB=P04083	phospholipase A2 activity
PC00179	HUMAN|HGNC=3964|UniProtKB=P01225,HUMAN|HGNC=16452|UniProtKB=P0DN86,HUMAN|HGNC=16721|UniProtKB=A6NKQ9	peptide hormone
GO:0034622	HUMAN|HGNC=7641|UniProtKB=P54920,HUMAN|HGNC=4926|UniProtKB=O15212,HUMAN|HGNC=12644|UniProtKB=Q15836,HUMAN|HGNC=11582|UniProtKB=Q15813,HUMAN|HGNC=11131|UniProtKB=O00161,HUMAN|HGNC=12647|UniProtKB=Q9BV40,HUMAN|HGNC=2092|UniProtKB=Q00610,HUMAN|HGNC=2288|UniProtKB=P14406,HUMAN|HGNC=2090|UniProtKB=P09496,HUMAN|HGNC=8515|UniProtKB=Q9HC10,HUMAN|HGNC=11579|UniProtKB=O75347	cellular protein-containing complex assembly
GO:0005938	HUMAN|HGNC=16051|UniProtKB=Q8TEW0,HUMAN|HGNC=15848|UniProtKB=Q14141,HUMAN|HGNC=18756|UniProtKB=Q9BYZ6,HUMAN|HGNC=9802|UniProtKB=P15153,HUMAN|HGNC=9164|UniProtKB=Q99719,HUMAN|HGNC=1717|UniProtKB=Q16181,HUMAN|HGNC=29327|UniProtKB=Q5VZ46,HUMAN|HGNC=9801|UniProtKB=P63000,HUMAN|HGNC=2090|UniProtKB=P09496,HUMAN|HGNC=7729|UniProtKB=Q15019	cell cortex
GO:0006206	HUMAN|HGNC=2520|UniProtKB=Q9NRF8	pyrimidine nucleobase metabolic process
PC00197	HUMAN|HGNC=4045|UniProtKB=O75084,HUMAN|HGNC=4439|UniProtKB=P07359,HUMAN|HGNC=7821|UniProtKB=P14543,HUMAN|HGNC=5468|UniProtKB=P35858,HUMAN|HGNC=19100|UniProtKB=Q5VWK5,HUMAN|HGNC=4244|UniProtKB=O00451,HUMAN|HGNC=29953|UniProtKB=Q8WWZ8,HUMAN|HGNC=11119|UniProtKB=Q99835,HUMAN|HGNC=16089|UniProtKB=O15173,HUMAN|HGNC=1681|UniProtKB=P16070,HUMAN|HGNC=4243|UniProtKB=P56159,HUMAN|HGNC=10661|UniProtKB=P31431,HUMAN|HGNC=10608|UniProtKB=P47872,HUMAN|HGNC=13937|UniProtKB=O95866,HUMAN|HGNC=19296|UniProtKB=Q9UF33,HUMAN|HGNC=16090|UniProtKB=O00264,HUMAN|HGNC=1628|UniProtKB=P08571,HUMAN|HGNC=16768|UniProtKB=O60391,HUMAN|HGNC=2313	transmembrane signal receptor

<table>
<tr><td></td><td>|UniProtKB=P22792,HUMAN|HGNC=30384|UniProtKB=Q9UMX5,HUMAN|HGNC=6091|UniProtKB=P06213</td><td></td></tr>
<tr><td>GO:0016042</td><td>HUMAN|HGNC=607|UniProtKB=P02654,HUMAN|HGNC=108|UniProtKB=P22303</td><td>lipid catabolic process</td></tr>
<tr><td>GO:0006469</td><td>HUMAN|HGNC=19388|UniProtKB=P49593,HUMAN|HGNC=29884|UniProtKB=Q8TF42</td><td>negative regulation of protein kinase activity</td></tr>
<tr><td>GO:0000398</td><td>HUMAN|HGNC=20471|UniProtKB=O95777,HUMAN|HGNC=17821|UniProtKB=O00148,HUMAN|HGNC=9583|UniProtKB=P26599,HUMAN|HGNC=14959|UniProtKB=Q96T37,HUMAN|HGNC=5044|UniProtKB=P61978,HUMAN|HGNC=1743|UniProtKB=Q99459,HUMAN|HGNC=10774|UniProtKB=P23246,HUMAN|HGNC=24932|UniProtKB=Q9NZ63</td><td>mRNA splicing, via spliceosome</td></tr>
<tr><td>GO:0009653</td><td>HUMAN|HGNC=6767|UniProtKB=Q15797,HUMAN|HGNC=3815|UniProtKB=Q12951</td><td>anatomical structure morphogenesis</td></tr>
<tr><td>GO:0046540</td><td>HUMAN|HGNC=20471|UniProtKB=O95777</td><td>U4/U6 x U5 tri-snRNP complex</td></tr>
<tr><td>GO:0030100</td><td>HUMAN|HGNC=9782|UniProtKB=P61018,HUMAN|HGNC=9785|UniProtKB=P51148,HUMAN|HGNC=9784|UniProtKB=P61020,HUMAN|HGNC=19760|UniProtKB=Q96T51</td><td>regulation of endocytosis</td></tr>
<tr><td>GO:0051606</td><td>HUMAN|HGNC=30013|UniProtKB=Q96PD5</td><td>detection of stimulus</td></tr>
<tr><td>GO:0034551</td><td>HUMAN|HGNC=12582|UniProtKB=P14927</td><td>mitochondrial respiratory chain complex III assembly</td></tr>
<tr><td>GO:0070491</td><td>HUMAN|HGNC=9349|UniProtKB=Q9NQX1</td><td>repressing transcription factor binding</td></tr>
<tr><td>GO:0009636</td><td>HUMAN|HGNC=253|UniProtKB=P11766,HUMAN|HGNC=11179|UniProtKB=P00441,HUMAN|HGNC=9353|UniProtKB=P32119,HUMAN|HGNC=251|UniProtKB=P00326,HUMAN|HGNC=9352|UniProtKB=Q06830,HUMAN|HGNC=255|UniProtKB=P28332</td><td>response to toxic substance</td></tr>
<tr><td>GO:0017116</td><td>HUMAN|HGNC=17196|UniProtKB=Q8NG08</td><td>single-stranded DNA helicase activity</td></tr>
<tr><td>GO:0048814</td><td>HUMAN|HGNC=3338|UniProtKB=Q14247,HUMAN|HGNC=2695|UniProtKB=Q16643,HUMAN|HGNC=2696|UniProtKB=Q9UJU6,HUMAN|HGNC=4844|UniProtKB=P14317</td><td>regulation of dendrite morphogenesis</td></tr>
<tr><td>GO:0016705</td><td>HUMAN|HGNC=2689|UniProtKB=P09172</td><td>oxidoreductase activity, acting on paired donors, with incorporation or reduction of molecular oxygen</td></tr>
<tr><td>GO:0002460</td><td>HUMAN|HGNC=5575|UniProtKB=A0A0B4J1V2,HUMAN|HGNC=5645|UniProtKB=A0A0C4DH34,HUMAN|HGNC=5549|UniProtKB=A0A0C4DH31,HUMAN|HGNC=5554|UniProtKB=P01743,HUMAN|HGNC=5480|UniProtKB=P01880,HUMAN|HGNC=5659|UniProtKB=A0A0C4DH38,HUMAN|HGNC=5634|UniProtKB=A0A075B7F0,HUMAN|HGNC=38476|UniProtKB=B9A064,HUMAN|HGNC=5861|UniProtKB=A0M8Q6,HUMAN|HGNC=5647|UniProtKB=A0A087WSY4,HUMAN|HGNC=5563|UniProtKB=A0A075B7D0,HUMAN|HGNC=5856|UniProtKB=P0DOY2,HUMAN|HGNC=2367|UniProtKB=P02741,HUMAN|HGNC=5606|UniProtKB=P01763,HUMAN|HGNC=533|UniProtKB=P04083,HUMAN|HGNC=5576|UniProtKB=P01817,HUMAN|HGNC=5528|UniProtKB=P01861,HUMAN|HGNC=5541|UniProtKB=P01871,HUMAN|HGNC=5585|UniProtKB=A0A0C4DH32,HUMAN|HGNC=5551|UniProtKB=A0A0C4DH33,HUMAN|HGNC=5526|UniProtKB=P01859,HUMAN|HGNC=5657|UniProtKB=P0DP08,HUMAN|Ensembl=</td><td>adaptive immune response based on somatic recombination of immune receptors built from immunoglobulin superfamily domains</td></tr>
</table>

<table>
<tr><td></td><td>ENSG00000282639|UniProtKB=A0A0J9YX35,HUMAN|Gene=HV309_HUMAN|UniProtKB=P01782,HUMAN|HGNC=5654|UniProtKB=P01825,HUMAN|HGNC=5553|UniProtKB=A0A0A0MS14,HUMAN|Gene=HV108_HUMAN|UniProtKB=P0DP01,HUMAN|HGNC=5624|UniProtKB=A0A0B4J1X5,HUMAN|HGNC=5620|UniProtKB=P01780,HUMAN|HGNC=5598|UniProtKB=A0A0C4DH35,HUMAN|HGNC=5580|UniProtKB=P01762,HUMAN|HGNC=5633|UniProtKB=A0A075B7D8,HUMAN|HGNC=5582|UniProtKB=A0A0B4J1V0,HUMAN|HGNC=5478|UniProtKB=P01876,HUMAN|HGNC=5617|UniProtKB=A0A075B6Q5,HUMAN|HGNC=5594|UniProtKB=P0DP03,HUMAN|HGNC=5655|UniProtKB=A0A0C4DH41,HUMAN|HGNC=5636|UniProtKB=A0A075B7B8,HUMAN|HGNC=5586|UniProtKB=A0A0B4J1V1,HUMAN|HGNC=5581|UniProtKB=P01766,HUMAN|HGNC=5622|UniProtKB=A0A0B4J1Y9,HUMAN|HGNC=5652|UniProtKB=A0A075B6R2,HUMAN|HGNC=5593|UniProtKB=P0DP02,HUMAN|HGNC=5479|UniProtKB=P01877,HUMAN|HGNC=5619|UniProtKB=A0A0C4DH42,HUMAN|HGNC=5596|UniProtKB=P01772,HUMAN|HGNC=5550|UniProtKB=P23083,HUMAN|HGNC=5604|UniProtKB=A0A0B4J1X8,HUMAN|HGNC=5525|UniProtKB=P01857,HUMAN|HGNC=5662|UniProtKB=A0A0B4J1U7,HUMAN|HGNC=16961|UniProtKB=Q15762,HUMAN|HGNC=5601|UniProtKB=A0A0C4DH36,HUMAN|HGNC=5716|UniProtKB=P01834,HUMAN|HGNC=5588|UniProtKB=P01764,HUMAN|HGNC=5857|UniProtKB=P0DOY3,HUMAN|HGNC=5610|UniProtKB=P01767,HUMAN|Ensembl=ENSG00000282150|UniProtKB=A0A0J9YY99,HUMAN|HGNC=5527|UniProtKB=P01860,HUMAN|HGNC=5584|UniProtKB=P02743</td><td></td></tr>
<tr><td>GO:0006164</td><td>HUMAN|HGNC=9465|UniProtKB=P11908,HUMAN|HGNC=9462|UniProtKB=P60891</td><td>purine nucleotide biosynthetic process</td></tr>
<tr><td>GO:0043328</td><td>HUMAN|HGNC=11358|UniProtKB=O75886,HUMAN|HGNC=11357|UniProtKB=Q92783</td><td>protein transport to vacuole involved in ubiquitin-dependent protein catabolic process via the multivesicular body sorting pathway</td></tr>
<tr><td>GO:0031175</td><td>HUMAN|HGNC=9621|UniProtKB=Q6IBS0,HUMAN|HGNC=15461|UniProtKB=P55145,HUMAN|HGNC=11590|UniProtKB=Q16650,HUMAN|HGNC=6510|UniProtKB=P16949,HUMAN|HGNC=12648|UniProtKB=Q9P0L0</td><td>neuron projection development</td></tr>
<tr><td>GO:1902808</td><td>HUMAN|HGNC=533|UniProtKB=P04083</td><td>positive regulation of cell cycle G1/S phase transition</td></tr>
<tr><td>GO:0051209</td><td>HUMAN|HGNC=29884|UniProtKB=Q8TF42</td><td>release of sequestered calcium ion into cytosol</td></tr>
<tr><td>PC00258</td><td>HUMAN|HGNC=11030|UniProtKB=Q9Y6R1,HUMAN|HGNC=10996|UniProtKB=O14975,HUMAN|HGNC=11071|UniProtKB=P19634,HUMAN|HGNC=11027|UniProtKB=P02730,HUMAN|HGNC=17292|UniProtKB=Q8IWA5,HUMAN|HGNC=20653|UniProtKB=Q8IVB4,HUMAN|HGNC=11034|UniProtKB=Q2Y0W8</td><td>secondary carrier transporter</td></tr>
<tr><td>GO:0031461</td><td>HUMAN|HGNC=2551|UniProtKB=Q13616,HUMAN|HGNC=2717|UniProtKB=Q16531</td><td>cullin-RING ubiquitin ligase complex</td></tr>
<tr><td>GO:0004386</td><td>HUMAN|HGNC=10898|UniProtKB=Q15477,HUMAN|HGNC=17821|UniProtKB=O00148</td><td>helicase activity</td></tr>
<tr><td>GO:0048741</td><td>HUMAN|HGNC=15999|UniProtKB=Q9NZV5</td><td>skeletal muscle fiber development</td></tr>
</table>

GO:0005884	HUMAN|HGNC=6528|UniProtKB=P13796,HUMAN|HGNC=2067|UniProtKB=O00151,HUMAN|HGNC=23397|UniProtKB=Q5T1M5,HUMAN|HGNC=11148|UniProtKB=Q16658,HUMAN|HGNC=2695|UniProtKB=Q16643,HUMAN|HGNC=13200|UniProtKB=Q15942,HUMAN|HGNC=2696|UniProtKB=Q9UJU6,HUMAN|HGNC=18304|UniProtKB=Q14019,HUMAN|HGNC=3338|UniProtKB=Q14247,HUMAN|HGNC=12010|UniProtKB=P09493,HUMAN|HGNC=9581|UniProtKB=Q9H939,HUMAN|HGNC=12736|UniProtKB=O43516,HUMAN|HGNC=9621|UniProtKB=Q6IBS0,HUMAN|HGNC=12012|UniProtKB=P06753,HUMAN|HGNC=7590|UniProtKB=Q15746,HUMAN|HGNC=18142|UniProtKB=Q9Y4D1,HUMAN|HGNC=17468|UniProtKB=Q96HC4,HUMAN|HGNC=4381|UniProtKB=Q14344,HUMAN|HGNC=12013|UniProtKB=P67936,HUMAN|HGNC=12011|UniProtKB=P07951,HUMAN|HGNC=22958|UniProtKB=Q9NR12,HUMAN|HGNC=6040|UniProtKB=Q13418,HUMAN|HGNC=4844|UniProtKB=P14317,HUMAN|HGNC=11126|UniProtKB=P53814	actin filament
GO:0034599	HUMAN|HGNC=11179|UniProtKB=P00441,HUMAN|HGNC=9355|UniProtKB=P30044,HUMAN|HGNC=9353|UniProtKB=P32119,HUMAN|HGNC=533|UniProtKB=P04083,HUMAN|HGNC=9354|UniProtKB=P30048,HUMAN|HGNC=9352|UniProtKB=Q06830,HUMAN|HGNC=4623|UniProtKB=P00390,HUMAN|HGNC=7377|UniProtKB=Q9UJ68	cellular response to oxidative stress
PC00038	HUMAN|HGNC=30244|UniProtKB=Q6P1A2,HUMAN|HGNC=82|UniProtKB=P09110	acetyltransferase
PC00186	HUMAN|HGNC=4459|UniProtKB=P80108,HUMAN|HGNC=6737|UniProtKB=O75608,HUMAN|HGNC=9065|UniProtKB=P19174,HUMAN|HGNC=17038|UniProtKB=Q99685,HUMAN|HGNC=9055|UniProtKB=Q00722	phospholipase
GO:0015078	HUMAN|HGNC=7687|UniProtKB=O00483,HUMAN|HGNC=7421|UniProtKB=P00403,HUMAN|HGNC=813|UniProtKB=Q93084,HUMAN|HGNC=851|UniProtKB=P38606,HUMAN|HGNC=2267|UniProtKB=P20674,HUMAN|HGNC=830|UniProtKB=P06576,HUMAN|HGNC=850|UniProtKB=P48047,HUMAN|HGNC=812|UniProtKB=P16615	proton transmembrane transporter activity
PC00120	HUMAN|HGNC=118|UniProtKB=Q99798,HUMAN|HGNC=3149|UniProtKB=Q13011,HUMAN|HGNC=3151|UniProtKB=P30084	hydratase
GO:0005887	HUMAN|HGNC=8710|UniProtKB=Q9Y5G1,HUMAN|HGNC=1630|UniProtKB=P48509,HUMAN|HGNC=6156|UniProtKB=P05106,HUMAN|HGNC=3616|UniProtKB=P12318,HUMAN|HGNC=8823|UniProtKB=P16284,HUMAN|HGNC=3537|UniProtKB=P25116,HUMAN|HGNC=19100|UniProtKB=Q5VWK5,HUMAN|HGNC=6153|UniProtKB=P05556,HUMAN|HGNC=14674|UniProtKB=Q96QU1,HUMAN|HGNC=4472|UniProtKB=Q14330,HUMAN|HGNC=3540|UniProtKB=Q96RI0,HUMAN|HGNC=1709|UniProtKB=P21926,HUMAN|HGNC=3338|UniProtKB=Q14247,HUMAN|HGNC=1692|UniProtKB=P08962,HUMAN|HGNC=19296|UniProtKB=Q9UF33,HUMAN|HGNC=806|UniProtKB=P54709,HUMAN|HGNC=10594|UniProtKB=Q01118,HUMAN|HGNC=10582|UniProtKB=Q9Y5Y9,HUMAN|HGNC=6091|UniProtKB=P06213,HUMAN|HGNC=237|UniProtKB=O43306	integral component of plasma membrane
GO:0016874	HUMAN|HGNC=2520|UniProtKB=Q9NRF8,HUMAN|HGNC=20|UniProtKB=P49588,HUMAN|HGNC=4162|UniProtKB=P41250,HUMAN|HGNC=12840|UniProtKB=P54577,HUMAN|HGNC=12729|UniProtKB=P23381,HUMAN|HGNC=850|UniProtKB=P48047,HUMAN|HGNC=4624|UniProtKB=P48637,HUMAN|HGNC=24728|UniProtKB=A2RTX5,HUMAN|HGNC=2678|UniProtKB=P14868,HUMAN|HGNC=87	ligase activity

	1|UniProtKB=Q9UII2,HUMAN|HGNC=11572|UniProtKB=P26639,HUMAN|HGNC=26664|UniProtKB=Q8N841,HUMAN|HGNC=830|UniProtKB=P06576	
GO:1901615	HUMAN|HGNC=3579|UniProtKB=P16930	organic hydroxy compound metabolic process
GO:0004175	HUMAN|HGNC=9545|UniProtKB=P28062,HUMAN|HGNC=9533|UniProtKB=P25789,HUMAN|HGNC=2479|UniProtKB=O76096,HUMAN|HGNC=8993|UniProtKB=P12273,HUMAN|HGNC=9750|UniProtKB=P20742,HUMAN|HGNC=2482|UniProtKB=P04080,HUMAN|HGNC=9554|UniProtKB=Q99460,HUMAN|HGNC=9559|UniProtKB=Q13200,HUMAN|HGNC=9531|UniProtKB=P25787,HUMAN|HGNC=9535|UniProtKB=P60900,HUMAN|HGNC=9539|UniProtKB=P49721,HUMAN|HGNC=3658|UniProtKB=Q9UGM5,HUMAN|HGNC=9536|UniProtKB=O14818,HUMAN|HGNC=9540|UniProtKB=P49720,HUMAN|HGNC=6383|UniProtKB=P01042,HUMAN|HGNC=9530|UniProtKB=P25786,HUMAN|HGNC=9568|UniProtKB=Q06323,HUMAN|HGNC=9532|UniProtKB=P25788,HUMAN|HGNC=9537|UniProtKB=P20618	endopeptidase activity
PC00219	HUMAN|HGNC=7694|UniProtKB=O14561,HUMAN|HGNC=2690|UniProtKB=P07108,HUMAN|HGNC=4187|UniProtKB=P02774,HUMAN|HGNC=316|UniProtKB=P43652,HUMAN|HGNC=317|UniProtKB=P02771,HUMAN|HGNC=10992|UniProtKB=P12236,HUMAN|HGNC=3951|UniProtKB=Q16595,HUMAN|HGNC=18396|UniProtKB=O95969,HUMAN|HGNC=9922|UniProtKB=P02753,HUMAN|HGNC=10991|UniProtKB=P05141,HUMAN|HGNC=11740|UniProtKB=P02787,HUMAN|HGNC=6526|UniProtKB=P80188,HUMAN|HGNC=399|UniProtKB=P02768	transfer/carrier protein
GO:0042113	HUMAN|HGNC=5575|UniProtKB=A0A0B4J1V2,HUMAN|HGNC=5645|UniProtKB=A0A0C4DH34,HUMAN|HGNC=5549|UniProtKB=A0A0C4DH31,HUMAN|HGNC=5554|UniProtKB=P01743,HUMAN|HGNC=5480|UniProtKB=P01880,HUMAN|HGNC=5659|UniProtKB=A0A0C4DH38,HUMAN|HGNC=5634|UniProtKB=A0A075B7F0,HUMAN|HGNC=38476|UniProtKB=B9A064,HUMAN|HGNC=18233|UniProtKB=Q8NDB2,HUMAN|HGNC=5861|UniProtKB=A0M8Q6,HUMAN|HGNC=5647|UniProtKB=A0A087WSY4,HUMAN|HGNC=5563|UniProtKB=A0A075B7D0,HUMAN|HGNC=5856|UniProtKB=P0DOY2,HUMAN|HGNC=5606|UniProtKB=P01763,HUMAN|HGNC=5576|UniProtKB=P01817,HUMAN|HGNC=5528|UniProtKB=P01861,HUMAN|HGNC=5541|UniProtKB=P01871,HUMAN|HGNC=5585|UniProtKB=A0A0C4DH32,HUMAN|HGNC=5551|UniProtKB=A0A0C4DH33,HUMAN|HGNC=5526|UniProtKB=P01859,HUMAN|HGNC=5657|UniProtKB=P0DP08,HUMAN|Ensembl=ENSG00000282639|UniProtKB=A0A0J9YX35,HUMAN|Gene=HV309_HUMAN|UniProtKB=P01782,HUMAN|HGNC=5654|UniProtKB=P01825,HUMAN|HGNC=5553|UniProtKB=A0A0A0MS14,HUMAN|Gene=HV108_HUMAN|UniProtKB=P0DP01,HUMAN|HGNC=5624|UniProtKB=A0A0B4J1X5,HUMAN|HGNC=5620|UniProtKB=P01780,HUMAN|HGNC=5598|UniProtKB=A0A0C4DH35,HUMAN|HGNC=5580|UniProtKB=P01762,HUMAN|HGNC=5633|UniProtKB=A0A075B7D8,HUMAN|HGNC=5582|UniProtKB=A0A0B4J1V0,HUMAN|HGNC=5478|UniProtKB=P01876,HUMAN|HGNC=5617|UniProtKB=A0A075B6Q5,HUMAN|HGNC=5594|UniProtKB=P0DP03,HUMAN|HGNC=5655|UniProtKB=A0A0C4DH41,HUMAN|HGNC=5636|UniProtKB=A0A075B7B8,HUMAN|HGNC=5586|UniProtKB=A0A0B4J1V1,HUMAN|HGNC=5581|UniProtKB=P01766,H	B cell activation

<table>
<tr><td></td><td>UMAN|HGNC=5622|UniProtKB=A0A0B4J1Y9,HUMAN|HGNC=5652|UniProtKB=A0A075B6R2,HUMAN|HGNC=5593|UniProtKB=P0DP02,HUMAN|HGNC=5479|UniProtKB=P01877,HUMAN|HGNC=5619|UniProtKB=A0A0C4DH42,HUMAN|HGNC=5596|UniProtKB=P01772,HUMAN|HGNC=5550|UniProtKB=P23083,HUMAN|HGNC=5604|UniProtKB=A0A0B4J1X8,HUMAN|HGNC=5525|UniProtKB=P01857,HUMAN|HGNC=5662|UniProtKB=A0A0B4J1U7,HUMAN|HGNC=5601|UniProtKB=A0A0C4DH36,HUMAN|HGNC=5716|UniProtKB=P01834,HUMAN|HGNC=5588|UniProtKB=P01764,HUMAN|HGNC=5857|UniProtKB=P0DOY3,HUMAN|HGNC=5610|UniProtKB=P01767,HUMAN|Ensembl=ENSG00000282150|UniProtKB=A0A0J9YY99,HUMAN|HGNC=5527|UniProtKB=P01860</td><td></td></tr>
<tr><td>GO:0034364</td><td>HUMAN|HGNC=607|UniProtKB=P02654,HUMAN|HGNC=1869|UniProtKB=P11597</td><td>high-density lipoprotein particle</td></tr>
<tr><td>GO:0008574</td><td>HUMAN|HGNC=6325|UniProtKB=O60282,HUMAN|HGNC=6317|UniProtKB=O43896</td><td>ATP-dependent microtubule motor activity, plus-end-directed</td></tr>
<tr><td>GO:0097193</td><td>HUMAN|HGNC=1050|UniProtKB=P55957,HUMAN|HGNC=11283|UniProtKB=P12931</td><td>intrinsic apoptotic signaling pathway</td></tr>
<tr><td>GO:0051253</td><td>HUMAN|HGNC=9583|UniProtKB=P26599</td><td>negative regulation of RNA metabolic process</td></tr>
<tr><td>GO:0072594</td><td>HUMAN|HGNC=6499|UniProtKB=P11279,HUMAN|HGNC=2974|UniProtKB=P50570,HUMAN|HGNC=29125|UniProtKB=Q9UQ16</td><td>establishment of protein localization to organelle</td></tr>
<tr><td>GO:0016747</td><td>HUMAN|HGNC=82|UniProtKB=P09110</td><td>transferase activity, transferring acyl groups other than amino-acyl groups</td></tr>
<tr><td>GO:0006939</td><td>HUMAN|HGNC=7590|UniProtKB=Q15746</td><td>smooth muscle contraction</td></tr>
<tr><td>GO:0051130</td><td>HUMAN|HGNC=3757|UniProtKB=O75955,HUMAN|HGNC=243|UniProtKB=P35611</td><td>positive regulation of cellular component organization</td></tr>
<tr><td>GO:0002821</td><td>HUMAN|HGNC=4932|UniProtKB=P01889,HUMAN|HGNC=533|UniProtKB=P04083</td><td>positive regulation of adaptive immune response</td></tr>
<tr><td>GO:0006221</td><td>HUMAN|HGNC=2520|UniProtKB=Q9NRF8</td><td>pyrimidine nucleotide biosynthetic process</td></tr>
<tr><td>GO:0070328</td><td>HUMAN|HGNC=1869|UniProtKB=P11597</td><td>triglyceride homeostasis</td></tr>
<tr><td>GO:0000792</td><td>HUMAN|HGNC=4861|UniProtKB=Q9NRZ9</td><td>heterochromatin</td></tr>
<tr><td>GO:0016323</td><td>HUMAN|HGNC=11030|UniProtKB=Q9Y6R1,HUMAN|HGNC=1681|UniProtKB=P16070,HUMAN|HGNC=11027|UniProtKB=P02730,HUMAN|HGNC=30377|UniProtKB=Q14160</td><td>basolateral plasma membrane</td></tr>
<tr><td>GO:0005768</td><td>HUMAN|HGNC=9782|UniProtKB=P61018,HUMAN|HGNC=31332|UniProtKB=Q6IQ22,HUMAN|HGNC=7007|UniProtKB=P61006,HUMAN|HGNC=16075|UniProtKB=Q9H082,HUMAN|HGNC=24641|UniProtKB=Q7Z3J2,HUMAN|HGNC=11984|UniProtKB=Q6ZVM7,HUMAN|HGNC=9778|UniProtKB=P20337,HUMAN|HGNC=11358|UniProtKB=O75886,HUMAN|HGNC=11357|UniProtKB=Q92783,HUMAN|HGNC=23186|UniProtKB=Q14677</td><td>endosome</td></tr>
<tr><td>GO:0070588</td><td>HUMAN|HGNC=813|UniProtKB=Q93084,HUMAN|HGNC=812|UniProtKB=P16615</td><td>calcium ion transmembrane transport</td></tr>
<tr><td>GO:0042157</td><td>HUMAN|HGNC=6737|UniProtKB=O75608,HUMAN|HGNC=613|UniProtKB=P02649</td><td>lipoprotein metabolic process</td></tr>
</table>

GO:0019693	HUMAN|HGNC=8052|UniProtKB=Q9UKK9	ribose phosphate metabolic process
GO:0008194	HUMAN|HGNC=24293|UniProtKB=Q70JA7	UDP-glycosyltransferase activity
GO:0007043	HUMAN|HGNC=15902|UniProtKB=Q8IXH8,HUMAN|HGNC=14265|UniProtKB=Q86UP0,HUMAN|HGNC=2047|UniProtKB=O00501	cell-cell junction assembly
GO:0000149	HUMAN|HGNC=11442|UniProtKB=O15400,HUMAN|HGNC=20120|UniProtKB=Q17RC7,HUMAN|HGNC=11429|UniProtKB=O75558,HUMAN|HGNC=11512|UniProtKB=Q9H2B2	SNARE binding
GO:0016579	HUMAN|HGNC=7106|UniProtKB=P54252,HUMAN|HGNC=20063|UniProtKB=Q86T82,HUMAN|HGNC=12628|UniProtKB=P45974,HUMAN|HGNC=25035|UniProtKB=Q5VVQ6,HUMAN|HGNC=13485|UniProtKB=Q9BXU7,HUMAN|HGNC=20076|UniProtKB=Q96K76	protein deubiquitination
GO:0060048	HUMAN|HGNC=12010|UniProtKB=P09493	cardiac muscle contraction
GO:0030515	HUMAN|HGNC=12755|UniProtKB=Q9UNX4	snoRNA binding
GO:0043984	HUMAN|HGNC=25473|UniProtKB=Q9P2N6,HUMAN|HGNC=25544|UniProtKB=Q9HCI7	histone H4-K16 acetylation
GO:0022627	HUMAN|HGNC=10404|UniProtKB=P15880,HUMAN|HGNC=10385|UniProtKB=P25398,HUMAN|HGNC=10396|UniProtKB=P62249	cytosolic small ribosomal subunit
GO:0010508	HUMAN|HGNC=16276|UniProtKB=Q9C035	positive regulation of autophagy
GO:0000287	HUMAN|HGNC=5157|UniProtKB=P00492	magnesium ion binding
GO:0042144	HUMAN|HGNC=3350|UniProtKB=P06733,HUMAN|HGNC=3353|UniProtKB=P09104,HUMAN|HGNC=3354|UniProtKB=P13929	vacuole fusion, non-autophagic
GO:1901575	HUMAN|HGNC=253|UniProtKB=P11766,HUMAN|HGNC=13420|UniProtKB=Q86TP1,HUMAN|HGNC=251|UniProtKB=P00326,HUMAN|HGNC=255|UniProtKB=P28332	organic substance catabolic process
GO:0010485	HUMAN|HGNC=25473|UniProtKB=Q9P2N6	H4 histone acetyltransferase activity
GO:0044249	HUMAN|HGNC=12009|UniProtKB=P60174,HUMAN|HGNC=6517|UniProtKB=P18428	cellular biosynthetic process
GO:0032153	HUMAN|HGNC=15848|UniProtKB=Q14141,HUMAN|HGNC=9164|UniProtKB=Q99719,HUMAN|HGNC=7590|UniProtKB=Q15746,HUMAN|HGNC=1717|UniProtKB=Q16181,HUMAN|HGNC=667|UniProtKB=P61586,HUMAN|HGNC=7729|UniProtKB=Q15019	cell division site
GO:0005814	HUMAN|HGNC=14313|UniProtKB=Q8WYA0,HUMAN|HGNC=23432|UniProtKB=Q9NXG0,HUMAN|HGNC=10252|UniProtKB=O75116,HUMAN|HGNC=28959|UniProtKB=Q14008,HUMAN|HGNC=9847|UniProtKB=P43487,HUMAN|HGNC=5048|UniProtKB=Q00839,HUMAN|HGNC=14906|UniProtKB=Q8N4C6,HUMAN|HGNC=26281|UniProtKB=Q6ZN84,HUMAN|HGNC=7910|UniProtKB=P06748,HUMAN|HGNC=18994|UniProtKB=Q8TDC3,HUMAN|HGNC=29262|UniProtKB=Q9P2H3	centriole
GO:0015914	HUMAN|HGNC=9093|UniProtKB=P55058	phospholipid transport
GO:0006606	HUMAN|HGNC=6401|UniProtKB=Q92973,HUMAN|HGNC=2431|UniProtKB=P55060,HUMAN|HGNC=8062|UniProtKB=P49790,HUMAN|HGNC=9846|UniProtKB=P62826,HUMAN|HGNC=17103|UniProtKB=Q9Y5L0,HUMAN|HGNC=9852|UniProtKB=O95373,HUMAN|HGNC=6396|UniProtKB=O00505,HUMAN|HGNC=17859|UniProtKB=Q5SRE5,HUMAN|HGNC=6400|UniProtKB=Q14974	protein import into nucleus

GO:0002708	HUMAN|HGNC=4932|UniProtKB=P01889	positive regulation of lymphocyte mediated immunity
GO:0006891	HUMAN|HGNC=9786|UniProtKB=P20340,HUMAN|HGNC=2230|UniProtKB=P53621,HUMAN|HGNC=14902|UniProtKB=Q9NRW1,HUMAN|HGNC=18621|UniProtKB=Q9Y2V7,HUMAN|HGNC=2231|UniProtKB=P53618,HUMAN|HGNC=2232|UniProtKB=P35606,HUMAN|HGNC=8016|UniProtKB=P46459	intra-Golgi vesicle-mediated transport
GO:0001530	HUMAN|HGNC=1628|UniProtKB=P08571,HUMAN|HGNC=6517|UniProtKB=P18428,HUMAN|HGNC=1472|UniProtKB=P49913	lipopolysaccharide binding
GO:0071705	HUMAN|HGNC=11510|UniProtKB=Q8N9I0,HUMAN|HGNC=11512|UniProtKB=Q9H2B2	nitrogen compound transport
PC00068	HUMAN|HGNC=11056|UniProtKB=P48067,HUMAN|HGNC=800|UniProtKB=P50993,HUMAN|HGNC=18002|UniProtKB=Q9NS69,HUMAN|HGNC=813|UniProtKB=Q93084,HUMAN|HGNC=806|UniProtKB=P54709,HUMAN|HGNC=13531|UniProtKB=Q9Y2Q0,HUMAN|HGNC=17943|UniProtKB=Q9GZP9,HUMAN|HGNC=21101|UniProtKB=O60888,HUMAN|HGNC=11985|UniProtKB=O94826,HUMAN|HGNC=812|UniProtKB=P16615	primary active transporter
GO:1901700	HUMAN|HGNC=1516|UniProtKB=P04040,HUMAN|HGNC=4553|UniProtKB=P07203,HUMAN|HGNC=612|UniProtKB=P05090	response to oxygen-containing compound
GO:0044782	HUMAN|HGNC=26555|UniProtKB=Q3SY00,HUMAN|HGNC=17060|UniProtKB=Q92845	cilium organization
GO:0045921	HUMAN|HGNC=11491|UniProtKB=P43405,HUMAN|HGNC=9767|UniProtKB=O00194	positive regulation of exocytosis
GO:0030900	HUMAN|HGNC=11590|UniProtKB=Q16650	forebrain development
GO:0002181	HUMAN|HGNC=10372|UniProtKB=P05386,HUMAN|HGNC=4622|UniProtKB=Q8IYD1,HUMAN|HGNC=4621|UniProtKB=P15170	cytoplasmic translation
GO:0022407	HUMAN|HGNC=23151|UniProtKB=Q86UX7	regulation of cell-cell adhesion
GO:0042060	HUMAN|HGNC=1090|UniProtKB=Q03001,HUMAN|HGNC=544|UniProtKB=P08133,HUMAN|HGNC=533|UniProtKB=P04083,HUMAN|HGNC=9069|UniProtKB=Q15149,HUMAN|HGNC=25361|UniProtKB=Q8N264	wound healing
GO:0030010	HUMAN|HGNC=16051|UniProtKB=Q8TEW0,HUMAN|HGNC=18994|UniProtKB=Q8TDC3	establishment of cell polarity
GO:0006896	HUMAN|HGNC=555|UniProtKB=O43747,HUMAN|HGNC=568|UniProtKB=O14617	Golgi to vacuole transport
GO:0005815	HUMAN|HGNC=6891|UniProtKB=Q15555,HUMAN|HGNC=6890|UniProtKB=Q15691,HUMAN|HGNC=11126|UniProtKB=P53814	microtubule organizing center
GO:0007264	HUMAN|HGNC=10011|UniProtKB=Q15382,HUMAN|HGNC=21035|UniProtKB=Q8N392	small GTPase mediated signal transduction
GO:0051239	HUMAN|HGNC=613|UniProtKB=P02649	regulation of multicellular organismal process
PC00138	HUMAN|HGNC=16015|UniProtKB=Q9H8S9,HUMAN|HGNC=29801|UniProtKB=Q7L9L4,HUMAN|HGNC=8927|UniProtKB=Q93100	kinase activator
GO:0006289	HUMAN|HGNC=26200|UniProtKB=Q9H668,HUMAN|HGNC=20473|UniProtKB=Q9BX63,HUMAN|HGNC=2717|UniProtKB=Q16531	nucleotide-excision repair
GO:0016791	HUMAN|HGNC=17059|UniProtKB=Q9NTJ5,HUMAN|HGNC=7618|UniProtKB=O14974,HUMAN|HGNC=14451|UniProtKB=Q9BQK8,HUMAN|HGNC=10542|UniProtKB=O95248	phosphatase activity
PC00112	HUMAN|HGNC=16948|UniProtKB=Q969H8,HUMAN|HGNC=5466|UniProtKB=P01344,HUMAN|HGNC=11766|UniProtKB=P01137	growth factor

GO:0031503	HUMAN|HGNC=20120|UniProtKB=Q17RC7,HUMAN|HGNC=2974|UniProtKB=P50570,HUMAN|HGNC=29125|UniProtKB=Q9UQ16	protein-containing complex localization
GO:0008284	HUMAN|HGNC=8601|UniProtKB=Q06141,HUMAN|HGNC=19312|UniProtKB=Q56P03,HUMAN|HGNC=7002|UniProtKB=A6NDR6	positive regulation of cell population proliferation
GO:0016604	HUMAN|HGNC=10499|UniProtKB=P06702	nuclear body
GO:0045216	HUMAN|HGNC=6616|UniProtKB=P48059,HUMAN|HGNC=243|UniProtKB=P35611,HUMAN|HGNC=11828|UniProtKB=Q9UDY2,HUMAN|HGNC=2444|UniProtKB=P41240	cell-cell junction organization
GO:0030983	HUMAN|HGNC=7326|UniProtKB=P20585	mismatched DNA binding
GO:0005637	HUMAN|HGNC=8533|UniProtKB=P51575	nuclear inner membrane
GO:0000151	HUMAN|HGNC=463|UniProtKB=Q9UKV5,HUMAN|HGNC=11619|UniProtKB=Q15370,HUMAN|HGNC=17943|UniProtKB=Q9GZP9	ubiquitin ligase complex
GO:0090288	HUMAN|HGNC=7178|UniProtKB=Q13201	negative regulation of cellular response to growth factor stimulus
GO:0032432	HUMAN|HGNC=6528|UniProtKB=P13796,HUMAN|HGNC=11148|UniProtKB=Q16658,HUMAN|HGNC=4381|UniProtKB=Q14344	actin filament bundle
GO:0019934	HUMAN|HGNC=7237|UniProtKB=Q9Y6F6	cGMP-mediated signaling
GO:0006812	HUMAN|HGNC=11510|UniProtKB=Q8N9I0,HUMAN|HGNC=11512|UniProtKB=Q9H2B2	cation transport
GO:0030141	HUMAN|HGNC=11446|UniProtKB=O00186,HUMAN|HGNC=11445|UniProtKB=Q15833,HUMAN|HGNC=11510|UniProtKB=Q8N9I0,HUMAN|HGNC=2532|UniProtKB=P08311,HUMAN|HGNC=913|UniProtKB=P20160,HUMAN|HGNC=9767|UniProtKB=O00194	secretory granule
GO:0019538	HUMAN|HGNC=6517|UniProtKB=P18428	protein metabolic process
PC00102	HUMAN|HGNC=6561|UniProtKB=P09382,HUMAN|HGNC=7516|UniProtKB=Q9HC84,HUMAN|HGNC=13572|UniProtKB=Q9Y6R7,HUMAN|HGNC=12726|UniProtKB=P04275,HUMAN|HGNC=7515|UniProtKB=P98088,HUMAN|HGNC=12601|UniProtKB=O75445,HUMAN|HGNC=25012|UniProtKB=Q3ZCW2,HUMAN|HGNC=885|UniProtKB=O75882	extracellular matrix protein
GO:0032007	HUMAN|HGNC=18423|UniProtKB=O75140	negative regulation of TOR signaling
PC00061	HUMAN|HGNC=10494|UniProtKB=P26447,HUMAN|HGNC=8044|UniProtKB=P80303,HUMAN|HGNC=9935|UniProtKB=Q14257,HUMAN|HGNC=10498|UniProtKB=P05109,HUMAN|HGNC=10499|UniProtKB=P06702,HUMAN|HGNC=1458|UniProtKB=O43852,HUMAN|HGNC=10496|UniProtKB=P06703,HUMAN|HGNC=1449|UniProtKB=P0DP25,HUMAN|HGNC=1452|UniProtKB=P27482,HUMAN|HGNC=10497|UniProtKB=P31151,HUMAN|HGNC=8043|UniProtKB=Q02818	calmodulin-related
GO:0045597	HUMAN|HGNC=11491|UniProtKB=P43405	positive regulation of cell differentiation
GO:0002062	HUMAN|HGNC=544|UniProtKB=P08133	chondrocyte differentiation
GO:0006368	HUMAN|HGNC=11617|UniProtKB=Q15369,HUMAN|HGNC=24224|UniProtKB=Q9NYV4	transcription elongation from RNA polymerase II promoter

<table>
<tr><td>GO:0032511</td><td>HUMAN|HGNC=20954|UniProtKB=Q9NP79</td><td>late endosome to vacuole transport via multivesicular body sorting pathway</td></tr>
<tr><td>GO:0016829</td><td>HUMAN|HGNC=746|UniProtKB=P04424,HUMAN|HGNC=291|UniProtKB=P30566</td><td>lyase activity</td></tr>
<tr><td>GO:0030029</td><td>HUMAN|HGNC=12736|UniProtKB=O43516,HUMAN|HGNC=21230|UniProtKB=Q9P1F3</td><td>actin filament-based process</td></tr>
<tr><td>GO:0072676</td><td>HUMAN|HGNC=10632|UniProtKB=P13501</td><td>lymphocyte migration</td></tr>
<tr><td>GO:0050853</td><td>HUMAN|HGNC=5575|UniProtKB=A0A0B4J1V2,HUMAN|HGNC=5645|UniProtKB=A0A0C4DH34,HUMAN|HGNC=5549|UniProtKB=A0A0C4DH31,HUMAN|HGNC=5554|UniProtKB=P01743,HUMAN|HGNC=5480|UniProtKB=P01880,HUMAN|HGNC=5659|UniProtKB=A0A0C4DH38,HUMAN|HGNC=5634|UniProtKB=A0A075B7F0,HUMAN|HGNC=38476|UniProtKB=B9A064,HUMAN|HGNC=5861|UniProtKB=A0M8Q6,HUMAN|HGNC=5647|UniProtKB=A0A087WSY4,HUMAN|HGNC=5563|UniProtKB=A0A075B7D0,HUMAN|HGNC=5856|UniProtKB=P0DOY2,HUMAN|HGNC=5606|UniProtKB=P01763,HUMAN|HGNC=5576|UniProtKB=P01817,HUMAN|HGNC=5528|UniProtKB=P01861,HUMAN|HGNC=5541|UniProtKB=P01871,HUMAN|HGNC=5585|UniProtKB=A0A0C4DH32,HUMAN|HGNC=6171|UniProtKB=Q08881,HUMAN|HGNC=5551|UniProtKB=A0A0C4DH33,HUMAN|HGNC=5526|UniProtKB=P01859,HUMAN|HGNC=5657|UniProtKB=P0DP08,HUMAN|Ensembl=ENSG00000282639|UniProtKB=A0A0J9YX35,HUMAN|Gene=HV309_HUMAN|UniProtKB=P01782,HUMAN|HGNC=5654|UniProtKB=P01825,HUMAN|HGNC=5553|UniProtKB=A0A0A0MS14,HUMAN|Gene=HV108_HUMAN|UniProtKB=P0DP01,HUMAN|HGNC=5624|UniProtKB=A0A0B4J1X5,HUMAN|HGNC=5620|UniProtKB=P01780,HUMAN|HGNC=5598|UniProtKB=A0A0C4DH35,HUMAN|HGNC=5580|UniProtKB=P01762,HUMAN|HGNC=5633|UniProtKB=A0A075B7D8,HUMAN|HGNC=5582|UniProtKB=A0A0B4J1V0,HUMAN|HGNC=5478|UniProtKB=P01876,HUMAN|HGNC=5617|UniProtKB=A0A075B6Q5,HUMAN|HGNC=5594|UniProtKB=P0DP03,HUMAN|HGNC=5655|UniProtKB=A0A0C4DH41,HUMAN|HGNC=5636|UniProtKB=A0A075B7B8,HUMAN|HGNC=5586|UniProtKB=A0A0B4J1V1,HUMAN|HGNC=5581|UniProtKB=P01766,HUMAN|HGNC=5622|UniProtKB=A0A0B4J1Y9,HUMAN|HGNC=5652|UniProtKB=A0A075B6R2,HUMAN|HGNC=5593|UniProtKB=P0DP02,HUMAN|HGNC=5479|UniProtKB=P01877,HUMAN|HGNC=5619|UniProtKB=A0A0C4DH42,HUMAN|HGNC=5596|UniProtKB=P01772,HUMAN|HGNC=5550|UniProtKB=P23083,HUMAN|HGNC=11491|UniProtKB=P43405,HUMAN|HGNC=5604|UniProtKB=A0A0B4J1X8,HUMAN|HGNC=5525|UniProtKB=P01857,HUMAN|HGNC=5662|UniProtKB=A0A0B4J1U7,HUMAN|HGNC=5601|UniProtKB=A0A0C4DH36,HUMAN|HGNC=5716|UniProtKB=P01834,HUMAN|HGNC=5588|UniProtKB=P01764,HUMAN|HGNC=5857|UniProtKB=P0DOY3,HUMAN|HGNC=5610|UniProtKB=P01767,HUMAN|Ensembl=ENSG00000282150|UniProtKB=A0A0J9YY99,HUMAN|HGNC=5527|UniProtKB=P01860</td><td>B cell receptor signaling pathway</td></tr>
<tr><td>GO:0051050</td><td>HUMAN|HGNC=26486|UniProtKB=Q6PJW8,HUMAN|HGNC=3757|UniProtKB=O75955,HUMAN|HGNC=10534|UniProtKB=Q9NR31,HUMAN|HGNC=533|UniProtKB=P04083</td><td>positive regulation of transport</td></tr>
<tr><td>GO:0042127</td><td>HUMAN|HGNC=4037|UniProtKB=P06241,HUMAN|HGNC=11364|UniProtKB=P40763,HUMAN|HGNC=24588|UniProtKB=Q86XP6,HU</td><td>regulation of cell population proliferation</td></tr>
</table>

<table>
<tr><td></td><td>MAN|HGNC=12841|UniProtKB=P07947,HUMAN|HGNC=11283|UniProtKB=P12931,HUMAN|HGNC=6735|UniProtKB=P07948,HUMAN|HGNC=11766|UniProtKB=P01137</td><td></td></tr>
<tr><td>GO:0005654</td><td>HUMAN|HGNC=8648|UniProtKB=Q15366,HUMAN|HGNC=5043|UniProtKB=P31942,HUMAN|HGNC=23172|UniProtKB=Q8N699,HUMAN|HGNC=29190|UniProtKB=Q9UPQ9,HUMAN|HGNC=26022|UniProtKB=Q7L0Y3,HUMAN|HGNC=6396|UniProtKB=O00505,HUMAN|HGNC=9813|UniProtKB=P54727,HUMAN|HGNC=26224|UniProtKB=Q9H5Z6,HUMAN|HGNC=8087|UniProtKB=P29728,HUMAN|HGNC=8647|UniProtKB=Q15365,HUMAN|HGNC=9568|UniProtKB=Q06323,HUMAN|HGNC=5036|UniProtKB=Q14103,HUMAN|HGNC=26378|UniProtKB=Q8N7C3,HUMAN|HGNC=16272|UniProtKB=Q96JK9,HUMAN|HGNC=7910|UniProtKB=P06748</td><td>nucleoplasm</td></tr>
<tr><td>PC00028</td><td>HUMAN|HGNC=5253|UniProtKB=P07900,HUMAN|HGNC=5258|UniProtKB=P08238,HUMAN|HGNC=12028|UniProtKB=P14625</td><td>Hsp90 family chaperone</td></tr>
<tr><td>GO:0016746</td><td>HUMAN|HGNC=2422|UniProtKB=O75390,HUMAN|HGNC=3531|UniProtKB=P00488</td><td>transferase activity, transferring acyl groups</td></tr>
<tr><td>GO:0000187</td><td>HUMAN|HGNC=6766|UniProtKB=Q8WXG6,HUMAN|HGNC=6840|UniProtKB=Q02750</td><td>activation of MAPK activity</td></tr>
<tr><td>GO:0050794</td><td>HUMAN|HGNC=17169|UniProtKB=Q13162,HUMAN|HGNC=16753|UniProtKB=P30041,HUMAN|HGNC=9355|UniProtKB=P30044,HUMAN|HGNC=9353|UniProtKB=P32119,HUMAN|HGNC=9354|UniProtKB=P30048,HUMAN|HGNC=9352|UniProtKB=Q06830,HUMAN|HGNC=4623|UniProtKB=P00390,HUMAN|HGNC=10582|UniProtKB=Q9Y5Y9</td><td>regulation of cellular process</td></tr>
<tr><td>GO:0023051</td><td>HUMAN|HGNC=11510|UniProtKB=Q8N9I0,HUMAN|HGNC=11512|UniProtKB=Q9H2B2</td><td>regulation of signaling</td></tr>
<tr><td>GO:0003727</td><td>HUMAN|HGNC=5048|UniProtKB=Q00839,HUMAN|HGNC=6038|UniProtKB=Q12906,HUMAN|HGNC=3285|UniProtKB=P23588</td><td>single-stranded RNA binding</td></tr>
<tr><td>GO:0034976</td><td>HUMAN|HGNC=30167|UniProtKB=P13667,HUMAN|HGNC=8548|UniProtKB=P07237,HUMAN|HGNC=4606|UniProtKB=P30101</td><td>response to endoplasmic reticulum stress</td></tr>
<tr><td>GO:0046580</td><td>HUMAN|HGNC=25361|UniProtKB=Q8N264</td><td>negative regulation of Ras protein signal transduction</td></tr>
<tr><td>GO:1903706</td><td>HUMAN|HGNC=11491|UniProtKB=P43405,HUMAN|HGNC=29884|UniProtKB=Q8TF42</td><td>regulation of hemopoiesis</td></tr>
<tr><td>GO:0031234</td><td>HUMAN|HGNC=4395|UniProtKB=P19086,HUMAN|HGNC=4384|UniProtKB=P63096,HUMAN|HGNC=4398|UniProtKB=P62879,HUMAN|HGNC=4387|UniProtKB=P08754,HUMAN|HGNC=20731|UniProtKB=Q9HAV0,HUMAN|HGNC=4385|UniProtKB=P04899,HUMAN|HGNC=12841|UniProtKB=P07947,HUMAN|HGNC=11283|UniProtKB=P12931,HUMAN|HGNC=4390|UniProtKB=P50148,HUMAN|HGNC=6735|UniProtKB=P07948,HUMAN|HGNC=4037|UniProtKB=P06241,HUMAN|HGNC=4381|UniProtKB=Q14344,HUMAN|HGNC=4393|UniProtKB=P11488,HUMAN|HGNC=4396|UniProtKB=P62873</td><td>extrinsic component of cytoplasmic side of plasma membrane</td></tr>
<tr><td>GO:0006869</td><td>HUMAN|HGNC=9922|UniProtKB=P02753,HUMAN|HGNC=14637|UniProtKB=Q86UK0,HUMAN|HGNC=1869|UniProtKB=P11597</td><td>lipid transport</td></tr>
<tr><td>GO:0008270</td><td>HUMAN|HGNC=7900|UniProtKB=P55786,HUMAN|HGNC=253|UniProtKB=P11766,HUMAN|HGNC=317|UniProtKB=P02771,HUMAN|HGNC=251|UniProtKB=P00326,HUMAN|HGNC=395|UniProtKB=P13716,HUMAN|HGNC=18173|UniProtKB=Q9NZ08,HUMAN|HGNC=255|UniProtKB=P28332</td><td>zinc ion binding</td></tr>
<tr><td>GO:0072666</td><td>HUMAN|HGNC=1406|UniProtKB=Q9Y698</td><td>establishment of protein localization to vacuole</td></tr>
</table>

GO:0006509	HUMAN|HGNC=11820|UniProtKB=P01033,HUMAN|HGNC=188|UniProtKB=O14672	membrane protein ectodomain proteolysis
GO:0031981	HUMAN|HGNC=9552|UniProtKB=P62195	nuclear lumen
GO:0042162	HUMAN|HGNC=19246|UniProtKB=Q9NYB0	telomeric DNA binding
PC00180	HUMAN|HGNC=17169|UniProtKB=Q13162,HUMAN|HGNC=3423|UniProtKB=P11678,HUMAN|HGNC=16753|UniProtKB=P30041,HUMAN|HGNC=4555|UniProtKB=P22352,HUMAN|HGNC=7218|UniProtKB=P05164,HUMAN|HGNC=9353|UniProtKB=P32119,HUMAN|HGNC=1516|UniProtKB=P04040,HUMAN|HGNC=4553|UniProtKB=P07203,HUMAN|HGNC=9354|UniProtKB=P30048,HUMAN|HGNC=9352|UniProtKB=Q06830	peroxidase
GO:0005680	HUMAN|HGNC=15713|UniProtKB=Q9UJX4,HUMAN|HGNC=14452|UniProtKB=Q9NYG5	anaphase-promoting complex
PC00263	HUMAN|HGNC=782|UniProtKB=O95260,HUMAN|HGNC=12840|UniProtKB=P54577	translational protein
GO:0033674	HUMAN|HGNC=19296|UniProtKB=Q9UF33,HUMAN|HGNC=6091|UniProtKB=P06213	positive regulation of kinase activity
GO:0046579	HUMAN|HGNC=3537|UniProtKB=P25116,HUMAN|HGNC=4472|UniProtKB=Q14330,HUMAN|HGNC=3540|UniProtKB=Q96RI0	positive regulation of Ras protein signal transduction
GO:0030374	HUMAN|HGNC=30025|UniProtKB=Q5VV67,HUMAN|HGNC=7670|UniProtKB=Q9Y6Q9	nuclear receptor transcription coactivator activity
GO:0016787	HUMAN|HGNC=18344|UniProtKB=Q9BX68,HUMAN|HGNC=3579|UniProtKB=P16930,HUMAN|HGNC=343|UniProtKB=P23526	hydrolase activity
GO:0004540	HUMAN|HGNC=10046|UniProtKB=P12724,HUMAN|HGNC=10045|UniProtKB=P10153	ribonuclease activity
GO:0005488	HUMAN|HGNC=15848|UniProtKB=Q14141,HUMAN|HGNC=4555|UniProtKB=P22352,HUMAN|HGNC=9164|UniProtKB=Q99719,HUMAN|HGNC=1717|UniProtKB=Q16181,HUMAN|HGNC=6526|UniProtKB=P80188,HUMAN|HGNC=2905|UniProtKB=O14490,HUMAN|HGNC=7729|UniProtKB=Q15019	binding
GO:0071407	HUMAN|HGNC=9855|UniProtKB=P62834,HUMAN|HGNC=9857|UniProtKB=P61224	cellular response to organic cyclic compound
GO:0048194	HUMAN|HGNC=24878|UniProtKB=Q9BVK6	Golgi vesicle budding
GO:0005813	HUMAN|HGNC=14313|UniProtKB=Q8WYA0,HUMAN|HGNC=23432|UniProtKB=Q9NXG0,HUMAN|HGNC=10252|UniProtKB=O75116,HUMAN|HGNC=28959|UniProtKB=Q14008,HUMAN|HGNC=9847|UniProtKB=P43487,HUMAN|HGNC=5048|UniProtKB=Q00839,HUMAN|HGNC=14906|UniProtKB=Q8N4C6,HUMAN|HGNC=26281|UniProtKB=Q6ZN84,HUMAN|HGNC=7910|UniProtKB=P06748,HUMAN|HGNC=18994|UniProtKB=Q8TDC3,HUMAN|HGNC=29262|UniProtKB=Q9P2H3	centrosome
GO:0006836	HUMAN|HGNC=16937|UniProtKB=O75915	neurotransmitter transport
GO:0030518	HUMAN|HGNC=11283|UniProtKB=P12931	intracellular steroid hormone receptor signaling pathway
GO:0016569	HUMAN|HGNC=4861|UniProtKB=Q9NRZ9	covalent chromatin modification

GO:0019888	HUMAN|HGNC=1173|UniProtKB=Q5H9R7,HUMAN|HGNC=9302|UniProtKB=P30153,HUMAN|HGNC=9308|UniProtKB=Q15257,HUMAN|HGNC=9304|UniProtKB=P63151	protein phosphatase regulator activity
GO:0008417	HUMAN|HGNC=14683|UniProtKB=Q9Y2G5	fucosyltransferase activity
GO:0007029	HUMAN|HGNC=24526|UniProtKB=Q6DD88,HUMAN|HGNC=6631|UniProtKB=P49257,HUMAN|HGNC=25541|UniProtKB=P57088,HUMAN|HGNC=12649|UniProtKB=O95292,HUMAN|HGNC=11231|UniProtKB=Q8WXF7,HUMAN|HGNC=12648|UniProtKB=Q9P0L0	endoplasmic reticulum organization
GO:0031638	HUMAN|HGNC=3530|UniProtKB=P00748,HUMAN|HGNC=5141|UniProtKB=P00738,HUMAN|HGNC=21265|UniProtKB=Q9NZP8,HUMAN|HGNC=5156|UniProtKB=P00739	zymogen activation
PC00173	HUMAN|HGNC=18344|UniProtKB=Q9BX68	nucleotide phosphatase
GO:1901363	HUMAN|HGNC=11834|UniProtKB=P29401,HUMAN|HGNC=9723|UniProtKB=P11216,HUMAN|HGNC=9725|UniProtKB=P06737	heterocyclic compound binding
GO:0000781	HUMAN|HGNC=26200|UniProtKB=Q9H668	chromosome, telomeric region
GO:0019432	HUMAN|HGNC=14451|UniProtKB=Q9BQK8	triglyceride biosynthetic process
GO:0009113	HUMAN|HGNC=626|UniProtKB=P07741,HUMAN|HGNC=5157|UniProtKB=P00492	purine nucleobase biosynthetic process
GO:0033962	HUMAN|HGNC=17259|UniProtKB=Q9Y4Z0	cytoplasmic mRNA processing body assembly
GO:0006612	HUMAN|HGNC=30389|UniProtKB=Q96A65,HUMAN|HGNC=7629|UniProtKB=E9PAV3	protein targeting to membrane
GO:0034220	HUMAN|HGNC=16804|UniProtKB=O00308	ion transmembrane transport
GO:0008654	HUMAN|HGNC=9141|UniProtKB=Q15126	phospholipid biosynthetic process
GO:0034968	HUMAN|HGNC=9349|UniProtKB=Q9NQX1	histone lysine methylation
GO:0008250	HUMAN|HGNC=10381|UniProtKB=P04843,HUMAN|HGNC=10382|UniProtKB=P04844,HUMAN|HGNC=2728|UniProtKB=P39656	oligosaccharyltransferase complex
GO:0005856	HUMAN|HGNC=18756|UniProtKB=Q9BYZ6,HUMAN|HGNC=9802|UniProtKB=P15153,HUMAN|HGNC=9801|UniProtKB=P63000,HUMAN|HGNC=243|UniProtKB=P35611,HUMAN|HGNC=17905|UniProtKB=Q9Y613,HUMAN|HGNC=1738|UniProtKB=Q9Y5S2	cytoskeleton
GO:0014069	HUMAN|HGNC=3338|UniProtKB=Q14247,HUMAN|HGNC=15474|UniProtKB=Q9Y566,HUMAN|HGNC=2695|UniProtKB=Q16643,HUMAN|HGNC=2974|UniProtKB=P50570,HUMAN|HGNC=243|UniProtKB=P35611,HUMAN|HGNC=29125|UniProtKB=Q9UQ16,HUMAN|HGNC=2696|UniProtKB=Q9UJU6,HUMAN|HGNC=4844|UniProtKB=P14317,HUMAN|HGNC=14294|UniProtKB=Q9BYB0	postsynaptic density
GO:0042773	HUMAN|HGNC=7421|UniProtKB=P00403	ATP synthesis coupled electron transport
GO:0042165	HUMAN|HGNC=16768|UniProtKB=O60391	neurotransmitter binding
GO:0048675	HUMAN|HGNC=3338|UniProtKB=Q14247,HUMAN|HGNC=2695|UniProtKB=Q16643,HUMAN|HGNC=2696|UniProtKB=Q9UJU6,HUMAN|HGNC=4844|UniProtKB=P14317	axon extension
GO:0005876	HUMAN|HGNC=5048|UniProtKB=Q00839	spindle microtubule
PC00044	HUMAN|HGNC=418|UniProtKB=P09972,HUMAN|HGNC=414|UniProtKB=P04075,HUMAN|HGNC=11559|UniProtKB=P37837	aldolase

<table>
<tr><td>GO:0006259</td><td>HUMAN|HGNC=4861|UniProtKB=Q9NRZ9</td><td>DNA metabolic process</td></tr>
<tr><td>GO:0006367</td><td>HUMAN|HGNC=19985|UniProtKB=P53999,HUMAN|HGNC=17305|UniProtKB=Q9Y6J9</td><td>transcription initiation from RNA polymerase II promoter</td></tr>
<tr><td>PC00111</td><td>HUMAN|HGNC=4127|UniProtKB=Q7Z7M9,HUMAN|HGNC=24293|UniProtKB=Q70JA7,HUMAN|HGNC=10382|UniProtKB=P04844,HUMAN|HGNC=2728|UniProtKB=P39656,HUMAN|HGNC=30611|UniProtKB=Q8TCJ2,HUMAN|HGNC=9725|UniProtKB=P06737,HUMAN|HGNC=4699|UniProtKB=P46976,HUMAN|HGNC=10381|UniProtKB=P04843,HUMAN|HGNC=17973|UniProtKB=Q9P109,HUMAN|HGNC=3148|UniProtKB=P19971,HUMAN|HGNC=9723|UniProtKB=P11216,HUMAN|HGNC=5157|UniProtKB=P00492,HUMAN|HGNC=15663|UniProtKB=Q9NYU2,HUMAN|HGNC=14683|UniProtKB=Q9Y2G5</td><td>glycosyltransferase</td></tr>
<tr><td>GO:0005773</td><td>HUMAN|HGNC=25951|UniProtKB=Q6ULP2,HUMAN|Gene=SEP15|UniProtKB=O60613,HUMAN|HGNC=9784|UniProtKB=P61020,HUMAN|HGNC=11834|UniProtKB=P29401,HUMAN|HGNC=555|UniProtKB=O43747,HUMAN|HGNC=813|UniProtKB=Q93084,HUMAN|HGNC=13487|UniProtKB=Q96QK1,HUMAN|HGNC=463|UniProtKB=Q9UKV5,HUMAN|HGNC=11445|UniProtKB=Q15833,HUMAN|HGNC=9756|UniProtKB=O00391,HUMAN|HGNC=15453|UniProtKB=Q9H3P7,HUMAN|HGNC=9761|UniProtKB=Q15907,HUMAN|HGNC=10996|UniProtKB=O14975,HUMAN|HGNC=9785|UniProtKB=P51148,HUMAN|HGNC=2230|UniProtKB=P53621,HUMAN|HGNC=2431|UniProtKB=P55060,HUMAN|HGNC=16268|UniProtKB=Q8IY17,HUMAN|HGNC=9772|UniProtKB=Q13637,HUMAN|HGNC=24251|UniProtKB=Q9Y3A6,HUMAN|HGNC=8569|UniProtKB=P29122,HUMAN|HGNC=4606|UniProtKB=P30101,HUMAN|HGNC=25153|UniProtKB=Q96EY5,HUMAN|HGNC=9782|UniProtKB=P61018,HUMAN|HGNC=11446|UniProtKB=O00186,HUMAN|HGNC=11172|UniProtKB=Q13596,HUMAN|HGNC=16499|UniProtKB=Q96DA2,HUMAN|HGNC=16075|UniProtKB=Q9H082,HUMAN|HGNC=11442|UniProtKB=O15400,HUMAN|HGNC=9852|UniProtKB=O95373,HUMAN|HGNC=16090|UniProtKB=O00264,HUMAN|HGNC=11740|UniProtKB=P02787,HUMAN|HGNC=16996|UniProtKB=Q15363,HUMAN|HGNC=30384|UniProtKB=Q9UMX5,HUMAN|HGNC=7007|UniProtKB=P61006,HUMAN|HGNC=14902|UniProtKB=Q9NRW1,HUMAN|HGNC=26212|UniProtKB=P61009,HUMAN|HGNC=9935|UniProtKB=Q14257,HUMAN|HGNC=11984|UniProtKB=Q6ZVM7,HUMAN|HGNC=15999|UniProtKB=Q9NZV5,HUMAN|HGNC=23186|UniProtKB=Q14677,HUMAN|HGNC=8016|UniProtKB=P46459,HUMAN|HGNC=16524|UniProtKB=P61106,HUMAN|HGNC=20726|UniProtKB=Q8WVM8,HUMAN|HGNC=1458|UniProtKB=O43852,HUMAN|HGNC=13529|UniProtKB=Q9Y2B0,HUMAN|HGNC=2532|UniProtKB=P08311,HUMAN|HGNC=5238|UniProtKB=P11021,HUMAN|HGNC=16998|UniProtKB=P49755,HUMAN|HGNC=11357|UniProtKB=Q92783,HUMAN|HGNC=3419|UniProtKB=P42566,HUMAN|HGNC=16695|UniProtKB=P51572,HUMAN|HGNC=30167|UniProtKB=P13667,HUMAN|HGNC=8761|UniProtKB=Q9BUL8,HUMAN|HGNC=20954|UniProtKB=Q9NP79,HUMAN|HGNC=15461|UniProtKB=P55145,HUMAN|HGNC=21354|UniProtKB=Q86XR7,HUMAN|HGNC=24634|UniProtKB=Q9UBC2,HUMAN|HGNC=1546|UniProtKB=P50454,HUMAN|HGNC=30220|UniProtKB=Q96AA3,HUMAN|HGNC=3530|UniProtKB=P00748,HU</td><td>vacuole</td></tr>
</table>

<table>
<tr><td></td><td>MAN|HGNC=9786|UniProtKB=P20340,HUMAN|HGNC=17103|UniProtKB=Q9Y5L0,HUMAN|HGNC=9164|UniProtKB=Q99719,HUMAN|HGNC=10534|UniProtKB=Q9NR31,HUMAN|HGNC=15588|UniProtKB=Q96C24,HUMAN|HGNC=6631|UniProtKB=P49257,HUMAN|HGNC=649|UniProtKB=P48444,HUMAN|HGNC=18370|UniProtKB=Q9H0U4,HUMAN|HGNC=24641|UniProtKB=Q7Z3J2,HUMAN|HGNC=19983|UniProtKB=Q86YS6,HUMAN|HGNC=12666|UniProtKB=P55072,HUMAN|HGNC=10382|UniProtKB=P04844,HUMAN|HGNC=9411|UniProtKB=P14314,HUMAN|HGNC=17943|UniProtKB=Q9GZP9,HUMAN|HGNC=6752|UniProtKB=P20645,HUMAN|HGNC=15606|UniProtKB=Q9UHA4,HUMAN|HGNC=24175|UniProtKB=Q9P035,HUMAN|HGNC=16089|UniProtKB=O15173,HUMAN|HGNC=8533|UniProtKB=P51575,HUMAN|HGNC=17059|UniProtKB=Q9NTJ5,HUMAN|HGNC=11358|UniProtKB=O75886,HUMAN|HGNC=17859|UniProtKB=Q5SRE5,HUMAN|HGNC=25541|UniProtKB=P57088,HUMAN|HGNC=2090|UniProtKB=P09496,HUMAN|HGNC=8515|UniProtKB=Q9HC10,HUMAN|HGNC=30052|UniProtKB=Q8TEM1,HUMAN|HGNC=9778|UniProtKB=P20337,HUMAN|HGNC=8548|UniProtKB=P07237,HUMAN|HGNC=568|UniProtKB=O14617,HUMAN|HGNC=18673|UniProtKB=Q96JB5,HUMAN|HGNC=1473|UniProtKB=P27824,HUMAN|HGNC=8504|UniProtKB=Q969R2,HUMAN|HGNC=3244|UniProtKB=Q9NZN3,HUMAN|HGNC=11510|UniProtKB=Q8N9I0,HUMAN|HGNC=13921|UniProtKB=O95870,HUMAN|HGNC=12649|UniProtKB=O95292,HUMAN|HGNC=20653|UniProtKB=Q8IVB4,HUMAN|HGNC=26486|UniProtKB=Q6PJW8,HUMAN|HGNC=30550|UniProtKB=O60337,HUMAN|HGNC=4248|UniProtKB=Q92820,HUMAN|HGNC=2689|UniProtKB=P09172,HUMAN|HGNC=10701|UniProtKB=Q15436,HUMAN|HGNC=11326|UniProtKB=P51571,HUMAN|HGNC=4298|UniProtKB=P16278,HUMAN|HGNC=8062|UniProtKB=P49790,HUMAN|HGNC=9758|UniProtKB=P62820,HUMAN|HGNC=2232|UniProtKB=P35606,HUMAN|HGNC=561|UniProtKB=O95782,HUMAN|HGNC=11512|UniProtKB=Q9H2B2,HUMAN|HGNC=18263|UniProtKB=Q9UL25,HUMAN|HGNC=812|UniProtKB=P16615,HUMAN|HGNC=692|UniProtKB=P40616,HUMAN|HGNC=31332|UniProtKB=Q6IQ22,HUMAN|HGNC=18587|UniProtKB=O94901,HUMAN|HGNC=18621|UniProtKB=Q9Y2V7,HUMAN|HGNC=1455|UniProtKB=P27797,HUMAN|HGNC=2231|UniProtKB=P53618,HUMAN|HGNC=2728|UniProtKB=P39656,HUMAN|HGNC=17643|UniProtKB=Q9HD26,HUMAN|HGNC=20175|UniProtKB=Q9Y3E0,HUMAN|HGNC=12648|UniProtKB=Q9P0L0,HUMAN|HGNC=1801|UniProtKB=O95674,HUMAN|HGNC=10381|UniProtKB=P04843,HUMAN|HGNC=11429|UniProtKB=O75558,HUMAN|HGNC=9847|UniProtKB=P43487,HUMAN|HGNC=15663|UniProtKB=Q9NYU2,HUMAN|HGNC=24878|UniProtKB=Q9BVK6,HUMAN|HGNC=9767|UniProtKB=O00194</td><td></td></tr>
<tr><td>GO:0097191</td><td>HUMAN|HGNC=1050|UniProtKB=P55957,HUMAN|HGNC=17712|UniProtKB=O15304,HUMAN|HGNC=11283|UniProtKB=P12931</td><td>extrinsic apoptotic signaling pathway</td></tr>
<tr><td>GO:0006388</td><td>HUMAN|HGNC=28422|UniProtKB=Q8NCE0</td><td>tRNA splicing, via endonucleolytic cleavage and ligation</td></tr>
<tr><td>GO:0030119</td><td>HUMAN|HGNC=25951|UniProtKB=Q6ULP2,HUMAN|HGNC=555|UniProtKB=O43747,HUMAN|HGNC=568|UniProtKB=O14617,HUMAN|HGNC=561|UniProtKB=O95782</td><td>AP-type membrane coat adaptor complex</td></tr>
</table>

GO:0043124	HUMAN|HGNC=29890|UniProtKB=Q86UT6,HUMAN|HGNC=9275|UniProtKB=P35813	negative regulation of I-kappaB kinase/NF-kappaB signaling
GO:0072329	HUMAN|HGNC=3579|UniProtKB=P16930,HUMAN|HGNC=8891|UniProtKB=P52209	monocarboxylic acid catabolic process
GO:0061844	HUMAN|HGNC=8601|UniProtKB=Q06141,HUMAN|HGNC=9240|UniProtKB=P02775,HUMAN|HGNC=2761|UniProtKB=P59665,HUMAN|HGNC=8862|UniProtKB=P10720,HUMAN|HGNC=8861|UniProtKB=P02776,HUMAN|HGNC=1472|UniProtKB=P49913	antimicrobial humoral immune response mediated by antimicrobial peptide
GO:0007166	HUMAN|HGNC=3616|UniProtKB=P12318,HUMAN|HGNC=8823|UniProtKB=P16284,HUMAN|HGNC=6871|UniProtKB=P28482,HUMAN|HGNC=533|UniProtKB=P04083,HUMAN|HGNC=1628|UniProtKB=P08571,HUMAN|HGNC=12203|UniProtKB=A0A075B6N3,HUMAN|HGNC=6517|UniProtKB=P18428	cell surface receptor signaling pathway
GO:0000422	HUMAN|HGNC=21689|UniProtKB=Q9Y3D6,HUMAN|HGNC=2973|UniProtKB=O00429	autophagy of mitochondrion
GO:0051650	HUMAN|HGNC=9767|UniProtKB=O00194	establishment of vesicle localization
GO:0030173	HUMAN|HGNC=8569|UniProtKB=P29122,HUMAN|HGNC=9756|UniProtKB=O00391	integral component of Golgi membrane
GO:0010769	HUMAN|HGNC=6616|UniProtKB=P48059	regulation of cell morphogenesis involved in differentiation
GO:0090502	HUMAN|HGNC=20165|UniProtKB=Q9P2P1	RNA phosphodiester bond hydrolysis, endonucleolytic
PC00237	HUMAN|HGNC=39025|UniProtKB=Q9QC07,HUMAN|Gene=ENH1_HUMAN|UniProtKB=Q9N2K0	viral or transposable element protein
PC00185	HUMAN|HGNC=8783|UniProtKB=Q08499,HUMAN|HGNC=21416|UniProtKB=Q92485,HUMAN|HGNC=8784|UniProtKB=O76074	phosphodiesterase
GO:0007224	HUMAN|HGNC=11119|UniProtKB=Q99835	smoothened signaling pathway
GO:0043436	HUMAN|HGNC=13420|UniProtKB=Q86TP1	oxoacid metabolic process
GO:0006826	HUMAN|HGNC=11740|UniProtKB=P02787,HUMAN|HGNC=2295|UniProtKB=P00450,HUMAN|HGNC=11763|UniProtKB=P02786	iron ion transport
GO:0099512	HUMAN|HGNC=2209|UniProtKB=P20908	supramolecular fiber
GO:0055037	HUMAN|HGNC=3244|UniProtKB=Q9NZN3,HUMAN|HGNC=11740|UniProtKB=P02787,HUMAN|HGNC=20653|UniProtKB=Q8IVB4,HUMAN|HGNC=9761|UniProtKB=Q15907	recycling endosome
GO:0006518	HUMAN|HGNC=7900|UniProtKB=P55786,HUMAN|HGNC=5381|UniProtKB=P14735,HUMAN|HGNC=8569|UniProtKB=P29122,HUMAN|HGNC=18173|UniProtKB=Q9NZ08,HUMAN|HGNC=2312|UniProtKB=P15169	peptide metabolic process
GO:0004629	HUMAN|HGNC=9055|UniProtKB=Q00722	phospholipase C activity
GO:0003725	HUMAN|HGNC=8087|UniProtKB=P29728,HUMAN|HGNC=226|UniProtKB=P78563,HUMAN|HGNC=17904|UniProtKB=Q9NRR4,HUMAN|HGNC=6038|UniProtKB=Q12906,HUMAN|HGNC=3285|UniProtKB=P23588	double-stranded RNA binding
GO:0030509	HUMAN|HGNC=6767|UniProtKB=Q15797,HUMAN|HGNC=11766|UniProtKB=P01137	BMP signaling pathway
GO:0032990	HUMAN|HGNC=6047|UniProtKB=Q16891	cell part morphogenesis

<table>
<tr><td>GO:0035861</td><td>HUMAN|HGNC=26200|UniProtKB=Q9H668</td><td>site of double-strand break</td></tr>
<tr><td>GO:0006396</td><td>HUMAN|HGNC=226|UniProtKB=P78563,HUMAN|HGNC=17904|UniProtKB=Q9NRR4</td><td>RNA processing</td></tr>
<tr><td>GO:0005759</td><td>HUMAN|HGNC=5269|UniProtKB=P61604,HUMAN|HGNC=5261|UniProtKB=P10809,HUMAN|HGNC=2422|UniProtKB=O75390,HUMAN|HGNC=4801|UniProtKB=P40939</td><td>mitochondrial matrix</td></tr>
<tr><td>GO:0006790</td><td>HUMAN|HGNC=3189|UniProtKB=P68104,HUMAN|HGNC=7413|UniProtKB=Q13126,HUMAN|HGNC=4622|UniProtKB=Q8IYD1,HUMAN|HGNC=4621|UniProtKB=P15170,HUMAN|HGNC=3200|UniProtKB=Q5VTE0,HUMAN|HGNC=343|UniProtKB=P23526,HUMAN|HGNC=12388|UniProtKB=Q16762,HUMAN|HGNC=7223|UniProtKB=P25325</td><td>sulfur compound metabolic process</td></tr>
<tr><td>GO:0010008</td><td>HUMAN|HGNC=11172|UniProtKB=Q13596,HUMAN|HGNC=3244|UniProtKB=Q9NZN3,HUMAN|HGNC=568|UniProtKB=O14617</td><td>endosome membrane</td></tr>
<tr><td>GO:0007165</td><td>HUMAN|HGNC=8510|UniProtKB=Q92882,HUMAN|HGNC=8783|UniProtKB=Q08499,HUMAN|HGNC=28817|UniProtKB=Q96AG4,HUMAN|HGNC=11984|UniProtKB=Q6ZVM7,HUMAN|HGNC=10464|UniProtKB=Q15404,HUMAN|HGNC=11358|UniProtKB=O75886,HUMAN|HGNC=7967|UniProtKB=Q96RI1,HUMAN|HGNC=6050|UniProtKB=P29218,HUMAN|HGNC=15842|UniProtKB=Q96RT1,HUMAN|HGNC=8784|UniProtKB=O76074</td><td>signal transduction</td></tr>
<tr><td>GO:0005048</td><td>HUMAN|HGNC=6401|UniProtKB=Q92973,HUMAN|HGNC=8062|UniProtKB=P49790,HUMAN|HGNC=6396|UniProtKB=O00505,HUMAN|HGNC=6400|UniProtKB=Q14974</td><td>signal sequence binding</td></tr>
<tr><td>GO:0005746</td><td>HUMAN|HGNC=7687|UniProtKB=O00483,HUMAN|HGNC=2265|UniProtKB=P13073,HUMAN|HGNC=2288|UniProtKB=P14406,HUMAN|HGNC=2267|UniProtKB=P20674</td><td>mitochondrial respirasome</td></tr>
<tr><td>GO:0007186</td><td>HUMAN|HGNC=711|UniProtKB=P49407,HUMAN|HGNC=4398|UniProtKB=P62879,HUMAN|HGNC=10632|UniProtKB=P13501,HUMAN|HGNC=20731|UniProtKB=Q9HAV0,HUMAN|HGNC=3964|UniProtKB=P01225,HUMAN|HGNC=16452|UniProtKB=P0DN86,HUMAN|HGNC=16721|UniProtKB=A6NKQ9,HUMAN|HGNC=4396|UniProtKB=P62873</td><td>G protein-coupled receptor signaling pathway</td></tr>
<tr><td>GO:0010468</td><td>HUMAN|HGNC=8648|UniProtKB=Q15366,HUMAN|HGNC=6871|UniProtKB=P28482,HUMAN|HGNC=5036|UniProtKB=Q14103,HUMAN|HGNC=6876|UniProtKB=Q16539,HUMAN|HGNC=13506|UniProtKB=Q8TBJ5</td><td>regulation of gene expression</td></tr>
<tr><td>GO:0050821</td><td>HUMAN|HGNC=5253|UniProtKB=P07900,HUMAN|HGNC=5258|UniProtKB=P08238,HUMAN|HGNC=1735|UniProtKB=Q16543</td><td>protein stabilization</td></tr>
<tr><td>GO:0001671</td><td>HUMAN|HGNC=806|UniProtKB=P54709</td><td>ATPase activator activity</td></tr>
<tr><td>GO:0043161</td><td>HUMAN|HGNC=9545|UniProtKB=P28062,HUMAN|HGNC=9533|UniProtKB=P25789,HUMAN|HGNC=15713|UniProtKB=Q9UJX4,HUMAN|HGNC=9554|UniProtKB=Q99460,HUMAN|HGNC=2717|UniProtKB=Q16531,HUMAN|HGNC=9813|UniProtKB=P54727,HUMAN|HGNC=16804|UniProtKB=O00308,HUMAN|HGNC=9559|UniProtKB=Q13200,HUMAN|HGNC=9531|UniProtKB=P25787,HUMAN|HGNC=9535|UniProtKB=P60900,HUMAN|HGNC=9539|UniProtKB=P49721,HUMAN|HGNC=9536|UniProtKB=O14818,HUMAN|HGNC=9540|UniProtKB=P49720,HUMAN|HGNC=15912|UniProtKB=Q9UNZ2,HUMAN|HGNC=9530|UniProtKB=P25786,HUMAN|HGNC=9565|UniProtKB=P51665,HUMAN|HGNC=9532|UniProtKB=P25788,HUMAN|HGNC=9537|UniProtKB=P20618</td><td>proteasome-mediated ubiquitin-dependent protein catabolic process</td></tr>
</table>

GO:0015850	HUMAN|HGNC=9922|UniProtKB=P02753,HUMAN|HGNC=11510|UniProtKB=Q8N9I0,HUMAN|HGNC=11512|UniProtKB=Q9H2B2	organic hydroxy compound transport
PC00166	HUMAN|HGNC=6891|UniProtKB=Q15555,HUMAN|HGNC=18587|UniProtKB=O94901,HUMAN|HGNC=6890|UniProtKB=Q15691,HUMAN|HGNC=12022|UniProtKB=P13693,HUMAN|HGNC=28959|UniProtKB=Q14008,HUMAN|HGNC=6835|UniProtKB=P78559,HUMAN|HGNC=1990|UniProtKB=Q8WWK9	non-motor microtubule binding protein
PC00208	HUMAN|HGNC=18756|UniProtKB=Q9BYZ6,HUMAN|HGNC=9862|UniProtKB=P61225,HUMAN|HGNC=9801|UniProtKB=P63000,HUMAN|HGNC=6407|UniProtKB=P01116,HUMAN|HGNC=669|UniProtKB=P08134,HUMAN|HGNC=9857|UniProtKB=P61224,HUMAN|HGNC=10447|UniProtKB=P10301,HUMAN|HGNC=667|UniProtKB=P61586,HUMAN|HGNC=9855|UniProtKB=P62834,HUMAN|HGNC=10011|UniProtKB=Q15382,HUMAN|HGNC=9802|UniProtKB=P15153,HUMAN|HGNC=1736|UniProtKB=P60953,HUMAN|HGNC=9846|UniProtKB=P62826,HUMAN|HGNC=9840|UniProtKB=P11234	small GTPase
GO:0000307	HUMAN|HGNC=24224|UniProtKB=Q9NYV4	cyclin-dependent protein kinase holoenzyme complex
GO:0043021	HUMAN|HGNC=10372|UniProtKB=P05386,HUMAN|HGNC=5048|UniProtKB=Q00839,HUMAN|HGNC=3285|UniProtKB=P23588	ribonucleoprotein complex binding
GO:0043065	HUMAN|HGNC=1504|UniProtKB=P42574,HUMAN|HGNC=533|UniProtKB=P04083	positive regulation of apoptotic process
GO:0019722	HUMAN|HGNC=9314|UniProtKB=Q08209	calcium-mediated signaling
GO:0009059	HUMAN|HGNC=6517|UniProtKB=P18428	macromolecule biosynthetic process
PC00165	HUMAN|HGNC=1441|UniProtKB=Q05682,HUMAN|HGNC=12733|UniProtKB=Q9Y6W5,HUMAN|HGNC=1488|UniProtKB=P52907,HUMAN|HGNC=2696|UniProtKB=Q9UJU6,HUMAN|HGNC=4620|UniProtKB=P06396,HUMAN|HGNC=2252|UniProtKB=P31146,HUMAN|HGNC=15750|UniProtKB=P60981,HUMAN|HGNC=3338|UniProtKB=Q14247,HUMAN|HGNC=3750|UniProtKB=Q13045,HUMAN|HGNC=2254|UniProtKB=Q9ULV4,HUMAN|HGNC=2253|UniProtKB=Q9BR76,HUMAN|HGNC=6528|UniProtKB=P13796,HUMAN|HGNC=27937|UniProtKB=Q6ZVH7,HUMAN|HGNC=12690|UniProtKB=P09327,HUMAN|HGNC=11148|UniProtKB=Q16658,HUMAN|HGNC=2695|UniProtKB=Q16643,HUMAN|HGNC=12754|UniProtKB=O75083,HUMAN|HGNC=8881|UniProtKB=P07737,HUMAN|HGNC=1874|UniProtKB=P23528,HUMAN|HGNC=18304|UniProtKB=Q14019,HUMAN|HGNC=6707|UniProtKB=P33241,HUMAN|HGNC=26161|UniProtKB=P57737,HUMAN|HGNC=9621|UniProtKB=Q6IBS0,HUMAN|HGNC=243|UniProtKB=P35611,HUMAN|HGNC=1491|UniProtKB=P47756,HUMAN|HGNC=4844|UniProtKB=P14317,HUMAN|HGNC=11554|UniProtKB=P37802	non-motor actin binding protein
GO:0031072	HUMAN|HGNC=5241|UniProtKB=P11142,HUMAN|HGNC=5239|UniProtKB=P17066,HUMAN|HGNC=1735|UniProtKB=Q16543,HUMAN|HGNC=5238|UniProtKB=P11021,HUMAN|HGNC=5240|UniProtKB=P48741,HUMAN|HGNC=11343|UniProtKB=P50502	heat shock protein binding
GO:0006874	HUMAN|HGNC=813|UniProtKB=Q93084,HUMAN|HGNC=812|UniProtKB=P16615	cellular calcium ion homeostasis
GO:0034404	HUMAN|HGNC=5157|UniProtKB=P00492	nucleobase-containing small molecule biosynthetic process

GO:1902652	HUMAN|HGNC=5382|UniProtKB=O75874,HUMAN|HGNC=5383|UniProtKB=P48735,HUMAN|HGNC=5386|UniProtKB=P51553	secondary alcohol metabolic process
GO:0070382	HUMAN|HGNC=15588|UniProtKB=Q96C24,HUMAN|HGNC=11512|UniProtKB=Q9H2B2	exocytic vesicle
PC00121	HUMAN|HGNC=12405|UniProtKB=P02766,HUMAN|HGNC=24254|UniProtKB=Q96CN7,HUMAN|HGNC=1122|UniProtKB=P43251,HUMAN|HGNC=343|UniProtKB=P23526,HUMAN|HGNC=8903|UniProtKB=O95336	hydrolase
GO:0048024	HUMAN|HGNC=9583|UniProtKB=P26599,HUMAN|HGNC=5044|UniProtKB=P61978,HUMAN|HGNC=24932|UniProtKB=Q9NZ63	regulation of mRNA splicing, via spliceosome
GO:0015926	HUMAN|HGNC=15527|UniProtKB=Q86Z14	glucosidase activity
GO:0010517	HUMAN|HGNC=533|UniProtKB=P04083	regulation of phospholipase activity
PC00055	HUMAN|HGNC=14008|UniProtKB=Q9BW11,HUMAN|HGNC=29353|UniProtKB=Q6PI77,HUMAN|HGNC=7629|UniProtKB=E9PAV3	basic helix-loop-helix transcription factor
GO:0051651	HUMAN|HGNC=9621|UniProtKB=Q6IBS0,HUMAN|HGNC=1908|UniProtKB=Q96RL7,HUMAN|HGNC=11881|UniProtKB=P62328,HUMAN|HGNC=23594|UniProtKB=Q709C8	maintenance of location in cell
GO:0051279	HUMAN|HGNC=29884|UniProtKB=Q8TF42	regulation of release of sequestered calcium ion into cytosol
GO:0043624	HUMAN|HGNC=7641|UniProtKB=P54920	cellular protein complex disassembly
GO:0000462	HUMAN|HGNC=10396|UniProtKB=P62249	maturation of SSU-rRNA from tricistronic rRNA transcript (SSU-rRNA, 5.8S rRNA, LSU-rRNA)
GO:0036126	HUMAN|HGNC=8898|UniProtKB=P07205	sperm flagellum
GO:0051251	HUMAN|HGNC=5575|UniProtKB=A0A0B4J1V2,HUMAN|HGNC=5645|UniProtKB=A0A0C4DH34,HUMAN|HGNC=5549|UniProtKB=A0A0C4DH31,HUMAN|HGNC=5554|UniProtKB=P01743,HUMAN|HGNC=5480|UniProtKB=P01880,HUMAN|HGNC=5659|UniProtKB=A0A0C4DH38,HUMAN|HGNC=5634|UniProtKB=A0A075B7F0,HUMAN|HGNC=38476|UniProtKB=B9A064,HUMAN|HGNC=5861|UniProtKB=A0M8Q6,HUMAN|HGNC=5647|UniProtKB=A0A087WSY4,HUMAN|HGNC=5563|UniProtKB=A0A075B7D0,HUMAN|HGNC=5856|UniProtKB=P0DOY2,HUMAN|HGNC=5606|UniProtKB=P01763,HUMAN|HGNC=5576|UniProtKB=P01817,HUMAN|HGNC=5528|UniProtKB=P01861,HUMAN|HGNC=5541|UniProtKB=P01871,HUMAN|HGNC=5585|UniProtKB=A0A0C4DH32,HUMAN|HGNC=5551|UniProtKB=A0A0C4DH33,HUMAN|HGNC=5526|UniProtKB=P01859,HUMAN|HGNC=5657|UniProtKB=P0DP08,HUMAN|Ensembl=ENSG00000282639|UniProtKB=A0A0J9YX35,HUMAN|Gene=HV309_HUMAN|UniProtKB=P01782,HUMAN|HGNC=5654|UniProtKB=P01825,HUMAN|HGNC=5553|UniProtKB=A0A0A0MS14,HUMAN|Gene=HV108_HUMAN|UniProtKB=P0DP01,HUMAN|HGNC=5624|UniProtKB=A0A0B4J1X5,HUMAN|HGNC=5620|UniProtKB=P01780,HUMAN|HGNC=5598|UniProtKB=A0A0C4DH35,HUMAN|HGNC=5580|UniProtKB=P01762,HUMAN|HGNC=5633|UniProtKB=A0A075B7D8,HUMAN|HGNC=5582|UniProtKB=A0A0B4J1V0,HUMAN|HGNC=5478|UniProtKB=P01876,HUMAN|HGNC=5617|UniProtKB=A0A075B6Q5,HUMAN|HGNC=5594|UniProtKB=P0DP03,HUMAN|HGNC=5655|UniProtKB	positive regulation of lymphocyte activation

<table>
<tr><td></td><td>=A0A0C4DH41,HUMAN|HGNC=5636|UniProtKB=A0A075B7B8,HUMAN|HGNC=5586|UniProtKB=A0A0B4J1V1,HUMAN|HGNC=5581|UniProtKB=P01766,HUMAN|HGNC=5622|UniProtKB=A0A0B4J1Y9,HUMAN|HGNC=5652|UniProtKB=A0A075B6R2,HUMAN|HGNC=5593|UniProtKB=P0DP02,HUMAN|HGNC=5479|UniProtKB=P01877,HUMAN|HGNC=5619|UniProtKB=A0A0C4DH42,HUMAN|HGNC=5596|UniProtKB=P01772,HUMAN|HGNC=5550|UniProtKB=P23083,HUMAN|HGNC=5604|UniProtKB=A0A0B4J1X8,HUMAN|HGNC=5525|UniProtKB=P01857,HUMAN|HGNC=5662|UniProtKB=A0A0B4J1U7,HUMAN|HGNC=5601|UniProtKB=A0A0C4DH36,HUMAN|HGNC=5716|UniProtKB=P01834,HUMAN|HGNC=5588|UniProtKB=P01764,HUMAN|HGNC=5857|UniProtKB=P0DOY3,HUMAN|HGNC=5610|UniProtKB=P01767,HUMAN|Ensembl=ENSG00000282150|UniProtKB=A0A0J9YY99,HUMAN|HGNC=5527|UniProtKB=P01860</td><td></td></tr>
<tr><td>GO:0042383</td><td>HUMAN|HGNC=9069|UniProtKB=Q15149</td><td>sarcolemma</td></tr>
<tr><td>GO:0006487</td><td>HUMAN|HGNC=4242|UniProtKB=O94808,HUMAN|HGNC=10382|UniProtKB=P04844</td><td>protein N-linked glycosylation</td></tr>
<tr><td>GO:0016573</td><td>HUMAN|HGNC=17305|UniProtKB=Q9Y6J9,HUMAN|HGNC=10474|UniProtKB=Q9Y265,HUMAN|HGNC=10475|UniProtKB=Q9Y230</td><td>histone acetylation</td></tr>
<tr><td>GO:0031507</td><td>HUMAN|HGNC=4861|UniProtKB=Q9NRZ9</td><td>heterochromatin assembly</td></tr>
<tr><td>GO:0006338</td><td>HUMAN|HGNC=10474|UniProtKB=Q9Y265,HUMAN|HGNC=7910|UniProtKB=P06748,HUMAN|HGNC=10475|UniProtKB=Q9Y230,HUMAN|HGNC=18039|UniProtKB=Q9UGL1</td><td>chromatin remodeling</td></tr>
<tr><td>GO:0007044</td><td>HUMAN|HGNC=1090|UniProtKB=Q03001,HUMAN|HGNC=3778|UniProtKB=P02751,HUMAN|HGNC=9069|UniProtKB=Q15149</td><td>cell-substrate junction assembly</td></tr>
<tr><td>GO:0016763</td><td>HUMAN|HGNC=7413|UniProtKB=Q13126,HUMAN|HGNC=626|UniProtKB=P07741,HUMAN|HGNC=7892|UniProtKB=P00491,HUMAN|HGNC=5157|UniProtKB=P00492</td><td>transferase activity, transferring pentosyl groups</td></tr>
<tr><td>GO:0006793</td><td>HUMAN|HGNC=13420|UniProtKB=Q86TP1</td><td>phosphorus metabolic process</td></tr>
<tr><td>GO:0045453</td><td>HUMAN|HGNC=29884|UniProtKB=Q8TF42,HUMAN|HGNC=11283|UniProtKB=P12931</td><td>bone resorption</td></tr>
<tr><td>GO:0050660</td><td>HUMAN|HGNC=13397|UniProtKB=Q9UHQ9,HUMAN|HGNC=2898|UniProtKB=P09622,HUMAN|HGNC=121|UniProtKB=O15254,HUMAN|HGNC=119|UniProtKB=Q15067,HUMAN|HGNC=2873|UniProtKB=P00387,HUMAN|HGNC=4623|UniProtKB=P00390</td><td>flavin adenine dinucleotide binding</td></tr>
<tr><td>GO:0006979</td><td>HUMAN|HGNC=17169|UniProtKB=Q13162,HUMAN|HGNC=5046|UniProtKB=P52272,HUMAN|HGNC=1516|UniProtKB=P04040,HUMAN|HGNC=4553|UniProtKB=P07203,HUMAN|HGNC=612|UniProtKB=P05090</td><td>response to oxidative stress</td></tr>
<tr><td>GO:0007189</td><td>HUMAN|HGNC=4381|UniProtKB=Q14344,HUMAN|HGNC=237|UniProtKB=O43306</td><td>adenylate cyclase-activating G protein-coupled receptor signaling pathway</td></tr>
<tr><td>GO:0048193</td><td>HUMAN|HGNC=31332|UniProtKB=Q6IQ22,HUMAN|HGNC=7007|UniProtKB=P61006,HUMAN|HGNC=11172|UniProtKB=Q13596</td><td>Golgi vesicle transport</td></tr>
<tr><td>PC00226</td><td>HUMAN|HGNC=24335|UniProtKB=Q99627,HUMAN|HGNC=28920|UniProtKB=Q5SW79,HUMAN|HGNC=12851|UniProtKB=P62258,HUMAN|HGNC=18271|UniProtKB=Q8N8S7,HUMAN|HGNC=12849|UniProtKB=P31946,HUMAN|HGNC=23416|UniProtKB=Q641Q2,HUMAN|HGNC=25316|UniProtKB=Q5VZ19,HUMAN|HGNC=19974|UniProtKB=Q8IUC4,HUMAN|HGNC=2905|UniProtKB=O14490,HUMAN|HGNC=11320|UniProtKB=Q8IZP0,HUMAN|HGNC=15606|UniP</td><td>scaffold/adaptor protein</td></tr>
</table>

<table>
<tr><td></td><td>rotKB=Q9UHA4,HUMAN|HGNC=711|UniProtKB=P49407,HUMAN|HGNC=12852|UniProtKB=P61981,HUMAN|HGNC=7665|UniProtKB=O43639,HUMAN|HGNC=13759|UniProtKB=Q7L576,HUMAN|HGNC=28817|UniProtKB=Q96AG4,HUMAN|HGNC=22934|UniProtKB=Q8NAB2,HUMAN|HGNC=7237|UniProtKB=Q9Y6F6,HUMAN|HGNC=11358|UniProtKB=O75886,HUMAN|HGNC=15842|UniProtKB=Q96RT1,HUMAN|HGNC=11357|UniProtKB=Q92783,HUMAN|HGNC=29557|UniProtKB=Q0ZGT2,HUMAN|HGNC=29890|UniProtKB=Q86UT6,HUMAN|HGNC=15687|UniProtKB=O75563,HUMAN|HGNC=30377|UniProtKB=Q14160,HUMAN|HGNC=7219|UniProtKB=Q00013,HUMAN|HGNC=12652|UniProtKB=P50552,HUMAN|HGNC=21883|UniProtKB=Q9H3E2,HUMAN|HGNC=26614|UniProtKB=Q8NB46,HUMAN|HGNC=12853|UniProtKB=Q04917,HUMAN|HGNC=10464|UniProtKB=Q15404,HUMAN|HGNC=1226|UniProtKB=O14908,HUMAN|HGNC=12854|UniProtKB=P27348,HUMAN|HGNC=369|UniProtKB=Q9UKA4,HUMAN|HGNC=2362|UniProtKB=P46108,HUMAN|HGNC=16643|UniProtKB=Q14BN4,HUMAN|HGNC=14977|UniProtKB=Q9Y5W7,HUMAN|HGNC=18325|UniProtKB=Q2WGN9,HUMAN|HGNC=22880|UniProtKB=Q96JG8,HUMAN|HGNC=11172|UniProtKB=Q13596,HUMAN|HGNC=4036|UniProtKB=O15117,HUMAN|HGNC=6529|UniProtKB=Q13094,HUMAN|HGNC=12855|UniProtKB=P63104,HUMAN|HGNC=9847|UniProtKB=P43487,HUMAN|HGNC=2363|UniProtKB=P46109,HUMAN|HGNC=10840|UniProtKB=P29353,HUMAN|HGNC=21295|UniProtKB=Q8TE68</td><td></td></tr>
<tr><td>GO:0005930</td><td>HUMAN|HGNC=30391|UniProtKB=Q9UG01,HUMAN|HGNC=17060|UniProtKB=Q92845</td><td>axoneme</td></tr>
<tr><td>PC00105</td><td>HUMAN|HGNC=19155|UniProtKB=P57773</td><td>gap junction</td></tr>
<tr><td>GO:0120039</td><td>HUMAN|HGNC=12690|UniProtKB=P09327</td><td>plasma membrane bounded cell projection morphogenesis</td></tr>
<tr><td>GO:0005126</td><td>HUMAN|HGNC=19100|UniProtKB=Q5VWK5,HUMAN|HGNC=11766|UniProtKB=P01137</td><td>cytokine receptor binding</td></tr>
<tr><td>GO:0009132</td><td>HUMAN|HGNC=362|UniProtKB=P54819,HUMAN|HGNC=17376|UniProtKB=Q9UIJ7</td><td>nucleoside diphosphate metabolic process</td></tr>
<tr><td>GO:0045296</td><td>HUMAN|HGNC=15902|UniProtKB=Q8IXH8,HUMAN|HGNC=3244|UniProtKB=Q9NZN3,HUMAN|HGNC=6207|UniProtKB=P14923,HUMAN|HGNC=533|UniProtKB=P04083,HUMAN|HGNC=14265|UniProtKB=Q86UP0,HUMAN|HGNC=24634|UniProtKB=Q9UBC2,HUMAN|HGNC=9069|UniProtKB=Q15149,HUMAN|HGNC=3419|UniProtKB=P42566</td><td>cadherin binding</td></tr>
<tr><td>GO:0009112</td><td>HUMAN|HGNC=12405|UniProtKB=P02766</td><td>nucleobase metabolic process</td></tr>
<tr><td>GO:0001664</td><td>HUMAN|HGNC=4395|UniProtKB=P19086,HUMAN|HGNC=711|UniProtKB=P49407,HUMAN|HGNC=4384|UniProtKB=P63096,HUMAN|HGNC=4381|UniProtKB=Q14344,HUMAN|HGNC=4387|UniProtKB=P08754,HUMAN|HGNC=4385|UniProtKB=P04899,HUMAN|HGNC=4393|UniProtKB=P11488,HUMAN|HGNC=4390|UniProtKB=P50148</td><td>G protein-coupled receptor binding</td></tr>
<tr><td>GO:0030968</td><td>HUMAN|HGNC=463|UniProtKB=Q9UKV5,HUMAN|HGNC=1455|UniProtKB=P27797,HUMAN|HGNC=5238|UniProtKB=P11021,HUMAN|HGNC=25035|UniProtKB=Q5VVQ6,HUMAN|HGNC=1473|UniProtKB=P27824,HUMAN|HGNC=17943|UniProtKB=Q9GZP9</td><td>endoplasmic reticulum unfolded protein response</td></tr>
<tr><td>GO:0030099</td><td>HUMAN|HGNC=13937|UniProtKB=O95866</td><td>myeloid cell differentiation</td></tr>
</table>

GO:0004857	HUMAN|HGNC=607|UniProtKB=P02654,HUMAN|HGNC=7618|UniProtKB=O14974,HUMAN|HGNC=871|UniProtKB=Q9UII2,HUMAN|HGNC=533|UniProtKB=P04083	enzyme inhibitor activity
GO:0006047	HUMAN|HGNC=4242|UniProtKB=O94808	UDP-N-acetylglucosamine metabolic process
GO:0000289	HUMAN|HGNC=29190|UniProtKB=Q9UPQ9	nuclear-transcribed mRNA poly(A) tail shortening
GO:0045332	HUMAN|HGNC=13531|UniProtKB=Q9Y2Q0	phospholipid translocation
GO:0030017	HUMAN|HGNC=11873|UniProtKB=Q9NYL9	sarcomere
GO:0006417	HUMAN|HGNC=27424|UniProtKB=Q5T481,HUMAN|HGNC=13759|UniProtKB=Q7L576,HUMAN|HGNC=9583|UniProtKB=P26599,HUMAN|HGNC=17894|UniProtKB=Q14966	regulation of translation
GO:0005548	HUMAN|HGNC=1869|UniProtKB=P11597	phospholipid transporter activity
GO:0006123	HUMAN|HGNC=2265|UniProtKB=P13073,HUMAN|HGNC=2267|UniProtKB=P20674,HUMAN|HGNC=19986|UniProtKB=P99999,HUMAN|HGNC=2292|UniProtKB=P15954	mitochondrial electron transport, cytochrome c to oxygen
GO:0010648	HUMAN|HGNC=9855|UniProtKB=P62834,HUMAN|HGNC=9857|UniProtKB=P61224	negative regulation of cell communication
GO:0006641	HUMAN|HGNC=607|UniProtKB=P02654,HUMAN|HGNC=1869|UniProtKB=P11597,HUMAN|HGNC=603|UniProtKB=P04114	triglyceride metabolic process
GO:0044237	HUMAN|HGNC=11179|UniProtKB=P00441,HUMAN|HGNC=8527|UniProtKB=P55809	cellular metabolic process
GO:0006635	HUMAN|HGNC=4801|UniProtKB=P40939,HUMAN|HGNC=121|UniProtKB=O15254,HUMAN|HGNC=94|UniProtKB=Q9BWD1,HUMAN|HGNC=119|UniProtKB=Q15067,HUMAN|HGNC=3151|UniProtKB=P30084,HUMAN|HGNC=82|UniProtKB=P09110	fatty acid beta-oxidation
GO:0045944	HUMAN|HGNC=25473|UniProtKB=Q9P2N6,HUMAN|HGNC=7670|UniProtKB=Q9Y6Q9,HUMAN|HGNC=7133|UniProtKB=O14686,HUMAN|HGNC=13726|UniProtKB=Q8NEZ4,HUMAN|HGNC=30025|UniProtKB=Q5VV67,HUMAN|HGNC=9866|UniProtKB=P13631,HUMAN|HGNC=6767|UniProtKB=Q15797,HUMAN|HGNC=23688|UniProtKB=Q86UU0,HUMAN|HGNC=17305|UniProtKB=Q9Y6J9,HUMAN|HGNC=6207|UniProtKB=P14923,HUMAN|HGNC=7967|UniProtKB=Q96RI1,HUMAN|HGNC=8647|UniProtKB=Q15365,HUMAN|HGNC=14451|UniProtKB=Q9BQK8,HUMAN|HGNC=5048|UniProtKB=Q00839,HUMAN|HGNC=7547|UniProtKB=P10243,HUMAN|HGNC=16272|UniProtKB=Q96JK9,HUMAN|HGNC=10055|UniProtKB=Q8N5U6,HUMAN|HGNC=7910|UniProtKB=P06748,HUMAN|HGNC=7002|UniProtKB=A6NDR6	positive regulation of transcription by RNA polymerase II
PC00207	HUMAN|HGNC=3778|UniProtKB=P02751,HUMAN|HGNC=3662|UniProtKB=P02675,HUMAN|HGNC=8078|UniProtKB=O95158,HUMAN|HGNC=11891|UniProtKB=P05452,HUMAN|HGNC=3625|UniProtKB=O75636,HUMAN|HGNC=11756|UniProtKB=Q03403,HUMAN|HGNC=4374|UniProtKB=O60234,HUMAN|HGNC=3624|UniProtKB=Q15485,HUMAN|HGNC=3694|UniProtKB=P02679	intercellular signal molecule
GO:0030833	HUMAN|HGNC=3338|UniProtKB=Q14247,HUMAN|HGNC=2695|UniProtKB=Q16643,HUMAN|HGNC=8881|UniProtKB=P07737,HUMAN|HGNC=2696|UniProtKB=Q9UJU6,HUMAN|HGNC=4844|UniProtKB=P14317,HUMAN|HGNC=18304|UniProtKB=Q14019,HUMAN|HGNC=21035|UniProtKB=Q8N392	regulation of actin filament polymerization

GO:0009117	HUMAN|HGNC=16369|UniProtKB=Q99497	nucleotide metabolic process
GO:0048471	HUMAN|HGNC=8504|UniProtKB=Q969R2,HUMAN|HGNC=5253|UniProtKB=P07900,HUMAN|HGNC=5258|UniProtKB=P08238,HUMAN|HGNC=8783|UniProtKB=Q08499,HUMAN|HGNC=3244|UniProtKB=Q9NZN3,HUMAN|HGNC=10496|UniProtKB=P06703,HUMAN|HGNC=9069|UniProtKB=Q15149	perinuclear region of cytoplasm
PC00257	HUMAN|HGNC=18423|UniProtKB=O75140,HUMAN|HGNC=25694|UniProtKB=Q8TC07,HUMAN|HGNC=6111|UniProtKB=Q13576,HUMAN|HGNC=12363|UniProtKB=P49815,HUMAN|HGNC=14261|UniProtKB=Q9NS28,HUMAN|HGNC=20331|UniProtKB=Q14644,HUMAN|HGNC=16925|UniProtKB=Q96P48,HUMAN|HGNC=17073|UniProtKB=Q9UNA1,HUMAN|HGNC=25571|UniProtKB=Q9NVG8,HUMAN|HGNC=673|UniProtKB=Q07960,HUMAN|HGNC=21035|UniProtKB=Q8N392	GTPase-activating protein
GO:0005515	HUMAN|HGNC=26486|UniProtKB=Q6PJW8,HUMAN|HGNC=2949|UniProtKB=Q8TD57,HUMAN|HGNC=18587|UniProtKB=O94901,HUMAN|HGNC=12652|UniProtKB=P50552,HUMAN|HGNC=18271|UniProtKB=Q8N8S7,HUMAN|HGNC=6561|UniProtKB=P09382,HUMAN|HGNC=4398|UniProtKB=P62879,HUMAN|HGNC=4823|UniProtKB=P69905,HUMAN|HGNC=4829|UniProtKB=P02042,HUMAN|HGNC=10496|UniProtKB=P06703,HUMAN|HGNC=20731|UniProtKB=Q9HAV0,HUMAN|HGNC=12648|UniProtKB=Q9P0L0,HUMAN|HGNC=11320|UniProtKB=Q8IZP0,HUMAN|HGNC=15476|UniProtKB=P63167,HUMAN|HGNC=4827|UniProtKB=P68871,HUMAN|HGNC=2951|UniProtKB=Q9C0G6,HUMAN|HGNC=4831|UniProtKB=P69891,HUMAN|HGNC=2952|UniProtKB=Q96JB1,HUMAN|HGNC=16391|UniProtKB=Q9H257,HUMAN|HGNC=10899|UniProtKB=P63208,HUMAN|HGNC=12649|UniProtKB=O95292,HUMAN|HGNC=14452|UniProtKB=Q9NYG5,HUMAN|HGNC=4396|UniProtKB=P62873,HUMAN|HGNC=6091|UniProtKB=P06213	protein binding
GO:0006342	HUMAN|HGNC=20508|UniProtKB=Q8IUE6,HUMAN|HGNC=4716|UniProtKB=P16403,HUMAN|HGNC=4718|UniProtKB=P10412,HUMAN|HGNC=4719|UniProtKB=P16401	chromatin silencing
PC00175	HUMAN|HGNC=2265|UniProtKB=P13073,HUMAN|HGNC=6834|UniProtKB=P27338,HUMAN|HGNC=9756|UniProtKB=O00391,HUMAN|HGNC=2288|UniProtKB=P14406,HUMAN|HGNC=2267|UniProtKB=P20674,HUMAN|HGNC=2295|UniProtKB=P00450,HUMAN|HGNC=2292|UniProtKB=P15954	oxidase
GO:0042177	HUMAN|HGNC=11820|UniProtKB=P01033	negative regulation of protein catabolic process
GO:0001786	HUMAN|HGNC=11510|UniProtKB=Q8N9I0,HUMAN|HGNC=544|UniProtKB=P08133,HUMAN|HGNC=11512|UniProtKB=Q9H2B2	phosphatidylserine binding
GO:0036002	HUMAN|HGNC=5048|UniProtKB=Q00839	pre-mRNA binding
GO:0000808	HUMAN|HGNC=26200|UniProtKB=Q9H668	origin recognition complex
GO:0010817	HUMAN|HGNC=253|UniProtKB=P11766,HUMAN|HGNC=251|UniProtKB=P00326,HUMAN|HGNC=8569|UniProtKB=P29122,HUMAN|HGNC=255|UniProtKB=P28332	regulation of hormone levels
GO:0007134	HUMAN|HGNC=7326|UniProtKB=P20585,HUMAN|HGNC=20473|UniProtKB=Q9BX63,HUMAN|HGNC=23845|UniProtKB=Q8IY92	meiotic telophase I

GO:0000978	HUMAN|HGNC=33108|UniProtKB=Q6NX45,HUMAN|HGNC=14008|UniProtKB=Q9BW11,HUMAN|HGNC=13061|UniProtKB=Q14587,HUMAN|HGNC=18411|UniProtKB=Q3KNW1,HUMAN|HGNC=14010|UniProtKB=Q8IWI9,HUMAN|HGNC=23708|UniProtKB=Q6ZR52,HUMAN|HGNC=11364|UniProtKB=P40763,HUMAN|HGNC=6702|UniProtKB=Q32MZ4,HUMAN|HGNC=29084|UniProtKB=O75123,HUMAN|HGNC=26427|UniProtKB=Q96ND8,HUMAN|HGNC=17801|UniProtKB=Q8NHY6,HUMAN|HGNC=11594|UniProtKB=Q96SF7,HUMAN|HGNC=9459|UniProtKB=Q92786,HUMAN|HGNC=9866|UniProtKB=P13631,HUMAN|HGNC=6767|UniProtKB=Q15797,HUMAN|HGNC=13109|UniProtKB=P17038,HUMAN|HGNC=29989|UniProtKB=Q86YP4,HUMAN|HGNC=51815|UniProtKB=A0A1W2PPM1,HUMAN|HGNC=3815|UniProtKB=Q12951,HUMAN|HGNC=7967|UniProtKB=Q96RI1,HUMAN|HGNC=11590|UniProtKB=Q16650,HUMAN|HGNC=5048|UniProtKB=Q00839,HUMAN|HGNC=7547|UniProtKB=P10243,HUMAN|HGNC=7002|UniProtKB=A6NDR6	RNA polymerase II cis-regulatory region sequence-specific DNA binding
GO:0003723	HUMAN|HGNC=3189|UniProtKB=P68104,HUMAN|HGNC=30646|UniProtKB=Q7KZF4,HUMAN|HGNC=18018|UniProtKB=Q8IX18,HUMAN|HGNC=5043|UniProtKB=P31942,HUMAN|HGNC=39025|UniProtKB=Q9QC07,HUMAN|HGNC=4622|UniProtKB=Q8IYD1,HUMAN|HGNC=4621|UniProtKB=P15170,HUMAN|HGNC=3200|UniProtKB=Q5VTE0,HUMAN|HGNC=10353|UniProtKB=P36578,HUMAN|HGNC=17196|UniProtKB=Q8NG08,HUMAN|HGNC=20471|UniProtKB=O95777,HUMAN|HGNC=3300|UniProtKB=P63241,HUMAN|HGNC=2678|UniProtKB=P14868,HUMAN|HGNC=3214|UniProtKB=P13639,HUMAN|HGNC=17821|UniProtKB=O00148,HUMAN|HGNC=10363|UniProtKB=P18124,HUMAN|HGNC=15861|UniProtKB=Q9H5Z1,HUMAN|HGNC=30796|UniProtKB=Q9Y3F4,HUMAN|HGNC=5036|UniProtKB=Q14103,HUMAN|HGNC=12420|UniProtKB=P49411,HUMAN|HGNC=7910|UniProtKB=P06748,HUMAN|HGNC=10396|UniProtKB=P62249,HUMAN|HGNC=10305|UniProtKB=P50914	RNA binding
GO:0008373	HUMAN|HGNC=10869|UniProtKB=Q92185	sialyltransferase activity
GO:0061135	HUMAN|HGNC=9568|UniProtKB=Q06323	endopeptidase regulator activity
GO:0030234	HUMAN|HGNC=4312|UniProtKB=P48507	enzyme regulator activity
GO:0006935	HUMAN|HGNC=10046|UniProtKB=P12724,HUMAN|HGNC=10045|UniProtKB=P10153	chemotaxis
GO:0007018	HUMAN|HGNC=2951|UniProtKB=Q9C0G6,HUMAN|HGNC=6317|UniProtKB=O43896,HUMAN|HGNC=17060|UniProtKB=Q92845	microtubule-based movement
GO:0005198	HUMAN|HGNC=12407|UniProtKB=P68366,HUMAN|HGNC=1117|UniProtKB=Q9UPA5,HUMAN|HGNC=705|UniProtKB=O15144,HUMAN|HGNC=12410|UniProtKB=Q9NY65,HUMAN|HGNC=20778|UniProtKB=P07437,HUMAN|HGNC=16257|UniProtKB=Q9H4B7,HUMAN|HGNC=9069|UniProtKB=Q15149,HUMAN|HGNC=9086|UniProtKB=P60201,HUMAN|HGNC=20766|UniProtKB=Q71U36,HUMAN|HGNC=20774|UniProtKB=P04350,HUMAN|HGNC=13406|UniProtKB=Q9Y6V0,HUMAN|HGNC=20771|UniProtKB=P68371	structural molecule activity
GO:0010498	HUMAN|HGNC=20635|UniProtKB=Q14997,HUMAN|HGNC=9568|UniProtKB=Q06323	proteasomal protein catabolic process
GO:0006099	HUMAN|HGNC=118|UniProtKB=Q99798,HUMAN|HGNC=2422|UniProtKB=O75390,HUMAN|HGNC=117|UniProtKB=P21399,HUMAN	tricarboxylic acid cycle

	|HGNC=6970|UniProtKB=P40925,HUMAN|HGNC=5386|UniProtKB=P51553	
GO:0030594	HUMAN|HGNC=16768|UniProtKB=O60391	neurotransmitter receptor activity
GO:0014066	HUMAN|HGNC=6091|UniProtKB=P06213	regulation of phosphatidylinositol 3-kinase signaling
GO:0005516	HUMAN|HGNC=15720|UniProtKB=Q13033,HUMAN|HGNC=1459|UniProtKB=Q14012,HUMAN|HGNC=11424|UniProtKB=O43815,HUMAN|HGNC=6111|UniProtKB=Q13576,HUMAN|HGNC=9314|UniProtKB=Q08209	calmodulin binding
GO:0004520	HUMAN|HGNC=23845|UniProtKB=Q8IY92	endodeoxyribonuclease activity
GO:0055074	HUMAN|HGNC=15999|UniProtKB=Q9NZV5	calcium ion homeostasis
GO:0008233	HUMAN|HGNC=24437|UniProtKB=Q96KP4,HUMAN|HGNC=20635|UniProtKB=Q14997,HUMAN|HGNC=20675|UniProtKB=Q96KN2	peptidase activity
GO:0003743	HUMAN|HGNC=3282|UniProtKB=P60842,HUMAN|HGNC=3265|UniProtKB=P05198,HUMAN|Gene=EIF2S3L|UniProtKB=Q2VIR3,HUMAN|HGNC=3297|UniProtKB=P78344	translation initiation factor activity
GO:0031623	HUMAN|HGNC=1406|UniProtKB=Q9Y698,HUMAN|HGNC=3757|UniProtKB=O75955,HUMAN|HGNC=711|UniProtKB=P49407,HUMAN|HGNC=2974|UniProtKB=P50570,HUMAN|HGNC=29125|UniProtKB=Q9UQ16	receptor internalization
GO:0046165	HUMAN|HGNC=29035|UniProtKB=O43314,HUMAN|HGNC=16369|UniProtKB=Q99497,HUMAN|HGNC=2689|UniProtKB=P09172	alcohol biosynthetic process
GO:0071222	HUMAN|HGNC=9240|UniProtKB=P02775,HUMAN|HGNC=2761|UniProtKB=P59665,HUMAN|HGNC=8862|UniProtKB=P10720,HUMAN|HGNC=8861|UniProtKB=P02776,HUMAN|HGNC=1628|UniProtKB=P08571,HUMAN|HGNC=6517|UniProtKB=P18428	cellular response to lipopolysaccharide
GO:0048488	HUMAN|HGNC=2974|UniProtKB=P50570,HUMAN|HGNC=11510|UniProtKB=Q8N9I0,HUMAN|HGNC=29125|UniProtKB=Q9UQ16,HUMAN|HGNC=11138|UniProtKB=P37840	synaptic vesicle endocytosis
GO:0006312	HUMAN|HGNC=7326|UniProtKB=P20585	mitotic recombination
GO:0004843	HUMAN|HGNC=7106|UniProtKB=P54252,HUMAN|HGNC=23077|UniProtKB=Q96FW1,HUMAN|HGNC=25648|UniProtKB=Q8N5J2,HUMAN|HGNC=20063|UniProtKB=Q86T82,HUMAN|HGNC=12628|UniProtKB=P45974,HUMAN|HGNC=25035|UniProtKB=Q5VVQ6,HUMAN|HGNC=13485|UniProtKB=Q9BXU7,HUMAN|HGNC=20076|UniProtKB=Q96K76	thiol-dependent ubiquitin-specific protease activity
GO:0014065	HUMAN|HGNC=6091|UniProtKB=P06213	phosphatidylinositol 3-kinase signaling
GO:1902494	HUMAN|HGNC=28422|UniProtKB=Q8NCE0,HUMAN|HGNC=4395|UniProtKB=P19086,HUMAN|HGNC=4384|UniProtKB=P63096,HUMAN|HGNC=4398|UniProtKB=P62879,HUMAN|HGNC=4387|UniProtKB=P08754,HUMAN|HGNC=20731|UniProtKB=Q9HAV0,HUMAN|HGNC=4385|UniProtKB=P04899,HUMAN|HGNC=4390|UniProtKB=P50148,HUMAN|HGNC=3350|UniProtKB=P06733,HUMAN|HGNC=3353|UniProtKB=P09104,HUMAN|HGNC=4312|UniProtKB=P48507,HUMAN|HGNC=4381|UniProtKB=Q14344,HUMAN|HGNC=3354|UniProtKB=P13929,HUMAN|HGNC=4393|UniProtKB=P11488,HUMAN|HGNC=4396|UniProtKB=P62873	catalytic complex

GO:0043588	HUMAN|HGNC=1504|UniProtKB=P42574,HUMAN|HGNC=533|UniProtKB=P04083	skin development
GO:0009966	HUMAN|HGNC=1692|UniProtKB=P08962,HUMAN|HGNC=2444|UniProtKB=P41240	regulation of signal transduction
GO:0055072	HUMAN|HGNC=2295|UniProtKB=P00450	iron ion homeostasis
GO:0038023	HUMAN|HGNC=8601|UniProtKB=Q06141,HUMAN|HGNC=4243|UniProtKB=P56159,HUMAN|HGNC=30013|UniProtKB=Q96PD5,HUMAN|HGNC=7228|UniProtKB=P22897,HUMAN|HGNC=4244|UniProtKB=O00451	signaling receptor activity
GO:0006796	HUMAN|HGNC=29035|UniProtKB=O43314,HUMAN|HGNC=28883|UniProtKB=Q9H2U2,HUMAN|HGNC=4242|UniProtKB=O94808,HUMAN|HGNC=4456|UniProtKB=P43304	phosphate-containing compound metabolic process
GO:0019216	HUMAN|HGNC=607|UniProtKB=P02654	regulation of lipid metabolic process
PC00151	HUMAN|HGNC=12648|UniProtKB=Q9P0L0,HUMAN|HGNC=10458|UniProtKB=Q92681,HUMAN|HGNC=1663|UniProtKB=P16671,HUMAN|HGNC=6499|UniProtKB=P11279,HUMAN|HGNC=20726|UniProtKB=Q8WVM8,HUMAN|HGNC=11446|UniProtKB=O00186,HUMAN|HGNC=6501|UniProtKB=P13473,HUMAN|HGNC=11445|UniProtKB=Q15833,HUMAN|HGNC=11510|UniProtKB=Q8N9I0,HUMAN|HGNC=12649|UniProtKB=O95292,HUMAN|HGNC=11138|UniProtKB=P37840,HUMAN|HGNC=11512|UniProtKB=Q9H2B2,HUMAN|HGNC=8515|UniProtKB=Q9HC10	membrane trafficking regulatory protein
GO:0033617	HUMAN|HGNC=26801|UniProtKB=Q8N8Q8	mitochondrial cytochrome c oxidase assembly
GO:0048519	HUMAN|HGNC=2367|UniProtKB=P02741,HUMAN|HGNC=8087|UniProtKB=P29728,HUMAN|HGNC=584|UniProtKB=P02743	negative regulation of biological process
GO:1902905	HUMAN|HGNC=12754|UniProtKB=O75083,HUMAN|HGNC=8881|UniProtKB=P07737	positive regulation of supramolecular fiber organization
GO:0099565	HUMAN|HGNC=15474|UniProtKB=Q9Y566,HUMAN|HGNC=2905|UniProtKB=O14490,HUMAN|HGNC=14294|UniProtKB=Q9BYB0	chemical synaptic transmission, postsynaptic
GO:0070371	HUMAN|HGNC=1681|UniProtKB=P16070,HUMAN|HGNC=10632|UniProtKB=P13501	ERK1 and ERK2 cascade
GO:0070507	HUMAN|HGNC=9847|UniProtKB=P43487	regulation of microtubule cytoskeleton organization
GO:0047372	HUMAN|HGNC=13921|UniProtKB=O95870	acylglycerol lipase activity
GO:0006810	HUMAN|HGNC=14685|UniProtKB=Q9Y624,HUMAN|HGNC=4187|UniProtKB=P02774,HUMAN|HGNC=316|UniProtKB=P43652,HUMAN|HGNC=317|UniProtKB=P02771,HUMAN|HGNC=399|UniProtKB=P02768	transport
PC00113	HUMAN|HGNC=21740|UniProtKB=Q6ZV73,HUMAN|HGNC=16854|UniProtKB=Q9Y4G8,HUMAN|HGNC=9879|UniProtKB=Q7LDG7,HUMAN|HGNC=21213|UniProtKB=Q5TH69,HUMAN|HGNC=2988|UniProtKB=Q92608,HUMAN|HGNC=30282|UniProtKB=Q3MIN7,HUMAN|HGNC=15607|UniProtKB=Q14155,HUMAN|HGNC=9504|UniProtKB=O43739,HUMAN|HGNC=9878|UniProtKB=O95267,HUMAN|HGNC=29374|UniProtKB=Q76NI1,HUMAN|HGNC=23479|UniProtKB=Q96BY6,HUMAN|HGNC=21726|UniProtKB=Q96PE2,HUMAN|HGNC=4814|UniProtKB=O60229,HUMAN|HGNC=25516|UniProtKB=Q8TER5,HUMAN|HGNC=14545|UniProtKB=Q8IV61	guanyl-nucleotide exchange factor

PC00119	HUMAN|HGNC=9459|UniProtKB=Q92786,HUMAN|HGNC=51815|UniProtKB=A0A1W2PPM1,HUMAN|HGNC=19347|UniProtKB=O14529,HUMAN|HGNC=15935|UniProtKB=Q9H4I2,HUMAN|HGNC=7002|UniProtKB=A6NDR6	homeodomain transcription factor
GO:0031012	HUMAN|HGNC=4439|UniProtKB=P07359,HUMAN|HGNC=5468|UniProtKB=P35858,HUMAN|HGNC=3011|UniProtKB=Q07507,HUMAN|HGNC=7516|UniProtKB=Q9HC84,HUMAN|HGNC=13572|UniProtKB=Q9Y6R7,HUMAN|HGNC=2761|UniProtKB=P59665,HUMAN|HGNC=7515|UniProtKB=P98088,HUMAN|HGNC=11771|UniProtKB=Q15582,HUMAN|HGNC=5164|UniProtKB=Q9Y251,HUMAN|HGNC=11820|UniProtKB=P01033,HUMAN|HGNC=7060|UniProtKB=P08493,HUMAN|HGNC=12726|UniProtKB=P04275,HUMAN|HGNC=2313|UniProtKB=P22792	extracellular matrix
GO:0017157	HUMAN|HGNC=31332|UniProtKB=Q6IQ22,HUMAN|HGNC=7007|UniProtKB=P61006,HUMAN|HGNC=9855|UniProtKB=P62834,HUMAN|HGNC=7641|UniProtKB=P54920,HUMAN|HGNC=9164|UniProtKB=Q99719,HUMAN|HGNC=9778|UniProtKB=P20337,HUMAN|HGNC=11510|UniProtKB=Q8N9I0,HUMAN|HGNC=533|UniProtKB=P04083,HUMAN|HGNC=9857|UniProtKB=P61224,HUMAN|HGNC=11512|UniProtKB=Q9H2B2	regulation of exocytosis
GO:0048813	HUMAN|HGNC=3338|UniProtKB=Q14247,HUMAN|HGNC=15474|UniProtKB=Q9Y566,HUMAN|HGNC=2695|UniProtKB=Q16643,HUMAN|HGNC=2696|UniProtKB=Q9UJU6,HUMAN|HGNC=4844|UniProtKB=P14317,HUMAN|HGNC=14294|UniProtKB=Q9BYB0	dendrite morphogenesis
GO:0012507	HUMAN|HGNC=10534|UniProtKB=Q9NR31,HUMAN|HGNC=10701|UniProtKB=Q15436	ER to Golgi transport vesicle membrane
GO:0007034	HUMAN|HGNC=20347|UniProtKB=Q9H9C1	vacuolar transport
GO:0010324	HUMAN|HGNC=5575|UniProtKB=A0A0B4J1V2,HUMAN|HGNC=607|UniProtKB=P02654,HUMAN|HGNC=9784|UniProtKB=P61020,HUMAN|HGNC=5645|UniProtKB=A0A0C4DH34,HUMAN|HGNC=5549|UniProtKB=A0A0C4DH31,HUMAN|HGNC=555|UniProtKB=O43747,HUMAN|HGNC=5554|UniProtKB=P01743,HUMAN|HGNC=5480|UniProtKB=P01880,HUMAN|HGNC=5659|UniProtKB=A0A0C4DH38,HUMAN|HGNC=5634|UniProtKB=A0A075B7F0,HUMAN|HGNC=38476|UniProtKB=B9A064,HUMAN|HGNC=2090|UniProtKB=P09496,HUMAN|HGNC=5861|UniProtKB=A0M8Q6,HUMAN|HGNC=5647|UniProtKB=A0A087WSY4,HUMAN|HGNC=5563|UniProtKB=A0A075B7D0,HUMAN|HGNC=5856|UniProtKB=P0DOY2,HUMAN|HGNC=1406|UniProtKB=Q9Y698,HUMAN|HGNC=3757|UniProtKB=O75955,HUMAN|HGNC=5606|UniProtKB=P01763,HUMAN|HGNC=9785|UniProtKB=P51148,HUMAN|HGNC=9801|UniProtKB=P63000,HUMAN|HGNC=5576|UniProtKB=P01817,HUMAN|HGNC=5528|UniProtKB=P01861,HUMAN|HGNC=5541|UniProtKB=P01871,HUMAN|HGNC=5585|UniProtKB=A0A0C4DH32,HUMAN|HGNC=9782|UniProtKB=P61018,HUMAN|HGNC=9802|UniProtKB=P15153,HUMAN|HGNC=5551|UniProtKB=A0A0C4DH33,HUMAN|HGNC=3244|UniProtKB=Q9NZN3,HUMAN|HGNC=11510|UniProtKB=Q8N9I0,HUMAN|HGNC=7228|UniProtKB=P22897,HUMAN|HGNC=5526|UniProtKB=P01859,HUMAN|HGNC=5657|UniProtKB=P0DP08,HUMAN|Ensembl=ENSG00000282639|UniProtKB=A0A0J9YX35,HUMAN|HGNC=19760|UniProtKB=Q96T51,HUMAN|Gene=HV309_HUMAN|UniProtKB=P01782,HUMAN|HGNC=18756|UniProtKB=Q9BYZ6,HUMAN|HGNC=5654|UniProtKB=P01825,HUMAN|HGNC=5553|UniProtKB=A0A0	membrane invagination

<table>
<tr><td></td><td>A0MS14,HUMAN|Gene=HV108_HUMAN|UniProtKB=P0DP01,HUMAN|HGNC=5624|UniProtKB=A0A0B4J1X5,HUMAN|HGNC=5620|UniProtKB=P01780,HUMAN|HGNC=23186|UniProtKB=Q14677,HUMAN|HGNC=5598|UniProtKB=A0A0C4DH35,HUMAN|HGNC=5580|UniProtKB=P01762,HUMAN|HGNC=5633|UniProtKB=A0A075B7D8,HUMAN|HGNC=5582|UniProtKB=A0A0B4J1V0,HUMAN|HGNC=5478|UniProtKB=P01876,HUMAN|HGNC=26068|UniProtKB=Q6IAA8,HUMAN|HGNC=5617|UniProtKB=A0A075B6Q5,HUMAN|HGNC=711|UniProtKB=P49407,HUMAN|HGNC=5594|UniProtKB=P0DP03,HUMAN|HGNC=5655|UniProtKB=A0A0C4DH41,HUMAN|HGNC=5636|UniProtKB=A0A075B7B8,HUMAN|HGNC=5586|UniProtKB=A0A0B4J1V1,HUMAN|HGNC=5581|UniProtKB=P01766,HUMAN|HGNC=5622|UniProtKB=A0A0B4J1Y9,HUMAN|HGNC=5652|UniProtKB=A0A075B6R2,HUMAN|HGNC=5593|UniProtKB=P0DP02,HUMAN|HGNC=11138|UniProtKB=P37840,HUMAN|HGNC=3419|UniProtKB=P42566,HUMAN|HGNC=5479|UniProtKB=P01877,HUMAN|HGNC=561|UniProtKB=O95782,HUMAN|HGNC=5619|UniProtKB=A0A0C4DH42,HUMAN|HGNC=5596|UniProtKB=P01772,HUMAN|HGNC=5550|UniProtKB=P23083,HUMAN|HGNC=5604|UniProtKB=A0A0B4J1X8,HUMAN|HGNC=570|UniProtKB=P53677,HUMAN|HGNC=2974|UniProtKB=P50570,HUMAN|HGNC=5525|UniProtKB=P01857,HUMAN|HGNC=5662|UniProtKB=A0A0B4J1U7,HUMAN|HGNC=24634|UniProtKB=Q9UBC2,HUMAN|HGNC=5601|UniProtKB=A0A0C4DH36,HUMAN|HGNC=5716|UniProtKB=P01834,HUMAN|HGNC=5588|UniProtKB=P01764,HUMAN|HGNC=15578|UniProtKB=Q96D71,HUMAN|HGNC=5857|UniProtKB=P0DOY3,HUMAN|HGNC=1736|UniProtKB=P60953,HUMAN|HGNC=5610|UniProtKB=P01767,HUMAN|Ensembl=ENSG00000282150|UniProtKB=A0A0J9YY99,HUMAN|HGNC=29125|UniProtKB=Q9UQ16,HUMAN|HGNC=5527|UniProtKB=P01860,HUMAN|HGNC=2092|UniProtKB=Q00610</td><td></td></tr>
<tr><td>GO:0009187</td><td>HUMAN|HGNC=237|UniProtKB=O43306</td><td>cyclic nucleotide metabolic process</td></tr>
<tr><td>GO:0009100</td><td>HUMAN|HGNC=9411|UniProtKB=P14314</td><td>glycoprotein metabolic process</td></tr>
<tr><td>GO:0031406</td><td>HUMAN|HGNC=607|UniProtKB=P02654,HUMAN|HGNC=1681|UniProtKB=P16070,HUMAN|HGNC=317|UniProtKB=P02771,HUMAN|HGNC=121|UniProtKB=O15254,HUMAN|HGNC=119|UniProtKB=Q15067</td><td>carboxylic acid binding</td></tr>
<tr><td>GO:0019941</td><td>HUMAN|HGNC=12458|UniProtKB=P62987,HUMAN|HGNC=12468|UniProtKB=P0CG48,HUMAN|HGNC=28477|UniProtKB=Q8NBM8,HUMAN|HGNC=7732|UniProtKB=Q15843,HUMAN|HGNC=12463|UniProtKB=P0CG47</td><td>modification-dependent protein catabolic process</td></tr>
<tr><td>GO:0007600</td><td>HUMAN|HGNC=711|UniProtKB=P49407</td><td>sensory perception</td></tr>
<tr><td>GO:0000124</td><td>HUMAN|HGNC=31797|UniProtKB=P0C7V6,HUMAN|HGNC=12347|UniProtKB=Q9Y4A5</td><td>SAGA complex</td></tr>
<tr><td>GO:0000904</td><td>HUMAN|HGNC=13937|UniProtKB=O95866</td><td>cell morphogenesis involved in differentiation</td></tr>
<tr><td>GO:0008375</td><td>HUMAN|HGNC=8127|UniProtKB=O15294</td><td>acetylglucosaminyltransferase activity</td></tr>
<tr><td>GO:0007131</td><td>HUMAN|HGNC=7326|UniProtKB=P20585,HUMAN|HGNC=20473|UniProtKB=Q9BX63</td><td>reciprocal meiotic recombination</td></tr>
<tr><td>GO:0060047</td><td>HUMAN|HGNC=10582|UniProtKB=Q9Y5Y9</td><td>heart contraction</td></tr>
</table>

<table>
<tr><td>GO:0006974</td><td>HUMAN|HGNC=12469|UniProtKB=P22314</td><td>cellular response to DNA damage stimulus</td></tr>
<tr><td>GO:0000038</td><td>HUMAN|HGNC=24175|UniProtKB=Q9P035</td><td>very long-chain fatty acid metabolic process</td></tr>
<tr><td>GO:0045202</td><td>HUMAN|HGNC=17780|UniProtKB=Q562R1,HUMAN|HGNC=11168|UniProtKB=Q13884,HUMAN|HGNC=6835|UniProtKB=P78559,HUMAN|HGNC=11512|UniProtKB=Q9H2B2</td><td>synapse</td></tr>
<tr><td>GO:0016877</td><td>HUMAN|HGNC=10996|UniProtKB=O14975</td><td>ligase activity, forming carbon-sulfur bonds</td></tr>
<tr><td>GO:0045055</td><td>HUMAN|HGNC=11491|UniProtKB=P43405,HUMAN|HGNC=9761|UniProtKB=Q15907</td><td>regulated exocytosis</td></tr>
<tr><td>GO:0004620</td><td>HUMAN|HGNC=607|UniProtKB=P02654,HUMAN|HGNC=13921|UniProtKB=O95870</td><td>phospholipase activity</td></tr>
<tr><td>GO:0050870</td><td>HUMAN|HGNC=5261|UniProtKB=P10809</td><td>positive regulation of T cell activation</td></tr>
<tr><td>GO:0006611</td><td>HUMAN|HGNC=2431|UniProtKB=P55060</td><td>protein export from nucleus</td></tr>
<tr><td>GO:0005201</td><td>HUMAN|HGNC=3662|UniProtKB=P02675,HUMAN|HGNC=2209|UniProtKB=P20908</td><td>extracellular matrix structural constituent</td></tr>
<tr><td>GO:0042092</td><td>HUMAN|HGNC=533|UniProtKB=P04083</td><td>type 2 immune response</td></tr>
<tr><td>GO:0045766</td><td>HUMAN|HGNC=533|UniProtKB=P04083</td><td>positive regulation of angiogenesis</td></tr>
<tr><td>GO:0006406</td><td>HUMAN|HGNC=17821|UniProtKB=O00148,HUMAN|HGNC=19072|UniProtKB=Q96J01</td><td>mRNA export from nucleus</td></tr>
<tr><td>GO:0003700</td><td>HUMAN|HGNC=9349|UniProtKB=Q9NQX1</td><td>DNA-binding transcription factor activity</td></tr>
<tr><td>PC00211</td><td>HUMAN|HGNC=30391|UniProtKB=Q9UG01,HUMAN|HGNC=21054|UniProtKB=Q86UC2</td><td>structural protein</td></tr>
<tr><td>GO:0097120</td><td>HUMAN|HGNC=6325|UniProtKB=O60282,HUMAN|HGNC=1406|UniProtKB=Q9Y698,HUMAN|HGNC=30377|UniProtKB=Q14160</td><td>receptor localization to synapse</td></tr>
<tr><td>GO:0048786</td><td>HUMAN|HGNC=1117|UniProtKB=Q9UPA5,HUMAN|HGNC=13406|UniProtKB=Q9Y6V0,HUMAN|HGNC=8515|UniProtKB=Q9HC10</td><td>presynaptic active zone</td></tr>
<tr><td>GO:0051641</td><td>HUMAN|HGNC=29327|UniProtKB=Q5VZ46</td><td>cellular localization</td></tr>
<tr><td>GO:0016310</td><td>HUMAN|HGNC=19296|UniProtKB=Q9UF33,HUMAN|HGNC=2850|UniProtKB=Q9Y6T7</td><td>phosphorylation</td></tr>
<tr><td>GO:0019725</td><td>HUMAN|HGNC=17169|UniProtKB=Q13162,HUMAN|HGNC=16753|UniProtKB=P30041,HUMAN|HGNC=9355|UniProtKB=P30044,HUMAN|HGNC=9353|UniProtKB=P32119,HUMAN|HGNC=9354|UniProtKB=P30048,HUMAN|HGNC=9352|UniProtKB=Q06830,HUMAN|HGNC=4623|UniProtKB=P00390</td><td>cellular homeostasis</td></tr>
<tr><td>GO:0042475</td><td>HUMAN|HGNC=3157|UniProtKB=Q92838</td><td>odontogenesis of dentin-containing tooth</td></tr>
<tr><td>GO:0072521</td><td>HUMAN|HGNC=12405|UniProtKB=P02766</td><td>purine-containing compound metabolic process</td></tr>
<tr><td>GO:0044267</td><td>HUMAN|HGNC=333|UniProtKB=P01019,HUMAN|HGNC=2479|UniProtKB=O76096,HUMAN|HGNC=8583|UniProtKB=P05121,HUMAN|HGNC=3311|UniProtKB=P30740,HUMAN|HGNC=8948|UniProtKB=P29622,HUMAN|HGNC=1546|UniProtKB=P50454,HUMAN|HGNC=8941|UniProtKB=P01009,HUMAN|HGNC=1540|UniProtKB=P08185,HUMAN|HGNC=8824|UniProtKB=P36955,HUMAN|HGNC=19986|UniProtKB=P99999,HUMAN|HGNC=8942|UniProtKB=P48595,HU</td><td>cellular protein metabolic process</td></tr>
</table>

<table>
<tr><td></td><td>MAN|HGNC=1050|UniProtKB=P55957,HUMAN|HGNC=3658|UniProtKB=Q9UGM5,HUMAN|HGNC=11820|UniProtKB=P01033,HUMAN|HGNC=10569|UniProtKB=P29508,HUMAN|HGNC=4838|UniProtKB=P05546,HUMAN|HGNC=6383|UniProtKB=P01042,HUMAN|HGNC=16|UniProtKB=P01011,HUMAN|HGNC=775|UniProtKB=P01008,HUMAN|HGNC=1228|UniProtKB=P05155,HUMAN|HGNC=9075|UniProtKB=P08697,HUMAN|HGNC=11583|UniProtKB=P05543,HUMAN|HGNC=8955|UniProtKB=P50453</td><td></td></tr>
<tr><td>GO:0000209</td><td>HUMAN|HGNC=12491|UniProtKB=P61081,HUMAN|HGNC=12476|UniProtKB=P61077,HUMAN|HGNC=16276|UniProtKB=Q9C035,HUMAN|HGNC=9261|UniProtKB=Q13356,HUMAN|HGNC=17598|UniProtKB=Q9Y385</td><td>protein polyubiquitination</td></tr>
<tr><td>GO:0046785</td><td>HUMAN|HGNC=28959|UniProtKB=Q14008,HUMAN|HGNC=14906|UniProtKB=Q8N4C6</td><td>microtubule polymerization</td></tr>
<tr><td>PC00244</td><td>HUMAN|HGNC=7803|UniProtKB=Q12986,HUMAN|HGNC=17808|UniProtKB=Q9UPT8</td><td>zinc finger transcription factor</td></tr>
<tr><td>GO:0004725</td><td>HUMAN|HGNC=122|UniProtKB=P24666,HUMAN|HGNC=9308|UniProtKB=Q15257,HUMAN|HGNC=9645|UniProtKB=Q05209,HUMAN|HGNC=9673|UniProtKB=Q12913,HUMAN|HGNC=29884|UniProtKB=Q8TF42</td><td>protein tyrosine phosphatase activity</td></tr>
<tr><td>GO:0050829</td><td>HUMAN|HGNC=2761|UniProtKB=P59665,HUMAN|HGNC=6517|UniProtKB=P18428,HUMAN|HGNC=1472|UniProtKB=P49913,HUMAN|HGNC=6740|UniProtKB=P61626</td><td>defense response to Gram-negative bacterium</td></tr>
<tr><td>GO:0008514</td><td>HUMAN|HGNC=11030|UniProtKB=Q9Y6R1,HUMAN|HGNC=11027|UniProtKB=P02730,HUMAN|HGNC=11034|UniProtKB=Q2Y0W8</td><td>organic anion transmembrane transporter activity</td></tr>
<tr><td>GO:0046914</td><td>HUMAN|HGNC=11180|UniProtKB=P04179</td><td>transition metal ion binding</td></tr>
<tr><td>GO:0034446</td><td>HUMAN|HGNC=6616|UniProtKB=P48059,HUMAN|HGNC=14653|UniProtKB=Q9HBI1,HUMAN|HGNC=6040|UniProtKB=Q13418</td><td>substrate adhesion-dependent cell spreading</td></tr>
<tr><td>GO:0061564</td><td>HUMAN|HGNC=9086|UniProtKB=P60201</td><td>axon development</td></tr>
<tr><td>GO:0050728</td><td>HUMAN|HGNC=2479|UniProtKB=O76096,HUMAN|HGNC=7967|UniProtKB=Q96RI1</td><td>negative regulation of inflammatory response</td></tr>
<tr><td>GO:0035082</td><td>HUMAN|HGNC=15476|UniProtKB=P63167,HUMAN|HGNC=15848|UniProtKB=Q14141,HUMAN|HGNC=16053|UniProtKB=Q8N1V2,HUMAN|HGNC=20908|UniProtKB=Q86YF9,HUMAN|HGNC=25631|UniProtKB=Q96MT7,HUMAN|HGNC=1717|UniProtKB=Q16181,HUMAN|HGNC=3244|UniProtKB=Q9NZN3,HUMAN|HGNC=29262|UniProtKB=Q9P2H3,HUMAN|HGNC=7729|UniProtKB=Q15019</td><td>axoneme assembly</td></tr>
<tr><td>GO:0005089</td><td>HUMAN|HGNC=21740|UniProtKB=Q6ZV73,HUMAN|HGNC=21295|UniProtKB=Q8TE68</td><td>Rho guanyl-nucleotide exchange factor activity</td></tr>
<tr><td>GO:0030672</td><td>HUMAN|HGNC=11510|UniProtKB=Q8N9I0,HUMAN|HGNC=2090|UniProtKB=P09496,HUMAN|HGNC=8515|UniProtKB=Q9HC10</td><td>synaptic vesicle membrane</td></tr>
<tr><td>GO:0008237</td><td>HUMAN|HGNC=7900|UniProtKB=P55786,HUMAN|HGNC=15722|UniProtKB=Q8WXQ8,HUMAN|HGNC=2300|UniProtKB=Q96IY4,HUMAN|HGNC=18173|UniProtKB=Q9NZ08,HUMAN|HGNC=2312|UniProtKB=P15169</td><td>metallopeptidase activity</td></tr>
<tr><td>GO:0034501</td><td>HUMAN|HGNC=24054|UniProtKB=Q8NG31,HUMAN|HGNC=12401|UniProtKB=P33981</td><td>protein localization to kinetochore</td></tr>
<tr><td>GO:0046040</td><td>HUMAN|HGNC=794|UniProtKB=P31939,HUMAN|HGNC=5157|UniProtKB=P00492,HUMAN|HGNC=469|UniProtKB=Q01433</td><td>IMP metabolic process</td></tr>
<tr><td>GO:0000175</td><td>HUMAN|HGNC=9207|UniProtKB=Q9UFF9</td><td>3'-5'-exoribonuclease activity</td></tr>
</table>

<table>
<tr><td>GO:0072583</td><td>HUMAN|HGNC=2090|UniProtKB=P09496,HUMAN|HGNC=561|UniProtKB=O95782</td><td>clathrin-dependent endocytosis</td></tr>
<tr><td>GO:0009062</td><td>HUMAN|HGNC=14451|UniProtKB=Q9BQK8</td><td>fatty acid catabolic process</td></tr>
<tr><td>GO:0019637</td><td>HUMAN|HGNC=4242|UniProtKB=O94808</td><td>organophosphate metabolic process</td></tr>
<tr><td>GO:0006760</td><td>HUMAN|HGNC=4248|UniProtKB=Q92820</td><td>folic acid-containing compound metabolic process</td></tr>
<tr><td>GO:0001510</td><td>HUMAN|HGNC=17136|UniProtKB=Q8IY81</td><td>RNA methylation</td></tr>
<tr><td>GO:0051056</td><td>HUMAN|HGNC=21035|UniProtKB=Q8N392</td><td>regulation of small GTPase mediated signal transduction</td></tr>
<tr><td>GO:0034237</td><td>HUMAN|HGNC=12733|UniProtKB=Q9Y6W5,HUMAN|HGNC=9380|UniProtKB=P17612,HUMAN|HGNC=28164|UniProtKB=Q96M27</td><td>protein kinase A regulatory subunit binding</td></tr>
<tr><td>GO:0062023</td><td>HUMAN|HGNC=3778|UniProtKB=P02751,HUMAN|HGNC=3662|UniProtKB=P02675,HUMAN|HGNC=6561|UniProtKB=P09382,HUMAN|HGNC=2211|UniProtKB=P12109,HUMAN|HGNC=10496|UniProtKB=P06703,HUMAN|HGNC=8569|UniProtKB=P29122,HUMAN|HGNC=3625|UniProtKB=O75636,HUMAN|HGNC=3153|UniProtKB=Q16610,HUMAN|HGNC=3694|UniProtKB=P02679,HUMAN|HGNC=2213|UniProtKB=P12111,HUMAN|HGNC=8601|UniProtKB=Q06141,HUMAN|HGNC=3531|UniProtKB=P00488,HUMAN|HGNC=2188|UniProtKB=Q99715,HUMAN|HGNC=3624|UniProtKB=Q15485</td><td>collagen-containing extracellular matrix</td></tr>
<tr><td>PC00137</td><td>HUMAN|HGNC=9141|UniProtKB=Q15126,HUMAN|HGNC=4922|UniProtKB=P19367,HUMAN|HGNC=9021|UniProtKB=P14618,HUMAN|HGNC=24822|UniProtKB=Q9H479,HUMAN|HGNC=7850|UniProtKB=P22392,HUMAN|HGNC=8997|UniProtKB=P48426,HUMAN|HGNC=7849|UniProtKB=P15531,HUMAN|HGNC=2850|UniProtKB=Q9Y6T7</td><td>kinase</td></tr>
<tr><td>GO:0005791</td><td>HUMAN|HGNC=3530|UniProtKB=P00748</td><td>rough endoplasmic reticulum</td></tr>
<tr><td>PC00160</td><td>HUMAN|HGNC=8906|UniProtKB=Q96G03,HUMAN|HGNC=8905|UniProtKB=P36871,HUMAN|HGNC=8888|UniProtKB=P18669,HUMAN|HGNC=1093|UniProtKB=P07738</td><td>mutase</td></tr>
<tr><td>GO:0035097</td><td>HUMAN|HGNC=7133|UniProtKB=O14686,HUMAN|HGNC=13726|UniProtKB=Q8NEZ4,HUMAN|HGNC=18039|UniProtKB=Q9UGL1</td><td>histone methyltransferase complex</td></tr>
<tr><td>GO:1990126</td><td>HUMAN|HGNC=24641|UniProtKB=Q7Z3J2</td><td>retrograde transport, endosome to plasma membrane</td></tr>
<tr><td>GO:0071805</td><td>HUMAN|HGNC=800|UniProtKB=P50993,HUMAN|HGNC=806|UniProtKB=P54709</td><td>potassium ion transmembrane transport</td></tr>
<tr><td>GO:0007190</td><td>HUMAN|HGNC=4381|UniProtKB=Q14344,HUMAN|HGNC=237|UniProtKB=O43306</td><td>activation of adenylate cyclase activity</td></tr>
<tr><td>GO:0007409</td><td>HUMAN|HGNC=17780|UniProtKB=Q562R1,HUMAN|HGNC=6835|UniProtKB=P78559,HUMAN|HGNC=18994|UniProtKB=Q8TDC3</td><td>axonogenesis</td></tr>
<tr><td>GO:0030705</td><td>HUMAN|HGNC=6317|UniProtKB=O43896</td><td>cytoskeleton-dependent intracellular transport</td></tr>
<tr><td>GO:0043547</td><td>HUMAN|HGNC=25694|UniProtKB=Q8TC07,HUMAN|HGNC=10632|UniProtKB=P13501,HUMAN|HGNC=25361|UniProtKB=Q8N264,HUMAN|HGNC=17102|UniProtKB=Q92619,HUMAN|HGNC=9859|UniProtKB=P52306,HUMAN|HGNC=25571|UniProtKB=Q9NVG8</td><td>positive regulation of GTPase activity</td></tr>
</table>

GO:0005581	HUMAN|HGNC=2209|UniProtKB=P20908	collagen trimer
GO:0005844	HUMAN|HGNC=30547|UniProtKB=Q96K17,HUMAN|HGNC=18585|UniProtKB=Q96DH6	polysome
GO:0008528	HUMAN|HGNC=3537|UniProtKB=P25116,HUMAN|HGNC=10608|UniProtKB=P47872	G protein-coupled peptide receptor activity
GO:0072657	HUMAN|HGNC=1406|UniProtKB=Q9Y698	protein localization to membrane
GO:0016409	HUMAN|HGNC=2328|UniProtKB=P50416	palmitoyltransferase activity
GO:0006520	HUMAN|HGNC=24728|UniProtKB=A2RTX5,HUMAN|HGNC=2678|UniProtKB=P14868,HUMAN|HGNC=20|UniProtKB=P49588,HUMAN|HGNC=4162|UniProtKB=P41250,HUMAN|HGNC=11572|UniProtKB=P26639,HUMAN|HGNC=12729|UniProtKB=P23381	cellular amino acid metabolic process
GO:0032456	HUMAN|HGNC=24641|UniProtKB=Q7Z3J2,HUMAN|HGNC=3244|UniProtKB=Q9NZN3	endocytic recycling
GO:0007389	HUMAN|HGNC=7002|UniProtKB=A6NDR6	pattern specification process
GO:0036464	HUMAN|HGNC=20165|UniProtKB=Q9P2P1,HUMAN|HGNC=5048|UniProtKB=Q00839	cytoplasmic ribonucleoprotein granule
GO:0019956	HUMAN|HGNC=6153|UniProtKB=P05556	chemokine binding
GO:0000139	HUMAN|HGNC=25951|UniProtKB=Q6ULP2,HUMAN|HGNC=20726|UniProtKB=Q8WVM8,HUMAN|HGNC=16499|UniProtKB=Q96DA2,HUMAN|HGNC=6631|UniProtKB=P49257,HUMAN|HGNC=15453|UniProtKB=Q9H3P7	Golgi membrane
GO:0006261	HUMAN|HGNC=7326|UniProtKB=P20585	DNA-dependent DNA replication
GO:0042168	HUMAN|HGNC=5171|UniProtKB=P02790	heme metabolic process
GO:0004806	HUMAN|HGNC=6522|UniProtKB=P04180,HUMAN|HGNC=108|UniProtKB=P22303	triglyceride lipase activity
GO:0007420	HUMAN|HGNC=15474|UniProtKB=Q9Y566,HUMAN|HGNC=7002|UniProtKB=A6NDR6,HUMAN|HGNC=14294|UniProtKB=Q9BYB0	brain development
GO:0005903	HUMAN|HGNC=4381|UniProtKB=Q14344	brush border
GO:0030301	HUMAN|HGNC=607|UniProtKB=P02654,HUMAN|HGNC=603|UniProtKB=P04114	cholesterol transport
GO:0034728	HUMAN|HGNC=4716|UniProtKB=P16403,HUMAN|HGNC=4718|UniProtKB=P10412,HUMAN|HGNC=4719|UniProtKB=P16401	nucleosome organization
PC00052	HUMAN|HGNC=25268|UniProtKB=Q96LR9,HUMAN|HGNC=607|UniProtKB=P02654,HUMAN|HGNC=615|UniProtKB=Q13790,HUMAN|HGNC=618|UniProtKB=O14791,HUMAN|HGNC=613|UniProtKB=P02649,HUMAN|HGNC=600|UniProtKB=P02647,HUMAN|HGNC=612|UniProtKB=P05090,HUMAN|HGNC=603|UniProtKB=P04114	apolipoprotein
GO:0016790	HUMAN|HGNC=6737|UniProtKB=O75608,HUMAN|HGNC=3465|UniProtKB=P10768	thiolester hydrolase activity
GO:0006281	HUMAN|HGNC=12347|UniProtKB=Q9Y4A5	DNA repair
PC00217	HUMAN|HGNC=30025|UniProtKB=Q5VV67,HUMAN|HGNC=9885|UniProtKB=P29374,HUMAN|HGNC=19692|UniProtKB=Q96F24,HUMAN|HGNC=20250|UniProtKB=Q96IF1,HUMAN|HGNC=16272|UniProtKB=Q96JK9	transcription cofactor

GO:0050840	HUMAN|HGNC=6561|UniProtKB=P09382,HUMAN|HGNC=6153|UniProtKB=P05556,HUMAN|HGNC=11219|UniProtKB=P09486	extracellular matrix binding
GO:0008525	HUMAN|HGNC=9093|UniProtKB=P55058	phosphatidylcholine transporter activity
GO:0031491	HUMAN|HGNC=17780|UniProtKB=Q562R1,HUMAN|HGNC=4716|UniProtKB=P16403,HUMAN|HGNC=4718|UniProtKB=P10412,HUMAN|HGNC=4719|UniProtKB=P16401	nucleosome binding
GO:0043195	HUMAN|HGNC=7641|UniProtKB=P54920	terminal bouton
GO:0000729	HUMAN|HGNC=23845|UniProtKB=Q8IY92	DNA double-strand break processing
GO:0042771	HUMAN|HGNC=28771|UniProtKB=Q9BVC6	intrinsic apoptotic signaling pathway in response to DNA damage by p53 class mediator
GO:0042995	HUMAN|HGNC=18756|UniProtKB=Q9BYZ6,HUMAN|HGNC=9802|UniProtKB=P15153	cell projection
GO:0048589	HUMAN|HGNC=15474|UniProtKB=Q9Y566,HUMAN|HGNC=544|UniProtKB=P08133,HUMAN|HGNC=533|UniProtKB=P04083,HUMAN|HGNC=14294|UniProtKB=Q9BYB0	developmental growth
GO:0030175	HUMAN|HGNC=11148|UniProtKB=Q16658	filopodium
GO:0005795	HUMAN|HGNC=8016|UniProtKB=P46459	Golgi stack
GO:0035023	HUMAN|HGNC=3537|UniProtKB=P25116,HUMAN|HGNC=4472|UniProtKB=Q14330,HUMAN|HGNC=21295|UniProtKB=Q8TE68,HUMAN|HGNC=3540|UniProtKB=Q96RI0	regulation of Rho protein signal transduction
GO:0009074	HUMAN|HGNC=3579|UniProtKB=P16930	aromatic amino acid family catabolic process
GO:0034605	HUMAN|HGNC=5253|UniProtKB=P07900,HUMAN|HGNC=5258|UniProtKB=P08238	cellular response to heat
GO:0003777	HUMAN|HGNC=2949|UniProtKB=Q8TD57,HUMAN|HGNC=2951|UniProtKB=Q9C0G6,HUMAN|HGNC=2952|UniProtKB=Q96JB1	microtubule motor activity
PC00051	HUMAN|HGNC=2761|UniProtKB=P59665,HUMAN|HGNC=1472|UniProtKB=P49913	antimicrobial response protein
GO:0010833	HUMAN|HGNC=19246|UniProtKB=Q9NYB0	telomere maintenance via telomere lengthening
GO:0047496	HUMAN|HGNC=13206|UniProtKB=O60296	vesicle transport along microtubule
GO:0004521	HUMAN|HGNC=28422|UniProtKB=Q8NCE0,HUMAN|HGNC=20165|UniProtKB=Q9P2P1,HUMAN|HGNC=12379|UniProtKB=Q15631,HUMAN|HGNC=17904|UniProtKB=Q9NRR4	endoribonuclease activity
GO:0051254	HUMAN|HGNC=27424|UniProtKB=Q5T481,HUMAN|HGNC=29190|UniProtKB=Q9UPQ9	positive regulation of RNA metabolic process
GO:0008637	HUMAN|HGNC=21689|UniProtKB=Q9Y3D6,HUMAN|HGNC=5261|UniProtKB=P10809,HUMAN|HGNC=2973|UniProtKB=O00429,HUMAN|HGNC=17865|UniProtKB=Q96A26,HUMAN|HGNC=1050|UniProtKB=P55957	apoptotic mitochondrial changes
GO:0017121	HUMAN|HGNC=25240|UniProtKB=Q4KMQ2	plasma membrane phospholipid scrambling
GO:0043085	HUMAN|HGNC=4312|UniProtKB=P48507,HUMAN|HGNC=613|UniProtKB=P02649	positive regulation of catalytic activity
GO:0004222	HUMAN|HGNC=5171|UniProtKB=P02790,HUMAN|HGNC=7176|UniProtKB=P14780,HUMAN|HGNC=11820|UniProtKB=P01033,HUM	metalloendopeptidase activity

	AN|HGNC=12585|UniProtKB=P31930,HUMAN|HGNC=188|UniProtKB=O14672	
PC00153	HUMAN|HGNC=12586|UniProtKB=P22695,HUMAN|HGNC=5171|UniProtKB=P02790,HUMAN|HGNC=199|UniProtKB=O43506,HUMAN|HGNC=7176|UniProtKB=P14780,HUMAN|HGNC=12585|UniProtKB=P31930,HUMAN|HGNC=2300|UniProtKB=Q96IY4,HUMAN|HGNC=11763|UniProtKB=P02786,HUMAN|HGNC=18449|UniProtKB=P28838,HUMAN|HGNC=7900|UniProtKB=P55786,HUMAN|HGNC=24437|UniProtKB=Q96KP4,HUMAN|HGNC=5381|UniProtKB=P14735,HUMAN|HGNC=15722|UniProtKB=Q8WXQ8,HUMAN|HGNC=20675|UniProtKB=Q96KN2,HUMAN|HGNC=18173|UniProtKB=Q9NZ08	metalloprotease
GO:0005802	HUMAN|HGNC=25951|UniProtKB=Q6ULP2,HUMAN|HGNC=26486|UniProtKB=Q6PJW8,HUMAN|HGNC=9772|UniProtKB=Q13637,HUMAN|HGNC=8569|UniProtKB=P29122,HUMAN|HGNC=6752|UniProtKB=P20645	trans-Golgi network
GO:0005524	HUMAN|HGNC=8876|UniProtKB=P17858,HUMAN|HGNC=9465|UniProtKB=P11908,HUMAN|HGNC=20|UniProtKB=P49588,HUMAN|HGNC=5239|UniProtKB=P17066,HUMAN|HGNC=8878|UniProtKB=Q01813,HUMAN|HGNC=823|UniProtKB=P25705,HUMAN|HGNC=8898|UniProtKB=P07205,HUMAN|HGNC=5241|UniProtKB=P11142,HUMAN|HGNC=5253|UniProtKB=P07900,HUMAN|HGNC=5258|UniProtKB=P08238,HUMAN|HGNC=5238|UniProtKB=P11021,HUMAN|HGNC=5240|UniProtKB=P48741,HUMAN|HGNC=9462|UniProtKB=P60891	ATP binding
GO:0035257	HUMAN|HGNC=6207|UniProtKB=P14923	nuclear hormone receptor binding
GO:0045595	HUMAN|HGNC=9583|UniProtKB=P26599,HUMAN|HGNC=29884|UniProtKB=Q8TF42	regulation of cell differentiation
GO:0008154	HUMAN|HGNC=18271|UniProtKB=Q8N8S7	actin polymerization or depolymerization
GO:0098839	HUMAN|HGNC=1406|UniProtKB=Q9Y698	postsynaptic density membrane
GO:0006627	HUMAN|HGNC=12585|UniProtKB=P31930	protein processing involved in protein targeting to mitochondrion
GO:0002695	HUMAN|HGNC=2479|UniProtKB=O76096	negative regulation of leukocyte activation
PC00097	HUMAN|HGNC=983|UniProtKB=P06276,HUMAN|HGNC=16268|UniProtKB=Q8IY17,HUMAN|HGNC=16219|UniProtKB=Q8TEA8,HUMAN|HGNC=17820|UniProtKB=Q9H0P0,HUMAN|HGNC=108|UniProtKB=P22303	esterase
GO:0001227	HUMAN|HGNC=12879|UniProtKB=P21506	DNA-binding transcription repressor activity, RNA polymerase II-specific
GO:0004712	HUMAN|HGNC=12401|UniProtKB=P33981	protein serine/threonine/tyrosine kinase activity
GO:0005125	HUMAN|HGNC=9240|UniProtKB=P02775,HUMAN|HGNC=10632|UniProtKB=P13501,HUMAN|HGNC=8862|UniProtKB=P10720,HUMAN|HGNC=8861|UniProtKB=P02776,HUMAN|HGNC=11766|UniProtKB=P01137	cytokine activity
GO:0016616	HUMAN|HGNC=253|UniProtKB=P11766,HUMAN|HGNC=5382|UniProtKB=O75874,HUMAN|HGNC=6970|UniProtKB=P40925,HUMAN	oxidoreductase activity, acting on the CH-OH group

|HGNC=4801|UniProtKB=P40939,HUMAN|HGNC=6535|UniProtKB=P00338,HUMAN|HGNC=6984|UniProtKB=P23368,HUMAN|HGNC=8891|UniProtKB=P52209,HUMAN|HGNC=6971|UniProtKB=P40926,HUMAN|HGNC=6541|UniProtKB=P07195,HUMAN|HGNC=251|UniProtKB=P00326,HUMAN|HGNC=18985|UniProtKB=Q7Z4W1,HUMAN|HGNC=5383|UniProtKB=P48735,HUMAN|HGNC=255|UniProtKB=P28332,HUMAN|HGNC=5386|UniProtKB=P51553,HUMAN|HGNC=380|UniProtKB=P14550 — of donors, NAD or NADP as acceptor

GO:0008023 — HUMAN|HGNC=11617|UniProtKB=Q15369,HUMAN|HGNC=11619|UniProtKB=Q15370,HUMAN|HGNC=24224|UniProtKB=Q9NYV4 — transcription elongation factor complex

GO:0010506 — HUMAN|HGNC=18423|UniProtKB=O75140,HUMAN|HGNC=2973|UniProtKB=O00429 — regulation of autophagy

GO:1901701 — HUMAN|HGNC=26068|UniProtKB=Q6IAA8,HUMAN|HGNC=253|UniProtKB=P11766,HUMAN|HGNC=11179|UniProtKB=P00441,HUMAN|HGNC=9855|UniProtKB=P62834,HUMAN|HGNC=17955|UniProtKB=O43504,HUMAN|HGNC=9353|UniProtKB=P32119,HUMAN|HGNC=251|UniProtKB=P00326,HUMAN|HGNC=533|UniProtKB=P04083,HUMAN|HGNC=9857|UniProtKB=P61224,HUMAN|HGNC=9352|UniProtKB=Q06830,HUMAN|HGNC=255|UniProtKB=P28332,HUMAN|HGNC=15606|UniProtKB=Q9UHA4 — cellular response to oxygen-containing compound

GO:0005615 — HUMAN|HGNC=3778|UniProtKB=P02751,HUMAN|HGNC=333|UniProtKB=P01019,HUMAN|HGNC=316|UniProtKB=P43652,HUMAN|HGNC=7516|UniProtKB=Q9HC84,HUMAN|HGNC=11085|UniProtKB=O75093,HUMAN|HGNC=10496|UniProtKB=P06703,HUMAN|HGNC=8824|UniProtKB=P36955,HUMAN|HGNC=4831|UniProtKB=P69891,HUMAN|HGNC=38476|UniProtKB=B9A064,HUMAN|HGNC=5877|UniProtKB=P01703,HUMAN|HGNC=5834|UniProtKB=P06312,HUMAN|HGNC=9756|UniProtKB=O00391,HUMAN|HGNC=1472|UniProtKB=P49913,HUMAN|HGNC=8044|UniProtKB=P80303,HUMAN|HGNC=3011|UniProtKB=Q07507,HUMAN|HGNC=7218|UniProtKB=P05164,HUMAN|HGNC=9495|UniProtKB=P24158,HUMAN|HGNC=28883|UniProtKB=Q9H2U2,HUMAN|HGNC=5576|UniProtKB=P01817,HUMAN|HGNC=1540|UniProtKB=P08185,HUMAN|HGNC=11179|UniProtKB=P00441,HUMAN|HGNC=6522|UniProtKB=P04180,HUMAN|HGNC=21265|UniProtKB=Q9NZP8,HUMAN|HGNC=5897|UniProtKB=A0A075B6K4,HUMAN|HGNC=5526|UniProtKB=P01859,HUMAN|HGNC=5739|UniProtKB=A0A075B6S9,HUMAN|HGNC=6561|UniProtKB=P09382,HUMAN|HGNC=5580|UniProtKB=P01762,HUMAN|HGNC=5633|UniProtKB=A0A075B7D8,HUMAN|HGNC=5582|UniProtKB=A0A0B4J1V0,HUMAN|HGNC=11820|UniProtKB=P01033,HUMAN|HGNC=775|UniProtKB=P01008,HUMAN|HGNC=5652|UniProtKB=A0A075B6R2,HUMAN|HGNC=5836|UniProtKB=A0A0C4DH24,HUMAN|HGNC=3662|UniProtKB=P02675,HUMAN|HGNC=5887|UniProtKB=P01706,HUMAN|HGNC=5662|UniProtKB=A0A0B4J1U7,HUMAN|HGNC=5746|UniProtKB=P01611,HUMAN|HGNC=8898|UniProtKB=P07205,HUMAN|HGNC=17213|UniProtKB=Q9BWP8,HUMAN|HGNC=8601|UniProtKB=Q06141,HUMAN|HGNC=5857|UniProtKB=P0DOY3,HUMAN|HGNC=5610|UniProtKB=P01767,HUMAN|HGNC=16|UniProtKB=P01011,HUMAN|Ensembl=ENSG00000282150|UniProtKB=A0A0J9YY99,HUMAN|HGNC=5527|UniProtKB=P01860,HUMAN|HGNC=24588|UniProtKB=Q86XP6,HUMAN|HGNC=108|UniProtKB=P22303,HUMAN|HGNC=584|UniProtKB=P02743,HUMAN|HGNC=5783|UniProtKB=A0A075B6P5,HUMAN|HGNC=5753|UniProtKB=P01593,HUMAN|HGNC=5239|UniProtKB=P1706 — extracellular space

6,HUMAN|HGNC=4932|UniProtKB=P01889,HUMAN|HGNC=3625|
UniProtKB=O75636,HUMAN|HGNC=5554|UniProtKB=P01743,HU
MAN|HGNC=5634|UniProtKB=A0A075B7F0,HUMAN|HGNC=2300|
UniProtKB=Q96IY4,HUMAN|HGNC=5931|UniProtKB=A0A075B6I0,
HUMAN|HGNC=615|UniProtKB=Q13790,HUMAN|HGNC=8861|Un
iProtKB=P02776,HUMAN|HGNC=5933|UniProtKB=A0A0B4J1Y8,HU
MAN|HGNC=1324|UniProtKB=P0C0L5,HUMAN|HGNC=11583|Uni
ProtKB=P05543,HUMAN|HGNC=5563|UniProtKB=A0A075B7D0,HU
MAN|HGNC=8583|UniProtKB=P05121,HUMAN|HGNC=5890|UniPr
otKB=P01705,HUMAN|HGNC=533|UniProtKB=P04083,HUMAN|H
GNC=1095|UniProtKB=P17213,HUMAN|HGNC=7515|UniProtKB=P
98088,HUMAN|HGNC=5528|UniProtKB=P01861,HUMAN|HGNC=8
941|UniProtKB=P01009,HUMAN|HGNC=5888|UniProtKB=P01704,
HUMAN|HGNC=5141|UniProtKB=P00738,HUMAN|HGNC=30456|
UniProtKB=Q96DA0,HUMAN|HGNC=3309|UniProtKB=P08246,HU
MAN|HGNC=5657|UniProtKB=P0DP08,HUMAN|HGNC=5781|UniP
rotKB=A0A0C4DH68,HUMAN|HGNC=5901|UniProtKB=A0A075B6K
0,HUMAN|HGNC=5743|UniProtKB=A0A0C4DH67,HUMAN|Gene=
HV309_HUMAN|UniProtKB=P01782,HUMAN|HGNC=5468|UniProt
KB=P35858,HUMAN|HGNC=2478|UniProtKB=Q15828,HUMAN|HG
NC=4620|UniProtKB=P06396,HUMAN|HGNC=3964|UniProtKB=P0
1225,HUMAN|HGNC=11219|UniProtKB=P09486,HUMAN|HGNC=2
689|UniProtKB=P09172,HUMAN|HGNC=3694|UniProtKB=P02679,
HUMAN|HGNC=5617|UniProtKB=A0A075B6Q5,HUMAN|HGNC=55
94|UniProtKB=P0DP03,HUMAN|HGNC=5735|UniProtKB=A0A075B
6S5,HUMAN|HGNC=5636|UniProtKB=A0A075B7B8,HUMAN|HGNC
=5581|UniProtKB=P01766,HUMAN|HGNC=613|UniProtKB=P02649
,HUMAN|HGNC=3624|UniProtKB=Q15485,HUMAN|HGNC=16948|
UniProtKB=Q969H8,HUMAN|HGNC=5479|UniProtKB=P01877,HU
MAN|HGNC=16721|UniProtKB=A6NKQ9,HUMAN|HGNC=5898|Un
iProtKB=A0A075B6K2,HUMAN|HGNC=3311|UniProtKB=P30740,H
UMAN|HGNC=5882|UniProtKB=P01701,HUMAN|HGNC=9750|Uni
ProtKB=P20742,HUMAN|HGNC=4823|UniProtKB=P69905,HUMAN
|HGNC=11891|UniProtKB=P05452,HUMAN|HGNC=5525|UniProtK
B=P01857,HUMAN|HGNC=2761|UniProtKB=P59665,HUMAN|HGN
C=10632|UniProtKB=P13501,HUMAN|HGNC=3157|UniProtKB=Q9
2838,HUMAN|HGNC=5741|UniProtKB=P01602,HUMAN|HGNC=12
726|UniProtKB=P04275,HUMAN|HGNC=5817|UniProtKB=P01619,
HUMAN|HGNC=10497|UniProtKB=P31151,HUMAN|HGNC=10045
|UniProtKB=P10153,HUMAN|HGNC=8043|UniProtKB=Q02818,HU
MAN|HGNC=5575|UniProtKB=A0A0B4J1V2,HUMAN|HGNC=5825|
UniProtKB=A0A0C4DH25,HUMAN|HGNC=11834|UniProtKB=P2940
1,HUMAN|HGNC=5549|UniProtKB=A0A0C4DH31,HUMAN|HGNC=
11092|UniProtKB=P03973,HUMAN|HGNC=43788|UniProtKB=Q8N
HM4,HUMAN|HGNC=5480|UniProtKB=P01880,HUMAN|HGNC=56
59|UniProtKB=A0A0C4DH38,HUMAN|Gene=KV311_HUMAN|UniP
rotKB=P04433,HUMAN|HGNC=6724|UniProtKB=P51884,HUMAN|
HGNC=399|UniProtKB=P02768,HUMAN|HGNC=5861|UniProtKB=A
0M8Q6,HUMAN|HGNC=5647|UniProtKB=A0A087WSY4,HUMAN|H
GNC=5606|UniProtKB=P01763,HUMAN|HGNC=9451|UniProtKB=P
04070,HUMAN|HGNC=11937|UniProtKB=P32970,HUMAN|HGNC=
8569|UniProtKB=P29122,HUMAN|HGNC=5759|UniProtKB=A0A08
7WSZ0,HUMAN|HGNC=5541|UniProtKB=P01871,HUMAN|HGNC=
5585|UniProtKB=A0A0C4DH32,HUMAN|HGNC=3423|UniProtKB=P

11678,HUMAN|HGNC=11740|UniProtKB=P02787,HUMAN|HGNC=
9093|UniProtKB=P55058,HUMAN|Ensembl=ENSG00000282639|U
niProtKB=A0A0J9YX35,HUMAN|HGNC=11766|UniProtKB=P01137,
HUMAN|HGNC=4439|UniProtKB=P07359,HUMAN|HGNC=4187|U
niProtKB=P02774,HUMAN|HGNC=5880|UniProtKB=P01700,HUMA
N|HGNC=5624|UniProtKB=A0A0B4J1X5,HUMAN|HGNC=5905|Uni
ProtKB=P80748,HUMAN|HGNC=5896|UniProtKB=P01715,HUMAN
|HGNC=3153|UniProtKB=Q16610,HUMAN|HGNC=603|UniProtKB=
P04114,HUMAN|HGNC=5478|UniProtKB=P01876,HUMAN|HGNC=
2528|UniProtKB=P53634,HUMAN|HGNC=910|UniProtKB=P25311,
HUMAN|HGNC=9240|UniProtKB=P02775,HUMAN|HGNC=5240|U
niProtKB=P48741,HUMAN|HGNC=3528|UniProtKB=P00742,HUMA
N|HGNC=5156|UniProtKB=P00739,HUMAN|HGNC=10046|UniProt
KB=P12724,HUMAN|HGNC=5895|UniProtKB=P01709,HUMAN|HG
NC=19409|UniProtKB=O43300,HUMAN|HGNC=5619|UniProtKB=A
0A0C4DH42,HUMAN|HGNC=2479|UniProtKB=O76096,HUMAN|H
GNC=5604|UniProtKB=A0A0B4J1X8,HUMAN|HGNC=8993|UniProt
KB=P12273,HUMAN|HGNC=8948|UniProtKB=P29622,HUMAN|HG
NC=2475|UniProtKB=P01034,HUMAN|HGNC=15461|UniProtKB=P
55145,HUMAN|HGNC=1546|UniProtKB=P50454,HUMAN|HGNC=5
821|UniProtKB=A0A075B6H7,HUMAN|HGNC=4827|UniProtKB=P6
8871,HUMAN|HGNC=3530|UniProtKB=P00748,HUMAN|HGNC=58
89|UniProtKB=A0A075B6J9,HUMAN|HGNC=4838|UniProtKB=P055
46,HUMAN|HGNC=10499|UniProtKB=P06702,HUMAN|HGNC=220
9|UniProtKB=P20908,HUMAN|HGNC=5730|UniProtKB=A0A0C4DH
73,HUMAN|HGNC=2092|UniProtKB=Q00610,HUMAN|HGNC=6901
|UniProtKB=P48740,HUMAN|HGNC=5879|UniProtKB=P01699,HU
MAN|HGNC=4555|UniProtKB=P22352,HUMAN|HGNC=5800|UniPr
otKB=A0A075B6S2,HUMAN|HGNC=5645|UniProtKB=A0A0C4DH34
,HUMAN|HGNC=5742|UniProtKB=A0A0C4DH72,HUMAN|HGNC=5
816|UniProtKB=P01624,HUMAN|HGNC=5910|UniProtKB=P01718,
HUMAN|HGNC=8942|UniProtKB=P48595,HUMAN|HGNC=9075|U
niProtKB=P08697,HUMAN|HGNC=6902|UniProtKB=O00187,HUMA
N|HGNC=2312|UniProtKB=P15169,HUMAN|HGNC=16452|UniProt
KB=P0DN86,HUMAN|HGNC=21685|UniProtKB=Q6YHK3,HUMAN|
HGNC=5856|UniProtKB=P0DOY2,HUMAN|HGNC=2367|UniProtKB
=P02741,HUMAN|HGNC=15582|UniProtKB=Q8WXI7,HUMAN|HG
NC=5732|UniProtKB=P04430,HUMAN|HGNC=5733|UniProtKB=P0
1599,HUMAN|HGNC=11756|UniProtKB=Q03403,HUMAN|HGNC=4
798|UniProtKB=Q14520,HUMAN|HGNC=9456|UniProtKB=P07225,
HUMAN|HGNC=15722|UniProtKB=Q8WXQ8,HUMAN|HGNC=5551
|UniProtKB=A0A0C4DH33,HUMAN|HGNC=5927|UniProtKB=P0172
1,HUMAN|HGNC=5918|UniProtKB=A0A075B6K5,HUMAN|HGNC=5
930|UniProtKB=A0A075B6I9,HUMAN|HGNC=5654|UniProtKB=P01
825,HUMAN|HGNC=1247|UniProtKB=P09871,HUMAN|HGNC=555
3|UniProtKB=A0A0A0MS14,HUMAN|Gene=HV108_HUMAN|UniPr
otKB=P0DP01,HUMAN|HGNC=5620|UniProtKB=P01780,HUMAN|
HGNC=5598|UniProtKB=A0A0C4DH35,HUMAN|HGNC=5756|UniPr
otKB=P04432,HUMAN|HGNC=21245|UniProtKB=Q6UXB8,HUMAN
|HGNC=6383|UniProtKB=P01042,HUMAN|HGNC=5655|UniProtKB
=A0A0C4DH41,HUMAN|HGNC=1228|UniProtKB=P05155,HUMAN|
HGNC=5586|UniProtKB=A0A0B4J1V1,HUMAN|HGNC=5622|UniPr
otKB=A0A0B4J1Y9,HUMAN|HGNC=5785|UniProtKB=P06310,HUM
AN|HGNC=5593|UniProtKB=P0DP02,HUMAN|HGNC=6517|UniPro

<table>
<tr><td></td><td>tKB=P18428,HUMAN|HGNC=1690|UniProtKB=O43866,HUMAN|HGNC=2313|UniProtKB=P22792,HUMAN|HGNC=5596|UniProtKB=P01772,HUMAN|HGNC=3551|UniProtKB=P00740,HUMAN|HGNC=5550|UniProtKB=P23083,HUMAN|HGNC=5929|UniProtKB=P04211,HUMAN|HGNC=317|UniProtKB=P02771,HUMAN|HGNC=13572|UniProtKB=Q9Y6R7,HUMAN|HGNC=4829|UniProtKB=P02042,HUMAN|HGNC=5908|UniProtKB=P01717,HUMAN|HGNC=29953|UniProtKB=Q8WWZ8,HUMAN|HGNC=5601|UniProtKB=A0A0C4DH36,HUMAN|HGNC=5716|UniProtKB=P01834,HUMAN|HGNC=5588|UniProtKB=P01764,HUMAN|HGNC=9922|UniProtKB=P02753,HUMAN|HGNC=10569|UniProtKB=P29508,HUMAN|HGNC=8862|UniProtKB=P10720,HUMAN|HGNC=6526|UniProtKB=P80188,HUMAN|HGNC=8955|UniProtKB=P50453</td><td></td></tr>
<tr><td>GO:0006402</td><td>HUMAN|HGNC=5046|UniProtKB=P52272,HUMAN|HGNC=17150|UniProtKB=Q9Y2V2,HUMAN|HGNC=5048|UniProtKB=Q00839</td><td>mRNA catabolic process</td></tr>
<tr><td>PC00214</td><td>HUMAN|HGNC=29327|UniProtKB=Q5VZ46,HUMAN|HGNC=2047|UniProtKB=O00501,HUMAN|HGNC=11828|UniProtKB=Q9UDY2</td><td>tight junction</td></tr>
<tr><td>GO:0006024</td><td>HUMAN|HGNC=24293|UniProtKB=Q70JA7</td><td>glycosaminoglycan biosynthetic process</td></tr>
<tr><td>GO:0110165</td><td>HUMAN|HGNC=11317|UniProtKB=Q04837</td><td>cellular anatomical entity</td></tr>
<tr><td>GO:0010212</td><td>HUMAN|HGNC=28771|UniProtKB=Q9BVC6,HUMAN|HGNC=533|UniProtKB=P04083</td><td>response to ionizing radiation</td></tr>
<tr><td>GO:0000993</td><td>HUMAN|HGNC=5048|UniProtKB=Q00839</td><td>RNA polymerase II complex binding</td></tr>
<tr><td>GO:0007249</td><td>HUMAN|HGNC=30140|UniProtKB=A7MCY6,HUMAN|HGNC=16391|UniProtKB=Q9H257,HUMAN|HGNC=16276|UniProtKB=Q9C035,HUMAN|HGNC=29890|UniProtKB=Q86UT6,HUMAN|HGNC=9275|UniProtKB=P35813</td><td>I-kappaB kinase/NF-kappaB signaling</td></tr>
<tr><td>GO:0009986</td><td>HUMAN|HGNC=453|UniProtKB=P02760,HUMAN|HGNC=5253|UniProtKB=P07900,HUMAN|HGNC=6156|UniProtKB=P05106,HUMAN|HGNC=30167|UniProtKB=P13667,HUMAN|HGNC=5258|UniProtKB=P08238,HUMAN|HGNC=10661|UniProtKB=P31431,HUMAN|HGNC=6153|UniProtKB=P05556,HUMAN|HGNC=8569|UniProtKB=P29122,HUMAN|HGNC=29953|UniProtKB=Q8WWZ8,HUMAN|HGNC=4606|UniProtKB=P30101</td><td>cell surface</td></tr>
<tr><td>PC00034</td><td>HUMAN|HGNC=11442|UniProtKB=O15400,HUMAN|HGNC=11429|UniProtKB=O75558,HUMAN|HGNC=11133|UniProtKB=O95721,HUMAN|HGNC=11131|UniProtKB=O00161</td><td>SNARE protein</td></tr>
<tr><td>GO:0050727</td><td>HUMAN|HGNC=533|UniProtKB=P04083</td><td>regulation of inflammatory response</td></tr>
<tr><td>GO:0000813</td><td>HUMAN|HGNC=25153|UniProtKB=Q96EY5</td><td>ESCRT I complex</td></tr>
<tr><td>GO:0030992</td><td>HUMAN|HGNC=30391|UniProtKB=Q9UG01,HUMAN|HGNC=14313|UniProtKB=Q8WYA0,HUMAN|HGNC=29262|UniProtKB=Q9P2H3</td><td>intraciliary transport particle B</td></tr>
<tr><td>GO:0043277</td><td>HUMAN|HGNC=18756|UniProtKB=Q9BYZ6,HUMAN|HGNC=9802|UniProtKB=P15153,HUMAN|HGNC=9801|UniProtKB=P63000,HUMAN|HGNC=533|UniProtKB=P04083</td><td>apoptotic cell clearance</td></tr>
<tr><td>GO:1905475</td><td>HUMAN|HGNC=26486|UniProtKB=Q6PJW8,HUMAN|HGNC=17643|UniProtKB=Q9HD26</td><td>regulation of protein localization to membrane</td></tr>
<tr><td>GO:0005840</td><td>HUMAN|HGNC=30547|UniProtKB=Q96K17</td><td>ribosome</td></tr>
<tr><td>GO:0046434</td><td>HUMAN|HGNC=4456|UniProtKB=P43304,HUMAN|HGNC=6050|UniProtKB=P29218</td><td>organophosphate catabolic process</td></tr>
</table>

GO:0019318	HUMAN|HGNC=4116|UniProtKB=Q14376	hexose metabolic process
GO:0002443	HUMAN|HGNC=11491|UniProtKB=P43405	leukocyte mediated immunity
GO:0000778	HUMAN|HGNC=9112|UniProtKB=Q6P1K2	condensed nuclear chromosome kinetochore
PC00088	HUMAN|HGNC=469|UniProtKB=Q01433	deaminase
GO:0032300	HUMAN|HGNC=7127|UniProtKB=P40692,HUMAN|HGNC=7326|UniProtKB=P20585	mismatch repair complex
GO:0004869	HUMAN|HGNC=2479|UniProtKB=O76096,HUMAN|HGNC=6383|UniProtKB=P01042,HUMAN|HGNC=2482|UniProtKB=P04080	cysteine-type endopeptidase inhibitor activity
GO:0006310	HUMAN|HGNC=4716|UniProtKB=P16403,HUMAN|HGNC=4718|UniProtKB=P10412,HUMAN|HGNC=4719|UniProtKB=P16401	DNA recombination
GO:0097712	HUMAN|HGNC=15476|UniProtKB=P63167,HUMAN|HGNC=15848|UniProtKB=Q14141,HUMAN|HGNC=16053|UniProtKB=Q8N1V2,HUMAN|HGNC=20908|UniProtKB=Q86YF9,HUMAN|HGNC=2952|UniProtKB=Q96JB1,HUMAN|HGNC=25631|UniProtKB=Q96MT7,HUMAN|HGNC=1717|UniProtKB=Q16181,HUMAN|HGNC=3244|UniProtKB=Q9NZN3,HUMAN|HGNC=29262|UniProtKB=Q9P2H3,HUMAN|HGNC=7729|UniProtKB=Q15019	vesicle targeting, trans-Golgi to periciliary membrane compartment
GO:0005794	HUMAN|HGNC=692|UniProtKB=P40616,HUMAN|HGNC=8761|UniProtKB=Q9BUL8,HUMAN|HGNC=2479|UniProtKB=O76096,HUMAN|HGNC=14902|UniProtKB=Q9NRW1,HUMAN|HGNC=21354|UniProtKB=Q86XR7,HUMAN|HGNC=24251|UniProtKB=Q9Y3A6,HUMAN|HGNC=555|UniProtKB=O43747,HUMAN|HGNC=17643|UniProtKB=Q9HD26,HUMAN|HGNC=9786|UniProtKB=P20340,HUMAN|HGNC=16075|UniProtKB=Q9H082,HUMAN|HGNC=16998|UniProtKB=P49755,HUMAN|HGNC=16996|UniProtKB=Q15363,HUMAN|HGNC=24878|UniProtKB=Q9BVK6	Golgi apparatus
GO:0051306	HUMAN|HGNC=15713|UniProtKB=Q9UJX4,HUMAN|HGNC=12401|UniProtKB=P33981,HUMAN|HGNC=14452|UniProtKB=Q9NYG5	mitotic sister chromatid separation
GO:0008134	HUMAN|HGNC=30025|UniProtKB=Q5VV67,HUMAN|HGNC=6767|UniProtKB=Q15797,HUMAN|HGNC=5048|UniProtKB=Q00839	transcription factor binding
GO:0004518	HUMAN|HGNC=30646|UniProtKB=Q7KZF4	nuclease activity
GO:0070527	HUMAN|HGNC=23151|UniProtKB=Q86UX7,HUMAN|HGNC=3662|UniProtKB=P02675,HUMAN|HGNC=11491|UniProtKB=P43405,HUMAN|HGNC=11845|UniProtKB=Q9Y490,HUMAN|HGNC=29884|UniProtKB=Q8TF42	platelet aggregation
GO:0010720	HUMAN|HGNC=6616|UniProtKB=P48059,HUMAN|HGNC=243|UniProtKB=P35611	positive regulation of cell development
GO:0120035	HUMAN|HGNC=12690|UniProtKB=P09327,HUMAN|HGNC=25361|UniProtKB=Q8N264,HUMAN|HGNC=21295|UniProtKB=Q8TE68	regulation of plasma membrane bounded cell projection organization
GO:0005539	HUMAN|HGNC=8601|UniProtKB=Q06141,HUMAN|HGNC=1681|UniProtKB=P16070,HUMAN|HGNC=30013|UniProtKB=Q96PD5,HUMAN|HGNC=544|UniProtKB=P08133	glycosaminoglycan binding
GO:0098969	HUMAN|HGNC=7007|UniProtKB=P61006	neurotransmitter receptor transport to postsynaptic membrane
GO:0030140	HUMAN|HGNC=25951|UniProtKB=Q6ULP2,HUMAN|HGNC=31332|UniProtKB=Q6IQ22,HUMAN|HGNC=7007|UniProtKB=P61006,HU	trans-Golgi network transport vesicle

<table>
<tr><td></td><td>MAN|HGNC=555|UniProtKB=O43747,HUMAN|HGNC=17643|UniProtKB=Q9HD26</td><td></td></tr>
<tr><td>GO:0006909</td><td>HUMAN|HGNC=5575|UniProtKB=A0A0B4J1V2,HUMAN|HGNC=5645|UniProtKB=A0A0C4DH34,HUMAN|HGNC=5549|UniProtKB=A0A0C4DH31,HUMAN|HGNC=5554|UniProtKB=P01743,HUMAN|HGNC=5480|UniProtKB=P01880,HUMAN|HGNC=5659|UniProtKB=A0A0C4DH38,HUMAN|HGNC=5634|UniProtKB=A0A075B7F0,HUMAN|HGNC=38476|UniProtKB=B9A064,HUMAN|HGNC=5861|UniProtKB=A0M8Q6,HUMAN|HGNC=5647|UniProtKB=A0A087WSY4,HUMAN|HGNC=5563|UniProtKB=A0A075B7D0,HUMAN|HGNC=5856|UniProtKB=P0DOY2,HUMAN|HGNC=5606|UniProtKB=P01763,HUMAN|HGNC=9495|UniProtKB=P24158,HUMAN|HGNC=5576|UniProtKB=P01817,HUMAN|HGNC=5528|UniProtKB=P01861,HUMAN|HGNC=5541|UniProtKB=P01871,HUMAN|HGNC=5585|UniProtKB=A0A0C4DH32,HUMAN|HGNC=535|UniProtKB=P50995,HUMAN|HGNC=3309|UniProtKB=P08246,HUMAN|HGNC=5551|UniProtKB=A0A0C4DH33,HUMAN|HGNC=5526|UniProtKB=P01859,HUMAN|HGNC=5657|UniProtKB=P0DP08,HUMAN|Ensembl=ENSG00000282639|UniProtKB=A0A0J9YX35,HUMAN|Gene=HV309_HUMAN|UniProtKB=P01782,HUMAN|HGNC=5654|UniProtKB=P01825,HUMAN|HGNC=5553|UniProtKB=A0A0A0MS14,HUMAN|Gene=HV108_HUMAN|UniProtKB=P0DP01,HUMAN|HGNC=5624|UniProtKB=A0A0B4J1X5,HUMAN|HGNC=5620|UniProtKB=P01780,HUMAN|HGNC=5598|UniProtKB=A0A0C4DH35,HUMAN|HGNC=5580|UniProtKB=P01762,HUMAN|HGNC=5633|UniProtKB=A0A075B7D8,HUMAN|HGNC=5582|UniProtKB=A0A0B4J1V0,HUMAN|HGNC=5478|UniProtKB=P01876,HUMAN|HGNC=9788|UniProtKB=P51149,HUMAN|HGNC=5617|UniProtKB=A0A075B6Q5,HUMAN|HGNC=5594|UniProtKB=P0DP03,HUMAN|HGNC=5655|UniProtKB=A0A0C4DH41,HUMAN|HGNC=5636|UniProtKB=A0A075B7B8,HUMAN|HGNC=5586|UniProtKB=A0A0B4J1V1,HUMAN|HGNC=5581|UniProtKB=P01766,HUMAN|HGNC=5622|UniProtKB=A0A0B4J1Y9,HUMAN|HGNC=5652|UniProtKB=A0A075B6R2,HUMAN|HGNC=5593|UniProtKB=P0DP02,HUMAN|HGNC=5479|UniProtKB=P01877,HUMAN|HGNC=5619|UniProtKB=A0A0C4DH42,HUMAN|HGNC=5596|UniProtKB=P01772,HUMAN|HGNC=5550|UniProtKB=P23083,HUMAN|HGNC=5604|UniProtKB=A0A0B4J1X8,HUMAN|HGNC=5525|UniProtKB=P01857,HUMAN|HGNC=5662|UniProtKB=A0A0B4J1U7,HUMAN|HGNC=5601|UniProtKB=A0A0C4DH36,HUMAN|HGNC=5716|UniProtKB=P01834,HUMAN|HGNC=5588|UniProtKB=P01764,HUMAN|HGNC=5857|UniProtKB=P0DOY3,HUMAN|HGNC=5610|UniProtKB=P01767,HUMAN|Ensembl=ENSG00000282150|UniProtKB=A0A0J9YY99,HUMAN|HGNC=5527|UniProtKB=P01860</td><td>phagocytosis</td></tr>
<tr><td>GO:0034613</td><td>HUMAN|HGNC=26068|UniProtKB=Q6IAA8,HUMAN|HGNC=20120|UniProtKB=Q17RC7,HUMAN|HGNC=9621|UniProtKB=Q6IBS0,HUMAN|HGNC=9164|UniProtKB=Q99719,HUMAN|HGNC=11881|UniProtKB=P62328</td><td>cellular protein localization</td></tr>
<tr><td>GO:0034381</td><td>HUMAN|HGNC=607|UniProtKB=P02654</td><td>plasma lipoprotein particle clearance</td></tr>
<tr><td>GO:0031307</td><td>HUMAN|HGNC=21689|UniProtKB=Q9Y3D6</td><td>integral component of mitochondrial outer membrane</td></tr>
<tr><td>GO:0001706</td><td>HUMAN|HGNC=2188|UniProtKB=Q99715</td><td>endoderm formation</td></tr>
</table>

GO:0000811	HUMAN|HGNC=26200|UniProtKB=Q9H668	GINS complex
PC00103	HUMAN|HGNC=3602|UniProtKB=Q9UBX5,HUMAN|HGNC=2209|UniProtKB=P20908,HUMAN|HGNC=6714|UniProtKB=Q14766,HUMAN|HGNC=2188|UniProtKB=Q99715,HUMAN|HGNC=2211|UniProtKB=P12109,HUMAN|HGNC=6922|UniProtKB=P11226,HUMAN|HGNC=15855|UniProtKB=Q9NPY3,HUMAN|HGNC=3218|UniProtKB=Q12805,HUMAN|HGNC=2213|UniProtKB=P12111	extracellular matrix structural protein
GO:0032870	HUMAN|HGNC=7670|UniProtKB=Q9Y6Q9	cellular response to hormone stimulus
GO:0006482	HUMAN|HGNC=30178|UniProtKB=Q9Y570	protein demethylation
GO:0032869	HUMAN|HGNC=31332|UniProtKB=Q6IQ22,HUMAN|HGNC=7007|UniProtKB=P61006,HUMAN|HGNC=9021|UniProtKB=P14618,HUMAN|HGNC=14451|UniProtKB=Q9BQK8	cellular response to insulin stimulus
GO:0016247	HUMAN|HGNC=1406|UniProtKB=Q9Y698	channel regulator activity
GO:0046632	HUMAN|HGNC=6171|UniProtKB=Q08881,HUMAN|HGNC=533|UniProtKB=P04083	alpha-beta T cell differentiation
PC00100	HUMAN|HGNC=11219|UniProtKB=P09486,HUMAN|HGNC=2464|UniProtKB=P13611	extracellular matrix glycoprotein
PC00065	HUMAN|HGNC=8898|UniProtKB=P07205,HUMAN|HGNC=8876|UniProtKB=P17858,HUMAN|HGNC=8878|UniProtKB=Q01813	carbohydrate kinase
GO:0002218	HUMAN|HGNC=7183|UniProtKB=P41218	activation of innate immune response
GO:0007507	HUMAN|HGNC=27424|UniProtKB=Q5T481,HUMAN|HGNC=3778|UniProtKB=P02751,HUMAN|HGNC=2067|UniProtKB=O00151,HUMAN|HGNC=17468|UniProtKB=Q96HC4,HUMAN|HGNC=22958|UniProtKB=Q9NR12	heart development
PC00108	HUMAN|HGNC=28101|UniProtKB=Q2M2H8,HUMAN|HGNC=4138|UniProtKB=Q14697	glucosidase
GO:0032501	HUMAN|HGNC=9065|UniProtKB=P19174,HUMAN|HGNC=613|UniProtKB=P02649,HUMAN|HGNC=1869|UniProtKB=P11597,HUMAN|HGNC=9093|UniProtKB=P55058	multicellular organismal process
GO:0006605	HUMAN|HGNC=13206|UniProtKB=O60296	protein targeting
GO:0043177	HUMAN|HGNC=4827|UniProtKB=P68871,HUMAN|HGNC=4831|UniProtKB=P69891,HUMAN|HGNC=4823|UniProtKB=P69905,HUMAN|HGNC=4829|UniProtKB=P02042	organic acid binding
PC00056	HUMAN|HGNC=18808|UniProtKB=Q9Y6E2	basic leucine zipper transcription factor
GO:0061061	HUMAN|HGNC=2067|UniProtKB=O00151,HUMAN|HGNC=17468|UniProtKB=Q96HC4,HUMAN|HGNC=22958|UniProtKB=Q9NR12	muscle structure development
GO:0003785	HUMAN|HGNC=9621|UniProtKB=Q6IBS0,HUMAN|HGNC=11881|UniProtKB=P62328	actin monomer binding
GO:0005496	HUMAN|HGNC=4187|UniProtKB=P02774,HUMAN|HGNC=10839|UniProtKB=P04278	steroid binding
GO:0030258	HUMAN|HGNC=30244|UniProtKB=Q6P1A2,HUMAN|HGNC=2850|UniProtKB=Q9Y6T7	lipid modification
GO:0090303	HUMAN|HGNC=3537|UniProtKB=P25116,HUMAN|HGNC=533|UniProtKB=P04083	positive regulation of wound healing
PC00143	HUMAN|HGNC=1165|UniProtKB=Q9Y4D2,HUMAN|HGNC=20175|UniProtKB=Q9Y3E0	lipase

GO:0043539	HUMAN|HGNC=20292|UniProtKB=Q9Y376	protein serine/threonine kinase activator activity
GO:0017147	HUMAN|HGNC=4045|UniProtKB=O75084,HUMAN|HGNC=11119|UniProtKB=Q99835	Wnt-protein binding
GO:0007346	HUMAN|HGNC=18673|UniProtKB=Q96JB5	regulation of mitotic cell cycle
GO:0016485	HUMAN|HGNC=2479|UniProtKB=O76096,HUMAN|HGNC=8569|UniProtKB=P29122,HUMAN|HGNC=2312|UniProtKB=P15169	protein processing
GO:0016311	HUMAN|HGNC=5461|UniProtKB=P78318,HUMAN|HGNC=6050|UniProtKB=P29218	dephosphorylation
GO:0030658	HUMAN|HGNC=25951|UniProtKB=Q6ULP2,HUMAN|HGNC=555|UniProtKB=O43747	transport vesicle membrane
GO:0030276	HUMAN|HGNC=25951|UniProtKB=Q6ULP2,HUMAN|HGNC=11984|UniProtKB=Q6ZVM7,HUMAN|HGNC=11510|UniProtKB=Q8N9I0,HUMAN|HGNC=555|UniProtKB=O43747,HUMAN|HGNC=23186|UniProtKB=Q14677,HUMAN|HGNC=2092|UniProtKB=Q00610,HUMAN|HGNC=13667|UniProtKB=Q9BXS5,HUMAN|HGNC=2090|UniProtKB=P09496,HUMAN|HGNC=561|UniProtKB=O95782,HUMAN|HGNC=11512|UniProtKB=Q9H2B2	clathrin binding
GO:0090575	HUMAN|HGNC=6767|UniProtKB=Q15797,HUMAN|HGNC=23688|UniProtKB=Q86UU0,HUMAN|HGNC=5048|UniProtKB=Q00839	RNA polymerase II transcription regulator complex
GO:0031109	HUMAN|HGNC=6891|UniProtKB=Q15555,HUMAN|HGNC=6890|UniProtKB=Q15691	microtubule polymerization or depolymerization
GO:0030014	HUMAN|HGNC=9207|UniProtKB=Q9UFF9	CCR4-NOT complex
GO:0048585	HUMAN|HGNC=533|UniProtKB=P04083	negative regulation of response to stimulus
GO:0022607	HUMAN|HGNC=3602|UniProtKB=Q9UBX5,HUMAN|HGNC=15855|UniProtKB=Q9NPY3	cellular component assembly
GO:0045321	HUMAN|HGNC=2479|UniProtKB=O76096,HUMAN|HGNC=9353|UniProtKB=P32119,HUMAN|HGNC=9352|UniProtKB=Q06830,HUMAN|HGNC=16961|UniProtKB=Q15762	leukocyte activation
GO:0043666	HUMAN|HGNC=1173|UniProtKB=Q5H9R7	regulation of phosphoprotein phosphatase activity
GO:0090114	HUMAN|HGNC=10534|UniProtKB=Q9NR31,HUMAN|HGNC=10701|UniProtKB=Q15436	COPII-coated vesicle budding
GO:1902495	HUMAN|HGNC=806|UniProtKB=P54709	transmembrane transporter complex
GO:0005507	HUMAN|HGNC=11179|UniProtKB=P00441,HUMAN|HGNC=11138|UniProtKB=P37840,HUMAN|HGNC=21101|UniProtKB=O60888,HUMAN|HGNC=2689|UniProtKB=P09172	copper ion binding
GO:0006936	HUMAN|HGNC=11873|UniProtKB=Q9NYL9,HUMAN|HGNC=12012|UniProtKB=P06753,HUMAN|HGNC=544|UniProtKB=P08133,HUMAN|HGNC=12013|UniProtKB=P67936,HUMAN|HGNC=12011|UniProtKB=P07951	muscle contraction
GO:0030176	HUMAN|HGNC=30550|UniProtKB=O60337,HUMAN|HGNC=17943|UniProtKB=Q9GZP9,HUMAN|HGNC=24175|UniProtKB=Q9P035	integral component of endoplasmic reticulum membrane
GO:0003887	HUMAN|HGNC=14060|UniProtKB=Q9UBZ9	DNA-directed DNA polymerase activity

GO:0005929	HUMAN|HGNC=14313|UniProtKB=Q8WYA0,HUMAN|HGNC=26664|UniProtKB=Q8N841,HUMAN|HGNC=11119|UniProtKB=Q99835,HUMAN|HGNC=29262|UniProtKB=Q9P2H3	cilium
GO:0007039	HUMAN|HGNC=11358|UniProtKB=O75886,HUMAN|HGNC=11357|UniProtKB=Q92783	protein catabolic process in the vacuole
GO:0006631	HUMAN|HGNC=2328|UniProtKB=P50416	fatty acid metabolic process
PC00172	HUMAN|HGNC=9465|UniProtKB=P11908,HUMAN|HGNC=7413|UniProtKB=Q13126,HUMAN|HGNC=29035|UniProtKB=O43314,HUMAN|HGNC=362|UniProtKB=P54819,HUMAN|HGNC=363|UniProtKB=P27144,HUMAN|HGNC=361|UniProtKB=P00568,HUMAN|HGNC=18170|UniProtKB=P30085,HUMAN|HGNC=17376|UniProtKB=Q9UIJ7,HUMAN|HGNC=7892|UniProtKB=P00491,HUMAN|HGNC=9462|UniProtKB=P60891	nucleotide kinase
GO:0036158	HUMAN|HGNC=2952|UniProtKB=Q96JB1	outer dynein arm assembly
GO:0048646	HUMAN|HGNC=12652|UniProtKB=P50552,HUMAN|HGNC=13937|UniProtKB=O95866	anatomical structure formation involved in morphogenesis
GO:0010033	HUMAN|HGNC=253|UniProtKB=P11766,HUMAN|HGNC=11820|UniProtKB=P01033,HUMAN|HGNC=251|UniProtKB=P00326,HUMAN|HGNC=255|UniProtKB=P28332	response to organic substance
GO:0050868	HUMAN|HGNC=533|UniProtKB=P04083	negative regulation of T cell activation
GO:0070603	HUMAN|HGNC=29989|UniProtKB=Q86YP4	SWI/SNF superfamily-type complex
GO:0016597	HUMAN|HGNC=20|UniProtKB=P49588	amino acid binding
GO:0097352	HUMAN|HGNC=6501|UniProtKB=P13473,HUMAN|HGNC=12666|UniProtKB=P55072	autophagosome maturation
GO:0071162	HUMAN|HGNC=26200|UniProtKB=Q9H668	CMG complex
GO:0048016	HUMAN|HGNC=3537|UniProtKB=P25116,HUMAN|HGNC=9314|UniProtKB=Q08209,HUMAN|HGNC=4472|UniProtKB=Q14330,HUMAN|HGNC=4390|UniProtKB=P50148,HUMAN|HGNC=3540|UniProtKB=Q96RI0	inositol phosphate-mediated signaling
GO:0010557	HUMAN|HGNC=6517|UniProtKB=P18428	positive regulation of macromolecule biosynthetic process
PC00002	HUMAN|HGNC=14247|UniProtKB=O75964,HUMAN|HGNC=837|UniProtKB=P30049,HUMAN|HGNC=846|UniProtKB=P56385,HUMAN|HGNC=845|UniProtKB=O75947,HUMAN|HGNC=851|UniProtKB=P38606,HUMAN|HGNC=833|UniProtKB=P36542,HUMAN|HGNC=823|UniProtKB=P25705,HUMAN|HGNC=830|UniProtKB=P06576,HUMAN|HGNC=850|UniProtKB=P48047	ATP synthase
GO:0000266	HUMAN|HGNC=21689|UniProtKB=Q9Y3D6,HUMAN|HGNC=2973|UniProtKB=O00429,HUMAN|HGNC=2974|UniProtKB=P50570,HUMAN|HGNC=29125|UniProtKB=Q9UQ16	mitochondrial fission
GO:0090287	HUMAN|HGNC=1692|UniProtKB=P08962	regulation of cellular response to growth factor stimulus
GO:0006165	HUMAN|HGNC=363|UniProtKB=P27144,HUMAN|HGNC=361|UniProtKB=P00568	nucleoside diphosphate phosphorylation

<table>
<tr><td>PC00092</td><td>HUMAN|HGNC=253|UniProtKB=P11766,HUMAN|HGNC=1062|UniProtKB=P53004,HUMAN|HGNC=4141|UniProtKB=P04406,HUMAN|HGNC=7863|UniProtKB=Q13423,HUMAN|HGNC=5382|UniProtKB=O75874,HUMAN|HGNC=6970|UniProtKB=P40925,HUMAN|HGNC=4057|UniProtKB=P11413,HUMAN|HGNC=4335|UniProtKB=P00367,HUMAN|HGNC=6535|UniProtKB=P00338,HUMAN|HGNC=8891|UniProtKB=P52209,HUMAN|HGNC=18399|UniProtKB=Q8N5I4,HUMAN|HGNC=6971|UniProtKB=P40926,HUMAN|HGNC=6541|UniProtKB=P07195,HUMAN|HGNC=17964|UniProtKB=Q8TC12,HUMAN|HGNC=251|UniProtKB=P00326,HUMAN|HGNC=4456|UniProtKB=P43304,HUMAN|HGNC=18647|UniProtKB=Q8WWV3,HUMAN|HGNC=92|UniProtKB=P49748,HUMAN|HGNC=7717|UniProtKB=P19404,HUMAN|HGNC=5383|UniProtKB=P48735,HUMAN|HGNC=255|UniProtKB=P28332,HUMAN|HGNC=5386|UniProtKB=P51553,HUMAN|HGNC=412|UniProtKB=P49189</td><td>dehydrogenase</td></tr>
<tr><td>GO:0006749</td><td>HUMAN|HGNC=4635|UniProtKB=P21266,HUMAN|HGNC=4553|UniProtKB=P07203,HUMAN|HGNC=4634|UniProtKB=P28161,HUMAN|HGNC=4312|UniProtKB=P48507,HUMAN|HGNC=4632|UniProtKB=P09488,HUMAN|HGNC=4623|UniProtKB=P00390,HUMAN|HGNC=4638|UniProtKB=P09211</td><td>glutathione metabolic process</td></tr>
<tr><td>PC00141</td><td>HUMAN|HGNC=8533|UniProtKB=P51575,HUMAN|HGNC=2153|UniProtKB=Q9NQW8</td><td>ligand-gated ion channel</td></tr>
<tr><td>PC00091</td><td>HUMAN|HGNC=395|UniProtKB=P13716</td><td>dehydratase</td></tr>
<tr><td>GO:0035869</td><td>HUMAN|HGNC=26555|UniProtKB=Q3SY00,HUMAN|HGNC=17060|UniProtKB=Q92845</td><td>ciliary transition zone</td></tr>
<tr><td>GO:0016592</td><td>HUMAN|HGNC=11957|UniProtKB=Q93074</td><td>mediator complex</td></tr>
<tr><td>GO:0050818</td><td>HUMAN|HGNC=11491|UniProtKB=P43405,HUMAN|HGNC=3537|UniProtKB=P25116</td><td>regulation of coagulation</td></tr>
<tr><td>GO:0004864</td><td>HUMAN|HGNC=14871|UniProtKB=Q96A00,HUMAN|HGNC=14952|UniProtKB=Q8TAE6</td><td>protein phosphatase inhibitor activity</td></tr>
<tr><td>GO:0042734</td><td>HUMAN|HGNC=8515|UniProtKB=Q9HC10</td><td>presynaptic membrane</td></tr>
<tr><td>PC00070</td><td>HUMAN|HGNC=6616|UniProtKB=P48059</td><td>cell junction protein</td></tr>
<tr><td>GO:0016491</td><td>HUMAN|HGNC=8876|UniProtKB=P17858,HUMAN|Gene=SEP15|UniProtKB=O60613,HUMAN|HGNC=11179|UniProtKB=P00441,HUMAN|HGNC=4141|UniProtKB=P04406,HUMAN|HGNC=7863|UniProtKB=Q13423,HUMAN|HGNC=11180|UniProtKB=P04179,HUMAN|HGNC=9021|UniProtKB=P14618,HUMAN|HGNC=8878|UniProtKB=Q01813,HUMAN|HGNC=4456|UniProtKB=P43304,HUMAN|HGNC=4335|UniProtKB=P00367,HUMAN|HGNC=7717|UniProtKB=P19404,HUMAN|HGNC=412|UniProtKB=P49189</td><td>oxidoreductase activity</td></tr>
<tr><td>GO:0051250</td><td>HUMAN|HGNC=18233|UniProtKB=Q8NDB2</td><td>negative regulation of lymphocyte activation</td></tr>
<tr><td>GO:0043197</td><td>HUMAN|HGNC=7007|UniProtKB=P61006,HUMAN|HGNC=15474|UniProtKB=Q9Y566,HUMAN|HGNC=1736|UniProtKB=P60953,HUMAN|HGNC=2974|UniProtKB=P50570,HUMAN|HGNC=9801|UniProtKB=P63000,HUMAN|HGNC=29125|UniProtKB=Q9UQ16,HUMAN|HGNC=667|UniProtKB=P61586,HUMAN|HGNC=14294|UniProtKB=Q9BYB0</td><td>dendritic spine</td></tr>
<tr><td>GO:0000932</td><td>HUMAN|HGNC=17259|UniProtKB=Q9Y4Z0,HUMAN|HGNC=29190|UniProtKB=Q9UPQ9,HUMAN|HGNC=20250|UniProtKB=Q96IF1,HUMAN|HGNC=9207|UniProtKB=Q9UFF9</td><td>P-body</td></tr>
</table>

GO:0019985	HUMAN|HGNC=14060|UniProtKB=Q9UBZ9	translesion synthesis
GO:0050684	HUMAN|HGNC=21605|UniProtKB=Q14151	regulation of mRNA processing
GO:2000780	HUMAN|HGNC=23077|UniProtKB=Q96FW1	negative regulation of double-strand break repair
GO:0009605	HUMAN|HGNC=2479|UniProtKB=O76096,HUMAN|HGNC=11491|UniProtKB=P43405,HUMAN|HGNC=3537|UniProtKB=P25116,HUMAN|HGNC=6383|UniProtKB=P01042,HUMAN|HGNC=7967|UniProtKB=Q96RI1,HUMAN|HGNC=2300|UniProtKB=Q96IY4	response to external stimulus
GO:2000116	HUMAN|HGNC=2479|UniProtKB=O76096	regulation of cysteine-type endopeptidase activity
GO:0043933	HUMAN|HGNC=1869|UniProtKB=P11597,HUMAN|HGNC=9093|UniProtKB=P55058	protein-containing complex subunit organization
GO:0017025	HUMAN|HGNC=9552|UniProtKB=P62195	TBP-class protein binding
GO:0009887	HUMAN|HGNC=15474|UniProtKB=Q9Y566,HUMAN|HGNC=544|UniProtKB=P08133,HUMAN|HGNC=12601|UniProtKB=O75445,HUMAN|HGNC=7002|UniProtKB=A6NDR6,HUMAN|HGNC=885|UniProtKB=O75882,HUMAN|HGNC=14294|UniProtKB=Q9BYB0	animal organ morphogenesis
GO:0018024	HUMAN|HGNC=7133|UniProtKB=O14686,HUMAN|HGNC=13726|UniProtKB=Q8NEZ4	histone-lysine N-methyltransferase activity
GO:0030027	HUMAN|HGNC=3338|UniProtKB=Q14247,HUMAN|HGNC=12690|UniProtKB=P09327,HUMAN|HGNC=11148|UniProtKB=Q16658,HUMAN|HGNC=12733|UniProtKB=Q9Y6W5,HUMAN|HGNC=2695|UniProtKB=Q16643,HUMAN|HGNC=7590|UniProtKB=Q15746,HUMAN|HGNC=2696|UniProtKB=Q9UJU6,HUMAN|HGNC=14653|UniProtKB=Q9HBI1,HUMAN|HGNC=4844|UniProtKB=P14317,HUMAN|HGNC=11320|UniProtKB=Q8IZP0	lamellipodium
GO:0030332	HUMAN|HGNC=24224|UniProtKB=Q9NYV4	cyclin binding
GO:0061077	HUMAN|HGNC=5241|UniProtKB=P11142,HUMAN|HGNC=5269|UniProtKB=P61604,HUMAN|HGNC=5239|UniProtKB=P17066,HUMAN|HGNC=30594|UniProtKB=Q9H3U1,HUMAN|HGNC=5238|UniProtKB=P11021,HUMAN|HGNC=5270|UniProtKB=P25685,HUMAN|HGNC=5240|UniProtKB=P48741,HUMAN|HGNC=11343|UniProtKB=P50502	chaperone-mediated protein folding
GO:0019221	HUMAN|HGNC=9240|UniProtKB=P02775,HUMAN|HGNC=19100|UniProtKB=Q5VWK5,HUMAN|HGNC=11364|UniProtKB=P40763,HUMAN|HGNC=10632|UniProtKB=P13501,HUMAN|HGNC=8862|UniProtKB=P10720,HUMAN|HGNC=8861|UniProtKB=P02776	cytokine-mediated signaling pathway
GO:0010950	HUMAN|HGNC=9568|UniProtKB=Q06323	positive regulation of endopeptidase activity
GO:0006584	HUMAN|HGNC=2689|UniProtKB=P09172	catecholamine metabolic process
GO:0048015	HUMAN|HGNC=9055|UniProtKB=Q00722	phosphatidylinositol-mediated signaling
GO:0045124	HUMAN|HGNC=29884|UniProtKB=Q8TF42	regulation of bone resorption
GO:0044390	HUMAN|HGNC=16476|UniProtKB=Q9H0E2	ubiquitin-like protein conjugating enzyme binding
GO:0030866	HUMAN|HGNC=10252|UniProtKB=O75116,HUMAN|HGNC=17905|UniProtKB=Q9Y613	cortical actin cytoskeleton organization

<table>
<tr><th></th><th></th><th></th></tr>
<tr><td>GO:0071218</td><td>HUMAN|HGNC=12666|UniProtKB=P55072,HUMAN|HGNC=15663|UniProtKB=Q9NYU2</td><td>cellular response to misfolded protein</td></tr>
<tr><td>GO:0017056</td><td>HUMAN|HGNC=8062|UniProtKB=P49790,HUMAN|HGNC=17859|UniProtKB=Q5SRE5</td><td>structural constituent of nuclear pore</td></tr>
<tr><td>GO:0007155</td><td>HUMAN|HGNC=8710|UniProtKB=Q9Y5G1,HUMAN|HGNC=16051|UniProtKB=Q8TEW0,HUMAN|HGNC=1681|UniProtKB=P16070,HUMAN|HGNC=2209|UniProtKB=P20908,HUMAN|HGNC=14674|UniProtKB=Q96QU1,HUMAN|HGNC=2047|UniProtKB=O00501,HUMAN|HGNC=11283|UniProtKB=P12931,HUMAN|HGNC=11771|UniProtKB=Q15582</td><td>cell adhesion</td></tr>
<tr><td>PC00155</td><td>HUMAN|HGNC=8728|UniProtKB=P22061,HUMAN|HGNC=24550|UniProtKB=Q9H8H3,HUMAN|HGNC=12591|UniProtKB=P06132</td><td>methyltransferase</td></tr>
<tr><td>GO:0060491</td><td>HUMAN|HGNC=9621|UniProtKB=Q6IBS0,HUMAN|HGNC=25361|UniProtKB=Q8N264,HUMAN|HGNC=21295|UniProtKB=Q8TE68</td><td>regulation of cell projection assembly</td></tr>
<tr><td>GO:0030427</td><td>HUMAN|HGNC=3338|UniProtKB=Q14247,HUMAN|HGNC=2695|UniProtKB=Q16643,HUMAN|HGNC=2696|UniProtKB=Q9UJU6,HUMAN|HGNC=4844|UniProtKB=P14317</td><td>site of polarized growth</td></tr>
<tr><td>GO:0000790</td><td>HUMAN|HGNC=11594|UniProtKB=Q96SF7,HUMAN|HGNC=14008|UniProtKB=Q9BW11,HUMAN|HGNC=17780|UniProtKB=Q562R1,HUMAN|HGNC=18411|UniProtKB=Q3KNW1,HUMAN|HGNC=14010|UniProtKB=Q8IWI9,HUMAN|HGNC=11364|UniProtKB=P40763,HUMAN|HGNC=3815|UniProtKB=Q12951,HUMAN|HGNC=20508|UniProtKB=Q8IUE6,HUMAN|HGNC=11590|UniProtKB=Q16650,HUMAN|HGNC=5036|UniProtKB=Q14103,HUMAN|HGNC=12879|UniProtKB=P21506,HUMAN|HGNC=7002|UniProtKB=A6NDR6</td><td>nuclear chromatin</td></tr>
<tr><td>GO:0008033</td><td>HUMAN|HGNC=26022|UniProtKB=Q7L0Y3</td><td>tRNA processing</td></tr>
<tr><td>GO:0043951</td><td>HUMAN|HGNC=237|UniProtKB=O43306</td><td>negative regulation of cAMP-mediated signaling</td></tr>
<tr><td>GO:0017137</td><td>HUMAN|HGNC=25694|UniProtKB=Q8TC07,HUMAN|HGNC=4226|UniProtKB=P31150,HUMAN|HGNC=30512|UniProtKB=Q5JSH3,HUMAN|HGNC=4227|UniProtKB=P50395,HUMAN|HGNC=25571|UniProtKB=Q9NVG8</td><td>Rab GTPase binding</td></tr>
<tr><td>GO:0017022</td><td>HUMAN|HGNC=13206|UniProtKB=O60296</td><td>myosin binding</td></tr>
<tr><td>GO:0006066</td><td>HUMAN|HGNC=253|UniProtKB=P11766,HUMAN|HGNC=24552|UniProtKB=Q3LXA3,HUMAN|HGNC=12009|UniProtKB=P60174,HUMAN|HGNC=251|UniProtKB=P00326,HUMAN|HGNC=6050|UniProtKB=P29218,HUMAN|HGNC=255|UniProtKB=P28332</td><td>alcohol metabolic process</td></tr>
<tr><td>GO:0051560</td><td>HUMAN|HGNC=23526|UniProtKB=Q8NE86,HUMAN|HGNC=544|UniProtKB=P08133</td><td>mitochondrial calcium ion homeostasis</td></tr>
<tr><td>GO:0050790</td><td>HUMAN|HGNC=9386|UniProtKB=Q9UGJ0</td><td>regulation of catalytic activity</td></tr>
<tr><td>GO:0000276</td><td>HUMAN|HGNC=14247|UniProtKB=O75964,HUMAN|HGNC=845|UniProtKB=O75947,HUMAN|HGNC=850|UniProtKB=P48047</td><td>mitochondrial proton-transporting ATP synthase complex, coupling factor F(o)</td></tr>
<tr><td>GO:0030182</td><td>HUMAN|HGNC=1504|UniProtKB=P42574,HUMAN|HGNC=51815|UniProtKB=A0A1W2PPM1</td><td>neuron differentiation</td></tr>
<tr><td>GO:0030695</td><td>HUMAN|HGNC=4226|UniProtKB=P31150,HUMAN|HGNC=679|UniProtKB=P52566,HUMAN|HGNC=4227|UniProtKB=P50395,HUMAN|HGNC=678|UniProtKB=P52565</td><td>GTPase regulator activity</td></tr>
<tr><td>GO:0035251</td><td>HUMAN|HGNC=4699|UniProtKB=P46976,HUMAN|HGNC=15663|UniProtKB=Q9NYU2</td><td>UDP-glucosyltransferase activity</td></tr>
</table>

<table>
<tr><td>GO:0001725</td><td>HUMAN|HGNC=2067|UniProtKB=O00151,HUMAN|HGNC=7590|UniProtKB=Q15746,HUMAN|HGNC=13200|UniProtKB=Q15942,HUMAN|HGNC=18142|UniProtKB=Q9Y4D1,HUMAN|HGNC=17468|UniProtKB=Q96HC4,HUMAN|HGNC=22958|UniProtKB=Q9NR12,HUMAN|HGNC=6040|UniProtKB=Q13418</td><td>stress fiber</td></tr>
<tr><td>GO:0043596</td><td>HUMAN|HGNC=26200|UniProtKB=Q9H668</td><td>nuclear replication fork</td></tr>
<tr><td>GO:0044380</td><td>HUMAN|HGNC=6891|UniProtKB=Q15555,HUMAN|HGNC=6890|UniProtKB=Q15691</td><td>protein localization to cytoskeleton</td></tr>
<tr><td>GO:0031098</td><td>HUMAN|HGNC=11408|UniProtKB=Q13043,HUMAN|HGNC=30765|UniProtKB=Q9UKE5</td><td>stress-activated protein kinase signaling cascade</td></tr>
<tr><td>PC00073</td><td>HUMAN|HGNC=5269|UniProtKB=P61604,HUMAN|HGNC=1623|UniProtKB=P50990,HUMAN|HGNC=11655|UniProtKB=P17987,HUMAN|HGNC=1617|UniProtKB=P50991,HUMAN|HGNC=1622|UniProtKB=Q99832,HUMAN|HGNC=1618|UniProtKB=P48643,HUMAN|HGNC=1620|UniProtKB=P40227,HUMAN|HGNC=32153|UniProtKB=A6NM43,HUMAN|HGNC=11579|UniProtKB=O75347,HUMAN|HGNC=1615|UniProtKB=P78371</td><td>chaperonin</td></tr>
<tr><td>GO:0061630</td><td>HUMAN|HGNC=463|UniProtKB=Q9UKV5,HUMAN|HGNC=27146|UniProtKB=Q5TA31,HUMAN|HGNC=16276|UniProtKB=Q9C035,HUMAN|HGNC=9261|UniProtKB=Q13356,HUMAN|HGNC=14452|UniProtKB=Q9NYG5,HUMAN|HGNC=26378|UniProtKB=Q8N7C3,HUMAN|HGNC=25544|UniProtKB=Q9HCI7</td><td>ubiquitin protein ligase activity</td></tr>
<tr><td>GO:0033627</td><td>HUMAN|HGNC=23151|UniProtKB=Q86UX7,HUMAN|HGNC=6156|UniProtKB=P05106,HUMAN|HGNC=11491|UniProtKB=P43405,HUMAN|HGNC=6153|UniProtKB=P05556</td><td>cell adhesion mediated by integrin</td></tr>
<tr><td>GO:0045732</td><td>HUMAN|HGNC=16804|UniProtKB=O00308</td><td>positive regulation of protein catabolic process</td></tr>
<tr><td>GO:0006096</td><td>HUMAN|HGNC=8876|UniProtKB=P17858,HUMAN|HGNC=418|UniProtKB=P09972,HUMAN|HGNC=4458|UniProtKB=P06744,HUMAN|HGNC=4141|UniProtKB=P04406,HUMAN|HGNC=8878|UniProtKB=Q01813,HUMAN|HGNC=8898|UniProtKB=P07205,HUMAN|HGNC=4922|UniProtKB=P19367,HUMAN|HGNC=414|UniProtKB=P04075,HUMAN|HGNC=3350|UniProtKB=P06733,HUMAN|HGNC=12009|UniProtKB=P60174,HUMAN|HGNC=3353|UniProtKB=P09104,HUMAN|HGNC=9021|UniProtKB=P14618,HUMAN|HGNC=3354|UniProtKB=P13929</td><td>glycolytic process</td></tr>
<tr><td>GO:0007052</td><td>HUMAN|HGNC=2712|UniProtKB=Q13561,HUMAN|HGNC=12666|UniProtKB=P55072,HUMAN|HGNC=28959|UniProtKB=Q14008,HUMAN|HGNC=9308|UniProtKB=Q15257</td><td>mitotic spindle organization</td></tr>
<tr><td>GO:0001505</td><td>HUMAN|HGNC=16937|UniProtKB=O75915,HUMAN|HGNC=2689|UniProtKB=P09172</td><td>regulation of neurotransmitter levels</td></tr>
<tr><td>PC00033</td><td>HUMAN|HGNC=26022|UniProtKB=Q7L0Y3,HUMAN|HGNC=17136|UniProtKB=Q8IY81</td><td>RNA methyltransferase</td></tr>
<tr><td>GO:0016684</td><td>HUMAN|HGNC=17169|UniProtKB=Q13162,HUMAN|HGNC=4555|UniProtKB=P22352,HUMAN|HGNC=7218|UniProtKB=P05164,HUMAN|HGNC=9355|UniProtKB=P30044,HUMAN|HGNC=4553|UniProtKB=P07203,HUMAN|HGNC=4823|UniProtKB=P69905,HUMAN|HGNC=4829|UniProtKB=P02042,HUMAN|HGNC=9352|UniProtKB=Q06830,HUMAN|HGNC=4827|UniProtKB=P68871,HUMAN|HGNC=3423|UniProtKB=P11678,HUMAN|HGNC=9353|UniProtKB=P32119,HUMAN|HGNC=4831|UniProtKB=P69891,HUMAN|HGNC=1516|UniProtKB=P04040,HUMAN|HGNC=9354|UniProtKB=P30048</td><td>oxidoreductase activity, acting on peroxide as acceptor</td></tr>
</table>

GO:0006366	HUMAN|HGNC=11957|UniProtKB=Q93074,HUMAN|HGNC=25473|UniProtKB=Q9P2N6,HUMAN|HGNC=7670|UniProtKB=Q9Y6Q9,HUMAN|HGNC=7133|UniProtKB=O14686,HUMAN|HGNC=23708|UniProtKB=Q6ZR52,HUMAN|HGNC=15935|UniProtKB=Q9H4I2,HUMAN|HGNC=29084|UniProtKB=O75123,HUMAN|HGNC=26427|UniProtKB=Q96ND8,HUMAN|HGNC=9866|UniProtKB=P13631,HUMAN|HGNC=51815|UniProtKB=A0A1W2PPM1,HUMAN|HGNC=3815|UniProtKB=Q12951,HUMAN|HGNC=11590|UniProtKB=Q16650,HUMAN|HGNC=14451|UniProtKB=Q9BQK8,HUMAN|HGNC=5048|UniProtKB=Q00839,HUMAN|HGNC=12879|UniProtKB=P21506,HUMAN|HGNC=16272|UniProtKB=Q96JK9,HUMAN|HGNC=7910|UniProtKB=P06748,HUMAN|HGNC=10475|UniProtKB=Q9Y230,HUMAN|HGNC=7002|UniProtKB=A6NDR6,HUMAN|HGNC=33108|UniProtKB=Q6NX45,HUMAN|HGNC=14008|UniProtKB=Q9BW11,HUMAN|HGNC=13061|UniProtKB=Q14587,HUMAN|HGNC=14010|UniProtKB=Q8IWI9,HUMAN|HGNC=11364|UniProtKB=P40763,HUMAN|HGNC=21605|UniProtKB=Q14151,HUMAN|HGNC=6702|UniProtKB=Q32MZ4,HUMAN|HGNC=10474|UniProtKB=Q9Y265,HUMAN|HGNC=18807|UniProtKB=O94983,HUMAN|HGNC=13726|UniProtKB=Q8NEZ4,HUMAN|HGNC=16804|UniProtKB=O00308,HUMAN|HGNC=17801|UniProtKB=Q8NHY6,HUMAN|HGNC=11594|UniProtKB=Q96SF7,HUMAN|HGNC=31797|UniProtKB=P0C7V6,HUMAN|HGNC=30025|UniProtKB=Q5VV67,HUMAN|HGNC=9459|UniProtKB=Q92786,HUMAN|HGNC=6767|UniProtKB=Q15797,HUMAN|HGNC=13109|UniProtKB=P17038,HUMAN|HGNC=29989|UniProtKB=Q86YP4,HUMAN|HGNC=23688|UniProtKB=Q86UU0,HUMAN|HGNC=9885|UniProtKB=P29374,HUMAN|HGNC=6207|UniProtKB=P14923,HUMAN|HGNC=7967|UniProtKB=Q96RI1,HUMAN|HGNC=8647|UniProtKB=Q15365,HUMAN|HGNC=7547|UniProtKB=P10243,HUMAN|HGNC=5044|UniProtKB=P61978,HUMAN|HGNC=10055|UniProtKB=Q8N5U6	transcription by RNA polymerase II
GO:0051346	HUMAN|HGNC=607|UniProtKB=P02654,HUMAN|HGNC=871|UniProtKB=Q9UII2,HUMAN|HGNC=533|UniProtKB=P04083	negative regulation of hydrolase activity
GO:0043280	HUMAN|HGNC=19388|UniProtKB=P49593,HUMAN|HGNC=19986|UniProtKB=P99999	positive regulation of cysteine-type endopeptidase activity involved in apoptotic process
GO:0016627	HUMAN|HGNC=121|UniProtKB=O15254,HUMAN|HGNC=119|UniProtKB=Q15067	oxidoreductase activity, acting on the CH-CH group of donors
GO:0008641	HUMAN|HGNC=12469|UniProtKB=P22314	ubiquitin-like modifier activating enzyme activity
PC00046	HUMAN|HGNC=16937|UniProtKB=O75915	amino acid transporter
GO:0009267	HUMAN|HGNC=6501|UniProtKB=P13473,HUMAN|HGNC=9386|UniProtKB=Q9UGJ0	cellular response to starvation
GO:0009056	HUMAN|HGNC=5171|UniProtKB=P02790,HUMAN|HGNC=7176|UniProtKB=P14780	catabolic process
GO:0044057	HUMAN|HGNC=2479|UniProtKB=O76096,HUMAN|HGNC=15474|UniProtKB=Q9Y566,HUMAN|HGNC=2905|UniProtKB=O14490,HUMAN|HGNC=14294|UniProtKB=Q9BYB0	regulation of system process
GO:0030195	HUMAN|HGNC=6383|UniProtKB=P01042,HUMAN|HGNC=2300|UniProtKB=Q96IY4	negative regulation of blood coagulation

GO:0043235	HUMAN|HGNC=1681|UniProtKB=P16070,HUMAN|HGNC=6156|UniProtKB=P05106,HUMAN|HGNC=4243|UniProtKB=P56159,HUMAN|HGNC=19100|UniProtKB=Q5VWK5,HUMAN|HGNC=6153|UniProtKB=P05556,HUMAN|HGNC=19296|UniProtKB=Q9UF33,HUMAN|HGNC=1628|UniProtKB=P08571,HUMAN|HGNC=4244|UniProtKB=O00451,HUMAN|HGNC=6091|UniProtKB=P06213	receptor complex
GO:0001947	HUMAN|HGNC=14010|UniProtKB=Q8IWI9,HUMAN|HGNC=11590|UniProtKB=Q16650	heart looping
GO:0007163	HUMAN|HGNC=18756|UniProtKB=Q9BYZ6,HUMAN|HGNC=9802|UniProtKB=P15153,HUMAN|HGNC=11148|UniProtKB=Q16658,HUMAN|HGNC=29327|UniProtKB=Q5VZ46,HUMAN|HGNC=9801|UniProtKB=P63000,HUMAN|HGNC=28959|UniProtKB=Q14008,HUMAN|HGNC=14653|UniProtKB=Q9HBI1	establishment or maintenance of cell polarity
PC00079	HUMAN|HGNC=24552|UniProtKB=Q3LXA3	cyclase
PC00150	HUMAN|HGNC=7642|UniProtKB=Q99747,HUMAN|HGNC=28984|UniProtKB=Q12768,HUMAN|HGNC=555|UniProtKB=O43747,HUMAN|HGNC=13487|UniProtKB=Q96QK1,HUMAN|HGNC=8766|UniProtKB=Q8WUM4,HUMAN|HGNC=37094|UniProtKB=P0CG39,HUMAN|HGNC=6752|UniProtKB=P20645,HUMAN|HGNC=8016|UniProtKB=P46459,HUMAN|HGNC=30904|UniProtKB=O60763,HUMAN|HGNC=2973|UniProtKB=O00429,HUMAN|HGNC=18451|UniProtKB=Q8NI22,HUMAN|HGNC=13667|UniProtKB=Q9BXS5,HUMAN|HGNC=3419|UniProtKB=P42566,HUMAN|HGNC=561|UniProtKB=O95782,HUMAN|HGNC=9087|UniProtKB=Q04941,HUMAN|HGNC=554|UniProtKB=Q10567,HUMAN|HGNC=16695|UniProtKB=P51572,HUMAN|HGNC=13206|UniProtKB=O60296,HUMAN|HGNC=20120|UniProtKB=Q17RC7,HUMAN|HGNC=7641|UniProtKB=P54920,HUMAN|HGNC=570|UniProtKB=P53677,HUMAN|HGNC=2974|UniProtKB=P50570,HUMAN|HGNC=20954|UniProtKB=Q9NP79,HUMAN|HGNC=24634|UniProtKB=Q9UBC2,HUMAN|HGNC=17643|UniProtKB=Q9HD26,HUMAN|HGNC=23594|UniProtKB=Q709C8,HUMAN|HGNC=15578|UniProtKB=Q96D71,HUMAN|HGNC=10690|UniProtKB=O95810,HUMAN|HGNC=16820|UniProtKB=Q14162,HUMAN|HGNC=3244|UniProtKB=Q9NZN3,HUMAN|HGNC=33895|UniProtKB=Q6S8J3,HUMAN|HGNC=19176|UniProtKB=Q96DZ9,HUMAN|HGNC=29125|UniProtKB=Q9UQ16,HUMAN|HGNC=6631|UniProtKB=P49257,HUMAN|HGNC=30389|UniProtKB=Q96A65,HUMAN|HGNC=1908|UniProtKB=Q96RL7	membrane traffic protein
GO:0035239	HUMAN|HGNC=12652|UniProtKB=P50552	tube morphogenesis
GO:0010811	HUMAN|HGNC=6616|UniProtKB=P48059	positive regulation of cell-substrate adhesion
PC00110	HUMAN|HGNC=5164|UniProtKB=Q9Y251,HUMAN|HGNC=15527|UniProtKB=Q86Z14	glycosidase
PC00261	HUMAN|HGNC=7670|UniProtKB=Q9Y6Q9,HUMAN|HGNC=18039|UniProtKB=Q9UGL1	histone modifying enzyme
PC00212	HUMAN|HGNC=17213|UniProtKB=Q9BWP8	surfactant
GO:0003697	HUMAN|HGNC=8648|UniProtKB=Q15366,HUMAN|HGNC=8647|UniProtKB=Q15365,HUMAN|HGNC=11317|UniProtKB=Q04837	single-stranded DNA binding
GO:0044087	HUMAN|HGNC=5048|UniProtKB=Q00839	regulation of cellular component biogenesis

GO:0006914	HUMAN|HGNC=18423|UniProtKB=O75140,HUMAN|HGNC=16276|UniProtKB=Q9C035,HUMAN|HGNC=19692|UniProtKB=Q96F24,HUMAN|HGNC=1908|UniProtKB=Q96RL7	autophagy
GO:0032147	HUMAN|HGNC=11408|UniProtKB=Q13043,HUMAN|HGNC=30765|UniProtKB=Q9UKE5,HUMAN|HGNC=28164|UniProtKB=Q96M27	activation of protein kinase activity
GO:0008017	HUMAN|HGNC=1090|UniProtKB=Q03001,HUMAN|HGNC=6325|UniProtKB=O60282,HUMAN|HGNC=6891|UniProtKB=Q15555,HUMAN|HGNC=2973|UniProtKB=O00429,HUMAN|HGNC=6890|UniProtKB=Q15691,HUMAN|HGNC=2974|UniProtKB=P50570,HUMAN|HGNC=6317|UniProtKB=O43896,HUMAN|HGNC=28959|UniProtKB=Q14008,HUMAN|HGNC=29125|UniProtKB=Q9UQ16,HUMAN|HGNC=6835|UniProtKB=P78559	microtubule binding
GO:0006403	HUMAN|HGNC=5048|UniProtKB=Q00839	RNA localization
GO:0008276	HUMAN|HGNC=8728|UniProtKB=P22061	protein methyltransferase activity
GO:0005687	HUMAN|HGNC=20471|UniProtKB=O95777	U4 snRNP
GO:0014032	HUMAN|HGNC=544|UniProtKB=P08133,HUMAN|HGNC=2254|UniProtKB=Q9ULV4	neural crest cell development
PC00234	HUMAN|HGNC=20589|UniProtKB=Q9P0J7,HUMAN|HGNC=12488|UniProtKB=P68036,HUMAN|HGNC=12491|UniProtKB=P61081,HUMAN|HGNC=12469|UniProtKB=P22314,HUMAN|HGNC=30688|UniProtKB=Q86VP6,HUMAN|HGNC=30550|UniProtKB=O60337,HUMAN|HGNC=12476|UniProtKB=P61077,HUMAN|HGNC=2552|UniProtKB=Q13617,HUMAN|HGNC=12475|UniProtKB=P62837,HUMAN|HGNC=16804|UniProtKB=O00308,HUMAN|HGNC=463|UniProtKB=Q9UKV5,HUMAN|HGNC=27146|UniProtKB=Q5TA31,HUMAN|HGNC=12492|UniProtKB=P61088,HUMAN|HGNC=20296|UniProtKB=Q9Y2H6,HUMAN|HGNC=30866|UniProtKB=Q8IX04,HUMAN|HGNC=16276|UniProtKB=Q9C035,HUMAN|HGNC=10899|UniProtKB=P63208,HUMAN|HGNC=14452|UniProtKB=Q9NYG5,HUMAN|HGNC=2551|UniProtKB=Q13616,HUMAN|HGNC=26378|UniProtKB=Q8N7C3,HUMAN|HGNC=12494|UniProtKB=Q13404,HUMAN|HGNC=17598|UniProtKB=Q9Y385	ubiquitin-protein ligase
GO:0051283	HUMAN|HGNC=544|UniProtKB=P08133	negative regulation of sequestering of calcium ion
GO:0034329	HUMAN|HGNC=3757|UniProtKB=O75955,HUMAN|HGNC=10252|UniProtKB=O75116	cell junction assembly
GO:0008156	HUMAN|HGNC=7326|UniProtKB=P20585	negative regulation of DNA replication
GO:0022604	HUMAN|HGNC=12690|UniProtKB=P09327	regulation of cell morphogenesis
GO:0061024	HUMAN|HGNC=25541|UniProtKB=P57088,HUMAN|HGNC=12649|UniProtKB=O95292,HUMAN|HGNC=12648|UniProtKB=Q9P0L0	membrane organization
GO:0008242	HUMAN|HGNC=4248|UniProtKB=Q92820	omega peptidase activity
GO:0048666	HUMAN|HGNC=29943|UniProtKB=Q9NT68	neuron development
GO:0010467	HUMAN|HGNC=8648|UniProtKB=Q15366,HUMAN|HGNC=10898|UniProtKB=Q15477,HUMAN|HGNC=17150|UniProtKB=Q9Y2V2,HUMAN|HGNC=6871|UniProtKB=P28482,HUMAN|HGNC=29190|UniProtKB=Q9UPQ9,HUMAN|HGNC=5036|UniProtKB=Q14103,HUMA	gene expression

	N|HGNC=6876|UniProtKB=Q16539,HUMAN|HGNC=17196|UniProtKB=Q8NG08,HUMAN|HGNC=13506|UniProtKB=Q8TBJ5	
GO:0033365	HUMAN|HGNC=23432|UniProtKB=Q9NXG0	protein localization to organelle
GO:0000165	HUMAN|HGNC=26068|UniProtKB=Q6IAA8,HUMAN|HGNC=8761|UniProtKB=Q9BUL8,HUMAN|HGNC=11408|UniProtKB=Q13043,HUMAN|HGNC=6766|UniProtKB=Q8WXG6,HUMAN|HGNC=30765|UniProtKB=Q9UKE5,HUMAN|HGNC=9673|UniProtKB=Q12913,HUMAN|HGNC=6840|UniProtKB=Q02750,HUMAN|HGNC=6091|UniProtKB=P06213,HUMAN|HGNC=8630|UniProtKB=P30086	MAPK cascade
GO:0032984	HUMAN|HGNC=607|UniProtKB=P02654	protein-containing complex disassembly
GO:0015036	HUMAN|HGNC=4623|UniProtKB=P00390,HUMAN|HGNC=13312|UniProtKB=P78417	disulfide oxidoreductase activity
GO:0034399	HUMAN|HGNC=5048|UniProtKB=Q00839	nuclear periphery
GO:0019901	HUMAN|HGNC=10372|UniProtKB=P05386,HUMAN|HGNC=9391|UniProtKB=P13861,HUMAN|HGNC=18756|UniProtKB=Q9BYZ6,HUMAN|HGNC=1742|UniProtKB=P50851,HUMAN|HGNC=8761|UniProtKB=Q9BUL8,HUMAN|HGNC=9801|UniProtKB=P63000,HUMAN|HGNC=9392|UniProtKB=P31323,HUMAN|HGNC=17780|UniProtKB=Q562R1,HUMAN|HGNC=10690|UniProtKB=O95810,HUMAN|HGNC=9802|UniProtKB=P15153,HUMAN|HGNC=29327|UniProtKB=Q5VZ46,HUMAN|HGNC=20292|UniProtKB=Q9Y376,HUMAN|HGNC=16276|UniProtKB=Q9C035,HUMAN|HGNC=9386|UniProtKB=Q9UGJ0	protein kinase binding
GO:0020037	HUMAN|HGNC=4827|UniProtKB=P68871,HUMAN|HGNC=4831|UniProtKB=P69891,HUMAN|HGNC=1516|UniProtKB=P04040,HUMAN|HGNC=4823|UniProtKB=P69905,HUMAN|HGNC=4829|UniProtKB=P02042	heme binding
GO:0007030	HUMAN|HGNC=8761|UniProtKB=Q9BUL8,HUMAN|HGNC=15912|UniProtKB=Q9UNZ2,HUMAN|HGNC=21354|UniProtKB=Q86XR7,HUMAN|HGNC=24251|UniProtKB=Q9Y3A6,HUMAN|HGNC=6631|UniProtKB=P49257,HUMAN|HGNC=16998|UniProtKB=P49755,HUMAN|HGNC=16996|UniProtKB=Q15363,HUMAN|HGNC=24878|UniProtKB=Q9BVK6	Golgi organization
GO:0048365	HUMAN|HGNC=6111|UniProtKB=Q13576,HUMAN|HGNC=679|UniProtKB=P52566,HUMAN|HGNC=21295|UniProtKB=Q8TE68,HUMAN|HGNC=678|UniProtKB=P52565	Rac GTPase binding
GO:0005227	HUMAN|HGNC=23787|UniProtKB=Q9P1W3	calcium activated cation channel activity
GO:0000380	HUMAN|HGNC=5046|UniProtKB=P52272,HUMAN|HGNC=5048|UniProtKB=Q00839	alternative mRNA splicing, via spliceosome
GO:0033047	HUMAN|HGNC=5048|UniProtKB=Q00839	regulation of mitotic sister chromatid segregation
GO:0042886	HUMAN|HGNC=9093|UniProtKB=P55058	amide transport
GO:0035418	HUMAN|HGNC=1117|UniProtKB=Q9UPA5,HUMAN|HGNC=13406|UniProtKB=Q9Y6V0	protein localization to synapse
GO:0000082	HUMAN|HGNC=20063|UniProtKB=Q86T82,HUMAN|HGNC=533|UniProtKB=P04083,HUMAN|HGNC=9568|UniProtKB=Q06323	G1/S transition of mitotic cell cycle
GO:0030838	HUMAN|HGNC=12652|UniProtKB=P50552	positive regulation of actin filament polymerization

<table>
<tr><td>GO:0030169</td><td>HUMAN|HGNC=2367|UniProtKB=P02741,HUMAN|HGNC=584|UniProtKB=P02743</td><td>low-density lipoprotein particle binding</td></tr>
<tr><td>GO:0045047</td><td>HUMAN|HGNC=26212|UniProtKB=P61009</td><td>protein targeting to ER</td></tr>
<tr><td>GO:0045761</td><td>HUMAN|HGNC=4395|UniProtKB=P19086,HUMAN|HGNC=20040|UniProtKB=Q01518,HUMAN|HGNC=4384|UniProtKB=P63096,HUMAN|HGNC=10608|UniProtKB=P47872,HUMAN|HGNC=4381|UniProtKB=Q14344,HUMAN|HGNC=4387|UniProtKB=P08754,HUMAN|HGNC=4385|UniProtKB=P04899,HUMAN|HGNC=4393|UniProtKB=P11488,HUMAN|HGNC=4390|UniProtKB=P50148,HUMAN|HGNC=237|UniProtKB=O43306</td><td>regulation of adenylate cyclase activity</td></tr>
<tr><td>GO:0051345</td><td>HUMAN|HGNC=15476|UniProtKB=P63167,HUMAN|HGNC=7326|UniProtKB=P20585,HUMAN|HGNC=11317|UniProtKB=Q04837</td><td>positive regulation of hydrolase activity</td></tr>
<tr><td>GO:0050650</td><td>HUMAN|HGNC=24293|UniProtKB=Q70JA7</td><td>chondroitin sulfate proteoglycan biosynthetic process</td></tr>
<tr><td>GO:0009152</td><td>HUMAN|HGNC=626|UniProtKB=P07741,HUMAN|HGNC=794|UniProtKB=P31939,HUMAN|HGNC=5157|UniProtKB=P00492,HUMAN|HGNC=469|UniProtKB=Q01433,HUMAN|HGNC=291|UniProtKB=P30566,HUMAN|HGNC=237|UniProtKB=O43306</td><td>purine ribonucleotide biosynthetic process</td></tr>
<tr><td>GO:0045182</td><td>HUMAN|HGNC=3189|UniProtKB=P68104,HUMAN|HGNC=3300|UniProtKB=P63241,HUMAN|HGNC=3214|UniProtKB=P13639,HUMAN|HGNC=4622|UniProtKB=Q8IYD1,HUMAN|HGNC=1243|UniProtKB=Q07021,HUMAN|HGNC=4621|UniProtKB=P15170,HUMAN|HGNC=3200|UniProtKB=Q5VTE0,HUMAN|HGNC=17970|UniProtKB=Q9BPZ3,HUMAN|HGNC=12420|UniProtKB=P49411</td><td>translation regulator activity</td></tr>
<tr><td>GO:0015630</td><td>HUMAN|HGNC=15848|UniProtKB=Q14141,HUMAN|HGNC=2974|UniProtKB=P50570,HUMAN|HGNC=9164|UniProtKB=Q99719,HUMAN|HGNC=1717|UniProtKB=Q16181,HUMAN|HGNC=29125|UniProtKB=Q9UQ16,HUMAN|HGNC=11579|UniProtKB=O75347,HUMAN|HGNC=7729|UniProtKB=Q15019</td><td>microtubule cytoskeleton</td></tr>
<tr><td>GO:0051258</td><td>HUMAN|HGNC=2973|UniProtKB=O00429,HUMAN|HGNC=2974|UniProtKB=P50570,HUMAN|HGNC=29125|UniProtKB=Q9UQ16</td><td>protein polymerization</td></tr>
<tr><td>GO:0008047</td><td>HUMAN|HGNC=20635|UniProtKB=Q14997,HUMAN|HGNC=9308|UniProtKB=Q15257,HUMAN|HGNC=9568|UniProtKB=Q06323</td><td>enzyme activator activity</td></tr>
<tr><td>GO:0008104</td><td>HUMAN|HGNC=16051|UniProtKB=Q8TEW0,HUMAN|HGNC=1742|UniProtKB=P50851,HUMAN|HGNC=16276|UniProtKB=Q9C035</td><td>protein localization</td></tr>
<tr><td>GO:0030544</td><td>HUMAN|HGNC=10519|UniProtKB=Q9NZJ4,HUMAN|HGNC=5270|UniProtKB=P25685</td><td>Hsp70 protein binding</td></tr>
<tr><td>GO:0050772</td><td>HUMAN|HGNC=3338|UniProtKB=Q14247,HUMAN|HGNC=2695|UniProtKB=Q16643,HUMAN|HGNC=2696|UniProtKB=Q9UJU6,HUMAN|HGNC=4844|UniProtKB=P14317</td><td>positive regulation of axonogenesis</td></tr>
<tr><td>GO:0097159</td><td>HUMAN|HGNC=11834|UniProtKB=P29401,HUMAN|HGNC=9723|UniProtKB=P11216,HUMAN|HGNC=9725|UniProtKB=P06737</td><td>organic cyclic compound binding</td></tr>
<tr><td>GO:0009895</td><td>HUMAN|HGNC=607|UniProtKB=P02654</td><td>negative regulation of catabolic process</td></tr>
<tr><td>GO:0047555</td><td>HUMAN|HGNC=8784|UniProtKB=O76074</td><td>3',5'-cyclic-GMP phosphodiesterase activity</td></tr>
<tr><td>GO:0090559</td><td>HUMAN|HGNC=14685|UniProtKB=Q9Y624</td><td>regulation of membrane permeability</td></tr>
<tr><td>GO:0070972</td><td>HUMAN|HGNC=16695|UniProtKB=P51572</td><td>protein localization to endoplasmic reticulum</td></tr>
<tr><td>GO:0045899</td><td>HUMAN|HGNC=9553|UniProtKB=P62333,HUMAN|HGNC=9552|UniProtKB=P62195,HUMAN|HGNC=9551|UniProtKB=P43686</td><td>positive regulation of RNA polymerase II transcription</td></tr>
</table>

		preinitiation complex assembly
GO:0030307	HUMAN|HGNC=3338|UniProtKB=Q14247,HUMAN|HGNC=2695|UniProtKB=Q16643,HUMAN|HGNC=2696|UniProtKB=Q9UJU6,HUMAN|HGNC=4844|UniProtKB=P14317	positive regulation of cell growth
GO:0005682	HUMAN|HGNC=20471|UniProtKB=O95777,HUMAN|HGNC=5046|UniProtKB=P52272,HUMAN|HGNC=29513|UniProtKB=O60306,HUMAN|HGNC=5048|UniProtKB=Q00839	U5 snRNP
GO:0009142	HUMAN|HGNC=361|UniProtKB=P00568	nucleoside triphosphate biosynthetic process
GO:0005750	HUMAN|HGNC=12582|UniProtKB=P14927,HUMAN|HGNC=12590|UniProtKB=P07919	mitochondrial respiratory chain complex III
GO:0016758	HUMAN|HGNC=24293|UniProtKB=Q70JA7,HUMAN|HGNC=9723|UniProtKB=P11216,HUMAN|HGNC=9725|UniProtKB=P06737	transferase activity, transferring hexosyl groups
GO:0000339	HUMAN|HGNC=13759|UniProtKB=Q7L576	RNA cap binding
GO:0070566	HUMAN|HGNC=8087|UniProtKB=P29728	adenylyltransferase activity
PC00114	HUMAN|HGNC=4684|UniProtKB=P33402,HUMAN|HGNC=14237|UniProtKB=Q96NT3	guanylate cyclase
GO:0007156	HUMAN|HGNC=19194|UniProtKB=Q96RW7,HUMAN|HGNC=29557|UniProtKB=Q0ZGT2	homophilic cell adhesion via plasma membrane adhesion molecules
GO:1990720	HUMAN|HGNC=8898|UniProtKB=P07205	C2 axonemal microtubule
GO:0032813	HUMAN|HGNC=17712|UniProtKB=O15304,HUMAN|HGNC=3157|UniProtKB=Q92838,HUMAN|HGNC=6711|UniProtKB=Q06643	tumor necrosis factor receptor superfamily binding
GO:0002699	HUMAN|HGNC=533|UniProtKB=P04083	positive regulation of immune effector process
GO:0031929	HUMAN|HGNC=18423|UniProtKB=O75140,HUMAN|HGNC=15606|UniProtKB=Q9UHA4	TOR signaling
GO:0097649	HUMAN|HGNC=8898|UniProtKB=P07205	A axonemal microtubule
GO:0008283	HUMAN|HGNC=8601|UniProtKB=Q06141,HUMAN|HGNC=4037|UniProtKB=P06241,HUMAN|HGNC=11364|UniProtKB=P40763,HUMAN|HGNC=24588|UniProtKB=Q86XP6,HUMAN|HGNC=19312|UniProtKB=Q56P03,HUMAN|HGNC=12841|UniProtKB=P07947,HUMAN|HGNC=11283|UniProtKB=P12931,HUMAN|HGNC=7002|UniProtKB=A6NDR6,HUMAN|HGNC=6735|UniProtKB=P07948,HUMAN|HGNC=11766|UniProtKB=P01137	cell population proliferation
GO:0030295	HUMAN|HGNC=10372|UniProtKB=P05386	protein kinase activator activity
GO:0065002	HUMAN|HGNC=5261|UniProtKB=P10809	intracellular protein transmembrane transport
GO:0032509	HUMAN|HGNC=25153|UniProtKB=Q96EY5	endosome transport via multivesicular body sorting pathway
GO:0008608	HUMAN|HGNC=24054|UniProtKB=Q8NG31	attachment of spindle microtubules to kinetochore
GO:0005769	HUMAN|HGNC=9785|UniProtKB=P51148,HUMAN|HGNC=9784|UniProtKB=P61020,HUMAN|HGNC=3244|UniProtKB=Q9NZN3,HUMAN|HGNC=11740|UniProtKB=P02787,HUMAN|HGNC=18263|UniProtKB=Q9UL25	early endosome

PC00057	HUMAN|HGNC=8710|UniProtKB=Q9Y5G1,HUMAN|HGNC=15902|UniProtKB=Q8IXH8,HUMAN|HGNC=3230|UniProtKB=Q9NYQ7,HUMAN|HGNC=3048|UniProtKB=Q02413,HUMAN|HGNC=14674|UniProtKB=Q96QU1,HUMAN|HGNC=14265|UniProtKB=Q86UP0,HUMAN|HGNC=3231|UniProtKB=Q9HCU4,HUMAN|HGNC=23112|UniProtKB=Q8TDW7	cadherin
GO:0016776	HUMAN|HGNC=29035|UniProtKB=O43314,HUMAN|HGNC=9141|UniProtKB=Q15126	phosphotransferase activity, phosphate group as acceptor
GO:0030425	HUMAN|HGNC=15720|UniProtKB=Q13033,HUMAN|HGNC=13206|UniProtKB=O60296,HUMAN|HGNC=11424|UniProtKB=O43815,HUMAN|HGNC=3338|UniProtKB=Q14247,HUMAN|HGNC=2695|UniProtKB=Q16643,HUMAN|HGNC=6317|UniProtKB=O43896,HUMAN|HGNC=2696|UniProtKB=Q9UJU6,HUMAN|HGNC=6835|UniProtKB=P78559,HUMAN|HGNC=4844|UniProtKB=P14317,HUMAN|HGNC=11119|UniProtKB=Q99835	dendrite
GO:0000408	HUMAN|HGNC=20356|UniProtKB=Q9BXV9	EKC/KEOPS complex
GO:0036297	HUMAN|HGNC=23845|UniProtKB=Q8IY92	interstrand cross-link repair
GO:0005319	HUMAN|HGNC=8504|UniProtKB=Q969R2,HUMAN|HGNC=10996|UniProtKB=O14975,HUMAN|HGNC=14637|UniProtKB=Q86UK0,HUMAN|HGNC=603|UniProtKB=P04114	lipid transporter activity
GO:0032508	HUMAN|HGNC=533|UniProtKB=P04083	DNA duplex unwinding
GO:0071214	HUMAN|HGNC=28771|UniProtKB=Q9BVC6	cellular response to abiotic stimulus
GO:0030042	HUMAN|HGNC=15750|UniProtKB=P60981,HUMAN|HGNC=9621|UniProtKB=Q6IBS0,HUMAN|HGNC=12690|UniProtKB=P09327,HUMAN|HGNC=12754|UniProtKB=O75083,HUMAN|HGNC=1874|UniProtKB=P23528,HUMAN|HGNC=243|UniProtKB=P35611,HUMAN|HGNC=1488|UniProtKB=P52907,HUMAN|HGNC=3750|UniProtKB=Q13045,HUMAN|HGNC=1491|UniProtKB=P47756,HUMAN|HGNC=15667|UniProtKB=P58546	actin filament depolymerization
GO:0016595	HUMAN|HGNC=16768|UniProtKB=O60391	glutamate binding
GO:0006906	HUMAN|HGNC=11442|UniProtKB=O15400,HUMAN|HGNC=12644|UniProtKB=Q15836,HUMAN|HGNC=12647|UniProtKB=Q9BV40,HUMAN|HGNC=9788|UniProtKB=P51149	vesicle fusion
GO:0070374	HUMAN|HGNC=1681|UniProtKB=P16070,HUMAN|HGNC=10632|UniProtKB=P13501	positive regulation of ERK1 and ERK2 cascade
GO:1901362	HUMAN|HGNC=2689|UniProtKB=P09172	organic cyclic compound biosynthetic process
GO:0034394	HUMAN|HGNC=17643|UniProtKB=Q9HD26	protein localization to cell surface
GO:0048771	HUMAN|HGNC=533|UniProtKB=P04083	tissue remodeling
GO:0007094	HUMAN|HGNC=12401|UniProtKB=P33981	mitotic spindle assembly checkpoint
GO:0006629	HUMAN|HGNC=612|UniProtKB=P05090	lipid metabolic process
GO:0017048	HUMAN|HGNC=29327|UniProtKB=Q5VZ46	Rho GTPase binding

GO:1901605	HUMAN|HGNC=4433|UniProtKB=P00505,HUMAN|HGNC=4335|UniProtKB=P00367,HUMAN|HGNC=746|UniProtKB=P04424,HUMAN|HGNC=12388|UniProtKB=Q16762,HUMAN|HGNC=1424|UniProtKB=P27708,HUMAN|HGNC=7223|UniProtKB=P25325	alpha-amino acid metabolic process
GO:0009260	HUMAN|HGNC=2520|UniProtKB=Q9NRF8	ribonucleotide biosynthetic process
GO:0031593	HUMAN|HGNC=13919|UniProtKB=P46379,HUMAN|HGNC=12666|UniProtKB=P55072,HUMAN|HGNC=9813|UniProtKB=P54727	polyubiquitin modification-dependent protein binding
GO:0032543	HUMAN|HGNC=1243|UniProtKB=Q07021	mitochondrial translation
GO:0065004	HUMAN|HGNC=4861|UniProtKB=Q9NRZ9	protein-DNA complex assembly
GO:0032088	HUMAN|HGNC=27146|UniProtKB=Q5TA31	negative regulation of NF-kappaB transcription factor activity
GO:0030031	HUMAN|HGNC=1736|UniProtKB=P60953	cell projection assembly
GO:0044743	HUMAN|HGNC=5261|UniProtKB=P10809	protein transmembrane import into intracellular organelle
GO:0006364	HUMAN|HGNC=25758|UniProtKB=Q8TED0,HUMAN|HGNC=23818|UniProtKB=Q14684	rRNA processing
GO:0120036	HUMAN|HGNC=12690|UniProtKB=P09327	plasma membrane bounded cell projection organization
GO:0031210	HUMAN|HGNC=1869|UniProtKB=P11597,HUMAN|HGNC=9093|UniProtKB=P55058	phosphatidylcholine binding
GO:0016298	HUMAN|HGNC=17038|UniProtKB=Q99685	lipase activity
GO:0097190	HUMAN|HGNC=17865|UniProtKB=Q96A26,HUMAN|HGNC=544|UniProtKB=P08133	apoptotic signaling pathway
GO:0051051	HUMAN|HGNC=9855|UniProtKB=P62834,HUMAN|HGNC=607|UniProtKB=P02654,HUMAN|HGNC=533|UniProtKB=P04083,HUMAN|HGNC=9857|UniProtKB=P61224,HUMAN|HGNC=17643|UniProtKB=Q9HD26,HUMAN|HGNC=16937|UniProtKB=O75915	negative regulation of transport
GO:0030687	HUMAN|HGNC=23818|UniProtKB=Q14684	preribosome, large subunit precursor
GO:0060261	HUMAN|HGNC=19985|UniProtKB=P53999	positive regulation of transcription initiation from RNA polymerase II promoter
GO:0019200	HUMAN|HGNC=8876|UniProtKB=P17858,HUMAN|HGNC=4922|UniProtKB=P19367,HUMAN|HGNC=8878|UniProtKB=Q01813	carbohydrate kinase activity
GO:2001235	HUMAN|HGNC=17865|UniProtKB=Q96A26	positive regulation of apoptotic signaling pathway
GO:0032101	HUMAN|HGNC=11491|UniProtKB=P43405,HUMAN|HGNC=3537|UniProtKB=P25116	regulation of response to external stimulus
GO:0019438	HUMAN|HGNC=2689|UniProtKB=P09172	aromatic compound biosynthetic process
PC00122	HUMAN|HGNC=2689|UniProtKB=P09172	hydroxylase
GO:0023057	HUMAN|HGNC=9855|UniProtKB=P62834,HUMAN|HGNC=9857|UniProtKB=P61224	negative regulation of signaling
GO:0008021	HUMAN|HGNC=31332|UniProtKB=Q6IQ22,HUMAN|HGNC=7007|UniProtKB=P61006,HUMAN|HGNC=11442|UniProtKB=O15400,HU	synaptic vesicle

	MAN|HGNC=9164|UniProtKB=Q99719,HUMAN|HGNC=9778|UniProtKB=P20337	
PC00135	HUMAN|HGNC=4458|UniProtKB=P06744,HUMAN|HGNC=12009|UniProtKB=P60174,HUMAN|HGNC=11609|UniProtKB=P24557	isomerase
GO:0007059	HUMAN|HGNC=9112|UniProtKB=Q6P1K2	chromosome segregation
GO:0031965	HUMAN|HGNC=17103|UniProtKB=Q9Y5L0	nuclear membrane
GO:0031397	HUMAN|HGNC=23077|UniProtKB=Q96FW1	negative regulation of protein ubiquitination
GO:2000045	HUMAN|HGNC=533|UniProtKB=P04083,HUMAN|HGNC=9568|UniProtKB=Q06323	regulation of G1/S transition of mitotic cell cycle
GO:0070936	HUMAN|HGNC=463|UniProtKB=Q9UKV5,HUMAN|HGNC=12475|UniProtKB=P62837	protein K48-linked ubiquitination
GO:0015301	HUMAN|HGNC=10991|UniProtKB=P05141,HUMAN|HGNC=10992|UniProtKB=P12236,HUMAN|HGNC=11027|UniProtKB=P02730	anion:anion antiporter activity
GO:0008540	HUMAN|HGNC=9554|UniProtKB=Q99460,HUMAN|HGNC=9553|UniProtKB=P62333,HUMAN|HGNC=9552|UniProtKB=P62195,HUMAN|HGNC=9551|UniProtKB=P43686,HUMAN|HGNC=9559|UniProtKB=Q13200	proteasome regulatory particle, base subcomplex
GO:0035725	HUMAN|HGNC=11056|UniProtKB=P48067,HUMAN|HGNC=800|UniProtKB=P50993,HUMAN|HGNC=806|UniProtKB=P54709,HUMAN|HGNC=10594|UniProtKB=Q01118,HUMAN|HGNC=10582|UniProtKB=Q9Y5Y9	sodium ion transmembrane transport
GO:0099572	HUMAN|HGNC=2905|UniProtKB=O14490	postsynaptic specialization
GO:2001238	HUMAN|HGNC=1050|UniProtKB=P55957	positive regulation of extrinsic apoptotic signaling pathway
GO:0007275	HUMAN|HGNC=15902|UniProtKB=Q8IXH8,HUMAN|HGNC=243|UniProtKB=P35611,HUMAN|HGNC=14265|UniProtKB=Q86UP0,HUMAN|HGNC=6091|UniProtKB=P06213	multicellular organism development
GO:0005764	HUMAN|HGNC=26068|UniProtKB=Q6IAA8,HUMAN|HGNC=17955|UniProtKB=O43504,HUMAN|HGNC=2479|UniProtKB=O76096,HUMAN|HGNC=2528|UniProtKB=P53634,HUMAN|HGNC=913|UniProtKB=P20160,HUMAN|HGNC=9788|UniProtKB=P51149	lysosome
GO:0048709	HUMAN|HGNC=9086|UniProtKB=P60201	oligodendrocyte differentiation
GO:0051642	HUMAN|HGNC=16051|UniProtKB=Q8TEW0,HUMAN|HGNC=14906|UniProtKB=Q8N4C6	centrosome localization
PC00169	HUMAN|HGNC=9866|UniProtKB=P13631,HUMAN|HGNC=7967|UniProtKB=Q96RI1	C4 zinc finger nuclear receptor
GO:0050803	HUMAN|HGNC=2974|UniProtKB=P50570,HUMAN|HGNC=29125|UniProtKB=Q9UQ16	regulation of synapse structure or activity
GO:0007204	HUMAN|HGNC=3537|UniProtKB=P25116,HUMAN|HGNC=6383|UniProtKB=P01042,HUMAN|HGNC=4472|UniProtKB=Q14330,HUMAN|HGNC=3540|UniProtKB=Q96RI0	positive regulation of cytosolic calcium ion concentration
GO:0017069	HUMAN|HGNC=17259|UniProtKB=Q9Y4Z0,HUMAN|HGNC=5048|UniProtKB=Q00839	snRNA binding
GO:0060341	HUMAN|HGNC=11491|UniProtKB=P43405,HUMAN|HGNC=29327|UniProtKB=Q5VZ46,HUMAN|HGNC=10534|UniProtKB=Q9NR31,HUMAN|HGNC=5048|UniProtKB=Q00839	regulation of cellular localization

<table>
<tr><td>GO:0097367</td><td>HUMAN|HGNC=12407|UniProtKB=P68366,HUMAN|HGNC=654|UniProtKB=P61204,HUMAN|HGNC=3778|UniProtKB=P02751,HUMAN|HGNC=9391|UniProtKB=P13861,HUMAN|HGNC=18756|UniProtKB=Q9BYZ6,HUMAN|HGNC=9414|UniProtKB=Q13976,HUMAN|HGNC=9862|UniProtKB=P61225,HUMAN|HGNC=652|UniProtKB=P84077,HUMAN|HGNC=12410|UniProtKB=Q9NY65,HUMAN|HGNC=9380|UniProtKB=P17612,HUMAN|HGNC=20778|UniProtKB=P07437,HUMAN|HGNC=6407|UniProtKB=P01116,HUMAN|HGNC=9392|UniProtKB=P31323,HUMAN|HGNC=20766|UniProtKB=Q71U36,HUMAN|HGNC=667|UniProtKB=P61586,HUMAN|HGNC=4227|UniProtKB=P50395,HUMAN|HGNC=9855|UniProtKB=P62834,HUMAN|HGNC=659|UniProtKB=P62330,HUMAN|HGNC=9840|UniProtKB=P11234,HUMAN|HGNC=9381|UniProtKB=P22694,HUMAN|HGNC=678|UniProtKB=P52565,HUMAN|HGNC=692|UniProtKB=P40616,HUMAN|HGNC=4226|UniProtKB=P31150,HUMAN|HGNC=626|UniProtKB=P07741,HUMAN|HGNC=9801|UniProtKB=P63000,HUMAN|HGNC=669|UniProtKB=P08134,HUMAN|HGNC=9857|UniProtKB=P61224,HUMAN|HGNC=679|UniProtKB=P52566,HUMAN|HGNC=20774|UniProtKB=P04350,HUMAN|HGNC=10447|UniProtKB=P10301,HUMAN|HGNC=11231|UniProtKB=Q8WXF7,HUMAN|HGNC=658|UniProtKB=P84085,HUMAN|HGNC=20771|UniProtKB=P68371,HUMAN|HGNC=655|UniProtKB=P18085,HUMAN|HGNC=24526|UniProtKB=Q6DD88,HUMAN|HGNC=10011|UniProtKB=Q15382,HUMAN|HGNC=9802|UniProtKB=P15153,HUMAN|HGNC=1736|UniProtKB=P60953,HUMAN|HGNC=16257|UniProtKB=Q9H4B7,HUMAN|HGNC=2153|UniProtKB=Q9NQW8,HUMAN|HGNC=9386|UniProtKB=Q9UGJ0,HUMAN|HGNC=4234|UniProtKB=P55040</td><td>carbohydrate derivative binding</td></tr>
<tr><td>GO:0030677</td><td>HUMAN|HGNC=26022|UniProtKB=Q7L0Y3</td><td>ribonuclease P complex</td></tr>
<tr><td>PC00021</td><td>HUMAN|HGNC=3537|UniProtKB=P25116,HUMAN|HGNC=4472|UniProtKB=Q14330,HUMAN|HGNC=3540|UniProtKB=Q96RI0</td><td>G-protein coupled receptor</td></tr>
<tr><td>PC00081</td><td>HUMAN|HGNC=23077|UniProtKB=Q96FW1,HUMAN|HGNC=2528|UniProtKB=P53634,HUMAN|HGNC=1476|UniProtKB=P07384,HUMAN|HGNC=20063|UniProtKB=Q86T82,HUMAN|HGNC=12628|UniProtKB=P45974,HUMAN|HGNC=735|UniProtKB=Q13510,HUMAN|HGNC=12612|UniProtKB=P54578,HUMAN|HGNC=25035|UniProtKB=Q5VVQ6,HUMAN|HGNC=4248|UniProtKB=Q92820,HUMAN|HGNC=9472|UniProtKB=Q99538,HUMAN|HGNC=13485|UniProtKB=Q9BXU7,HUMAN|HGNC=20076|UniProtKB=Q96K76</td><td>cysteine protease</td></tr>
<tr><td>GO:0019783</td><td>HUMAN|HGNC=23077|UniProtKB=Q96FW1</td><td>ubiquitin-like protein-specific protease activity</td></tr>
<tr><td>GO:0016765</td><td>HUMAN|HGNC=4635|UniProtKB=P21266,HUMAN|HGNC=4634|UniProtKB=P28161,HUMAN|HGNC=4632|UniProtKB=P09488,HUMAN|HGNC=4638|UniProtKB=P09211</td><td>transferase activity, transferring alkyl or aryl (other than methyl) groups</td></tr>
<tr><td>GO:0000776</td><td>HUMAN|HGNC=12401|UniProtKB=P33981,HUMAN|HGNC=28959|UniProtKB=Q14008,HUMAN|HGNC=5048|UniProtKB=Q00839</td><td>kinetochore</td></tr>
<tr><td>GO:0033017</td><td>HUMAN|HGNC=813|UniProtKB=Q93084,HUMAN|HGNC=812|UniProtKB=P16615</td><td>sarcoplasmic reticulum membrane</td></tr>
<tr><td>GO:0005737</td><td>HUMAN|HGNC=654|UniProtKB=P61204,HUMAN|HGNC=5046|UniProtKB=P52272,HUMAN|HGNC=9545|UniProtKB=P28062,HUMAN|HGNC=316|UniProtKB=P43652,HUMAN|HGNC=12410|UniProtKB=Q9NY65,HUMAN|HGNC=17905|UniProtKB=Q9Y613,HUMAN|HGNC=22125|UniProtKB=O95522,HUMAN|HGNC=14653|UniProtKB=Q9HBI1,HUMAN|HGNC=18585|UniProtKB=Q96DH6,HUMAN|HGN</td><td>cytoplasm</td></tr>
</table>

C=10494|UniProtKB=P26447,HUMAN|HGNC=27146|UniProtKB=Q
5TA31,HUMAN|HGNC=9536|UniProtKB=O14818,HUMAN|HGNC=1
2468|UniProtKB=P0CG48,HUMAN|HGNC=9846|UniProtKB=P6282
6,HUMAN|HGNC=4312|UniProtKB=P48507,HUMAN|HGNC=9021|
UniProtKB=P14618,HUMAN|HGNC=13420|UniProtKB=Q86TP1,HU
MAN|HGNC=9723|UniProtKB=P11216,HUMAN|HGNC=30535|Uni
ProtKB=Q8WVJ2,HUMAN|HGNC=612|UniProtKB=P05090,HUMAN
|HGNC=399|UniProtKB=P02768,HUMAN|HGNC=9281|UniProtKB=
P62136,HUMAN|HGNC=30646|UniProtKB=Q7KZF4,HUMAN|HGNC
=6528|UniProtKB=P13796,HUMAN|HGNC=6616|UniProtKB=P4805
9,HUMAN|HGNC=9355|UniProtKB=P30044,HUMAN|HGNC=4622|
UniProtKB=Q8IYD1,HUMAN|HGNC=8881|UniProtKB=P07737,HUM
AN|HGNC=13200|UniProtKB=Q15942,HUMAN|HGNC=4621|UniPr
otKB=P15170,HUMAN|HGNC=16219|UniProtKB=Q8TEA8,HUMAN|
HGNC=11226|UniProtKB=Q96JI7,HUMAN|HGNC=369|UniProtKB=
Q9UKA4,HUMAN|HGNC=20774|UniProtKB=P04350,HUMAN|HGN
C=7732|UniProtKB=Q15843,HUMAN|HGNC=12729|UniProtKB=P2
3381,HUMAN|HGNC=9531|UniProtKB=P25787,HUMAN|HGNC=53
5|UniProtKB=P50995,HUMAN|HGNC=6767|UniProtKB=Q15797,H
UMAN|HGNC=4699|UniProtKB=P46976,HUMAN|HGNC=9539|Uni
ProtKB=P49721,HUMAN|HGNC=20908|UniProtKB=Q86YF9,HUMA
N|HGNC=1476|UniProtKB=P07384,HUMAN|HGNC=19825|UniProt
KB=Q6SA08,HUMAN|HGNC=9568|UniProtKB=Q06323,HUMAN|H
GNC=226|UniProtKB=P78563,HUMAN|HGNC=1373|UniProtKB=P0
0918,HUMAN|HGNC=9386|UniProtKB=Q9UGJ0,HUMAN|HGNC=3
297|UniProtKB=P78344,HUMAN|HGNC=19760|UniProtKB=Q96T5
1,HUMAN|HGNC=12407|UniProtKB=P68366,HUMAN|HGNC=9533
|UniProtKB=P25789,HUMAN|HGNC=12022|UniProtKB=P13693,HU
MAN|HGNC=4187|UniProtKB=P02774,HUMAN|HGNC=4926|UniPr
otKB=O15212,HUMAN|HGNC=12733|UniProtKB=Q9Y6W5,HUMA
N|HGNC=11582|UniProtKB=Q15813,HUMAN|HGNC=20778|UniPr
otKB=P07437,HUMAN|HGNC=20766|UniProtKB=Q71U36,HUMAN
|HGNC=26363|UniProtKB=Q86YJ7,HUMAN|HGNC=4227|UniProtK
B=P50395,HUMAN|HGNC=12463|UniProtKB=P0CG47,HUMAN|HG
NC=33749|UniProtKB=Q52MB2,HUMAN|HGNC=711|UniProtKB=P
49407,HUMAN|HGNC=12458|UniProtKB=P62987,HUMAN|HGNC=
26928|UniProtKB=Q9UKY7,HUMAN|HGNC=10823|UniProtKB=O75
368,HUMAN|HGNC=544|UniProtKB=P08133,HUMAN|HGNC=5240
|UniProtKB=P48741,HUMAN|HGNC=11138|UniProtKB=P37840,HU
MAN|HGNC=30205|UniProtKB=Q9H7C9,HUMAN|HGNC=8868|Uni
ProtKB=Q9NQP4,HUMAN|HGNC=708|UniProtKB=O15511,HUMAN
|HGNC=537|UniProtKB=P07355,HUMAN|HGNC=20040|UniProtKB
=Q01518,HUMAN|HGNC=6111|UniProtKB=Q13576,HUMAN|HGN
C=11148|UniProtKB=Q16658,HUMAN|HGNC=9302|UniProtKB=P3
0153,HUMAN|HGNC=1735|UniProtKB=Q16543,HUMAN|HGNC=15
568|UniProtKB=Q9H299,HUMAN|HGNC=29884|UniProtKB=Q8TF4
2,HUMAN|HGNC=11619|UniProtKB=Q15370,HUMAN|HGNC=746|
UniProtKB=P04424,HUMAN|HGNC=1507|UniProtKB=P55212,HUM
AN|HGNC=658|UniProtKB=P84085,HUMAN|HGNC=28235|UniProt
KB=Q96IU4,HUMAN|HGNC=1090|UniProtKB=Q03001,HUMAN|HG
NC=15578|UniProtKB=Q96D71,HUMAN|HGNC=10690|UniProtKB=
O95810,HUMAN|HGNC=17150|UniProtKB=Q9Y2V2,HUMAN|HGN
C=10499|UniProtKB=P06702,HUMAN|HGNC=16391|UniProtKB=Q
9H257,HUMAN|HGNC=16276|UniProtKB=Q9C035,HUMAN|HGNC

=6871|UniProtKB=P28482,HUMAN|HGNC=9308|UniProtKB=Q152
57,HUMAN|HGNC=12363|UniProtKB=P49815,HUMAN|HGNC=164
76|UniProtKB=Q9H0E2,HUMAN|HGNC=9462|UniProtKB=P60891,
HUMAN|HGNC=5044|UniProtKB=P61978,HUMAN|HGNC=4396|U
niProtKB=P62873,HUMAN|HGNC=1745|UniProtKB=O00311,HUMA
N|HGNC=543|UniProtKB=P08758,HUMAN|HGNC=9465|UniProtKB
=P11908,HUMAN|HGNC=2520|UniProtKB=Q9NRF8,HUMAN|HGN
C=20597|UniProtKB=P61960,HUMAN|HGNC=5239|UniProtKB=P1
7066,HUMAN|HGNC=3213|UniProtKB=P26641,HUMAN|HGNC=24
254|UniProtKB=Q96CN7,HUMAN|HGNC=1424|UniProtKB=P27708
,HUMAN|HGNC=18994|UniProtKB=Q8TDC3,HUMAN|HGNC=7377
|UniProtKB=Q9UJ68,HUMAN|HGNC=6876|UniProtKB=Q16539,HU
MAN|HGNC=10432|UniProtKB=P51812,HUMAN|HGNC=1504|Uni
ProtKB=P42574,HUMAN|HGNC=9581|UniProtKB=Q9H939,HUMAN
|HGNC=1516|UniProtKB=P04040,HUMAN|HGNC=18170|UniProtK
B=P30085,HUMAN|HGNC=9530|UniProtKB=P25786,HUMAN|HGN
C=10542|UniProtKB=O95248,HUMAN|HGNC=15687|UniProtKB=O
75563,HUMAN|HGNC=16452|UniProtKB=P0DN86,HUMAN|HGNC
=7910|UniProtKB=P06748,HUMAN|HGNC=21740|UniProtKB=Q6Z
V73,HUMAN|HGNC=27937|UniProtKB=Q6ZVH7,HUMAN|HGNC=1
2690|UniProtKB=P09327,HUMAN|HGNC=2712|UniProtKB=Q1356
1,HUMAN|HGNC=626|UniProtKB=P07741,HUMAN|HGNC=533|Un
iProtKB=P04083,HUMAN|HGNC=30765|UniProtKB=Q9UKE5,HUM
AN|HGNC=6510|UniProtKB=P16949,HUMAN|HGNC=6400|UniProt
KB=Q14974,HUMAN|HGNC=17820|UniProtKB=Q9H0P0,HUMAN|
HGNC=20771|UniProtKB=P68371,HUMAN|HGNC=8903|UniProtKB
=O95336,HUMAN|HGNC=9725|UniProtKB=P06737,HUMAN|HGNC
=6971|UniProtKB=P40926,HUMAN|HGNC=1368|UniProtKB=P0091
5,HUMAN|HGNC=7590|UniProtKB=Q15746,HUMAN|HGNC=10252
|UniProtKB=O75116,HUMAN|HGNC=8647|UniProtKB=Q15365,HU
MAN|HGNC=15878|UniProtKB=Q9NQ75,HUMAN|HGNC=7629|Un
iProtKB=E9PAV3,HUMAN|HGNC=11881|UniProtKB=P62328,HUMA
N|HGNC=1738|UniProtKB=Q9Y5S2,HUMAN|HGNC=21035|UniPro
tKB=Q8N392,HUMAN|HGNC=6401|UniProtKB=Q92973,HUMAN|H
GNC=652|UniProtKB=P84077,HUMAN|HGNC=4162|UniProtKB=P4
1250,HUMAN|HGNC=12469|UniProtKB=P22314,HUMAN|HGNC=2
0731|UniProtKB=Q9HAV0,HUMAN|HGNC=7892|UniProtKB=P0049
1,HUMAN|HGNC=8927|UniProtKB=Q93100,HUMAN|HGNC=3964|
UniProtKB=P01225,HUMAN|HGNC=7900|UniProtKB=P55786,HUM
AN|HGNC=10898|UniProtKB=Q15477,HUMAN|HGNC=13759|UniP
rotKB=Q7L576,HUMAN|HGNC=11408|UniProtKB=Q13043,HUMAN
|HGNC=29351|UniProtKB=Q9C0H5,HUMAN|HGNC=9540|UniProt
KB=P49720,HUMAN|HGNC=6737|UniProtKB=O75608,HUMAN|HG
NC=9354|UniProtKB=P30048,HUMAN|HGNC=659|UniProtKB=P62
330,HUMAN|HGNC=3750|UniProtKB=Q13045,HUMAN|HGNC=178
60|UniProtKB=Q8NC51,HUMAN|HGNC=15667|UniProtKB=P58546
,HUMAN|HGNC=16721|UniProtKB=A6NKQ9,HUMAN|HGNC=8648
|UniProtKB=Q15366,HUMAN|HGNC=4226|UniProtKB=P31150,HU
MAN|HGNC=317|UniProtKB=P02771,HUMAN|HGNC=545|UniProt
KB=P20073,HUMAN|HGNC=4398|UniProtKB=P62879,HUMAN|HG
NC=15567|UniProtKB=Q9UJC5,HUMAN|HGNC=28164|UniProtKB=
Q96M27,HUMAN|HGNC=17970|UniProtKB=Q9BPZ3,HUMAN|HGN
C=655|UniProtKB=P18085,HUMAN|HGNC=17780|UniProtKB=Q56
2R1,HUMAN|HGNC=9535|UniProtKB=P60900,HUMAN|HGNC=485

	1|UniProtKB=P42858,HUMAN|HGNC=12379|UniProtKB=Q15631,HUMAN|HGNC=30140|UniProtKB=A7MCY6,HUMAN|HGNC=30410|UniProtKB=Q9BXA6,HUMAN|HGNC=8728|UniProtKB=P22061,HUMAN|HGNC=18808|UniProtKB=Q9Y6E2,HUMAN|HGNC=16257|UniProtKB=Q9H4B7,HUMAN|HGNC=9645|UniProtKB=Q05209,HUMAN|HGNC=10899|UniProtKB=P63208,HUMAN|HGNC=20347|UniProtKB=Q9H9C1,HUMAN|HGNC=18173|UniProtKB=Q9NZ08,HUMAN|HGNC=9532|UniProtKB=P25788,HUMAN|HGNC=9537|UniProtKB=P20618,HUMAN|HGNC=8955|UniProtKB=P50453	
GO:0044089	HUMAN|HGNC=3757|UniProtKB=O75955,HUMAN|HGNC=8881|UniProtKB=P07737,HUMAN|HGNC=21295|UniProtKB=Q8TE68	positive regulation of cellular component biogenesis
GO:0055114	HUMAN|HGNC=8876|UniProtKB=P17858,HUMAN|HGNC=418|UniProtKB=P09972,HUMAN|HGNC=253|UniProtKB=P11766,HUMAN|HGNC=4458|UniProtKB=P06744,HUMAN|HGNC=4141|UniProtKB=P04406,HUMAN|HGNC=7863|UniProtKB=Q13423,HUMAN|HGNC=2898|UniProtKB=P09622,HUMAN|HGNC=11834|UniProtKB=P29401,HUMAN|HGNC=8878|UniProtKB=Q01813,HUMAN|HGNC=8891|UniProtKB=P52209,HUMAN|HGNC=8903|UniProtKB=O95336,HUMAN|HGNC=8898|UniProtKB=P07205,HUMAN|HGNC=4922|UniProtKB=P19367,HUMAN|HGNC=414|UniProtKB=P04075,HUMAN|HGNC=3350|UniProtKB=P06733,HUMAN|HGNC=12009|UniProtKB=P60174,HUMAN|HGNC=3353|UniProtKB=P09104,HUMAN|HGNC=9021|UniProtKB=P14618,HUMAN|HGNC=251|UniProtKB=P00326,HUMAN|HGNC=3354|UniProtKB=P13929,HUMAN|HGNC=613|UniProtKB=P02649,HUMAN|HGNC=13312|UniProtKB=P78417,HUMAN|HGNC=255|UniProtKB=P28332	oxidation-reduction process
GO:0006301	HUMAN|HGNC=12492|UniProtKB=P61088,HUMAN|HGNC=12494|UniProtKB=Q13404	postreplication repair
GO:0051235	HUMAN|HGNC=17643|UniProtKB=Q9HD26	maintenance of location
GO:0071704	HUMAN|HGNC=343|UniProtKB=P23526	organic substance metabolic process
GO:0050804	HUMAN|HGNC=2905|UniProtKB=O14490	modulation of chemical synaptic transmission
GO:0007411	HUMAN|HGNC=6325|UniProtKB=O60282,HUMAN|HGNC=12652|UniProtKB=P50552,HUMAN|HGNC=18271|UniProtKB=Q8N8S7,HUMAN|HGNC=11085|UniProtKB=O75093,HUMAN|HGNC=19296|UniProtKB=Q9UF33,HUMAN|HGNC=29557|UniProtKB=Q0ZGT2,HUMAN|HGNC=11119|UniProtKB=Q99835	axon guidance
PC00252	HUMAN|HGNC=11594|UniProtKB=Q96SF7,HUMAN|HGNC=14010|UniProtKB=Q8IWI9,HUMAN|HGNC=11590|UniProtKB=Q16650	Rel homology transcription factor
GO:0006753	HUMAN|HGNC=8052|UniProtKB=Q9UKK9	nucleoside phosphate metabolic process
GO:0034314	HUMAN|HGNC=706|UniProtKB=O15145,HUMAN|HGNC=12733|UniProtKB=Q9Y6W5,HUMAN|HGNC=705|UniProtKB=O15144,HUMAN|HGNC=169|UniProtKB=P61160,HUMAN|HGNC=704|UniProtKB=O15143,HUMAN|HGNC=4374|UniProtKB=O60234,HUMAN|HGNC=170|UniProtKB=P61158,HUMAN|HGNC=708|UniProtKB=O15511	Arp2/3 complex-mediated actin nucleation
PC00118	HUMAN|HGNC=4750|UniProtKB=Q99879,HUMAN|HGNC=32060|UniProtKB=Q5TEC6,HUMAN|HGNC=20508|UniProtKB=Q8IUE6,HUMAN|HGNC=4746|UniProtKB=P62807,HUMAN|HGNC=20514|UniProtKB=Q8N257,HUMAN|HGNC=24700|UniProtKB=Q5QNW6,HUMAN|HGNC=4719|UniProtKB=P16401,HUMAN|HGNC=4747|UniPr	histone

<table>
<tr><td></td><td>otKB=P58876,HUMAN|HGNC=13954|UniProtKB=O60814,HUMAN|HGNC=4762|UniProtKB=P57053,HUMAN|HGNC=4716|UniProtKB=P16403,HUMAN|HGNC=4718|UniProtKB=P10412,HUMAN|HGNC=4755|UniProtKB=Q93079,HUMAN|HGNC=4749|UniProtKB=Q99877</td><td></td></tr>
<tr><td>GO:0015085</td><td>HUMAN|HGNC=813|UniProtKB=Q93084,HUMAN|HGNC=812|UniProtKB=P16615</td><td>calcium ion transmembrane transporter activity</td></tr>
<tr><td>GO:0043123</td><td>HUMAN|HGNC=16391|UniProtKB=Q9H257,HUMAN|HGNC=16276|UniProtKB=Q9C035,HUMAN|HGNC=9275|UniProtKB=P35813</td><td>positive regulation of I-kappaB kinase/NF-kappaB signaling</td></tr>
<tr><td>GO:0006040</td><td>HUMAN|HGNC=9959|UniProtKB=P51606,HUMAN|HGNC=24822|UniProtKB=Q9H479</td><td>amino sugar metabolic process</td></tr>
<tr><td>PC00144</td><td>HUMAN|HGNC=3350|UniProtKB=P06733,HUMAN|HGNC=3353|UniProtKB=P09104,HUMAN|HGNC=28477|UniProtKB=Q8NBM8,HUMAN|HGNC=3354|UniProtKB=P13929,HUMAN|HGNC=746|UniProtKB=P04424,HUMAN|HGNC=291|UniProtKB=P30566</td><td>lyase</td></tr>
<tr><td>GO:0043565</td><td>HUMAN|HGNC=21605|UniProtKB=Q14151</td><td>sequence-specific DNA binding</td></tr>
<tr><td>GO:0005770</td><td>HUMAN|HGNC=2479|UniProtKB=O76096,HUMAN|HGNC=20954|UniProtKB=Q9NP79,HUMAN|HGNC=13487|UniProtKB=Q96QK1,HUMAN|HGNC=9788|UniProtKB=P51149</td><td>late endosome</td></tr>
<tr><td>GO:0003341</td><td>HUMAN|HGNC=2949|UniProtKB=Q8TD57,HUMAN|HGNC=25631|UniProtKB=Q96MT7</td><td>cilium movement</td></tr>
<tr><td>GO:0097539</td><td>HUMAN|HGNC=14906|UniProtKB=Q8N4C6</td><td>ciliary transition fiber</td></tr>
<tr><td>GO:0007031</td><td>HUMAN|HGNC=21689|UniProtKB=Q9Y3D6</td><td>peroxisome organization</td></tr>
<tr><td>PC00140</td><td>HUMAN|HGNC=9391|UniProtKB=P13861,HUMAN|HGNC=9392|UniProtKB=P31323,HUMAN|HGNC=9386|UniProtKB=Q9UGJ0,HUMAN|HGNC=9388|UniProtKB=P10644</td><td>kinase modulator</td></tr>
<tr><td>GO:0005347</td><td>HUMAN|HGNC=10991|UniProtKB=P05141,HUMAN|HGNC=10992|UniProtKB=P12236,HUMAN|HGNC=19375|UniProtKB=Q9BV35</td><td>ATP transmembrane transporter activity</td></tr>
<tr><td>GO:0044281</td><td>HUMAN|HGNC=8527|UniProtKB=P55809</td><td>small molecule metabolic process</td></tr>
<tr><td>GO:0005102</td><td>HUMAN|HGNC=5575|UniProtKB=A0A0B4J1V2,HUMAN|HGNC=5645|UniProtKB=A0A0C4DH34,HUMAN|HGNC=11085|UniProtKB=O75093,HUMAN|HGNC=5549|UniProtKB=A0A0C4DH31,HUMAN|HGNC=14637|UniProtKB=Q86UK0,HUMAN|HGNC=5554|UniProtKB=P01743,HUMAN|HGNC=5480|UniProtKB=P01880,HUMAN|HGNC=5659|UniProtKB=A0A0C4DH38,HUMAN|HGNC=5634|UniProtKB=A0A075B7F0,HUMAN|HGNC=12841|UniProtKB=P07947,HUMAN|HGNC=6735|UniProtKB=P07948,HUMAN|HGNC=11075|UniProtKB=O14745,HUMAN|HGNC=38476|UniProtKB=B9A064,HUMAN|HGNC=4037|UniProtKB=P06241,HUMAN|HGNC=18233|UniProtKB=Q8NDB2,HUMAN|HGNC=5861|UniProtKB=A0M8Q6,HUMAN|HGNC=5647|UniProtKB=A0A087WSY4,HUMAN|HGNC=5563|UniProtKB=A0A075B7D0,HUMAN|HGNC=5856|UniProtKB=P0DOY2,HUMAN|HGNC=5606|UniProtKB=P01763,HUMAN|HGNC=15474|UniProtKB=Q9Y566,HUMAN|HGNC=5576|UniProtKB=P01817,HUMAN|HGNC=5528|UniProtKB=P01861,HUMAN|HGNC=5541|UniProtKB=P01871,HUMAN|HGNC=5585|UniProtKB=A0A0C4DH32,HUMAN|HGNC=11119|UniProtKB=Q99835,HUMAN|HGNC=5551|UniProtKB=A0A0C4DH33,HUMAN|HGNC=5526|UniProtKB=P01859,HUMAN|HGNC=5657|UniProtKB=P0DP08,HUMAN|Ensembl=ENSG00000282639|</td><td>signaling receptor binding</td></tr>
</table>

	UniProtKB=A0A0J9YX35,HUMAN|Gene=HV309_HUMAN|UniProtKB=P01782,HUMAN|HGNC=5654|UniProtKB=P01825,HUMAN|HGNC=5553|UniProtKB=A0A0A0MS14,HUMAN|Gene=HV108_HUMAN|UniProtKB=P0DP01,HUMAN|HGNC=5624|UniProtKB=A0A0B4J1X5,HUMAN|HGNC=5620|UniProtKB=P01780,HUMAN|HGNC=5598|UniProtKB=A0A0C4DH35,HUMAN|HGNC=14294|UniProtKB=Q9BYB0,HUMAN|HGNC=603|UniProtKB=P04114,HUMAN|HGNC=5580|UniProtKB=P01762,HUMAN|HGNC=5633|UniProtKB=A0A075B7D8,HUMAN|HGNC=5582|UniProtKB=A0A0B4J1V0,HUMAN|HGNC=5478|UniProtKB=P01876,HUMAN|HGNC=5617|UniProtKB=A0A075B6Q5,HUMAN|HGNC=5594|UniProtKB=P0DP03,HUMAN|HGNC=5655|UniProtKB=A0A0C4DH41,HUMAN|HGNC=5636|UniProtKB=A0A075B7B8,HUMAN|HGNC=5586|UniProtKB=A0A0B4J1V1,HUMAN|HGNC=5581|UniProtKB=P01766,HUMAN|HGNC=5622|UniProtKB=A0A0B4J1Y9,HUMAN|HGNC=5652|UniProtKB=A0A075B6R2,HUMAN|HGNC=5593|UniProtKB=P0DP02,HUMAN|HGNC=5479|UniProtKB=P01877,HUMAN|HGNC=5619|UniProtKB=A0A0C4DH42,HUMAN|HGNC=5596|UniProtKB=P01772,HUMAN|HGNC=13206|UniProtKB=O60296,HUMAN|HGNC=5550|UniProtKB=P23083,HUMAN|HGNC=5604|UniProtKB=A0A0B4J1X8,HUMAN|HGNC=8078|UniProtKB=O95158,HUMAN|HGNC=5525|UniProtKB=P01857,HUMAN|HGNC=5662|UniProtKB=A0A0B4J1U7,HUMAN|HGNC=5601|UniProtKB=A0A0C4DH36,HUMAN|HGNC=5716|UniProtKB=P01834,HUMAN|HGNC=5588|UniProtKB=P01764,HUMAN|HGNC=5857|UniProtKB=P0DOY3,HUMAN|HGNC=5610|UniProtKB=P01767,HUMAN|HGNC=15588|UniProtKB=Q96C24,HUMAN|Ensembl=ENSG00000282150|UniProtKB=A0A0J9YY99,HUMAN|HGNC=5527|UniProtKB=P01860	
GO:0051247	HUMAN|HGNC=6517|UniProtKB=P18428	positive regulation of protein metabolic process
GO:0005885	HUMAN|HGNC=706|UniProtKB=O15145,HUMAN|HGNC=705|UniProtKB=O15144,HUMAN|HGNC=169|UniProtKB=P61160,HUMAN|HGNC=704|UniProtKB=O15143,HUMAN|HGNC=170|UniProtKB=P61158,HUMAN|HGNC=708|UniProtKB=O15511	Arp2/3 protein complex
GO:0006493	HUMAN|HGNC=8127|UniProtKB=O15294	protein O-linked glycosylation
GO:0051252	HUMAN|HGNC=8648|UniProtKB=Q15366,HUMAN|HGNC=8087|UniProtKB=P29728	regulation of RNA metabolic process
GO:0042592	HUMAN|HGNC=533|UniProtKB=P04083	homeostatic process
GO:0006119	HUMAN|HGNC=8898|UniProtKB=P07205,HUMAN|HGNC=2288|UniProtKB=P14406,HUMAN|HGNC=830|UniProtKB=P06576,HUMAN|HGNC=850|UniProtKB=P48047	oxidative phosphorylation
GO:0006397	HUMAN|HGNC=21605|UniProtKB=Q14151	mRNA processing
GO:0032787	HUMAN|HGNC=253|UniProtKB=P11766,HUMAN|HGNC=251|UniProtKB=P00326,HUMAN|HGNC=6984|UniProtKB=P23368,HUMAN|HGNC=255|UniProtKB=P28332,HUMAN|HGNC=18144|UniProtKB=Q8IZU8	monocarboxylic acid metabolic process
GO:0006325	HUMAN|HGNC=5048|UniProtKB=Q00839	chromatin organization
GO:0006486	HUMAN|HGNC=10869|UniProtKB=Q92185	protein glycosylation

<table>
<tr><td>GO:0043168</td><td>HUMAN|HGNC=12407|UniProtKB=P68366,HUMAN|HGNC=654|UniProtKB=P61204,HUMAN|HGNC=9391|UniProtKB=P13861,HUMAN|HGNC=18756|UniProtKB=Q9BYZ6,HUMAN|HGNC=9414|UniProtKB=Q13976,HUMAN|HGNC=9862|UniProtKB=P61225,HUMAN|HGNC=652|UniProtKB=P84077,HUMAN|HGNC=12410|UniProtKB=Q9NY65,HUMAN|HGNC=9380|UniProtKB=P17612,HUMAN|HGNC=11834|UniProtKB=P29401,HUMAN|HGNC=20778|UniProtKB=P07437,HUMAN|HGNC=6407|UniProtKB=P01116,HUMAN|HGNC=9392|UniProtKB=P31323,HUMAN|HGNC=20766|UniProtKB=Q71U36,HUMAN|HGNC=667|UniProtKB=P61586,HUMAN|HGNC=4227|UniProtKB=P50395,HUMAN|HGNC=4624|UniProtKB=P48637,HUMAN|HGNC=9855|UniProtKB=P62834,HUMAN|HGNC=659|UniProtKB=P62330,HUMAN|HGNC=9840|UniProtKB=P11234,HUMAN|HGNC=9723|UniProtKB=P11216,HUMAN|HGNC=9381|UniProtKB=P22694,HUMAN|HGNC=678|UniProtKB=P52565,HUMAN|HGNC=692|UniProtKB=P40616,HUMAN|HGNC=4226|UniProtKB=P31150,HUMAN|HGNC=626|UniProtKB=P07741,HUMAN|HGNC=9801|UniProtKB=P63000,HUMAN|HGNC=669|UniProtKB=P08134,HUMAN|HGNC=9857|UniProtKB=P61224,HUMAN|HGNC=679|UniProtKB=P52566,HUMAN|HGNC=20774|UniProtKB=P04350,HUMAN|HGNC=10447|UniProtKB=P10301,HUMAN|HGNC=11231|UniProtKB=Q8WXF7,HUMAN|HGNC=658|UniProtKB=P84085,HUMAN|HGNC=20771|UniProtKB=P68371,HUMAN|HGNC=9725|UniProtKB=P06737,HUMAN|HGNC=655|UniProtKB=P18085,HUMAN|HGNC=24526|UniProtKB=Q6DD88,HUMAN|HGNC=10011|UniProtKB=Q15382,HUMAN|HGNC=9802|UniProtKB=P15153,HUMAN|HGNC=1736|UniProtKB=P60953,HUMAN|HGNC=16257|UniProtKB=Q9H4B7,HUMAN|HGNC=2153|UniProtKB=Q9NQW8,HUMAN|HGNC=9386|UniProtKB=Q9UGJ0,HUMAN|HGNC=4234|UniProtKB=P55040</td><td>anion binding</td></tr>
<tr><td>GO:0006357</td><td>HUMAN|HGNC=11957|UniProtKB=Q93074,HUMAN|HGNC=23708|UniProtKB=Q6ZR52,HUMAN|HGNC=15935|UniProtKB=Q9H4I2,HUMAN|HGNC=29084|UniProtKB=O75123,HUMAN|HGNC=26427|UniProtKB=Q96ND8,HUMAN|HGNC=51815|UniProtKB=A0A1W2PPM1,HUMAN|HGNC=3815|UniProtKB=Q12951,HUMAN|HGNC=11590|UniProtKB=Q16650,HUMAN|HGNC=10475|UniProtKB=Q9Y230,HUMAN|HGNC=33108|UniProtKB=Q6NX45,HUMAN|HGNC=14008|UniProtKB=Q9BW11,HUMAN|HGNC=13061|UniProtKB=Q14587,HUMAN|HGNC=14010|UniProtKB=Q8IWI9,HUMAN|HGNC=11364|UniProtKB=P40763,HUMAN|HGNC=21605|UniProtKB=Q14151,HUMAN|HGNC=6702|UniProtKB=Q32MZ4,HUMAN|HGNC=10474|UniProtKB=Q9Y265,HUMAN|HGNC=18807|UniProtKB=O94983,HUMAN|HGNC=17801|UniProtKB=Q8NHY6,HUMAN|HGNC=11594|UniProtKB=Q96SF7,HUMAN|HGNC=31797|UniProtKB=P0C7V6,HUMAN|HGNC=9459|UniProtKB=Q92786,HUMAN|HGNC=13109|UniProtKB=P17038,HUMAN|HGNC=9885|UniProtKB=P29374,HUMAN|HGNC=5044|UniProtKB=P61978</td><td>regulation of transcription by RNA polymerase II</td></tr>
<tr><td>GO:0005832</td><td>HUMAN|HGNC=1623|UniProtKB=P50990,HUMAN|HGNC=11655|UniProtKB=P17987,HUMAN|HGNC=1618|UniProtKB=P48643,HUMAN|HGNC=1620|UniProtKB=P40227,HUMAN|HGNC=32153|UniProtKB=A6NM43,HUMAN|HGNC=1615|UniProtKB=P78371</td><td>chaperonin-containing T-complex</td></tr>
<tr><td>GO:0001894</td><td>HUMAN|HGNC=11756|UniProtKB=Q03403,HUMAN|HGNC=11828|UniProtKB=Q9UDY2</td><td>tissue homeostasis</td></tr>
<tr><td>GO:0030055</td><td>HUMAN|HGNC=23151|UniProtKB=Q86UX7</td><td>cell-substrate junction</td></tr>
</table>

<table>
<tr><td>PC00089</td><td>HUMAN|HGNC=7097|UniProtKB=P14174</td><td>decarboxylase</td></tr>
<tr><td>GO:1902414</td><td>HUMAN|HGNC=11828|UniProtKB=Q9UDY2</td><td>protein localization to cell junction</td></tr>
<tr><td>GO:0007212</td><td>HUMAN|HGNC=4390|UniProtKB=P50148</td><td>dopamine receptor signaling pathway</td></tr>
<tr><td>GO:0071375</td><td>HUMAN|HGNC=11364|UniProtKB=P40763</td><td>cellular response to peptide hormone stimulus</td></tr>
<tr><td>GO:0043488</td><td>HUMAN|HGNC=5046|UniProtKB=P52272,HUMAN|HGNC=17150|UniProtKB=Q9Y2V2</td><td>regulation of mRNA stability</td></tr>
<tr><td>GO:0021953</td><td>HUMAN|HGNC=11590|UniProtKB=Q16650</td><td>central nervous system neuron differentiation</td></tr>
<tr><td>GO:0009968</td><td>HUMAN|HGNC=711|UniProtKB=P49407,HUMAN|HGNC=29884|UniProtKB=Q8TF42,HUMAN|HGNC=7178|UniProtKB=Q13201</td><td>negative regulation of signal transduction</td></tr>
<tr><td>PC00022</td><td>HUMAN|HGNC=4226|UniProtKB=P31150,HUMAN|HGNC=679|UniProtKB=P52566,HUMAN|HGNC=17168|UniProtKB=Q9H2M9,HUMAN|HGNC=4227|UniProtKB=P50395,HUMAN|HGNC=678|UniProtKB=P52565</td><td>G-protein modulator</td></tr>
<tr><td>GO:0004708</td><td>HUMAN|HGNC=6840|UniProtKB=Q02750</td><td>MAP kinase kinase activity</td></tr>
<tr><td>GO:0005819</td><td>HUMAN|HGNC=12401|UniProtKB=P33981,HUMAN|HGNC=2092|UniProtKB=Q00610</td><td>spindle</td></tr>
<tr><td>GO:0030334</td><td>HUMAN|HGNC=11881|UniProtKB=P62328</td><td>regulation of cell migration</td></tr>
<tr><td>GO:0005245</td><td>HUMAN|HGNC=1406|UniProtKB=Q9Y698,HUMAN|HGNC=1393|UniProtKB=O60840</td><td>voltage-gated calcium channel activity</td></tr>
<tr><td>PC00167</td><td>HUMAN|HGNC=9414|UniProtKB=Q13976,HUMAN|HGNC=9406|UniProtKB=Q16513,HUMAN|HGNC=12347|UniProtKB=Q9Y4A5,HUMAN|HGNC=9380|UniProtKB=P17612,HUMAN|HGNC=12401|UniProtKB=P33981,HUMAN|HGNC=30765|UniProtKB=Q9UKE5,HUMAN|HGNC=9395|UniProtKB=P05771,HUMAN|HGNC=24224|UniProtKB=Q9NYV4,HUMAN|HGNC=6840|UniProtKB=Q02750,HUMAN|HGNC=18994|UniProtKB=Q8TDC3,HUMAN|HGNC=6876|UniProtKB=Q16539,HUMAN|HGNC=1459|UniProtKB=Q14012,HUMAN|HGNC=11408|UniProtKB=Q13043,HUMAN|HGNC=30410|UniProtKB=Q9BXA6,HUMAN|HGNC=10252|UniProtKB=O75116,HUMAN|HGNC=6871|UniProtKB=P28482,HUMAN|HGNC=19825|UniProtKB=Q6SA08,HUMAN|HGNC=9381|UniProtKB=P22694,HUMAN|HGNC=6040|UniProtKB=Q13418,HUMAN|HGNC=19286|UniProtKB=Q6P3W7,HUMAN|HGNC=1745|UniProtKB=O00311,HUMAN|HGNC=1738|UniProtKB=Q9Y5S2</td><td>non-receptor serine/threonine protein kinase</td></tr>
<tr><td>PC00177</td><td>HUMAN|HGNC=9604|UniProtKB=P23219</td><td>oxygenase</td></tr>
<tr><td>GO:0019005</td><td>HUMAN|HGNC=2552|UniProtKB=Q13617</td><td>SCF ubiquitin ligase complex</td></tr>
<tr><td>GO:0006693</td><td>HUMAN|HGNC=533|UniProtKB=P04083</td><td>prostaglandin metabolic process</td></tr>
<tr><td>GO:0016866</td><td>HUMAN|HGNC=8876|UniProtKB=P17858,HUMAN|HGNC=8905|UniProtKB=P36871,HUMAN|HGNC=9021|UniProtKB=P14618,HUMAN|HGNC=8878|UniProtKB=Q01813</td><td>intramolecular transferase activity</td></tr>
<tr><td>GO:0005576</td><td>HUMAN|HGNC=3658|UniProtKB=Q9UGM5,HUMAN|HGNC=14669|UniProtKB=P81605,HUMAN|HGNC=7539|UniProtKB=Q9NR99</td><td>extracellular region</td></tr>
<tr><td>GO:0043231</td><td>HUMAN|HGNC=18756|UniProtKB=Q9BYZ6,HUMAN|HGNC=9802|UniProtKB=P15153,HUMAN|HGNC=9253|UniProtKB=P62937,HUMAN|HGNC=570|UniProtKB=P53677,HUMAN|HGNC=575|UniProtK</td><td>intracellular membrane-bounded organelle</td></tr>
</table>

	B=Q9Y587,HUMAN|HGNC=9801|UniProtKB=P63000,HUMAN|HGNC=14637|UniProtKB=Q86UK0,HUMAN|HGNC=13667|UniProtKB=Q9BXS5,HUMAN|HGNC=9255|UniProtKB=P23284	
GO:0030856	HUMAN|HGNC=243|UniProtKB=P35611	regulation of epithelial cell differentiation
GO:0004707	HUMAN|HGNC=6871|UniProtKB=P28482,HUMAN|HGNC=6840|UniProtKB=Q02750,HUMAN|HGNC=6876|UniProtKB=Q16539	MAP kinase activity
GO:0005902	HUMAN|HGNC=11148|UniProtKB=Q16658,HUMAN|HGNC=4381|UniProtKB=Q14344	microvillus
GO:0098840	HUMAN|HGNC=6325|UniProtKB=O60282	protein transport along microtubule
GO:0004888	HUMAN|HGNC=4045|UniProtKB=O75084,HUMAN|HGNC=3616|UniProtKB=P12318,HUMAN|HGNC=8823|UniProtKB=P16284,HUMAN|HGNC=11119|UniProtKB=Q99835	transmembrane signaling receptor activity
GO:0051208	HUMAN|HGNC=544|UniProtKB=P08133,HUMAN|HGNC=29884|UniProtKB=Q8TF42	sequestering of calcium ion
PC00196	HUMAN|HGNC=28883|UniProtKB=Q9H2U2,HUMAN|HGNC=8052|UniProtKB=Q9UKK9	pyrophosphatase
GO:0000184	HUMAN|HGNC=10898|UniProtKB=Q15477,HUMAN|HGNC=17196|UniProtKB=Q8NG08	nuclear-transcribed mRNA catabolic process, nonsense-mediated decay
GO:0043130	HUMAN|HGNC=23077|UniProtKB=Q96FW1,HUMAN|HGNC=15912|UniProtKB=Q9UNZ2,HUMAN|HGNC=16476|UniProtKB=Q9H0E2,HUMAN|HGNC=24103|UniProtKB=Q9H1I8,HUMAN|HGNC=9813|UniProtKB=P54727	ubiquitin binding
GO:0098655	HUMAN|HGNC=1406|UniProtKB=Q9Y698,HUMAN|HGNC=15474|UniProtKB=Q9Y566,HUMAN|HGNC=14294|UniProtKB=Q9BYB0	cation transmembrane transport
GO:0009063	HUMAN|HGNC=4433|UniProtKB=P00505,HUMAN|HGNC=4335|UniProtKB=P00367	cellular amino acid catabolic process
GO:0008064	HUMAN|HGNC=12754|UniProtKB=O75083	regulation of actin polymerization or depolymerization
GO:0098798	HUMAN|HGNC=4801|UniProtKB=P40939,HUMAN|HGNC=26022|UniProtKB=Q7L0Y3	mitochondrial protein complex
GO:0030864	HUMAN|HGNC=3338|UniProtKB=Q14247,HUMAN|HGNC=2695|UniProtKB=Q16643,HUMAN|HGNC=12754|UniProtKB=O75083,HUMAN|HGNC=2696|UniProtKB=Q9UJU6,HUMAN|HGNC=4844|UniProtKB=P14317,HUMAN|HGNC=18304|UniProtKB=Q14019	cortical actin cytoskeleton
GO:0045840	HUMAN|HGNC=15713|UniProtKB=Q9UJX4,HUMAN|HGNC=9847|UniProtKB=P43487,HUMAN|HGNC=5048|UniProtKB=Q00839,HUMAN|HGNC=14452|UniProtKB=Q9NYG5	positive regulation of mitotic nuclear division
PC00074	HUMAN|HGNC=9240|UniProtKB=P02775,HUMAN|HGNC=8862|UniProtKB=P10720,HUMAN|HGNC=8861|UniProtKB=P02776	chemokine
GO:0002429	HUMAN|HGNC=2444|UniProtKB=P41240	immune response-activating cell surface receptor signaling pathway
GO:0030137	HUMAN|HGNC=16998|UniProtKB=P49755	COPI-coated vesicle
GO:0010038	HUMAN|HGNC=2315|UniProtKB=Q96FN4,HUMAN|HGNC=11510|UniProtKB=Q8N9I0,HUMAN|HGNC=11512|UniProtKB=Q9H2B2	response to metal ion
GO:0046907	HUMAN|HGNC=25951|UniProtKB=Q6ULP2,HUMAN|HGNC=830|UniProtKB=P06576,HUMAN|HGNC=850|UniProtKB=P48047	intracellular transport
PC00227	HUMAN|HGNC=6401|UniProtKB=Q92973,HUMAN|HGNC=2431|UniProtKB=P55060,HUMAN|HGNC=8062|UniProtKB=P49790,HUMA	transporter

<table>
<tr><td></td><td>N|HGNC=26801|UniProtKB=Q8N8Q8,HUMAN|HGNC=17103|UniProtKB=Q9Y5L0,HUMAN|HGNC=9852|UniProtKB=O95373,HUMAN|HGNC=30052|UniProtKB=Q8TEM1,HUMAN|HGNC=6396|UniProtKB=O00505,HUMAN|HGNC=568|UniProtKB=O14617,HUMAN|HGNC=6400|UniProtKB=Q14974</td><td></td></tr>
<tr><td>GO:0009790</td><td>HUMAN|HGNC=7002|UniProtKB=A6NDR6,HUMAN|HGNC=30377|UniProtKB=Q14160</td><td>embryo development</td></tr>
<tr><td>GO:0031331</td><td>HUMAN|HGNC=29190|UniProtKB=Q9UPQ9</td><td>positive regulation of cellular catabolic process</td></tr>
<tr><td>GO:0098797</td><td>HUMAN|HGNC=6156|UniProtKB=P05106,HUMAN|HGNC=19100|UniProtKB=Q5VWK5,HUMAN|HGNC=6153|UniProtKB=P05556,HUMAN|HGNC=4387|UniProtKB=P08754,HUMAN|HGNC=20731|UniProtKB=Q9HAV0,HUMAN|HGNC=3338|UniProtKB=Q14247,HUMAN|HGNC=15902|UniProtKB=Q8IXH8,HUMAN|HGNC=14265|UniProtKB=Q86UP0,HUMAN|HGNC=11168|UniProtKB=Q13884,HUMAN|HGNC=19747|UniProtKB=Q86UK5,HUMAN|HGNC=806|UniProtKB=P54709,HUMAN|HGNC=10594|UniProtKB=Q01118,HUMAN|HGNC=4393|UniProtKB=P11488,HUMAN|HGNC=3419|UniProtKB=P42566,HUMAN|HGNC=10582|UniProtKB=Q9Y5Y9,HUMAN|HGNC=561|UniProtKB=O95782,HUMAN|HGNC=6091|UniProtKB=P06213,HUMAN|HGNC=4395|UniProtKB=P19086,HUMAN|HGNC=3757|UniProtKB=O75955,HUMAN|HGNC=4384|UniProtKB=P63096,HUMAN|HGNC=4398|UniProtKB=P62879,HUMAN|HGNC=24634|UniProtKB=Q9UBC2,HUMAN|HGNC=4385|UniProtKB=P04899,HUMAN|HGNC=4390|UniProtKB=P50148,HUMAN|HGNC=4381|UniProtKB=Q14344,HUMAN|HGNC=6207|UniProtKB=P14923,HUMAN|HGNC=4396|UniProtKB=P62873</td><td>plasma membrane protein complex</td></tr>
<tr><td>GO:0001848</td><td>HUMAN|HGNC=2367|UniProtKB=P02741,HUMAN|HGNC=584|UniProtKB=P02743</td><td>complement binding</td></tr>
<tr><td>GO:0005923</td><td>HUMAN|HGNC=14685|UniProtKB=Q9Y624,HUMAN|HGNC=2047|UniProtKB=O00501,HUMAN|HGNC=11828|UniProtKB=Q9UDY2</td><td>bicellular tight junction</td></tr>
<tr><td>GO:0046474</td><td>HUMAN|HGNC=30244|UniProtKB=Q6P1A2</td><td>glycerophospholipid biosynthetic process</td></tr>
<tr><td>GO:0005635</td><td>HUMAN|HGNC=18587|UniProtKB=O94901,HUMAN|HGNC=2431|UniProtKB=P55060,HUMAN|HGNC=9852|UniProtKB=O95373</td><td>nuclear envelope</td></tr>
<tr><td>GO:0030316</td><td>HUMAN|HGNC=29884|UniProtKB=Q8TF42,HUMAN|HGNC=11283|UniProtKB=P12931</td><td>osteoclast differentiation</td></tr>
<tr><td>GO:0003684</td><td>HUMAN|HGNC=2717|UniProtKB=Q16531</td><td>damaged DNA binding</td></tr>
<tr><td>GO:0140326</td><td>HUMAN|HGNC=13531|UniProtKB=Q9Y2Q0</td><td>ATPase-coupled intramembrane lipid transporter activity</td></tr>
<tr><td>GO:0019208</td><td>HUMAN|HGNC=7618|UniProtKB=O14974</td><td>phosphatase regulator activity</td></tr>
<tr><td>PC00184</td><td>HUMAN|HGNC=9295|UniProtKB=Q15435,HUMAN|HGNC=1173|UniProtKB=Q5H9R7,HUMAN|HGNC=7618|UniProtKB=O14974,HUMAN|HGNC=9302|UniProtKB=P30153,HUMAN|HGNC=14871|UniProtKB=Q96A00,HUMAN|HGNC=14952|UniProtKB=Q8TAE6</td><td>phosphatase modulator</td></tr>
<tr><td>GO:0030278</td><td>HUMAN|HGNC=3153|UniProtKB=Q16610</td><td>regulation of ossification</td></tr>
<tr><td>GO:0016079</td><td>HUMAN|HGNC=9855|UniProtKB=P62834,HUMAN|HGNC=7641|UniProtKB=P54920,HUMAN|HGNC=11510|UniProtKB=Q8N9I0,HUMAN|HGNC=11131|UniProtKB=O00161,HUMAN|HGNC=9857|UniProtKB=P61224,HUMAN|HGNC=8515|UniProtKB=Q9HC10</td><td>synaptic vesicle exocytosis</td></tr>
</table>

GO:0044282	HUMAN|HGNC=8876|UniProtKB=P17858,HUMAN|HGNC=253|UniProtKB=P11766,HUMAN|HGNC=4116|UniProtKB=Q14376,HUMAN|HGNC=24552|UniProtKB=Q3LXA3,HUMAN|HGNC=12009|UniProtKB=P60174,HUMAN|HGNC=8878|UniProtKB=Q01813,HUMAN|HGNC=251|UniProtKB=P00326,HUMAN|HGNC=6050|UniProtKB=P29218,HUMAN|HGNC=613|UniProtKB=P02649,HUMAN|HGNC=255|UniProtKB=P28332	small molecule catabolic process
GO:0009057	HUMAN|HGNC=9723|UniProtKB=P11216,HUMAN|HGNC=25153|UniProtKB=Q96EY5,HUMAN|HGNC=9725|UniProtKB=P06737	macromolecule catabolic process
GO:0065003	HUMAN|HGNC=23151|UniProtKB=Q86UX7,HUMAN|HGNC=24526|UniProtKB=Q6DD88,HUMAN|HGNC=30688|UniProtKB=Q86VP6,HUMAN|HGNC=10534|UniProtKB=Q9NR31,HUMAN|HGNC=11231|UniProtKB=Q8WXF7,HUMAN|HGNC=11343|UniProtKB=P50502	protein-containing complex assembly
GO:0006639	HUMAN|HGNC=2850|UniProtKB=Q9Y6T7	acylglycerol metabolic process
GO:0061041	HUMAN|HGNC=11491|UniProtKB=P43405	regulation of wound healing
GO:0043086	HUMAN|HGNC=237|UniProtKB=O43306	negative regulation of catalytic activity
GO:0050852	HUMAN|HGNC=6171|UniProtKB=Q08881,HUMAN|HGNC=4036|UniProtKB=O15117,HUMAN|HGNC=4037|UniProtKB=P06241,HUMAN|HGNC=16961|UniProtKB=Q15762	T cell receptor signaling pathway
GO:0042063	HUMAN|HGNC=533|UniProtKB=P04083	gliogenesis
GO:0045765	HUMAN|HGNC=7178|UniProtKB=Q13201	regulation of angiogenesis
GO:0016575	HUMAN|HGNC=9349|UniProtKB=Q9NQX1	histone deacetylation
PC00216	HUMAN|HGNC=4432|UniProtKB=P17174,HUMAN|HGNC=4242|UniProtKB=O94808,HUMAN|HGNC=4433|UniProtKB=P00505	transaminase
GO:0030479	HUMAN|HGNC=15750|UniProtKB=P60981,HUMAN|HGNC=1874|UniProtKB=P23528,HUMAN|HGNC=169|UniProtKB=P61160,HUMAN|HGNC=1488|UniProtKB=P52907,HUMAN|HGNC=4374|UniProtKB=O60234,HUMAN|HGNC=170|UniProtKB=P61158	actin cortical patch
GO:0016070	HUMAN|HGNC=8648|UniProtKB=Q15366	RNA metabolic process
GO:0051962	HUMAN|HGNC=2479|UniProtKB=O76096	positive regulation of nervous system development
GO:0005774	HUMAN|HGNC=7641|UniProtKB=P54920,HUMAN|HGNC=851|UniProtKB=P38606	vacuolar membrane
GO:0005544	HUMAN|HGNC=2315|UniProtKB=Q96FN4,HUMAN|HGNC=11510|UniProtKB=Q8N9I0,HUMAN|HGNC=544|UniProtKB=P08133,HUMAN|HGNC=533|UniProtKB=P04083,HUMAN|HGNC=11512|UniProtKB=Q9H2B2	calcium-dependent phospholipid binding
GO:0036064	HUMAN|HGNC=30391|UniProtKB=Q9UG01,HUMAN|HGNC=26555|UniProtKB=Q3SY00,HUMAN|HGNC=20908|UniProtKB=Q86YF9	ciliary basal body
GO:0030199	HUMAN|HGNC=3011|UniProtKB=Q07507,HUMAN|HGNC=2209|UniProtKB=P20908,HUMAN|HGNC=1546|UniProtKB=P50454	collagen fibril organization
GO:0072659	HUMAN|HGNC=31332|UniProtKB=Q6IQ22,HUMAN|HGNC=7007|UniProtKB=P61006,HUMAN|HGNC=3757|UniProtKB=O75955,HUMAN|HGNC=4036|UniProtKB=O15117,HUMAN|HGNC=30032|UniProtKB=Q6VY07,HUMAN|HGNC=9778|UniProtKB=P20337,HUMAN	protein localization to plasma membrane

<table>
<tr><td></td><td>|HGNC=3244|UniProtKB=Q9NZN3,HUMAN|HGNC=11075|UniProtKB=O14745,HUMAN|HGNC=492|UniProtKB=P16157</td><td></td></tr>
<tr><td>GO:0000502</td><td>HUMAN|HGNC=9539|UniProtKB=P49721,HUMAN|HGNC=9545|UniProtKB=P28062,HUMAN|HGNC=9540|UniProtKB=P49720,HUMAN|HGNC=9565|UniProtKB=P51665,HUMAN|HGNC=9537|UniProtKB=P20618</td><td>proteasome complex</td></tr>
<tr><td>GO:0005741</td><td>HUMAN|HGNC=992|UniProtKB=Q07817,HUMAN|HGNC=12669|UniProtKB=P21796,HUMAN|HGNC=12674|UniProtKB=Q9Y277,HUMAN|HGNC=12672|UniProtKB=P45880</td><td>mitochondrial outer membrane</td></tr>
<tr><td>GO:0034204</td><td>HUMAN|HGNC=30220|UniProtKB=Q96AA3</td><td>lipid translocation</td></tr>
<tr><td>GO:0046456</td><td>HUMAN|HGNC=533|UniProtKB=P04083</td><td>icosanoid biosynthetic process</td></tr>
<tr><td>GO:0098590</td><td>HUMAN|HGNC=2949|UniProtKB=Q8TD57,HUMAN|HGNC=7007|UniProtKB=P61006,HUMAN|HGNC=26555|UniProtKB=Q3SY00,HUMAN|HGNC=12733|UniProtKB=Q9Y6W5,HUMAN|HGNC=14653|UniProtKB=Q9HBI1,HUMAN|HGNC=667|UniProtKB=P61586,HUMAN|HGNC=11320|UniProtKB=Q8IZP0,HUMAN|HGNC=492|UniProtKB=P16157,HUMAN|HGNC=14313|UniProtKB=Q8WYA0,HUMAN|HGNC=19296|UniProtKB=Q9UF33,HUMAN|HGNC=14906|UniProtKB=Q8N4C6,HUMAN|HGNC=29557|UniProtKB=Q0ZGT2,HUMAN|HGNC=10594|UniProtKB=Q01118,HUMAN|HGNC=11138|UniProtKB=P37840,HUMAN|HGNC=10582|UniProtKB=Q9Y5Y9,HUMAN|HGNC=11512|UniProtKB=Q9H2B2,HUMAN|HGNC=6091|UniProtKB=P06213,HUMAN|HGNC=29262|UniProtKB=Q9P2H3,HUMAN|HGNC=1117|UniProtKB=Q9UPA5,HUMAN|HGNC=13206|UniProtKB=O60296,HUMAN|HGNC=11424|UniProtKB=O43815,HUMAN|HGNC=199|UniProtKB=O43506,HUMAN|HGNC=7641|UniProtKB=P54920,HUMAN|HGNC=12690|UniProtKB=P09327,HUMAN|HGNC=9801|UniProtKB=P63000,HUMAN|HGNC=6835|UniProtKB=P78559,HUMAN|HGNC=6510|UniProtKB=P16949,HUMAN|HGNC=13406|UniProtKB=Q9Y6V0,HUMAN|HGNC=11119|UniProtKB=Q99835,HUMAN|HGNC=17060|UniProtKB=Q92845,HUMAN|HGNC=8898|UniProtKB=P07205,HUMAN|HGNC=30391|UniProtKB=Q9UG01,HUMAN|HGNC=17780|UniProtKB=Q562R1,HUMAN|HGNC=29943|UniProtKB=Q9NT68,HUMAN|HGNC=15720|UniProtKB=Q13033,HUMAN|HGNC=20908|UniProtKB=Q86YF9,HUMAN|HGNC=1736|UniProtKB=P60953,HUMAN|HGNC=2952|UniProtKB=Q96JB1,HUMAN|HGNC=6317|UniProtKB=O43896,HUMAN|HGNC=7590|UniProtKB=Q15746,HUMAN|HGNC=11510|UniProtKB=Q8N9I0,HUMAN|HGNC=26664|UniProtKB=Q8N841</td><td>plasma membrane region</td></tr>
<tr><td>GO:0017119</td><td>HUMAN|HGNC=18621|UniProtKB=Q9Y2V7</td><td>Golgi transport complex</td></tr>
<tr><td>GO:0008219</td><td>HUMAN|HGNC=5141|UniProtKB=P00738,HUMAN|HGNC=5156|UniProtKB=P00739</td><td>cell death</td></tr>
<tr><td>GO:0015101</td><td>HUMAN|HGNC=17292|UniProtKB=Q8IWA5</td><td>organic cation transmembrane transporter activity</td></tr>
<tr><td>GO:0000054</td><td>HUMAN|HGNC=9846|UniProtKB=P62826,HUMAN|HGNC=7910|UniProtKB=P06748</td><td>ribosomal subunit export from nucleus</td></tr>
<tr><td>GO:0043409</td><td>HUMAN|HGNC=8630|UniProtKB=P30086</td><td>negative regulation of MAPK cascade</td></tr>
<tr><td>GO:0048608</td><td>HUMAN|HGNC=533|UniProtKB=P04083</td><td>reproductive structure development</td></tr>
</table>

GO:0097435	HUMAN|HGNC=3602|UniProtKB=Q9UBX5,HUMAN|HGNC=15855|UniProtKB=Q9NPY3	supramolecular fiber organization
GO:0000145	HUMAN|HGNC=20120|UniProtKB=Q17RC7,HUMAN|HGNC=30389|UniProtKB=Q96A65	exocyst
GO:0008066	HUMAN|HGNC=16768|UniProtKB=O60391	glutamate receptor activity
GO:0045087	HUMAN|HGNC=5575|UniProtKB=A0A0B4J1V2,HUMAN|HGNC=5645|UniProtKB=A0A0C4DH34,HUMAN|HGNC=5549|UniProtKB=A0A0C4DH31,HUMAN|HGNC=11092|UniProtKB=P03973,HUMAN|HGNC=5554|UniProtKB=P01743,HUMAN|HGNC=5480|UniProtKB=P01880,HUMAN|HGNC=5659|UniProtKB=A0A0C4DH38,HUMAN|HGNC=5634|UniProtKB=A0A075B7F0,HUMAN|HGNC=12841|UniProtKB=P07947,HUMAN|HGNC=11283|UniProtKB=P12931,HUMAN|HGNC=6735|UniProtKB=P07948,HUMAN|HGNC=27146|UniProtKB=Q5TA31,HUMAN|HGNC=38476|UniProtKB=B9A064,HUMAN|HGNC=4037|UniProtKB=P06241,HUMAN|HGNC=1472|UniProtKB=P49913,HUMAN|HGNC=5861|UniProtKB=A0M8Q6,HUMAN|HGNC=5647|UniProtKB=A0A087WSY4,HUMAN|HGNC=5563|UniProtKB=A0A075B7D0,HUMAN|HGNC=5856|UniProtKB=P0DOY2,HUMAN|HGNC=2367|UniProtKB=P02741,HUMAN|HGNC=5606|UniProtKB=P01763,HUMAN|HGNC=5576|UniProtKB=P01817,HUMAN|HGNC=5528|UniProtKB=P01861,HUMAN|HGNC=5541|UniProtKB=P01871,HUMAN|HGNC=5585|UniProtKB=A0A0C4DH32,HUMAN|HGNC=5551|UniProtKB=A0A0C4DH33,HUMAN|HGNC=5526|UniProtKB=P01859,HUMAN|HGNC=5657|UniProtKB=P0DP08,HUMAN|Ensembl=ENSG00000282639|UniProtKB=A0A0J9YX35,HUMAN|Gene=HV309_HUMAN|UniProtKB=P01782,HUMAN|HGNC=5654|UniProtKB=P01825,HUMAN|HGNC=5553|UniProtKB=A0A0A0MS14,HUMAN|HGNC=7183|UniProtKB=P41218,HUMAN|Gene=HV108_HUMAN|UniProtKB=P0DP01,HUMAN|HGNC=5624|UniProtKB=A0A0B4J1X5,HUMAN|HGNC=5620|UniProtKB=P01780,HUMAN|HGNC=5598|UniProtKB=A0A0C4DH35,HUMAN|HGNC=5580|UniProtKB=P01762,HUMAN|HGNC=5633|UniProtKB=A0A075B7D8,HUMAN|HGNC=5582|UniProtKB=A0A0B4J1V0,HUMAN|HGNC=5478|UniProtKB=P01876,HUMAN|HGNC=5617|UniProtKB=A0A075B6Q5,HUMAN|HGNC=5594|UniProtKB=P0DP03,HUMAN|HGNC=5655|UniProtKB=A0A0C4DH41,HUMAN|HGNC=5636|UniProtKB=A0A075B7B8,HUMAN|HGNC=5586|UniProtKB=A0A0B4J1V1,HUMAN|HGNC=5581|UniProtKB=P01766,HUMAN|HGNC=5622|UniProtKB=A0A0B4J1Y9,HUMAN|HGNC=5652|UniProtKB=A0A075B6R2,HUMAN|HGNC=5593|UniProtKB=P0DP02,HUMAN|HGNC=6517|UniProtKB=P18428,HUMAN|HGNC=10046|UniProtKB=P12724,HUMAN|HGNC=5479|UniProtKB=P01877,HUMAN|HGNC=5619|UniProtKB=A0A0C4DH42,HUMAN|HGNC=5596|UniProtKB=P01772,HUMAN|HGNC=5550|UniProtKB=P23083,HUMAN|HGNC=5604|UniProtKB=A0A0B4J1X8,HUMAN|HGNC=5525|UniProtKB=P01857,HUMAN|HGNC=2761|UniProtKB=P59665,HUMAN|HGNC=10632|UniProtKB=P13501,HUMAN|HGNC=5662|UniProtKB=A0A0B4J1U7,HUMAN|HGNC=16961|UniProtKB=Q15762,HUMAN|HGNC=5601|UniProtKB=A0A0C4DH36,HUMAN|HGNC=5716|UniProtKB=P01834,HUMAN|HGNC=5588|UniProtKB=P01764,HUMAN|HGNC=5857|UniProtKB=P0DOY3,HUMAN|HGNC=5610|UniProtKB=P01767,HUMAN|Ensembl=ENSG00000282150|UniProtKB=A0A0J9YY99,HUMAN|HGNC=5527|UniProtKB=P01860,HU	innate immune response

	MAN|HGNC=584|UniProtKB=P02743,HUMAN|HGNC=10045|UniProtKB=P10153	
GO:0043113	HUMAN|HGNC=30377|UniProtKB=Q14160	receptor clustering
GO:0006886	HUMAN|HGNC=654|UniProtKB=P61204,HUMAN|HGNC=652|UniProtKB=P84077,HUMAN|HGNC=9784|UniProtKB=P61020,HUMAN|HGNC=14902|UniProtKB=Q9NRW1,HUMAN|HGNC=19983|UniProtKB=Q86YS6,HUMAN|HGNC=12666|UniProtKB=P55072,HUMAN|HGNC=13487|UniProtKB=Q96QK1,HUMAN|HGNC=16524|UniProtKB=P61106,HUMAN|HGNC=20726|UniProtKB=Q8WVM8,HUMAN|HGNC=11445|UniProtKB=Q15833,HUMAN|HGNC=9758|UniProtKB=P62820,HUMAN|HGNC=16998|UniProtKB=P49755,HUMAN|HGNC=659|UniProtKB=P62330,HUMAN|HGNC=2232|UniProtKB=P35606,HUMAN|HGNC=18263|UniProtKB=Q9UL25,HUMAN|HGNC=692|UniProtKB=P40616,HUMAN|HGNC=9785|UniProtKB=P51148,HUMAN|HGNC=2230|UniProtKB=P53621,HUMAN|HGNC=7641|UniProtKB=P54920,HUMAN|HGNC=26801|UniProtKB=Q8N8Q8,HUMAN|HGNC=9772|UniProtKB=Q13637,HUMAN|HGNC=21354|UniProtKB=Q86XR7,HUMAN|HGNC=24251|UniProtKB=Q9Y3A6,HUMAN|HGNC=658|UniProtKB=P84085,HUMAN|HGNC=655|UniProtKB=P18085,HUMAN|HGNC=11446|UniProtKB=O00186,HUMAN|HGNC=25694|UniProtKB=Q8TC07,HUMAN|HGNC=9786|UniProtKB=P20340,HUMAN|HGNC=11442|UniProtKB=O15400,HUMAN|HGNC=11429|UniProtKB=O75558,HUMAN|HGNC=10534|UniProtKB=Q9NR31,HUMAN|HGNC=20347|UniProtKB=Q9H9C1,HUMAN|HGNC=18370|UniProtKB=Q9H0U4,HUMAN|HGNC=16996|UniProtKB=Q15363,HUMAN|HGNC=25571|UniProtKB=Q9NVG8,HUMAN|HGNC=24878|UniProtKB=Q9BVK6	intracellular protein transport
GO:0051495	HUMAN|HGNC=12754|UniProtKB=O75083,HUMAN|HGNC=8881|UniProtKB=P07737,HUMAN|HGNC=9847|UniProtKB=P43487	positive regulation of cytoskeleton organization
GO:0003779	HUMAN|HGNC=20040|UniProtKB=Q01518,HUMAN|HGNC=2067|UniProtKB=O00151,HUMAN|HGNC=8881|UniProtKB=P07737,HUMAN|HGNC=17468|UniProtKB=Q96HC4,HUMAN|HGNC=6835|UniProtKB=P78559,HUMAN|HGNC=14653|UniProtKB=Q9HBI1,HUMAN|HGNC=22958|UniProtKB=Q9NR12,HUMAN|HGNC=21295|UniProtKB=Q8TE68	actin binding
GO:0001228	HUMAN|HGNC=6767|UniProtKB=Q15797,HUMAN|HGNC=13109|UniProtKB=P17038,HUMAN|HGNC=13061|UniProtKB=Q14587,HUMAN|HGNC=23708|UniProtKB=Q6ZR52,HUMAN|HGNC=7002|UniProtKB=A6NDR6	DNA-binding transcription activator activity, RNA polymerase II-specific
GO:0003690	HUMAN|HGNC=7183|UniProtKB=P41218,HUMAN|HGNC=4716|UniProtKB=P16403,HUMAN|HGNC=4718|UniProtKB=P10412,HUMAN|HGNC=18807|UniProtKB=O94983,HUMAN|HGNC=4719|UniProtKB=P16401	double-stranded DNA binding
GO:0016567	HUMAN|HGNC=27146|UniProtKB=Q5TA31,HUMAN|HGNC=12458|UniProtKB=P62987,HUMAN|HGNC=12468|UniProtKB=P0CG48,HUMAN|HGNC=12469|UniProtKB=P22314,HUMAN|HGNC=30688|UniProtKB=Q86VP6,HUMAN|HGNC=2552|UniProtKB=Q13617,HUMAN|HGNC=14452|UniProtKB=Q9NYG5,HUMAN|HGNC=2551|UniProtKB=Q13616,HUMAN|HGNC=7732|UniProtKB=Q15843,HUMAN|HGNC=26378|UniProtKB=Q8N7C3,HUMAN|HGNC=25544|UniProtKB=Q9HCI7,HUMAN|HGNC=12463|UniProtKB=P0CG47	protein ubiquitination

GO:0051603	HUMAN|HGNC=2528|UniProtKB=P53634,HUMAN|HGNC=5381|UniProtKB=P14735,HUMAN|HGNC=9472|UniProtKB=Q99538	proteolysis involved in cellular protein catabolic process
GO:0016772	HUMAN|HGNC=9465|UniProtKB=P11908,HUMAN|HGNC=9462|UniProtKB=P60891	transferase activity, transferring phosphorus-containing groups
GO:0045211	HUMAN|HGNC=3338|UniProtKB=Q14247,HUMAN|HGNC=15474|UniProtKB=Q9Y566,HUMAN|HGNC=2695|UniProtKB=Q16643,HUMAN|HGNC=2974|UniProtKB=P50570,HUMAN|HGNC=29125|UniProtKB=Q9UQ16,HUMAN|HGNC=2696|UniProtKB=Q9UJU6,HUMAN|HGNC=4844|UniProtKB=P14317,HUMAN|HGNC=14294|UniProtKB=Q9BYB0	postsynaptic membrane
GO:0030433	HUMAN|HGNC=12028|UniProtKB=P14625,HUMAN|HGNC=13919|UniProtKB=P46379,HUMAN|HGNC=12666|UniProtKB=P55072,HUMAN|HGNC=30550|UniProtKB=O60337,HUMAN|HGNC=5238|UniProtKB=P11021,HUMAN|HGNC=9553|UniProtKB=P62333,HUMAN|HGNC=25035|UniProtKB=Q5VVQ6,HUMAN|HGNC=17943|UniProtKB=Q9GZP9	ubiquitin-dependent ERAD pathway
GO:0004197	HUMAN|HGNC=2528|UniProtKB=P53634,HUMAN|HGNC=1476|UniProtKB=P07384,HUMAN|HGNC=20063|UniProtKB=Q86T82,HUMAN|HGNC=12628|UniProtKB=P45974,HUMAN|HGNC=9472|UniProtKB=Q99538,HUMAN|HGNC=13485|UniProtKB=Q9BXU7,HUMAN|HGNC=20076|UniProtKB=Q96K76	cysteine-type endopeptidase activity
GO:0016197	HUMAN|HGNC=15578|UniProtKB=Q96D71,HUMAN|HGNC=24634|UniProtKB=Q9UBC2,HUMAN|HGNC=3419|UniProtKB=P42566	endosomal transport
GO:0030894	HUMAN|HGNC=26200|UniProtKB=Q9H668	replisome
GO:0051241	HUMAN|HGNC=607|UniProtKB=P02654,HUMAN|HGNC=533|UniProtKB=P04083,HUMAN|HGNC=29884|UniProtKB=Q8TF42,HUMAN|HGNC=7178|UniProtKB=Q13201	negative regulation of multicellular organismal process
GO:0030120	HUMAN|HGNC=10534|UniProtKB=Q9NR31,HUMAN|HGNC=10701|UniProtKB=Q15436	vesicle coat
GO:0061640	HUMAN|HGNC=15848|UniProtKB=Q14141,HUMAN|HGNC=2713|UniProtKB=O75935,HUMAN|HGNC=9164|UniProtKB=Q99719,HUMAN|HGNC=1717|UniProtKB=Q16181,HUMAN|HGNC=7729|UniProtKB=Q15019	cytoskeleton-dependent cytokinesis
GO:0022409	HUMAN|HGNC=3757|UniProtKB=O75955	positive regulation of cell-cell adhesion
GO:0033202	HUMAN|HGNC=26200|UniProtKB=Q9H668	DNA helicase complex
GO:0052745	HUMAN|HGNC=6050|UniProtKB=P29218	inositol phosphate phosphatase activity
GO:0000977	HUMAN|HGNC=7803|UniProtKB=Q12986,HUMAN|HGNC=25397|UniProtKB=Q6MZP7,HUMAN|HGNC=9349|UniProtKB=Q9NQX1,HUMAN|HGNC=15935|UniProtKB=Q9H4I2,HUMAN|HGNC=7446|UniProtKB=Q9Y4I5,HUMAN|HGNC=12879|UniProtKB=P21506	RNA polymerase II transcription regulatory region sequence-specific DNA binding
GO:0050877	HUMAN|HGNC=1406|UniProtKB=Q9Y698,HUMAN|HGNC=2479|UniProtKB=O76096,HUMAN|HGNC=10594|UniProtKB=Q01118,HUMAN|HGNC=10582|UniProtKB=Q9Y5Y9	nervous system process
GO:0006898	HUMAN|HGNC=607|UniProtKB=P02654,HUMAN|HGNC=555|UniProtKB=O43747,HUMAN|HGNC=7228|UniProtKB=P22897,HUMAN|HGNC=2092|UniProtKB=Q00610	receptor-mediated endocytosis
GO:0005216	HUMAN|HGNC=12669|UniProtKB=P21796,HUMAN|HGNC=12674|UniProtKB=Q9Y277,HUMAN|HGNC=12672|UniProtKB=P45880	ion channel activity

PC00157	HUMAN|HGNC=15476|UniProtKB=P63167,HUMAN|HGNC=30535|UniProtKB=Q8WVJ2,HUMAN|HGNC=26664|UniProtKB=Q8N841	microtubule or microtubule-binding cytoskeletal protein
GO:0071363	HUMAN|HGNC=1692|UniProtKB=P08962,HUMAN|HGNC=533|UniProtKB=P04083,HUMAN|HGNC=7178|UniProtKB=Q13201	cellular response to growth factor stimulus
GO:0007010	HUMAN|HGNC=20250|UniProtKB=Q96IF1	cytoskeleton organization
GO:0002718	HUMAN|HGNC=16961|UniProtKB=Q15762	regulation of cytokine production involved in immune response
GO:0120031	HUMAN|HGNC=9621|UniProtKB=Q6IBS0,HUMAN|HGNC=3382|UniProtKB=Q08495,HUMAN|HGNC=25361|UniProtKB=Q8N264,HUMAN|HGNC=14653|UniProtKB=Q9HBI1,HUMAN|HGNC=21295|UniProtKB=Q8TE68	plasma membrane bounded cell projection assembly
GO:0005667	HUMAN|HGNC=19985|UniProtKB=P53999,HUMAN|HGNC=20250|UniProtKB=Q96IF1	transcription regulator complex
GO:0060090	HUMAN|HGNC=26068|UniProtKB=Q6IAA8,HUMAN|HGNC=15848|UniProtKB=Q14141,HUMAN|HGNC=9164|UniProtKB=Q99719,HUMAN|HGNC=1717|UniProtKB=Q16181,HUMAN|HGNC=2905|UniProtKB=O14490,HUMAN|HGNC=7729|UniProtKB=Q15019	molecular adaptor activity
GO:0004721	HUMAN|HGNC=1173|UniProtKB=Q5H9R7,HUMAN|HGNC=14871|UniProtKB=Q96A00,HUMAN|HGNC=14952|UniProtKB=Q8TAE6,HUMAN|HGNC=9304|UniProtKB=P63151	phosphoprotein phosphatase activity
GO:0090307	HUMAN|HGNC=5048|UniProtKB=Q00839	mitotic spindle assembly
GO:0005697	HUMAN|HGNC=5048|UniProtKB=Q00839	telomerase holoenzyme complex
GO:0046890	HUMAN|HGNC=533|UniProtKB=P04083	regulation of lipid biosynthetic process
GO:0035591	HUMAN|HGNC=15474|UniProtKB=Q9Y566,HUMAN|HGNC=14294|UniProtKB=Q9BYB0	signaling adaptor activity
GO:0051896	HUMAN|HGNC=18233|UniProtKB=Q8NDB2	regulation of protein kinase B signaling
GO:0043001	HUMAN|HGNC=26486|UniProtKB=Q6PJW8,HUMAN|HGNC=17643|UniProtKB=Q9HD26,HUMAN|HGNC=8016|UniProtKB=P46459	Golgi to plasma membrane protein transport
GO:0010639	HUMAN|HGNC=4374|UniProtKB=O60234	negative regulation of organelle organization
GO:0000727	HUMAN|HGNC=1745|UniProtKB=O00311	double-strand break repair via break-induced replication
GO:0030667	HUMAN|HGNC=2689|UniProtKB=P09172	secretory granule membrane
GO:0051445	HUMAN|HGNC=12401|UniProtKB=P33981	regulation of meiotic cell cycle
GO:0008203	HUMAN|HGNC=615|UniProtKB=Q13790,HUMAN|HGNC=613|UniProtKB=P02649,HUMAN|HGNC=1869|UniProtKB=P11597	cholesterol metabolic process
GO:0000288	HUMAN|HGNC=9207|UniProtKB=Q9UFF9	nuclear-transcribed mRNA catabolic process, deadenylation-dependent decay
GO:0004553	HUMAN|HGNC=4298|UniProtKB=P16278,HUMAN|HGNC=6740|UniProtKB=P61626	hydrolase activity, hydrolyzing O-glycosyl compounds

<table>
<tr><th></th><th></th><th></th></tr>
<tr><td>GO:0016741</td><td>HUMAN|HGNC=794|UniProtKB=P31939</td><td>transferase activity, transferring one-carbon groups</td></tr>
<tr><td>GO:0015986</td><td>HUMAN|HGNC=14247|UniProtKB=O75964,HUMAN|HGNC=7421|UniProtKB=P00403,HUMAN|HGNC=837|UniProtKB=P30049,HUMAN|HGNC=823|UniProtKB=P25705,HUMAN|HGNC=19986|UniProtKB=P99999,HUMAN|HGNC=850|UniProtKB=P48047,HUMAN|HGNC=8898|UniProtKB=P07205,HUMAN|HGNC=2265|UniProtKB=P13073,HUMAN|HGNC=845|UniProtKB=O75947,HUMAN|HGNC=2288|UniProtKB=P14406,HUMAN|HGNC=2267|UniProtKB=P20674,HUMAN|HGNC=12582|UniProtKB=P14927,HUMAN|HGNC=12590|UniProtKB=P07919,HUMAN|HGNC=833|UniProtKB=P36542,HUMAN|HGNC=2292|UniProtKB=P15954,HUMAN|HGNC=830|UniProtKB=P06576</td><td>ATP synthesis coupled proton transport</td></tr>
<tr><td>GO:0099500</td><td>HUMAN|HGNC=31332|UniProtKB=Q6IQ22,HUMAN|HGNC=7007|UniProtKB=P61006,HUMAN|HGNC=20120|UniProtKB=Q17RC7,HUMAN|HGNC=7641|UniProtKB=P54920,HUMAN|HGNC=11491|UniProtKB=P43405,HUMAN|HGNC=9778|UniProtKB=P20337,HUMAN|HGNC=11131|UniProtKB=O00161,HUMAN|HGNC=533|UniProtKB=P04083,HUMAN|HGNC=9857|UniProtKB=P61224,HUMAN|HGNC=9855|UniProtKB=P62834,HUMAN|HGNC=11446|UniProtKB=O00186,HUMAN|HGNC=11445|UniProtKB=Q15833,HUMAN|HGNC=9164|UniProtKB=Q99719,HUMAN|HGNC=11429|UniProtKB=O75558,HUMAN|HGNC=11510|UniProtKB=Q8N9I0,HUMAN|HGNC=15588|UniProtKB=Q96C24,HUMAN|HGNC=30389|UniProtKB=Q96A65,HUMAN|HGNC=9761|UniProtKB=Q15907,HUMAN|HGNC=11512|UniProtKB=Q9H2B2,HUMAN|HGNC=8515|UniProtKB=Q9HC10,HUMAN|HGNC=9767|UniProtKB=O00194</td><td>vesicle fusion to plasma membrane</td></tr>
<tr><td>GO:1901215</td><td>HUMAN|HGNC=613|UniProtKB=P02649</td><td>negative regulation of neuron death</td></tr>
<tr><td>GO:0000226</td><td>HUMAN|HGNC=12407|UniProtKB=P68366,HUMAN|HGNC=16051|UniProtKB=Q8TEW0,HUMAN|HGNC=12410|UniProtKB=Q9NY65,HUMAN|HGNC=11582|UniProtKB=Q15813,HUMAN|HGNC=20778|UniProtKB=P07437,HUMAN|HGNC=16257|UniProtKB=Q9H4B7,HUMAN|HGNC=20766|UniProtKB=Q71U36,HUMAN|HGNC=20774|UniProtKB=P04350,HUMAN|HGNC=20771|UniProtKB=P68371,HUMAN|HGNC=26664|UniProtKB=Q8N841</td><td>microtubule cytoskeleton organization</td></tr>
<tr><td>GO:0015031</td><td>HUMAN|HGNC=1406|UniProtKB=Q9Y698,HUMAN|HGNC=2974|UniProtKB=P50570,HUMAN|HGNC=29125|UniProtKB=Q9UQ16,HUMAN|HGNC=603|UniProtKB=P04114,HUMAN|HGNC=19760|UniProtKB=Q96T51</td><td>protein transport</td></tr>
<tr><td>GO:0007268</td><td>HUMAN|HGNC=10594|UniProtKB=Q01118,HUMAN|HGNC=11138|UniProtKB=P37840,HUMAN|HGNC=4390|UniProtKB=P50148,HUMAN|HGNC=10582|UniProtKB=Q9Y5Y9</td><td>chemical synaptic transmission</td></tr>
<tr><td>PC00072</td><td>HUMAN|HGNC=30167|UniProtKB=P13667,HUMAN|HGNC=11476|UniProtKB=O15260,HUMAN|HGNC=1455|UniProtKB=P27797,HUMAN|HGNC=1735|UniProtKB=Q16543,HUMAN|HGNC=11582|UniProtKB=Q15813,HUMAN|HGNC=8548|UniProtKB=P07237,HUMAN|HGNC=9261|UniProtKB=Q13356,HUMAN|HGNC=8988|UniProtKB=Q13526,HUMAN|HGNC=1473|UniProtKB=P27824,HUMAN|HGNC=4606|UniProtKB=P30101,HUMAN|HGNC=5228|UniProtKB=P25686,HUMAN|HGNC=9253|UniProtKB=P62937,HUMAN|HGNC=2711|UniProtKB=Q14203,HUMAN|HGNC=13919|UniProtKB=P46379,HUMAN|HGNC=5461|UniProtKB=P78318,HUMAN|HGNC=24718|Uni</td><td>chaperone</td></tr>
</table>

	ProtKB=Q96JJ7,HUMAN|HGNC=21073|UniProtKB=Q8NBS9,HUMAN|HGNC=24811|UniProtKB=Q14554,HUMAN|HGNC=5270|UniProtKB=P25685,HUMAN|HGNC=30168|UniProtKB=Q15084,HUMAN|HGNC=3711|UniProtKB=P62942,HUMAN|HGNC=7910|UniProtKB=P06748,HUMAN|HGNC=9255|UniProtKB=P23284,HUMAN|HGNC=9259|UniProtKB=P30405	
GO:0019905	HUMAN|HGNC=20726|UniProtKB=Q8WVM8,HUMAN|HGNC=7641|UniProtKB=P54920,HUMAN|HGNC=11510|UniProtKB=Q8N9I0,HUMAN|HGNC=11131|UniProtKB=O00161,HUMAN|HGNC=12647|UniProtKB=Q9BV40	syntaxin binding
GO:0098796	HUMAN|HGNC=26068|UniProtKB=Q6IAA8,HUMAN|HGNC=17955|UniProtKB=O43504,HUMAN|HGNC=18587|UniProtKB=O94901,HUMAN|HGNC=26212|UniProtKB=P61009,HUMAN|HGNC=12666|UniProtKB=P55072,HUMAN|HGNC=13487|UniProtKB=Q96QK1,HUMAN|HGNC=1628|UniProtKB=P08571,HUMAN|HGNC=17943|UniProtKB=Q9GZP9,HUMAN|HGNC=15606|UniProtKB=Q9UHA4	membrane protein complex
GO:0004713	HUMAN|HGNC=6171|UniProtKB=Q08881,HUMAN|HGNC=4037|UniProtKB=P06241,HUMAN|HGNC=12841|UniProtKB=P07947,HUMAN|HGNC=11283|UniProtKB=P12931,HUMAN|HGNC=6735|UniProtKB=P07948,HUMAN|HGNC=2444|UniProtKB=P41240	protein tyrosine kinase activity
GO:0030335	HUMAN|HGNC=9065|UniProtKB=P19174,HUMAN|HGNC=533|UniProtKB=P04083	positive regulation of cell migration
PC00222	HUMAN|HGNC=3214|UniProtKB=P13639,HUMAN|HGNC=3208|UniProtKB=P24534,HUMAN|HGNC=12420|UniProtKB=P49411,HUMAN|HGNC=3211|UniProtKB=P29692	translation elongation factor
GO:0043087	HUMAN|HGNC=17168|UniProtKB=Q9H2M9	regulation of GTPase activity
GO:0051298	HUMAN|HGNC=28959|UniProtKB=Q14008,HUMAN|HGNC=7910|UniProtKB=P06748	centrosome duplication
GO:0030426	HUMAN|HGNC=11148|UniProtKB=Q16658	growth cone
GO:0046872	HUMAN|HGNC=5269|UniProtKB=P61604	metal ion binding
GO:0007528	HUMAN|HGNC=15474|UniProtKB=Q9Y566,HUMAN|HGNC=14294|UniProtKB=Q9BYB0	neuromuscular junction development
GO:0097061	HUMAN|HGNC=3338|UniProtKB=Q14247,HUMAN|HGNC=15474|UniProtKB=Q9Y566,HUMAN|HGNC=2695|UniProtKB=Q16643,HUMAN|HGNC=2696|UniProtKB=Q9UJU6,HUMAN|HGNC=4844|UniProtKB=P14317,HUMAN|HGNC=14294|UniProtKB=Q9BYB0	dendritic spine organization
GO:0031400	HUMAN|HGNC=21314|UniProtKB=Q8WXC6	negative regulation of protein modification process
GO:0036503	HUMAN|HGNC=15663|UniProtKB=Q9NYU2	ERAD pathway
GO:0004672	HUMAN|HGNC=10372|UniProtKB=P05386,HUMAN|HGNC=6040|UniProtKB=Q13418	protein kinase activity
PC00093	HUMAN|HGNC=28422|UniProtKB=Q8NCE0	endodeoxyribonuclease
GO:0009967	HUMAN|HGNC=6616|UniProtKB=P48059,HUMAN|HGNC=15474|UniProtKB=Q9Y566,HUMAN|HGNC=16961|UniProtKB=Q15762,HUMAN|HGNC=14294|UniProtKB=Q9BYB0,HUMAN|HGNC=11119|UniProtKB=Q99835,HUMAN|HGNC=11766|UniProtKB=P01137	positive regulation of signal transduction
GO:0044271	HUMAN|HGNC=2689|UniProtKB=P09172	cellular nitrogen compound biosynthetic process

<table>
<tr><td>GO:0048311</td><td>HUMAN|HGNC=13206|UniProtKB=O60296,HUMAN|HGNC=2973|UniProtKB=O00429</td><td>mitochondrion distribution</td></tr>
<tr><td>GO:0007417</td><td>HUMAN|HGNC=11119|UniProtKB=Q99835</td><td>central nervous system development</td></tr>
<tr><td>GO:0006695</td><td>HUMAN|HGNC=9141|UniProtKB=Q15126</td><td>cholesterol biosynthetic process</td></tr>
<tr><td>GO:0031301</td><td>HUMAN|HGNC=8533|UniProtKB=P51575,HUMAN|HGNC=6501|UniProtKB=P13473</td><td>integral component of organelle membrane</td></tr>
<tr><td>GO:0005875</td><td>HUMAN|HGNC=6325|UniProtKB=O60282,HUMAN|HGNC=2713|UniProtKB=O75935,HUMAN|HGNC=2712|UniProtKB=Q13561,HUMAN|HGNC=6317|UniProtKB=O43896,HUMAN|HGNC=6835|UniProtKB=P78559,HUMAN|HGNC=17060|UniProtKB=Q92845</td><td>microtubule associated complex</td></tr>
<tr><td>GO:0015631</td><td>HUMAN|HGNC=14313|UniProtKB=Q8WYA0,HUMAN|HGNC=11582|UniProtKB=Q15813,HUMAN|HGNC=6510|UniProtKB=P16949,HUMAN|HGNC=11579|UniProtKB=O75347,HUMAN|HGNC=26664|UniProtKB=Q8N841</td><td>tubulin binding</td></tr>
<tr><td>GO:0051082</td><td>HUMAN|HGNC=1623|UniProtKB=P50990,HUMAN|HGNC=1617|UniProtKB=P50991,HUMAN|HGNC=1622|UniProtKB=Q99832,HUMAN|HGNC=5239|UniProtKB=P17066,HUMAN|HGNC=1735|UniProtKB=Q16543,HUMAN|HGNC=1615|UniProtKB=P78371,HUMAN|HGNC=5241|UniProtKB=P11142,HUMAN|HGNC=5269|UniProtKB=P61604,HUMAN|HGNC=5253|UniProtKB=P07900,HUMAN|HGNC=11655|UniProtKB=P17987,HUMAN|HGNC=1618|UniProtKB=P48643,HUMAN|HGNC=5258|UniProtKB=P08238,HUMAN|HGNC=1620|UniProtKB=P40227,HUMAN|HGNC=12028|UniProtKB=P14625,HUMAN|HGNC=5238|UniProtKB=P11021,HUMAN|HGNC=32153|UniProtKB=A6NM43,HUMAN|HGNC=5270|UniProtKB=P25685,HUMAN|HGNC=5240|UniProtKB=P48741,HUMAN|HGNC=30535|UniProtKB=Q8WVJ2,HUMAN|HGNC=15663|UniProtKB=Q9NYU2,HUMAN|HGNC=7629|UniProtKB=E9PAV3</td><td>unfolded protein binding</td></tr>
<tr><td>GO:0006511</td><td>HUMAN|HGNC=463|UniProtKB=Q9UKV5,HUMAN|HGNC=11617|UniProtKB=Q15369,HUMAN|HGNC=9556|UniProtKB=O00231,HUMAN|HGNC=9571|UniProtKB=Q92530,HUMAN|HGNC=12469|UniProtKB=P22314,HUMAN|HGNC=12476|UniProtKB=P61077,HUMAN|HGNC=16476|UniProtKB=Q9H0E2,HUMAN|HGNC=12475|UniProtKB=P62837,HUMAN|HGNC=14452|UniProtKB=Q9NYG5,HUMAN|HGNC=2239|UniProtKB=Q9UNS2</td><td>ubiquitin-dependent protein catabolic process</td></tr>
<tr><td>GO:0030863</td><td>HUMAN|HGNC=1117|UniProtKB=Q9UPA5,HUMAN|HGNC=13406|UniProtKB=Q9Y6V0</td><td>cortical cytoskeleton</td></tr>
<tr><td>GO:0046464</td><td>HUMAN|HGNC=13921|UniProtKB=O95870</td><td>acylglycerol catabolic process</td></tr>
<tr><td>GO:0006816</td><td>HUMAN|HGNC=1393|UniProtKB=O60840</td><td>calcium ion transport</td></tr>
<tr><td>GO:0031146</td><td>HUMAN|HGNC=2552|UniProtKB=Q13617,HUMAN|HGNC=10899|UniProtKB=P63208</td><td>SCF-dependent proteasomal ubiquitin-dependent protein catabolic process</td></tr>
<tr><td>GO:0045446</td><td>HUMAN|HGNC=14685|UniProtKB=Q9Y624,HUMAN|HGNC=243|UniProtKB=P35611,HUMAN|HGNC=11828|UniProtKB=Q9UDY2</td><td>endothelial cell differentiation</td></tr>
<tr><td>GO:0045197</td><td>HUMAN|HGNC=16051|UniProtKB=Q8TEW0,HUMAN|HGNC=30377|UniProtKB=Q14160</td><td>establishment or maintenance of epithelial cell apical/basal polarity</td></tr>
<tr><td>GO:0070887</td><td>HUMAN|HGNC=2315|UniProtKB=Q96FN4,HUMAN|HGNC=11510|UniProtKB=Q8N9I0,HUMAN|HGNC=11512|UniProtKB=Q9H2B2,HUMAN|HGNC=82|UniProtKB=P09110</td><td>cellular response to chemical stimulus</td></tr>
</table>

<table>
<tr><td>GO:0002286</td><td>HUMAN|HGNC=533|UniProtKB=P04083</td><td>T cell activation involved in immune response</td></tr>
<tr><td>PC00117</td><td>HUMAN|HGNC=24526|UniProtKB=Q6DD88,HUMAN|HGNC=4395|UniProtKB=P19086,HUMAN|HGNC=4384|UniProtKB=P63096,HUMAN|HGNC=4398|UniProtKB=P62879,HUMAN|HGNC=4381|UniProtKB=Q14344,HUMAN|HGNC=4387|UniProtKB=P08754,HUMAN|HGNC=20731|UniProtKB=Q9HAV0,HUMAN|HGNC=4385|UniProtKB=P04899,HUMAN|HGNC=4393|UniProtKB=P11488,HUMAN|HGNC=4390|UniProtKB=P50148,HUMAN|HGNC=11231|UniProtKB=Q8WXF7,HUMAN|HGNC=4396|UniProtKB=P62873</td><td>heterotrimeric G-protein</td></tr>
<tr><td>GO:0000118</td><td>HUMAN|HGNC=29989|UniProtKB=Q86YP4,HUMAN|HGNC=10474|UniProtKB=Q9Y265,HUMAN|HGNC=10475|UniProtKB=Q9Y230</td><td>histone deacetylase complex</td></tr>
<tr><td>GO:0045272</td><td>HUMAN|HGNC=7717|UniProtKB=P19404</td><td>plasma membrane respiratory chain complex I</td></tr>
<tr><td>GO:0048018</td><td>HUMAN|HGNC=3157|UniProtKB=Q92838</td><td>receptor ligand activity</td></tr>
<tr><td>GO:0005179</td><td>HUMAN|HGNC=3964|UniProtKB=P01225,HUMAN|HGNC=16452|UniProtKB=P0DN86,HUMAN|HGNC=16721|UniProtKB=A6NKQ9</td><td>hormone activity</td></tr>
<tr><td>GO:0042102</td><td>HUMAN|HGNC=11491|UniProtKB=P43405,HUMAN|HGNC=533|UniProtKB=P04083</td><td>positive regulation of T cell proliferation</td></tr>
<tr><td>GO:0031902</td><td>HUMAN|HGNC=26068|UniProtKB=Q6IAA8,HUMAN|HGNC=6499|UniProtKB=P11279,HUMAN|HGNC=17955|UniProtKB=O43504,HUMAN|HGNC=6501|UniProtKB=P13473,HUMAN|HGNC=15606|UniProtKB=Q9UHA4</td><td>late endosome membrane</td></tr>
<tr><td>GO:0000974</td><td>HUMAN|HGNC=29513|UniProtKB=O60306,HUMAN|HGNC=5048|UniProtKB=Q00839,HUMAN|HGNC=1743|UniProtKB=Q99459</td><td>Prp19 complex</td></tr>
<tr><td>GO:0051402</td><td>HUMAN|HGNC=17865|UniProtKB=Q96A26,HUMAN|HGNC=613|UniProtKB=P02649</td><td>neuron apoptotic process</td></tr>
<tr><td>GO:0007040</td><td>HUMAN|HGNC=9788|UniProtKB=P51149</td><td>lysosome organization</td></tr>
<tr><td>PC00202</td><td>HUMAN|HGNC=10372|UniProtKB=P05386,HUMAN|HGNC=14511|UniProtKB=P82663,HUMAN|HGNC=10377|UniProtKB=P05387,HUMAN|HGNC=10404|UniProtKB=P15880,HUMAN|HGNC=10363|UniProtKB=P18124,HUMAN|HGNC=10385|UniProtKB=P25398,HUMAN|HGNC=10378|UniProtKB=P52815,HUMAN|HGNC=10396|UniProtKB=P62249,HUMAN|HGNC=10305|UniProtKB=P50914,HUMAN|HGNC=10353|UniProtKB=P36578</td><td>ribosomal protein</td></tr>
<tr><td>GO:0006998</td><td>HUMAN|HGNC=18587|UniProtKB=O94901,HUMAN|HGNC=15912|UniProtKB=Q9UNZ2</td><td>nuclear envelope organization</td></tr>
<tr><td>GO:0051656</td><td>HUMAN|HGNC=16051|UniProtKB=Q8TEW0,HUMAN|HGNC=11491|UniProtKB=P43405</td><td>establishment of organelle localization</td></tr>
<tr><td>PC00174</td><td>HUMAN|HGNC=8087|UniProtKB=P29728</td><td>nucleotidyltransferase</td></tr>
<tr><td>GO:0051879</td><td>HUMAN|HGNC=11387|UniProtKB=P31948,HUMAN|HGNC=30594|UniProtKB=Q9H3U1</td><td>Hsp90 protein binding</td></tr>
<tr><td>GO:0055085</td><td>HUMAN|HGNC=11030|UniProtKB=Q9Y6R1,HUMAN|HGNC=55|UniProtKB=O15439,HUMAN|HGNC=11027|UniProtKB=P02730,HUMAN|HGNC=17292|UniProtKB=Q8IWA5,HUMAN|HGNC=11034|UniProtKB=Q2Y0W8</td><td>transmembrane transport</td></tr>
<tr><td>GO:0042742</td><td>HUMAN|HGNC=5575|UniProtKB=A0A0B4J1V2,HUMAN|HGNC=5645|UniProtKB=A0A0C4DH34,HUMAN|HGNC=5549|UniProtKB=A0A0C4DH31,HUMAN|HGNC=5554|UniProtKB=P01743,HUMAN|HGNC=5480|UniProtKB=P01880,HUMAN|HGNC=5659|UniProtKB=A0A0C4DH38,HUMAN|HGNC=5634|UniProtKB=A0A075B7F0,HUMAN|HGNC=38476|UniProtKB=B9A064,HUMAN|HGNC=5861|UniProtK</td><td>defense response to bacterium</td></tr>
</table>

	B=A0M8Q6,HUMAN|HGNC=5647|UniProtKB=A0A087WSY4,HUMAN|HGNC=5563|UniProtKB=A0A075B7D0,HUMAN|HGNC=5856|UniProtKB=P0DOY2,HUMAN|HGNC=5606|UniProtKB=P01763,HUMAN|HGNC=5576|UniProtKB=P01817,HUMAN|HGNC=5528|UniProtKB=P01861,HUMAN|HGNC=5541|UniProtKB=P01871,HUMAN|HGNC=5585|UniProtKB=A0A0C4DH32,HUMAN|HGNC=5551|UniProtKB=A0A0C4DH33,HUMAN|HGNC=5526|UniProtKB=P01859,HUMAN|HGNC=5657|UniProtKB=P0DP08,HUMAN|Ensembl=ENSG00000282639|UniProtKB=A0A0J9YX35,HUMAN|Gene=HV309_HUMAN|UniProtKB=P01782,HUMAN|HGNC=5654|UniProtKB=P01825,HUMAN|HGNC=5553|UniProtKB=A0A0A0MS14,HUMAN|Gene=HV108_HUMAN|UniProtKB=P0DP01,HUMAN|HGNC=5624|UniProtKB=A0A0B4J1X5,HUMAN|HGNC=5620|UniProtKB=P01780,HUMAN|HGNC=5598|UniProtKB=A0A0C4DH35,HUMAN|HGNC=5580|UniProtKB=P01762,HUMAN|HGNC=5633|UniProtKB=A0A075B7D8,HUMAN|HGNC=5582|UniProtKB=A0A0B4J1V0,HUMAN|HGNC=5478|UniProtKB=P01876,HUMAN|HGNC=5617|UniProtKB=A0A075B6Q5,HUMAN|HGNC=5594|UniProtKB=P0DP03,HUMAN|HGNC=5655|UniProtKB=A0A0C4DH41,HUMAN|HGNC=5636|UniProtKB=A0A075B7B8,HUMAN|HGNC=5586|UniProtKB=A0A0B4J1V1,HUMAN|HGNC=5581|UniProtKB=P01766,HUMAN|HGNC=5622|UniProtKB=A0A0B4J1Y9,HUMAN|HGNC=5652|UniProtKB=A0A075B6R2,HUMAN|HGNC=5593|UniProtKB=P0DP02,HUMAN|HGNC=5479|UniProtKB=P01877,HUMAN|HGNC=5619|UniProtKB=A0A0C4DH42,HUMAN|HGNC=5596|UniProtKB=P01772,HUMAN|HGNC=5550|UniProtKB=P23083,HUMAN|HGNC=5604|UniProtKB=A0A0B4J1X8,HUMAN|HGNC=5525|UniProtKB=P01857,HUMAN|HGNC=5662|UniProtKB=A0A0B4J1U7,HUMAN|HGNC=5601|UniProtKB=A0A0C4DH36,HUMAN|HGNC=5716|UniProtKB=P01834,HUMAN|HGNC=5588|UniProtKB=P01764,HUMAN|HGNC=5857|UniProtKB=P0DOY3,HUMAN|HGNC=5610|UniProtKB=P01767,HUMAN|Ensembl=ENSG00000282150|UniProtKB=A0A0J9YY99,HUMAN|HGNC=5527|UniProtKB=P01860	
GO:0004017	HUMAN|HGNC=362|UniProtKB=P54819,HUMAN|HGNC=363|UniProtKB=P27144,HUMAN|HGNC=361|UniProtKB=P00568,HUMAN|HGNC=18170|UniProtKB=P30085,HUMAN|HGNC=17376|UniProtKB=Q9UIJ7	adenylate kinase activity
GO:0005776	HUMAN|HGNC=6501|UniProtKB=P13473	autophagosome
GO:0030071	HUMAN|HGNC=15713|UniProtKB=Q9UJX4,HUMAN|HGNC=14452|UniProtKB=Q9NYG5	regulation of mitotic metaphase/anaphase transition
GO:0006637	HUMAN|HGNC=9141|UniProtKB=Q15126	acyl-CoA metabolic process
GO:0051223	HUMAN|HGNC=26486|UniProtKB=Q6PJW8,HUMAN|HGNC=10534|UniProtKB=Q9NR31,HUMAN|HGNC=17643|UniProtKB=Q9HD26	regulation of protein transport
PC00218	HUMAN|HGNC=6703|UniProtKB=Q9Y608,HUMAN|HGNC=6767|UniProtKB=Q15797,HUMAN|HGNC=29989|UniProtKB=Q86YP4,HUMAN|HGNC=30547|UniProtKB=Q96K17,HUMAN|HGNC=7183|UniProtKB=P41218,HUMAN|HGNC=11364|UniProtKB=P40763,HUMAN|HGNC=6702|UniProtKB=Q32MZ4,HUMAN|HGNC=18807|UniProtKB=O94983	DNA-binding transcription factor
GO:0000724	HUMAN|HGNC=26200|UniProtKB=Q9H668,HUMAN|HGNC=1101|UniProtKB=P51587,HUMAN|HGNC=17196|UniProtKB=Q8NG08	double-strand break repair via homologous recombination

<table>
<tr><td>GO:0042273</td><td>HUMAN|HGNC=7910|UniProtKB=P06748,HUMAN|HGNC=10305|UniProtKB=P50914</td><td>ribosomal large subunit biogenesis</td></tr>
<tr><td>GO:0007173</td><td>HUMAN|HGNC=11283|UniProtKB=P12931,HUMAN|HGNC=25153|UniProtKB=Q96EY5</td><td>epidermal growth factor receptor signaling pathway</td></tr>
<tr><td>GO:0034703</td><td>HUMAN|HGNC=1406|UniProtKB=Q9Y698,HUMAN|HGNC=3338|UniProtKB=Q14247,HUMAN|HGNC=10594|UniProtKB=Q01118,HUMAN|HGNC=10582|UniProtKB=Q9Y5Y9</td><td>cation channel complex</td></tr>
<tr><td>GO:0006465</td><td>HUMAN|HGNC=26212|UniProtKB=P61009</td><td>signal peptide processing</td></tr>
<tr><td>GO:0005882</td><td>HUMAN|HGNC=1090|UniProtKB=Q03001,HUMAN|HGNC=9069|UniProtKB=Q15149</td><td>intermediate filament</td></tr>
<tr><td>GO:0034455</td><td>HUMAN|HGNC=12755|UniProtKB=Q9UNX4</td><td>t-UTP complex</td></tr>
<tr><td>GO:1902903</td><td>HUMAN|HGNC=6835|UniProtKB=P78559</td><td>regulation of supramolecular fiber organization</td></tr>
<tr><td>GO:0017148</td><td>HUMAN|HGNC=17970|UniProtKB=Q9BPZ3,HUMAN|HGNC=9207|UniProtKB=Q9UFF9</td><td>negative regulation of translation</td></tr>
<tr><td>GO:0004016</td><td>HUMAN|HGNC=237|UniProtKB=O43306</td><td>adenylate cyclase activity</td></tr>
<tr><td>GO:0032640</td><td>HUMAN|HGNC=6517|UniProtKB=P18428</td><td>tumor necrosis factor production</td></tr>
<tr><td>GO:0005669</td><td>HUMAN|HGNC=17305|UniProtKB=Q9Y6J9</td><td>transcription factor TFIID complex</td></tr>
<tr><td>GO:0061013</td><td>HUMAN|HGNC=29190|UniProtKB=Q9UPQ9</td><td>regulation of mRNA catabolic process</td></tr>
<tr><td>GO:0005643</td><td>HUMAN|HGNC=8062|UniProtKB=P49790,HUMAN|HGNC=30052|UniProtKB=Q8TEM1,HUMAN|HGNC=9847|UniProtKB=P43487,HUMAN|HGNC=17859|UniProtKB=Q5SRE5</td><td>nuclear pore</td></tr>
<tr><td>GO:0003008</td><td>HUMAN|HGNC=14685|UniProtKB=Q9Y624,HUMAN|HGNC=11756|UniProtKB=Q03403</td><td>system process</td></tr>
<tr><td>GO:0007041</td><td>HUMAN|HGNC=1406|UniProtKB=Q9Y698</td><td>lysosomal transport</td></tr>
<tr><td>GO:0043022</td><td>HUMAN|HGNC=3214|UniProtKB=P13639,HUMAN|HGNC=1243|UniProtKB=Q07021,HUMAN|HGNC=3265|UniProtKB=P05198</td><td>ribosome binding</td></tr>
<tr><td>GO:0022618</td><td>HUMAN|HGNC=10474|UniProtKB=Q9Y265,HUMAN|Gene=EIF2S3L|UniProtKB=Q2VIR3,HUMAN|HGNC=3285|UniProtKB=P23588,HUMAN|HGNC=10475|UniProtKB=Q9Y230</td><td>ribonucleoprotein complex assembly</td></tr>
<tr><td>PC00235</td><td>HUMAN|HGNC=2230|UniProtKB=P53621,HUMAN|HGNC=21354|UniProtKB=Q86XR7,HUMAN|HGNC=575|UniProtKB=Q9Y587,HUMAN|HGNC=24251|UniProtKB=Q9Y3A6,HUMAN|HGNC=2231|UniProtKB=P53618,HUMAN|HGNC=10701|UniProtKB=Q15436,HUMAN|HGNC=16998|UniProtKB=P49755,HUMAN|HGNC=2092|UniProtKB=Q00610,HUMAN|HGNC=649|UniProtKB=P48444,HUMAN|HGNC=2090|UniProtKB=P09496,HUMAN|HGNC=2232|UniProtKB=P35606,HUMAN|HGNC=16996|UniProtKB=Q15363,HUMAN|HGNC=24878|UniProtKB=Q9BVK6</td><td>vesicle coat protein</td></tr>
<tr><td>GO:0015485</td><td>HUMAN|HGNC=8504|UniProtKB=Q969R2,HUMAN|HGNC=544|UniProtKB=P08133,HUMAN|HGNC=1869|UniProtKB=P11597,HUMAN|HGNC=603|UniProtKB=P04114</td><td>cholesterol binding</td></tr>
<tr><td>GO:0005829</td><td>HUMAN|HGNC=17169|UniProtKB=Q13162,HUMAN|HGNC=16753|UniProtKB=P30041,HUMAN|HGNC=7413|UniProtKB=Q13126,HUMAN|HGNC=5382|UniProtKB=O75874,HUMAN|HGNC=16369|UniProtKB=Q99497,HUMAN|HGNC=4553|UniProtKB=P07203,HUMAN</td><td>cytosol</td></tr>
</table>

|HGNC=9554|UniProtKB=Q99460,HUMAN|HGNC=8988|UniProtKB
=Q13526,HUMAN|HGNC=463|UniProtKB=Q9UKV5,HUMAN|HGNC
=414|UniProtKB=P04075,HUMAN|HGNC=9353|UniProtKB=P32119
,HUMAN|HGNC=4831|UniProtKB=P69891,HUMAN|HGNC=4458|U
niProtKB=P06744,HUMAN|HGNC=1742|UniProtKB=P50851,HUMA
N|HGNC=29035|UniProtKB=O43314,HUMAN|HGNC=2431|UniPro
tKB=P55060,HUMAN|HGNC=8878|UniProtKB=Q01813,HUMAN|H
GNC=395|UniProtKB=P13716,HUMAN|HGNC=9275|UniProtKB=P3
5813,HUMAN|HGNC=3211|UniProtKB=P29692,HUMAN|HGNC=13
485|UniProtKB=Q9BXU7,HUMAN|HGNC=25153|UniProtKB=Q96EY
5,HUMAN|HGNC=11179|UniProtKB=P00441,HUMAN|HGNC=5253
|UniProtKB=P07900,HUMAN|HGNC=25648|UniProtKB=Q8N5J2,H
UMAN|HGNC=5258|UniProtKB=P08238,HUMAN|HGNC=4922|Uni
ProtKB=P19367,HUMAN|HGNC=8087|UniProtKB=P29728,HUMAN
|HGNC=9852|UniProtKB=O95373,HUMAN|HGNC=19388|UniProtK
B=P49593,HUMAN|HGNC=3148|UniProtKB=P19971,HUMAN|HGN
C=25035|UniProtKB=Q5VVQ6,HUMAN|HGNC=9859|UniProtKB=P5
2306,HUMAN|HGNC=24552|UniProtKB=Q3LXA3,HUMAN|HGNC=4
141|UniProtKB=P04406,HUMAN|HGNC=9304|UniProtKB=P63151,
HUMAN|HGNC=4116|UniProtKB=Q14376,HUMAN|HGNC=20726|
UniProtKB=Q8WVM8,HUMAN|HGNC=4638|UniProtKB=P09211,HU
MAN|HGNC=678|UniProtKB=P52565,HUMAN|HGNC=361|UniProt
KB=P00568,HUMAN|HGNC=9352|UniProtKB=Q06830,HUMAN|HG
NC=9552|UniProtKB=P62195,HUMAN|HGNC=21539|UniProtKB=Q
9BX46,HUMAN|HGNC=12016|UniProtKB=P29144,HUMAN|HGNC=
8898|UniProtKB=P07205,HUMAN|HGNC=4827|UniProtKB=P68871
,HUMAN|HGNC=24437|UniProtKB=Q96KP4,HUMAN|HGNC=1050|
UniProtKB=P55957,HUMAN|HGNC=3350|UniProtKB=P06733,HUM
AN|HGNC=13919|UniProtKB=P46379,HUMAN|HGNC=649|UniProt
KB=P48444,HUMAN|HGNC=291|UniProtKB=P30566,HUMAN|HGN
C=8587|UniProtKB=P22234,HUMAN|HGNC=12666|UniProtKB=P5
5072,HUMAN|HGNC=2482|UniProtKB=P04080,HUMAN|HGNC=46
9|UniProtKB=Q01433,HUMAN|HGNC=11579|UniProtKB=O75347,
HUMAN|HGNC=9813|UniProtKB=P54727,HUMAN|HGNC=8891|U
niProtKB=P52209,HUMAN|HGNC=11767|UniProtKB=O43294,HUM
AN|HGNC=4624|UniProtKB=P48637,HUMAN|HGNC=5237|UniProt
KB=P34932,HUMAN|HGNC=12009|UniProtKB=P60174,HUMAN|H
GNC=20063|UniProtKB=Q86T82,HUMAN|HGNC=20675|UniProtKB
=Q96KN2,HUMAN|HGNC=794|UniProtKB=P31939,HUMAN|HGNC
=343|UniProtKB=P23526,HUMAN|HGNC=9314|UniProtKB=Q0820
9,HUMAN|HGNC=255|UniProtKB=P28332,HUMAN|HGNC=380|Un
iProtKB=P14550,HUMAN|HGNC=6835|UniProtKB=P78559,HUMAN
|Gene=EIF2S3L|UniProtKB=Q2VIR3,HUMAN|HGNC=16969|UniPro
tKB=Q92598,HUMAN|HGNC=8504|UniProtKB=Q969R2,HUMAN|H
GNC=5241|UniProtKB=P11142,HUMAN|HGNC=8905|UniProtKB=P
36871,HUMAN|HGNC=2678|UniProtKB=P14868,HUMAN|HGNC=1
173|UniProtKB=Q5H9R7,HUMAN|HGNC=3353|UniProtKB=P09104
,HUMAN|HGNC=3214|UniProtKB=P13639,HUMAN|HGNC=5461|U
niProtKB=P78318,HUMAN|HGNC=12628|UniProtKB=P45974,HUM
AN|HGNC=3208|UniProtKB=P24534,HUMAN|HGNC=9551|UniProt
KB=P43686,HUMAN|HGNC=26378|UniProtKB=Q8N7C3,HUMAN|H
GNC=8876|UniProtKB=P17858,HUMAN|HGNC=418|UniProtKB=P0
9972,HUMAN|HGNC=253|UniProtKB=P11766,HUMAN|HGNC=118
|UniProtKB=Q99798,HUMAN|HGNC=30547|UniProtKB=Q96K17,H

<table>
<tr><td>GO:0003677</td><td>UMAN|HGNC=8783|UniProtKB=Q08499,HUMAN|HGNC=20635|UniProtKB=Q14997,HUMAN|HGNC=9559|UniProtKB=Q13200,HUMAN|HGNC=8906|UniProtKB=Q96G03,HUMAN|HGNC=25490|UniProtKB=Q96HA8,HUMAN|HGNC=15912|UniProtKB=Q9UNZ2,HUMAN|HGNC=251|UniProtKB=P00326,HUMAN|HGNC=3354|UniProtKB=P13929,HUMAN|HGNC=15818|UniProtKB=Q9H0Z9,HUMAN|HGNC=5270|UniProtKB=P25685,HUMAN|HGNC=4623|UniProtKB=P00390,HUMAN|HGNC=26951|UniProtKB=Q86YL5,HUMAN|HGNC=4823|UniProtKB=P69905,HUMAN|HGNC=4829|UniProtKB=P02042,HUMAN|HGNC=679|UniProtKB=P52566,HUMAN|HGNC=3465|UniProtKB=P10768,HUMAN|HGNC=4432|UniProtKB=P17174,HUMAN|HGNC=117|UniProtKB=P21399,HUMAN|HGNC=9553|UniProtKB=P62333,HUMAN|HGNC=20076|UniProtKB=Q96K76 HUMAN|HGNC=4747|UniProtKB=P58876,HUMAN|HGNC=4750|UniProtKB=Q99879,HUMAN|HGNC=13954|UniProtKB=O60814,HUMAN|HGNC=4762|UniProtKB=P57053,HUMAN|HGNC=20508|UniProtKB=Q8IUE6,HUMAN|HGNC=4746|UniProtKB=P62807,HUMAN|HGNC=20514|UniProtKB=Q8N257,HUMAN|HGNC=17177|UniProtKB=Q8IUI8,HUMAN|HGNC=4755|UniProtKB=Q93079,HUMAN|HGNC=24700|UniProtKB=Q5QNW6,HUMAN|HGNC=4749|UniProtKB=Q99877</td><td>DNA binding</td></tr>
<tr><td>GO:0043248</td><td>HUMAN|HGNC=9556|UniProtKB=O00231</td><td>proteasome assembly</td></tr>
<tr><td>GO:0070851</td><td>HUMAN|HGNC=19100|UniProtKB=Q5VWK5,HUMAN|HGNC=11283|UniProtKB=P12931</td><td>growth factor receptor binding</td></tr>
<tr><td>PC00095</td><td>HUMAN|HGNC=25842|UniProtKB=Q6UWX4,HUMAN|HGNC=15912|UniProtKB=Q9UNZ2,HUMAN|HGNC=9411|UniProtKB=P14314</td><td>protein-binding activity modulator</td></tr>
<tr><td>GO:0048523</td><td>HUMAN|HGNC=17643|UniProtKB=Q9HD26</td><td>negative regulation of cellular process</td></tr>
<tr><td>GO:0061650</td><td>HUMAN|HGNC=12488|UniProtKB=P68036,HUMAN|HGNC=12491|UniProtKB=P61081,HUMAN|HGNC=12492|UniProtKB=P61088,HUMAN|HGNC=12476|UniProtKB=P61077,HUMAN|HGNC=12475|UniProtKB=P62837,HUMAN|HGNC=12494|UniProtKB=Q13404,HUMAN|HGNC=17598|UniProtKB=Q9Y385</td><td>ubiquitin-like protein conjugating enzyme activity</td></tr>
<tr><td>GO:0030139</td><td>HUMAN|HGNC=9785|UniProtKB=P51148,HUMAN|HGNC=9784|UniProtKB=P61020,HUMAN|HGNC=3244|UniProtKB=Q9NZN3,HUMAN|HGNC=11358|UniProtKB=O75886,HUMAN|HGNC=2092|UniProtKB=Q00610,HUMAN|HGNC=561|UniProtKB=O95782,HUMAN|HGNC=9788|UniProtKB=P51149</td><td>endocytic vesicle</td></tr>
<tr><td>GO:0009067</td><td>HUMAN|HGNC=4432|UniProtKB=P17174,HUMAN|HGNC=7413|UniProtKB=Q13126</td><td>aspartate family amino acid biosynthetic process</td></tr>
<tr><td>GO:0015081</td><td>HUMAN|HGNC=800|UniProtKB=P50993,HUMAN|HGNC=806|UniProtKB=P54709</td><td>sodium ion transmembrane transporter activity</td></tr>
<tr><td>GO:0006355</td><td>HUMAN|HGNC=28843|UniProtKB=Q9HC44,HUMAN|HGNC=7803|UniProtKB=Q12986,HUMAN|HGNC=19246|UniProtKB=Q9NYB0,HUMAN|HGNC=18411|UniProtKB=Q3KNW1,HUMAN|HGNC=29158|UniProtKB=Q9Y2G9,HUMAN|HGNC=12347|UniProtKB=Q9Y4A5,HUMAN|HGNC=25397|UniProtKB=Q6MZP7,HUMAN|HGNC=1101|UniProtKB=P51587,HUMAN|HGNC=10774|UniProtKB=P23246,HUMAN|HGNC=7446|UniProtKB=Q9Y4I5,HUMAN|HGNC=20250|UniProtKB=Q96IF1</td><td>regulation of transcription, DNA-templated</td></tr>
<tr><td>GO:0003735</td><td>HUMAN|HGNC=10372|UniProtKB=P05386,HUMAN|HGNC=10404|UniProtKB=P15880,HUMAN|HGNC=10363|UniProtKB=P18124,HUMAN|HGNC=10385|UniProtKB=P25398,HUMAN|HGNC=10396|Un</td><td>structural constituent of ribosome</td></tr>
</table>

<table>
<tr><td></td><td>iProtKB=P62249,HUMAN|HGNC=10305|UniProtKB=P50914,HUMAN|HGNC=10353|UniProtKB=P36578</td><td></td></tr>
<tr><td>GO:0006302</td><td>HUMAN|HGNC=23077|UniProtKB=Q96FW1,HUMAN|HGNC=20473|UniProtKB=Q9BX63</td><td>double-strand break repair</td></tr>
<tr><td>GO:0008013</td><td>HUMAN|HGNC=23688|UniProtKB=Q86UU0</td><td>beta-catenin binding</td></tr>
<tr><td>GO:0001654</td><td>HUMAN|HGNC=7002|UniProtKB=A6NDR6</td><td>eye development</td></tr>
<tr><td>GO:0008757</td><td>HUMAN|HGNC=23435|UniProtKB=Q8N4J0,HUMAN|HGNC=8728|UniProtKB=P22061,HUMAN|HGNC=26022|UniProtKB=Q7L0Y3</td><td>S-adenosylmethionine-dependent methyltransferase activity</td></tr>
<tr><td>GO:0031346</td><td>HUMAN|HGNC=21295|UniProtKB=Q8TE68</td><td>positive regulation of cell projection organization</td></tr>
<tr><td>PC00133</td><td>HUMAN|HGNC=2062|UniProtKB=O00299,HUMAN|HGNC=13518|UniProtKB=Q9Y696,HUMAN|HGNC=22222|UniProtKB=Q9C0H2</td><td>ion channel</td></tr>
<tr><td>PC00142</td><td>HUMAN|HGNC=4624|UniProtKB=P48637,HUMAN|HGNC=2520|UniProtKB=Q9NRF8,HUMAN|HGNC=871|UniProtKB=Q9UII2,HUMAN|HGNC=4312|UniProtKB=P48507,HUMAN|HGNC=24723|UniProtKB=Q9H6R3,HUMAN|HGNC=1424|UniProtKB=P27708</td><td>ligase</td></tr>
<tr><td>GO:0008015</td><td>HUMAN|HGNC=11828|UniProtKB=Q9UDY2</td><td>blood circulation</td></tr>
<tr><td>GO:0000381</td><td>HUMAN|HGNC=5048|UniProtKB=Q00839</td><td>regulation of alternative mRNA splicing, via spliceosome</td></tr>
<tr><td>GO:0008088</td><td>HUMAN|HGNC=6325|UniProtKB=O60282,HUMAN|HGNC=13206|UniProtKB=O60296</td><td>axo-dendritic transport</td></tr>
<tr><td>GO:0060191</td><td>HUMAN|HGNC=607|UniProtKB=P02654</td><td>regulation of lipase activity</td></tr>
<tr><td>GO:0046470</td><td>HUMAN|HGNC=30244|UniProtKB=Q6P1A2,HUMAN|HGNC=1869|UniProtKB=P11597</td><td>phosphatidylcholine metabolic process</td></tr>
<tr><td>GO:0090305</td><td>HUMAN|HGNC=7326|UniProtKB=P20585,HUMAN|HGNC=23845|UniProtKB=Q8IY92</td><td>nucleic acid phosphodiester bond hydrolysis</td></tr>
<tr><td>GO:0010494</td><td>HUMAN|HGNC=3282|UniProtKB=P60842</td><td>cytoplasmic stress granule</td></tr>
<tr><td>GO:0002009</td><td>HUMAN|HGNC=12652|UniProtKB=P50552,HUMAN|HGNC=25361|UniProtKB=Q8N264</td><td>morphogenesis of an epithelium</td></tr>
<tr><td>GO:0009987</td><td>HUMAN|HGNC=4932|UniProtKB=P01889</td><td>cellular process</td></tr>
<tr><td>GO:0001819</td><td>HUMAN|HGNC=5261|UniProtKB=P10809,HUMAN|HGNC=7183|UniProtKB=P41218,HUMAN|HGNC=533|UniProtKB=P04083,HUMAN|HGNC=1628|UniProtKB=P08571,HUMAN|HGNC=6517|UniProtKB=P18428,HUMAN|HGNC=16961|UniProtKB=Q15762</td><td>positive regulation of cytokine production</td></tr>
<tr><td>GO:0030018</td><td>HUMAN|HGNC=2067|UniProtKB=O00151,HUMAN|HGNC=7618|UniProtKB=O14974,HUMAN|HGNC=17468|UniProtKB=Q96HC4,HUMAN|HGNC=22958|UniProtKB=Q9NR12</td><td>Z disc</td></tr>
<tr><td>GO:0001708</td><td>HUMAN|HGNC=11594|UniProtKB=Q96SF7,HUMAN|HGNC=14010|UniProtKB=Q8IWI9,HUMAN|HGNC=11590|UniProtKB=Q16650</td><td>cell fate specification</td></tr>
<tr><td>GO:0008483</td><td>HUMAN|HGNC=4432|UniProtKB=P17174,HUMAN|HGNC=4242|UniProtKB=O94808,HUMAN|HGNC=4433|UniProtKB=P00505</td><td>transaminase activity</td></tr>
<tr><td>GO:0016477</td><td>HUMAN|HGNC=9862|UniProtKB=P61225,HUMAN|HGNC=6156|UniProtKB=P05106,HUMAN|HGNC=11148|UniProtKB=Q16658,HUMAN|HGNC=6153|UniProtKB=P05556,HUMAN|HGNC=25361|UniProtKB=Q8N264,HUMAN|HGNC=667|UniProtKB=P61586,HUMAN|HGNC=2252|UniProtKB=P31146,HUMAN|HGNC=10661|UniProtKB=</td><td>cell migration</td></tr>
</table>

<table>
<tr><td></td><td>P31431,HUMAN|HGNC=1736|UniProtKB=P60953,HUMAN|HGNC=544|UniProtKB=P08133,HUMAN|HGNC=9065|UniProtKB=P19174,HUMAN|HGNC=15878|UniProtKB=Q9NQ75,HUMAN|HGNC=2254|UniProtKB=Q9ULV4,HUMAN|HGNC=2253|UniProtKB=Q9BR76,HUMAN|HGNC=11881|UniProtKB=P62328,HUMAN|HGNC=708|UniProtKB=O15511</td><td></td></tr>
<tr><td>GO:0000783</td><td>HUMAN|HGNC=19246|UniProtKB=Q9NYB0</td><td>nuclear telomere cap complex</td></tr>
<tr><td>GO:0050806</td><td>HUMAN|HGNC=1406|UniProtKB=Q9Y698,HUMAN|HGNC=15474|UniProtKB=Q9Y566,HUMAN|HGNC=14294|UniProtKB=Q9BYB0</td><td>positive regulation of synaptic transmission</td></tr>
<tr><td>GO:0034067</td><td>HUMAN|HGNC=1908|UniProtKB=Q96RL7,HUMAN|HGNC=23594|UniProtKB=Q709C8</td><td>protein localization to Golgi apparatus</td></tr>
<tr><td>GO:0006464</td><td>HUMAN|HGNC=3531|UniProtKB=P00488,HUMAN|HGNC=25490|UniProtKB=Q96HA8,HUMAN|HGNC=16369|UniProtKB=Q99497,HUMAN|HGNC=3951|UniProtKB=Q16595</td><td>cellular protein modification process</td></tr>
<tr><td>GO:0030674</td><td>HUMAN|HGNC=18587|UniProtKB=O94901,HUMAN|HGNC=11442|UniProtKB=O15400,HUMAN|HGNC=7641|UniProtKB=P54920,HUMAN|HGNC=11429|UniProtKB=O75558,HUMAN|HGNC=12644|UniProtKB=Q15836,HUMAN|HGNC=11131|UniProtKB=O00161,HUMAN|HGNC=555|UniProtKB=O43747,HUMAN|HGNC=12647|UniProtKB=Q9BV40,HUMAN|HGNC=13667|UniProtKB=Q9BXS5,HUMAN|HGNC=561|UniProtKB=O95782,HUMAN|HGNC=11075|UniProtKB=O14745,HUMAN|HGNC=492|UniProtKB=P16157</td><td>protein-macromolecule adaptor activity</td></tr>
<tr><td>GO:0061695</td><td>HUMAN|HGNC=8876|UniProtKB=P17858,HUMAN|HGNC=9465|UniProtKB=P11908,HUMAN|HGNC=8878|UniProtKB=Q01813,HUMAN|HGNC=9462|UniProtKB=P60891</td><td>transferase complex, transferring phosphorus-containing groups</td></tr>
<tr><td>GO:0000079</td><td>HUMAN|HGNC=18673|UniProtKB=Q96JB5</td><td>regulation of cyclin-dependent protein serine/threonine kinase activity</td></tr>
<tr><td>GO:0007266</td><td>HUMAN|HGNC=1736|UniProtKB=P60953,HUMAN|HGNC=3537|UniProtKB=P25116,HUMAN|HGNC=4381|UniProtKB=Q14344,HUMAN|HGNC=10252|UniProtKB=O75116,HUMAN|HGNC=679|UniProtKB=P52566,HUMAN|HGNC=4472|UniProtKB=Q14330,HUMAN|HGNC=21295|UniProtKB=Q8TE68,HUMAN|HGNC=3540|UniProtKB=Q96RI0,HUMAN|HGNC=678|UniProtKB=P52565</td><td>Rho protein signal transduction</td></tr>
<tr><td>PC00176</td><td>HUMAN|HGNC=7687|UniProtKB=O00483,HUMAN|HGNC=3481|UniProtKB=P13804,HUMAN|HGNC=9355|UniProtKB=P30044,HUMAN|HGNC=7421|UniProtKB=P00403,HUMAN|HGNC=2898|UniProtKB=P09622,HUMAN|HGNC=7716|UniProtKB=P49821,HUMAN|HGNC=2753|UniProtKB=Q16698,HUMAN|HGNC=3482|UniProtKB=P38117,HUMAN|HGNC=6984|UniProtKB=P23368,HUMAN|HGNC=4800|UniProtKB=Q99714,HUMAN|HGNC=11179|UniProtKB=P00441,HUMAN|HGNC=11180|UniProtKB=P04179,HUMAN|HGNC=12436|UniProtKB=O43396,HUMAN|HGNC=121|UniProtKB=O15254,HUMAN|HGNC=3542|UniProtKB=P12259,HUMAN|HGNC=119|UniProtKB=Q15067,HUMAN|HGNC=17772|UniProtKB=Q99757</td><td>oxidoreductase</td></tr>
<tr><td>GO:0070534</td><td>HUMAN|HGNC=23077|UniProtKB=Q96FW1,HUMAN|HGNC=12492|UniProtKB=P61088,HUMAN|HGNC=12494|UniProtKB=Q13404,HUMAN|HGNC=16804|UniProtKB=O00308</td><td>protein K63-linked ubiquitination</td></tr>
<tr><td>GO:0019898</td><td>HUMAN|HGNC=1908|UniProtKB=Q96RL7,HUMAN|HGNC=23594|UniProtKB=Q709C8</td><td>extrinsic component of membrane</td></tr>
<tr><td>PC00220</td><td>HUMAN|HGNC=26639|UniProtKB=Q8NBI6,HUMAN|HGNC=30004|UniProtKB=P0DMN0,HUMAN|HGNC=626|UniProtKB=P07741,HU</td><td>transferase</td></tr>
</table>

	MAN|HGNC=8527|UniProtKB=P55809,HUMAN|HGNC=10869|UniProtKB=Q92185,HUMAN|HGNC=12388|UniProtKB=Q16762,HUMAN|HGNC=7223|UniProtKB=P25325,HUMAN|HGNC=4635|UniProtKB=P21266,HUMAN|HGNC=1801|UniProtKB=O95674,HUMAN|HGNC=2422|UniProtKB=O75390,HUMAN|HGNC=11453|UniProtKB=P50225,HUMAN|HGNC=3531|UniProtKB=P00488,HUMAN|HGNC=2911|UniProtKB=P36957,HUMAN|HGNC=4634|UniProtKB=P28161,HUMAN|HGNC=4632|UniProtKB=P09488,HUMAN|HGNC=16906|UniProtKB=Q9Y2Q3,HUMAN|HGNC=4638|UniProtKB=P09211,HUMAN|HGNC=115|UniProtKB=P53396	
GO:0051053	HUMAN|HGNC=5048|UniProtKB=Q00839	negative regulation of DNA metabolic process
GO:0005215	HUMAN|HGNC=4827|UniProtKB=P68871,HUMAN|HGNC=6401|UniProtKB=Q92973,HUMAN|HGNC=2431|UniProtKB=P55060,HUMAN|HGNC=4831|UniProtKB=P69891,HUMAN|HGNC=4823|UniProtKB=P69905,HUMAN|HGNC=4829|UniProtKB=P02042,HUMAN|HGNC=6396|UniProtKB=O00505,HUMAN|HGNC=6400|UniProtKB=Q14974	transporter activity
PC00094	HUMAN|HGNC=20165|UniProtKB=Q9P2P1,HUMAN|HGNC=17904|UniProtKB=Q9NRR4	endoribonuclease
GO:0004866	HUMAN|HGNC=3658|UniProtKB=Q9UGM5,HUMAN|HGNC=11820|UniProtKB=P01033,HUMAN|HGNC=9750|UniProtKB=P20742	endopeptidase inhibitor activity
PC00003	HUMAN|HGNC=55|UniProtKB=O15439,HUMAN|HGNC=14637|UniProtKB=Q86UK0	ATP-binding cassette (ABC) transporter
GO:0031966	HUMAN|HGNC=2973|UniProtKB=O00429,HUMAN|HGNC=2974|UniProtKB=P50570,HUMAN|HGNC=29125|UniProtKB=Q9UQ16	mitochondrial membrane
GO:1990782	HUMAN|HGNC=18233|UniProtKB=Q8NDB2	protein tyrosine kinase binding
GO:0002449	HUMAN|HGNC=5575|UniProtKB=A0A0B4J1V2,HUMAN|HGNC=5645|UniProtKB=A0A0C4DH34,HUMAN|HGNC=5549|UniProtKB=A0A0C4DH31,HUMAN|HGNC=5554|UniProtKB=P01743,HUMAN|HGNC=5480|UniProtKB=P01880,HUMAN|HGNC=5659|UniProtKB=A0A0C4DH38,HUMAN|HGNC=5634|UniProtKB=A0A075B7F0,HUMAN|HGNC=38476|UniProtKB=B9A064,HUMAN|HGNC=5861|UniProtKB=A0M8Q6,HUMAN|HGNC=5647|UniProtKB=A0A087WSY4,HUMAN|HGNC=5563|UniProtKB=A0A075B7D0,HUMAN|HGNC=5856|UniProtKB=P0DOY2,HUMAN|HGNC=2367|UniProtKB=P02741,HUMAN|HGNC=5606|UniProtKB=P01763,HUMAN|HGNC=5576|UniProtKB=P01817,HUMAN|HGNC=5528|UniProtKB=P01861,HUMAN|HGNC=5541|UniProtKB=P01871,HUMAN|HGNC=5585|UniProtKB=A0A0C4DH32,HUMAN|HGNC=5551|UniProtKB=A0A0C4DH33,HUMAN|HGNC=5526|UniProtKB=P01859,HUMAN|HGNC=5657|UniProtKB=P0DP08,HUMAN|Ensembl=ENSG00000282639|UniProtKB=A0A0J9YX35,HUMAN|Gene=HV309_HUMAN|UniProtKB=P01782,HUMAN|HGNC=5654|UniProtKB=P01825,HUMAN|HGNC=5553|UniProtKB=A0A0A0MS14,HUMAN|Gene=HV108_HUMAN|UniProtKB=P0DP01,HUMAN|HGNC=5624|UniProtKB=A0A0B4J1X5,HUMAN|HGNC=5620|UniProtKB=P01780,HUMAN|HGNC=5598|UniProtKB=A0A0C4DH35,HUMAN|HGNC=5580|UniProtKB=P01762,HUMAN|HGNC=5633|UniProtKB=A0A075B7D8,HUMAN|HGNC=5582|UniProtKB=A0A0B4J1V0,HUMAN|HGNC=5478|UniProtKB=P01876,HUMAN|HGNC=5617|UniProtKB=A0A075B6Q5,HUMAN|HGNC=5594|UniProtKB=P0DP03,HUMAN|HGNC=5655|UniProtKB=A0A0C4DH41,HUMAN|HGNC=5636|UniProtKB=A0A075B7B8,HUMAN|HGNC=5586|UniPr	lymphocyte mediated immunity

	otKB=A0A0B4J1V1,HUMAN|HGNC=5581|UniProtKB=P01766,HUMAN|HGNC=5622|UniProtKB=A0A0B4J1Y9,HUMAN|HGNC=5652|UniProtKB=A0A075B6R2,HUMAN|HGNC=5593|UniProtKB=P0DP02,HUMAN|HGNC=5479|UniProtKB=P01877,HUMAN|HGNC=5619|UniProtKB=A0A0C4DH42,HUMAN|HGNC=5596|UniProtKB=P01772,HUMAN|HGNC=5550|UniProtKB=P23083,HUMAN|HGNC=5604|UniProtKB=A0A0B4J1X8,HUMAN|HGNC=5525|UniProtKB=P01857,HUMAN|HGNC=5662|UniProtKB=A0A0B4J1U7,HUMAN|HGNC=16961|UniProtKB=Q15762,HUMAN|HGNC=5601|UniProtKB=A0A0C4DH36,HUMAN|HGNC=5716|UniProtKB=P01834,HUMAN|HGNC=5588|UniProtKB=P01764,HUMAN|HGNC=5857|UniProtKB=P0DOY3,HUMAN|HGNC=5610|UniProtKB=P01767,HUMAN|Ensembl=ENSG00000282150|UniProtKB=A0A0J9YY99,HUMAN|HGNC=5527|UniProtKB=P01860,HUMAN|HGNC=584|UniProtKB=P02743	
GO:0098794	HUMAN|HGNC=9536|UniProtKB=O14818,HUMAN|HGNC=2090|UniProtKB=P09496	postsynapse
GO:0001705	HUMAN|HGNC=2188|UniProtKB=Q99715	ectoderm formation
GO:0005753	HUMAN|HGNC=846|UniProtKB=P56385,HUMAN|HGNC=830|UniProtKB=P06576	mitochondrial proton-transporting ATP synthase complex
GO:0007202	HUMAN|HGNC=3537|UniProtKB=P25116,HUMAN|HGNC=4472|UniProtKB=Q14330,HUMAN|HGNC=4390|UniProtKB=P50148,HUMAN|HGNC=3540|UniProtKB=Q96RI0	activation of phospholipase C activity
GO:0043044	HUMAN|HGNC=17780|UniProtKB=Q562R1	ATP-dependent chromatin remodeling
GO:0016301	HUMAN|HGNC=8898|UniProtKB=P07205,HUMAN|HGNC=24552|UniProtKB=Q3LXA3,HUMAN|HGNC=29035|UniProtKB=O43314,HUMAN|HGNC=9141|UniProtKB=Q15126,HUMAN|HGNC=9021|UniProtKB=P14618,HUMAN|HGNC=24822|UniProtKB=Q9H479,HUMAN|HGNC=2850|UniProtKB=Q9Y6T7	kinase activity
GO:0043484	HUMAN|HGNC=27424|UniProtKB=Q5T481,HUMAN|HGNC=5043|UniProtKB=P31942,HUMAN|HGNC=17894|UniProtKB=Q14966,HUMAN|HGNC=347|UniProtKB=Q09666	regulation of RNA splicing
GO:0017053	HUMAN|HGNC=29989|UniProtKB=Q86YP4	transcription repressor complex
GO:0046033	HUMAN|HGNC=626|UniProtKB=P07741,HUMAN|HGNC=469|UniProtKB=Q01433,HUMAN|HGNC=291|UniProtKB=P30566	AMP metabolic process
GO:1901224	HUMAN|HGNC=1628|UniProtKB=P08571,HUMAN|HGNC=3157|UniProtKB=Q92838	positive regulation of NIK/NF-kappaB signaling
GO:0005925	HUMAN|HGNC=6616|UniProtKB=P48059,HUMAN|HGNC=6156|UniProtKB=P05106,HUMAN|HGNC=13200|UniProtKB=Q15942,HUMAN|HGNC=6153|UniProtKB=P05556,HUMAN|HGNC=6513|UniProtKB=Q14847,HUMAN|HGNC=11845|UniProtKB=Q9Y490,HUMAN|HGNC=25361|UniProtKB=Q8N264,HUMAN|HGNC=15447|UniProtKB=Q9Y4G6,HUMAN|HGNC=14653|UniProtKB=Q9HBI1,HUMAN|HGNC=11767|UniProtKB=O43294,HUMAN|HGNC=1090|UniProtKB=Q03001,HUMAN|HGNC=3338|UniProtKB=Q14247,HUMAN|HGNC=243|UniProtKB=P35611,HUMAN|HGNC=544|UniProtKB=P08133,HUMAN|HGNC=9069|UniProtKB=Q15149,HUMAN|HGNC=6040|UniProtKB=Q13418	focal adhesion
GO:0006624	HUMAN|HGNC=9472|UniProtKB=Q99538	vacuolar protein processing

<table>
<tr><td>GO:0032506</td><td>HUMAN|HGNC=535|UniProtKB=P50995</td><td>cytokinetic process</td></tr>
<tr><td>GO:0004693</td><td>HUMAN|HGNC=24224|UniProtKB=Q9NYV4</td><td>cyclin-dependent protein serine/threonine kinase activity</td></tr>
<tr><td>GO:0042393</td><td>HUMAN|HGNC=29158|UniProtKB=Q9Y2G9,HUMAN|HGNC=20635|UniProtKB=Q14997,HUMAN|HGNC=7133|UniProtKB=O14686,HUMAN|HGNC=10760|UniProtKB=Q01105,HUMAN|HGNC=7640|UniProtKB=Q99733,HUMAN|HGNC=42937|UniProtKB=P0DME0,HUMAN|HGNC=13726|UniProtKB=Q8NEZ4,HUMAN|HGNC=7910|UniProtKB=P06748,HUMAN|HGNC=24358|UniProtKB=Q9H2G4</td><td>histone binding</td></tr>
<tr><td>GO:0007169</td><td>HUMAN|HGNC=11364|UniProtKB=P40763,HUMAN|HGNC=12841|UniProtKB=P07947,HUMAN|HGNC=29884|UniProtKB=Q8TF42,HUMAN|HGNC=6735|UniProtKB=P07948,HUMAN|HGNC=4037|UniProtKB=P06241,HUMAN|HGNC=2209|UniProtKB=P20908,HUMAN|HGNC=1692|UniProtKB=P08962,HUMAN|HGNC=6529|UniProtKB=Q13094,HUMAN|HGNC=19296|UniProtKB=Q9UF33,HUMAN|HGNC=15878|UniProtKB=Q9NQ75,HUMAN|HGNC=10840|UniProtKB=P29353,HUMAN|HGNC=7178|UniProtKB=Q13201,HUMAN|HGNC=6091|UniProtKB=P06213</td><td>transmembrane receptor protein tyrosine kinase signaling pathway</td></tr>
<tr><td>GO:0009055</td><td>HUMAN|HGNC=7687|UniProtKB=O00483,HUMAN|HGNC=7421|UniProtKB=P00403,HUMAN|HGNC=3482|UniProtKB=P38117,HUMAN|HGNC=2267|UniProtKB=P20674</td><td>electron transfer activity</td></tr>
<tr><td>GO:0140096</td><td>HUMAN|HGNC=30167|UniProtKB=P13667,HUMAN|HGNC=30178|UniProtKB=Q9Y570,HUMAN|HGNC=16369|UniProtKB=Q99497,HUMAN|HGNC=28959|UniProtKB=Q14008,HUMAN|HGNC=8548|UniProtKB=P07237,HUMAN|HGNC=8988|UniProtKB=Q13526,HUMAN|HGNC=4606|UniProtKB=P30101,HUMAN|HGNC=3531|UniProtKB=P00488,HUMAN|HGNC=6737|UniProtKB=O75608,HUMAN|HGNC=9756|UniProtKB=O00391,HUMAN|HGNC=14683|UniProtKB=Q9Y2G5,HUMAN|HGNC=26664|UniProtKB=Q8N841,HUMAN|HGNC=8127|UniProtKB=O15294,HUMAN|HGNC=18039|UniProtKB=Q9UGL1</td><td>catalytic activity, acting on a protein</td></tr>
<tr><td>PC00041</td><td>HUMAN|HGNC=20040|UniProtKB=Q01518,HUMAN|HGNC=2067|UniProtKB=O00151,HUMAN|HGNC=9944|UniProtKB=P35241,HUMAN|HGNC=13200|UniProtKB=Q15942,HUMAN|HGNC=12691|UniProtKB=P15311,HUMAN|HGNC=8765|UniProtKB=O75340,HUMAN|HGNC=29827|UniProtKB=O14950,HUMAN|HGNC=11767|UniProtKB=O43294,HUMAN|HGNC=9581|UniProtKB=Q9H939,HUMAN|HGNC=12736|UniProtKB=O43516,HUMAN|HGNC=706|UniProtKB=O15145,HUMAN|HGNC=11873|UniProtKB=Q9NYL9,HUMAN|HGNC=16701|UniProtKB=P19105,HUMAN|HGNC=3382|UniProtKB=Q08495,HUMAN|HGNC=17468|UniProtKB=Q96HC4,HUMAN|HGNC=7373|UniProtKB=P26038,HUMAN|HGNC=704|UniProtKB=O15143,HUMAN|HGNC=15754|UniProtKB=P24844,HUMAN|HGNC=22958|UniProtKB=Q9NR12,HUMAN|HGNC=24636|UniProtKB=Q9UHB6,HUMAN|HGNC=707|UniProtKB=P59998,HUMAN|HGNC=708|UniProtKB=O15511</td><td>actin or actin-binding cytoskeletal protein</td></tr>
<tr><td>GO:0097478</td><td>HUMAN|HGNC=5575|UniProtKB=A0A0B4J1V2,HUMAN|HGNC=5645|UniProtKB=A0A0C4DH34,HUMAN|HGNC=4932|UniProtKB=P01889,HUMAN|HGNC=5549|UniProtKB=A0A0C4DH31,HUMAN|HGNC=5554|UniProtKB=P01743,HUMAN|HGNC=5480|UniProtKB=P01880,HUMAN|HGNC=5659|UniProtKB=A0A0C4DH38,HUMAN|HGNC=5634|UniProtKB=A0A075B7F0,HUMAN|HGNC=12841|UniProtK</td><td>leaflet of membrane bilayer</td></tr>
</table>

	B=P07947,HUMAN|HGNC=11283|UniProtKB=P12931,HUMAN|HGNC=6735|UniProtKB=P07948,HUMAN|HGNC=38476|UniProtKB=B9A064,HUMAN|HGNC=4037|UniProtKB=P06241,HUMAN|HGNC=4393|UniProtKB=P11488,HUMAN|HGNC=5861|UniProtKB=A0M8Q6,HUMAN|HGNC=5647|UniProtKB=A0A087WSY4,HUMAN|HGNC=5563|UniProtKB=A0A075B7D0,HUMAN|HGNC=5856|UniProtKB=P0DOY2,HUMAN|HGNC=4395|UniProtKB=P19086,HUMAN|HGNC=5606|UniProtKB=P01763,HUMAN|HGNC=4384|UniProtKB=P63096,HUMAN|HGNC=199|UniProtKB=O43506,HUMAN|HGNC=5576|UniProtKB=P01817,HUMAN|HGNC=5528|UniProtKB=P01861,HUMAN|HGNC=5541|UniProtKB=P01871,HUMAN|HGNC=4244|UniProtKB=O00451,HUMAN|HGNC=5585|UniProtKB=A0A0C4DH32,HUMAN|HGNC=4243|UniProtKB=P56159,HUMAN|HGNC=5551|UniProtKB=A0A0C4DH33,HUMAN|HGNC=4381|UniProtKB=Q14344,HUMAN|HGNC=5526|UniProtKB=P01859,HUMAN|HGNC=1628|UniProtKB=P08571,HUMAN|HGNC=5657|UniProtKB=P0DP08,HUMAN|Ensembl=ENSG00000282639|UniProtKB=A0A0J9YX35,HUMAN|Gene=HV309_HUMAN|UniProtKB=P01782,HUMAN|HGNC=5654|UniProtKB=P01825,HUMAN|HGNC=5553|UniProtKB=A0A0A0MS14,HUMAN|HGNC=19100|UniProtKB=Q5VWK5,HUMAN|Gene=HV108_HUMAN|UniProtKB=P0DP01,HUMAN|HGNC=5624|UniProtKB=A0A0B4J1X5,HUMAN|HGNC=4387|UniProtKB=P08754,HUMAN|HGNC=5620|UniProtKB=P01780,HUMAN|HGNC=20731|UniProtKB=Q9HAV0,HUMAN|HGNC=5598|UniProtKB=A0A0C4DH35,HUMAN|HGNC=11763|UniProtKB=P02786,HUMAN|HGNC=5580|UniProtKB=P01762,HUMAN|HGNC=5633|UniProtKB=A0A075B7D8,HUMAN|HGNC=5582|UniProtKB=A0A0B4J1V0,HUMAN|HGNC=5478|UniProtKB=P01876,HUMAN|HGNC=5617|UniProtKB=A0A075B6Q5,HUMAN|HGNC=910|UniProtKB=P25311,HUMAN|HGNC=5594|UniProtKB=P0DP03,HUMAN|HGNC=5655|UniProtKB=A0A0C4DH41,HUMAN|HGNC=5636|UniProtKB=A0A075B7B8,HUMAN|HGNC=5586|UniProtKB=A0A0B4J1V1,HUMAN|HGNC=5581|UniProtKB=P01766,HUMAN|HGNC=5622|UniProtKB=A0A0B4J1Y9,HUMAN|HGNC=5652|UniProtKB=A0A075B6R2,HUMAN|HGNC=5593|UniProtKB=P0DP02,HUMAN|HGNC=5479|UniProtKB=P01877,HUMAN|HGNC=5619|UniProtKB=A0A0C4DH42,HUMAN|HGNC=5596|UniProtKB=P01772,HUMAN|HGNC=5550|UniProtKB=P23083,HUMAN|HGNC=5604|UniProtKB=A0A0B4J1X8,HUMAN|HGNC=4398|UniProtKB=P62879,HUMAN|HGNC=5525|UniProtKB=P01857,HUMAN|HGNC=5662|UniProtKB=A0A0B4J1U7,HUMAN|HGNC=4385|UniProtKB=P04899,HUMAN|HGNC=4390|UniProtKB=P50148,HUMAN|HGNC=16961|UniProtKB=Q15762,HUMAN|HGNC=5601|UniProtKB=A0A0C4DH36,HUMAN|HGNC=5716|UniProtKB=P01834,HUMAN|HGNC=5588|UniProtKB=P01764,HUMAN|HGNC=5857|UniProtKB=P0DOY3,HUMAN|HGNC=5610|UniProtKB=P01767,HUMAN|Ensembl=ENSG00000282150|UniProtKB=A0A0J9YY99,HUMAN|HGNC=5527|UniProtKB=P01860,HUMAN|HGNC=4396|UniProtKB=P62873	
GO:0007269	HUMAN|HGNC=11446|UniProtKB=O00186,HUMAN|HGNC=11445|UniProtKB=Q15833	neurotransmitter secretion
GO:0034198	HUMAN|HGNC=18423|UniProtKB=O75140	cellular response to amino acid starvation
GO:0008234	HUMAN|HGNC=25648|UniProtKB=Q8N5J2,HUMAN|HGNC=2479|UniProtKB=O76096,HUMAN|HGNC=6383|UniProtKB=P01042,HU	cysteine-type peptidase activity

<table>
<tr><td></td><td>MAN|HGNC=2482|UniProtKB=P04080,HUMAN|HGNC=4248|UniProtKB=Q92820</td><td></td></tr>
<tr><td>GO:0006401</td><td>HUMAN|HGNC=30646|UniProtKB=Q7KZF4</td><td>RNA catabolic process</td></tr>
<tr><td>GO:0030183</td><td>HUMAN|HGNC=11491|UniProtKB=P43405</td><td>B cell differentiation</td></tr>
<tr><td>GO:0048870</td><td>HUMAN|HGNC=15750|UniProtKB=P60981,HUMAN|HGNC=17780|UniProtKB=Q562R1,HUMAN|HGNC=18271|UniProtKB=Q8N8S7,HUMAN|HGNC=1874|UniProtKB=P23528</td><td>cell motility</td></tr>
<tr><td>GO:0030004</td><td>HUMAN|HGNC=800|UniProtKB=P50993,HUMAN|HGNC=806|UniProtKB=P54709</td><td>cellular monovalent inorganic cation homeostasis</td></tr>
<tr><td>GO:0035249</td><td>HUMAN|HGNC=1406|UniProtKB=Q9Y698,HUMAN|HGNC=7641|UniProtKB=P54920,HUMAN|HGNC=15474|UniProtKB=Q9Y566,HUMAN|HGNC=14294|UniProtKB=Q9BYB0</td><td>synaptic transmission, glutamatergic</td></tr>
<tr><td>GO:0030282</td><td>HUMAN|HGNC=11891|UniProtKB=P05452,HUMAN|HGNC=3153|UniProtKB=Q16610</td><td>bone mineralization</td></tr>
<tr><td>GO:0006888</td><td>HUMAN|HGNC=16695|UniProtKB=P51572,HUMAN|HGNC=20726|UniProtKB=Q8WVM8,HUMAN|HGNC=2230|UniProtKB=P53621,HUMAN|HGNC=21354|UniProtKB=Q86XR7,HUMAN|HGNC=24251|UniProtKB=Q9Y3A6,HUMAN|HGNC=2231|UniProtKB=P53618,HUMAN|HGNC=6631|UniProtKB=P49257,HUMAN|HGNC=16998|UniProtKB=P49755,HUMAN|HGNC=649|UniProtKB=P48444,HUMAN|HGNC=2232|UniProtKB=P35606,HUMAN|HGNC=16996|UniProtKB=Q15363,HUMAN|HGNC=24878|UniProtKB=Q9BVK6</td><td>endoplasmic reticulum to Golgi vesicle-mediated transport</td></tr>
<tr><td>GO:0017076</td><td>HUMAN|HGNC=12407|UniProtKB=P68366,HUMAN|HGNC=654|UniProtKB=P61204,HUMAN|HGNC=9391|UniProtKB=P13861,HUMAN|HGNC=18756|UniProtKB=Q9BYZ6,HUMAN|HGNC=9414|UniProtKB=Q13976,HUMAN|HGNC=9862|UniProtKB=P61225,HUMAN|HGNC=652|UniProtKB=P84077,HUMAN|HGNC=12410|UniProtKB=Q9NY65,HUMAN|HGNC=9380|UniProtKB=P17612,HUMAN|HGNC=20778|UniProtKB=P07437,HUMAN|HGNC=6407|UniProtKB=P01116,HUMAN|HGNC=9392|UniProtKB=P31323,HUMAN|HGNC=20766|UniProtKB=Q71U36,HUMAN|HGNC=667|UniProtKB=P61586,HUMAN|HGNC=4227|UniProtKB=P50395,HUMAN|HGNC=9855|UniProtKB=P62834,HUMAN|HGNC=544|UniProtKB=P08133,HUMAN|HGNC=659|UniProtKB=P62330,HUMAN|HGNC=9840|UniProtKB=P11234,HUMAN|HGNC=9381|UniProtKB=P22694,HUMAN|HGNC=678|UniProtKB=P52565,HUMAN|HGNC=692|UniProtKB=P40616,HUMAN|HGNC=4226|UniProtKB=P31150,HUMAN|HGNC=626|UniProtKB=P07741,HUMAN|HGNC=9801|UniProtKB=P63000,HUMAN|HGNC=669|UniProtKB=P08134,HUMAN|HGNC=9857|UniProtKB=P61224,HUMAN|HGNC=679|UniProtKB=P52566,HUMAN|HGNC=20774|UniProtKB=P04350,HUMAN|HGNC=10447|UniProtKB=P10301,HUMAN|HGNC=11231|UniProtKB=Q8WXF7,HUMAN|HGNC=658|UniProtKB=P84085,HUMAN|HGNC=20771|UniProtKB=P68371,HUMAN|HGNC=655|UniProtKB=P18085,HUMAN|HGNC=24526|UniProtKB=Q6DD88,HUMAN|HGNC=10011|UniProtKB=Q15382,HUMAN|HGNC=9802|UniProtKB=P15153,HUMAN|HGNC=1736|UniProtKB=P60953,HUMAN|HGNC=16257|UniProtKB=Q9H4B7,HUMAN|HGNC=2153|UniProtKB=Q9NQW8,HUMAN|HGNC=9386|UniProtKB=Q9UGJ0,HUMAN|HGNC=4234|UniProtKB=P55040</td><td>purine nucleotide binding</td></tr>
<tr><td>GO:0003824</td><td>HUMAN|HGNC=4312|UniProtKB=P48507</td><td>catalytic activity</td></tr>
</table>

GO:0140352	HUMAN|HGNC=800|UniProtKB=P50993,HUMAN|HGNC=806|UniProtKB=P54709	export from cell
GO:1902554	HUMAN|HGNC=8927|UniProtKB=Q93100	serine/threonine protein kinase complex
PC00248	HUMAN|HGNC=13109|UniProtKB=P17038,HUMAN|HGNC=13061|UniProtKB=Q14587,HUMAN|HGNC=18411|UniProtKB=Q3KNW1,HUMAN|HGNC=23708|UniProtKB=Q6ZR52,HUMAN|HGNC=9349|UniProtKB=Q9NQX1,HUMAN|HGNC=12879|UniProtKB=P21506,HUMAN|HGNC=29416|UniProtKB=Q5T7W0,HUMAN|HGNC=13506|UniProtKB=Q8TBJ5	C2H2 zinc finger transcription factor
GO:0061025	HUMAN|HGNC=2973|UniProtKB=O00429,HUMAN|HGNC=2974|UniProtKB=P50570,HUMAN|HGNC=15912|UniProtKB=Q9UNZ2,HUMAN|HGNC=29125|UniProtKB=Q9UQ16	membrane fusion
PC00158	HUMAN|HGNC=10989|UniProtKB=Q00325,HUMAN|HGNC=19375|UniProtKB=Q9BV35	mitochondrial carrier protein
GO:0032355	HUMAN|HGNC=533|UniProtKB=P04083	response to estradiol
GO:0006706	HUMAN|HGNC=613|UniProtKB=P02649	steroid catabolic process
GO:0060548	HUMAN|HGNC=28771|UniProtKB=Q9BVC6	negative regulation of cell death
GO:0044260	HUMAN|HGNC=25153|UniProtKB=Q96EY5	cellular macromolecule metabolic process
GO:0005178	HUMAN|HGNC=23151|UniProtKB=Q86UX7,HUMAN|HGNC=3778|UniProtKB=P02751,HUMAN|HGNC=6156|UniProtKB=P05106,HUMAN|HGNC=6153|UniProtKB=P05556,HUMAN|HGNC=11845|UniProtKB=Q9Y490,HUMAN|HGNC=15447|UniProtKB=Q9Y4G6	integrin binding
PC00228	HUMAN|HGNC=12407|UniProtKB=P68366,HUMAN|HGNC=12410|UniProtKB=Q9NY65,HUMAN|HGNC=20778|UniProtKB=P07437,HUMAN|HGNC=16257|UniProtKB=Q9H4B7,HUMAN|HGNC=20766|UniProtKB=Q71U36,HUMAN|HGNC=20774|UniProtKB=P04350,HUMAN|HGNC=20771|UniProtKB=P68371	tubulin
GO:0006399	HUMAN|HGNC=24728|UniProtKB=A2RTX5,HUMAN|HGNC=2678|UniProtKB=P14868,HUMAN|HGNC=4162|UniProtKB=P41250,HUMAN|HGNC=11572|UniProtKB=P26639,HUMAN|HGNC=16219|UniProtKB=Q8TEA8,HUMAN|HGNC=12729|UniProtKB=P23381	tRNA metabolic process
GO:0050789	HUMAN|HGNC=1869|UniProtKB=P11597,HUMAN|HGNC=9093|UniProtKB=P55058	regulation of biological process
GO:0008168	HUMAN|HGNC=24550|UniProtKB=Q9H8H3	methyltransferase activity
GO:0005244	HUMAN|HGNC=12669|UniProtKB=P21796,HUMAN|HGNC=12674|UniProtKB=Q9Y277,HUMAN|HGNC=12672|UniProtKB=P45880	voltage-gated ion channel activity
GO:0005656	HUMAN|HGNC=26200|UniProtKB=Q9H668	nuclear pre-replicative complex
GO:0022843	HUMAN|HGNC=16768|UniProtKB=O60391	voltage-gated cation channel activity
GO:0005261	HUMAN|HGNC=8533|UniProtKB=P51575,HUMAN|HGNC=2153|UniProtKB=Q9NQW8,HUMAN|HGNC=830|UniProtKB=P06576,HUMAN|HGNC=850|UniProtKB=P48047	cation channel activity
GO:0006091	HUMAN|HGNC=253|UniProtKB=P11766,HUMAN|HGNC=7863|UniProtKB=Q13423,HUMAN|HGNC=11834|UniProtKB=P29401,HUMAN|HGNC=251|UniProtKB=P00326,HUMAN|HGNC=8891|UniProtKB=P52209,HUMAN|HGNC=8903|UniProtKB=O95336,HUMAN|HGNC=255|UniProtKB=P28332	generation of precursor metabolites and energy

GO:0015276	HUMAN|HGNC=8533|UniProtKB=P51575,HUMAN|HGNC=544|UniProtKB=P08133,HUMAN|HGNC=2153|UniProtKB=Q9NQW8,HUMAN|HGNC=16768|UniProtKB=O60391	ligand-gated ion channel activity
GO:0032210	HUMAN|HGNC=5048|UniProtKB=Q00839	regulation of telomere maintenance via telomerase
GO:0016607	HUMAN|HGNC=5048|UniProtKB=Q00839	nuclear speck
GO:0004722	HUMAN|HGNC=19388|UniProtKB=P49593,HUMAN|HGNC=9302|UniProtKB=P30153,HUMAN|HGNC=9314|UniProtKB=Q08209,HUMAN|HGNC=9281|UniProtKB=P62136	protein serine/threonine phosphatase activity
GO:0042035	HUMAN|HGNC=6517|UniProtKB=P18428	regulation of cytokine biosynthetic process
GO:0016667	HUMAN|HGNC=2898|UniProtKB=P09622,HUMAN|HGNC=28477|UniProtKB=Q8NBM8,HUMAN|HGNC=9756|UniProtKB=O00391,HUMAN|HGNC=7377|UniProtKB=Q9UJ68	oxidoreductase activity, acting on a sulfur group of donors
GO:1901566	HUMAN|HGNC=30244|UniProtKB=Q6P1A2,HUMAN|HGNC=2689|UniProtKB=P09172	organonitrogen compound biosynthetic process
GO:0008630	HUMAN|HGNC=992|UniProtKB=Q07817	intrinsic apoptotic signaling pathway in response to DNA damage
GO:0031122	HUMAN|HGNC=1090|UniProtKB=Q03001	cytoplasmic microtubule organization
GO:0030686	HUMAN|HGNC=12755|UniProtKB=Q9UNX4	90S preribosome
GO:0006006	HUMAN|HGNC=8898|UniProtKB=P07205,HUMAN|HGNC=8876|UniProtKB=P17858,HUMAN|HGNC=4458|UniProtKB=P06744,HUMAN|HGNC=12009|UniProtKB=P60174,HUMAN|HGNC=8878|UniProtKB=Q01813,HUMAN|HGNC=18985|UniProtKB=Q7Z4W1	glucose metabolic process
GO:1901137	HUMAN|HGNC=12009|UniProtKB=P60174	carbohydrate derivative biosynthetic process
GO:0017112	HUMAN|HGNC=24748|UniProtKB=Q68D51,HUMAN|HGNC=6766|UniProtKB=Q8WXG6,HUMAN|HGNC=10542|UniProtKB=O95248	Rab guanyl-nucleotide exchange factor activity
GO:0061462	HUMAN|HGNC=1406|UniProtKB=Q9Y698	protein localization to lysosome
GO:0090407	HUMAN|HGNC=29035|UniProtKB=O43314,HUMAN|HGNC=12009|UniProtKB=P60174	organophosphate biosynthetic process
GO:0009060	HUMAN|HGNC=12582|UniProtKB=P14927,HUMAN|HGNC=12590|UniProtKB=P07919	aerobic respiration
GO:0035235	HUMAN|HGNC=1406|UniProtKB=Q9Y698,HUMAN|HGNC=15474|UniProtKB=Q9Y566,HUMAN|HGNC=16768|UniProtKB=O60391,HUMAN|HGNC=14294|UniProtKB=Q9BYB0	ionotropic glutamate receptor signaling pathway
GO:0005777	HUMAN|HGNC=11179|UniProtKB=P00441,HUMAN|HGNC=5382|UniProtKB=O75874,HUMAN|HGNC=121|UniProtKB=O15254,HUMAN|HGNC=119|UniProtKB=Q15067	peroxisome
GO:0072376	HUMAN|HGNC=5575|UniProtKB=A0A0B4J1V2,HUMAN|HGNC=5645|UniProtKB=A0A0C4DH34,HUMAN|HGNC=5549|UniProtKB=A0A0C4DH31,HUMAN|HGNC=5554|UniProtKB=P01743,HUMAN|HGNC=5480|UniProtKB=P01880,HUMAN|HGNC=5659|UniProtKB=A0A0C4DH38,HUMAN|HGNC=5634|UniProtKB=A0A075B7F0,HUMAN|HGNC=38476|UniProtKB=B9A064,HUMAN|HGNC=1324|UniProtKB=P0C0L5,HUMAN|HGNC=5861|UniProtKB=A0M8Q6,HUMAN|HGNC=5647|UniProtKB=A0A087WSY4,HUMAN|HGNC=5563|UniProtKB=A0A075B7D0,HUMAN|HGNC=5856|UniProtKB=P0DOY2,HUMAN|HGNC=2367|UniProtKB=P02741,HUMAN|HGNC=5606|UniProtK	protein activation cascade

	B=P01763,HUMAN|HGNC=5576|UniProtKB=P01817,HUMAN|HGNC=5528|UniProtKB=P01861,HUMAN|HGNC=5541|UniProtKB=P01871,HUMAN|HGNC=5585|UniProtKB=A0A0C4DH32,HUMAN|HGNC=5551|UniProtKB=A0A0C4DH33,HUMAN|HGNC=5526|UniProtKB=P01859,HUMAN|HGNC=5657|UniProtKB=P0DP08,HUMAN|Ensembl=ENSG00000282639|UniProtKB=A0A0J9YX35,HUMAN|Gene=HV309_HUMAN|UniProtKB=P01782,HUMAN|HGNC=5654|UniProtKB=P01825,HUMAN|HGNC=5553|UniProtKB=A0A0A0MS14,HUMAN|Gene=HV108_HUMAN|UniProtKB=P0DP01,HUMAN|HGNC=5624|UniProtKB=A0A0B4J1X5,HUMAN|HGNC=5620|UniProtKB=P01780,HUMAN|HGNC=5598|UniProtKB=A0A0C4DH35,HUMAN|HGNC=5580|UniProtKB=P01762,HUMAN|HGNC=5633|UniProtKB=A0A075B7D8,HUMAN|HGNC=5582|UniProtKB=A0A0B4J1V0,HUMAN|HGNC=5478|UniProtKB=P01876,HUMAN|HGNC=5617|UniProtKB=A0A075B6Q5,HUMAN|HGNC=5594|UniProtKB=P0DP03,HUMAN|HGNC=5655|UniProtKB=A0A0C4DH41,HUMAN|HGNC=5636|UniProtKB=A0A075B7B8,HUMAN|HGNC=5586|UniProtKB=A0A0B4J1V1,HUMAN|HGNC=5581|UniProtKB=P01766,HUMAN|HGNC=5622|UniProtKB=A0A0B4J1Y9,HUMAN|HGNC=5652|UniProtKB=A0A075B6R2,HUMAN|HGNC=5593|UniProtKB=P0DP02,HUMAN|HGNC=5479|UniProtKB=P01877,HUMAN|HGNC=5619|UniProtKB=A0A0C4DH42,HUMAN|HGNC=5596|UniProtKB=P01772,HUMAN|HGNC=3662|UniProtKB=P02675,HUMAN|HGNC=5550|UniProtKB=P23083,HUMAN|HGNC=5604|UniProtKB=A0A0B4J1X8,HUMAN|HGNC=5525|UniProtKB=P01857,HUMAN|HGNC=5662|UniProtKB=A0A0B4J1U7,HUMAN|HGNC=5601|UniProtKB=A0A0C4DH36,HUMAN|HGNC=5716|UniProtKB=P01834,HUMAN|HGNC=5588|UniProtKB=P01764,HUMAN|HGNC=5857|UniProtKB=P0DOY3,HUMAN|HGNC=3531|UniProtKB=P00488,HUMAN|HGNC=5610|UniProtKB=P01767,HUMAN|Ensembl=ENSG00000282150|UniProtKB=A0A0J9YY99,HUMAN|HGNC=5527|UniProtKB=P01860,HUMAN|HGNC=584|UniProtKB=P02743	
GO:0090092	HUMAN|HGNC=11766|UniProtKB=P01137	regulation of transmembrane receptor protein serine/threonine kinase signaling pathway
GO:0032970	HUMAN|HGNC=21230|UniProtKB=Q9P1F3	regulation of actin filament-based process
GO:0050801	HUMAN|HGNC=1869|UniProtKB=P11597,HUMAN|HGNC=10582|UniProtKB=Q9Y5Y9	ion homeostasis
GO:0000122	HUMAN|HGNC=9866|UniProtKB=P13631,HUMAN|HGNC=6767|UniProtKB=Q15797,HUMAN|HGNC=29989|UniProtKB=Q86YP4,HUMAN|HGNC=7967|UniProtKB=Q96RI1,HUMAN|HGNC=5048|UniProtKB=Q00839,HUMAN|HGNC=12879|UniProtKB=P21506,HUMAN|HGNC=16804|UniProtKB=O00308	negative regulation of transcription by RNA polymerase II
GO:0033218	HUMAN|HGNC=9093|UniProtKB=P55058	amide binding
GO:0007091	HUMAN|HGNC=15713|UniProtKB=Q9UJX4,HUMAN|HGNC=12401|UniProtKB=P33981,HUMAN|HGNC=14452|UniProtKB=Q9NYG5	metaphase/anaphase transition of mitotic cell cycle
GO:0048856	HUMAN|HGNC=11219|UniProtKB=P09486	anatomical structure development
GO:0022853	HUMAN|HGNC=800|UniProtKB=P50993,HUMAN|HGNC=813|UniProtKB=Q93084,HUMAN|HGNC=851|UniProtKB=P38606,HUMAN|H	active ion transmembrane transporter activity

<table>
<tr><th></th><th></th><th></th></tr>
<tr><td></td><td>GNC=806|UniProtKB=P54709,HUMAN|HGNC=812|UniProtKB=P16615</td><td></td></tr>
<tr><td>GO:0019882</td><td>HUMAN|HGNC=4932|UniProtKB=P01889</td><td>antigen processing and presentation</td></tr>
<tr><td>GO:0051017</td><td>HUMAN|HGNC=6528|UniProtKB=P13796,HUMAN|HGNC=27937|UniProtKB=Q6ZVH7,HUMAN|HGNC=11148|UniProtKB=Q16658,HUMAN|HGNC=3382|UniProtKB=Q08495,HUMAN|HGNC=8881|UniProtKB=P07737,HUMAN|HGNC=243|UniProtKB=P35611,HUMAN|HGNC=24636|UniProtKB=Q9UHB6</td><td>actin filament bundle assembly</td></tr>
<tr><td>GO:0098978</td><td>HUMAN|HGNC=1117|UniProtKB=Q9UPA5,HUMAN|HGNC=2905|UniProtKB=O14490,HUMAN|HGNC=13406|UniProtKB=Q9Y6V0</td><td>glutamatergic synapse</td></tr>
<tr><td>GO:1901136</td><td>HUMAN|HGNC=4456|UniProtKB=P43304</td><td>carbohydrate derivative catabolic process</td></tr>
<tr><td>GO:0044419</td><td>HUMAN|HGNC=16276|UniProtKB=Q9C035,HUMAN|HGNC=25153|UniProtKB=Q96EY5</td><td>interspecies interaction between organisms</td></tr>
<tr><td>GO:0030016</td><td>HUMAN|HGNC=9621|UniProtKB=Q6IBS0,HUMAN|HGNC=347|UniProtKB=Q09666</td><td>myofibril</td></tr>
<tr><td>PC00040</td><td>HUMAN|HGNC=29585|UniProtKB=Q8N6V9,HUMAN|HGNC=12010|UniProtKB=P09493,HUMAN|HGNC=12012|UniProtKB=P06753,HUMAN|HGNC=12013|UniProtKB=P67936,HUMAN|HGNC=12011|UniProtKB=P07951</td><td>actin binding motor protein</td></tr>
<tr><td>GO:0017046</td><td>HUMAN|HGNC=10608|UniProtKB=P47872,HUMAN|HGNC=6091|UniProtKB=P06213</td><td>peptide hormone binding</td></tr>
<tr><td>GO:0043025</td><td>HUMAN|HGNC=5253|UniProtKB=P07900,HUMAN|HGNC=6835|UniProtKB=P78559,HUMAN|HGNC=11138|UniProtKB=P37840</td><td>neuronal cell body</td></tr>
<tr><td>GO:0006970</td><td>HUMAN|HGNC=21696|UniProtKB=Q9Y3Q8</td><td>response to osmotic stress</td></tr>
<tr><td>GO:0043149</td><td>HUMAN|HGNC=667|UniProtKB=P61586</td><td>stress fiber assembly</td></tr>
<tr><td>GO:0048489</td><td>HUMAN|HGNC=6325|UniProtKB=O60282,HUMAN|HGNC=2974|UniProtKB=P50570,HUMAN|HGNC=29125|UniProtKB=Q9UQ16</td><td>synaptic vesicle transport</td></tr>
<tr><td>GO:0005262</td><td>HUMAN|HGNC=23526|UniProtKB=Q8NE86,HUMAN|HGNC=544|UniProtKB=P08133</td><td>calcium channel activity</td></tr>
<tr><td>GO:0060998</td><td>HUMAN|HGNC=3338|UniProtKB=Q14247,HUMAN|HGNC=15474|UniProtKB=Q9Y566,HUMAN|HGNC=2695|UniProtKB=Q16643,HUMAN|HGNC=2696|UniProtKB=Q9UJU6,HUMAN|HGNC=4844|UniProtKB=P14317,HUMAN|HGNC=14294|UniProtKB=Q9BYB0</td><td>regulation of dendritic spine development</td></tr>
<tr><td>GO:0045910</td><td>HUMAN|HGNC=4716|UniProtKB=P16403,HUMAN|HGNC=4718|UniProtKB=P10412,HUMAN|HGNC=4719|UniProtKB=P16401</td><td>negative regulation of DNA recombination</td></tr>
<tr><td>GO:0046928</td><td>HUMAN|HGNC=9855|UniProtKB=P62834,HUMAN|HGNC=7641|UniProtKB=P54920,HUMAN|HGNC=9857|UniProtKB=P61224</td><td>regulation of neurotransmitter secretion</td></tr>
<tr><td>GO:0035567</td><td>HUMAN|HGNC=4045|UniProtKB=O75084,HUMAN|HGNC=11119|UniProtKB=Q99835</td><td>non-canonical Wnt signaling pathway</td></tr>
<tr><td>GO:0035329</td><td>HUMAN|HGNC=20250|UniProtKB=Q96IF1</td><td>hippo signaling</td></tr>
<tr><td>PC00126</td><td>HUMAN|HGNC=6137|UniProtKB=P17301,HUMAN|HGNC=6156|UniProtKB=P05106,HUMAN|HGNC=6138|UniProtKB=P08514,HUMAN|HGNC=6153|UniProtKB=P05556,HUMAN|HGNC=6149|UniProtKB=P11215,HUMAN|HGNC=6142|UniProtKB=P23229</td><td>integrin</td></tr>
<tr><td>GO:0030246</td><td>HUMAN|HGNC=8876|UniProtKB=P17858,HUMAN|HGNC=8601|UniProtKB=Q06141,HUMAN|HGNC=4458|UniProtKB=P06744,HUMAN|HGNC=6561|UniProtKB=P09382,HUMAN|HGNC=8878|UniProtKB=Q01813,HUMAN|HGNC=6631|UniProtKB=P49257</td><td>carbohydrate binding</td></tr>
</table>

<table>
<tr><td>GO:0097526</td><td>HUMAN|HGNC=17259|UniProtKB=Q9Y4Z0</td><td>spliceosomal tri-snRNP complex</td></tr>
<tr><td>GO:0000049</td><td>HUMAN|HGNC=26022|UniProtKB=Q7L0Y3,HUMAN|Gene=EIF2S3L|UniProtKB=Q2VIR3</td><td>tRNA binding</td></tr>
<tr><td>GO:0042803</td><td>HUMAN|HGNC=29943|UniProtKB=Q9NT68,HUMAN|HGNC=992|UniProtKB=Q07817,HUMAN|HGNC=16276|UniProtKB=Q9C035</td><td>protein homodimerization activity</td></tr>
<tr><td>GO:0002440</td><td>HUMAN|HGNC=16961|UniProtKB=Q15762</td><td>production of molecular mediator of immune response</td></tr>
<tr><td>GO:0015103</td><td>HUMAN|HGNC=12669|UniProtKB=P21796,HUMAN|HGNC=12674|UniProtKB=Q9Y277,HUMAN|HGNC=12672|UniProtKB=P45880</td><td>inorganic anion transmembrane transporter activity</td></tr>
<tr><td>GO:1990266</td><td>HUMAN|HGNC=9240|UniProtKB=P02775,HUMAN|HGNC=10632|UniProtKB=P13501,HUMAN|HGNC=8862|UniProtKB=P10720,HUMAN|HGNC=8861|UniProtKB=P02776,HUMAN|HGNC=29953|UniProtKB=Q8WWZ8</td><td>neutrophil migration</td></tr>
<tr><td>GO:0022804</td><td>HUMAN|HGNC=55|UniProtKB=O15439,HUMAN|HGNC=14637|UniProtKB=Q86UK0</td><td>active transmembrane transporter activity</td></tr>
<tr><td>GO:0099568</td><td>HUMAN|HGNC=1117|UniProtKB=Q9UPA5,HUMAN|HGNC=2090|UniProtKB=P09496,HUMAN|HGNC=13406|UniProtKB=Q9Y6V0</td><td>cytoplasmic region</td></tr>
<tr><td>GO:0030198</td><td>HUMAN|HGNC=5171|UniProtKB=P02790,HUMAN|HGNC=7176|UniProtKB=P14780,HUMAN|HGNC=3602|UniProtKB=Q9UBX5,HUMAN|HGNC=11771|UniProtKB=Q15582,HUMAN|HGNC=15855|UniProtKB=Q9NPY3</td><td>extracellular matrix organization</td></tr>
<tr><td>GO:0005739</td><td>HUMAN|HGNC=7694|UniProtKB=O14561,HUMAN|HGNC=18344|UniProtKB=Q9BX68,HUMAN|HGNC=118|UniProtKB=Q99798,HUMAN|HGNC=16753|UniProtKB=P30041,HUMAN|HGNC=17865|UniProtKB=Q96A26,HUMAN|HGNC=5382|UniProtKB=O75874,HUMAN|HGNC=20|UniProtKB=P49588,HUMAN|HGNC=2328|UniProtKB=P50416,HUMAN|HGNC=4553|UniProtKB=P07203,HUMAN|HGNC=4433|UniProtKB=P00505,HUMAN|HGNC=363|UniProtKB=P27144,HUMAN|HGNC=3951|UniProtKB=Q16595,HUMAN|HGNC=2873|UniProtKB=P00387,HUMAN|HGNC=24808|UniProtKB=O14545,HUMAN|HGNC=12585|UniProtKB=P31930,HUMAN|HGNC=6984|UniProtKB=P23368,HUMAN|HGNC=12388|UniProtKB=Q16762,HUMAN|HGNC=3151|UniProtKB=P30084,HUMAN|HGNC=13397|UniProtKB=Q9UHQ9,HUMAN|HGNC=11572|UniProtKB=P26639,HUMAN|HGNC=29890|UniProtKB=Q86UT6,HUMAN|HGNC=13206|UniProtKB=O60296,HUMAN|HGNC=9772|UniProtKB=Q13637,HUMAN|HGNC=28883|UniProtKB=Q9H2U2,HUMAN|HGNC=1243|UniProtKB=Q07021,HUMAN|HGNC=8527|UniProtKB=P55809,HUMAN|HGNC=94|UniProtKB=Q9BWD1,HUMAN|HGNC=17376|UniProtKB=Q9UIJ7,HUMAN|HGNC=4335|UniProtKB=P00367,HUMAN|HGNC=7223|UniProtKB=P25325,HUMAN|HGNC=24728|UniProtKB=A2RTX5,HUMAN|HGNC=11179|UniProtKB=P00441,HUMAN|HGNC=4922|UniProtKB=P19367,HUMAN|HGNC=1050|UniProtKB=P55957,HUMAN|HGNC=5381|UniProtKB=P14735,HUMAN|HGNC=117|UniProtKB=P21399,HUMAN|HGNC=871|UniProtKB=Q9UII2,HUMAN|HGNC=362|UniProtKB=P54819,HUMAN|HGNC=4456|UniProtKB=P43304,HUMAN|HGNC=92|UniProtKB=P49748,HUMAN|HGNC=5386|UniProtKB=P51553</td><td>mitochondrion</td></tr>
<tr><td>PC00096</td><td>HUMAN|HGNC=4116|UniProtKB=Q14376,HUMAN|HGNC=9959|UniProtKB=P51606</td><td>epimerase/racemase</td></tr>
</table>

GO:0005604	HUMAN|HGNC=2209|UniProtKB=P20908,HUMAN|HGNC=12601|UniProtKB=O75445	basement membrane
GO:0070555	HUMAN|HGNC=533|UniProtKB=P04083,HUMAN|HGNC=10632|UniProtKB=P13501	response to interleukin-1
GO:0044283	HUMAN|HGNC=8898|UniProtKB=P07205,HUMAN|HGNC=4458|UniProtKB=P06744,HUMAN|HGNC=12009|UniProtKB=P60174	small molecule biosynthetic process
GO:0005977	HUMAN|HGNC=9723|UniProtKB=P11216,HUMAN|HGNC=9725|UniProtKB=P06737	glycogen metabolic process
GO:0061134	HUMAN|HGNC=20635|UniProtKB=Q14997	peptidase regulator activity
GO:0070979	HUMAN|HGNC=12488|UniProtKB=P68036,HUMAN|HGNC=15713|UniProtKB=Q9UJX4	protein K11-linked ubiquitination
GO:0055088	HUMAN|HGNC=121|UniProtKB=O15254,HUMAN|HGNC=119|UniProtKB=Q15067	lipid homeostasis
GO:0046475	HUMAN|HGNC=13921|UniProtKB=O95870	glycerophospholipid catabolic process
GO:0016021	HUMAN|HGNC=28450|UniProtKB=Q8NC44,HUMAN|HGNC=4045|UniProtKB=O75084,HUMAN|HGNC=11442|UniProtKB=O15400,HUMAN|HGNC=11429|UniProtKB=O75558,HUMAN|HGNC=19176|UniProtKB=Q96DZ9,HUMAN|HGNC=1628|UniProtKB=P08571,HUMAN|HGNC=11119|UniProtKB=Q99835,HUMAN|HGNC=9087|UniProtKB=Q04941	integral component of membrane
GO:0005788	HUMAN|Gene=SEP15|UniProtKB=O60613,HUMAN|HGNC=5238|UniProtKB=P11021	endoplasmic reticulum lumen
GO:0070997	HUMAN|HGNC=11138|UniProtKB=P37840	neuron death
GO:0048468	HUMAN|HGNC=14685|UniProtKB=Q9Y624,HUMAN|HGNC=243|UniProtKB=P35611,HUMAN|HGNC=11828|UniProtKB=Q9UDY2,HUMAN|HGNC=11283|UniProtKB=P12931	cell development
GO:0030239	HUMAN|HGNC=11873|UniProtKB=Q9NYL9,HUMAN|HGNC=3750|UniProtKB=Q13045	myofibril assembly
GO:0043269	HUMAN|HGNC=11510|UniProtKB=Q8N9I0,HUMAN|HGNC=11512|UniProtKB=Q9H2B2	regulation of ion transport
GO:0007188	HUMAN|HGNC=4395|UniProtKB=P19086,HUMAN|HGNC=4384|UniProtKB=P63096,HUMAN|HGNC=10608|UniProtKB=P47872,HUMAN|HGNC=4387|UniProtKB=P08754,HUMAN|HGNC=4385|UniProtKB=P04899,HUMAN|HGNC=4393|UniProtKB=P11488,HUMAN|HGNC=4390|UniProtKB=P50148	adenylate cyclase-modulating G protein-coupled receptor signaling pathway
GO:0019933	HUMAN|HGNC=9391|UniProtKB=P13861,HUMAN|HGNC=4395|UniProtKB=P19086,HUMAN|HGNC=20040|UniProtKB=Q01518,HUMAN|HGNC=4384|UniProtKB=P63096,HUMAN|HGNC=10608|UniProtKB=P47872,HUMAN|HGNC=4387|UniProtKB=P08754,HUMAN|HGNC=9392|UniProtKB=P31323,HUMAN|HGNC=4385|UniProtKB=P04899,HUMAN|HGNC=4393|UniProtKB=P11488,HUMAN|HGNC=4390|UniProtKB=P50148	cAMP-mediated signaling
PC00047	HUMAN|HGNC=24728|UniProtKB=A2RTX5,HUMAN|HGNC=2678|UniProtKB=P14868,HUMAN|HGNC=20|UniProtKB=P49588,HUMAN|HGNC=4162|UniProtKB=P41250,HUMAN|HGNC=11572|UniProtKB=P26639,HUMAN|HGNC=12729|UniProtKB=P23381	aminoacyl-tRNA synthetase
GO:0015370	HUMAN|HGNC=11030|UniProtKB=Q9Y6R1,HUMAN|HGNC=11034|UniProtKB=Q2Y0W8	solute:sodium symporter activity
GO:0007586	HUMAN|HGNC=14685|UniProtKB=Q9Y624,HUMAN|HGNC=11756|UniProtKB=Q03403	digestion

GO:0032418	HUMAN|HGNC=11491|UniProtKB=P43405	lysosome localization
GO:0031490	HUMAN|HGNC=17780|UniProtKB=Q562R1,HUMAN|HGNC=29158|UniProtKB=Q9Y2G9,HUMAN|HGNC=4716|UniProtKB=P16403,HUMAN|HGNC=4718|UniProtKB=P10412,HUMAN|HGNC=12738|UniProtKB=Q969T9,HUMAN|HGNC=4719|UniProtKB=P16401	chromatin DNA binding
GO:0032438	HUMAN|HGNC=9772|UniProtKB=Q13637	melanosome organization
GO:0016810	HUMAN|HGNC=794|UniProtKB=P31939,HUMAN|HGNC=226|UniProtKB=P78563,HUMAN|HGNC=469|UniProtKB=Q01433	hydrolase activity, acting on carbon-nitrogen (but not peptide) bonds
GO:0016052	HUMAN|HGNC=4116|UniProtKB=Q14376	carbohydrate catabolic process
GO:0007219	HUMAN|HGNC=188|UniProtKB=O14672,HUMAN|HGNC=16272|UniProtKB=Q96JK9	Notch signaling pathway
GO:1905349	HUMAN|HGNC=15476|UniProtKB=P63167,HUMAN|HGNC=15848|UniProtKB=Q14141,HUMAN|HGNC=16053|UniProtKB=Q8N1V2,HUMAN|HGNC=20908|UniProtKB=Q86YF9,HUMAN|HGNC=2952|UniProtKB=Q96JB1,HUMAN|HGNC=25631|UniProtKB=Q96MT7,HUMAN|HGNC=1717|UniProtKB=Q16181,HUMAN|HGNC=3244|UniProtKB=Q9NZN3,HUMAN|HGNC=29262|UniProtKB=Q9P2H3,HUMAN|HGNC=7729|UniProtKB=Q15019	ciliary transition zone assembly
GO:0006875	HUMAN|HGNC=800|UniProtKB=P50993,HUMAN|HGNC=806|UniProtKB=P54709	cellular metal ion homeostasis
GO:0052689	HUMAN|HGNC=30178|UniProtKB=Q9Y570,HUMAN|HGNC=20|UniProtKB=P49588,HUMAN|HGNC=6737|UniProtKB=O75608,HUMAN|HGNC=16219|UniProtKB=Q8TEA8,HUMAN|HGNC=8903|UniProtKB=O95336	carboxylic ester hydrolase activity
GO:0044248	HUMAN|HGNC=17169|UniProtKB=Q13162,HUMAN|HGNC=253|UniProtKB=P11766,HUMAN|HGNC=4555|UniProtKB=P22352,HUMAN|HGNC=9355|UniProtKB=P30044,HUMAN|HGNC=4553|UniProtKB=P07203,HUMAN|HGNC=4823|UniProtKB=P69905,HUMAN|HGNC=4829|UniProtKB=P02042,HUMAN|HGNC=9352|UniProtKB=Q06830,HUMAN|HGNC=25153|UniProtKB=Q96EY5,HUMAN|HGNC=4827|UniProtKB=P68871,HUMAN|HGNC=7900|UniProtKB=P55786,HUMAN|HGNC=11820|UniProtKB=P01033,HUMAN|HGNC=9353|UniProtKB=P32119,HUMAN|HGNC=4831|UniProtKB=P69891,HUMAN|HGNC=1516|UniProtKB=P04040,HUMAN|HGNC=13420|UniProtKB=Q86TP1,HUMAN|HGNC=251|UniProtKB=P00326,HUMAN|HGNC=18173|UniProtKB=Q9NZ08,HUMAN|HGNC=255|UniProtKB=P28332	cellular catabolic process
GO:0005096	HUMAN|HGNC=2720|UniProtKB=Q9ULH1,HUMAN|HGNC=6111|UniProtKB=Q13576,HUMAN|HGNC=25361|UniProtKB=Q8N264,HUMAN|HGNC=17102|UniProtKB=Q92619,HUMAN|HGNC=4390|UniProtKB=P50148,HUMAN|HGNC=10701|UniProtKB=Q15436,HUMAN|HGNC=18423|UniProtKB=O75140,HUMAN|HGNC=25694|UniProtKB=Q8TC07,HUMAN|HGNC=29351|UniProtKB=Q9C0H5,HUMAN|HGNC=9847|UniProtKB=P43487,HUMAN|HGNC=12363|UniProtKB=P49815,HUMAN|HGNC=17073|UniProtKB=Q9UNA1,HUMAN|HGNC=25571|UniProtKB=Q9NVG8,HUMAN|HGNC=21035|UniProtKB=Q8N392	GTPase activator activity
GO:0008037	HUMAN|HGNC=5575|UniProtKB=A0A0B4J1V2,HUMAN|HGNC=5645|UniProtKB=A0A0C4DH34,HUMAN|HGNC=5549|UniProtKB=A0A0C4DH31,HUMAN|HGNC=5554|UniProtKB=P01743,HUMAN|HG	cell recognition

<table>
<tr><th></th><th></th><th></th></tr>
<tr><td></td><td>NC=5480|UniProtKB=P01880,HUMAN|HGNC=5659|UniProtKB=A0A0C4DH38,HUMAN|HGNC=5634|UniProtKB=A0A075B7F0,HUMAN|HGNC=38476|UniProtKB=B9A064,HUMAN|HGNC=5861|UniProtKB=A0M8Q6,HUMAN|HGNC=5647|UniProtKB=A0A087WSY4,HUMAN|HGNC=5563|UniProtKB=A0A075B7D0,HUMAN|HGNC=5856|UniProtKB=P0DOY2,HUMAN|HGNC=5606|UniProtKB=P01763,HUMAN|HGNC=5576|UniProtKB=P01817,HUMAN|HGNC=5528|UniProtKB=P01861,HUMAN|HGNC=5541|UniProtKB=P01871,HUMAN|HGNC=5585|UniProtKB=A0A0C4DH32,HUMAN|HGNC=5551|UniProtKB=A0A0C4DH33,HUMAN|HGNC=5526|UniProtKB=P01859,HUMAN|HGNC=5657|UniProtKB=P0DP08,HUMAN|Ensembl=ENSG00000282639|UniProtKB=A0A0J9YX35,HUMAN|Gene=HV309_HUMAN|UniProtKB=P01782,HUMAN|HGNC=5654|UniProtKB=P01825,HUMAN|HGNC=5553|UniProtKB=A0A0A0MS14,HUMAN|Gene=HV108_HUMAN|UniProtKB=P0DP01,HUMAN|HGNC=5624|UniProtKB=A0A0B4J1X5,HUMAN|HGNC=5620|UniProtKB=P01780,HUMAN|HGNC=5598|UniProtKB=A0A0C4DH35,HUMAN|HGNC=5580|UniProtKB=P01762,HUMAN|HGNC=5633|UniProtKB=A0A075B7D8,HUMAN|HGNC=5582|UniProtKB=A0A0B4J1V0,HUMAN|HGNC=5478|UniProtKB=P01876,HUMAN|HGNC=5617|UniProtKB=A0A075B6Q5,HUMAN|HGNC=5594|UniProtKB=P0DP03,HUMAN|HGNC=5655|UniProtKB=A0A0C4DH41,HUMAN|HGNC=5636|UniProtKB=A0A075B7B8,HUMAN|HGNC=5586|UniProtKB=A0A0B4J1V1,HUMAN|HGNC=5581|UniProtKB=P01766,HUMAN|HGNC=29557|UniProtKB=Q0ZGT2,HUMAN|HGNC=5622|UniProtKB=A0A0B4J1Y9,HUMAN|HGNC=5652|UniProtKB=A0A075B6R2,HUMAN|HGNC=5593|UniProtKB=P0DP02,HUMAN|HGNC=5479|UniProtKB=P01877,HUMAN|HGNC=5619|UniProtKB=A0A0C4DH42,HUMAN|HGNC=5596|UniProtKB=P01772,HUMAN|HGNC=5550|UniProtKB=P23083,HUMAN|HGNC=5604|UniProtKB=A0A0B4J1X8,HUMAN|HGNC=5525|UniProtKB=P01857,HUMAN|HGNC=5662|UniProtKB=A0A0B4J1U7,HUMAN|HGNC=5601|UniProtKB=A0A0C4DH36,HUMAN|HGNC=5716|UniProtKB=P01834,HUMAN|HGNC=5588|UniProtKB=P01764,HUMAN|HGNC=5857|UniProtKB=P0DOY3,HUMAN|HGNC=5610|UniProtKB=P01767,HUMAN|Ensembl=ENSG00000282150|UniProtKB=A0A0J9YY99,HUMAN|HGNC=5527|UniProtKB=P01860</td><td></td></tr>
<tr><td>GO:0097373</td><td>HUMAN|HGNC=26200|UniProtKB=Q9H668</td><td>MCM core complex</td></tr>
<tr><td>GO:0032040</td><td>HUMAN|HGNC=12755|UniProtKB=Q9UNX4</td><td>small-subunit processome</td></tr>
<tr><td>GO:0009893</td><td>HUMAN|HGNC=613|UniProtKB=P02649</td><td>positive regulation of metabolic process</td></tr>
<tr><td>GO:0072655</td><td>HUMAN|HGNC=26801|UniProtKB=Q8N8Q8</td><td>establishment of protein localization to mitochondrion</td></tr>
<tr><td>GO:0051240</td><td>HUMAN|HGNC=11491|UniProtKB=P43405,HUMAN|HGNC=3537|UniProtKB=P25116,HUMAN|HGNC=9065|UniProtKB=P19174</td><td>positive regulation of multicellular organismal process</td></tr>
<tr><td>GO:0005905</td><td>HUMAN|HGNC=24634|UniProtKB=Q9UBC2,HUMAN|HGNC=3419|UniProtKB=P42566,HUMAN|HGNC=561|UniProtKB=O95782</td><td>clathrin-coated pit</td></tr>
<tr><td>PC00085</td><td>HUMAN|HGNC=15848|UniProtKB=Q14141,HUMAN|HGNC=3383|UniProtKB=P27105,HUMAN|HGNC=9164|UniProtKB=Q99719,HUMAN|HGNC=9070|UniProtKB=P08567,HUMAN|HGNC=1717|UniProtKB=Q16181,HUMAN|HGNC=7729|UniProtKB=Q15019</td><td>cytoskeletal protein</td></tr>
</table>

GO:0008622	HUMAN|HGNC=26200|UniProtKB=Q9H668	epsilon DNA polymerase complex
GO:1901264	HUMAN|HGNC=30220|UniProtKB=Q96AA3	carbohydrate derivative transport
GO:0016226	HUMAN|HGNC=3951|UniProtKB=Q16595	iron-sulfur cluster assembly
GO:0031625	HUMAN|HGNC=6767|UniProtKB=Q15797,HUMAN|HGNC=12458|UniProtKB=P62987,HUMAN|HGNC=12468|UniProtKB=P0CG48,HUMAN|HGNC=5239|UniProtKB=P17066,HUMAN|HGNC=2552|UniProtKB=Q13617,HUMAN|HGNC=5240|UniProtKB=P48741,HUMAN|HGNC=2551|UniProtKB=Q13616,HUMAN|HGNC=7732|UniProtKB=Q15843,HUMAN|HGNC=12463|UniProtKB=P0CG47	ubiquitin protein ligase binding
GO:0035371	HUMAN|HGNC=6891|UniProtKB=Q15555,HUMAN|HGNC=6890|UniProtKB=Q15691,HUMAN|HGNC=28959|UniProtKB=Q14008	microtubule plus-end
GO:0023061	HUMAN|HGNC=1628|UniProtKB=P08571,HUMAN|HGNC=11512|UniProtKB=Q9H2B2	signal release
GO:0006937	HUMAN|HGNC=544|UniProtKB=P08133	regulation of muscle contraction
GO:0007006	HUMAN|HGNC=26801|UniProtKB=Q8N8Q8	mitochondrial membrane organization
GO:0006879	HUMAN|HGNC=3951|UniProtKB=Q16595,HUMAN|HGNC=11763|UniProtKB=P02786	cellular iron ion homeostasis
GO:0045121	HUMAN|HGNC=26068|UniProtKB=Q6IAA8,HUMAN|HGNC=17955|UniProtKB=O43504,HUMAN|HGNC=1628|UniProtKB=P08571,HUMAN|HGNC=15606|UniProtKB=Q9UHA4	membrane raft
GO:0005868	HUMAN|HGNC=15476|UniProtKB=P63167	cytoplasmic dynein complex
GO:0003730	HUMAN|HGNC=17150|UniProtKB=Q9Y2V2,HUMAN|HGNC=21539|UniProtKB=Q9BX46,HUMAN|HGNC=17860|UniProtKB=Q8NC51	mRNA 3'-UTR binding
GO:0042407	HUMAN|HGNC=6047|UniProtKB=Q16891	cristae formation
GO:0045931	HUMAN|HGNC=533|UniProtKB=P04083	positive regulation of mitotic cell cycle
GO:0030117	HUMAN|HGNC=2974|UniProtKB=P50570,HUMAN|HGNC=29125|UniProtKB=Q9UQ16,HUMAN|HGNC=24634|UniProtKB=Q9UBC2,HUMAN|HGNC=2092|UniProtKB=Q00610,HUMAN|HGNC=3419|UniProtKB=P42566	membrane coat
GO:0065008	HUMAN|Gene=EIF2S3L|UniProtKB=Q2VIR3	regulation of biological quality
GO:0045259	HUMAN|HGNC=833|UniProtKB=P36542	proton-transporting ATP synthase complex
GO:0031032	HUMAN|HGNC=10252|UniProtKB=O75116,HUMAN|HGNC=1738|UniProtKB=Q9Y5S2	actomyosin structure organization
GO:0051315	HUMAN|HGNC=5048|UniProtKB=Q00839	attachment of mitotic spindle microtubules to kinetochore
PC00130	HUMAN|HGNC=1090|UniProtKB=Q03001,HUMAN|HGNC=9069|UniProtKB=Q15149	intermediate filament binding protein
GO:0043434	HUMAN|HGNC=8601|UniProtKB=Q06141,HUMAN|HGNC=533|UniProtKB=P04083	response to peptide hormone
GO:0002366	HUMAN|HGNC=11491|UniProtKB=P43405,HUMAN|HGNC=6517|UniProtKB=P18428	leukocyte activation involved in immune response

<table>
<tr><td>GO:0042325</td><td>HUMAN|HGNC=2288|UniProtKB=P14406</td><td>regulation of phosphorylation</td></tr>
<tr><td>GO:2001242</td><td>HUMAN|HGNC=1050|UniProtKB=P55957</td><td>regulation of intrinsic apoptotic signaling pathway</td></tr>
<tr><td>GO:0000922</td><td>HUMAN|HGNC=28959|UniProtKB=Q14008,HUMAN|HGNC=14906|UniProtKB=Q8N4C6</td><td>spindle pole</td></tr>
<tr><td>GO:0002377</td><td>HUMAN|HGNC=5753|UniProtKB=P01593,HUMAN|HGNC=5739|UniProtKB=A0A075B6S9,HUMAN|HGNC=5879|UniProtKB=P01699,HUMAN|HGNC=5825|UniProtKB=A0A0C4DH25,HUMAN|HGNC=5800|UniProtKB=A0A075B6S2,HUMAN|HGNC=5880|UniProtKB=P01700,HUMAN|HGNC=5905|UniProtKB=P80748,HUMAN|HGNC=5742|UniProtKB=A0A0C4DH72,HUMAN|HGNC=5756|UniProtKB=P04432,HUMAN|HGNC=5896|UniProtKB=P01715,HUMAN|HGNC=5816|UniProtKB=P01624,HUMAN|Gene=KV311_HUMAN|UniProtKB=P04433,HUMAN|HGNC=5910|UniProtKB=P01718,HUMAN|HGNC=5931|UniProtKB=A0A075B6I0,HUMAN|HGNC=5735|UniProtKB=A0A075B6S5,HUMAN|HGNC=5933|UniProtKB=A0A0B4J1Y8,HUMAN|HGNC=5877|UniProtKB=P01703,HUMAN|HGNC=5785|UniProtKB=P06310,HUMAN|HGNC=5834|UniProtKB=P06312,HUMAN|HGNC=5836|UniProtKB=A0A0C4DH24,HUMAN|HGNC=5895|UniProtKB=P01709,HUMAN|HGNC=5898|UniProtKB=A0A075B6K2,HUMAN|HGNC=5887|UniProtKB=P01706,HUMAN|HGNC=5929|UniProtKB=P04211,HUMAN|HGNC=5882|UniProtKB=P01701,HUMAN|HGNC=5890|UniProtKB=P01705,HUMAN|HGNC=5732|UniProtKB=P04430,HUMAN|HGNC=5759|UniProtKB=A0A087WSZ0,HUMAN|HGNC=5733|UniProtKB=P01599,HUMAN|HGNC=5908|UniProtKB=P01717,HUMAN|HGNC=5746|UniProtKB=P01611,HUMAN|HGNC=5821|UniProtKB=A0A075B6H7,HUMAN|HGNC=5888|UniProtKB=P01704,HUMAN|HGNC=5889|UniProtKB=A0A075B6J9,HUMAN|HGNC=5927|UniProtKB=P01721,HUMAN|HGNC=5897|UniProtKB=A0A075B6K4,HUMAN|HGNC=5741|UniProtKB=P01602,HUMAN|HGNC=5730|UniProtKB=A0A0C4DH73,HUMAN|HGNC=5918|UniProtKB=A0A075B6K5,HUMAN|HGNC=5817|UniProtKB=P01619,HUMAN|HGNC=5781|UniProtKB=A0A0C4DH68,HUMAN|HGNC=5783|UniProtKB=A0A075B6P5,HUMAN|HGNC=5930|UniProtKB=A0A075B6I9,HUMAN|HGNC=5901|UniProtKB=A0A075B6K0,HUMAN|HGNC=5743|UniProtKB=A0A0C4DH67</td><td>immunoglobulin production</td></tr>
<tr><td>GO:0003712</td><td>HUMAN|HGNC=12347|UniProtKB=Q9Y4A5,HUMAN|HGNC=18807|UniProtKB=O94983,HUMAN|HGNC=11767|UniProtKB=O43294</td><td>transcription coregulator activity</td></tr>
<tr><td>PC00086</td><td>HUMAN|HGNC=1101|UniProtKB=P51587,HUMAN|HGNC=9813|UniProtKB=P54727</td><td>damaged DNA-binding protein</td></tr>
<tr><td>GO:0004497</td><td>HUMAN|HGNC=2689|UniProtKB=P09172</td><td>monooxygenase activity</td></tr>
<tr><td>GO:0009201</td><td>HUMAN|HGNC=2520|UniProtKB=Q9NRF8</td><td>ribonucleoside triphosphate biosynthetic process</td></tr>
<tr><td>GO:0000281</td><td>HUMAN|HGNC=10252|UniProtKB=O75116</td><td>mitotic cytokinesis</td></tr>
<tr><td>GO:0002705</td><td>HUMAN|HGNC=11491|UniProtKB=P43405</td><td>positive regulation of leukocyte mediated immunity</td></tr>
<tr><td>PC00090</td><td>HUMAN|HGNC=10719|UniProtKB=Q13228,HUMAN|HGNC=21245|UniProtKB=Q6UXB8</td><td>defense/immunity protein</td></tr>
<tr><td>GO:0004896</td><td>HUMAN|HGNC=1681|UniProtKB=P16070,HUMAN|HGNC=19100|UniProtKB=Q5VWK5</td><td>cytokine receptor activity</td></tr>
</table>

<table>
<tr><td>GO:1905324</td><td>HUMAN|HGNC=5048|UniProtKB=Q00839</td><td>telomere-telomerase complex assembly</td></tr>
<tr><td>PC00039</td><td>HUMAN|HGNC=17780|UniProtKB=Q562R1,HUMAN|HGNC=167|UniProtKB=P61163,HUMAN|HGNC=169|UniProtKB=P61160,HUMAN|HGNC=143|UniProtKB=P68032,HUMAN|HGNC=14653|UniProtKB=Q9HBI1,HUMAN|HGNC=170|UniProtKB=P61158,HUMAN|HGNC=144|UniProtKB=P63261</td><td>actin and actin related protein</td></tr>
<tr><td>GO:0010951</td><td>HUMAN|HGNC=333|UniProtKB=P01019,HUMAN|HGNC=2479|UniProtKB=O76096,HUMAN|HGNC=8583|UniProtKB=P05121,HUMAN|HGNC=3311|UniProtKB=P30740,HUMAN|HGNC=8948|UniProtKB=P29622,HUMAN|HGNC=1546|UniProtKB=P50454,HUMAN|HGNC=8941|UniProtKB=P01009,HUMAN|HGNC=1540|UniProtKB=P08185,HUMAN|HGNC=8824|UniProtKB=P36955,HUMAN|HGNC=8942|UniProtKB=P48595,HUMAN|HGNC=3658|UniProtKB=Q9UGM5,HUMAN|HGNC=11820|UniProtKB=P01033,HUMAN|HGNC=10569|UniProtKB=P29508,HUMAN|HGNC=4838|UniProtKB=P05546,HUMAN|HGNC=6383|UniProtKB=P01042,HUMAN|HGNC=16|UniProtKB=P01011,HUMAN|HGNC=775|UniProtKB=P01008,HUMAN|HGNC=1228|UniProtKB=P05155,HUMAN|HGNC=9075|UniProtKB=P08697,HUMAN|HGNC=11583|UniProtKB=P05543,HUMAN|HGNC=8955|UniProtKB=P50453</td><td>negative regulation of endopeptidase activity</td></tr>
<tr><td>GO:2001251</td><td>HUMAN|HGNC=23077|UniProtKB=Q96FW1,HUMAN|HGNC=5048|UniProtKB=Q00839</td><td>negative regulation of chromosome organization</td></tr>
<tr><td>GO:0008094</td><td>HUMAN|HGNC=7326|UniProtKB=P20585</td><td>DNA-dependent ATPase activity</td></tr>
<tr><td>GO:1990778</td><td>HUMAN|HGNC=1406|UniProtKB=Q9Y698</td><td>protein localization to cell periphery</td></tr>
<tr><td>GO:0006360</td><td>HUMAN|HGNC=25758|UniProtKB=Q8TED0</td><td>transcription by RNA polymerase I</td></tr>
<tr><td>GO:0000976</td><td>HUMAN|HGNC=9885|UniProtKB=P29374,HUMAN|HGNC=10774|UniProtKB=P23246,HUMAN|HGNC=10055|UniProtKB=Q8N5U6</td><td>transcription regulatory region sequence-specific DNA binding</td></tr>
<tr><td>GO:0000347</td><td>HUMAN|HGNC=19072|UniProtKB=Q96J01</td><td>THO complex</td></tr>
<tr><td>PC00195</td><td>HUMAN|HGNC=122|UniProtKB=P24666,HUMAN|HGNC=9300|UniProtKB=P62714,HUMAN|HGNC=9635|UniProtKB=Q12974,HUMAN|HGNC=9658|UniProtKB=P29350,HUMAN|HGNC=19388|UniProtKB=P49593,HUMAN|HGNC=9666|UniProtKB=P08575,HUMAN|HGNC=9299|UniProtKB=P67775,HUMAN|HGNC=9314|UniProtKB=Q08209,HUMAN|HGNC=9673|UniProtKB=Q12913,HUMAN|HGNC=9304|UniProtKB=P63151,HUMAN|HGNC=9281|UniProtKB=P62136</td><td>protein phosphatase</td></tr>
<tr><td>GO:0005246</td><td>HUMAN|HGNC=4234|UniProtKB=P55040</td><td>calcium channel regulator activity</td></tr>
<tr><td>GO:0016358</td><td>HUMAN|HGNC=6835|UniProtKB=P78559</td><td>dendrite development</td></tr>
<tr><td>PC00148</td><td>HUMAN|HGNC=20471|UniProtKB=O95777,HUMAN|HGNC=5046|UniProtKB=P52272,HUMAN|HGNC=5043|UniProtKB=P31942,HUMAN|HGNC=17259|UniProtKB=Q9Y4Z0</td><td>RNA splicing factor</td></tr>
<tr><td>GO:0051453</td><td>HUMAN|HGNC=11030|UniProtKB=Q9Y6R1,HUMAN|HGNC=11027|UniProtKB=P02730,HUMAN|HGNC=11034|UniProtKB=Q2Y0W8</td><td>regulation of intracellular pH</td></tr>
<tr><td>GO:0008328</td><td>HUMAN|HGNC=1406|UniProtKB=Q9Y698,HUMAN|HGNC=15474|UniProtKB=Q9Y566,HUMAN|HGNC=14294|UniProtKB=Q9BYB0,HUMAN|HGNC=30377|UniProtKB=Q14160</td><td>ionotropic glutamate receptor complex</td></tr>
</table>

GO:0032587	HUMAN|HGNC=21295|UniProtKB=Q8TE68	ruffle membrane
PC00020	HUMAN|HGNC=654|UniProtKB=P61204,HUMAN|HGNC=692|UniProtKB=P40616,HUMAN|HGNC=652|UniProtKB=P84077,HUMAN|HGNC=659|UniProtKB=P62330,HUMAN|HGNC=658|UniProtKB=P84085,HUMAN|HGNC=655|UniProtKB=P18085	G-protein
GO:0050808	HUMAN|HGNC=1736|UniProtKB=P60953,HUMAN|HGNC=11138|UniProtKB=P37840	synapse organization
GO:0006515	HUMAN|HGNC=12666|UniProtKB=P55072,HUMAN|HGNC=15663|UniProtKB=Q9NYU2	protein quality control for misfolded or incompletely synthesized proteins
GO:0008374	HUMAN|HGNC=6522|UniProtKB=P04180,HUMAN|HGNC=30244|UniProtKB=Q6P1A2,HUMAN|HGNC=2328|UniProtKB=P50416	O-acyltransferase activity
GO:0045277	HUMAN|HGNC=7687|UniProtKB=O00483,HUMAN|HGNC=2265|UniProtKB=P13073,HUMAN|HGNC=2267|UniProtKB=P20674	respiratory chain complex IV
GO:0044853	HUMAN|HGNC=10690|UniProtKB=O95810,HUMAN|HGNC=3757|UniProtKB=O75955,HUMAN|HGNC=243|UniProtKB=P35611	plasma membrane raft
GO:0071013	HUMAN|HGNC=29513|UniProtKB=O60306,HUMAN|HGNC=5048|UniProtKB=Q00839	catalytic step 2 spliceosome
GO:0006122	HUMAN|HGNC=12582|UniProtKB=P14927,HUMAN|HGNC=12590|UniProtKB=P07919,HUMAN|HGNC=19986|UniProtKB=P99999	mitochondrial electron transport, ubiquinol to cytochrome c
GO:0030512	HUMAN|HGNC=23688|UniProtKB=Q86UU0	negative regulation of transforming growth factor beta receptor signaling pathway
GO:0008016	HUMAN|HGNC=10582|UniProtKB=Q9Y5Y9	regulation of heart contraction
GO:0006415	HUMAN|HGNC=4622|UniProtKB=Q8IYD1,HUMAN|HGNC=4621|UniProtKB=P15170	translational termination
GO:0003678	HUMAN|HGNC=10474|UniProtKB=Q9Y265,HUMAN|HGNC=20473|UniProtKB=Q9BX63,HUMAN|HGNC=10475|UniProtKB=Q9Y230	DNA helicase activity
GO:0016407	HUMAN|HGNC=94|UniProtKB=Q9BWD1	acetyltransferase activity
GO:0007005	HUMAN|HGNC=3951|UniProtKB=Q16595,HUMAN|HGNC=24808|UniProtKB=O14545,HUMAN|HGNC=2288|UniProtKB=P14406,HUMAN|HGNC=23594|UniProtKB=Q709C8	mitochondrion organization
GO:0008289	HUMAN|HGNC=25268|UniProtKB=Q96LR9,HUMAN|HGNC=9922|UniProtKB=P02753,HUMAN|HGNC=607|UniProtKB=P02654,HUMAN|HGNC=317|UniProtKB=P02771,HUMAN|HGNC=618|UniProtKB=O14791,HUMAN|HGNC=121|UniProtKB=O15254,HUMAN|HGNC=119|UniProtKB=Q15067	lipid binding
GO:1990542	HUMAN|HGNC=5261|UniProtKB=P10809,HUMAN|HGNC=23526|UniProtKB=Q8NE86,HUMAN|HGNC=830|UniProtKB=P06576,HUMAN|HGNC=850|UniProtKB=P48047	mitochondrial transmembrane transport
GO:0031056	HUMAN|HGNC=23077|UniProtKB=Q96FW1	regulation of histone modification
GO:0005911	HUMAN|HGNC=6616|UniProtKB=P48059,HUMAN|HGNC=3048|UniProtKB=Q02413	cell-cell junction
GO:0006636	HUMAN|HGNC=533|UniProtKB=P04083	unsaturated fatty acid biosynthetic process
GO:0031330	HUMAN|HGNC=11820|UniProtKB=P01033	negative regulation of cellular catabolic process

GO:0070847	HUMAN|HGNC=11957|UniProtKB=Q93074	core mediator complex
GO:0002891	HUMAN|HGNC=16961|UniProtKB=Q15762	positive regulation of immunoglobulin mediated immune response
GO:0008541	HUMAN|HGNC=9556|UniProtKB=O00231	proteasome regulatory particle, lid subcomplex
GO:0007596	HUMAN|HGNC=3530|UniProtKB=P00748,HUMAN|HGNC=3531|UniProtKB=P00488,HUMAN|HGNC=3537|UniProtKB=P25116,HUMAN|HGNC=13937|UniProtKB=O95866,HUMAN|HGNC=6383|UniProtKB=P01042,HUMAN|HGNC=12726|UniProtKB=P04275,HUMAN|HGNC=2300|UniProtKB=Q96IY4	blood coagulation
GO:0016773	HUMAN|HGNC=8876|UniProtKB=P17858,HUMAN|HGNC=24552|UniProtKB=Q3LXA3,HUMAN|HGNC=4922|UniProtKB=P19367,HUMAN|HGNC=9021|UniProtKB=P14618,HUMAN|HGNC=8878|UniProtKB=Q01813,HUMAN|HGNC=2850|UniProtKB=Q9Y6T7	phosphotransferase activity, alcohol group as acceptor
GO:0032968	HUMAN|HGNC=11617|UniProtKB=Q15369	positive regulation of transcription elongation from RNA polymerase II promoter
GO:0006457	HUMAN|HGNC=1623|UniProtKB=P50990,HUMAN|HGNC=1617|UniProtKB=P50991,HUMAN|HGNC=1622|UniProtKB=Q99832,HUMAN|HGNC=30167|UniProtKB=P13667,HUMAN|HGNC=4926|UniProtKB=O15212,HUMAN|HGNC=1455|UniProtKB=P27797,HUMAN|HGNC=1735|UniProtKB=Q16543,HUMAN|HGNC=11582|UniProtKB=Q15813,HUMAN|HGNC=8548|UniProtKB=P07237,HUMAN|HGNC=1473|UniProtKB=P27824,HUMAN|HGNC=11579|UniProtKB=O75347,HUMAN|HGNC=4606|UniProtKB=P30101,HUMAN|HGNC=1615|UniProtKB=P78371,HUMAN|HGNC=5261|UniProtKB=P10809,HUMAN|HGNC=5253|UniProtKB=P07900,HUMAN|HGNC=11655|UniProtKB=P17987,HUMAN|HGNC=1618|UniProtKB=P48643,HUMAN|HGNC=5258|UniProtKB=P08238,HUMAN|HGNC=1620|UniProtKB=P40227,HUMAN|HGNC=12028|UniProtKB=P14625,HUMAN|HGNC=32153|UniProtKB=A6NM43,HUMAN|HGNC=9756|UniProtKB=O00391,HUMAN|HGNC=30535|UniProtKB=Q8WVJ2	protein folding
GO:0006575	HUMAN|HGNC=2328|UniProtKB=P50416,HUMAN|HGNC=13921|UniProtKB=O95870	cellular modified amino acid metabolic process
GO:0098742	HUMAN|HGNC=29943|UniProtKB=Q9NT68,HUMAN|HGNC=8823|UniProtKB=P16284,HUMAN|HGNC=15902|UniProtKB=Q8IXH8,HUMAN|HGNC=14265|UniProtKB=Q86UP0	cell-cell adhesion via plasma-membrane adhesion molecules
GO:0015291	HUMAN|HGNC=23526|UniProtKB=Q8NE86,HUMAN|HGNC=10989|UniProtKB=Q00325	secondary active transmembrane transporter activity
GO:0042379	HUMAN|HGNC=9240|UniProtKB=P02775,HUMAN|HGNC=10632|UniProtKB=P13501,HUMAN|HGNC=8862|UniProtKB=P10720,HUMAN|HGNC=8861|UniProtKB=P02776	chemokine receptor binding
GO:0031410	HUMAN|HGNC=33704|UniProtKB=Q66K66,HUMAN|HGNC=13206|UniProtKB=O60296,HUMAN|HGNC=18756|UniProtKB=Q9BYZ6,HUMAN|HGNC=3757|UniProtKB=O75955,HUMAN|HGNC=9802|UniProtKB=P15153,HUMAN|HGNC=570|UniProtKB=P53677,HUMAN|HGNC=2974|UniProtKB=P50570,HUMAN|HGNC=9801|UniProtKB=P63000,HUMAN|HGNC=29125|UniProtKB=Q9UQ16,HUMAN|HGNC=30138|UniProtKB=Q8N131	cytoplasmic vesicle

GO:0000463	HUMAN|HGNC=10363|UniProtKB=P18124	maturation of LSU-rRNA from tricistronic rRNA transcript (SSU-rRNA, 5.8S rRNA, LSU-rRNA)
GO:0006839	HUMAN|HGNC=26801|UniProtKB=Q8N8Q8	mitochondrial transport
PC00221	HUMAN|HGNC=11834|UniProtKB=P29401	transketolase
GO:0000775	HUMAN|HGNC=4861|UniProtKB=Q9NRZ9	chromosome, centromeric region
GO:0008380	HUMAN|HGNC=27424|UniProtKB=Q5T481,HUMAN|HGNC=5043|UniProtKB=P31942,HUMAN|HGNC=17894|UniProtKB=Q14966,HUMAN|HGNC=347|UniProtKB=Q09666	RNA splicing
GO:0031982	HUMAN|HGNC=8898|UniProtKB=P07205,HUMAN|HGNC=8044|UniProtKB=P80303,HUMAN|HGNC=2478|UniProtKB=Q15828,HUMAN|HGNC=11937|UniProtKB=P32970,HUMAN|HGNC=10499|UniProtKB=P06702,HUMAN|HGNC=5239|UniProtKB=P17066,HUMAN|HGNC=11834|UniProtKB=P29401,HUMAN|HGNC=28883|UniProtKB=Q9H2U2,HUMAN|HGNC=5240|UniProtKB=P48741,HUMAN|HGNC=21295|UniProtKB=Q8TE68,HUMAN|HGNC=8043|UniProtKB=Q02818	vesicle
GO:0044262	HUMAN|HGNC=8876|UniProtKB=P17858,HUMAN|HGNC=29035|UniProtKB=O43314,HUMAN|HGNC=8878|UniProtKB=Q01813,HUMAN|HGNC=6050|UniProtKB=P29218	cellular carbohydrate metabolic process
GO:0043043	HUMAN|HGNC=4312|UniProtKB=P48507	peptide biosynthetic process
GO:0051276	HUMAN|HGNC=7326|UniProtKB=P20585	chromosome organization
GO:0005085	HUMAN|HGNC=26068|UniProtKB=Q6IAA8,HUMAN|HGNC=17955|UniProtKB=O43504,HUMAN|HGNC=3208|UniProtKB=P24534,HUMAN|HGNC=3211|UniProtKB=P29692	guanyl-nucleotide exchange factor activity
GO:0017049	HUMAN|HGNC=10252|UniProtKB=O75116	GTP-Rho binding
GO:0055082	HUMAN|HGNC=4922|UniProtKB=P19367	cellular chemical homeostasis
GO:0030336	HUMAN|HGNC=9862|UniProtKB=P61225,HUMAN|HGNC=7178|UniProtKB=Q13201	negative regulation of cell migration
GO:0030126	HUMAN|HGNC=2230|UniProtKB=P53621,HUMAN|HGNC=2231|UniProtKB=P53618,HUMAN|HGNC=649|UniProtKB=P48444,HUMAN|HGNC=2232|UniProtKB=P35606	COPI vesicle coat
GO:0031110	HUMAN|HGNC=6891|UniProtKB=Q15555,HUMAN|HGNC=6890|UniProtKB=Q15691,HUMAN|HGNC=6835|UniProtKB=P78559,HUMAN|HGNC=6510|UniProtKB=P16949	regulation of microtubule polymerization or depolymerization
GO:1902275	HUMAN|HGNC=5048|UniProtKB=Q00839	regulation of chromatin organization
GO:0016192	HUMAN|HGNC=654|UniProtKB=P61204,HUMAN|HGNC=692|UniProtKB=P40616,HUMAN|HGNC=652|UniProtKB=P84077,HUMAN|HGNC=4226|UniProtKB=P31150,HUMAN|HGNC=575|UniProtKB=Q9Y587,HUMAN|HGNC=658|UniProtKB=P84085,HUMAN|HGNC=4227|UniProtKB=P50395,HUMAN|HGNC=655|UniProtKB=P18085,HUMAN|HGNC=5241|UniProtKB=P11142,HUMAN|HGNC=6317|UniProtKB=O43896,HUMAN|HGNC=659|UniProtKB=P62330,HUMAN|HGNC=13667|UniProtKB=Q9BXS5,HUMAN|HGNC=1365|UniProtKB=Q9UGQ2,HUMAN|HGNC=554|UniProtKB=Q10567	vesicle-mediated transport

GO:0016462	HUMAN|HGNC=13420|UniProtKB=Q86TP1,HUMAN|HGNC=28883|UniProtKB=Q9H2U2,HUMAN|HGNC=8052|UniProtKB=Q9UKK9	pyrophosphatase activity
GO:0060285	HUMAN|HGNC=25631|UniProtKB=Q96MT7	cilium-dependent cell motility
GO:0043169	HUMAN|HGNC=8876|UniProtKB=P17858,HUMAN|HGNC=626|UniProtKB=P07741,HUMAN|HGNC=11834|UniProtKB=P29401,HUMAN|HGNC=8878|UniProtKB=Q01813,HUMAN|HGNC=9386|UniProtKB=Q9UGJ0	cation binding
GO:0006954	HUMAN|HGNC=2479|UniProtKB=O76096,HUMAN|HGNC=533|UniProtKB=P04083,HUMAN|HGNC=10632|UniProtKB=P13501,HUMAN|HGNC=1681|UniProtKB=P16070,HUMAN|HGNC=5141|UniProtKB=P00738,HUMAN|HGNC=3309|UniProtKB=P08246,HUMAN|HGNC=9240|UniProtKB=P02775,HUMAN|HGNC=7967|UniProtKB=Q96RI1,HUMAN|HGNC=8862|UniProtKB=P10720,HUMAN|HGNC=8861|UniProtKB=P02776,HUMAN|HGNC=1628|UniProtKB=P08571,HUMAN|HGNC=5156|UniProtKB=P00739,HUMAN|HGNC=6517|UniProtKB=P18428	inflammatory response
GO:0045954	HUMAN|HGNC=16961|UniProtKB=Q15762	positive regulation of natural killer cell mediated cytotoxicity
GO:0005789	HUMAN|HGNC=16695|UniProtKB=P51572,HUMAN|HGNC=1801|UniProtKB=O95674,HUMAN|HGNC=26212|UniProtKB=P61009,HUMAN|HGNC=11834|UniProtKB=P29401,HUMAN|HGNC=12666|UniProtKB=P55072,HUMAN|HGNC=1455|UniProtKB=P27797,HUMAN|HGNC=6631|UniProtKB=P49257,HUMAN|HGNC=15999|UniProtKB=Q9NZV5,HUMAN|HGNC=12649|UniProtKB=O95292,HUMAN|HGNC=1473|UniProtKB=P27824,HUMAN|HGNC=12648|UniProtKB=Q9P0L0,HUMAN|HGNC=30220|UniProtKB=Q96AA3	endoplasmic reticulum membrane
PC00161	HUMAN|HGNC=9086|UniProtKB=P60201,HUMAN|HGNC=6925|UniProtKB=P02686	myelin protein
GO:0051640	HUMAN|HGNC=31332|UniProtKB=Q6IQ22,HUMAN|HGNC=7007|UniProtKB=P61006,HUMAN|HGNC=11446|UniProtKB=O00186,HUMAN|HGNC=11442|UniProtKB=O15400,HUMAN|HGNC=11445|UniProtKB=Q15833,HUMAN|HGNC=11429|UniProtKB=O75558,HUMAN|HGNC=9778|UniProtKB=P20337,HUMAN|HGNC=12649|UniProtKB=O95292,HUMAN|HGNC=649|UniProtKB=P48444,HUMAN|HGNC=12648|UniProtKB=Q9P0L0,HUMAN|HGNC=8016|UniProtKB=P46459	organelle localization
GO:0031328	HUMAN|HGNC=533|UniProtKB=P04083,HUMAN|HGNC=6517|UniProtKB=P18428	positive regulation of cellular biosynthetic process
GO:0042278	HUMAN|HGNC=5157|UniProtKB=P00492	purine nucleoside metabolic process
GO:0060968	HUMAN|HGNC=20250|UniProtKB=Q96IF1	regulation of gene silencing
GO:0005874	HUMAN|HGNC=12407|UniProtKB=P68366,HUMAN|HGNC=2949|UniProtKB=Q8TD57,HUMAN|HGNC=12410|UniProtKB=Q9NY65,HUMAN|HGNC=20778|UniProtKB=P07437,HUMAN|HGNC=6835|UniProtKB=P78559,HUMAN|HGNC=20766|UniProtKB=Q71U36,HUMAN|HGNC=20774|UniProtKB=P04350,HUMAN|HGNC=20771|UniProtKB=P68371,HUMAN|HGNC=17060|UniProtKB=Q92845,HUMAN|HGNC=30391|UniProtKB=Q9UG01,HUMAN|HGNC=6325|UniProtKB=O60282,HUMAN|HGNC=2952|UniProtKB=Q96JB1,HUMAN|HGNC=6317|UniProtKB=O43896,HUMAN|HGNC=16257|UniProtKB=Q9H4B7	microtubule

<table>
<tr><td>GO:0030073</td><td>HUMAN|HGNC=533|UniProtKB=P04083</td><td>insulin secretion</td></tr>
<tr><td>GO:0002020</td><td>HUMAN|HGNC=333|UniProtKB=P01019,HUMAN|HGNC=20635|UniProtKB=Q14997,HUMAN|HGNC=11092|UniProtKB=P03973,HUMAN|HGNC=2482|UniProtKB=P04080,HUMAN|HGNC=17943|UniProtKB=Q9GZP9,HUMAN|HGNC=8824|UniProtKB=P36955,HUMAN|HGNC=8942|UniProtKB=P48595,HUMAN|HGNC=3658|UniProtKB=Q9UGM5,HUMAN|HGNC=11820|UniProtKB=P01033,HUMAN|HGNC=6383|UniProtKB=P01042,HUMAN|HGNC=775|UniProtKB=P01008,HUMAN|HGNC=1228|UniProtKB=P05155,HUMAN|HGNC=9075|UniProtKB=P08697,HUMAN|HGNC=11583|UniProtKB=P05543,HUMAN|HGNC=453|UniProtKB=P02760,HUMAN|HGNC=2479|UniProtKB=O76096,HUMAN|HGNC=8583|UniProtKB=P05121,HUMAN|HGNC=3311|UniProtKB=P30740,HUMAN|HGNC=8948|UniProtKB=P29622,HUMAN|HGNC=9750|UniProtKB=P20742,HUMAN|HGNC=1546|UniProtKB=P50454,HUMAN|HGNC=8941|UniProtKB=P01009,HUMAN|HGNC=1540|UniProtKB=P08185,HUMAN|HGNC=10569|UniProtKB=P29508,HUMAN|HGNC=4838|UniProtKB=P05546,HUMAN|HGNC=16|UniProtKB=P01011,HUMAN|HGNC=9568|UniProtKB=Q06323,HUMAN|HGNC=8955|UniProtKB=P50453</td><td>protease binding</td></tr>
<tr><td>GO:0008171</td><td>HUMAN|HGNC=8728|UniProtKB=P22061</td><td>O-methyltransferase activity</td></tr>
<tr><td>GO:0060627</td><td>HUMAN|HGNC=26486|UniProtKB=Q6PJW8,HUMAN|HGNC=10534|UniProtKB=Q9NR31,HUMAN|HGNC=17643|UniProtKB=Q9HD26</td><td>regulation of vesicle-mediated transport</td></tr>
<tr><td>GO:0031497</td><td>HUMAN|HGNC=4716|UniProtKB=P16403,HUMAN|HGNC=4718|UniProtKB=P10412,HUMAN|HGNC=4719|UniProtKB=P16401</td><td>chromatin assembly</td></tr>
<tr><td>GO:0007159</td><td>HUMAN|HGNC=23151|UniProtKB=Q86UX7,HUMAN|HGNC=5261|UniProtKB=P10809,HUMAN|HGNC=11491|UniProtKB=P43405,HUMAN|HGNC=533|UniProtKB=P04083</td><td>leukocyte cell-cell adhesion</td></tr>
<tr><td>GO:0030837</td><td>HUMAN|HGNC=9621|UniProtKB=Q6IBS0,HUMAN|HGNC=12690|UniProtKB=P09327,HUMAN|HGNC=243|UniProtKB=P35611,HUMAN|HGNC=1488|UniProtKB=P52907,HUMAN|HGNC=3750|UniProtKB=Q13045,HUMAN|HGNC=1491|UniProtKB=P47756,HUMAN|HGNC=15667|UniProtKB=P58546,HUMAN|HGNC=11881|UniProtKB=P62328</td><td>negative regulation of actin filament polymerization</td></tr>
<tr><td>GO:0002376</td><td>HUMAN|HGNC=8993|UniProtKB=P12273</td><td>immune system process</td></tr>
<tr><td>GO:0051129</td><td>HUMAN|HGNC=25361|UniProtKB=Q8N264</td><td>negative regulation of cellular component organization</td></tr>
<tr><td>GO:0016836</td><td>HUMAN|HGNC=8876|UniProtKB=P17858,HUMAN|HGNC=118|UniProtKB=Q99798,HUMAN|HGNC=16369|UniProtKB=Q99497,HUMAN|HGNC=4801|UniProtKB=P40939,HUMAN|HGNC=8878|UniProtKB=Q01813,HUMAN|HGNC=395|UniProtKB=P13716,HUMAN|HGNC=3151|UniProtKB=P30084,HUMAN|HGNC=24175|UniProtKB=Q9P035,HUMAN|HGNC=1368|UniProtKB=P00915,HUMAN|HGNC=117|UniProtKB=P21399,HUMAN|HGNC=3350|UniProtKB=P06733,HUMAN|HGNC=3353|UniProtKB=P09104,HUMAN|HGNC=9021|UniProtKB=P14618,HUMAN|HGNC=3354|UniProtKB=P13929,HUMAN|HGNC=1373|UniProtKB=P00918</td><td>hydro-lyase activity</td></tr>
<tr><td>GO:0042147</td><td>HUMAN|HGNC=11172|UniProtKB=Q13596,HUMAN|HGNC=9786|UniProtKB=P20340,HUMAN|HGNC=14902|UniProtKB=Q9NRW1,HUMAN|HGNC=13487|UniProtKB=Q96QK1</td><td>retrograde transport, endosome to Golgi</td></tr>
</table>

GO:0008045	HUMAN|HGNC=9802|UniProtKB=P15153,HUMAN|HGNC=9801|UniProtKB=P63000	motor neuron axon guidance
GO:0140101	HUMAN|HGNC=28422|UniProtKB=Q8NCE0,HUMAN|HGNC=24728|UniProtKB=A2RTX5,HUMAN|HGNC=2678|UniProtKB=P14868,HUMAN|HGNC=20|UniProtKB=P49588,HUMAN|HGNC=4162|UniProtKB=P41250,HUMAN|HGNC=11572|UniProtKB=P26639,HUMAN|HGNC=16219|UniProtKB=Q8TEA8,HUMAN|HGNC=12840|UniProtKB=P54577,HUMAN|HGNC=12729|UniProtKB=P23381	catalytic activity, acting on a tRNA
GO:0038061	HUMAN|HGNC=1628|UniProtKB=P08571,HUMAN|HGNC=3157|UniProtKB=Q92838	NIK/NF-kappaB signaling
GO:0060070	HUMAN|HGNC=33704|UniProtKB=Q66K66,HUMAN|HGNC=4045|UniProtKB=O75084,HUMAN|HGNC=23688|UniProtKB=Q86UU0,HUMAN|HGNC=9275|UniProtKB=P35813,HUMAN|HGNC=11119|UniProtKB=Q99835	canonical Wnt signaling pathway
GO:0006458	HUMAN|HGNC=5241|UniProtKB=P11142,HUMAN|HGNC=5269|UniProtKB=P61604,HUMAN|HGNC=5239|UniProtKB=P17066,HUMAN|HGNC=5238|UniProtKB=P11021,HUMAN|HGNC=5270|UniProtKB=P25685,HUMAN|HGNC=5240|UniProtKB=P48741,HUMAN|HGNC=11343|UniProtKB=P50502	'de novo' protein folding
GO:0015711	HUMAN|HGNC=11030|UniProtKB=Q9Y6R1,HUMAN|HGNC=11027|UniProtKB=P02730,HUMAN|HGNC=11034|UniProtKB=Q2Y0W8	organic anion transport
GO:0004252	HUMAN|HGNC=586|UniProtKB=P13798,HUMAN|HGNC=333|UniProtKB=P01019,HUMAN|HGNC=1247|UniProtKB=P09871,HUMAN|HGNC=11092|UniProtKB=P03973,HUMAN|HGNC=43788|UniProtKB=Q8NHM4,HUMAN|HGNC=8824|UniProtKB=P36955,HUMAN|HGNC=8942|UniProtKB=P48595,HUMAN|HGNC=775|UniProtKB=P01008,HUMAN|HGNC=1228|UniProtKB=P05155,HUMAN|HGNC=9075|UniProtKB=P08697,HUMAN|HGNC=5156|UniProtKB=P00739,HUMAN|HGNC=6902|UniProtKB=O00187,HUMAN|HGNC=11583|UniProtKB=P05543,HUMAN|HGNC=453|UniProtKB=P02760,HUMAN|HGNC=8583|UniProtKB=P05121,HUMAN|HGNC=9495|UniProtKB=P24158,HUMAN|HGNC=3311|UniProtKB=P30740,HUMAN|HGNC=7380|UniProtKB=P26927,HUMAN|HGNC=8948|UniProtKB=P29622,HUMAN|HGNC=8569|UniProtKB=P29122,HUMAN|HGNC=1546|UniProtKB=P50454,HUMAN|HGNC=8941|UniProtKB=P01009,HUMAN|HGNC=1540|UniProtKB=P08185,HUMAN|HGNC=12016|UniProtKB=P29144,HUMAN|HGNC=4798|UniProtKB=Q14520,HUMAN|HGNC=3530|UniProtKB=P00748,HUMAN|HGNC=5141|UniProtKB=P00738,HUMAN|HGNC=10569|UniProtKB=P29508,HUMAN|HGNC=4838|UniProtKB=P05546,HUMAN|HGNC=3309|UniProtKB=P08246,HUMAN|HGNC=21265|UniProtKB=Q9NZP8,HUMAN|HGNC=16|UniProtKB=P01011,HUMAN|HGNC=6901|UniProtKB=P48740,HUMAN|HGNC=9071|UniProtKB=P00747,HUMAN|HGNC=8955|UniProtKB=P50453	serine-type endopeptidase activity
GO:0005975	HUMAN|HGNC=8905|UniProtKB=P36871,HUMAN|HGNC=2422|UniProtKB=O75390,HUMAN|HGNC=10869|UniProtKB=Q92185	carbohydrate metabolic process
GO:0007416	HUMAN|HGNC=1117|UniProtKB=Q9UPA5,HUMAN|HGNC=15474|UniProtKB=Q9Y566,HUMAN|HGNC=13406|UniProtKB=Q9Y6V0,HUMAN|HGNC=14294|UniProtKB=Q9BYB0	synapse assembly
GO:0061136	HUMAN|HGNC=9568|UniProtKB=Q06323	regulation of proteasomal protein catabolic process
GO:0032451	HUMAN|HGNC=18039|UniProtKB=Q9UGL1	demethylase activity

GO:0005978	HUMAN|HGNC=4699|UniProtKB=P46976	glycogen biosynthetic process
PC00149	HUMAN|HGNC=910|UniProtKB=P25311,HUMAN|HGNC=4932|UniProtKB=P01889	major histocompatibility complex protein
GO:0097731	HUMAN|HGNC=26555|UniProtKB=Q3SY00	9+0 non-motile cilium
GO:0043406	HUMAN|HGNC=8761|UniProtKB=Q9BUL8	positive regulation of MAP kinase activity
GO:0019897	HUMAN|HGNC=15902|UniProtKB=Q8IXH8,HUMAN|HGNC=6207|UniProtKB=P14923,HUMAN|HGNC=14265|UniProtKB=Q86UP0	extrinsic component of plasma membrane
GO:0006919	HUMAN|HGNC=1050|UniProtKB=P55957	activation of cysteine-type endopeptidase activity involved in apoptotic process
GO:0050714	HUMAN|HGNC=1628|UniProtKB=P08571	positive regulation of protein secretion
GO:0016811	HUMAN|HGNC=25490|UniProtKB=Q96HA8,HUMAN|HGNC=735|UniProtKB=Q13510	hydrolase activity, acting on carbon-nitrogen (but not peptide) bonds, in linear amides
GO:0006508	HUMAN|HGNC=333|UniProtKB=P01019,HUMAN|HGNC=43788|UniProtKB=Q8NHM4,HUMAN|HGNC=2300|UniProtKB=Q96IY4,HUMAN|HGNC=8824|UniProtKB=P36955,HUMAN|HGNC=19986|UniProtKB=P99999,HUMAN|HGNC=7900|UniProtKB=P55786,HUMAN|HGNC=8942|UniProtKB=P48595,HUMAN|HGNC=3658|UniProtKB=Q9UGM5,HUMAN|HGNC=6383|UniProtKB=P01042,HUMAN|HGNC=20675|UniProtKB=Q96KN2,HUMAN|HGNC=775|UniProtKB=P01008,HUMAN|HGNC=1228|UniProtKB=P05155,HUMAN|HGNC=9758|UniProtKB=P62820,HUMAN|HGNC=9075|UniProtKB=P08697,HUMAN|HGNC=11583|UniProtKB=P05543,HUMAN|HGNC=8583|UniProtKB=P05121,HUMAN|HGNC=9495|UniProtKB=P24158,HUMAN|HGNC=3311|UniProtKB=P30740,HUMAN|HGNC=8993|UniProtKB=P12273,HUMAN|HGNC=8948|UniProtKB=P29622,HUMAN|HGNC=1546|UniProtKB=P50454,HUMAN|HGNC=8941|UniProtKB=P01009,HUMAN|HGNC=1540|UniProtKB=P08185,HUMAN|HGNC=4798|UniProtKB=Q14520,HUMAN|HGNC=24437|UniProtKB=Q96KP4,HUMAN|HGNC=1050|UniProtKB=P55957,HUMAN|HGNC=10569|UniProtKB=P29508,HUMAN|HGNC=1476|UniProtKB=P07384,HUMAN|HGNC=4838|UniProtKB=P05546,HUMAN|HGNC=3309|UniProtKB=P08246,HUMAN|HGNC=15722|UniProtKB=Q8WXQ8,HUMAN|HGNC=19388|UniProtKB=P49593,HUMAN|HGNC=16|UniProtKB=P01011,HUMAN|HGNC=18173|UniProtKB=Q9NZ08,HUMAN|HGNC=18370|UniProtKB=Q9H0U4,HUMAN|HGNC=8955|UniProtKB=P50453	proteolysis
GO:0006851	HUMAN|HGNC=23526|UniProtKB=Q8NE86	mitochondrial calcium ion transmembrane transport
GO:0035195	HUMAN|HGNC=29190|UniProtKB=Q9UPQ9,HUMAN|HGNC=20250|UniProtKB=Q96IF1	gene silencing by miRNA
GO:0007004	HUMAN|HGNC=5048|UniProtKB=Q00839	telomere maintenance via telomerase
GO:0051091	HUMAN|HGNC=30025|UniProtKB=Q5VV67	positive regulation of DNA-binding transcription factor activity

<table>
<tr><td>GO:0007193</td><td>HUMAN|HGNC=237|UniProtKB=O43306</td><td>adenylate cyclase-inhibiting G protein-coupled receptor signaling pathway</td></tr>
<tr><td>GO:0048598</td><td>HUMAN|HGNC=12652|UniProtKB=P50552,HUMAN|HGNC=10252|UniProtKB=O75116</td><td>embryonic morphogenesis</td></tr>
<tr><td>GO:0034142</td><td>HUMAN|HGNC=1628|UniProtKB=P08571</td><td>toll-like receptor 4 signaling pathway</td></tr>
<tr><td>GO:0003713</td><td>HUMAN|HGNC=19985|UniProtKB=P53999,HUMAN|HGNC=11957|UniProtKB=Q93074,HUMAN|HGNC=7133|UniProtKB=O14686,HUMAN|HGNC=17305|UniProtKB=Q9Y6J9,HUMAN|HGNC=6207|UniProtKB=P14923,HUMAN|HGNC=14451|UniProtKB=Q9BQK8,HUMAN|HGNC=12738|UniProtKB=Q969T9,HUMAN|HGNC=13726|UniProtKB=Q8NEZ4,HUMAN|HGNC=16272|UniProtKB=Q96JK9</td><td>transcription coactivator activity</td></tr>
<tr><td>GO:0032797</td><td>HUMAN|HGNC=30796|UniProtKB=Q9Y3F4</td><td>SMN complex</td></tr>
<tr><td>GO:0008360</td><td>HUMAN|HGNC=18756|UniProtKB=Q9BYZ6,HUMAN|HGNC=9802|UniProtKB=P15153,HUMAN|HGNC=9801|UniProtKB=P63000,HUMAN|HGNC=533|UniProtKB=P04083</td><td>regulation of cell shape</td></tr>
<tr><td>GO:0030218</td><td>HUMAN|HGNC=1504|UniProtKB=P42574</td><td>erythrocyte differentiation</td></tr>
<tr><td>GO:0048167</td><td>HUMAN|HGNC=15474|UniProtKB=Q9Y566,HUMAN|HGNC=14294|UniProtKB=Q9BYB0</td><td>regulation of synaptic plasticity</td></tr>
<tr><td>GO:0004867</td><td>HUMAN|HGNC=453|UniProtKB=P02760,HUMAN|HGNC=333|UniProtKB=P01019,HUMAN|HGNC=8583|UniProtKB=P05121,HUMAN|HGNC=3311|UniProtKB=P30740,HUMAN|HGNC=8948|UniProtKB=P29622,HUMAN|HGNC=11092|UniProtKB=P03973,HUMAN|HGNC=1546|UniProtKB=P50454,HUMAN|HGNC=8941|UniProtKB=P01009,HUMAN|HGNC=1540|UniProtKB=P08185,HUMAN|HGNC=8824|UniProtKB=P36955,HUMAN|HGNC=8942|UniProtKB=P48595,HUMAN|HGNC=10569|UniProtKB=P29508,HUMAN|HGNC=4838|UniProtKB=P05546,HUMAN|HGNC=16|UniProtKB=P01011,HUMAN|HGNC=775|UniProtKB=P01008,HUMAN|HGNC=1228|UniProtKB=P05155,HUMAN|HGNC=9075|UniProtKB=P08697,HUMAN|HGNC=11583|UniProtKB=P05543,HUMAN|HGNC=8955|UniProtKB=P50453</td><td>serine-type endopeptidase inhibitor activity</td></tr>
<tr><td>GO:0006570</td><td>HUMAN|HGNC=3579|UniProtKB=P16930</td><td>tyrosine metabolic process</td></tr>
<tr><td>GO:0001666</td><td>HUMAN|HGNC=20250|UniProtKB=Q96IF1</td><td>response to hypoxia</td></tr>
<tr><td>GO:0035303</td><td>HUMAN|HGNC=5461|UniProtKB=P78318</td><td>regulation of dephosphorylation</td></tr>
<tr><td>GO:0017075</td><td>HUMAN|HGNC=11446|UniProtKB=O00186,HUMAN|HGNC=11445|UniProtKB=Q15833,HUMAN|HGNC=12644|UniProtKB=Q15836</td><td>syntaxin-1 binding</td></tr>
<tr><td>GO:0046332</td><td>HUMAN|HGNC=6767|UniProtKB=Q15797</td><td>SMAD binding</td></tr>
<tr><td>GO:0031323</td><td>HUMAN|HGNC=3757|UniProtKB=O75955</td><td>regulation of cellular metabolic process</td></tr>
<tr><td>GO:0007015</td><td>HUMAN|HGNC=18756|UniProtKB=Q9BYZ6,HUMAN|HGNC=9801|UniProtKB=P63000,HUMAN|HGNC=17905|UniProtKB=Q9Y613,HUMAN|HGNC=2252|UniProtKB=P31146,HUMAN|HGNC=26161|UniProtKB=P57737,HUMAN|HGNC=12010|UniProtKB=P09493,HUMAN|HGNC=12012|UniProtKB=P06753,HUMAN|HGNC=9802|UniProtKB=P15153,HUMAN|HGNC=12013|UniProtKB=P67936,HUMAN|HGNC=12011|UniProtKB=P07951,HUMAN|HGNC=15878|UniProtKB</td><td>actin filament organization</td></tr>
</table>

<table>
<tr><td></td><td>=Q9NQ75,HUMAN|HGNC=2254|UniProtKB=Q9ULV4,HUMAN|HGNC=2253|UniProtKB=Q9BR76</td><td></td></tr>
<tr><td>GO:0019731</td><td>HUMAN|HGNC=11092|UniProtKB=P03973,HUMAN|HGNC=2761|UniProtKB=P59665,HUMAN|HGNC=11740|UniProtKB=P02787</td><td>antibacterial humoral response</td></tr>
<tr><td>GO:0006468</td><td>HUMAN|HGNC=10432|UniProtKB=P51812,HUMAN|HGNC=30410|UniProtKB=Q9BXA6,HUMAN|HGNC=19388|UniProtKB=P49593,HUMAN|HGNC=28164|UniProtKB=Q96M27,HUMAN|HGNC=19825|UniProtKB=Q6SA08,HUMAN|HGNC=29884|UniProtKB=Q8TF42,HUMAN|HGNC=9386|UniProtKB=Q9UGJ0,HUMAN|HGNC=18673|UniProtKB=Q96JB5,HUMAN|HGNC=24224|UniProtKB=Q9NYV4,HUMAN|HGNC=18994|UniProtKB=Q8TDC3,HUMAN|HGNC=11766|UniProtKB=P01137</td><td>protein phosphorylation</td></tr>
<tr><td>GO:0005758</td><td>HUMAN|HGNC=19986|UniProtKB=P99999</td><td>mitochondrial intermembrane space</td></tr>
<tr><td>GO:0048522</td><td>HUMAN|HGNC=26486|UniProtKB=Q6PJW8,HUMAN|HGNC=2479|UniProtKB=O76096,HUMAN|HGNC=4932|UniProtKB=P01889,HUMAN|HGNC=10534|UniProtKB=Q9NR31,HUMAN|HGNC=20250|UniProtKB=Q96IF1</td><td>positive regulation of cellular process</td></tr>
<tr><td>GO:0071108</td><td>HUMAN|HGNC=23077|UniProtKB=Q96FW1,HUMAN|HGNC=25648|UniProtKB=Q8N5J2</td><td>protein K48-linked deubiquitination</td></tr>
<tr><td>GO:0030971</td><td>HUMAN|HGNC=10840|UniProtKB=P29353</td><td>receptor tyrosine kinase binding</td></tr>
<tr><td>GO:0008333</td><td>HUMAN|HGNC=25153|UniProtKB=Q96EY5,HUMAN|HGNC=9788|UniProtKB=P51149</td><td>endosome to lysosome transport</td></tr>
<tr><td>GO:0060170</td><td>HUMAN|HGNC=19747|UniProtKB=Q86UK5</td><td>ciliary membrane</td></tr>
<tr><td>PC00198</td><td>HUMAN|HGNC=24056|UniProtKB=Q8NHP1,HUMAN|HGNC=1063|UniProtKB=P30043,HUMAN|HGNC=1548|UniProtKB=P16152,HUMAN|HGNC=5006|UniProtKB=P04035,HUMAN|HGNC=2873|UniProtKB=P00387,HUMAN|HGNC=7377|UniProtKB=Q9UJ68,HUMAN|HGNC=13397|UniProtKB=Q9UHQ9,HUMAN|HGNC=4376|UniProtKB=P36959,HUMAN|HGNC=12582|UniProtKB=P14927,HUMAN|HGNC=12590|UniProtKB=P07919,HUMAN|HGNC=4623|UniProtKB=P00390,HUMAN|HGNC=18985|UniProtKB=Q7Z4W1,HUMAN|HGNC=380|UniProtKB=P14550</td><td>reductase</td></tr>
<tr><td>GO:0009897</td><td>HUMAN|HGNC=5575|UniProtKB=A0A0B4J1V2,HUMAN|HGNC=5645|UniProtKB=A0A0C4DH34,HUMAN|HGNC=4932|UniProtKB=P01889,HUMAN|HGNC=5549|UniProtKB=A0A0C4DH31,HUMAN|HGNC=5554|UniProtKB=P01743,HUMAN|HGNC=5480|UniProtKB=P01880,HUMAN|HGNC=5659|UniProtKB=A0A0C4DH38,HUMAN|HGNC=5634|UniProtKB=A0A075B7F0,HUMAN|HGNC=38476|UniProtKB=B9A064,HUMAN|HGNC=5861|UniProtKB=A0M8Q6,HUMAN|HGNC=5647|UniProtKB=A0A087WSY4,HUMAN|HGNC=5563|UniProtKB=A0A075B7D0,HUMAN|HGNC=5856|UniProtKB=P0DOY2,HUMAN|HGNC=5606|UniProtKB=P01763,HUMAN|HGNC=199|UniProtKB=O43506,HUMAN|HGNC=5576|UniProtKB=P01817,HUMAN|HGNC=5528|UniProtKB=P01861,HUMAN|HGNC=5541|UniProtKB=P01871,HUMAN|HGNC=4244|UniProtKB=O00451,HUMAN|HGNC=5585|UniProtKB=A0A0C4DH32,HUMAN|HGNC=4243|UniProtKB=P56159,HUMAN|HGNC=5551|UniProtKB=A0A0C4DH33,HUMAN|HGNC=5526|UniProtKB=P01859,HUMAN|HGNC=1628|UniProtKB=P08571,HUMAN|HGNC=5657|UniProtKB=P0DP08,HUMAN|Ensembl=ENSG00000282639|UniProtKB=A0A0J9YX35,HUMAN|Gene=HV309_HUMAN|UniProtKB=P01782,HUMAN|HGNC=5654|UniProtKB=P0182</td><td>external side of plasma membrane</td></tr>
</table>

<table>
<tr><th></th><th></th><th></th></tr>
<tr><td></td><td>5,HUMAN|HGNC=5553|UniProtKB=A0A0A0MS14,HUMAN|HGNC=19100|UniProtKB=Q5VWK5,HUMAN|Gene=HV108_HUMAN|UniProtKB=P0DP01,HUMAN|HGNC=5624|UniProtKB=A0A0B4J1X5,HUMAN|HGNC=5620|UniProtKB=P01780,HUMAN|HGNC=5598|UniProtKB=A0A0C4DH35,HUMAN|HGNC=11763|UniProtKB=P02786,HUMAN|HGNC=5580|UniProtKB=P01762,HUMAN|HGNC=5633|UniProtKB=A0A075B7D8,HUMAN|HGNC=5582|UniProtKB=A0A0B4J1V0,HUMAN|HGNC=5478|UniProtKB=P01876,HUMAN|HGNC=5617|UniProtKB=A0A075B6Q5,HUMAN|HGNC=910|UniProtKB=P25311,HUMAN|HGNC=5594|UniProtKB=P0DP03,HUMAN|HGNC=5655|UniProtKB=A0A0C4DH41,HUMAN|HGNC=5636|UniProtKB=A0A075B7B8,HUMAN|HGNC=5586|UniProtKB=A0A0B4J1V1,HUMAN|HGNC=5581|UniProtKB=P01766,HUMAN|HGNC=5622|UniProtKB=A0A0B4J1Y9,HUMAN|HGNC=5652|UniProtKB=A0A075B6R2,HUMAN|HGNC=5593|UniProtKB=P0DP02,HUMAN|HGNC=5479|UniProtKB=P01877,HUMAN|HGNC=5619|UniProtKB=A0A0C4DH42,HUMAN|HGNC=5596|UniProtKB=P01772,HUMAN|HGNC=5550|UniProtKB=P23083,HUMAN|HGNC=5604|UniProtKB=A0A0B4J1X8,HUMAN|HGNC=5525|UniProtKB=P01857,HUMAN|HGNC=5662|UniProtKB=A0A0B4J1U7,HUMAN|HGNC=16961|UniProtKB=Q15762,HUMAN|HGNC=5601|UniProtKB=A0A0C4DH36,HUMAN|HGNC=5716|UniProtKB=P01834,HUMAN|HGNC=5588|UniProtKB=P01764,HUMAN|HGNC=5857|UniProtKB=P0DOY3,HUMAN|HGNC=5610|UniProtKB=P01767,HUMAN|Ensembl=ENSG00000282150|UniProtKB=A0A0J9YY99,HUMAN|HGNC=5527|UniProtKB=P01860</td><td></td></tr>
<tr><td>GO:0097650</td><td>HUMAN|HGNC=8898|UniProtKB=P07205</td><td>B axonemal microtubule</td></tr>
<tr><td>GO:0051225</td><td>HUMAN|HGNC=6891|UniProtKB=Q15555,HUMAN|HGNC=6890|UniProtKB=Q15691</td><td>spindle assembly</td></tr>
<tr><td>GO:0005622</td><td>HUMAN|HGNC=9391|UniProtKB=P13861,HUMAN|HGNC=9392|UniProtKB=P31323</td><td>intracellular</td></tr>
<tr><td>PC00260</td><td>HUMAN|HGNC=10432|UniProtKB=P51812,HUMAN|HGNC=30178|UniProtKB=Q9Y570,HUMAN|HGNC=8575|UniProtKB=P68402,HUMAN|HGNC=8127|UniProtKB=O15294</td><td>protein modifying enzyme</td></tr>
<tr><td>GO:0007179</td><td>HUMAN|HGNC=6767|UniProtKB=Q15797,HUMAN|HGNC=23688|UniProtKB=Q86UU0,HUMAN|HGNC=13200|UniProtKB=Q15942,HUMAN|HGNC=11766|UniProtKB=P01137</td><td>transforming growth factor beta receptor signaling pathway</td></tr>
<tr><td>PC00146</td><td>HUMAN|HGNC=9207|UniProtKB=Q9UFF9</td><td>mRNA polyadenylation factor</td></tr>
<tr><td>GO:1901888</td><td>HUMAN|HGNC=3757|UniProtKB=O75955,HUMAN|HGNC=10252|UniProtKB=O75116</td><td>regulation of cell junction assembly</td></tr>
<tr><td>GO:0045582</td><td>HUMAN|HGNC=533|UniProtKB=P04083</td><td>positive regulation of T cell differentiation</td></tr>
<tr><td>GO:0071417</td><td>HUMAN|HGNC=26068|UniProtKB=Q6IAA8,HUMAN|HGNC=9855|UniProtKB=P62834,HUMAN|HGNC=17955|UniProtKB=O43504,HUMAN|HGNC=9857|UniProtKB=P61224,HUMAN|HGNC=15606|UniProtKB=Q9UHA4</td><td>cellular response to organonitrogen compound</td></tr>
<tr><td>GO:0060255</td><td>HUMAN|HGNC=3757|UniProtKB=O75955</td><td>regulation of macromolecule metabolic process</td></tr>
<tr><td>GO:0016051</td><td>HUMAN|HGNC=8898|UniProtKB=P07205,HUMAN|HGNC=4458|UniProtKB=P06744,HUMAN|HGNC=12009|UniProtKB=P60174</td><td>carbohydrate biosynthetic process</td></tr>
<tr><td>GO:0051336</td><td>HUMAN|HGNC=8087|UniProtKB=P29728</td><td>regulation of hydrolase activity</td></tr>
</table>

GO:1902533	HUMAN|HGNC=1050|UniProtKB=P55957	positive regulation of intracellular signal transduction
GO:0030490	HUMAN|HGNC=12755|UniProtKB=Q9UNX4	maturation of SSU-rRNA
GO:0042277	HUMAN|HGNC=4624|UniProtKB=P48637,HUMAN|HGNC=7900|UniProtKB=P55786,HUMAN|HGNC=3537|UniProtKB=P25116,HUMAN|HGNC=18173|UniProtKB=Q9NZ08	peptide binding
GO:0031589	HUMAN|HGNC=12726|UniProtKB=P04275	cell-substrate adhesion
GO:0044255	HUMAN|HGNC=253|UniProtKB=P11766,HUMAN|HGNC=251|UniProtKB=P00326,HUMAN|HGNC=255|UniProtKB=P28332	cellular lipid metabolic process
GO:0043296	HUMAN|HGNC=16051|UniProtKB=Q8TEW0	apical junction complex
GO:0000123	HUMAN|HGNC=25473|UniProtKB=Q9P2N6,HUMAN|HGNC=25544|UniProtKB=Q9HCI7	histone acetyltransferase complex
GO:0030204	HUMAN|HGNC=24293|UniProtKB=Q70JA7,HUMAN|HGNC=18144|UniProtKB=Q8IZU8	chondroitin sulfate metabolic process
PC00191	HUMAN|HGNC=6169|UniProtKB=Q14624,HUMAN|HGNC=333|UniProtKB=P01019,HUMAN|HGNC=11092|UniProtKB=P03973,HUMAN|HGNC=2482|UniProtKB=P04080,HUMAN|HGNC=8824|UniProtKB=P36955,HUMAN|HGNC=8630|UniProtKB=P30086,HUMAN|HGNC=620|UniProtKB=P05067,HUMAN|HGNC=8942|UniProtKB=P48595,HUMAN|HGNC=3658|UniProtKB=Q9UGM5,HUMAN|HGNC=11820|UniProtKB=P01033,HUMAN|HGNC=6383|UniProtKB=P01042,HUMAN|HGNC=6167|UniProtKB=P19823,HUMAN|HGNC=775|UniProtKB=P01008,HUMAN|HGNC=1228|UniProtKB=P05155,HUMAN|HGNC=9075|UniProtKB=P08697,HUMAN|HGNC=21449|UniProtKB=Q86UX2,HUMAN|HGNC=1324|UniProtKB=P0C0L5,HUMAN|HGNC=1318|UniProtKB=P01024,HUMAN|HGNC=21685|UniProtKB=Q6YHK3,HUMAN|HGNC=11583|UniProtKB=P05543,HUMAN|HGNC=1331|UniProtKB=P01031,HUMAN|HGNC=8583|UniProtKB=P05121,HUMAN|HGNC=3311|UniProtKB=P30740,HUMAN|HGNC=8948|UniProtKB=P29622,HUMAN|HGNC=9750|UniProtKB=P20742,HUMAN|HGNC=1546|UniProtKB=P50454,HUMAN|HGNC=598|UniProtKB=Q06481,HUMAN|HGNC=8941|UniProtKB=P01009,HUMAN|HGNC=1540|UniProtKB=P08185,HUMAN|HGNC=10569|UniProtKB=P29508,HUMAN|HGNC=4838|UniProtKB=P05546,HUMAN|HGNC=6168|UniProtKB=Q06033,HUMAN|HGNC=9571|UniProtKB=Q92530,HUMAN|HGNC=16|UniProtKB=P01011,HUMAN|HGNC=8955|UniProtKB=P50453	protease inhibitor
PC00203	HUMAN|HGNC=586|UniProtKB=P13798,HUMAN|HGNC=1247|UniProtKB=P09871,HUMAN|HGNC=43788|UniProtKB=Q8NHM4,HUMAN|HGNC=2532|UniProtKB=P08311,HUMAN|HGNC=3528|UniProtKB=P00742,HUMAN|HGNC=5156|UniProtKB=P00739,HUMAN|HGNC=6902|UniProtKB=O00187,HUMAN|HGNC=3551|UniProtKB=P00740,HUMAN|HGNC=9451|UniProtKB=P04070,HUMAN|HGNC=9495|UniProtKB=P24158,HUMAN|HGNC=7380|UniProtKB=P26927,HUMAN|HGNC=9469|UniProtKB=O14668,HUMAN|HGNC=8569|UniProtKB=P29122,HUMAN|HGNC=913|UniProtKB=P20160,HUMAN|HGNC=12016|UniProtKB=P29144,HUMAN|HGNC=3465|UniProtKB=P10768,HUMAN|HGNC=8738|UniProtKB=Q15113,HUMAN|HGNC=4798|UniProtKB=Q14520,HUMAN|HGNC=3530|UniProtKB=P00748,HUMAN|HGNC=5141|UniProtKB=P00738,HUMAN|HGNC=3309	serine protease

	|UniProtKB=P08246,HUMAN|HGNC=21265|UniProtKB=Q9NZP8,HUMAN|HGNC=13921|UniProtKB=O95870,HUMAN|HGNC=6901|UniProtKB=P48740,HUMAN|HGNC=9071|UniProtKB=P00747	
GO:0010824	HUMAN|HGNC=7910|UniProtKB=P06748	regulation of centrosome duplication
GO:0045727	HUMAN|HGNC=3300|UniProtKB=P63241,HUMAN|HGNC=1243|UniProtKB=Q07021,HUMAN|Gene=EIF2S3L|UniProtKB=Q2VIR3	positive regulation of translation
GO:0018105	HUMAN|HGNC=1459|UniProtKB=Q14012,HUMAN|HGNC=9414|UniProtKB=Q13976,HUMAN|HGNC=9406|UniProtKB=Q16513,HUMAN|HGNC=9380|UniProtKB=P17612,HUMAN|HGNC=12401|UniProtKB=P33981,HUMAN|HGNC=9381|UniProtKB=P22694,HUMAN|HGNC=9395|UniProtKB=P05771,HUMAN|HGNC=1745|UniProtKB=O00311	peptidyl-serine phosphorylation
GO:0030660	HUMAN|HGNC=25951|UniProtKB=Q6ULP2,HUMAN|HGNC=555|UniProtKB=O43747	Golgi-associated vesicle membrane
GO:0051897	HUMAN|HGNC=6091|UniProtKB=P06213	positive regulation of protein kinase B signaling
GO:0042274	HUMAN|HGNC=7910|UniProtKB=P06748	ribosomal small subunit biogenesis
GO:0051233	HUMAN|HGNC=6891|UniProtKB=Q15555,HUMAN|HGNC=6890|UniProtKB=Q15691	spindle midzone
GO:0099536	HUMAN|HGNC=20589|UniProtKB=Q9P0J7	synaptic signaling
GO:0003714	HUMAN|HGNC=30747|UniProtKB=P61201,HUMAN|HGNC=5270|UniProtKB=P25685,HUMAN|HGNC=5048|UniProtKB=Q00839,HUMAN|HGNC=20250|UniProtKB=Q96IF1	transcription corepressor activity
PC00259	HUMAN|HGNC=19985|UniProtKB=P53999,HUMAN|HGNC=11957|UniProtKB=Q93074,HUMAN|HGNC=11617|UniProtKB=Q15369,HUMAN|HGNC=23074|UniProtKB=Q9NX70,HUMAN|HGNC=17305|UniProtKB=Q9Y6J9,HUMAN|HGNC=11619|UniProtKB=Q15370	general transcription factor
GO:0060236	HUMAN|HGNC=5048|UniProtKB=Q00839	regulation of mitotic spindle organization
GO:0000963	HUMAN|HGNC=26022|UniProtKB=Q7L0Y3	mitochondrial RNA processing
GO:0036157	HUMAN|HGNC=8898|UniProtKB=P07205,HUMAN|HGNC=2952|UniProtKB=Q96JB1	outer dynein arm
GO:0006783	HUMAN|HGNC=871|UniProtKB=Q9UII2,HUMAN|HGNC=395|UniProtKB=P13716	heme biosynthetic process
GO:0017156	HUMAN|HGNC=11510|UniProtKB=Q8N9I0,HUMAN|HGNC=11512|UniProtKB=Q9H2B2	calcium-ion regulated exocytosis
GO:0005104	HUMAN|HGNC=15527|UniProtKB=Q86Z14	fibroblast growth factor receptor binding
GO:0008201	HUMAN|HGNC=2209|UniProtKB=P20908,HUMAN|HGNC=11085|UniProtKB=O75093	heparin binding
GO:0002696	HUMAN|HGNC=6517|UniProtKB=P18428,HUMAN|HGNC=16961|UniProtKB=Q15762	positive regulation of leukocyte activation
GO:0005518	HUMAN|HGNC=6153|UniProtKB=P05556,HUMAN|HGNC=11219|UniProtKB=P09486	collagen binding
GO:0071011	HUMAN|HGNC=20471|UniProtKB=O95777,HUMAN|HGNC=6038|UniProtKB=Q12906	precatalytic spliceosome
GO:0022839	HUMAN|HGNC=22222|UniProtKB=Q9C0H2	ion gated channel activity
GO:0042981	HUMAN|HGNC=6766|UniProtKB=Q8WXG6,HUMAN|HGNC=7850|UniProtKB=P22392	regulation of apoptotic process

GO:0010628	HUMAN|HGNC=27424|UniProtKB=Q5T481	positive regulation of gene expression
GO:0005272	HUMAN|HGNC=10594|UniProtKB=Q01118,HUMAN|HGNC=10582|UniProtKB=Q9Y5Y9	sodium channel activity
GO:0010518	HUMAN|HGNC=4381|UniProtKB=Q14344	positive regulation of phospholipase activity
GO:0006890	HUMAN|HGNC=9786|UniProtKB=P20340,HUMAN|HGNC=2230|UniProtKB=P53621,HUMAN|HGNC=14902|UniProtKB=Q9NRW1,HUMAN|HGNC=649|UniProtKB=P48444,HUMAN|HGNC=2232|UniProtKB=P35606	retrograde vesicle-mediated transport, Golgi to endoplasmic reticulum
GO:0001655	HUMAN|HGNC=533|UniProtKB=P04083	urogenital system development
PC00124	HUMAN|HGNC=3616|UniProtKB=P12318,HUMAN|HGNC=5|UniProtKB=P04217,HUMAN|HGNC=8823|UniProtKB=P16284,HUMAN|HGNC=3611|UniProtKB=P30273,HUMAN|HGNC=8968|UniProtKB=P01833,HUMAN|HGNC=1704|UniProtKB=Q9UIB8,HUMAN|HGNC=14388|UniProtKB=Q9HCN6,HUMAN|HGNC=20434|UniProtKB=Q86YW5	immunoglobulin receptor superfamily
GO:0140014	HUMAN|HGNC=12407|UniProtKB=P68366,HUMAN|HGNC=2712|UniProtKB=Q13561,HUMAN|HGNC=12410|UniProtKB=Q9NY65,HUMAN|HGNC=12666|UniProtKB=P55072,HUMAN|HGNC=20778|UniProtKB=P07437,HUMAN|HGNC=28959|UniProtKB=Q14008,HUMAN|HGNC=533|UniProtKB=P04083,HUMAN|HGNC=20766|UniProtKB=Q71U36,HUMAN|HGNC=20774|UniProtKB=P04350,HUMAN|HGNC=18673|UniProtKB=Q96JB5,HUMAN|HGNC=20771|UniProtKB=P68371,HUMAN|HGNC=20063|UniProtKB=Q86T82,HUMAN|HGNC=10252|UniProtKB=O75116,HUMAN|HGNC=9847|UniProtKB=P43487,HUMAN|HGNC=9308|UniProtKB=Q15257,HUMAN|HGNC=16257|UniProtKB=Q9H4B7,HUMAN|HGNC=9568|UniProtKB=Q06323,HUMAN|HGNC=7547|UniProtKB=P10243,HUMAN|HGNC=2092|UniProtKB=Q00610	mitotic nuclear division
GO:0017016	HUMAN|HGNC=9847|UniProtKB=P43487	Ras GTPase binding
GO:0038202	HUMAN|HGNC=17955|UniProtKB=O43504	TORC1 signaling
PC00077	HUMAN|HGNC=31797|UniProtKB=P0C7V6,HUMAN|HGNC=7106|UniProtKB=P54252,HUMAN|HGNC=10760|UniProtKB=Q01105,HUMAN|HGNC=7640|UniProtKB=Q99733,HUMAN|HGNC=1859|UniProtKB=Q9BV73,HUMAN|HGNC=42937|UniProtKB=P0DME0,HUMAN|HGNC=24358|UniProtKB=Q9H2G4	chromatin/chromatin-binding, or -regulatory protein
GO:0050661	HUMAN|HGNC=7863|UniProtKB=Q13423,HUMAN|HGNC=8891|UniProtKB=P52209	NADP binding
GO:0051087	HUMAN|HGNC=5269|UniProtKB=P61604,HUMAN|HGNC=5261|UniProtKB=P10809,HUMAN|HGNC=10519|UniProtKB=Q9NZJ4,HUMAN|HGNC=4926|UniProtKB=O15212,HUMAN|HGNC=1735|UniProtKB=Q16543,HUMAN|HGNC=5270|UniProtKB=P25685	chaperone binding
GO:0006633	HUMAN|HGNC=24175|UniProtKB=Q9P035	fatty acid biosynthetic process
GO:0005765	HUMAN|HGNC=6499|UniProtKB=P11279,HUMAN|HGNC=6501|UniProtKB=P13473,HUMAN|HGNC=25564|UniProtKB=Q9NVJ2	lysosomal membrane
GO:0030162	HUMAN|HGNC=7732|UniProtKB=Q15843	regulation of proteolysis
GO:0006334	HUMAN|HGNC=4747|UniProtKB=P58876,HUMAN|HGNC=4750|UniProtKB=Q99879,HUMAN|HGNC=13954|UniProtKB=O60814,HU	nucleosome assembly

	MAN|HGNC=4762|UniProtKB=P57053,HUMAN|HGNC=4746|UniProtKB=P62807,HUMAN|HGNC=20514|UniProtKB=Q8N257,HUMAN|HGNC=4755|UniProtKB=Q93079,HUMAN|HGNC=7640|UniProtKB=Q99733,HUMAN|HGNC=24700|UniProtKB=Q5QNW6,HUMAN|HGNC=4749|UniProtKB=Q99877	
GO:0002250	HUMAN|HGNC=6171|UniProtKB=Q08881,HUMAN|HGNC=11491|UniProtKB=P43405	adaptive immune response
GO:0046777	HUMAN|HGNC=6171|UniProtKB=Q08881,HUMAN|HGNC=4037|UniProtKB=P06241,HUMAN|HGNC=12841|UniProtKB=P07947,HUMAN|HGNC=11283|UniProtKB=P12931,HUMAN|HGNC=6735|UniProtKB=P07948	protein autophosphorylation
GO:0031201	HUMAN|HGNC=11442|UniProtKB=O15400,HUMAN|HGNC=7641|UniProtKB=P54920,HUMAN|HGNC=11429|UniProtKB=O75558,HUMAN|HGNC=12644|UniProtKB=Q15836,HUMAN|HGNC=11131|UniProtKB=O00161,HUMAN|HGNC=12647|UniProtKB=Q9BV40	SNARE complex
GO:2000311	HUMAN|HGNC=1406|UniProtKB=Q9Y698,HUMAN|HGNC=15474|UniProtKB=Q9Y566,HUMAN|HGNC=14294|UniProtKB=Q9BYB0	regulation of AMPA receptor activity
GO:0060348	HUMAN|HGNC=13937|UniProtKB=O95866,HUMAN|HGNC=544|UniProtKB=P08133,HUMAN|HGNC=11283|UniProtKB=P12931	bone development
PC00032	HUMAN|HGNC=18018|UniProtKB=Q8IX18,HUMAN|HGNC=15861|UniProtKB=Q9H5Z1,HUMAN|HGNC=17196|UniProtKB=Q8NG08	RNA helicase
GO:0006952	HUMAN|HGNC=11364|UniProtKB=P40763	defense response
PC00182	HUMAN|HGNC=9308|UniProtKB=Q15257	phosphatase activator
PC00009	HUMAN|HGNC=23168|UniProtKB=Q8IYD8,HUMAN|HGNC=10898|UniProtKB=Q15477,HUMAN|HGNC=7127|UniProtKB=P40692,HUMAN|HGNC=19246|UniProtKB=Q9NYB0,HUMAN|HGNC=9969|UniProtKB=P35251,HUMAN|HGNC=14060|UniProtKB=Q9UBZ9,HUMAN|HGNC=7326|UniProtKB=P20585,HUMAN|HGNC=17177|UniProtKB=Q8IUI8,HUMAN|HGNC=11317|UniProtKB=Q04837	DNA metabolism protein
GO:0007019	HUMAN|HGNC=6835|UniProtKB=P78559,HUMAN|HGNC=6510|UniProtKB=P16949	microtubule depolymerization
GO:0030163	HUMAN|HGNC=6501|UniProtKB=P13473,HUMAN|HGNC=11820|UniProtKB=P01033,HUMAN|HGNC=6737|UniProtKB=O75608,HUMAN|HGNC=613|UniProtKB=P02649	protein catabolic process
GO:0033554	HUMAN|HGNC=17169|UniProtKB=Q13162,HUMAN|HGNC=5046|UniProtKB=P52272	cellular response to stress
GO:1990719	HUMAN|HGNC=8898|UniProtKB=P07205	C1 axonemal microtubule
GO:0042110	HUMAN|HGNC=5261|UniProtKB=P10809	T cell activation
GO:0009755	HUMAN|HGNC=9866|UniProtKB=P13631,HUMAN|HGNC=3964|UniProtKB=P01225,HUMAN|HGNC=16452|UniProtKB=P0DN86,HUMAN|HGNC=16721|UniProtKB=A6NKQ9	hormone-mediated signaling pathway
GO:0005254	HUMAN|HGNC=2062|UniProtKB=O00299,HUMAN|HGNC=25240|UniProtKB=Q4KMQ2,HUMAN|HGNC=13518|UniProtKB=Q9Y696,HUMAN|HGNC=22222|UniProtKB=Q9C0H2	chloride channel activity
GO:0046883	HUMAN|HGNC=533|UniProtKB=P04083	regulation of hormone secretion
GO:0046578	HUMAN|HGNC=6766|UniProtKB=Q8WXG6	regulation of Ras protein signal transduction
GO:0042391	HUMAN|HGNC=15474|UniProtKB=Q9Y566,HUMAN|HGNC=14294|UniProtKB=Q9BYB0	regulation of membrane potential

GO:0060401 HUMAN|HGNC=23526|UniProtKB=Q8NE86 cytosolic calcium ion transport

GO:0032991 HUMAN|HGNC=5575|UniProtKB=A0A0B4J1V2,HUMAN|HGNC=9391|UniProtKB=P13861,HUMAN|HGNC=9112|UniProtKB=Q6P1K2,HUMAN|HGNC=9414|UniProtKB=Q13976,HUMAN|HGNC=5645|UniProtKB=A0A0C4DH34,HUMAN|HGNC=5549|UniProtKB=A0A0C4DH31,HUMAN|HGNC=5554|UniProtKB=P01743,HUMAN|HGNC=9411|UniProtKB=P14314,HUMAN|HGNC=9392|UniProtKB=P31323,HUMAN|HGNC=5480|UniProtKB=P01880,HUMAN|HGNC=5659|UniProtKB=A0A0C4DH38,HUMAN|HGNC=5634|UniProtKB=A0A075B7F0,HUMAN|HGNC=23845|UniProtKB=Q8IY92,HUMAN|HGNC=4831|UniProtKB=P69891,HUMAN|HGNC=38476|UniProtKB=B9A064,HUMAN|HGNC=11358|UniProtKB=O75886,HUMAN|HGNC=11126|UniProtKB=P53814,HUMAN|HGNC=5861|UniProtKB=A0M8Q6,HUMAN|HGNC=7729|UniProtKB=Q15019,HUMAN|HGNC=5647|UniProtKB=A0A087WSY4,HUMAN|HGNC=5563|UniProtKB=A0A075B7D0,HUMAN|HGNC=5856|UniProtKB=P0DOY2,HUMAN|HGNC=5606|UniProtKB=P01763,HUMAN|HGNC=2067|UniProtKB=O00151,HUMAN|HGNC=4622|UniProtKB=Q8IYD1,HUMAN|HGNC=4621|UniProtKB=P15170,HUMAN|HGNC=5576|UniProtKB=P01817,HUMAN|HGNC=5528|UniProtKB=P01861,HUMAN|HGNC=5541|UniProtKB=P01871,HUMAN|Gene=EIF2S3L|UniProtKB=Q2VIR3,HUMAN|HGNC=5585|UniProtKB=A0A0C4DH32,HUMAN|HGNC=2678|UniProtKB=P14868,HUMAN|HGNC=5551|UniProtKB=A0A0C4DH33,HUMAN|HGNC=17468|UniProtKB=Q96HC4,HUMAN|HGNC=5526|UniProtKB=P01859,HUMAN|HGNC=22958|UniProtKB=Q9NR12,HUMAN|HGNC=5657|UniProtKB=P0DP08,HUMAN|Ensembl=ENSG00000282639|UniProtKB=A0A0J9YX35,HUMAN|HGNC=3297|UniProtKB=P78344,HUMAN|Gene=HV309_HUMAN|UniProtKB=P01782,HUMAN|HGNC=15848|UniProtKB=Q14141,HUMAN|HGNC=5654|UniProtKB=P01825,HUMAN|HGNC=4926|UniProtKB=O15212,HUMAN|HGNC=12733|UniProtKB=Q9Y6W5,HUMAN|HGNC=5553|UniProtKB=A0A0A0MS14,HUMAN|HGNC=9380|UniProtKB=P17612,HUMAN|Gene=HV108_HUMAN|UniProtKB=P0DP01,HUMAN|HGNC=1488|UniProtKB=P52907,HUMAN|HGNC=5624|UniProtKB=A0A0B4J1X5,HUMAN|HGNC=5620|UniProtKB=P01780,HUMAN|HGNC=5598|UniProtKB=A0A0C4DH35,HUMAN|HGNC=603|UniProtKB=P04114,HUMAN|HGNC=5580|UniProtKB=P01762,HUMAN|HGNC=5633|UniProtKB=A0A075B7D8,HUMAN|HGNC=5582|UniProtKB=A0A0B4J1V0,HUMAN|HGNC=5478|UniProtKB=P01876,HUMAN|HGNC=18423|UniProtKB=O75140,HUMAN|HGNC=5617|UniProtKB=A0A075B6Q5,HUMAN|HGNC=10898|UniProtKB=Q15477,HUMAN|HGNC=13759|UniProtKB=Q7L576,HUMAN|HGNC=5594|UniProtKB=P0DP03,HUMAN|HGNC=1717|UniProtKB=Q16181,HUMAN|HGNC=5655|UniProtKB=A0A0C4DH41,HUMAN|HGNC=5238|UniProtKB=P11021,HUMAN|HGNC=5636|UniProtKB=A0A075B7B8,HUMAN|HGNC=5586|UniProtKB=A0A0B4J1V1,HUMAN|HGNC=5581|UniProtKB=P01766,HUMAN|HGNC=11357|UniProtKB=Q92783,HUMAN|HGNC=5622|UniProtKB=A0A0B4J1Y9,HUMAN|HGNC=613|UniProtKB=P02649,HUMAN|HGNC=5652|UniProtKB=A0A075B6R2,HUMAN|HGNC=5593|UniProtKB=P0DP02,HUMAN|HGNC=9381|UniProtKB=P22694,HUMAN|HGNC=5479|UniProtKB=P01877,HUMAN|HGNC=5619|UniProtKB=A0A0C4DH42,HUMAN|HGNC=5596|UniProtKB=P01772,HUMAN|HGNC=8868|UniProtKB=Q9NQP4,HUMAN|HGNC=5550|U protein-containing complex

GO:1901565	niProtKB=P23083,HUMAN|HGNC=8761|UniProtKB=Q9BUL8,HUMAN|HGNC=5604|UniProtKB=A0A0B4J1X8,HUMAN|HGNC=4823|UniProtKB=P69905,HUMAN|HGNC=4829|UniProtKB=P02042,HUMAN|HGNC=5525|UniProtKB=P01857,HUMAN|HGNC=5662|UniProtKB=A0A0B4J1U7,HUMAN|HGNC=5601|UniProtKB=A0A0C4DH36,HUMAN|HGNC=5716|UniProtKB=P01834,HUMAN|HGNC=4827|UniProtKB=P68871,HUMAN|HGNC=5588|UniProtKB=P01764,HUMAN|HGNC=5857|UniProtKB=P0DOY3,HUMAN|HGNC=9164|UniProtKB=Q99719,HUMAN|HGNC=5610|UniProtKB=P01767,HUMAN|HGNC=13919|UniProtKB=P46379,HUMAN|Ensembl=ENSG00000282150|UniProtKB=A0A0J9YY99,HUMAN|HGNC=5527|UniProtKB=P01860,HUMAN|HGNC=1491|UniProtKB=P47756 HUMAN|HGNC=7900|UniProtKB=P55786,HUMAN|HGNC=13921|UniProtKB=O95870,HUMAN|HGNC=18173|UniProtKB=Q9NZ08,HUMAN|HGNC=2689|UniProtKB=P09172	organonitrogen compound catabolic process
GO:0046651	HUMAN|HGNC=4861|UniProtKB=Q9NRZ9	lymphocyte proliferation
GO:0003924	HUMAN|HGNC=3189|UniProtKB=P68104,HUMAN|HGNC=9862|UniProtKB=P61225,HUMAN|HGNC=9784|UniProtKB=P61020,HUMAN|HGNC=19983|UniProtKB=Q86YS6,HUMAN|HGNC=6407|UniProtKB=P01116,HUMAN|HGNC=3200|UniProtKB=Q5VTE0,HUMAN|HGNC=17102|UniProtKB=Q92619,HUMAN|HGNC=667|UniProtKB=P61586,HUMAN|HGNC=9855|UniProtKB=P62834,HUMAN|HGNC=2973|UniProtKB=O00429,HUMAN|HGNC=9846|UniProtKB=P62826,HUMAN|HGNC=9840|UniProtKB=P11234,HUMAN|HGNC=4393|UniProtKB=P11488,HUMAN|HGNC=7729|UniProtKB=Q15019,HUMAN|HGNC=4395|UniProtKB=P19086,HUMAN|HGNC=9785|UniProtKB=P51148,HUMAN|HGNC=4384|UniProtKB=P63096,HUMAN|HGNC=9772|UniProtKB=Q13637,HUMAN|HGNC=4622|UniProtKB=Q8IYD1,HUMAN|HGNC=9801|UniProtKB=P63000,HUMAN|HGNC=4621|UniProtKB=P15170,HUMAN|HGNC=669|UniProtKB=P08134,HUMAN|HGNC=10447|UniProtKB=P10301,HUMAN|HGNC=11231|UniProtKB=Q8WXF7,HUMAN|HGNC=24526|UniProtKB=Q6DD88,HUMAN|HGNC=25694|UniProtKB=Q8TC07,HUMAN|HGNC=9802|UniProtKB=P15153,HUMAN|HGNC=3214|UniProtKB=P13639,HUMAN|HGNC=4381|UniProtKB=Q14344,HUMAN|HGNC=25571|UniProtKB=Q9NVG8,HUMAN|HGNC=21035|UniProtKB=Q8N392,HUMAN|HGNC=15848|UniProtKB=Q14141,HUMAN|HGNC=18756|UniProtKB=Q9BYZ6,HUMAN|HGNC=2720|UniProtKB=Q9ULH1,HUMAN|HGNC=14902|UniProtKB=Q9NRW1,HUMAN|HGNC=4387|UniProtKB=P08754,HUMAN|HGNC=25361|UniProtKB=Q8N264,HUMAN|HGNC=4227|UniProtKB=P50395,HUMAN|HGNC=10701|UniProtKB=Q15436,HUMAN|HGNC=18423|UniProtKB=O75140,HUMAN|HGNC=16524|UniProtKB=P61106,HUMAN|HGNC=29351|UniProtKB=Q9C0H5,HUMAN|HGNC=1717|UniProtKB=Q16181,HUMAN|HGNC=9758|UniProtKB=P62820,HUMAN|HGNC=18263|UniProtKB=Q9UL25,HUMAN|HGNC=678|UniProtKB=P52565,HUMAN|HGNC=6111|UniProtKB=Q13576,HUMAN|HGNC=4226|UniProtKB=P31150,HUMAN|HGNC=2974|UniProtKB=P50570,HUMAN|HGNC=9857|UniProtKB=P61224,HUMAN|HGNC=679|UniProtKB=P52566,HUMAN|HGNC=4385|UniProtKB=P04899,HUMAN|HGNC=4390|UniProtKB=P50148,HUMAN|HGNC=9786|UniProtKB=P20340,HUMAN|HGNC=10011|UniProtKB=Q15382,HUMAN|HGNC=1736|UniProtKB=P60953,HUMAN|HGNC=9164|UniProtKB=Q99719,HUMAN|HGNC=10534|Uni	GTPase activity

<table>
<tr><td></td><td>ProtKB=Q9NR31,HUMAN|HGNC=29125|UniProtKB=Q9UQ16,HUMAN|HGNC=9847|UniProtKB=P43487,HUMAN|HGNC=12363|UniProtKB=P49815,HUMAN|HGNC=18370|UniProtKB=Q9H0U4,HUMAN|HGNC=17073|UniProtKB=Q9UNA1</td><td></td></tr>
<tr><td>GO:0010942</td><td>HUMAN|HGNC=5141|UniProtKB=P00738,HUMAN|HGNC=5156|UniProtKB=P00739</td><td>positive regulation of cell death</td></tr>
<tr><td>GO:0031011</td><td>HUMAN|HGNC=10474|UniProtKB=Q9Y265,HUMAN|HGNC=10475|UniProtKB=Q9Y230</td><td>Ino80 complex</td></tr>
<tr><td>GO:0043066</td><td>HUMAN|HGNC=613|UniProtKB=P02649</td><td>negative regulation of apoptotic process</td></tr>
<tr><td>GO:0015871</td><td>HUMAN|HGNC=17292|UniProtKB=Q8IWA5</td><td>choline transport</td></tr>
<tr><td>GO:0071014</td><td>HUMAN|HGNC=5046|UniProtKB=P52272</td><td>post-mRNA release spliceosomal complex</td></tr>
<tr><td>PC00190</td><td>HUMAN|HGNC=12822|UniProtKB=Q9NQW7,HUMAN|HGNC=9545|UniProtKB=P28062,HUMAN|HGNC=9533|UniProtKB=P25789,HUMAN|HGNC=26212|UniProtKB=P61009,HUMAN|HGNC=9554|UniProtKB=Q99460,HUMAN|HGNC=9552|UniProtKB=P62195,HUMAN|HGNC=188|UniProtKB=O14672,HUMAN|HGNC=1507|UniProtKB=P55212,HUMAN|HGNC=9559|UniProtKB=Q13200,HUMAN|HGNC=9531|UniProtKB=P25787,HUMAN|HGNC=9535|UniProtKB=P60900,HUMAN|HGNC=9539|UniProtKB=P49721,HUMAN|HGNC=1504|UniProtKB=P42574,HUMAN|HGNC=9536|UniProtKB=O14818,HUMAN|HGNC=9540|UniProtKB=P49720,HUMAN|HGNC=9530|UniProtKB=P25786,HUMAN|HGNC=9553|UniProtKB=P62333,HUMAN|HGNC=9551|UniProtKB=P43686,HUMAN|HGNC=2312|UniProtKB=P15169,HUMAN|HGNC=2239|UniProtKB=Q9UNS2,HUMAN|HGNC=9532|UniProtKB=P25788,HUMAN|HGNC=9537|UniProtKB=P20618</td><td>protease</td></tr>
<tr><td>GO:0048812</td><td>HUMAN|HGNC=11408|UniProtKB=Q13043,HUMAN|HGNC=30765|UniProtKB=Q9UKE5</td><td>neuron projection morphogenesis</td></tr>
<tr><td>GO:0016020</td><td>HUMAN|HGNC=8876|UniProtKB=P17858,HUMAN|HGNC=1742|UniProtKB=P50851,HUMAN|HGNC=618|UniProtKB=O14791,HUMAN|HGNC=8878|UniProtKB=Q01813,HUMAN|HGNC=679|UniProtKB=P52566,HUMAN|HGNC=17102|UniProtKB=Q92619,HUMAN|HGNC=17038|UniProtKB=Q99685,HUMAN|HGNC=17292|UniProtKB=Q8IWA5,HUMAN|HGNC=28973|UniProtKB=Q14165,HUMAN|HGNC=16937|UniProtKB=O75915,HUMAN|HGNC=1090|UniProtKB=Q03001,HUMAN|HGNC=25268|UniProtKB=Q96LR9,HUMAN|HGNC=30244|UniProtKB=Q6P1A2,HUMAN|HGNC=8087|UniProtKB=P29728,HUMAN|HGNC=18808|UniProtKB=Q9Y6E2,HUMAN|HGNC=5048|UniProtKB=Q00839,HUMAN|HGNC=10542|UniProtKB=O95248,HUMAN|HGNC=678|UniProtKB=P52565</td><td>membrane</td></tr>
<tr><td>GO:0030286</td><td>HUMAN|HGNC=2951|UniProtKB=Q9C0G6</td><td>dynein complex</td></tr>
<tr><td>GO:0018107</td><td>HUMAN|HGNC=10252|UniProtKB=O75116,HUMAN|HGNC=1738|UniProtKB=Q9Y5S2</td><td>peptidyl-threonine phosphorylation</td></tr>
<tr><td>GO:0035601</td><td>HUMAN|HGNC=6737|UniProtKB=O75608</td><td>protein deacylation</td></tr>
<tr><td>GO:0030133</td><td>HUMAN|HGNC=26486|UniProtKB=Q6PJW8</td><td>transport vesicle</td></tr>
<tr><td>GO:0034704</td><td>HUMAN|HGNC=23526|UniProtKB=Q8NE86</td><td>calcium channel complex</td></tr>
<tr><td>GO:0006400</td><td>HUMAN|HGNC=20|UniProtKB=P49588</td><td>tRNA modification</td></tr>
</table>

GO:0071621	HUMAN|HGNC=9240|UniProtKB=P02775,HUMAN|HGNC=533|UniProtKB=P04083,HUMAN|HGNC=10632|UniProtKB=P13501,HUMAN|HGNC=8862|UniProtKB=P10720,HUMAN|HGNC=8861|UniProtKB=P02776	granulocyte chemotaxis
GO:0002040	HUMAN|HGNC=533|UniProtKB=P04083,HUMAN|HGNC=7178|UniProtKB=Q13201	sprouting angiogenesis
GO:0046839	HUMAN|HGNC=17059|UniProtKB=Q9NTJ5,HUMAN|HGNC=10542|UniProtKB=O95248	phospholipid dephosphorylation
GO:0030865	HUMAN|HGNC=18756|UniProtKB=Q9BYZ6,HUMAN|HGNC=9802|UniProtKB=P15153,HUMAN|HGNC=9801|UniProtKB=P63000	cortical cytoskeleton organization
GO:0031224	HUMAN|HGNC=18587|UniProtKB=O94901	intrinsic component of membrane
GO:0071456	HUMAN|HGNC=17865|UniProtKB=Q96A26	cellular response to hypoxia
GO:0035091	HUMAN|HGNC=16051|UniProtKB=Q8TEW0,HUMAN|HGNC=11172|UniProtKB=Q13596	phosphatidylinositol binding
GO:0071356	HUMAN|HGNC=10632|UniProtKB=P13501	cellular response to tumor necrosis factor
GO:0120115	HUMAN|HGNC=20471|UniProtKB=O95777,HUMAN|HGNC=17259|UniProtKB=Q9Y4Z0	Lsm2-8 complex
GO:0003729	HUMAN|HGNC=27424|UniProtKB=Q5T481,HUMAN|HGNC=8648|UniProtKB=Q15366,HUMAN|HGNC=5046|UniProtKB=P52272,HUMAN|HGNC=20165|UniProtKB=Q9P2P1,HUMAN|HGNC=117|UniProtKB=P21399,HUMAN|HGNC=9583|UniProtKB=P26599,HUMAN|HGNC=1243|UniProtKB=Q07021,HUMAN|HGNC=8647|UniProtKB=Q15365,HUMAN|HGNC=17894|UniProtKB=Q14966,HUMAN|HGNC=29513|UniProtKB=O60306,HUMAN|HGNC=5044|UniProtKB=P61978,HUMAN|HGNC=3297|UniProtKB=P78344	mRNA binding
GO:0010976	HUMAN|HGNC=9621|UniProtKB=Q6IBS0,HUMAN|HGNC=15474|UniProtKB=Q9Y566,HUMAN|HGNC=14294|UniProtKB=Q9BYB0	positive regulation of neuron projection development
GO:0036094	HUMAN|HGNC=7694|UniProtKB=O14561,HUMAN|HGNC=9922|UniProtKB=P02753,HUMAN|HGNC=4458|UniProtKB=P06744,HUMAN|HGNC=4187|UniProtKB=P02774,HUMAN|HGNC=26801|UniProtKB=Q8N8Q8,HUMAN|HGNC=11834|UniProtKB=P29401,HUMAN|HGNC=6631|UniProtKB=P49257,HUMAN|HGNC=3951|UniProtKB=Q16595,HUMAN|HGNC=9723|UniProtKB=P11216,HUMAN|HGNC=9725|UniProtKB=P06737	small molecule binding
GO:0030041	HUMAN|HGNC=12733|UniProtKB=Q9Y6W5,HUMAN|HGNC=1488|UniProtKB=P52907,HUMAN|HGNC=2696|UniProtKB=Q9UJU6,HUMAN|HGNC=4374|UniProtKB=O60234,HUMAN|HGNC=3338|UniProtKB=Q14247,HUMAN|HGNC=9581|UniProtKB=Q9H939,HUMAN|HGNC=706|UniProtKB=O15145,HUMAN|HGNC=170|UniProtKB=P61158,HUMAN|HGNC=3750|UniProtKB=Q13045,HUMAN|HGNC=15667|UniProtKB=P58546,HUMAN|HGNC=708|UniProtKB=O15511,HUMAN|HGNC=12652|UniProtKB=P50552,HUMAN|HGNC=12690|UniProtKB=P09327,HUMAN|HGNC=705|UniProtKB=O15144,HUMAN|HGNC=2695|UniProtKB=Q16643,HUMAN|HGNC=8881|UniProtKB=P07737,HUMAN|HGNC=169|UniProtKB=P61160,HUMAN|HGNC=18304|UniProtKB=Q14019,HUMAN|HGNC=9621|UniProtKB=Q6IBS0,HUMAN|HGNC=243|UniProtKB=P35611,HUMAN|HGNC=704|UniProtKB=O15143,HUMAN|HGNC=1491|UniProtKB=P47756,HUMAN|HGNC=4844|UniProtKB=P14317,HUMAN|HGNC=11881|UniProtKB=P62328,HUMAN|HGNC=21035|UniProtKB=Q8N392	actin filament polymerization

<table>
<tr><td>GO:0000387</td><td>HUMAN|HGNC=17259|UniProtKB=Q9Y4Z0,HUMAN|HGNC=30796|UniProtKB=Q9Y3F4</td><td>spliceosomal snRNP assembly</td></tr>
<tr><td>GO:0008173</td><td>HUMAN|HGNC=17136|UniProtKB=Q8IY81</td><td>RNA methyltransferase activity</td></tr>
<tr><td>GO:0042058</td><td>HUMAN|HGNC=25153|UniProtKB=Q96EY5</td><td>regulation of epidermal growth factor receptor signaling pathway</td></tr>
<tr><td>GO:0043407</td><td>HUMAN|HGNC=9673|UniProtKB=Q12913</td><td>negative regulation of MAP kinase activity</td></tr>
<tr><td>GO:0072686</td><td>HUMAN|HGNC=14906|UniProtKB=Q8N4C6</td><td>mitotic spindle</td></tr>
<tr><td>GO:0030125</td><td>HUMAN|HGNC=25951|UniProtKB=Q6ULP2,HUMAN|HGNC=555|UniProtKB=O43747,HUMAN|HGNC=23186|UniProtKB=Q14677,HUMAN|HGNC=2090|UniProtKB=P09496,HUMAN|HGNC=561|UniProtKB=O95782</td><td>clathrin vesicle coat</td></tr>
<tr><td>GO:0043005</td><td>HUMAN|HGNC=29943|UniProtKB=Q9NT68,HUMAN|HGNC=26555|UniProtKB=Q3SY00,HUMAN|HGNC=19296|UniProtKB=Q9UF33,HUMAN|HGNC=6510|UniProtKB=P16949,HUMAN|HGNC=492|UniProtKB=P16157</td><td>neuron projection</td></tr>
<tr><td>GO:0007259</td><td>HUMAN|HGNC=11364|UniProtKB=P40763</td><td>receptor signaling pathway via JAK-STAT</td></tr>
<tr><td>GO:0006956</td><td>HUMAN|HGNC=5575|UniProtKB=A0A0B4J1V2,HUMAN|HGNC=5645|UniProtKB=A0A0C4DH34,HUMAN|HGNC=5549|UniProtKB=A0A0C4DH31,HUMAN|HGNC=5554|UniProtKB=P01743,HUMAN|HGNC=5480|UniProtKB=P01880,HUMAN|HGNC=5659|UniProtKB=A0A0C4DH38,HUMAN|HGNC=5634|UniProtKB=A0A075B7F0,HUMAN|HGNC=38476|UniProtKB=B9A064,HUMAN|HGNC=1324|UniProtKB=P0C0L5,HUMAN|HGNC=5861|UniProtKB=A0M8Q6,HUMAN|HGNC=5647|UniProtKB=A0A087WSY4,HUMAN|HGNC=5563|UniProtKB=A0A075B7D0,HUMAN|HGNC=5856|UniProtKB=P0DOY2,HUMAN|HGNC=2367|UniProtKB=P02741,HUMAN|HGNC=5606|UniProtKB=P01763,HUMAN|HGNC=5576|UniProtKB=P01817,HUMAN|HGNC=5528|UniProtKB=P01861,HUMAN|HGNC=5541|UniProtKB=P01871,HUMAN|HGNC=5585|UniProtKB=A0A0C4DH32,HUMAN|HGNC=5551|UniProtKB=A0A0C4DH33,HUMAN|HGNC=5526|UniProtKB=P01859,HUMAN|HGNC=5657|UniProtKB=P0DP08,HUMAN|Ensembl=ENSG00000282639|UniProtKB=A0A0J9YX35,HUMAN|Gene=HV309_HUMAN|UniProtKB=P01782,HUMAN|HGNC=5654|UniProtKB=P01825,HUMAN|HGNC=5553|UniProtKB=A0A0A0MS14,HUMAN|Gene=HV108_HUMAN|UniProtKB=P0DP01,HUMAN|HGNC=5624|UniProtKB=A0A0B4J1X5,HUMAN|HGNC=5620|UniProtKB=P01780,HUMAN|HGNC=5598|UniProtKB=A0A0C4DH35,HUMAN|HGNC=5580|UniProtKB=P01762,HUMAN|HGNC=5633|UniProtKB=A0A075B7D8,HUMAN|HGNC=5582|UniProtKB=A0A0B4J1V0,HUMAN|HGNC=5478|UniProtKB=P01876,HUMAN|HGNC=5617|UniProtKB=A0A075B6Q5,HUMAN|HGNC=5594|UniProtKB=P0DP03,HUMAN|HGNC=5655|UniProtKB=A0A0C4DH41,HUMAN|HGNC=5636|UniProtKB=A0A075B7B8,HUMAN|HGNC=5586|UniProtKB=A0A0B4J1V1,HUMAN|HGNC=5581|UniProtKB=P01766,HUMAN|HGNC=5622|UniProtKB=A0A0B4J1Y9,HUMAN|HGNC=5652|UniProtKB=A0A075B6R2,HUMAN|HGNC=5593|UniProtKB=P0DP02,HUMAN|HGNC=5479|UniProtKB=P01877,HUMAN|HGNC=5619|UniProtKB=A0A0C4DH42,HUMAN|HGNC=5596|UniProtKB=P01772,HUMAN|HGNC=5550|UniProtKB=P23083,HUMAN|HGNC=5604|UniProtKB=A0A0B4J1X8,HU</td><td>complement activation</td></tr>
</table>

<table>
<tr><td></td><td>MAN|HGNC=5525|UniProtKB=P01857,HUMAN|HGNC=5662|UniProtKB=A0A0B4J1U7,HUMAN|HGNC=5601|UniProtKB=A0A0C4DH36,HUMAN|HGNC=5716|UniProtKB=P01834,HUMAN|HGNC=5588|UniProtKB=P01764,HUMAN|HGNC=5857|UniProtKB=P0DOY3,HUMAN|HGNC=5610|UniProtKB=P01767,HUMAN|Ensembl=ENSG00000282150|UniProtKB=A0A0J9YY99,HUMAN|HGNC=5527|UniProtKB=P01860,HUMAN|HGNC=584|UniProtKB=P02743</td><td></td></tr>
<tr><td>GO:0022008</td><td>HUMAN|HGNC=13206|UniProtKB=O60296</td><td>neurogenesis</td></tr>
<tr><td>GO:0001726</td><td>HUMAN|HGNC=11148|UniProtKB=Q16658</td><td>ruffle</td></tr>
<tr><td>GO:0008198</td><td>HUMAN|HGNC=3951|UniProtKB=Q16595</td><td>ferrous iron binding</td></tr>
<tr><td>GO:0008584</td><td>HUMAN|HGNC=199|UniProtKB=O43506</td><td>male gonad development</td></tr>
<tr><td>GO:2000026</td><td>HUMAN|HGNC=3153|UniProtKB=Q16610</td><td>regulation of multicellular organismal development</td></tr>
<tr><td>GO:0019903</td><td>HUMAN|HGNC=15720|UniProtKB=Q13033,HUMAN|HGNC=11424|UniProtKB=O43815,HUMAN|HGNC=1173|UniProtKB=Q5H9R7,HUMAN|HGNC=5461|UniProtKB=P78318,HUMAN|HGNC=9302|UniProtKB=P30153,HUMAN|HGNC=6207|UniProtKB=P14923,HUMAN|HGNC=14871|UniProtKB=Q96A00,HUMAN|HGNC=9308|UniProtKB=Q15257,HUMAN|HGNC=14952|UniProtKB=Q8TAE6,HUMAN|HGNC=9304|UniProtKB=P63151</td><td>protein phosphatase binding</td></tr>
<tr><td>GO:0007160</td><td>HUMAN|HGNC=23151|UniProtKB=Q86UX7,HUMAN|HGNC=3662|UniProtKB=P02675,HUMAN|HGNC=6156|UniProtKB=P05106,HUMAN|HGNC=6153|UniProtKB=P05556,HUMAN|HGNC=6040|UniProtKB=Q13418</td><td>cell-matrix adhesion</td></tr>
<tr><td>GO:0010821</td><td>HUMAN|HGNC=2973|UniProtKB=O00429,HUMAN|HGNC=17865|UniProtKB=Q96A26,HUMAN|HGNC=1050|UniProtKB=P55957</td><td>regulation of mitochondrion organization</td></tr>
<tr><td>GO:0004322</td><td>HUMAN|HGNC=3951|UniProtKB=Q16595,HUMAN|HGNC=2295|UniProtKB=P00450</td><td>ferroxidase activity</td></tr>
<tr><td>PC00042</td><td>HUMAN|HGNC=6522|UniProtKB=P04180,HUMAN|HGNC=2328|UniProtKB=P50416,HUMAN|HGNC=94|UniProtKB=Q9BWD1</td><td>acyltransferase</td></tr>
<tr><td>GO:0034453</td><td>HUMAN|HGNC=14906|UniProtKB=Q8N4C6</td><td>microtubule anchoring</td></tr>
<tr><td>GO:0051018</td><td>HUMAN|HGNC=369|UniProtKB=Q9UKA4</td><td>protein kinase A binding</td></tr>
<tr><td>GO:0042098</td><td>HUMAN|HGNC=11491|UniProtKB=P43405,HUMAN|HGNC=533|UniProtKB=P04083</td><td>T cell proliferation</td></tr>
<tr><td>GO:0015267</td><td>HUMAN|HGNC=4234|UniProtKB=P55040</td><td>channel activity</td></tr>
<tr><td>GO:0035335</td><td>HUMAN|HGNC=9645|UniProtKB=Q05209,HUMAN|HGNC=29884|UniProtKB=Q8TF42</td><td>peptidyl-tyrosine dephosphorylation</td></tr>
<tr><td>GO:1902476</td><td>HUMAN|HGNC=25240|UniProtKB=Q4KMQ2</td><td>chloride transmembrane transport</td></tr>
<tr><td>GO:0005681</td><td>HUMAN|HGNC=1743|UniProtKB=Q99459,HUMAN|HGNC=24932|UniProtKB=Q9NZ63</td><td>spliceosomal complex</td></tr>
<tr><td>PC00224</td><td>HUMAN|HGNC=3300|UniProtKB=P63241,HUMAN|HGNC=3250|UniProtKB=P47813,HUMAN|HGNC=37237|UniProtKB=A6NDY0,HUMAN|HGNC=3252|UniProtKB=O14602,HUMAN|HGNC=3265|UniProtKB=P05198,HUMAN|HGNC=9565|UniProtKB=P51665,HUMAN|HGNC=30796|UniProtKB=Q9Y3F4,HUMAN|Gene=EIF2S3L|UniProt</td><td>translation initiation factor</td></tr>
</table>

<table>
<tr><td></td><td>KB=Q2VIR3,HUMAN|HGNC=3285|UniProtKB=P23588,HUMAN|HGNC=3297|UniProtKB=P78344</td><td></td></tr>
<tr><td>PC00156</td><td>HUMAN|HGNC=2949|UniProtKB=Q8TD57,HUMAN|HGNC=6325|UniProtKB=O60282,HUMAN|HGNC=2951|UniProtKB=Q9C0G6,HUMAN|HGNC=2713|UniProtKB=O75935,HUMAN|HGNC=2952|UniProtKB=Q96JB1,HUMAN|HGNC=2712|UniProtKB=Q13561,HUMAN|HGNC=6317|UniProtKB=O43896,HUMAN|HGNC=6387|UniProtKB=Q07866,HUMAN|HGNC=17060|UniProtKB=Q92845</td><td>microtubule binding motor protein</td></tr>
<tr><td>GO:0051156</td><td>HUMAN|HGNC=4458|UniProtKB=P06744,HUMAN|HGNC=4922|UniProtKB=P19367,HUMAN|HGNC=11834|UniProtKB=P29401,HUMAN|HGNC=8891|UniProtKB=P52209,HUMAN|HGNC=8903|UniProtKB=O95336</td><td>glucose 6-phosphate metabolic process</td></tr>
<tr><td>GO:0051607</td><td>HUMAN|HGNC=8087|UniProtKB=P29728</td><td>defense response to virus</td></tr>
<tr><td>GO:0006622</td><td>HUMAN|HGNC=6501|UniProtKB=P13473</td><td>protein targeting to lysosome</td></tr>
<tr><td>GO:1905368</td><td>HUMAN|HGNC=26212|UniProtKB=P61009</td><td>peptidase complex</td></tr>
<tr><td>GO:0051966</td><td>HUMAN|HGNC=1406|UniProtKB=Q9Y698,HUMAN|HGNC=15474|UniProtKB=Q9Y566,HUMAN|HGNC=14294|UniProtKB=Q9BYB0</td><td>regulation of synaptic transmission, glutamatergic</td></tr>
<tr><td>GO:0001889</td><td>HUMAN|HGNC=533|UniProtKB=P04083</td><td>liver development</td></tr>
<tr><td>GO:0005743</td><td>HUMAN|HGNC=5261|UniProtKB=P10809,HUMAN|HGNC=7863|UniProtKB=Q13423</td><td>mitochondrial inner membrane</td></tr>
<tr><td>PC00104</td><td>HUMAN|HGNC=4298|UniProtKB=P16278</td><td>galactosidase</td></tr>
<tr><td>PC00011</td><td>HUMAN|HGNC=20473|UniProtKB=Q9BX63</td><td>DNA helicase</td></tr>
<tr><td>GO:0031532</td><td>HUMAN|HGNC=9070|UniProtKB=P08567,HUMAN|HGNC=533|UniProtKB=P04083,HUMAN|HGNC=14653|UniProtKB=Q9HBI1,HUMAN|HGNC=15878|UniProtKB=Q9NQ75</td><td>actin cytoskeleton reorganization</td></tr>
<tr><td>GO:0019887</td><td>HUMAN|HGNC=9391|UniProtKB=P13861,HUMAN|HGNC=9392|UniProtKB=P31323,HUMAN|HGNC=9386|UniProtKB=Q9UGJ0</td><td>protein kinase regulator activity</td></tr>
<tr><td>GO:0032446</td><td>HUMAN|HGNC=21314|UniProtKB=Q8WXC6,HUMAN|HGNC=20597|UniProtKB=P61960</td><td>protein modification by small protein conjugation</td></tr>
<tr><td>GO:0006260</td><td>HUMAN|HGNC=26200|UniProtKB=Q9H668</td><td>DNA replication</td></tr>
<tr><td>GO:0051123</td><td>HUMAN|HGNC=9553|UniProtKB=P62333,HUMAN|HGNC=9552|UniProtKB=P62195,HUMAN|HGNC=9551|UniProtKB=P43686</td><td>RNA polymerase II preinitiation complex assembly</td></tr>
<tr><td>GO:0030136</td><td>HUMAN|HGNC=2092|UniProtKB=Q00610,HUMAN|HGNC=13667|UniProtKB=Q9BXS5</td><td>clathrin-coated vesicle</td></tr>
<tr><td>PC00060</td><td>HUMAN|HGNC=535|UniProtKB=P50995,HUMAN|HGNC=537|UniProtKB=P07355,HUMAN|HGNC=545|UniProtKB=P20073,HUMAN|HGNC=544|UniProtKB=P08133,HUMAN|HGNC=533|UniProtKB=P04083,HUMAN|HGNC=543|UniProtKB=P08758,HUMAN|HGNC=9449|UniProtKB=P04156</td><td>calcium-binding protein</td></tr>
<tr><td>GO:0045893</td><td>HUMAN|HGNC=25758|UniProtKB=Q8TED0,HUMAN|HGNC=12738|UniProtKB=Q969T9</td><td>positive regulation of transcription, DNA-templated</td></tr>
<tr><td>GO:0030424</td><td>HUMAN|HGNC=17780|UniProtKB=Q562R1,HUMAN|HGNC=1117|UniProtKB=Q9UPA5,HUMAN|HGNC=2974|UniProtKB=P50570,HUMAN|HGNC=6317|UniProtKB=O43896,HUMAN|HGNC=11510|UniProtKB=Q8N9I0,HUMAN|HGNC=29125|UniProtKB=Q9UQ16,HUMAN|HGNC=29557|UniProtKB=Q0ZGT2,HUMAN|HGNC=10594|Uni</td><td>axon</td></tr>
</table>

	ProtKB=Q01118,HUMAN|HGNC=13406|UniProtKB=Q9Y6V0,HUMAN|HGNC=10582|UniProtKB=Q9Y5Y9,HUMAN|HGNC=11512|UniProtKB=Q9H2B2,HUMAN|HGNC=6091|UniProtKB=P06213	
GO:0048255	HUMAN|HGNC=5048|UniProtKB=Q00839	mRNA stabilization
GO:0002456	HUMAN|HGNC=4932|UniProtKB=P01889	T cell mediated immunity
GO:0004550	HUMAN|HGNC=363|UniProtKB=P27144,HUMAN|HGNC=7850|UniProtKB=P22392,HUMAN|HGNC=7849|UniProtKB=P15531	nucleoside diphosphate kinase activity
GO:0005881	HUMAN|HGNC=6891|UniProtKB=Q15555,HUMAN|HGNC=6890|UniProtKB=Q15691	cytoplasmic microtubule
GO:0050807	HUMAN|HGNC=3338|UniProtKB=Q14247,HUMAN|HGNC=2695|UniProtKB=Q16643,HUMAN|HGNC=2696|UniProtKB=Q9UJU6,HUMAN|HGNC=4844|UniProtKB=P14317	regulation of synapse organization
GO:0007229	HUMAN|HGNC=6616|UniProtKB=P48059,HUMAN|HGNC=6156|UniProtKB=P05106,HUMAN|HGNC=4036|UniProtKB=O15117,HUMAN|HGNC=13937|UniProtKB=O95866,HUMAN|HGNC=13200|UniProtKB=Q15942,HUMAN|HGNC=6153|UniProtKB=P05556,HUMAN|HGNC=6040|UniProtKB=Q13418	integrin-mediated signaling pathway
GO:0006739	HUMAN|HGNC=7863|UniProtKB=Q13423,HUMAN|HGNC=5382|UniProtKB=O75874,HUMAN|HGNC=11834|UniProtKB=P29401,HUMAN|HGNC=5383|UniProtKB=P48735,HUMAN|HGNC=8891|UniProtKB=P52209,HUMAN|HGNC=8903|UniProtKB=O95336	NADP metabolic process
GO:0016853	HUMAN|HGNC=4116|UniProtKB=Q14376,HUMAN|HGNC=4458|UniProtKB=P06744,HUMAN|HGNC=30167|UniProtKB=P13667,HUMAN|HGNC=9959|UniProtKB=P51606,HUMAN|HGNC=12009|UniProtKB=P60174,HUMAN|HGNC=8548|UniProtKB=P07237,HUMAN|HGNC=9308|UniProtKB=Q15257,HUMAN|HGNC=9261|UniProtKB=Q13356,HUMAN|HGNC=9756|UniProtKB=O00391,HUMAN|HGNC=8988|UniProtKB=Q13526,HUMAN|HGNC=4606|UniProtKB=P30101,HUMAN|HGNC=18144|UniProtKB=Q8IZU8	isomerase activity
GO:0008253	HUMAN|HGNC=17820|UniProtKB=Q9H0P0	5'-nucleotidase activity
GO:0001816	HUMAN|HGNC=5261|UniProtKB=P10809,HUMAN|HGNC=7183|UniProtKB=P41218,HUMAN|HGNC=533|UniProtKB=P04083,HUMAN|HGNC=1628|UniProtKB=P08571,HUMAN|HGNC=16961|UniProtKB=Q15762	cytokine production
GO:0031936	HUMAN|HGNC=4716|UniProtKB=P16403,HUMAN|HGNC=4718|UniProtKB=P10412,HUMAN|HGNC=4719|UniProtKB=P16401	negative regulation of chromatin silencing
GO:0045892	HUMAN|HGNC=30747|UniProtKB=P61201,HUMAN|HGNC=9349|UniProtKB=Q9NQX1	negative regulation of transcription, DNA-templated
GO:0004622	HUMAN|HGNC=16268|UniProtKB=Q8IY17	lysophospholipase activity
GO:0007098	HUMAN|HGNC=23432|UniProtKB=Q9NXG0,HUMAN|HGNC=29327|UniProtKB=Q5VZ46,HUMAN|HGNC=9847|UniProtKB=P43487	centrosome cycle
GO:0009306	HUMAN|HGNC=31332|UniProtKB=Q6IQ22,HUMAN|HGNC=7007|UniProtKB=P61006,HUMAN|HGNC=9778|UniProtKB=P20337,HUMAN|HGNC=1628|UniProtKB=P08571	protein secretion
GO:0043410	HUMAN|HGNC=26068|UniProtKB=Q6IAA8,HUMAN|HGNC=6091|UniProtKB=P06213	positive regulation of MAPK cascade
GO:0098661	HUMAN|HGNC=10989|UniProtKB=Q00325	inorganic anion transmembrane transport

<table>
<tr><td>GO:0005886</td><td>HUMAN|HGNC=25951|UniProtKB=Q6ULP2,HUMAN|Gene=SEP15|UniProtKB=O60613,HUMAN|HGNC=9862|UniProtKB=P61225,HUMAN|HGNC=3383|UniProtKB=P27105,HUMAN|HGNC=9784|UniProtKB=P61020,HUMAN|HGNC=11834|UniProtKB=P29401,HUMAN|HGNC=555|UniProtKB=O43747,HUMAN|HGNC=6407|UniProtKB=P01116,HUMAN|HGNC=813|UniProtKB=Q93084,HUMAN|HGNC=13487|UniProtKB=Q96QK1,HUMAN|HGNC=463|UniProtKB=Q9UKV5,HUMAN|HGNC=11445|UniProtKB=Q15833,HUMAN|HGNC=851|UniProtKB=P38606,HUMAN|HGNC=9756|UniProtKB=O00391,HUMAN|HGNC=15453|UniProtKB=Q9H3P7,HUMAN|HGNC=9761|UniProtKB=Q15907,HUMAN|HGNC=24636|UniProtKB=Q9UHB6,HUMAN|HGNC=22222|UniProtKB=Q9C0H2,HUMAN|HGNC=453|UniProtKB=P02760,HUMAN|HGNC=20589|UniProtKB=Q9P0J7,HUMAN|HGNC=10996|UniProtKB=O14975,HUMAN|HGNC=9785|UniProtKB=P51148,HUMAN|HGNC=2230|UniProtKB=P53621,HUMAN|HGNC=2431|UniProtKB=P55060,HUMAN|HGNC=16268|UniProtKB=Q8IY17,HUMAN|HGNC=11937|UniProtKB=P32970,HUMAN|HGNC=9772|UniProtKB=Q13637,HUMAN|HGNC=3380|UniProtKB=Q9Y2J2,HUMAN|HGNC=12644|UniProtKB=Q15836,HUMAN|HGNC=24251|UniProtKB=Q9Y3A6,HUMAN|HGNC=8569|UniProtKB=P29122,HUMAN|HGNC=4606|UniProtKB=P30101,HUMAN|HGNC=25153|UniProtKB=Q96EY5,HUMAN|HGNC=9782|UniProtKB=P61018,HUMAN|HGNC=5253|UniProtKB=P07900,HUMAN|HGNC=11446|UniProtKB=O00186,HUMAN|HGNC=11172|UniProtKB=Q13596,HUMAN|HGNC=16499|UniProtKB=Q96DA2,HUMAN|HGNC=16075|UniProtKB=Q9H082,HUMAN|HGNC=11442|UniProtKB=O15400,HUMAN|HGNC=5258|UniProtKB=P08238,HUMAN|HGNC=9852|UniProtKB=O95373,HUMAN|HGNC=16090|UniProtKB=O00264,HUMAN|HGNC=11740|UniProtKB=P02787,HUMAN|HGNC=2295|UniProtKB=P00450,HUMAN|HGNC=16996|UniProtKB=Q15363,HUMAN|HGNC=30384|UniProtKB=Q9UMX5,HUMAN|HGNC=14902|UniProtKB=Q9NRW1,HUMAN|HGNC=26212|UniProtKB=P61009,HUMAN|HGNC=9935|UniProtKB=Q14257,HUMAN|HGNC=11984|UniProtKB=Q6ZVM7,HUMAN|HGNC=15999|UniProtKB=Q9NZV5,HUMAN|HGNC=23186|UniProtKB=Q14677,HUMAN|HGNC=8016|UniProtKB=P46459,HUMAN|HGNC=9788|UniProtKB=P51149,HUMAN|HGNC=26068|UniProtKB=Q6IAA8,HUMAN|HGNC=33704|UniProtKB=Q66K66,HUMAN|HGNC=16524|UniProtKB=P61106,HUMAN|HGNC=20726|UniProtKB=Q8WVM8,HUMAN|HGNC=3382|UniProtKB=Q08495,HUMAN|HGNC=1458|UniProtKB=O43852,HUMAN|HGNC=13529|UniProtKB=Q9Y2B0,HUMAN|HGNC=2532|UniProtKB=P08311,HUMAN|HGNC=5238|UniProtKB=P11021,HUMAN|HGNC=23787|UniProtKB=Q9P1W3,HUMAN|HGNC=16998|UniProtKB=P49755,HUMAN|HGNC=11357|UniProtKB=Q92783,HUMAN|HGNC=13531|UniProtKB=Q9Y2Q0,HUMAN|HGNC=16695|UniProtKB=P51572,HUMAN|HGNC=30167|UniProtKB=P13667,HUMAN|HGNC=8761|UniProtKB=Q9BUL8,HUMAN|HGNC=2479|UniProtKB=O76096,HUMAN|HGNC=20954|UniProtKB=Q9NP79,HUMAN|HGNC=15461|UniProtKB=P55145,HUMAN|HGNC=21354|UniProtKB=Q86XR7,HUMAN|HGNC=9857|UniProtKB=P61224,HUMAN|HGNC=1546|UniProtKB=P50454,HUMAN|HGNC=15447|UniProtKB=Q9Y4G6,HUMAN|HGNC=9086|UniProtKB=P60201,HUMAN|HGNC=30220|UniProtKB=Q96AA3,HUMAN|HGNC=15578|UniProtKB=Q96D71,HUMAN|HGNC=3530|UniProtKB=P00748,HUMAN|HGNC=9786|UniProtKB=P20340,HUMAN|HGNC</td><td>plasma membrane</td></tr>
</table>

=17103|UniProtKB=Q9Y5L0,HUMAN|HGNC=9164|UniProtKB=Q99
719,HUMAN|HGNC=9070|UniProtKB=P08567,HUMAN|HGNC=55|
UniProtKB=O15439,HUMAN|HGNC=10534|UniProtKB=Q9NR31,H
UMAN|HGNC=15588|UniProtKB=Q96C24,HUMAN|HGNC=6631|U
niProtKB=P49257,HUMAN|HGNC=649|UniProtKB=P48444,HUMAN
|HGNC=18370|UniProtKB=Q9H0U4,HUMAN|HGNC=24641|UniPro
tKB=Q7Z3J2,HUMAN|HGNC=19983|UniProtKB=Q86YS6,HUMAN|H
GNC=12666|UniProtKB=P55072,HUMAN|HGNC=11133|UniProtKB
=O95721,HUMAN|HGNC=10382|UniProtKB=P04844,HUMAN|HGN
C=11131|UniProtKB=O00161,HUMAN|HGNC=25240|UniProtKB=Q
4KMQ2,HUMAN|HGNC=9411|UniProtKB=P14314,HUMAN|HGNC=
11034|UniProtKB=Q2Y0W8,HUMAN|HGNC=17943|UniProtKB=Q9
GZP9,HUMAN|HGNC=6752|UniProtKB=P20645,HUMAN|HGNC=15
606|UniProtKB=Q9UHA4,HUMAN|HGNC=11767|UniProtKB=O432
94,HUMAN|HGNC=24175|UniProtKB=Q9P035,HUMAN|HGNC=160
89|UniProtKB=O15173,HUMAN|HGNC=9855|UniProtKB=P62834,H
UMAN|HGNC=9581|UniProtKB=Q9H939,HUMAN|HGNC=8533|Un
iProtKB=P51575,HUMAN|HGNC=17059|UniProtKB=Q9NTJ5,HUMA
N|HGNC=11358|UniProtKB=O75886,HUMAN|HGNC=17859|UniPr
otKB=Q5SRE5,HUMAN|HGNC=11828|UniProtKB=Q9UDY2,HUMAN
|HGNC=25541|UniProtKB=P57088,HUMAN|HGNC=9840|UniProtK
B=P11234,HUMAN|HGNC=2090|UniProtKB=P09496,HUMAN|HGN
C=15687|UniProtKB=O75563,HUMAN|HGNC=11056|UniProtKB=P
48067,HUMAN|HGNC=7821|UniProtKB=P14543,HUMAN|HGNC=3
0052|UniProtKB=Q8TEM1,HUMAN|HGNC=9778|UniProtKB=P2033
7,HUMAN|HGNC=8548|UniProtKB=P07237,HUMAN|HGNC=533|U
niProtKB=P04083,HUMAN|HGNC=2047|UniProtKB=O00501,HUMA
N|HGNC=568|UniProtKB=O14617,HUMAN|HGNC=10447|UniProt
KB=P10301,HUMAN|HGNC=18673|UniProtKB=Q96JB5,HUMAN|H
GNC=1473|UniProtKB=P27824,HUMAN|HGNC=8504|UniProtKB=Q
969R2,HUMAN|HGNC=5241|UniProtKB=P11142,HUMAN|HGNC=9
802|UniProtKB=P15153,HUMAN|HGNC=10608|UniProtKB=P47872
,HUMAN|HGNC=13937|UniProtKB=O95866,HUMAN|HGNC=3244|
UniProtKB=Q9NZN3,HUMAN|HGNC=7228|UniProtKB=P22897,HU
MAN|HGNC=13921|UniProtKB=O95870,HUMAN|HGNC=12649|U
niProtKB=O95292,HUMAN|HGNC=15878|UniProtKB=Q9NQ75,HU
MAN|HGNC=20653|UniProtKB=Q8IVB4,HUMAN|HGNC=4234|Uni
ProtKB=P55040,HUMAN|HGNC=26486|UniProtKB=Q6PJW8,HUMA
N|HGNC=4045|UniProtKB=O75084,HUMAN|HGNC=18756|UniPro
tKB=Q9BYZ6,HUMAN|HGNC=652|UniProtKB=P84077,HUMAN|HG
NC=2315|UniProtKB=Q96FN4,HUMAN|HGNC=30697|UniProtKB=
Q8TB96,HUMAN|HGNC=30550|UniProtKB=O60337,HUMAN|HGN
C=3377|UniProtKB=P11171,HUMAN|HGNC=2689|UniProtKB=P09
172,HUMAN|HGNC=10701|UniProtKB=Q15436,HUMAN|HGNC=64
99|UniProtKB=P11279,HUMAN|HGNC=11326|UniProtKB=P51571,
HUMAN|HGNC=8062|UniProtKB=P49790,HUMAN|HGNC=9758|U
niProtKB=P62820,HUMAN|HGNC=12647|UniProtKB=Q9BV40,HUM
AN|HGNC=2232|UniProtKB=P35606,HUMAN|HGNC=885|UniProt
KB=O75882,HUMAN|HGNC=18263|UniProtKB=Q9UL25,HUMAN|H
GNC=812|UniProtKB=P16615,HUMAN|HGNC=692|UniProtKB=P40
616,HUMAN|HGNC=31332|UniProtKB=Q6IQ22,HUMAN|HGNC=18
587|UniProtKB=O94901,HUMAN|HGNC=18621|UniProtKB=Q9Y2V
7,HUMAN|HGNC=1455|UniProtKB=P27797,HUMAN|HGNC=2231|
UniProtKB=P53618,HUMAN|HGNC=11845|UniProtKB=Q9Y490,HU

<table>
<tr><td></td><td>MAN|HGNC=913|UniProtKB=P20160,HUMAN|HGNC=2728|UniProtKB=P39656,HUMAN|HGNC=3157|UniProtKB=Q92838,HUMAN|HGNC=17643|UniProtKB=Q9HD26,HUMAN|HGNC=20175|UniProtKB=Q9Y3E0,HUMAN|HGNC=12648|UniProtKB=Q9P0L0,HUMAN|HGNC=18304|UniProtKB=Q14019,HUMAN|HGNC=2850|UniProtKB=Q9Y6T7,HUMAN|HGNC=1801|UniProtKB=O95674,HUMAN|HGNC=10381|UniProtKB=P04843,HUMAN|HGNC=17955|UniProtKB=O43504,HUMAN|HGNC=4036|UniProtKB=O15117,HUMAN|HGNC=6501|UniProtKB=P13473,HUMAN|HGNC=10011|UniProtKB=Q15382,HUMAN|HGNC=11429|UniProtKB=O75558,HUMAN|HGNC=9847|UniProtKB=P43487,HUMAN|HGNC=12203|UniProtKB=A0A075B6N3,HUMAN|HGNC=10840|UniProtKB=P29353,HUMAN|HGNC=15663|UniProtKB=Q9NYU2,HUMAN|HGNC=16768|UniProtKB=O60391,HUMAN|HGNC=24878|UniProtKB=Q9BVK6,HUMAN|HGNC=9767|UniProtKB=O00194</td><td></td></tr>
<tr><td>GO:0008092</td><td>HUMAN|HGNC=9069|UniProtKB=Q15149,HUMAN|HGNC=492|UniProtKB=P16157</td><td>cytoskeletal protein binding</td></tr>
<tr><td>GO:0006626</td><td>HUMAN|HGNC=5261|UniProtKB=P10809</td><td>protein targeting to mitochondrion</td></tr>
<tr><td>GO:0031253</td><td>HUMAN|HGNC=11148|UniProtKB=Q16658,HUMAN|HGNC=4381|UniProtKB=Q14344</td><td>cell projection membrane</td></tr>
<tr><td>GO:0030261</td><td>HUMAN|HGNC=4716|UniProtKB=P16403,HUMAN|HGNC=4718|UniProtKB=P10412,HUMAN|HGNC=4719|UniProtKB=P16401</td><td>chromosome condensation</td></tr>
<tr><td>GO:0006897</td><td>HUMAN|HGNC=5575|UniProtKB=A0A0B4J1V2,HUMAN|HGNC=9784|UniProtKB=P61020,HUMAN|HGNC=5645|UniProtKB=A0A0C4DH34,HUMAN|HGNC=5549|UniProtKB=A0A0C4DH31,HUMAN|HGNC=5554|UniProtKB=P01743,HUMAN|HGNC=5480|UniProtKB=P01880,HUMAN|HGNC=5659|UniProtKB=A0A0C4DH38,HUMAN|HGNC=5634|UniProtKB=A0A075B7F0,HUMAN|HGNC=38476|UniProtKB=B9A064,HUMAN|HGNC=5861|UniProtKB=A0M8Q6,HUMAN|HGNC=5647|UniProtKB=A0A087WSY4,HUMAN|HGNC=5563|UniProtKB=A0A075B7D0,HUMAN|HGNC=5856|UniProtKB=P0DOY2,HUMAN|HGNC=5606|UniProtKB=P01763,HUMAN|HGNC=9785|UniProtKB=P51148,HUMAN|HGNC=9801|UniProtKB=P63000,HUMAN|HGNC=5576|UniProtKB=P01817,HUMAN|HGNC=5528|UniProtKB=P01861,HUMAN|HGNC=5541|UniProtKB=P01871,HUMAN|HGNC=5585|UniProtKB=A0A0C4DH32,HUMAN|HGNC=9782|UniProtKB=P61018,HUMAN|HGNC=9802|UniProtKB=P15153,HUMAN|HGNC=5551|UniProtKB=A0A0C4DH33,HUMAN|HGNC=3244|UniProtKB=Q9NZN3,HUMAN|HGNC=5526|UniProtKB=P01859,HUMAN|HGNC=5657|UniProtKB=P0DP08,HUMAN|Ensembl=ENSG00000282639|UniProtKB=A0A0J9YX35,HUMAN|HGNC=19760|UniProtKB=Q96T51,HUMAN|Gene=HV309_HUMAN|UniProtKB=P01782,HUMAN|HGNC=18756|UniProtKB=Q9BYZ6,HUMAN|HGNC=5654|UniProtKB=P01825,HUMAN|HGNC=5553|UniProtKB=A0A0A0MS14,HUMAN|Gene=HV108_HUMAN|UniProtKB=P0DP01,HUMAN|HGNC=5624|UniProtKB=A0A0B4J1X5,HUMAN|HGNC=5620|UniProtKB=P01780,HUMAN|HGNC=23186|UniProtKB=Q14677,HUMAN|HGNC=5598|UniProtKB=A0A0C4DH35,HUMAN|HGNC=5580|UniProtKB=P01762,HUMAN|HGNC=5633|UniProtKB=A0A075B7D8,HUMAN|HGNC=5582|UniProtKB=A0A0B4J1V0,HUMAN|HGNC=5478|UniProtKB=P01876,HUMAN|HGNC=26068|UniProtKB=Q6IAA8,HUMAN|HGNC=5617|UniProtKB=A0A075B6Q5,HUMAN|HGNC=5594|UniProtKB=P0DP03,HUMAN|HGNC=5655|UniProtKB=A0A0C4DH41,HUMAN|HGNC=563</td><td>endocytosis</td></tr>
</table>

<table>
<tr><td></td><td>6|UniProtKB=A0A075B7B8,HUMAN|HGNC=5586|UniProtKB=A0A0B4J1V1,HUMAN|HGNC=5581|UniProtKB=P01766,HUMAN|HGNC=5622|UniProtKB=A0A0B4J1Y9,HUMAN|HGNC=5652|UniProtKB=A0A075B6R2,HUMAN|HGNC=5593|UniProtKB=P0DP02,HUMAN|HGNC=3419|UniProtKB=P42566,HUMAN|HGNC=5479|UniProtKB=P01877,HUMAN|HGNC=5619|UniProtKB=A0A0C4DH42,HUMAN|HGNC=5596|UniProtKB=P01772,HUMAN|HGNC=5550|UniProtKB=P23083,HUMAN|HGNC=5604|UniProtKB=A0A0B4J1X8,HUMAN|HGNC=570|UniProtKB=P53677,HUMAN|HGNC=5525|UniProtKB=P01857,HUMAN|HGNC=5662|UniProtKB=A0A0B4J1U7,HUMAN|HGNC=24634|UniProtKB=Q9UBC2,HUMAN|HGNC=5601|UniProtKB=A0A0C4DH36,HUMAN|HGNC=5716|UniProtKB=P01834,HUMAN|HGNC=5588|UniProtKB=P01764,HUMAN|HGNC=15578|UniProtKB=Q96D71,HUMAN|HGNC=5857|UniProtKB=P0DOY3,HUMAN|HGNC=1736|UniProtKB=P60953,HUMAN|HGNC=5610|UniProtKB=P01767,HUMAN|Ensembl=ENSG00000282150|UniProtKB=A0A0J9YY99,HUMAN|HGNC=5527|UniProtKB=P01860</td><td></td></tr>
<tr><td>GO:0051015</td><td>HUMAN|HGNC=1488|UniProtKB=P52907,HUMAN|HGNC=2696|UniProtKB=Q9UJU6,HUMAN|HGNC=6513|UniProtKB=Q14847,HUMAN|HGNC=17905|UniProtKB=Q9Y613,HUMAN|HGNC=2252|UniProtKB=P31146,HUMAN|HGNC=15750|UniProtKB=P60981,HUMAN|HGNC=3338|UniProtKB=Q14247,HUMAN|HGNC=9581|UniProtKB=Q9H939,HUMAN|HGNC=706|UniProtKB=O15145,HUMAN|HGNC=12012|UniProtKB=P06753,HUMAN|HGNC=3382|UniProtKB=Q08495,HUMAN|HGNC=544|UniProtKB=P08133,HUMAN|HGNC=170|UniProtKB=P61158,HUMAN|HGNC=3750|UniProtKB=Q13045,HUMAN|HGNC=24636|UniProtKB=Q9UHB6,HUMAN|HGNC=2254|UniProtKB=Q9ULV4,HUMAN|HGNC=2253|UniProtKB=Q9BR76,HUMAN|HGNC=708|UniProtKB=O15511,HUMAN|HGNC=6528|UniProtKB=P13796,HUMAN|HGNC=27937|UniProtKB=Q6ZVH7,HUMAN|HGNC=6111|UniProtKB=Q13576,HUMAN|HGNC=12690|UniProtKB=P09327,HUMAN|HGNC=11148|UniProtKB=Q16658,HUMAN|HGNC=705|UniProtKB=O15144,HUMAN|HGNC=2695|UniProtKB=Q16643,HUMAN|HGNC=12754|UniProtKB=O75083,HUMAN|HGNC=1874|UniProtKB=P23528,HUMAN|HGNC=169|UniProtKB=P61160,HUMAN|HGNC=18304|UniProtKB=Q14019,HUMAN|HGNC=26161|UniProtKB=P57737,HUMAN|HGNC=12010|UniProtKB=P09493,HUMAN|HGNC=9621|UniProtKB=Q6IBS0,HUMAN|HGNC=243|UniProtKB=P35611,HUMAN|HGNC=704|UniProtKB=O15143,HUMAN|HGNC=12013|UniProtKB=P67936,HUMAN|HGNC=12011|UniProtKB=P07951,HUMAN|HGNC=1491|UniProtKB=P47756,HUMAN|HGNC=4844|UniProtKB=P14317</td><td>actin filament binding</td></tr>
<tr><td>GO:0005546</td><td>HUMAN|HGNC=12690|UniProtKB=P09327,HUMAN|HGNC=3750|UniProtKB=Q13045</td><td>phosphatidylinositol-4,5-bisphosphate binding</td></tr>
<tr><td>GO:0072330</td><td>HUMAN|HGNC=16369|UniProtKB=Q99497</td><td>monocarboxylic acid biosynthetic process</td></tr>
<tr><td>PC00083</td><td>HUMAN|HGNC=10632|UniProtKB=P13501</td><td>cytokine</td></tr>
<tr><td>GO:2001243</td><td>HUMAN|HGNC=992|UniProtKB=Q07817,HUMAN|HGNC=11283|UniProtKB=P12931</td><td>negative regulation of intrinsic apoptotic signaling pathway</td></tr>
<tr><td>GO:0030036</td><td>HUMAN|HGNC=6111|UniProtKB=Q13576,HUMAN|HGNC=2067|UniProtKB=O00151,HUMAN|HGNC=17468|UniProtKB=Q96HC4,HUMAN|HGNC=22958|UniProtKB=Q9NR12,HUMAN|HGNC=11126|UniProtKB=P53814</td><td>actin cytoskeleton organization</td></tr>
</table>

<table>
<tr><td>GO:0071345</td><td>HUMAN|HGNC=7183|UniProtKB=P41218</td><td>cellular response to cytokine stimulus</td></tr>
<tr><td>GO:0006915</td><td>HUMAN|HGNC=1504|UniProtKB=P42574,HUMAN|HGNC=19388|UniProtKB=P49593,HUMAN|HGNC=6766|UniProtKB=Q8WXG6,HUMAN|HGNC=533|UniProtKB=P04083,HUMAN|HGNC=7850|UniProtKB=P22392,HUMAN|HGNC=1507|UniProtKB=P55212,HUMAN|HGNC=19986|UniProtKB=P99999</td><td>apoptotic process</td></tr>
<tr><td>GO:0010638</td><td>HUMAN|HGNC=17865|UniProtKB=Q96A26,HUMAN|HGNC=1050|UniProtKB=P55957,HUMAN|HGNC=533|UniProtKB=P04083</td><td>positive regulation of organelle organization</td></tr>
<tr><td>GO:0090148</td><td>HUMAN|HGNC=535|UniProtKB=P50995,HUMAN|HGNC=15848|UniProtKB=Q14141,HUMAN|HGNC=2973|UniProtKB=O00429,HUMAN|HGNC=2713|UniProtKB=O75935,HUMAN|HGNC=2974|UniProtKB=P50570,HUMAN|HGNC=9164|UniProtKB=Q99719,HUMAN|HGNC=1717|UniProtKB=Q16181,HUMAN|HGNC=10252|UniProtKB=O75116,HUMAN|HGNC=29125|UniProtKB=Q9UQ16,HUMAN|HGNC=7729|UniProtKB=Q15019</td><td>membrane fission</td></tr>
<tr><td>GO:0018108</td><td>HUMAN|HGNC=6171|UniProtKB=Q08881,HUMAN|HGNC=4037|UniProtKB=P06241,HUMAN|HGNC=12841|UniProtKB=P07947,HUMAN|HGNC=11283|UniProtKB=P12931,HUMAN|HGNC=6735|UniProtKB=P07948</td><td>peptidyl-tyrosine phosphorylation</td></tr>
<tr><td>GO:0043414</td><td>HUMAN|HGNC=4861|UniProtKB=Q9NRZ9</td><td>macromolecule methylation</td></tr>
<tr><td>GO:0005543</td><td>HUMAN|HGNC=23186|UniProtKB=Q14677</td><td>phospholipid binding</td></tr>
<tr><td>GO:0042555</td><td>HUMAN|HGNC=26200|UniProtKB=Q9H668</td><td>MCM complex</td></tr>
<tr><td>GO:0071819</td><td>HUMAN|HGNC=31797|UniProtKB=P0C7V6,HUMAN|HGNC=12347|UniProtKB=Q9Y4A5</td><td>DUBm complex</td></tr>
<tr><td>GO:0051307</td><td>HUMAN|HGNC=12401|UniProtKB=P33981</td><td>meiotic chromosome separation</td></tr>
<tr><td>GO:0007274</td><td>HUMAN|HGNC=16831|UniProtKB=O95153</td><td>neuromuscular synaptic transmission</td></tr>
<tr><td>GO:0008543</td><td>HUMAN|HGNC=15527|UniProtKB=Q86Z14</td><td>fibroblast growth factor receptor signaling pathway</td></tr>
<tr><td>PC00069</td><td>HUMAN|HGNC=3011|UniProtKB=Q07507,HUMAN|HGNC=11771|UniProtKB=Q15582,HUMAN|HGNC=1682|UniProtKB=Q08722</td><td>cell adhesion molecule</td></tr>
<tr><td>GO:0098800</td><td>HUMAN|HGNC=6047|UniProtKB=Q16891,HUMAN|HGNC=7687|UniProtKB=O00483,HUMAN|HGNC=23526|UniProtKB=Q8NE86,HUMAN|HGNC=2265|UniProtKB=P13073,HUMAN|HGNC=2267|UniProtKB=P20674</td><td>inner mitochondrial membrane protein complex</td></tr>
<tr><td>GO:0035556</td><td>HUMAN|HGNC=9414|UniProtKB=Q13976,HUMAN|HGNC=3757|UniProtKB=O75955,HUMAN|HGNC=9406|UniProtKB=Q16513,HUMAN|HGNC=9380|UniProtKB=P17612,HUMAN|HGNC=9395|UniProtKB=P05771,HUMAN|HGNC=18994|UniProtKB=Q8TDC3,HUMAN|HGNC=6876|UniProtKB=Q16539,HUMAN|HGNC=2850|UniProtKB=Q9Y6T7,HUMAN|HGNC=10432|UniProtKB=P51812,HUMAN|HGNC=30410|UniProtKB=Q9BXA6,HUMAN|HGNC=6529|UniProtKB=Q13094,HUMAN|HGNC=20292|UniProtKB=Q9Y376,HUMAN|HGNC=6871|UniProtKB=P28482,HUMAN|HGNC=19825|UniProtKB=Q6SA08,HUMAN|HGNC=9381|UniProtKB=P22694</td><td>intracellular signal transduction</td></tr>
<tr><td>GO:0002685</td><td>HUMAN|HGNC=533|UniProtKB=P04083</td><td>regulation of leukocyte migration</td></tr>
<tr><td>GO:0030134</td><td>HUMAN|HGNC=21354|UniProtKB=Q86XR7,HUMAN|HGNC=24251|UniProtKB=Q9Y3A6,HUMAN|HGNC=6631|UniProtKB=P49257,HU</td><td>COPII-coated ER to Golgi transport vesicle</td></tr>
</table>

	MAN|HGNC=16998|UniProtKB=P49755,HUMAN|HGNC=16996|UniProtKB=Q15363,HUMAN|HGNC=24878|UniProtKB=Q9BVK6	
GO:0010035	HUMAN|HGNC=11179|UniProtKB=P00441,HUMAN|HGNC=9353|UniProtKB=P32119,HUMAN|HGNC=1516|UniProtKB=P04040,HUMAN|HGNC=4553|UniProtKB=P07203,HUMAN|HGNC=533|UniProtKB=P04083,HUMAN|HGNC=9352|UniProtKB=Q06830	response to inorganic substance
GO:0071897	HUMAN|HGNC=26200|UniProtKB=Q9H668,HUMAN|HGNC=7326|UniProtKB=P20585	DNA biosynthetic process
GO:0070925	HUMAN|HGNC=15474|UniProtKB=Q9Y566,HUMAN|HGNC=14294|UniProtKB=Q9BYB0,HUMAN|HGNC=9788|UniProtKB=P51149	organelle assembly
GO:0035032	HUMAN|HGNC=19692|UniProtKB=Q96F24	phosphatidylinositol 3-kinase complex, class III
GO:0015629	HUMAN|HGNC=2713|UniProtKB=O75935,HUMAN|HGNC=11873|UniProtKB=Q9NYL9,HUMAN|HGNC=12690|UniProtKB=P09327,HUMAN|HGNC=2712|UniProtKB=Q13561,HUMAN|HGNC=3382|UniProtKB=Q08495,HUMAN|HGNC=14653|UniProtKB=Q9HBI1,HUMAN|HGNC=3750|UniProtKB=Q13045,HUMAN|HGNC=1491|UniProtKB=P47756,HUMAN|HGNC=24636|UniProtKB=Q9UHB6	actin cytoskeleton
GO:0001934	HUMAN|HGNC=11766|UniProtKB=P01137	positive regulation of protein phosphorylation
GO:0005730	HUMAN|HGNC=25758|UniProtKB=Q8TED0,HUMAN|HGNC=7183|UniProtKB=P41218,HUMAN|HGNC=226|UniProtKB=P78563,HUMAN|HGNC=7910|UniProtKB=P06748	nucleolus
GO:0070509	HUMAN|HGNC=23526|UniProtKB=Q8NE86	calcium ion import
GO:0051649	HUMAN|HGNC=16051|UniProtKB=Q8TEW0,HUMAN|HGNC=11491|UniProtKB=P43405	establishment of localization in cell
GO:0016922	HUMAN|HGNC=7670|UniProtKB=Q9Y6Q9	nuclear receptor binding
GO:1902532	HUMAN|HGNC=18233|UniProtKB=Q8NDB2,HUMAN|HGNC=20250|UniProtKB=Q96IF1	negative regulation of intracellular signal transduction
GO:1902600	HUMAN|HGNC=800|UniProtKB=P50993	proton transmembrane transport
GO:0032008	HUMAN|HGNC=17955|UniProtKB=O43504,HUMAN|HGNC=15606|UniProtKB=Q9UHA4	positive regulation of TOR signaling
GO:0015079	HUMAN|HGNC=800|UniProtKB=P50993,HUMAN|HGNC=806|UniProtKB=P54709	potassium ion transmembrane transporter activity
GO:0000777	HUMAN|HGNC=24054|UniProtKB=Q8NG31	condensed chromosome kinetochore
GO:0002183	HUMAN|HGNC=3282|UniProtKB=P60842,HUMAN|Gene=EIF2S3L|UniProtKB=Q2VIR3,HUMAN|HGNC=3285|UniProtKB=P23588	cytoplasmic translational initiation
GO:0000981	HUMAN|HGNC=33108|UniProtKB=Q6NX45,HUMAN|HGNC=14008|UniProtKB=Q9BW11,HUMAN|HGNC=7803|UniProtKB=Q12986,HUMAN|HGNC=18411|UniProtKB=Q3KNW1,HUMAN|HGNC=14010|UniProtKB=Q8IWI9,HUMAN|HGNC=11364|UniProtKB=P40763,HUMAN|HGNC=6702|UniProtKB=Q32MZ4,HUMAN|HGNC=15935|UniProtKB=Q9H4I2,HUMAN|HGNC=29084|UniProtKB=O75123,HUMAN|HGNC=26427|UniProtKB=Q96ND8,HUMAN|HGNC=17801|UniProtKB=Q8NHY6,HUMAN|HGNC=11594|UniProtKB=Q96SF7,HUMAN|HGNC=9459|UniProtKB=Q92786,HUMAN|HGNC=9866|UniProtKB=P13631,HUMAN|HGNC=51815|UniProtKB=A0A1W2PPM1,HUMAN|HGNC=3815|UniProtKB=Q12951,HUMAN|HGNC=7967|UniP	DNA-binding transcription factor activity, RNA polymerase II-specific

<table>
<tr><td></td><td>rotKB=Q96RI1,HUMAN|HGNC=11590|UniProtKB=Q16650,HUMAN|HGNC=7547|UniProtKB=P10243</td><td></td></tr>
<tr><td>GO:0009913</td><td>HUMAN|HGNC=1504|UniProtKB=P42574,HUMAN|HGNC=533|UniProtKB=P04083</td><td>epidermal cell differentiation</td></tr>
<tr><td>GO:0016925</td><td>HUMAN|HGNC=11124|UniProtKB=P55854,HUMAN|HGNC=11125|UniProtKB=P61956</td><td>protein sumoylation</td></tr>
<tr><td>GO:0004930</td><td>HUMAN|HGNC=4472|UniProtKB=Q14330,HUMAN|HGNC=3540|UniProtKB=Q96RI0</td><td>G protein-coupled receptor activity</td></tr>
<tr><td>GO:0042802</td><td>HUMAN|HGNC=8876|UniProtKB=P17858,HUMAN|HGNC=2520|UniProtKB=Q9NRF8,HUMAN|HGNC=8878|UniProtKB=Q01813</td><td>identical protein binding</td></tr>
<tr><td>GO:0042552</td><td>HUMAN|HGNC=2479|UniProtKB=O76096,HUMAN|HGNC=9086|UniProtKB=P60201</td><td>myelination</td></tr>
<tr><td>GO:0042593</td><td>HUMAN|HGNC=4922|UniProtKB=P19367,HUMAN|HGNC=6091|UniProtKB=P06213</td><td>glucose homeostasis</td></tr>
<tr><td>GO:0009451</td><td>HUMAN|HGNC=226|UniProtKB=P78563</td><td>RNA modification</td></tr>
<tr><td>GO:0043949</td><td>HUMAN|HGNC=9391|UniProtKB=P13861,HUMAN|HGNC=4395|UniProtKB=P19086,HUMAN|HGNC=4384|UniProtKB=P63096,HUMAN|HGNC=10608|UniProtKB=P47872,HUMAN|HGNC=4381|UniProtKB=Q14344,HUMAN|HGNC=4387|UniProtKB=P08754,HUMAN|HGNC=9392|UniProtKB=P31323,HUMAN|HGNC=4385|UniProtKB=P04899,HUMAN|HGNC=4393|UniProtKB=P11488,HUMAN|HGNC=4390|UniProtKB=P50148</td><td>regulation of cAMP-mediated signaling</td></tr>
<tr><td>GO:0019902</td><td>HUMAN|HGNC=7618|UniProtKB=O14974</td><td>phosphatase binding</td></tr>
<tr><td>GO:0012505</td><td>HUMAN|HGNC=16089|UniProtKB=O15173,HUMAN|HGNC=16524|UniProtKB=P61106,HUMAN|HGNC=11326|UniProtKB=P51571,HUMAN|HGNC=11429|UniProtKB=O75558,HUMAN|HGNC=19983|UniProtKB=Q86YS6,HUMAN|HGNC=16090|UniProtKB=O00264,HUMAN|HGNC=9758|UniProtKB=P62820,HUMAN|HGNC=13921|UniProtKB=O95870,HUMAN|HGNC=18673|UniProtKB=Q96JB5,HUMAN|HGNC=18370|UniProtKB=Q9H0U4,HUMAN|HGNC=30384|UniProtKB=Q9UMX5</td><td>endomembrane system</td></tr>
</table>
