# Supplementary material for: Platelet-Rich Fibrin Increases BMP2 Expression in Oral Fibroblasts via Activation of TGF-β Signaling
Source: Int J Mol Sci. 2021 Jul 25;22(15):7935. doi: 10.3390/ijms22157935 (PMC8347014; doi:10.3390/ijms22157935)
Supplement: Supplementary file 1 [file ijms-22-07935-s001.zip › Supplementary Table 3.pdf]

Table S3: Revigo analysis reduced the visualize gene ontology to 350 GO term that are listed.

TermID	Name	Frequency	PlotX	PlotY	LogSize
GO:0007190	"activation of adenylate cyclase activity"	0.003	-6.602	-3.297	2.839
GO:0019882	"antigen processing and presentation"	0.025	1.579	-0.073	3.756
GO:0043473	"pigmentation"	0.029	2.153	-1.686	3.82
GO:0048771	"tissue remodeling"	0.019	2.849	-5.723	3.642
GO:1905324	"telomere-telomerase complex assembly"	0	6.445	-0.588	0.903
GO:0045197	"establishment or maintenance of epithelial cell apical/basal polarity"	0.009	2.926	-2.279	3.283
GO:0051402	"neuron apoptotic process"	0.009	-0.229	-1.143	3.314
GO:0006458	"'de novo' protein folding"	0.028	-5.085	-6.046	3.793
GO:0006509	"membrane protein ectodomain proteolysis"	0.007	5.512	4.945	3.186
GO:0006312	"mitotic recombination"	0.013	-0.703	7.377	3.475
GO:0060998	"regulation of dendritic spine development"	0.007	-3.507	0.834	3.167
GO:0090559	"regulation of membrane permeability"	0.013	-5.788	-2.58	3.458
GO:0051445	"regulation of meiotic cell cycle"	0.021	-5.543	1.523	3.665
GO:0043949	"regulation of cAMP-mediated signaling"	0.005	-4.186	-0.388	3.042
GO:0050818	"regulation of coagulation"	0.018	-7.26	-1.567	3.609
GO:0051642	"centrosome localization"	0.007	-2.69	-5.74	3.181
GO:1902414	"protein localization to cell junction"	0.016	-2.532	-6.567	3.548
GO:0015871	"choline transport"	0.01	-0.644	-6.915	3.362
GO:0010821	"regulation of mitochondrion organization"	0.027	-7.322	1.61	3.78
GO:0060968	"regulation of gene silencing"	0.027	-7.57	0.774	3.789
GO:0007041	"lysosomal transport"	0.022	0.003	-6.703	3.699
GO:0008333	"endosome to lysosome transport"	0.01	null	null	3.337
GO:0006622	"protein targeting to lysosome"	0.005	null	null	3.012
GO:0000380	"alternative mRNA splicing	via spliceosome"	0.005	-0.499	7.194
GO:0072376	"protein activation cascade"	0.003	-3.114	6.348	2.868

GO:0090148	"membrane fission"	0.006	5.67	-1.409	3.156
GO:0032355	"response to estradiol"	0.009	-4.701	6.431	3.301
GO:0046456	"icosanoid biosynthetic process"	0.013	1.907	3.078	3.473
GO:0006693	"prostaglandin metabolic process"	0.015	null	null	3.522
GO:0035195	"gene silencing by miRNA"	0.02	-7.535	-0.166	3.66
GO:0032438	"melanosome organization"	0.004	5.975	-2.005	2.974
GO:0060261	"positive regulation of transcription initiation from RNA polymerase II promoter"	0.014	-7.534	0.451	3.503
GO:0045899	"positive regulation of RNA polymerase II transcription preinitiation complex assembly"	0.01	null	null	3.348
GO:0007528	"neuromuscular junction development"	0.011	6.194	-2.076	3.385
GO:0007044	"cell-substrate junction assembly"	0.007	null	null	3.182
GO:0097061	"dendritic spine organization"	0.004	null	null	2.966
GO:0018107	"peptidyl-threonine phosphorylation"	0.019	4.788	5.198	3.637
GO:0038202	"TORC1 signaling"	0.003	-5.49	4.162	2.86
GO:0034142	"toll-like receptor 4 signaling pathway"	0.003	-5.342	4.208	2.892
GO:0019934	"cGMP-mediated signaling"	0.003	-5.592	4.194	2.81
GO:0048016	"inositol phosphate-mediated signaling"	0.01	null	null	3.332
GO:0019933	"cAMP-mediated signaling"	0.007	null	null	3.176
GO:0042144	"vacuole fusion	non-autophagic"	0.006	6.092	-1.928
GO:0099500	"vesicle fusion to plasma membrane"	0.002	null	null	2.621
GO:0007040	"lysosome organization"	0.021	null	null	3.673
GO:0007259	"receptor signaling pathway via JAK-STAT"	0.007	-5.606	3.87	3.212
GO:0070371	"ERK1 and ERK2 cascade"	0.005	-5.247	4.034	3.017
GO:0032640	"tumor necrosis factor production"	0	3.088	-4.26	1
GO:0030204	"chondroitin sulfate metabolic process"	0.007	4.984	4.533	3.217
GO:0050650	"chondroitin sulfate proteoglycan biosynthetic process"	0.008	null	null	3.255
GO:0038061	"NIK/NF-kappaB signaling"	0.007	-5.467	3.939	3.187
GO:0007200	"phospholipase C-activating G protein-coupled receptor signaling pathway"	0.015	-5.743	3.677	3.524

GO:0043491	"protein kinase B signaling"	0.008	-5.206	3.861	3.238
GO:0000963	"mitochondrial RNA processing"	0.011	-0.369	7.144	3.403
GO:0035329	"hippo signaling"	0.01	-5.342	3.809	3.331
GO:0000729	"DNA double-strand break processing"	0.007	-2.587	6.162	3.183
GO:0007249	"I-kappaB kinase/NF-kappaB signaling"	0.02	-5.445	3.613	3.652
GO:0033962	"P-body assembly"	0.007	5.995	-0.927	3.182
GO:0007039	"protein catabolic process in the vacuole"	0.003	5.563	4.174	2.754
GO:0032088	"negative regulation of NF-kappaB transcription factor activity"	0.016	-7.317	-0.668	3.558
GO:0035418	"protein localization to synapse"	0.011	-2.673	-6.531	3.398
GO:0098969	"neurotransmitter receptor transport to postsynaptic membrane"	0.002	null	null	2.732
GO:0097120	"receptor localization to synapse"	0.008	null	null	3.269
GO:0034394	"protein localization to cell surface"	0.006	-2.709	-6.101	3.14
GO:0001508	"action potential"	0.015	-5.981	-2.516	3.523
GO:0034381	"plasma lipoprotein particle clearance"	0.003	-2.101	-1.988	2.812
GO:0001816	"cytokine production"	0.004	3.128	-4.021	2.953
GO:0045761	"regulation of adenylate cyclase activity"	0.008	-6.721	-2.901	3.276
GO:0045104	"intermediate filament cytoskeleton organization"	0.024	5.947	-1.709	3.728
GO:0043113	"receptor clustering"	0.008	-2.611	-6.173	3.277
GO:0060191	"regulation of lipase activity"	0.01	-6.629	-2.869	3.372
GO:0007586	"digestion"	0.02	2.878	-5.822	3.66
GO:0034501	"protein localization to kinetochore"	0.01	-2.531	-6.143	3.358
GO:0019985	"translesion synthesis"	0.02	-1.916	5.919	3.654
GO:0031098	"stress-activated protein kinase signaling cascade"	0.021	-5.069	3.723	3.673
GO:0006301	"postreplication repair"	0.03	-2.397	6.096	3.835
GO:0010769	"regulation of cell morphogenesis involved in differentiation"	0.015	-3.884	0.94	3.518
GO:0042475	"odontogenesis of dentin-containing tooth"	0.012	3.67	-5.319	3.428
GO:0061041	"regulation of wound healing"	0.025	-4.617	-0.699	3.746

GO:0030195	"negative regulation of blood coagulation"	0.008	null	null	3.248
GO:0090303	"positive regulation of wound healing"	0.01	null	null	3.362
GO:0008156	"negative regulation of DNA replication"	0.029	-7.256	0.087	3.812
GO:0043277	"apoptotic cell clearance"	0.008	0.025	-6.083	3.272
GO:0001708	"cell fate specification"	0.027	4.38	-5.167	3.786
GO:0010518	"positive regulation of phospholipase activity"	0.007	-6.801	-2.939	3.175
GO:0007202	"activation of phospholipase C activity"	0.003	null	null	2.836
GO:0010517	"regulation of phospholipase activity"	0.008	null	null	3.247
GO:0031397	"negative regulation of protein ubiquitination"	0.017	-7.43	0.094	3.572
GO:0000038	"very long-chain fatty acid metabolic process"	0.021	4.581	2.338	3.674
GO:0042092	"type 2 immune response"	0.001	-3.542	5.662	2.354
GO:0070555	"response to interleukin-1"	0.017	-4.752	6.351	3.577
GO:0042058	"regulation of epidermal growth factor receptor signaling pathway"	0.013	-4.428	-0.212	3.476
GO:0032456	"endocytic recycling"	0.022	-1.811	-5.655	3.698
GO:0032511	"late endosome to vacuole transport via multivesicular body sorting pathway"	0.026	null	null	3.772
GO:0043328	"protein transport to vacuole involved in ubiquitin-dependent protein catabolic process via the multivesicular body sorting pathway"	0.017	null	null	3.574
GO:0002040	"sprouting angiogenesis"	0.015	3.461	-5.554	3.529
GO:0043951	"negative regulation of cAMP-mediated signaling"	0.003	-4.354	-0.559	2.753
GO:0048814	"regulation of dendrite morphogenesis"	0.01	-4.919	1.316	3.353
GO:0045055	"regulated exocytosis"	0.031	-0.219	-5.942	3.838
GO:0017156	"calcium-ion regulated exocytosis"	0.012	null	null	3.414
GO:0007224	"smoothened signaling pathway"	0.024	-5.291	3.489	3.74
GO:0071218	"cellular response to misfolded protein"	0.019	-4.5	6.195	3.633
GO:0061462	"protein localization to lysosome"	0.007	-2.623	-6.05	3.22
GO:1905349	"ciliary transition zone assembly"	0	6.268	-0.666	1.813
GO:0042552	"myelination"	0.023	3.445	-5.331	3.706

GO:0006636	"unsaturated fatty acid biosynthetic process"	0.025	3.192	3.015	3.749
GO:0061512	"protein localization to cilium"	0.011	-2.531	-6.04	3.377
GO:0097712	"vesicle targeting	trans-Golgi to periciliary membrane compartment"	0	1.352	-4.237	
GO:0030307	"positive regulation of cell growth"	0.027	-7.164	1.317	3.778
GO:0002377	"immunoglobulin production"	0.005	1.412	1.375	3.064
GO:0010324	"membrane invagination"	0.014	6.316	-1.859	3.488
GO:0007193	"adenylate cyclase-inhibiting G protein-coupled receptor signaling pathway"	0.015	-5.699	3.661	3.534
GO:0007189	"adenylate cyclase-activating G protein-coupled receptor signaling pathway"	0.028	null	null	3.792
GO:0002440	"production of molecular mediator of immune response"	0.005	1.312	1.47	3.091
GO:0001705	"ectoderm formation"	0.001	3.577	-5.601	2.201
GO:0001706	"endoderm formation"	0.01	null	null	3.372
GO:0001707	"mesoderm formation"	0.015	null	null	3.521
GO:0070509	"calcium ion import"	0.018	-0.551	-6.834	3.606
GO:0006851	"mitochondrial calcium ion transmembrane transport"	0.014	null	null	3.487
GO:0060401	"cytosolic calcium ion transport"	0.031	null	null	3.838
GO:0044380	"protein localization to cytoskeleton"	0.014	-2.454	-6.04	3.503
GO:0061844	"antimicrobial humoral immune response mediated by antimicrobial peptide"	0.007	-3.695	5.583	3.171
GO:0006956	"complement activation"	0.023	null	null	3.722
GO:0002429	"immune response-activating cell surface receptor signaling pathway"	0.03	null	null	3.829
GO:0019731	"antibacterial humoral response"	0.006	null	null	3.137
GO:0050853	"B cell receptor signaling pathway"	0.013	null	null	3.465
GO:0050852	"T cell receptor signaling pathway"	0.014	null	null	3.497
GO:0001889	"liver development"	0.026	3.49	-5.473	3.77
GO:0045124	"regulation of bone resorption"	0.006	-6.542	-2.131	3.144
GO:0034067	"protein localization to Golgi apparatus"	0.016	-2.437	-6.128	3.55

GO:0099601	"regulation of neurotransmitter receptor activity"	0.014	-5.026	-0.803	3.486
GO:2000311	"regulation of AMPA receptor activity"	0.004	null	null	2.935
GO:0070979	"protein K11-linked ubiquitination"	0.008	4.802	5.872	3.246
GO:0070936	"protein K48-linked ubiquitination"	0.012	null	null	3.43
GO:0070534	"protein K63-linked ubiquitination"	0.018	null	null	3.606
GO:0010824	"regulation of centrosome duplication"	0.012	-7.395	1.804	3.437
GO:0042407	"cristae formation"	0.013	6.189	-1.726	3.474
GO:0090288	"negative regulation of cellular response to growth factor stimulus"	0.024	-5.125	-0.365	3.74
GO:0030514	"negative regulation of BMP signaling pathway"	0.015	null	null	3.523
GO:0030512	"negative regulation of transforming growth factor beta receptor signaling pathway"	0.018	null	null	3.611
GO:0033522	"histone H2A ubiquitination"	0.008	5.689	0.407	3.271
GO:0000082	"G1/S transition of mitotic cell cycle"	0.028	-5.043	-5.655	3.805
GO:0007091	"metaphase/anaphase transition of mitotic cell cycle"	0.004	null	null	2.934
GO:0014066	"regulation of phosphatidylinositol 3-kinase signaling"	0.017	-4.536	-0.242	3.581
GO:0070328	"triglyceride homeostasis"	0.007	-5.834	-2.787	3.188
GO:0042632	"cholesterol homeostasis"	0.018	null	null	3.614
GO:0002718	"regulation of cytokine production involved in immune response"	0.014	-7.38	-1.082	3.511
GO:1901224	"positive regulation of NIK/NF-kappaB signaling"	0.011	-4.633	-0.299	3.374
GO:0008277	"regulation of G protein-coupled receptor signaling pathway"	0.029	-4.71	-0.072	3.812
GO:0072676	"lymphocyte migration"	0.009	-0.865	-4.535	3.299
GO:1990266	"neutrophil migration"	0.012	null	null	3.449
GO:0071621	"granulocyte chemotaxis"	0.012	null	null	3.447
GO:0002449	"lymphocyte mediated immunity"	0.026	1.527	-0.118	3.767
GO:0002456	"T cell mediated immunity"	0.003	null	null	2.859
GO:0030278	"regulation of ossification"	0.025	-7.221	-1.463	3.744
GO:0032418	"lysosome localization"	0.009	-2.597	-5.71	3.306
GO:0034551	"mitochondrial respiratory chain complex III assembly"	0.011	5.98	-1.053	3.401

GO:0007097	"nuclear migration"	0.009	-2.458	-5.675	3.284
GO:0051896	"regulation of protein kinase B signaling"	0.027	-4.671	-0.135	3.781
GO:0006624	"vacuolar protein processing"	0.003	4.494	4.688	2.846
GO:0097192	"extrinsic apoptotic signaling pathway in absence of ligand"	0.005	-4.84	3.708	3.041
GO:0008625	"extrinsic apoptotic signaling pathway via death domain receptors"	0.005	null	null	3.062
GO:0006584	"catecholamine metabolic process"	0.01	-1.009	7.726	3.365
GO:0000266	"mitochondrial fission"	0.02	6.098	-1.704	3.654
GO:0007019	"microtubule depolymerization"	0.004	5.739	-1.856	2.997
GO:0030042	"actin filament depolymerization"	0.022	null	null	3.686
GO:0030041	"actin filament polymerization"	0.023	null	null	3.715
GO:0030282	"bone mineralization"	0.01	3.701	-5.385	3.363
GO:0048741	"skeletal muscle fiber development"	0.01	3.72	-5.281	3.372
GO:0045214	"sarcomere organization"	0.01	null	null	3.353
GO:0030239	"myofibril assembly"	0.017	null	null	3.572
GO:0070997	"neuron death"	0.011	-0.169	-1.114	3.386
GO:0006123	"mitochondrial electron transport	cytochrome c to oxygen"	0.006	6.527	3.192
GO:0006122	"mitochondrial electron transport	ubiquinol to cytochrome c"	0.024	null	null
GO:0048194	"Golgi vesicle budding"	0.003	4.294	-2.678	2.754
GO:0090263	"positive regulation of canonical Wnt signaling pathway"	0.026	-4.932	-0.133	3.77
GO:0051306	"mitotic sister chromatid separation"	0.003	5.622	-1.738	2.875
GO:0051307	"meiotic chromosome separation"	0.012	null	null	3.421
GO:0045144	"meiotic sister chromatid segregation"	0.008	null	null	3.254
GO:0007129	"homologous chromosome pairing at meiosis"	0.019	null	null	3.63
GO:0045446	"endothelial cell differentiation"	0.015	4.329	-5.2	3.518
GO:0009913	"epidermal cell differentiation"	0.03	null	null	3.834
GO:0043124	"negative regulation of I-kappaB kinase/NF-kappaB signaling"	0.009	-4.77	-0.431	3.298
GO:0043123	"positive regulation of I-kappaB kinase/NF-kappaB signaling"	0.03	null	null	3.826

GO:0008088	"axo-dendritic transport"	0.012	-1.998	-5.467	3.426
GO:0072384	"organelle transport along microtubule"	0.018	null	null	3.6
GO:0098840	"protein transport along microtubule"	0.03	null	null	3.83
GO:0035735	"intraciliary transport involved in cilium assembly"	0.006	null	null	3.11
GO:0042073	"intraciliary transport"	0.028	null	null	3.806
GO:0034453	"microtubule anchoring"	0.012	5.733	-1.815	3.443
GO:0002821	"positive regulation of adaptive immune response"	0.016	-4.828	-0.728	3.568
GO:0043542	"endothelial cell migration"	0.013	1.222	-5.829	3.459
GO:0048709	"oligodendrocyte differentiation"	0.014	3.646	-5.237	3.499
GO:0014032	"neural crest cell development"	0.022	3.648	-5.427	3.691
GO:0033627	"cell adhesion mediated by integrin"	0.005	2.152	-2.985	3.021
GO:0031638	"zymogen activation"	0.01	4.354	4.816	3.348
GO:0051897	"positive regulation of protein kinase B signaling"	0.017	-4.796	-0.227	3.575
GO:2000045	"regulation of G1/S transition of mitotic cell cycle"	0.027	null	null	3.778
GO:0048488	"synaptic vesicle endocytosis"	0.009	null	null	3.301
GO:0016358	"dendrite development"	0.021	null	null	3.684
GO:0006706	"steroid catabolic process"	0.007	null	null	3.185
GO:2001242	"regulation of intrinsic apoptotic signaling pathway"	0.029	null	null	3.821
GO:0007159	"leukocyte cell-cell adhesion"	0.009	null	null	3.287
GO:0035567	"non-canonical Wnt signaling pathway"	0.007	null	null	3.207
GO:0007098	"centrosome cycle"	0.03	null	null	3.836
GO:0030856	"regulation of epithelial cell differentiation"	0.024	null	null	3.736
GO:0048813	"dendrite morphogenesis"	0.012	null	null	3.445
GO:0008045	"motor neuron axon guidance"	0.009	null	null	3.326
GO:0030072	"peptide hormone secretion"	0.012	null	null	3.446
GO:0018279	"protein N-linked glycosylation via asparagine"	0.013	null	null	3.449
GO:0007004	"telomere maintenance via telomerase"	0.009	null	null	3.328

GO:0002460	"adaptive immune response based on somatic recombination of immune receptors built from immunoglobulin superfamily domains"	0.03	null	null	3.835
GO:0051208	"sequestering of calcium ion"	0.001	null	null	2.408
GO:0051279	"regulation of release of sequestered calcium ion into cytosol"	0.011	null	null	3.396
GO:0072583	"clathrin-dependent endocytosis"	0.026	null	null	3.765
GO:0042177	"negative regulation of protein catabolic process"	0.022	null	null	3.702
GO:0051315	"attachment of mitotic spindle microtubules to kinetochore"	0.011	null	null	3.41
GO:0007266	"Rho protein signal transduction"	0.02	null	null	3.663
GO:0010833	"telomere maintenance via telomere lengthening"	0.015	null	null	3.528
GO:0060070	"canonical Wnt signaling pathway"	0.021	null	null	3.677
GO:0030301	"cholesterol transport"	0.017	null	null	3.58
GO:0007173	"epidermal growth factor receptor signaling pathway"	0.01	null	null	3.334
GO:0030316	"osteoclast differentiation"	0.008	null	null	3.256
GO:0006998	"nuclear envelope organization"	0.022	null	null	3.692
GO:0048311	"mitochondrion distribution"	0.019	null	null	3.629
GO:0051123	"RNA polymerase II preinitiation complex assembly"	0.009	null	null	3.33
GO:0000289	"nuclear-transcribed mRNA poly(A) tail shortening"	0.016	null	null	3.548
GO:0008637	"apoptotic mitochondrial changes"	0.01	null	null	3.354
GO:0032210	"regulation of telomere maintenance via telomerase"	0.014	null	null	3.495
GO:0097352	"autophagosome maturation"	0.008	null	null	3.246
GO:0031532	"actin cytoskeleton reorganization"	0.021	null	null	3.67
GO:0030866	"cortical actin cytoskeleton organization"	0.023	null	null	3.705
GO:0002218	"activation of innate immune response"	0.011	null	null	3.386
GO:0007274	"neuromuscular synaptic transmission"	0.005	null	null	3.084
GO:0031507	"heterochromatin assembly"	0.022	null	null	3.689
GO:0035249	"synaptic transmission	glutamatergic "	0.006	null	null
GO:0032007	"negative regulation of TOR signaling"	0.023	null	null	3.721

GO:0045840	"positive regulation of mitotic nuclear division"	0.014	null	null	3.494
GO:0043984	"histone H4-K16 acetylation"	0.005	null	null	3.067
GO:0097191	"extrinsic apoptotic signaling pathway"	0.015	null	null	3.534
GO:0002062	"chondrocyte differentiation"	0.014	null	null	3.504
GO:0070527	"platelet aggregation"	0.006	null	null	3.114
GO:0000727	"double-strand break repair via break-induced replication"	0.011	null	null	3.395
GO:0050830	"defense response to Gram-positive bacterium"	0.015	null	null	3.539
GO:2001259	"positive regulation of cation channel activity"	0.015	null	null	3.525
GO:0050829	"defense response to Gram-negative bacterium"	0.015	null	null	3.542
GO:0071108	"protein K48-linked deubiquitination"	0.022	null	null	3.69
GO:0045921	"positive regulation of exocytosis"	0.014	null	null	3.494
GO:0045453	"bone resorption"	0.004	null	null	2.986
GO:0010976	"positive regulation of neuron projection development"	0.018	null	null	3.608
GO:0006695	"cholesterol biosynthetic process"	0.014	null	null	3.497
GO:0007212	"dopamine receptor signaling pathway"	0.01	null	null	3.372
GO:0048675	"axon extension"	0.01	null	null	3.335
GO:0050728	"negative regulation of inflammatory response"	0.021	null	null	3.684
GO:0048255	"mRNA stabilization"	0.008	null	null	3.23
GO:0048259	"regulation of receptor-mediated endocytosis"	0.018	null	null	3.609
GO:0050868	"negative regulation of T cell activation"	0.019	null	null	3.632
GO:0071222	"cellular response to lipopolysaccharide"	0.024	null	null	3.732
GO:0046632	"alpha-beta T cell differentiation"	0.011	null	null	3.379
GO:0046579	"positive regulation of Ras protein signal transduction"	0.014	null	null	3.49
GO:0010811	"positive regulation of cell-substrate adhesion"	0.021	null	null	3.668
GO:0000422	"autophagy of mitochondrion"	0.023	null	null	3.709
GO:0001947	"heart looping"	0.019	null	null	3.623
GO:0030218	"erythrocyte differentiation"	0.028	null	null	3.799

GO:0046651	"lymphocyte proliferation"	0.012	null	null	3.445
GO:0031936	"negative regulation of chromatin silencing"	0.008	null	null	3.256
GO:0008630	"intrinsic apoptotic signaling pathway in response to DNA damage"	0.014	null	null	3.499
GO:0051966	"regulation of synaptic transmission	glutamatergic "	0.007	null	null
GO:2000780	"negative regulation of double-strand break repair"	0.007	null	null	3.224
GO:0046580	"negative regulation of Ras protein signal transduction"	0.017	null	null	3.588
GO:0007043	"cell-cell junction assembly"	0.027	null	null	3.79
GO:0050772	"positive regulation of axonogenesis"	0.012	null	null	3.417
GO:0036158	"outer dynein arm assembly"	0.005	null	null	3.015
GO:0043149	"stress fiber assembly"	0.003	null	null	2.829
GO:0030183	"B cell differentiation"	0.017	null	null	3.575
GO:0051453	"regulation of intracellular pH"	0.026	null	null	3.761
GO:0071356	"cellular response to tumor necrosis factor"	0.028	null	null	3.795
GO:0008584	"male gonad development"	0.02	null	null	3.644
GO:2001237	"negative regulation of extrinsic apoptotic signaling pathway"	0.016	null	null	3.552
GO:0051928	"positive regulation of calcium ion transport"	0.016	null	null	3.566
GO:0031623	"receptor internalization"	0.011	null	null	3.394
GO:0060236	"regulation of mitotic spindle organization"	0.014	null	null	3.485
GO:0006627	"protein processing involved in protein targeting to mitochondrion"	0.016	null	null	3.553
GO:0002708	"positive regulation of lymphocyte mediated immunity"	0.017	null	null	3.587
GO:0042771	"intrinsic apoptotic signaling pathway in response to DNA damage by p53 class mediator"	0.006	null	null	3.121
GO:0051560	"mitochondrial calcium ion homeostasis"	0.017	null	null	3.591
GO:0006896	"Golgi to vacuole transport"	0.015	null	null	3.542
GO:0046464	"acylglycerol catabolic process"	0.007	null	null	3.208
GO:0048489	"synaptic vesicle transport"	0.007	null	null	3.187
GO:0034198	"cellular response to amino acid starvation"	0.016	null	null	3.547

GO:0014065	"phosphatidylinositol 3-kinase signaling"	0.009	null	null	3.323
GO:1990126	"retrograde transport	endosome to plasma membrane"	0.006	null	null
GO:0034446	"substrate adhesion-dependent cell spreading"	0.01	null	null	3.373
GO:0030509	"BMP signaling pathway"	0.018	null	null	3.615
GO:0035023	"regulation of Rho protein signal transduction"	0.029	null	null	3.814
GO:0030199	"collagen fibril organization"	0.01	null	null	3.35
GO:0097193	"intrinsic apoptotic signaling pathway"	0.03	null	null	3.823
GO:0042113	"B cell activation"	0.025	null	null	3.759
GO:0047496	"vesicle transport along microtubule"	0.009	null	null	3.32
GO:0000132	"establishment of mitotic spindle orientation"	0.014	null	null	3.485
GO:0002366	"leukocyte activation involved in immune response"	0.027	null	null	3.788
GO:0050714	"positive regulation of protein secretion"	0.02	null	null	3.647
GO:0046928	"regulation of neurotransmitter secretion"	0.015	null	null	3.533
GO:0060047	"heart contraction"	0.019	null	null	3.625
GO:0007416	"synapse assembly"	0.018	null	null	3.601
GO:0030518	"intracellular steroid hormone receptor signaling pathway"	0.01	null	null	3.333
GO:0007269	"neurotransmitter secretion"	0.029	null	null	3.819
GO:2001243	"negative regulation of intrinsic apoptotic signaling pathway"	0.018	null	null	3.607
GO:0017121	"plasma membrane phospholipid scrambling"	0.019	null	null	3.627
GO:0007179	"transforming growth factor beta receptor signaling pathway"	0.026	null	null	3.765
GO:0051092	"positive regulation of NF-kappaB transcription factor activity"	0.027	null	null	3.787
GO:0008543	"fibroblast growth factor receptor signaling pathway"	0.027	null	null	3.78
GO:0000187	"activation of MAPK activity"	0.022	null	null	3.703
GO:0090307	"mitotic spindle assembly"	0.017	null	null	3.577
GO:2001235	"positive regulation of apoptotic signaling pathway"	0.023	null	null	3.715
GO:0000712	"resolution of meiotic recombination intermediates"	0.01	null	null	3.36

GO:0050806	"positive regulation of synaptic transmission"	0.019	null	null	3.628
GO:0031110	"regulation of microtubule polymerization or depolymerization"	0.027	null	null	3.777
GO:0045954	"positive regulation of natural killer cell mediated cytotoxicity"	0.004	null	null	2.978
GO:0006939	"smooth muscle contraction"	0.013	null	null	3.455
GO:0045582	"positive regulation of T cell differentiation"	0.014	null	null	3.51
GO:2001238	"positive regulation of extrinsic apoptotic signaling pathway"	0.009	null	null	3.316
GO:0042102	"positive regulation of T cell proliferation"	0.015	null	null	3.523
GO:0043407	"negative regulation of MAP kinase activity"	0.023	null	null	3.723
GO:0048199	"vesicle targeting	to	from or within Golgi"	0.014	null	
GO:0051283	"negative regulation of sequestering of calcium ion"	0.017	null	null	3.591
GO:0043001	"Golgi to plasma membrane protein transport"	0.013	null	null	3.451
GO:0046475	"glycerophospholipid catabolic process"	0.013	null	null	3.45
GO:1902808	"positive regulation of cell cycle G1/S phase transition"	0.01	null	null	3.341
GO:0002891	"positive regulation of immunoglobulin mediated immune response"	0.007	null	null	3.169
GO:0002705	"positive regulation of leukocyte mediated immunity"	0.02	null	null	3.647
GO:0048167	"regulation of synaptic plasticity"	0.029	null	null	3.813
GO:0043280	"positive regulation of cysteine-type endopeptidase activity involved in apoptotic process"	0.022	null	null	3.692
GO:0030168	"platelet activation"	0.015	null	null	3.536
GO:0051298	"centrosome duplication"	0.016	null	null	3.56
GO:0030968	"endoplasmic reticulum unfolded protein response"	0.024	null	null	3.741
GO:0002286	"T cell activation involved in immune response"	0.01	null	null	3.359
GO:0051209	"release of sequestered calcium ion into cytosol"	0.017	null	null	3.585
GO:0071456	"cellular response to hypoxia"	0.017	null	null	3.586
GO:0045766	"positive regulation of angiogenesis"	0.026	null	null	3.773
GO:0032869	"cellular response to insulin stimulus"	0.028	null	null	3.794

GO:0060048	"cardiac muscle contraction"	0.011	null	null	3.404
GO:0006919	"activation of cysteine-type endopeptidase activity involved in apoptotic process"	0.014	null	null	3.503
GO:0090277	"positive regulation of peptide hormone secretion"	0.014	null	null	3.492
GO:0051250	"negative regulation of lymphocyte activation"	0.025	null	null	3.75
GO:0030073	"insulin secretion"	0.009	null	null	3.303
GO:0016079	"synaptic vesicle exocytosis"	0.016	null	null	3.559
GO:0042098	"T cell proliferation"	0.007	null	null	3.179
GO:2000042	"negative regulation of double-strand break repair via homologous recombination"	0.005	null	null	3.02
GO:0002695	"negative regulation of leukocyte activation"	0.03	null	null	3.828
